# Characterization and phylogenetic analysis of multiple C2 domain and transmembrane region proteins in maize

**DOI:** 10.1186/s12870-022-03771-x

**Published:** 2022-08-03

**Authors:** Yujun Zhao, Qianqian Qin, Li Chen, Yun Long, Nannan Song, Haiyang Jiang, Weina Si

**Affiliations:** National Engineering Laboratory of Crop Stress Resistance Breeding, School of Life Sciences, Anhui Agricultural University, Hefei, 230036 China

**Keywords:** MCTPs, Phylogenetic analysis, Gene duplication, Expression, Abiotic stress, Maize

## Abstract

**Background:**

Multiple C2 domain and transmembrane region proteins (MCTPs) are evolutionarily conserved and important signaling molecules. However, the MCTP gene family has not been comprehensively analyzed in maize.

**Results:**

In this study, 385 *MCTP* genes were identified in all surveyed 38 species. Moreover, gene duplication mode exploration showed that whole genome duplication (WGD) mainly contributed to the expansion of *MCTP* genes in angiosperms. Phylogeny reconstruction with all surveyed species by the maximum-likelihood (ML) method showed five clades of MCTPs, Clades I to V. Each clade of MCTPs had conservative structures and motifs. Focusing on maize, 17 MCTPs were identified, and a neighborjoining (NJ) phylogenetic tree with only ZmMCTPs was also constructed. As expected, 17 MCTPs showed similar phylogenetic relationships in the neighbor-joining (NJ) tree with those in the maximum-likelihood (ML) tree and could also be divided into five subclades. Moreover, ZmMCTP members in different clades showed specific gene structure, conserved motif, and domain structure compositions. Intriguingly, most *ZmMCTP* genes were intronless. Analyses of isoelectric points (pIs) and grand averages of hydropathicity (GRAVYs) indicated that the N-terminus was more dispersive than the C-terminus. Further tissue-specific expression analysis indicated that duplicated *ZmMCTP* pairs involved in whole genome duplication (WGD) had similar expression trends. Finally, *ZmMCTPs* were transcriptionally altered under diverse abiotic stresses and hormone treatments.

**Conclusions:**

Our results contribute to deciphering the evolutionary history of MCTPs in maize and other plants, facilitating further functional analysis of these factors, and provide a basis for further clarification of the molecular mechanism of stress responses.

**Supplementary Information:**

The online version contains supplementary material available at 10.1186/s12870-022-03771-x.

## Background

Cell to cell communication is essential for the development and differentiation of organisms [1]. In plants, many channels are able to transport a variety of materials, including small molecules (sucrose), macromolecules (proteins) and nucleic acids [2, 3]. Interestingly, during evolution, plants developed cell walls and prevent the transmission of information and substances between cells [4]. Instead, plants evolved the substance and information transmission channel known as plasmodesmata (PD). PD are not only involved in information transfer, but also in material transport, such as in the transport of nutrients between cells [5–7].

Previous studies have shown that PD play an important role in the process of nutrient transport and signal transduction [6, 8]. Among many PD proteins, some multiple C2 domain and transmembrane region proteins (MCTPs) were significantly enriched in PD and in close contact with the endoplasmic reticulum (ER)–plasma membrane (PM) [9]. The loss of function double mutant, *Atmctp3/Atmctp4*, showed defective developmental phenotypes, with reduced intercellular migration and notably altered PD proteome [9]. Recent studies have found that MCTPs were significantly conserved during evolution, with each MCTP containing three to four C2 domains at the N-terminus and one to four transmembrane regions at the C-terminus. MCTPs were important components of intercellular signaling in plants and played a vital role as signaling molecules, mediating the transport process of plant cells [10, 11].

The *MCTP* family was first found in the animal kingdom, in the invertebrate *Caenorhabditis elegans*, and RNAi research has shown that loss of *MCTP* function could inhibit early embryonic development [12]. In *Drosophila melanogaster*, two *MCTPs* were involved in baseline neuronal release and highlights homeostasis plasticity [13]. There are more *MCTP* genes in plants. Sixteen *MCTPs* members were identified in *Arabidopsis thaliana* that regulate flowering, growth and development through cellular transport pathways, including the *QUIRKY (QKY)* and *FT-INTERACTING PROTEIN1 (FTIP1)*, which had the functions of intercellular molecule exchange, flowering time control, and signal transduction [11, 14, 15]. *FTIP3* and *FTIP4* prevented intracellular trafficking of a key regulator, SHOOTMERISTEMLESS (STM), to the PM in cells of the peripheral shoot meristem region and played an essential role in mediating proliferation and differentiation of shoot stem cells in *Arabidopsis* [16]. In rice, *OsFTIP1* and *OsFTIP7* acted as factors regulating flowering time, and *OsFTIP7* negatively regulated auxin biosynthesis [17, 18]. In maize, a carbohydrate partitioning defective33 (*cpd33*) mutant has been reported, the gene encoded a protein containing multiple C2 domains and transmembrane regions that caused blade accumulation of excessive amounts of carbohydrates, and the protein was localized to the ER, PM, and PD. It potentially functions were to promote sucrose symplastic movement in the phloem [19]. These studies showed that *MCTP* members in cell and molecular transport played an important regulatory role in cellular and molecular transport [14].

As one of the important food crops, the demand for maize will increase greatly in the future [20]. Sugar and protein transportation and metabolism play a key role in maize growth and production. Sucrose and its derivatives could be directly used as signaling molecules or combined with other pathways to regulate the expression of related genes [21]. However, the evolution and functions of MCTPs in maize remain elusive. In this study, MCTPs were identified in the whole maize genome. We studied their genetic structure, chromosome location, conserved motifs, promoter *cis*-elements genetic classifications and gene expression patterns. This study also used the homologous MCTPs from another 37 species to reconstruct phylogeny and trace the evolutionary history of MCTPs. The results not only help to decipher the evolution fates of MCTPs in maize and other species but also provide basic theoretical support for the application of ZmMCTPs in maize improvement.

## Results

### Genome-wide identification of *MCTP* genes in maize and other 37 species

**Fig. 1:**
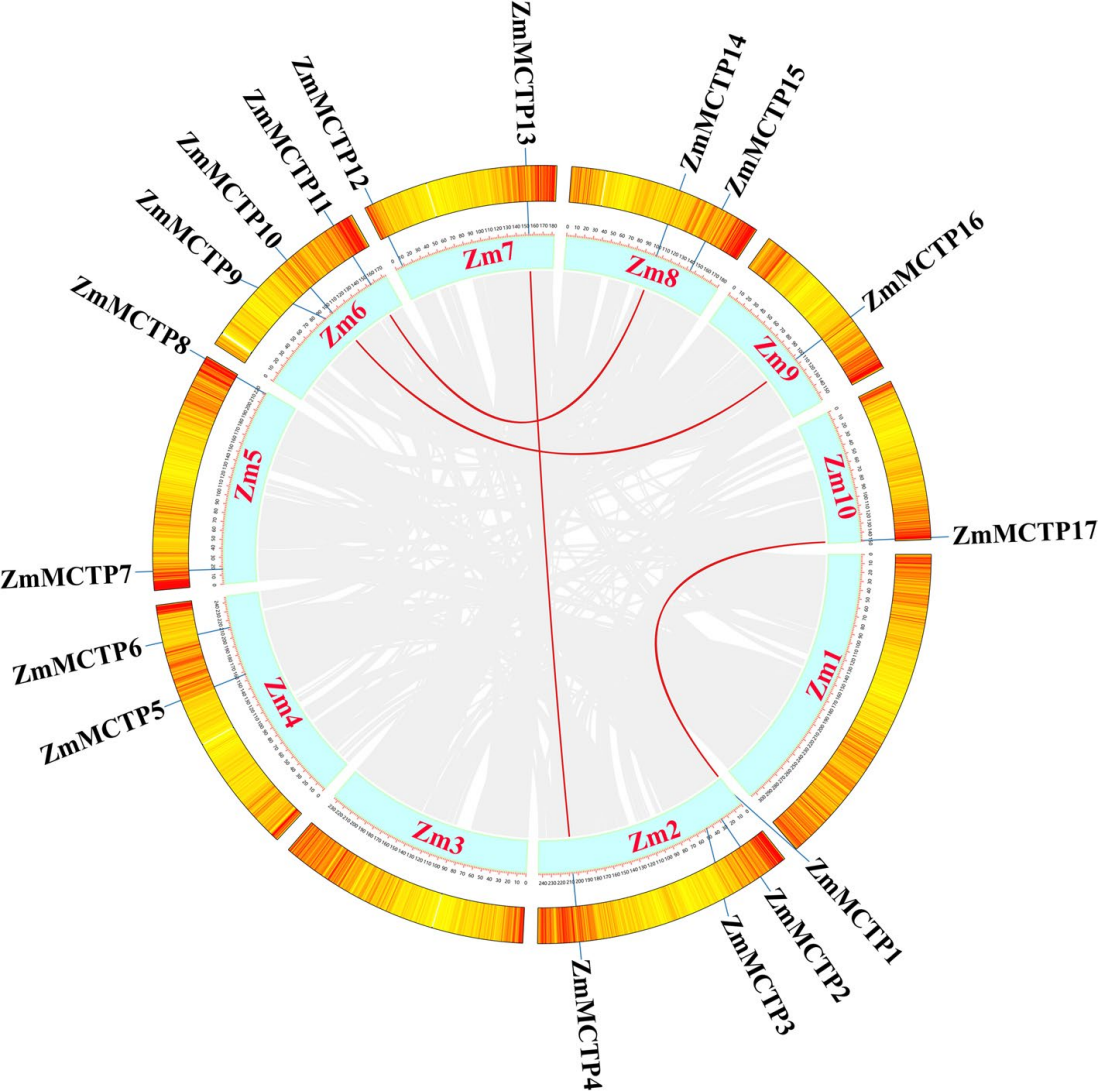
Chromosomal distribution of maize *MCTP* genes and gene densities, genome-wide collinear genes in maize were linked with gray lines and collinear *MCTP* genes were marked with red lines. From yellow to red. Number of genes from yellow to red (distance = 10^^5^ bp)

In this study, seventeen candidate *MCTP* genes were identified in maize by a genome-wide pfam homology search. All these ZmMCTPs contained three to four C2 domains in their N-terminus and one to four transmembrane regions in their C-terminus, and these 17 *ZmMCTP* genes were distributed unevenly on eight of the ten maize chromosomes (Table 1 and Fig. 1). Four *ZmMCTP* genes were located on chromosome 2, three and two on chromosomes 6, 4, 5, 7 and 8, respectively, and one each on chromosomes 9 and 10. The gene density of maize chromosomes was also calculated with 10^^5^ spacing (Fig. 1). The *ZmMCTP* genes were named *ZmMCTP1* through *ZmMCTP17* according to their positions on chromosomes. Overall, the lengths of ZmMCTPs were relatively conserved, ranging from 676 to 1084 aa, with an average length of 930 aa. The isoelectric point (pI), molecular weight (Mw) and domain numbers of the seventeen ZmMCTPs and subcellular localization prediction were listed in Table 1. ZmMCTPs were generally localized to the PM and ER, which might be related to the transmembrane structure of the MCTPs.

**Table 1:** Information of maize MCTPs

Gene name	v4 Gene ID	Chromosomal location	Amino acid Length	pI ^a^	Mw/Da ^b^	MCTP domain		Duplication type ^c^	Predicted subcellular localization ^d^
						C2	TM		
*ZmMCTP1*	*Zm00001d001785*	Chr2:833,688–836,726	1012	9.17	114,917.42	4	4	WGD	PM, ER
*ZmMCTP2*	*Zm00001d002939*	Chr2:27,520,572–27,523,808	1056	9.27	117,601.39	4	2	/	PM, ER
*ZmMCTP3*	*Zm00001d003321*	Chr2:40,129,192–40,131,783	863	9.3	94,965.27	3	3	/	ER, pero
*ZmMCTP4*	*Zm00001d006371*	Chr2:205,967,632–205,970,670	1012	9.03	115,619.01	4	2	WGD	PM, ER
*ZmMCTP5*	*Zm00001d051389*	Chr4:156,895,149–156,897,641	776	9.31	88,874.21	3	3	/	PM
*ZmMCTP6*	*Zm00001d053062*	Chr4:211,658,302–211,661,304	1000	9.48	110,141.2	4	2	/	PM, ER
*ZmMCTP7*	*Zm00001d013654*	Chr5:16,325,465–16,328,605	1046	8.98	114,325.82	4	2	/	PM, ER
*ZmMCTP8*	*Zm00001d018473*	Chr5:221,415,304–221,418,321	1005	9.42	111,464.35	4	2	/	PM, ER
*ZmMCTP9*	*Zm00001d036801*	Chr6:101,297,189–101,299,513	774	9.19	89,459.82	3	2	TD/WGD	PM, ER
*ZmMCTP10*	*Zm00001d036804*	Chr6:101,456,488–101,458,914	676	9.47	76,491.32	3	2	TD/WGD	PM, ER
*ZmMCTP11*	*Zm00001d038340*	Chr6:154,857,022–154,859,451	809	9.53	91,096.99	3	3	WGD	PM, ER
*ZmMCTP12*	*Zm00001d018871*	Chr7:7,869,775–7,872,987	1070	6.53	116,893.74	4	1	/	ER
*ZmMCTP13*	*Zm00001d021494*	Chr7:154,089,912–154,092,986	1024	8.87	116,097.1	4	2	WGD	PM, ER
*ZmMCTP14*	*Zm00001d010202*	Chr8:103,899,795–103,902,224	809	9.51	91,169.01	3	4	WGD	PM
*ZmMCTP15*	*Zm00001d011239*	Chr8:144,197,398–144,200,652	1084	7.68	120,086.8	4	2	/	PM, ER, PD
*ZmMCTP16*	*Zm00001d046749*	Chr9:104,340,583–104,342,910	775	9.17	89,154.32	3	2	WGD	PM, ER
*ZmMCTP17*	*Zm00001d026650*	Chr10:149,387,703–149,390,780	1025	9.18	116,338.04	4	4	WGD	PM, ER

^a^ Isoelectronic point

^b^ Molecular weight

^c^ *TD* Represents transposed duplication, *WGD* Represents whole-genome/segmental duplication

^d^ *PM* Plasma membrane, *PD* Plasmodesmata, *ER* Endoplasmic Reticulum, *pero* Peroxisome

**Fig. 2:**
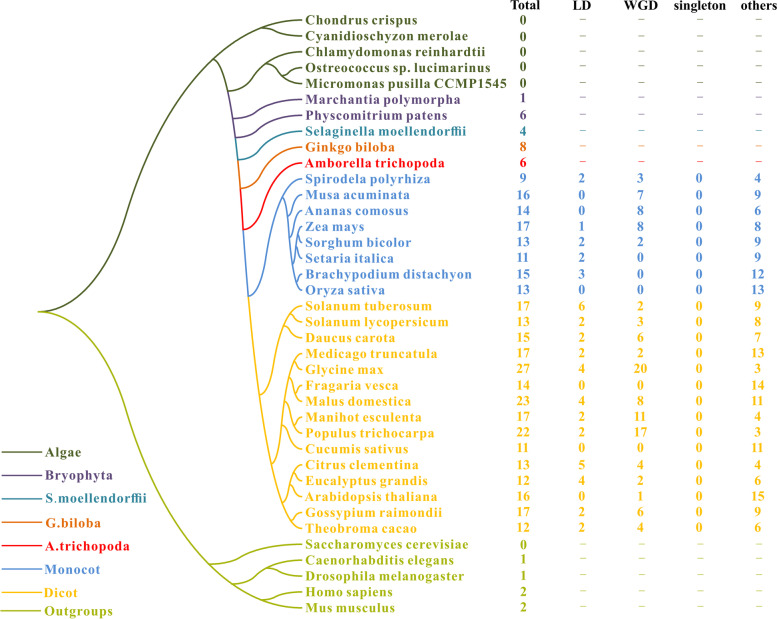
Taxonomic tree of surveyed 38 species. Total number of genome-widely identified *MCTPs* in each species and the numbers of *MCTPs* involved in different duplication-modes were also listed. Species from different taxonomy and/or species were marked with different colors. “Total” represented total *MCTP* gene numbers in each species; “LD” represented local duplication, including tandem and proximal duplication; “WGD” represented whole-genome/segmental duplication; “singleton” represented genes without paralogous pairs in MCScanX; “others” represents genes other than LD, WGD and singleton; “-” means duplication mode were not estimated

To better understand the evolutionary relationship of MCTPs, we also characterized MCTP homologs from 32 other plant species, ranging from single-celled aquatic plants to higher angiosperms. In addition, *MCTP* genes in other kingdoms, including *Saccharomyces cerevisiae*, *Caenorhabditis elegans*, *Mus musculus*, *Homo sapiens* and *Drosophila melanogaster*, were explored and further analyzed as outgroups (Fig. 2 and Table S1). Intriguingly, no *MCTP* gene was identified in prokaryotes or eukaryotic single-celled organisms, including *Chondrus crispus*, *Cyanidioschyzon merolae, Chlamydomonas reinhardtii, Ostreococcus lucimarinus, Micromonas pusilla CCMP1545* and *Saccharomyces cerevisiae*. A total of 385 *MCTP* genes were found in all surveyed species with variable *MCTP* gene numbers from 1 to 27. Among these plant species, *Glycine max* had the most *MCTP* genes, with 27, whereas the common ancestor of dicots and monocots harbored the lowest *MCTP* number. Additionally, the number of *MCTPs* in plants was significantly greater than that in other kingdoms, and with one or two *MCTP* genes in other kingdoms. By evaluating between the numbers of *MCTP* genes and genome size and gene locus number, our results showed that the numbers of *MCTP* genes correlated positively with each gene locus (Figure S1) (R^2^ = 0.69, *p*-value < 0.01, regression analysis).

### Identification of duplication modes of MCTP members

Plants have substantially higher gene duplication rates than most other eukaryotes, which mostly derive from whole-genome duplication (WGD) and/or tandem duplication [22]. MCTPs showed gene expansion in plants, especially angiosperms according to our results, which generally resulted in gene duplication events. As a result, the duplication modes of MCTPs were investigated in the surveyed angiosperms (Fig. 2). As expected, MCTPs in most angiosperms were involved in WGD or/and local duplication (LD, including tandem and proximal duplication) events. In maize, 7 of the 18 MCTPs have been generated by WGD events. In *Glycine max*, which possessed the maximum number of MCTPs, 20 of 27 MCTP homologs were generated from WGD events. These results suggested that WGD and LD events were important for gene number amplification in angiosperms.

**Fig. 3:**
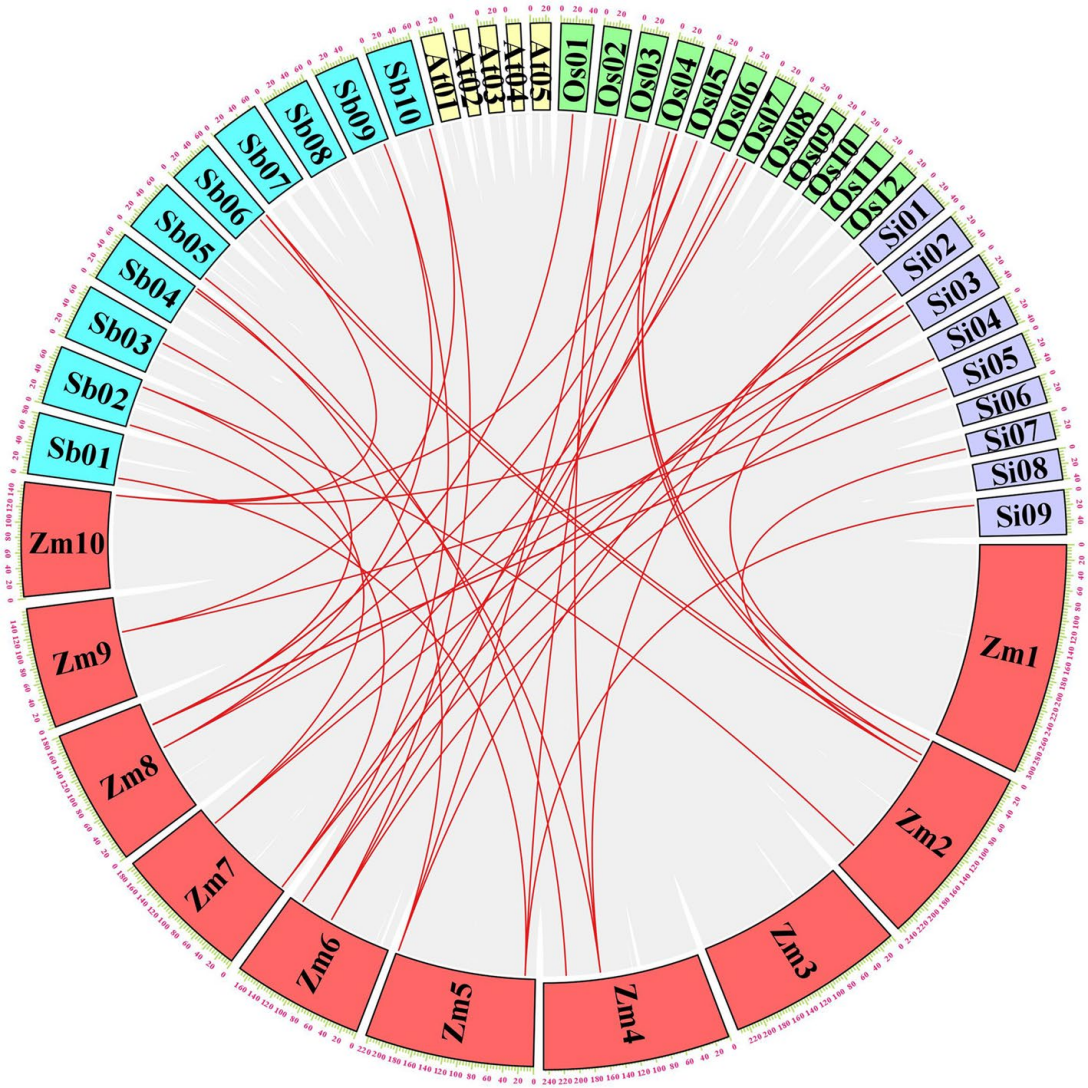
Syntenic analysis of MCTPs between maize and four representative plant species. The results of the syntenic analysis between maize and monocotyledons, model plant, including sorghum, millet, rice, and *Arabidopsis thaliana*. The gray lines at the bottom indicate the collinear blocks within the maize and other plant genomes. The red lines indicate the pairs of MCTPs

We also investigated chromosomal synteny among maize MCTPs and four other plant species (Fig. 3). The homolog pairs of maize MCTPs with rice, sorghum, millet and *Arabidopsis* were 13, 15, 13 and 0, respectively. In addition, MCTP replication pairs were found in sorghum, millet and rice. The results showed ZmMCTPs to be closely related to the rice, sorghum and millet. We also found that no pairs of collinearity MCTPs were shared between rice, millet, sorghum and *Arabidopsis*, which indicated the long-distance phylogenetic relationship among the four species (Fig. 3 and Table S2). These results suggested that these proteins possibly played an important role in the evolution of the MCTPs.

### Estimation of evolutionary rates of *MCTP* paralog genes in angiosperms

**Fig. 4:**
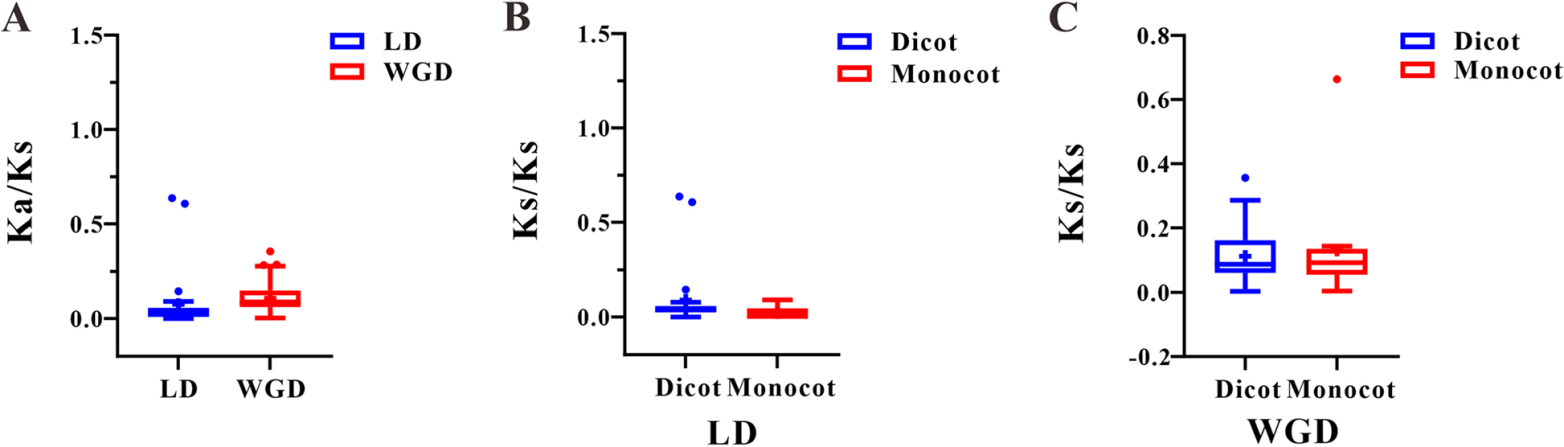
Boxplots of Non-synonymous (Ka) to synonymous (Ks) ratio of WGD and LD duplicated pairs. **A** Ka/Ks values of WGD and LD *MCTP* genes pairs in all angiosperm plants. **B** Ka/Ks values of LD *MCTP* gene pairs in Dicot and Monocot plants. **C** Ka/Ks values of WGD *MCTP* gene pairs in Dicot and Monocot plants

Calculation of molecular evolutionary rates might help to trace the evolution process of the *MCTP* gene family in plants [23]. The ratio of non-synonymous/synonymous mutations (Ka/Ks) is one of the most important parameters for estimating molecular evolutionary rates. In general, Ka/Ks < 1 indicates conservative or purifying selection, Ka/Ks = 1 represents neutral selection, and Ka/Ks > 1 suggests positive selection. Thus, Ka/Ks values were calculated to assess molecular evolutionary rates among *MCTP* paralogous gene pairs. Ka/Ks analysis was firstly performed to explore the evolutionary force of WGD and LD *MCTP* paralog pairs. The results showed that Ka/Ks values of all these WGD and LD duplicated pairs were lower than 1, indicating purifying selection (Table S3). Additionally, the average Ka/Ks value of WGD duplicated pairs was 0.108 in all surveyed angiosperms, which was greater than the average Ka/Ks value of the LD duplicated pairs at 0.076, suggesting more rapid sequence divergence of WGD duplicated gene pairs (Fig. 4). The Ka/Ks values of WGD and LD duplicated gene pairs in monocots were compared with those dicot plants, and both the average Ka/Ks value of WGD and LD duplicated gene pairs in dicots were higher than those in monocots, suggesting that *MCTP* paralogous gene pairs in monocots were slightly more conserved than those in dicots (Fig. 4).

### Phylogenetic analysis of MCTPs in all surveyed species

**Fig. 5:**
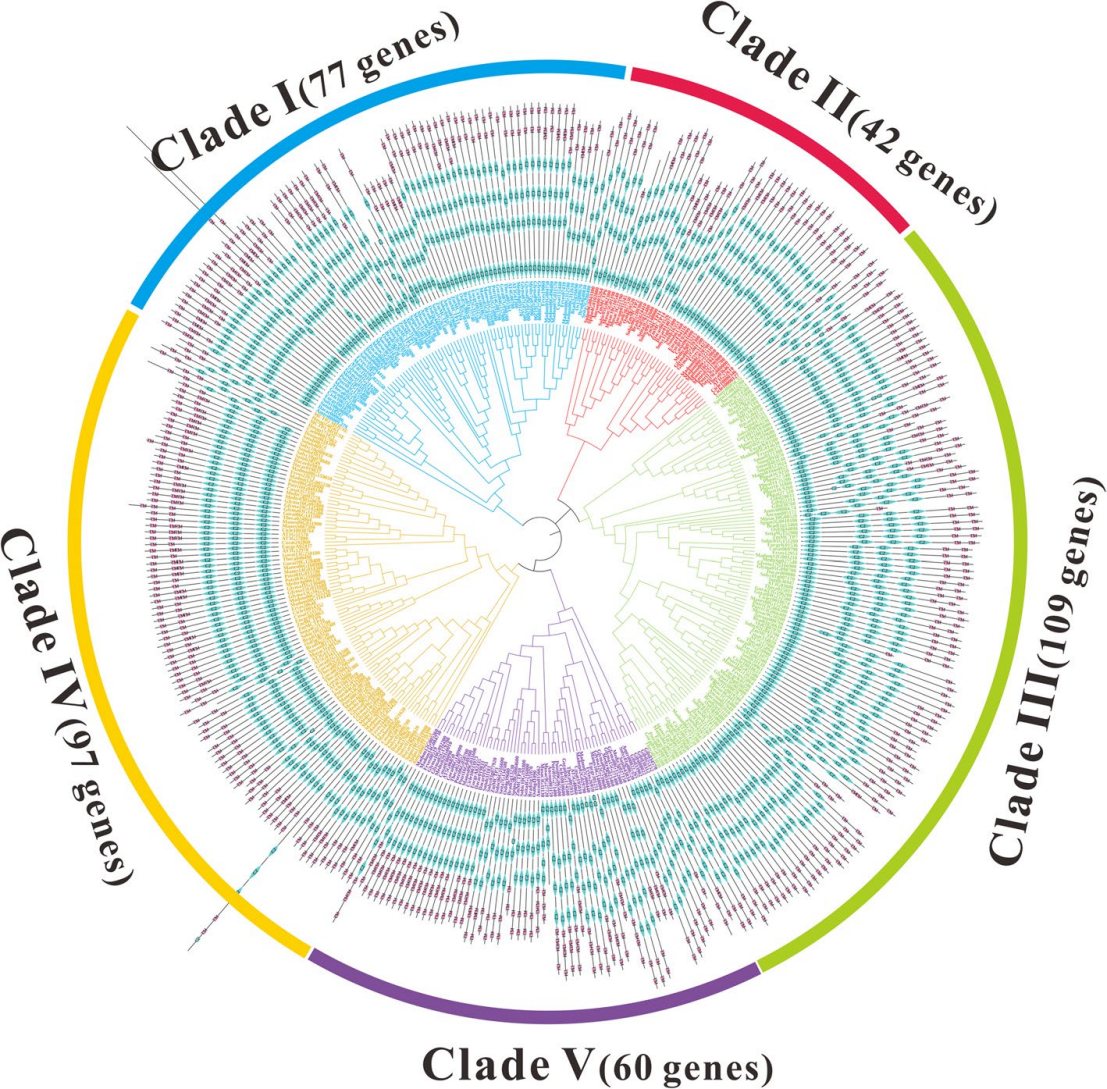
The maximum-likelihood (ML) phylogenetic tree built by MCTPs from 28 plant species and four out-group species and corresponding domain structure. Abbreviation of species names in Table S1 were used as prefixes in the protein names to denote the species. Additionally, MCTP members belonging to different lineages or/and species were marked in the colors same as those in Figure S2. The phylogenetic tree could be categorized into five clades and branches of different clades were marked with different colors. Blue, red, green, purple and yellow branch represented Clade I to V, respectively. TM region topology across MCTPs were also showed, red squares represented TM regions, blue diamonds represented C2 domains

To better trace the evolutionary history of MCTPs in maize and other species, we constructed a maximum-likelihood (ML) phylogenetic tree with 385 MCTP protein sequences from 32 species (See materials and methods). According to the topology of the phylogenetic tree and taxonomy of the species (Fig. 5), five clades were observed and named Clades I-V (Fig. 5 and Figure S2). There were 77, 42, 109, 60 and 97 MCTPs in Clades I-V, respectively. Clade III harbored the most MCTP members and Clade II the least. Clade V contained MCTPs from 24 plant genomes and the four outgroup species, suggesting the evolutionary conservation and ancient origination of MCTPs in these clades. Clade III included MCTPs from 28 surveyed plant genomes, Clade I and II only included MCTPs from angiosperms, revealing a potentially distinct evolutionary history of MCTPs in different clades (Fig. 5).

**Fig. 6:**
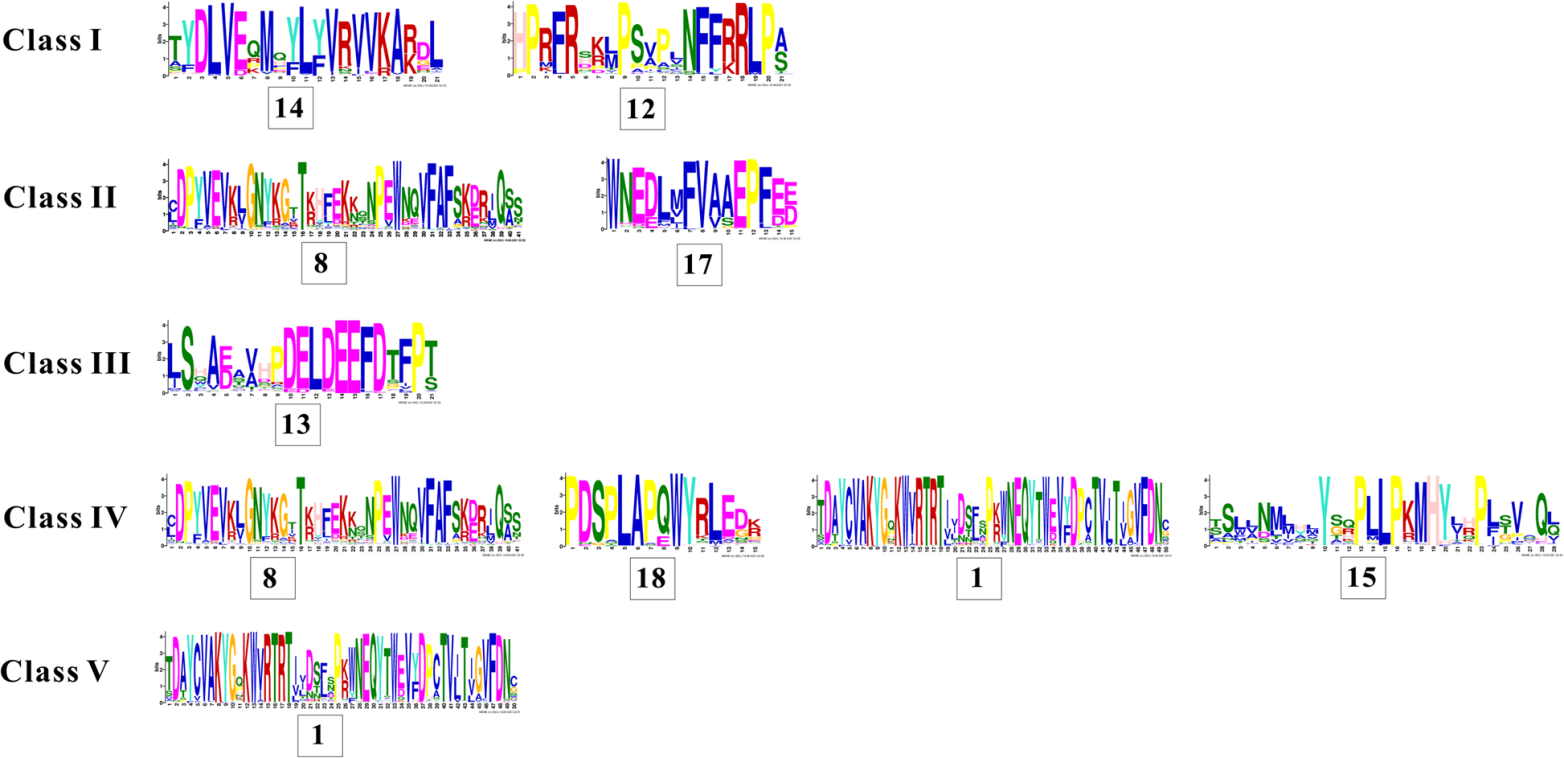
Sequence logos for conserved motifs in MCTPs of five clades, respectively. The results produced by multiple expectation for motif elicitation (MEME) program, as described in the method section. The height of different amino acids represents repeatability. The scale bar at the bottom indicates the length of the motif protein sequence

It has been reported that MCTPs contain three to four C2 domains and one to four transmembrane domains. We used the SMART procedure to predict conserved motifs in MCTPs, and the results showed C2 domains to be evenly distributed at the N-terminus, and that TM regions were located near the C-terminus (Fig. 5). Additionally, conserved motifs were identified, and these MCTP sequences show significant motifs in different species. MCTPs clustering within the same clade shared similar conserved motifs (Fig. 6). For example, motifs 14 and 12 were specific for Clade I, and motifs 8 and 17 were completely conserved in Clade II. In Clade III, motifs 8 and 9 were almost absent, but motif 13 was completely conserved. Similarly, motifs 16 and 20 were hardly absent in Clades IV and V, but motifs 8, 18, 1, and 15 were completely conserved in Clade IV. Motif 1 was completely conserved in Clade V, but not present in three outgroups (Fig. 6 and Figure S3).

### Phylogenetic and structural analyses of maize *MCTP* genes and encoded proteins

**Fig. 7:**
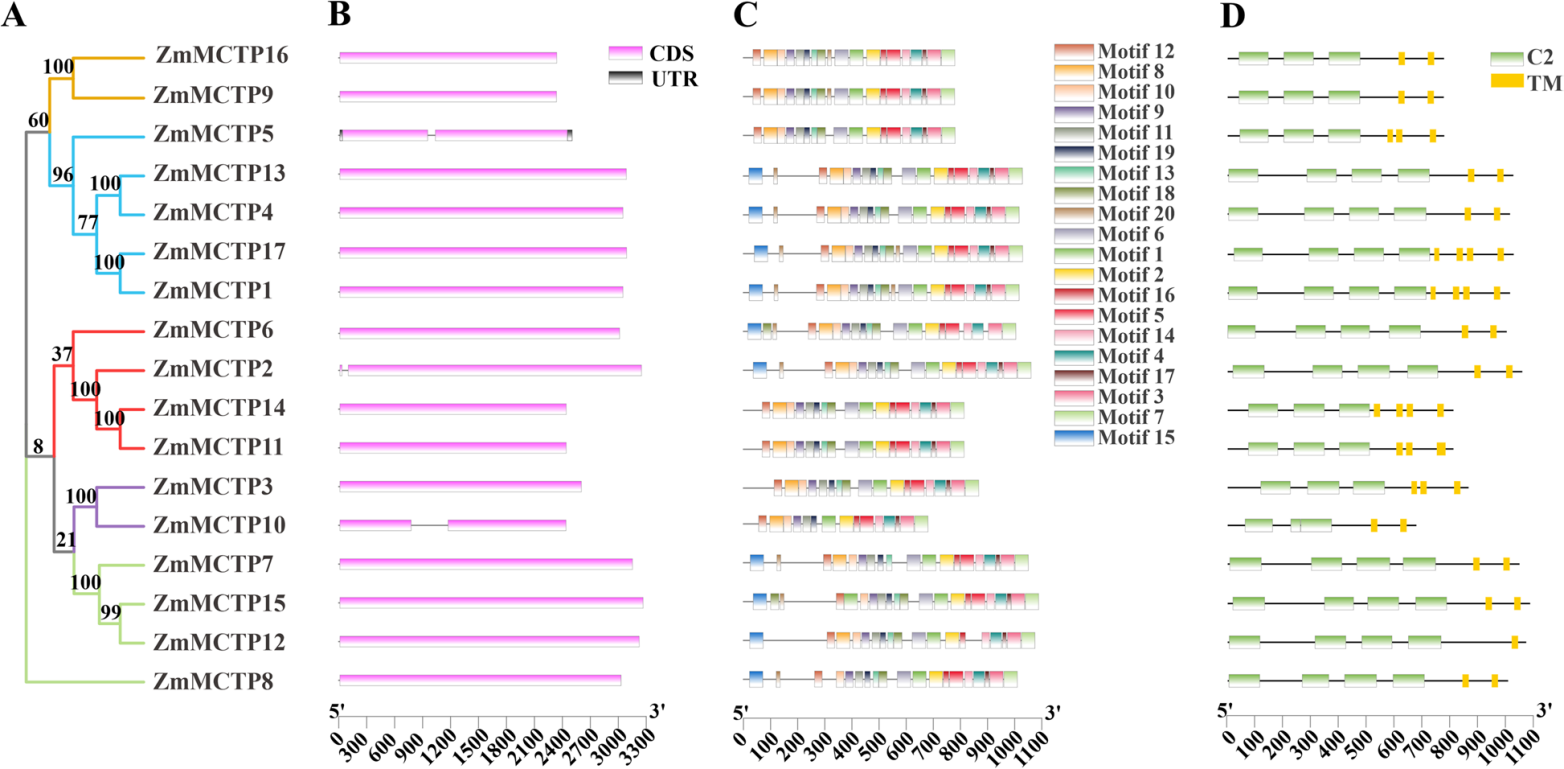
Phylogenetic and structural analysis of ZmMCTP members. **A** The phylogenetic tree is constructed with the amino acid sequences of ZmMCTPs. Blue, red, green, purple and yellow branch represented Clade I to V, respectively. **B** The structure of *ZmMCTP* genes. **C** The motif structure of ZmMCTPs. **D** The domain structure of ZmMCTPs. Green and yellow rectangles represent C2 and TM domains

Focusing on MCTPs in maize, a neighbor-joining (NJ) tree with only maize MCTPs was constructed. MCTPs in the NJ tree showed almost the same topological relationship with those in the above ML tree, suggesting the accuracy of this phylogeny reconstruction of MCTPs (Fig. 7A). Further analyzing the gene structure of maize *MCTPs* revealed that 15 of the 18 *ZmMCTPs* were intronless, *ZmMCTP2, ZmMCTP5* and *ZmMCTP10* were not (Fig. 7B). When further determining conserved motifs and domains in MCTPs, we found that ZmMCTPs with close phylogenetic relationships shared similar motif and/or domain composition (Fig. 7C, D and Figure S3). In addition, C2 domains and TM domains jointly showed that these genes may be related to biological function.

### *Cis*-element analysis of *ZmMCTPs*

**Fig. 8:**
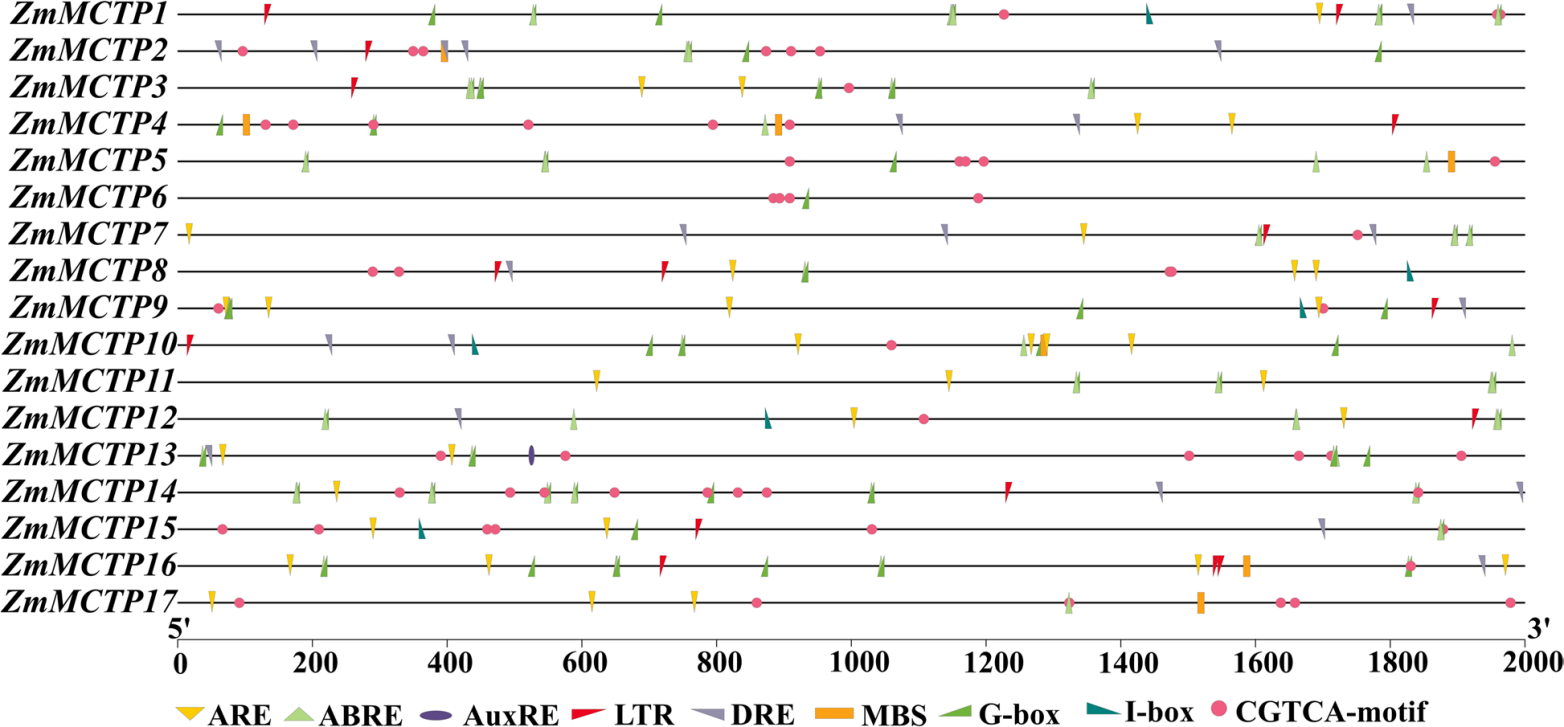
Prediction of *cis*-elements in the promoter regions of *ZmMCTP* genes

Promoter regions play an important regulatory role in gene transcription and expression. Thus, to understand the response mechanism of *ZmMCTPs*, the sequence 2000 bp upstream of the initiation codon of *ZmMCTP* genes was used as a presumptive promoter. The distribution of *cis*-elements in these regions were analyzed by PlantCare, and the results showed that *ZmMCTP* genes had a number of different common *cis*-elements (Fig. 8). According to reports, anaerobic responsive elements (AREs) were important stress response *cis*-acting factors and were found in most *ZmMCTP* gene promoters [24]. Furthermore, most of the *ZmMCTP* gene promoters were found to contain abscisic acid responsive (ABRE) and CGTCA elements, which were two kinds of hormone responsive elements for abscisic acid (ABA) and methyl jasmonate (MeJA) [25–27]. This result indicated that *ZmMCTP* genes were potentially related to ABA and MeJA. In addition, auxin response elements (auxRE) combined with the ARF family activated and inhibited the auxin response genes and recruited the second transcription factor family Aux/IAA repressors to respond to auxin [28], suggesting that *ZmMCTP* genes could be involved in the auxin signaling pathway. Dehydration response element (DRE) and MBS were two *cis*-acting elements with the same function and were involved in gene expression in plants in response to drought stress [29]. Most of the *ZmMCTP* gene promoters contained these two elements, except for *ZmMCTP6* and *ZmMCTP11*. Low temperature response elements (LTRs) were involved in high/low temperature stress [30], and more than half of the *ZmMCTP* genes contained these motifs, *ZmMCTP5, ZmMCTP6, ZmMCTP11* and *ZmMCTP17* did not. Therefore, the results indicated that *ZmMCTP* genes responded to different abiotic stresses and growth signal transductions depending on their *cis*-elements.

### Physicochemical characterization of the ZmMCTP N-terminus and C-terminus

**Fig. 9:**
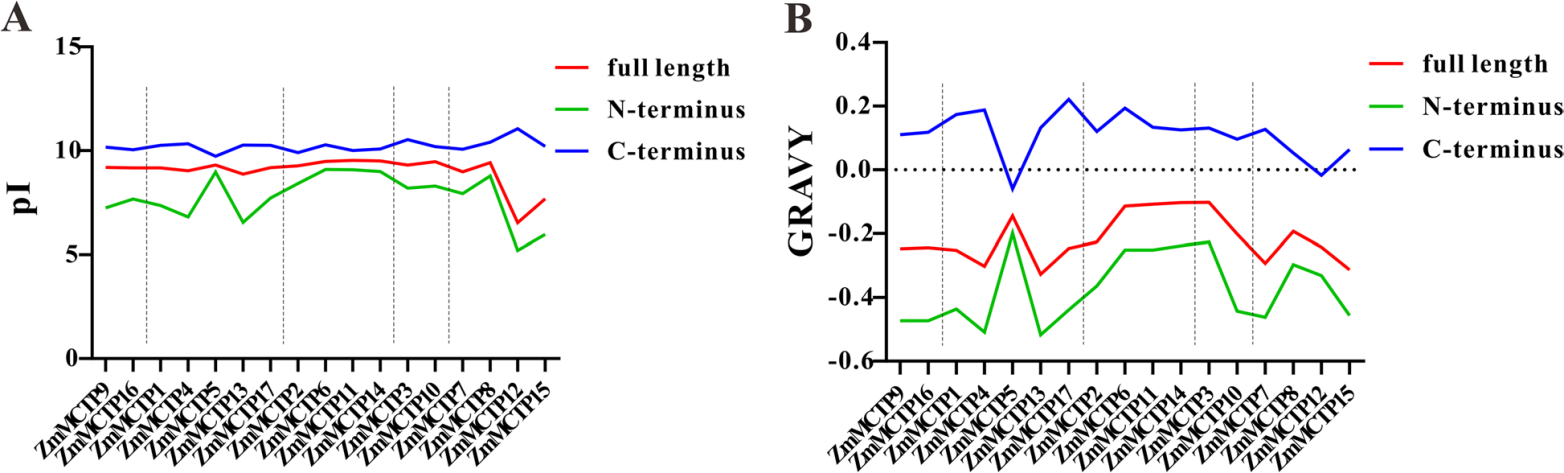
Distinct pIs and GRAVYs between the N-terminus and C-terminus of ZmMCTPs. 17 ZmMCTPs on the x-axis are arranged according to their positions in the phylogenetic tree. Five subfamilies are separated by the dotted lines. **A** The pIs of the N-terminus, C-terminus and full length of 17 ZmMCTPs. **B** The GRAVYs of the N-terminus, C-terminus and full length of 17 ZmMCTPs. pI: isoelectric point, GRAVY: grand average of hydropathicity

In previous studies, the N-terminal and C-terminal regions of ZmMCTPs were reported to contain different structural and functional domains, which possibly defined different physicochemical properties. Thus, we further analyzed the isoelectric points (pIs) and grand averages of hydropathicity (GRAVYs) of the N-terminal and C-terminal regions of ZmMCTPs (Fig. 9). The pIs and GRAVYs of the full-length ZmMCTPs were between those of the N-terminal and C-terminal regions, and the pIs and GRAVYs of the C-terminal region were higher than those of the N-terminal region. Interestingly, the pIs of the C-terminus of all ZmMCTPs were almost unchanged, whereas those of the N-terminus differed among subfamilies, with those in subfamily I and III being significantly different (Fig. 9), indicating that N-terminus pIs differed from C-terminus, which may account for functional differences among ZmMCTPs. However, in the same subfamily of ZmMCTPs, both the N- and C-terminus showed significantly different GRAVYs (Fig. 9), especially, the GRAVYs of the C-terminus tended to be hydrophilic, which may be related to its transmembrane structure.

### Expression profiles of *ZmMCTP* genes in different tissues

**Fig. 10:**
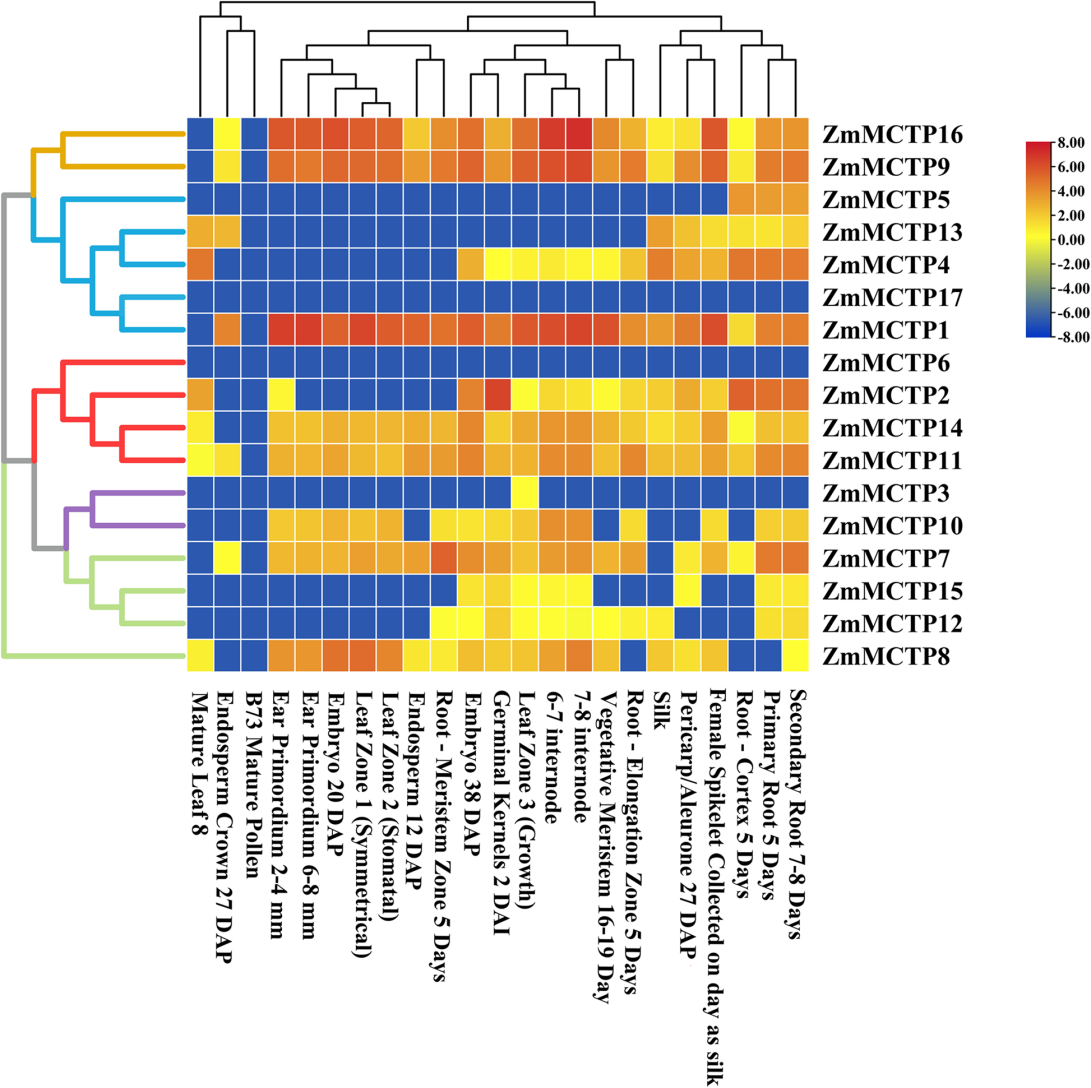
Tissue-specific expression profiles of *ZmMCTP* genes. In left, blue, red, green, purple and yellow branch represented Clade I to V, respectively. Cluster analysis of different tissues is shown at the top of Figure

Using microarray data of 10 organs or tissues at 23 different developmental stages, tissue-specific expression profiles of all *ZmMCTP* genes were explored to better gain insight into their possible functions (Fig. 10). All *ZmMCTPs* showed different tissue expression patterns. In the mature pollen stage, no *ZmMCTPs* were expressed, though *ZmMCTP1, ZmMCTP9, ZmMCTP11, ZmMCTP14* and *ZmMCTP16* were expressed at almost all developmental stages. Moreover, *ZmMCTP1* was expressed at high levels in almost all developmental stages (FPKM ≥ 10). In contrast, expressions of *ZmMCTP3, ZmMCTP5, ZmMCTP6* and *ZmMCTP17* were almost undetectable in different tissues. The expression level of *ZmMCTP16* was highest in the 7–8 internode phase. Interestingly, three of four duplicated pairs due to WGD events, such as *ZmMCTP14* and *ZmMCTP11, ZmMCTP4* and *ZmMCTP13*, and *ZmMCTP16* and *ZmMCTP9*, showed similar tissue-specific expression patterns, though *ZmMCTP1* and *ZmMCTP17* showed different profiles (Fig. 10).

### Relative expression levels of *ZmMCTP* genes in response to stress treatment

**Fig. 11:**
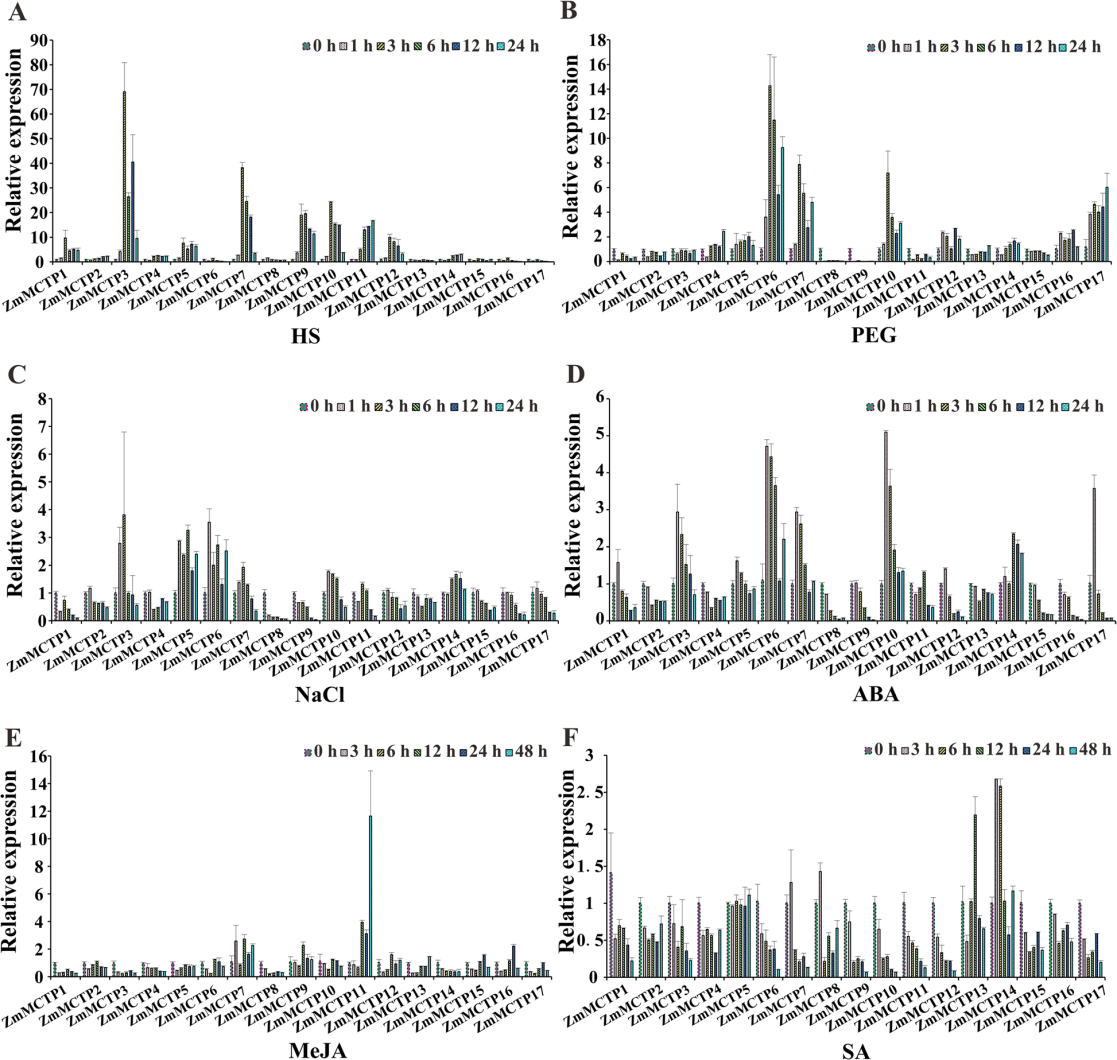
Expression patterns of three abiotic stress and three hormones treatment of 17 *ZmMCTP* genes. Relative expression levels and stress treatments are indicated on the y-axis and x-axis, respectively. **A** HS: heat stress, **B** PEG: polyethylene glycol, **C** NaCl: salt, **D** ABA: abscisic acid, **E** MeJA: methyl jasmonate, and **F** SA: salicylic acid

Our results demonstrated the presence of multiple abiotic stress and hormone responsive *cis*-elements in the promoter region of *ZmMCTP* genes, including the anaerobic responsive element (ARE), the low temperature response element (LTR), the drought response and induction elements (DRE and MBS), the light response related elements G-box and I-box, which are related to abiotic stress, and the hormone pathway-related ABRE and MeJA response element. Therefore, we studied the expression level of *ZmMCTP* genes in response to abiotic stress and hormones to analyze whether the functions of *ZmMCTP* genes were related to abiotic stress and hormones. Leaves of 2-week-old seedlings were treated with heat, simulated drought, salt, ABA, MeJA or salicylic acid (SA) stress (Fig. 11). Ten of the *ZmMCTP* genes were up-regulated more than twofold at one or several time points under heat treatment, including *ZmMCTP1, ZmMCTP2, ZmMCTP3, ZmMCTP4, ZmMCTP5, ZmMCTP7, ZmMCTP9, ZmMCTP10, ZmMCTP11, ZmMCTP12* and *ZmMCTP14*. Among them, the expression levels of *ZmMCTP1, ZmMCTP7, ZmMCTP9, ZmMCTP10 and ZmMCTP12* reached the highest at 3 h after heat shock and then decreased gradually with the increase of treatment time. The expression trends of *ZmMCTP2, ZmMCTP4* and *ZmMCTP11* were the same, with expression levels increasing with heat shock duration. Moreover, the expression trends of *ZmMCTP3* and *ZmMCTP5* were the same and peaked at 3 h followed by 12 h of heat shock. Eight of the *ZmMCTP* genes were up-regulated by drought treatment. In particular, three *MCTPs*, *ZmMCTP6, ZmMCTP7* and *ZmMCTP17,* were up-regulated throughout drought treatment. The expression trends of *ZmMCTP6, ZmMCTP7* and *ZmMCTP10* induced by drought were the same, and the gene expression levels increased from 0 to 3 h and then decreased from 3 to 12 h. The other five genes exhibited different increasing expression trends during drought stress. There were three *ZmMCTP* genes that were up-regulated by salt stress, including *ZmMCTP3, ZmMCTP5* and *ZmMCTP6*. Interestingly, the three genes had different expression trends induced by salt, and the expression level of *ZmMCTP3* peaked at 3 h with salt treatment time and then gradually decreased. The gene expression level of *ZmMCTP5* peaked at 6 h after salt treatment, followed by 1 h, whereas *ZmMCTP6* peaked at 1 h after salt treatment, followed by 6 h. These results indicate that the majority of *ZmMCTP* genes were induced by heat and drought stress rather than salt stress. In addition, 6, 3, 2 *ZmMCTP* genes were up-regulated by ABA, MeJA, SA treatment, respectively. The expression level of *ZmMCTP7* was induced by ABA and MeJA, and *ZmMCTP14* was induced by ABA and SA treatment. Other *ZmMCTPs* were only induced by one hormonal treatment. We also found that the expression trends of *ZmMCTP3* and *ZmMCTP17* were similar under ABA treatment, peaking at 1 h and then gradually decreasing. The expression trends of *ZmMCTP6*, *ZmMCTP7* and *ZmMCTP10* were similar and were highest at 1 h after ABA treatment, they gradually decreased but increased again after 24 h. Under MeJA and SA treatments, the upregulated genes had different expression trends. The expression levels of *ZmMCTP7* and *ZmMCTP9* were highest at 12 h of MeJA treatment, and the expression level of *ZmMCTP11* was highest at 48 h of MeJA treatment. Two genes induced by SA, *ZmMCTP13* and *ZmMCTP14*, showed the highest expression levels at 12 h and 3 h after SA treatment, respectively. These results suggested that *ZmMCTP* genes were probably involved in abiotic stresses or hormone response pathways.

## Discussion

MCTPs, were firstly discovered in *Caenorhabditis elegans,* were mainly composed of three or four C2 domains and one to four transmembrane domains [12]. MCTPs had a conservative Ca^2+^ binding element, GXSD, they could bind to Ca^2+^ and act as a novel ER calcium sensor [31, 32]. In *Drosophila melanogaster*, two *MCTPs* acting as ER localization calcium sensors and calcium-dependent feedback sources, participated in baseline neuronal release and highlight homeostasis plasticity [13]. Four MCTPs in zebrafish were mainly localized to the ER, and knocking down *MCTP2b* expression impaired embryonic development [31]. In plants, *MCTP* genes regulated flowering, growth and development through intercellular transport processes [11]. For example, two *MCTP* genes in *Arabidopsis*, *QKY* and *FTIP1*, regulated intercellular molecular exchange and flowering time [14, 15]. *FTIP3* and *FTIP4* prevented intracellular trafficking of a key regulator, SHOOTMERISTEMLESS (STM), to the PM in cells in the peripheral shoot meristem region and played an essential role in mediating proliferation and differentiation of shoot stem cells in *Arabidopsis* [16]*.* In maize, the *cpd33* mutant of the *MCTP* gene family was localized to the ER, PM, and PD and was involved in excess carbohydrate accumulation and potentially functions to promote sucrose symplastic movement in the phloem [19]. In the present study, *MCTP* genes were identified in 38 species, but no *MCTP* genes were found in prokaryotes and eukaryotic single-celled organisms, indicating that this gene family may have emerged and evolved from multicellular animals and bryophytes. This was consistent with previous reports on evolutionary analysis in animal and cotton species (Fig. 2) [32, 33]. Additionally, MCTPs in plants showed gene number expansion, compared with those in other kingdoms. This is probably because plants are sessile and require more genes to respond to variable environments [34].

MCTPs exhibit apparent evolutionary diversity according to our evolutionary analysis. Phylogeny reconstruction of MCTPs in 38 surveyed species showed that MCTPs can be divided into five clades (Fig. 5). Clade III included MCTPs from 28 surveyed plant genomes, but not four outgroup species, this result indicated that these members of Clade III derived from a common ancestor of plants, and that the family members were relatively conserved. In addition, QUIRKY (At1G74720), a member of the *Arabidopsis* MCTP family previously reported to be homologous to ZmMCTP15, clustered in Clade III [14]. Another FTIP1 (At5G06850) clustered in Clade IV with ZmMCTP3 and ZmMCTP10, suggesting that the two genes were involved in the florigen transport pathway and influenced plant flowering processes (Fig. 5) [15]. Among the five clades, only Clade V had outgroups, suggesting ancient origins and that the members of the clade belonged to a fairly conserved clade. Other clades had either lost genes over the course of evolution or developed new population characteristics that probably were involved in different plant specialized functions.

Previous studies have shown that the expansion of gene families and the retention of duplicates in plants had obvious functional deviations that were strongly affected by the duplication mechanism. In particular, genes involved in stress responses had an elevated probability of retention in a single-lineage fashion following tandem duplication for adaptive evolution to rapidly changing environments [35]. However, replication fragments were generated in the evolution process, resulting in functional redundancy and differences, which played an important role in the response to abiotic stress by participating in intercellular molecule exchange, regulating flowering time and material transport pathways [36, 37]. Gene duplication mode detection showed that WGD events mainly contributed to the gene expansion of *MCTPs* in angiosperms. There were four WGD duplicated pairs among the 17 *ZmMCTP* genes, *ZmMCTP9* and *ZmMCTP16*, *ZmMCTP1* and *ZmMCTP17*, *ZmMCTP4* and *ZmMCTP13*, *ZmMCTP11* and *ZmMCTP14* (Table 1). Specifically, *ZmMCTP9* and *ZmMCTP16* and *AtMCTP3* (*At3g57880*) and *AtMCTP4* (*At1g51570*) belonged to Clade V, and *AtMCTP3* and *AtMCTP4* mutants resulted in defects in plant development, this also showed that *ZmMCTP9* and *ZmMCTP16* had similar functions, with functional redundancy [9]. Moreover, *ZmMCTP9* and *ZmMCTP16* showed similar tissue-specific expression patterns, which could explain their similar functions (Fig. 10).

Conserved motifs and domains were generally regarded as important functional or regulatory elements [38]. MCTPs of the surveyed species showed differences in conserved motifs, similar functions in the same clades, and functional diversity in different clades (Fig. 6, 7 and Figure S3). The number of *MCTP* introns determined the adaptability of plants to different developmental processes and stresses. Therefore, MCTPs had a positive tendency in the evolution process (Fig. 7B). Previous studies have found that MCTPs contained the N-terminal C2 structural domain alone were localized to the PM and nucleus and were mainly related to Ca^2+^ binding. The N-terminal region contained variable amino acid sequences, which may be the reason for the functional differences among MCTPs [11]. MCTPs with only C-terminal transmembrane domains were mainly localized to the ER and were an indispensable part of other MCTPs being targeted to the ER [11]. C-terminal transmembrane domains could also promote the formation of lipid droplets and increase their number [39]. Our study found that the pIs and GRAVYs of the N-terminus of ZmMCTPs were different from those of the C-terminus and that the characteristics of the full-length ZmMCTPs were similar to those of the N-terminus (Fig. 9). These results indicated that ZmMCTPs had different functions and participated in different biological pathways, which may be due to the different N-terminal structures [11, 33]. The C-terminus was also essential for the function of ZmMCTPs. *Cis*-elements played an important regulatory role in gene transcription and expression in response to abiotic stress in plants [40]. ABA activated expression of many genes through ABREs in the promoter region, affecting plant responses to abiotic stresses [41]. AP2/DREB-type transcription factors can bind to the ethylene responsive GCC box and DRE to alter expression of genes related to light, ethylene, and drought to coordinately regulate multiple developmental processes and stress responses [42]. By predicting the *cis*-elements of *ZmMCTP* genes, we found that more than half contained the elements responding to anaerobic, drought and temperature stresses, consistent with the relative expression levels of *ZmMCTP* genes under stress. For example, 8 and 10 genes were responsive to drought and heat (Figs. 8 and 11). Most *ZmMCTP* genes contained ABREs and MeJA response elements, but the number of *ZmMCTP* genes upregulated by ABA, MeJA and SA treatments were 6, 3 and 2, respectively, indicating that *ZmMCTP* genes may be involved in different biological pathways (Figs. 8 and 11). Interestingly, we found that expression of *ZmMCTP3* and *ZmMCTP10,* two members of Clade IV, were simultaneously induced by heat and ABA. This suggested that these two genes potentially were involved in plant stress response involving an ABA-dependent pathway. Subcellular localization and domain structure prediction indicated that different MCTP localizations were related to domain structure but that the same evolutionary branch had similar localizations (Fig. 7B and Table 1). The specific localization and function of ZmMCTPs need to be further explored. Other studies have reported that *ZmMCTP15 (Cpd33)* was related to sucrose transport, and the structure was relatively conserved (Fig. 7), providing clues to decipher the functions of other MCTPs [19].

## Conclusions

A total of 385 *MCTP* genes were identified in all surveyed species, and the maize genome was found to contain 17 *MCTP* genes. Gene duplication event analysis showed that WGD events mainly contributed to the expansion of MCTPs in angiosperms. Purifying selection was the main force acting on MCTPs. The MCTPs were grouped into five clades (Clade I to V) according to phylogenetic analysis, conserved motifs, and structural features. Most *ZmMCTPs* were intronless, and the pIs and GRAVYs analysis indicated that the N-terminus was more dispersive than the C-terminus. Analysis of *cis*-elements showed that *ZmMCTP* genes participated in light signaling and were responsive to stress and hormones. Finally, the expression profiles derived from microarray data and quantitative real-time PCR analysis indicated distinct expression patterns of *ZmMCTP* genes in different organs or in response to abiotic and hormone stresses. Taken together, our results contribute to deciphering the evolutionary history of MCTPs in plants and maize, facilitate further functional analysis of them, and also provide a basis for further clarification of the molecular mechanism of stress responses.

## Materials and methods

### Data sources and identification of the *MCTP* gene family

A total of 38 species gene models and proteomes were downloaded and utilized in the present study, including 33 plant genomes, 4 animal genomes, and 1 microorganism genome. Annotation resources of *Amborella trichopoda, Ananas comosus, Arabidopsis thaliana, Brachypodium distachyon, Chlamydomonas reinhardtii, Citrus Clementina, Cucumis sativus, Daucus carota, Eucalyptus grandis, Fragaria vesca, Glycine max, Gossypium raimondii, Malus domestica, Manihot esculenta, Marchantia polymorpha, Medicago truncatula, Micromonas pusilla CCMP1545, Musa acuminata, Oryza sativa, Ostreococcus lucimarinus, Physcomitrella patens, Populus trichocarpa, Selaginella moellendorffii, Setaria italica, Solanum lycopersicum, Solanum tuberosum, Sorghum bicolor, Spirodela polyrhiza, Theobroma cacao* and *Zea mays* were downloaded from Phytozome (https://phytozome-next.jgi.doe.gov/) [43]. Annotation of the *Ginkgo biloba* genome was downloaded from previous literature [44]. Genome annotations for *Chondrus crispus, Cyanidioschyzon merolae, Mus musculus, Drosophila melanogaster, Caenorhabditis elegans, Saccharomyces cerevisiae* and *Homo sapiens* were downloaded from Ensembl Genome (http://ensemblgenomes.org/) [45]. The local perl script “Pfam_scan pfam” downloaded from HMMER3.1 was used to search the local pfam library (http://hmmer.org/) for the proteomes of these surveyed species [46]. The E-value was set as the default value. All candidate MCTPs were selected with C2 and transmembrane region domains.

The Mw and pI of each ZmMCTPs were estimated using the pI/Mw tool at the ExPASy website (https://web.expasy.org/compute_pi/) [47]. The pI and GRAVY of the full length, N-terminus and C-terminus of ZmMCTPs were calculated using ExPASy. The *ZmMCTP* gene structures were displayed by comparing the coding and genomic sequences with TBtools [48]. The chromosomal locations of *ZmMCTP* genes were mapped onto the maize linkage map with TBtools. The predicted subcellular localizations of ZmMCTPs were analyzed using WoLF PSORTII (http://www.genscript.com/wolf-psort.html). The promoter sequences of *ZmMCTP* genes were obtained from the Phytozome database, and the *cis*-elements using PlantCARE (http://bioinformatics.psb.ugent.be/webtools/plantcare/html/) [49]. The phylogenetic species tree was constructed using the Taxonomy Browser online program (https://www.ncbi.nlm.nih.gov/Taxonomy/CommonTree/wwwcmt.cgi).

### Collinearity and gene duplication mode prediction

Collinearity within or cross species genomes was detected by utilizing the MCScanX package, and the duplication modes of angiosperm *MCTPs* were explored [50]. MCScanX can efficiently classify duplicate gene types within a gene family, including dispersed, proximal, tandem and WGD/segmental duplication **(**SD) duplicates, depending on their copy number and genomic distribution. The orthologous *MCTPs* in maize, sorghum, millet, rice, and *Arabidopsis thaliana* were identified and their relationships plotted using TBtools.

### Calculation of the Ka to Ks ratio

The non-synonymous (Ka) to synonymous (Ks) ratio of *MCTP* gene duplicated pairs in angiosperms was calculated to estimate natural selection pressure. CDS sequences were selected from genomes and translated to amino acid sequences and then aligned by local clustalw2. The aligned sequences and CDS sequences of each gene duplicated pair were submitted to PAL2NAL to estimate the Ka and Ks substitution rates with the PAML package [51].

### Phylogenetic analysis

MCTP sequences from all surveyed species were selected and aligned using MAFFT with the auto-strategy [52]. Gaps in aligned sequences were deleted by TrimAL v1.2 using -automated1 or -strictplus for ML and NJ trees, respectively [53]. Then, we used ProtTest3.4 to further assess the alignment sequence to select the most suitable amino acid substitution model for ML phylogenetic tree construction [54]. The best model according to AIC was JTT + G (-lnL = 111,863.18). Finally, the trimmed aligned protein sequences were submitted to phyML 3.0 to construct the ML phylogenetic tree [55]. The branch-supported measure based on fast approximate likelihood (Shimodaira-Hasegawa Approximate Likelihood Ratio Test, SH-aLRT) was used for branching. Other parameters were set according to the results of the ProtTest test (gamma shape = 1.254, amino acid frequency = observed value). The obtained tree was edited using MEGA-X and iTOL [56, 57].

### Conserved motif analysis

To detect conserved motifs in MCTPs, the online MEME program (https://meme-suite.org/) was utilized with the command line as follows: meme all_protein_sequnece. fas -o result -protein -evt 0.05 -maxsize 10,000,000 -nmotifs 20 [58]. The MEME program identified conserved motifs of the ZmMCTPs with the default parameters, except the number of motifs were 20.

### Expression analysis of *ZmMCTP* genes in different tissues

The expression profiles for *ZmMCTP* genes were obtained from the MaizeGDB website (https://www.maizegdb.org/) [59], and a heat map was generated using TBtools.

### Plant material growth and stress treatment

Two-week-old seedlings of the maize B73 inbred line were used to examine *ZmMCTP* genes expression patterns in response to different stress treatments. The plants were grown in a greenhouse with 28 ± 2℃ and a 16 h light/8 h dark cycle at the School of Life Sciences, Anhui Agricultural University. The treatments were 42℃, 200 mm NaCl, 20% PEG6000, 100 μM ABA for 0 h, 1 h, 3 h, 6 h, 12 h and 24 h, respectively and 50 μM MeJA and 1 mM SA for 0 h, 3 h, 6 h, 12 h, 24 h and 48 h. For sampling, the third leaves were selected and wiped with 75% alcohol. The samples were immediately frozen in liquid nitrogen and stored at -80℃, and RNA was extracted. Three seedlings were taken as three repeat samples.

### RNA extraction and RT-qPCR analysis

RNA was extracted using RNAiso Plus (TaKaRa, Code NO. 9108), the concentration and purity were checked with a nucleic acid concentration analyzer and agarose gel electrophoresis. The obtained RNA was reverse transcribed to complementary DNA (cDNA) using a reverse transcription kit (Vazyme, R323). For RT-qPCR, each reaction had a total volume of 16 µL, consisting of 4.4 µL RNA-free water, 8 µL of AceQ qPCR SYBR Green Master Mix (Vazyme, Q111), 0.8 µL forward, 0.8 µL reverse primers, and 2µL diluted cDNA. Three technical replications were performed per sample. The cycling of qPCR validation was 95℃ for 5 min, followed by 40 cycles of 95℃ for 10 s, 60℃ for 30 s, and 60℃ for 60 s. The RT-qPCR assay was conducted at least three times under identical conditions. *ZmActin1* and *GAPDH* were used as internal controls, and primers were designed with Primer Premier Software (v 5.0). The primers used for RT-qPCR are listed in Supplementary Table S4. The relative expression levels of these genes were calculated by the 2^−ΔΔCt^ method and were displayed by Excel [60].

## Supplementary Information

**Additional file 1. **

**Additional file 2. **

**Additional file 3: Figure S1.** Correlation analysis between MCTP numbers with genome size (A) and Gene Loci No. (B), respectively. **Figure S2.** The maximum-likelihood (ML) phylogenetic tree was built by MCTPs from 32 species. **Figure S3.** Conserved motif compositions of MCTPs in five clades. **Table S1.** Genome information of 33 plant species and five outgroup species. **Table S2.** The homolog pairs of MCTP genes between maize and four other plant species. **Table S3.** Ka, Ks and Ka/Ks ratio of WGD/SD and LD duplicated gene pairs in surveyed angiosperms. **Table S4.** Primer sequences used for RT-qPCR analysis.

## Data Availability

All data generated or analyzed during this study are included in this article and its supplementary information files. However, the sequence data in this study can also be accessed at https://download.maizegdb.org/Zm-B73-REFERENCE-GRAMENE-4.0/. In addition, all databases used in this study are open for public and the links are as follows: Phytozome: https://data.jgi.doe.gov/refine-download/phytozome?q=Arabidopsis+thaliana Ensembl Plants: https://plants.ensembl.org/Chondrus_crispus/Info/Index ExPASy: https://web.expasy.org/compute_pi/ MEME: https://meme-suite.org/meme/tools/meme PlantCARE: http://bioinformatics.psb.ugent.be/webtools/plantcare/html/.

## Abbreviations

MCTP: Multiple C2 domains and transmembrane domain protein
WGD: Whole-genome duplication
LD: Local duplication
Mw: Molecular weight
pI: Isoelectric points
GRAVY: Grand average of hydropathicity
PD: Plasmodesmata
ER: Endoplasmic reticulum
PM: Plasma membrane
ML: Maximum-likelihood
NJ: Neighbor-joining
Ks: Synonymous substitutions
Ka: Non-synonymous substitutions
ABA: Abscisic acid
ABRE: Abscisic acid responsive element
MeJA: Methyl jasmonate
SA: Salicylic acid
DRE: Dehydration response element
