# Supplementary material for: Characterization and phylogenetic analysis of multiple C2 domain and transmembrane region proteins in maize
Source: BMC Plant Biol. 2022 Aug 3;22:388. doi: 10.1186/s12870-022-03771-x (PMC9347167; doi:10.1186/s12870-022-03771-x)
Supplement: Supplementary file 1 — Additional file 1. [file 12870_2022_3771_MOESM1_ESM.doc]

All CDS and protein sequences of MCTPs in surveyed species.

>Ancom003159

ATGAAGTTGGTGGTAGAAGTAGTAGAAGCCCATGATCTTATGCCCAAGGACGGGGAGGGCTCGTCGAGCGCGTTCGCGGAAGTCGACTTCGGTAACCAGATTACGAGAACAAGAACTGTTCCTAAAGATCTCAACCCTGCGTGGAACGAGAAGATCTCATTCTTTCTCGACGACGACCCGGAGGGTACTGCTGCCGACCTTTCGAAGCGGCAGATTGAGGTGAGCGTCTACCACGAGAGGAAGGGGTTTCCGGGCCACGGTTTTCTCGGCAGGGTGCGGATCCTCGGGTCGAATATAGTGAAGCAGGGGGAGGAGATTGTTCATCTGTTCCCACTAGAGAGGAAGTGGTTCTTCTCTCCGGTTAAAGGCGAGATCGCGTTGAAGGTTTACGTCTCCCCCGAACCGAAATCTCCTCCGCCTGGTTCAGCTTCTGCTGAGAAGTCGAAGGTCTCTTCGAATTGTGGTGTTGCAGGCTCTGAAGAAGAAAAGGAACCAAGTACTATAGTTGCTCAAGCGCCAACGCCGCCTAAGAAAGAAGAAAAGGAAGAAGGAGAAGAAGAAGCAGAGGAAGTGATAAAAGTAGAGACGACGCACCACATAAACAAGCAACAGGTCTCGTCCCAGCCCGGAAAATCTGTAGAGCAGCAGACTCCGGCGACGGTCCCCGTGGTGCCCGTCATGGCGTACCAACAGGTCCACTTCGGCCAGGCTCAGCCGAGCCGCGCAGAGGAGTACAAGCTCAAGGACACGAAGCCTCAGCTCGGGGAGCGGTGGCCGATCGGAGGCGGCCACGTTCGCGGGTCCGGAGGCGGCTGGATGGGCCTCGGCACCGGCGACAAGTTCACGAGCACCTACGACCTCGTCGAACAGATGTACTACTTGTACGTGCGCGTCGTGAAGGCGAAGGACCTCCCCGCGAACCCCATTACCGGGAGCTGCGACCCTTACGTGGAGGCAAAGCTCGGAAACTACAAAGGAACCACCAGACACTACGAAAAGAGGATGAACCCGGAATGGAACCAGGTGTTTGCGTTCTCGAAAGAGAGGATCCAGTCCACGGTTCTTGAAGTGTTCGTGAAGGATAAGGAGATGGTCACGAGAGATGAGTACGTCGGTAAGGTGGTGTTCGATTTGAACGAGGTCCCGACGCGCGTCCCCCCAGATAGCCCGCTGGCGCCTCAGTGGTACCGGCTCGAGGACCGCCGCGGGGAGGGGTTTAAGGTGAGGGGGGAGATCATGCTGGCGGTGTGGATCGGGACGCAGGCCGACGAGGCGTTCCCGGATGCATGGCACGCCGACGCGGCGTCGGTGCAAGGGGAGGGCGTGTATAGTATCCGGTCCAAGGTGTACGTGTCGCCGAAGCTGTGGTACCTCCGGGTGAACGTGATCGAGGCGCAGGACGTCGAGCCCAACGAGAAGGGCCGGCTGCCGGAGGTGTTCGTGAAGGCTCAAGTCGGCAACCAGGTGCTGAAGACGAAGCCGTGCCCGATGAGGCCGGGGCTGAGTCCCATGTGGAACGAGGACCTGGTGTTCGTCGCCGCGGAACCTTTCGACGAACAGCTGGTGCTGACGGTGGAGGACCGGGTGTCGGCCACCAAGGACGAGGTGCTGGGCCGGGCTGCGCTGCCGCTGACGCTGTTCGAGAAGCGTTTAGACCACCGCCCGGTCCACTCTCGCTGGTTCAACCTGGAGCGCTTCGGCTTTGCGGTGCTCGAGGGAAACCATCGGAAGGAGCTCCGGTTCGCGAGCCGTGTCCACCTCCGGGTGTGCCTGGAAGGAGCCTACCACGTGATGGACGAGTCGACAATGTACATCAGCGACACGCGCCCCACGGCGCGCCAGCTGTGGAAGCCGCCGATCGGCGTGCTGGAAGTCGGCATTCTCAGCGCGCAGGGGCTGATGCCGATGAAGGCCAAAGACAGCCGCGGCACCACCGACGCGTACTGCGTCGCCAAGTACGGCCAGAAATGGGTGCGCACGCGCACCGTCGTCAGCTCGTTCAGCCCGAAGTGGAACGAGCAGTACACCTGGGAGGTCTACGACCCCTGCACCGTGATCACGCTCGGCGTGTTCGACAACTGCCATCTCGGCAACAGCGGCGGCGGCAACAGCAACGGCGCCGCGGCACAGGCGAAGGATTCGAGAATCGGCAAAGTGAGGATCCGGCTCTCGACCTTGGAAATGGATCGGATATACACGCATTCGTACCCGCTGATCGCGCTGCAGCCGTCGGGGGTGAAGAAGATGGGCGAGCTCCAGCTGGCCGTGCGGTTCACGTGCCTGTCGCTCGCGAGCGTGATCTACCTCTACGGCCACCCGCTGCTCCCCAAGATGCACTACATCCACCCCTTCACCGTGAGCCAGGTCGACAGCCTCCAGTACATGCTCGACGTGGACTCGCACATGTGGAGCATGCGGCGGAGCAAGGCCAACTTCTTCCGGATCGTGTCGCTCTTCTCGGGCGCGGTCGGCGCCTGCCGCTGGTTCGACAGCGTGTGCCGGTGGCAGAACCCGATCACGACCGTCCTCGTCCACGTGCTCTTCGTAATTCTCCTCAGCTACCCGGAGCTGATCCTTCCCACGGTGTTCCTCTACATGTTCATGATCGGGCTGCGGAACTACCGATGGCGGCCGCGGTACCCGCCGCACATGGACACGAAGCTGTCGTGGGCGGAGGCCATCCACCCTGACGAGCTGGACGAGGAGTTCGACACGTTTCCGACGTCGAGACCGCACGACCTGGTGCGGATGAGGTACGACCGGCTGCGGAGCGTCGCGGGGAGGATACAGACGGTGGCGGGGGATCTGGCGACGCAGGGGGAGCGCACGCAGTCGCTGCTCAGCTGGAGGGACCCGCGGGCGACGGCGCTGTTTATAATGTTCTGCCTCGTGGCCGCAGTGGTGCTCTACGTGACCCCGTTCCGGGTGGTGGCGCTCGTCGCGGGGCTCTACGTGCTCCGCCACCCCAGGTTCCGGAGCAAGTTGCCGTCCGTGCCCAGTAATTTCTTCAAACGGCTACCATCCAGGATCGATAGCATGCTCTGA

>Ancom003264

ATGAAGGTGGCGGTGGAGGTGGTGGACGCGCGGGACCTCCTATCGAAGGACGGCCACGGCACGGCGAGCCCCTACGTCGAGGTGGAGCTCGACGGGCAGCGGCGCCGCACGCAGACGAAGCGCGACGACCTCAACCCCACGTGGAACGAGACGCTTTTCTTCGACGTCTCCGACCCCCTCGACCTCCCCAGCCGCACCATCCACGTCTCAGTCCTCCACCACCGCCTCTCTGGCTCGTCCGTTGCGCCATCGAAGGCCGATGCAGCTATCCAGCGCTGCCCCCTCGACCGCCGCCCCGGCCTATCCTTCTCCCGCGTCCGCGGCGATATCGCTCTCCGCCTCTACGCCGTCTCGAATGATCGCCACCCTCCCCCTCTCCGTCGCTCCCGCGCTGTCGATTTCTCCACCGCTTACTCGTCGGCTCCGTCGACGGCTCCCCCTCGCTCTCGCGCCACCGACTTCTCCACCGCATACACGTCGGCTCCGTCAACGGCTCCAACAGAGAACGAGGAGAAGGAAGAGAAGAAGAAGAGGAGGAGGAGCACCACGAGAACCTTCCACTCCATCGGGAGCCAATTTGGCGATCCTGCCGGGCCGATGCCGTTCGACAACCGAGACACTACTCCTCCAGCAGTAGGTGCAGAGGGACCAGCAATAGGCATTCGTCCTCCGGCGGAGACACGGCCAACCCCGACCGCCGCCCCAACCGGCACCACGAAGAAAGGTGGCGGCGACGATAAGATCACCGCCACGTACGATCTGGTGGAGCCGACGACCTTCCTGTACGTGAACGTCGTGAAAGCGCGGGACCTCCCCGCCATGGACATAACGGGAGGGGTCGACCCCTACGTCGAGGTGCGCGTGGGCAACTACCGCAACGCGACGAAGCCTCTGCAGGGGAACCAGAGCCCGGAGTGGCACCAGGTGTTCGCATTCGCCAACGACCACATTCAATCCGATGACGTCGAACTCACGGTCAAGGACGAGAACGTCGTGAGGGACGGGTTCGTGGGGAGAGTCTGCGTCTTGCTCGCCGAGGTGCCGCGGCGCACGCCTCCCGGGAGCCCGCTGGCGCCGCAGTGGTACAGGCTCAAGGACAAGCAGGGGTACTACACGCGCGGCGAGATCATGATGGCCGTGTGGAAGGGGACCCAGGCCGACGAGGCCTACCCGGAGGCGTCGCACTCCGACACCCACGGGTTGCCCTTCCAGGCCCTCGTCCACACCCACTGCAAAATGTACTACCTCCCGCGGCTGTGCTACCTCCGGCTGGACATCATCTCGGCGCAGGACCTGGTCACATCGGAGCCGACGAGGTTGCACCCGGACCTGTTCGTCCGGGCCCAGCTGGGCAACCAGCACGACCAGACCCGCATCTCCCCGTCGCGCTCCGTCAGCCCCACGTGGAACCACCGCACCCTCTTCGTCGCGACATTGCCATCAAAGCCGACCACCGCCAATCCCCCCCTCCTCGATGGTTCAGCCTCTCCGACCCCTCCGATGGCGGCGGCGCCTCCACCTCTAGGTTCCATCCAGCTCCGCGTGTTCTACGATGCGGGCTACCACGTGCTCGACGAGATGGCCCACTACTGCAGCGACTTCCAGCCGTCTGCGCGGCCGCTGCGGAAGGACCCCATCGGCGCACTCGAGCTCGGCATCCGCAGCGGAAAGGACCTCGTTCCCGTCCGAAGCCTCAACGGCGCGGCCCCCACCACCGGGTACTACTGCGTCGCCAAGTACGGGCCCAAGTGGGCCCGCACCCGCACCCTGGTCAACACGCTGAACCCCATGTGGCAGGAGCAGTACACGTGGGAGGTGTTCGACCCCTGCACCGTGCTCACCGTCGCCGTCTACCACAACAACCAGCTCGACGCCCACGATAGCGGCGGCGGCCTCAGGGATCAGCCGCTGGGGAAGCTCCGCATCCGCCTGTCCACGCTGGACGCGGGGCGCGCGTACTTCTACCACCACCCGCTGCAACTGGTCCACCCCTCGGGGATCAAACGGACCGGGGAGCTCCGCCTCGCGGTGCGGTTCAGGTGCACGGCGTGGGTCAACATGATGCGGCTCTACGCCAGGCCCATGCTCCCCAAGCAGCACTACGCGGAGCCCATCCCGCCGCCTGAGGCACCACGCGACCGCCATCTGCGCGGGGAGGTGGTGGAGCACGTGCTCGAGGGGCTCCCGCGGAACGCGCCGCACGCGTACAGCTTCCGCAAGAGCCTGGCCAACTGGCGCCGCGCGGAGGCGCTGCTGAGCGGGGCGGCGGCGTGGGCGGCGTGGGCGGGCTACGTCCGGGACTGGCGCAACCCCGTGACGACGCTGCTCGCGCACGCGAACTTGGTGCTGCTGGTGCGGCACCCGGATCTGATCATCCCCGTGGGGTTTATCTACCTGTTCGGCAGAGGGGTGTGGAACTATCGACATCGGGCGAAGCTGCAGGCAGAGCAAGTGCAGTCGGTGGAGGCGGGGGTGGGGGCGGACGAGGTGGACGAGGAGTTCGACGAGATACCGACGAGGAGGGCGGTAGAGGTGGTGAGGATGCGGTACGACCGGCTGAGGCTGCGGGCGGGGAGGGTGCAGACGATGCTGGGGGACGTGGCCGGCCAGGCAGAGAGGGCCCACGCGCTGCTCAGCTGGCGGGACCCGCGAGCAACGGGGGTGTTCCTGCTCTTCTTGGGACTCGCGACCGCGGTGGTGTACAACGTCCCGTTTTGGCTGCTGCTGCTCGGCTTCGGGCTCTACTACATGCGCCACCCCGTGCTCCGGAGCCGGGTGCCCCCCGCGCCCGTCAATTTTTACAAGCGCCTGCCCTCGAAGGTCGATATGCTACTATGA

>Ancom005172

ATGGCGGTGGCGGAGGGGGGGAGTCGGAGGTTGGTGGTGGAGGTGTGCAACGCGAGGAATCTGATGCCCAAGGATGGGCAGGGGACGGCGAGCGCCTACGCCATTGTCGACTTCGACGGGCAGAGGAGGAGGACGAAGACGAAGCTCCGGGACCTCAATCCCCAGTGGGACGAGAAGTTCGAGTTCCTGGTCCACGACCCCGAGTCCATGACCGGGGAAACCCTGGAGCTCAACGTGTACAACGACAAGAGGACGGGGAAGCGGAACACCTTCCTCGGCAAGGTGAAGATCTCGGGGTCGAGCTTCGCCAAGGCCGGGTCCGAAGTCCTAATTTACTACCCCTTGGAGAAAAGGAGCGTCTTTTCTCAGATCAAAGGCGAGGTCGCGCTTAAGGTGTGGTATGTGGATGATCCCCCTGCAACAACGGCTAGTTCGGAAGGGGGTTCGGGTTCGGAGGCGAAGCCGGAGAAAGCAGCGGCGGAGGGGAGCGAGAAGGCGACGACGGCGGCGGAGGGGGAGAAGAAGGAGAAGGGGGAAAAGAAAACAGAAGAGGCGAAAACAGAGGAGAAGAAGAGCTCTCCTGCTAAAGAGGAGAAGAAGAATAAGTCCCCGGAGAAGCCGAAAGCGGAGGAGTCTCCGGCCTCCGATGCTGCCGCCGCGGAGAAATCGAAGCCGAAGGAGGAGGAGGAGAAGCAAAAGGCGACTAACGCGGCGGCTTCCCCTGCTAAGGAAACACAGCCAGCCGGATTCTCCGGCGACCTCGAGATCCGGCCGAGCGCCGCCGTCGCCGACCGGACCACCACCGGCGGCGGCGGCGGCTCCTACGACCTCGTGGATCGCGTCCCCTACCTCTTCGTCCGCCTCCTCAAGGCGAAGCGCTCCGGCGCGGACGCCGAGAAGCGGCCGGCCCACGCGAAGATCGCGATCGGCAGCCACAGCGTCCGCACGCGGCCGGCCAAAGGCGGGGACTGGCACCAGGTGTTCGCCTTCCACAAGGCGAGCCTCAATTCGACGGCACTGGAGGTGTCCGTCTACGAGGAGACGCCGCAGCCGCCGCCGTCGTCGGAGGGCGACAAGCCGGCCGCCGCCGCCGCCGCCGCCGCCGACACGAGCCTCGGATCCGTCTCCTTCGACCTCCAGGAGATCCCCAAGCGCGTCGCGCCCGACAGCCCCCTCGCTCCGCAGTGGTACACTCTCGACGGCCCCGACGAGGCCGCCGCCTGCGACGTGATGCTCGCCGTGTGGATAGGGACGCAGGCCGACGAGGCGTTCCAGGAGGCGTGGCAATCGGACTCCGGCGGCCTCGTCGTCCACACGCGCTCGAAGGCTTATCTCTCGCCGAAGCTGTGGTATCTGCGGCTCACCGTGATCCAGACGCAGGACTTGAGGCTGCCGCCGCTTCCGGATTCGAAGGCCCGCCCGGCCGGCTCGGTCGGCCCGGAGCTGCACATCAAGGCCCAGCTCGGCGGGCAGGTGTTCAAGACCAACCGGGTGGCGCCCGGCGCCAGCACCAACCCGAGCTGGAACGAGGACCTGCTCTTCGTGGCCGCGGAGCCGTTCGATCCGTATCTGACGATCTCCGTCGAGGACGCGTCATCGTCGTCGTCGGCAGCGGGAGCTCCGGCGGTGGTGGGGCAGGCGAGGGTGCCGCTCTCGACGGTGCACCGCCGGCTAGACGACCGGGCCGAGCCGCCCTCGCGCTGGCTCAACCTGGCCGGCGGGGACGAGGCGCGGCCCTACGCGGGGCGGGTGCACGTGCGGGTGTGCCTGGAGGGCGGGTACCACGTGCTGGACGAGGCGGCGCACGTGGCCAGCGACGTGCGCGCGGCGTCGAAGCAGCTGTCGAAGCCGCCCGTGGGGCTGCTGGAGGTCGGCGTCCGGGGCGCCGCCAACCTCGTGCCCATGAAGCTCGCCAAGGACGGCTCCGGCGGGTCCACGGACGCGTACGTCGTGCTCAAGTACGGGCCCAAGTGGGCGCGAACCCGAACAATCCTGGACCAGTTCAATCCCCGGTGGAACGAGCAGTACGCCTGGGACGTGTTCGACCCCTGCACCGTGCTCACCATCGGCGTCTTCGACAACGTCCGGTTCAAGCAGGCGGAGCCGGGAACCAAGGACGCGAGGATCGGAAAGGTCCGGATTCGGCTGTCGACGCTGGACACCAACCGGGTGTACGCGAACACGTACCCGCTCACGGCGGTGCACCCGTCGGGGGTGAAGAAGATGGGGGAGATCGAGCTGGCGATCCGGTTCAGCTGCCCGTCGTGGCTGAACCTGCTGCAGGCCTACACGACCCCTCTGCTGCCGAGAATGCACTACGTGCGCCCGCTGGGGCCGGCGCAGCAGGACGTGCTGCGGCACACCGCCATGCGCGCCGTCTCGGCCCGGCTCGCCCGCTCCGAGCCGCCGCTGGGCCCGGAGGTGGTCCAGTACCTGCTCGACACCGACGCGCACGTCTGGAGCATGCGGCGGAGCAAGGCCAACTGGTTCCGCGTCGTCGGGAGCCTCTCCGGATTGGCGAATGTGGCCCGGTGGGCGCACGGCGTCCGCACGTGGGCGCACCCGCCCACGACGGTGCTCGTGCACGCGCTGCTCGTCGCGGTGATCCTCTGTCCCCACATGATCCTCCCCACGCTGTCCCTTTACCTGTTCTTGGTCGTCGTCTGGAGGTACCGGGCGCGGCCCCGGGAGCCCGCGGGGATGGACCCGAAGCTGTCCCACACGGACGGCGTCGCGCCCGACGAGCTCGACGAGGAGTTCGACGGGTTCCCGTCGAGCCGGGCGGCCGACGTGGTGCGGATGCGGTACGACCGGCTGCGGGCGCTGGCGGGGCGCGCGCAGACGCTGCTGGGGGACGTGGCGGCTCAGGGGGAGAGGGTGGCGGCGCTTATGAGCTGGCGGGACCCGCGCGCCACCGGGATCTTCGCCGTGCTCTGCTTGCTGGCCTCGCTCGTGTTCTACGTCGTGCCCTTCAAACTGCTGGTGCTAGTGATGGGATTCTATTATCTGCGGCACCCGCGGTTTCGGGACGACATGCCGTCGGCGTCGTTCAACTTTTTCCGGCGGCTGCCGTCGCTGTCCGACCGGATTTTGTAG

>Ancom005910

ATGACGGGCCCCCCCACCGCCGCGGCCGCCGGGGGCAACGTGCGGAAGCTGGTGGTGGAGGTGGTGGACGCGCGCGACCTCGTCCCCAAGGACGGGCAGGGCTCCTGCAGCGCCTACGCCGTCGTCGACTTCGACGGGCAGCGCAAGCGCACCCCCACAGCCCCCCGCGACCTCAACCCCCAGTGGCACCACCGCCTCGAGTTCGTCGTGTCGGAGCCCGCCGCCATGGACGCCGAGGAGCTCGACGTCGAGCTCTACCACGACCGCCGCTTCTCCGCCCCCTCCTCCGCTCCCCGCAAGAACCAGTTCCTCGGCCGCGTCCGCATCTGCGGCTCCCAGTTCGCCCGCCGCGGCGACGAGGGCATCATCTACTTCCCCCTCGAGAAGCGCAACCTCCTCAGCTGGGTCCGTGGCGAGATCGGCCTCAAGATCTACTACTACGACGAGCCCGCCCCACCTCCCCCCGAGGAGAAGCAGCCCGACTCCGCCGGAGCCGCGGAGCCCCCCGCGGCGCCGGATCACTCCGCCGCCGCCGAGGAGCCGAAGGAGCTCCCGGAGGTCCCCGCCCCGACGGAGGCCGCCGTGGAGATACAGCAGCTCCCCCCTCCCTCGCCCCCTCCCGCCGGGGCGGAGGAGGCCTCTCCGCCGCCGGAGGCTCACCCGGCGCCGGAGGCGGAGGTGGCGGATCCGTACCCGCCGGAGGTGAGGAAGGCGCATACGACGTCGAGCACGGAGAGGGTCCGTAGGTCGAGGAGATCGAACGGCGGGGACTACCACCCGGCCCCCGCCCCCGCCCCCGCCCCCGGCCCCGCTCACGCGCCGCGGGTGATCTCGGGCCGGTTCGTGTCGTCGGCCGAGCCGGTCGAGCGGGTCCAGACCACCTACGACCTCGTGGAGCCGATGCAGTACCTCTTCGTCCGCGTCGTCAGGGCGCGGGGCCTCCGCCCGTGCGAGTCGCCCTACGTGAAGGTCCGGACCGGGTCCCAGTCGTTCCGGTCGAAGCCGGCGCGCGACTCCGGCACGGGCGAGCCGGAGTGGAACCAGGTGTTCGCGCTGAGCCACGCGAAGCCGGAGCCGACGCTGGAGATCTCCGTGTGGGACGGCGCCCCCGCCGCGGCGGACGCGTTCCTCGGCGGGGTCTGCTTCGACCTCTCCGACGTGCCGGTCCGGGACCAGCCCGACGGCCCGCTAGCCCCGCAGTGGTACCGGCTCGAGGGTTCCGAGCCGGGCCCCCGCTCGGTGTCGGGCGACATCATGGTGTCGGTGTGGATCGGGACCCAGGCCGACGACGCGTTCGCGGAGGCGTGGATCTCGGACGCGCCGTACGTGAGCCACACCCGGTCCAAGGTGTACCAGTCCCCCAAGCTGTGGTACCTCCGCGTGTCGGTGATCGAGGCCCAGGACCTGCGGCTCCCCGCGGCCGCGGCCCCGGCCCCGGCCCCGGCCCCCTGCACGCCGTTGGACGTGCGCGTGAAGGTGCAGCTGGGCTTCCAGTCGGCGCGCACCCGCAGGTCGACGGTGAGCGGGAGCGGGTCGACGTTCTCGTGGGCGGAGGACCTGATGTTCGTGGCCGCGGAGCCGCTCGACGATCAGCTGGTCGTGCTCGTCGAGGACCGGTCGGCGGCCAAGGACCCGGCGCTGCTGGGCCTCGCGGCGGTCCCCGTCCCCTCCGTCGAGCAGCGCCTGGACGAGCGCCACGTGCCGGCCTCCAGGTGGGTCAACCTCGAGGCCGAGGCCGAGGGCGGGTACCGCGGGCGGGTCCACCTGCGGCTCTGCCTGGAGGGCGGGTACCACGTGCTCGACGAGGCGGCGCACGTCTGCAGCGACTACAGGCCCACGGCGAAGCAGCTGTGGAAGCCCCCCGTGGGGGTGCTGGAGCTGGGGATCCTCGGGGCGCGGGGCCTGCTCCCGATGAAGACCAAAGGAGGCGGAGGAGGCAAGGGCTCCACCGACGCCTACTGCGTGGCCAAGTACGGAAAGAAGTGGGTCCGGACGCGGACCGTGACGGACAGCCTCGACCCGCGGTGGAACGAGCAGTACACCTGGCAGGTGTACGACCCCTGCACGGTGCTGACGGTGGCGGTGTTCGACAACTGGCGCATGTTCGCTGATGCGGCCGGCGAGGAGCGGCCCGACTACCGCATCGGCAAGGTGCGCATCCGGGTGTCGACGCTGGAGAGCAACCGCGCGTACACGGCGTCGTTCCCGCTGCTCGTGCTGCTGCGCTCGGGGCTGAAGAAGATGGGCGAGGTGCAGCTGGCGGTGCGGTTCGCCTGCCCGGCGCTGCTGCCCGACACCTGGGCCATGTACGCGCAGCCGATGCTGCCGCGCATGCACTACCTGCGCCCCATCGGGGTGGCCCAGCAGGAGGCGCTGCGCGGGGCGGCAATTCGCACCGTCGCCGGGTGGCTGGCGCGGGCGGAGCCGCCGCTGGGCCCGGAGGTGGTGCGCTACGTGCTGGACGCGGACGCGCACGGGTGGAGCGTGCGGCGGAGCAAGGCGAACTGGTTCCGGATCATGGGGGTGGTGGCGTGGGCCGTGGGGCTGGCTCGGTGGGTGGACGACGTGCGGCGGTGGAGGAGCCCCGTGACGACGGTGCTGGTGCACGTGCTGTACCTGGTGCTGGTGTGGTATCCGGAGCTGGTGGTGCCGACGGGGGCGCTGTACGTGTTCCTGATAGGGGTGTGGTACTACCGGTTCAGGCCAAAGGGCCCGGCCGGGATGGACGCAAGGCTGTCGCAGGCCGACACGGTGGAGCAGGACGAACTGGAGGAGGAGTTCGAGCCGGTGCCCACGTCGGTGGAGGTGCTGCGGGTGCGGTACGAGAGGCTGAGGACGCTGGCAGGGAGGGTGCAGAGGGTGATCGGGGACCTGGCGGCGCAGGGAGAGCGGCTGCAGGCGCTGGTGAGCTGGAGGGACCCCAGGGCCACCAGGATATTCATCGCCGTCTGCCTTGCGGTGGCGCTGGTGCTCTACACGGTGCCGCCGAAGATGGTGGCGGTAGCGCTCGGCTTCTACTTCCTCCGGCACCCCATGTTCAGGGACCCCATGCCGCCCGCCGCCGTCAACTTCTTCCGCCGCCTGCCCAGCCTCTCAGACAGACTTTTGTAA

>Ancom006924

ATGGGTGCTAAAGAGGAGGAGGAGGAGAAGCTGGTGGTGGAGGTGGTGGCGGCGCACAACCTGATGCCCAAGGATGGGCAGGGGTCGTCGTCGCCGTACGTGGAGGTGGAGTTCGAGCACCAGAAGAGGCGCACCCGCTCCGTGCCCAAGGAGCTAAACCCCGTGTGGAACGAGCGACTCCTCTTCCCCGTCTCCGAACCCGACGACCTACCCTACCGCGCCATCGACGTCGCCGTCTACAACGACCGAGCCCACGCACACGGCGGCGGCGCCCGCAACTTCCTCGGCAGGGTCCGCGTCCCCGCCGCCGGAGTCCCCGCCCCGGGCGAGGACGCCGTGCCCCAGCTCTTCCCCCTCGAGAAAAGGAGCCTCTTTTCCCACATCCGCGGCGAGATCAGCCTTAAGATATACCGCACCTACGGCCACGCCCACGCGCCCGCCCAGCAAGTGGGGCTCGGCCCCGGCCCCGGCCCCGGCCCCGGCCCCGGCCCCGGCACCGCCAAAGCCAAGGAGAAAGCCGCGCCGAAGCACCAGAAGCAGCAGCAGCAGCAGCAGCAGCAGCCGCCGCAGCTGCCGCCCGCGGTGGCGGCGCAGCCTCCGCCGCAGCAGCAGCCCTCGGCGGCGCAGAAGCAGCCGCGGCCGCGCCCGGGGCCGGAGCTCTCGGGCCCGGGCCCCGCGGGCGCCATCAAGCCGATGATCCTCGCCACGGGGCCCGCGCTGGGCCCGGAGCCGTACCCCGTCCTCTGCGCCGGCTCCGGGGCGCAGGAGTTCGCGCTGAAGGAGACGCGGCCGCGGCTGGGCGGCGCCGCCGCGGCGGCCGGGGGGCACCGGGACAAGTCGAGCGCGACCTACGACCTGGTGGAGCAGGTGCAGTACCTGTACGTCCGCGTGGTGCGCGCGCGCGACCTCCCGCAGAGCGGCGGCGAGGTCCACGCGGAGGTGAAGCTCGGGAACTACCGCGGCGTCACCCCCCCGGCCCCCGCCTCCGCCGCGCACTGGGGCCAGGTGTTCGCCTTCTCCGGGGACTCCGTCCACTCGTCGGTGGCCGAGGTGCTCGTGAAGGAGCGCGAGCGCGAGGAGTCGATCGGGCGCGTGGTGTTCGACCTGAGCGAGGTCCCGCGGCGGGCCCCCCCGGACAGCACCCTGGCCCCGCAGTGGTACCGGCTGGAGGACCCCAGCGGCGACCGCCGGGGCGAGCTCATGGCGTCGGCGTGGTTCGGCACCCAAGCCGACGAGGCCTTCGCCGAGGCCTGGCACTCCAAGGCCGCCGGGGTCCACGGCGACGGCCTCGGCTCCATCAAATCCAAGGTCTACGTCGCCCCCAAGCTCTGGTACCTCCGCGTCTCCGTCCTCGAGGCCCAGGACCTGCCCCCGGGCGCCGGGGACAAGGCCCGGTTCCCGGAGCTCTTCGCCCGCGCCATGGTCGGGGCCCAGATCCTCCGCACCCGGGCCGCCGCCCCGGCCCACCCGAACCGGGGCCCCTCGAACCCGGTCTGGGCGGAGGACCTCATGTTCGTGGTGGCCGAGCCGTTCGACGACCTGCTGGTGGTCTCCGTCGAGGACCGGGTCGGGCCGGGCCGGGACGACGTGCTGGGCCGGGCCGGCTCGGGGGCCCAGAGTTGCCGGTTCGGCGCGGGCCGGGTCCACCTCCGGCTCAGCCTCGACGGCGGGTACCACGTGCTCGACGAGTCCACCGCGCACAGCAGCGACCTCCGCCCCACCGCGAAGCAGCTGTGGGCCCCGCCCGTGGGCGTGCTGGAGCTGGGCGTGCTGGGCGCCACGGCGCTCGCGCCCATGAAGCCCCCGCGCGAGGGCAAGCCCGGGTCCTCCGCCGACGCCTACTGCGTCGCCAAGTACGCCCACAAGTGGGTCCGGACCCGCACCGTCGTCGACTCGCTCTGCCCCCGGTGGAACGAGCAGTACACGTGGGAGGTGTTCGACCCCTGCACGGTGATCACCGTCGGCGTCTTCGACAACTGCCACGTCGACAGGACCTCCTCCTCCTCCTCATCCTCGCCGCCTTCGCCCCGCGACGCCCGCATCGGCAAGATCCGCATCCGGCTGTCGACGCTCGAGACGGATCGGGTGTACACGCACGCGTACCCGCTCCTGATGCTGCACCCGTCGTCGGGGTTGAAGAAGATGGGGGAGCTCCACCTCGCCGTGCGCTTCTCCTGCGCCAACGCCGCGAACATGCTCCACGCCTACGCGGGCCCCTTGCTCCCGAAGATGCACTACGCGGACCCGCTCCTCGTGCGCCAGGTGGAGAACCTGCGGTACCAGGCCACCAACGTGGTGGCCGCGCGCCTGGGCCGCGCCGAGCCCCCGCTGGGCCGGGAGGTGGTGGAGTACATGCTCGACCGCGGCTCCCACCTCTGGAGCATGCGCCGCAGCAAGGCCAACTTCTTCCGCCTCGTCGCCGTGCTCTCCGCACCCATCGCCGTCGGCCGCTGGATCGAATCCGTCCGGTCGTGGACCCGCCCCGTGCACTCCTTCCTCGTCGCGTCGGCCTACGTGTTGTTCGTGCTCTTTCCGGAGCTGATACTCCCGACCCTCTTCTTGGCCCTGGCGGCGGCGGGGCTGTGGCGGTATCGATCGCGGGAGCGGCACCCCCCGCATATGGACGTCCGGCTGTCGTACGCGGACGCGGTGTACGCAGACGAATTGGACGAGGAGTTCGACACGTTCCCGACGAGGAGACCGCACGACCTGGTGCGGATGAGGTACGACCGGCTGCGGAGCGTCGCGACGCAGGGCGAGCGCGTGCAGGCGCTGCTCAGCTGGAGGGACCCGCGGGCCACGCTGATATTCCTCGCATTCTGCGTCATCGCCGCCGCCGCGCTCTACGCCGTCCCCATGAAGGCCATCCTCGGAATGTGGGGATTGTACGCGCTGCGGCCGCCGCGGTTCCGGAGCCGCATGCCGTCGCCGTTCATGAGCTTCTTCAGGAGGCTGCCCACCAAGGCGGATAGCTTGTTGTGA

>Ancom008766

ATGGCCGCAACGATGAGGAAGCTCGTAGTGGAGGTGGTGGAGGCCCGGAACCTCCTCCCGAAGGACGGCACCGGCACGTCGAGCCCCTACGTGCGCGTCGACTTCGACGGGCAACGCAGAAAGACCCGCACGGCGCAGCGCGACCTCAACCCGACGTGGAACGAGGCGCTGGAGTTCGACGTCGCGGCCGCGGCGGACCTCGACGAGCCGCTGGAGGTGGACGTGTTTCACGACGTCAGGGTGGGGCCGAGCCGGCGGAACAACTTCCTCGGGCGGGTGAGGTTGGATTCGCGGCAGTTTTGCGTGCGGAAGGGCGAGGAGGCGCTGATGCACTTCCCGCTGGAGAAGAAGAGCTTCTTCAGCTGGGTCAGGGGGGAGATCGGCTTGAAGGTGTATTATGTCGACGACCCCGTCCCCGCACCTGCTGCTGCTGCTGCTGCTGCTGCTGCTGCGGAGCCGGATAATTCTGCCGCTAACGATAGTAGTGATGAACCGAATTCTGTCCATGAGAATTGTAATGAACCTGCAGCACCGCCGCTGGCGGAGGCTCCAAATGCGGAGAAGCCTGCGGAAGCGCCACCGGCCGAGGCCGAGGCCGACGGAGCAACCGCAGCTGCAAGTGCTGCTACTTCCGAATTGGAAGAAACGGCGCCGGCGGCGGTGCCTACGGAGAATTCTGAAAAGCCCGCGGAAGAATCGGATCGGCAAAACATGGAGGCAAAAGAAGAAGCGCCGCCCGAGCCAATTGCGGCGGCGGCGGTGGAGACAAGAAGTGAGCCGGAAAAGGAAGCGGCGGGTCCCGAATGGGCGCCGCAGCGGCCGCGTCGGATGAAGGGGATGGGGGCGGAGATCGCCAGGGAGGGGCCCGCCAAGTACGACCTGGTGGACAAGATGCAGTACCTGTTCGTGCGCGTGGTGCGCGCGCGCGGGCTCCCCGCGGGGGCGGCGCCGCGCGTGCGCGTGGCCGCGCACGGGCGCCGCGCCAGCACGCGGGAGGCGCGGCGCGCGGGGCCCCACCACGAGTGGGACCGCACCTTCGCCTTCGCCCGCGAACCCTACTCCGGCGACCCCGCCGCCGCCCCCGCCGCGTCGCTCGAGGTCTCCGTGTGGGACCTGCCCCCCGGGGAGGAGGACGACCACGACGCCGACGCCGACGACGCCGAGGGGGAGGAGAGGCATCTCCTCGGCGCGCTCTGCTTCGACGCGTCCGAGGTCCCCCTGCGGGACCCACCCGACAGCCCCCTGGCCCCGCAGTGGTACCGCCTCGAGGGCCCCCGCGGCGGCGGCGGCGGCGGCCAACTGATGCTCGCCACGTGGATCGGGACCCAGGCGGACGAATCCTTCGCCGACGCGTGGAAGGCCGACGCGCCGCCCTCCGCCGGGTCCTCGCGCTCCAAGGTCTACGTCTCCCCCAAGCTGTGGTACCTCCGCGTCACGGTGATCGAGGCCCAGGACGCGCTCCCCTCGCCGACGCGCGACGCCGCGATCGCCGTCGCCGTGCGCGCGGCACTGGGGTCTCAGGTGCTCCGGACCCGCGCCGCCGCGTGCCGCGGCGGCGCCCCGTCGTGGAACGAGGACCTCATCTTCGTGGCCGCGGAGCCCTTCGGCGAGGACGAGCGCCTCCTCCTCTCCCTCGAGATCCGCGGCGCCGGCAAGGACGCCGCCGCCGCCGCCGTGGGCTCGGCCGCGCTCCCCCTCTCCGCCGTGGAGCGCCGCGTCGACGACCGCACGGTAGCCTCGCGCTGGCTCGACGTCCTCCCCACCGTCAAGAAGAAGGGGGGCCCGGGACAGGGACACGGTCACGTGCCGGGGGGGAGGCTGCACGTGCGGGCGTGCCTGGACGGGGGGTACCACGTGGCGGACGAGGCGCCGCACGCGTGCAGCGACTACCGCCCCGCGGCGCGCCAGCTGTGGCGGGCGCCGCTGGGGGCCGTGGAGCTCGGCGTGGTCGGCTGCAGGGGCCTGCTCCCCATGCGGACCCTCCGGGGGAAGGGCTCCACCGACGCCTACGCGGTCGCCAAGTACGGGCCCAAGTGGGCCCGCACCCGCACCGTCGCCGACTCCCTCGACCCGGCCTGGAACGAGCAGTACACGTGGCCCGTCTACGACCCCTGCACCGTCCTCACCCTCGCCGTCTTCGACGAGTCCCCGCCGTCCGATCCCGACGCCGGGCCCAAGGACCCCGCCCCACCCCCGTGCTCCCGGCCCATGGGGAAGGTCCGGATCCGGGTCTCGACCCTCGAGACCGATCGGGTCTACCGCGGATCCTACCCGTTAATTTTAATGCTCCCCGGTGGCGCCAAGCGCATGGGGGAGATCGAGCTCGCGGTGCGCTTCGCGCGCGCGGGGTCCGTGCTGGATCTGGTGCACGCGTACGGCCTCCCGATGCTCCCCCCAATGCACCACGTCCGCCCGATCCCCCCGGCACTGCAGGAGCCGCTGCGGCTCCTGGATGCAGCCGAGCCCCGCGGGTTCAGCATGCGGAAGGTGCGCGCCAATTGGCACCGGATCGTGGCCGCGCTGGCGTGGACCGCCGACGCGGCGCGGTGGGTCGAGGACACTCGGAATTGGCGGAACCCGACGGCGACAGCGCTCGCGCACGGCGCGCTCGTGCTGCTGGCGTGGCACCCGGACCTGCTGCTGCCCGTGCTGGCGGTGCACTTCGCGGCGGTCGGGGCCTGGAGGTACCGGAGACGCCTGCAGGGGCCCGTGCCTCACCCTTGCGTGAGGGTGTCGATGGCAGAGACGGCAGATAGGGAGGAGCTGGACGAGGAGTTCGACCCGGTGCCGAGCACGAGGGCCGCAGAGGTGGTGTGGGCGAGGTATGACAGACTCCGGGCGGTTGGGGCGAGGGTGCAGGCGATGCTGGGCGACGTGGCGGCGCAGGCGGAGAGGGTGCAGGCGCTGGTGACGTGGAGGGACCCGCGCGCCACCGGAATGTTCGTGGCGATGTGCCTGGCGGTTGCAGTGGTGCTTTATGTGGTCCCATCGAGGATGATGGCGGTGGCGGCGGGGTTTTACTACCTCCGGCACCCGATGTTTCGGGATCGGATGCCGCCAGCGGCGCACAACTTCTTCCGGCGGCTGCCCGCGCTATCCGAGCGCATCATATAA

>Ancom009308

ATGGGTAGTTACAAGCTCGGTGTCGAAGTGATCAATGCGTACAATCTCATGCCCAAGGAGAAGGGCTCTTCGAACCCTCACGTCGAGCTTGAATTCAACGGCCAAAAATTCCGCACCACCGTCAAGGAGAAAGAACTCAATCCGGTGTGGAACGAGCGCTTCTTCTTCAACATCTCGGACCCTTCTTCTATCCCCAACCTTTCTCTTGAAGCTTCTATCTACAGCATCAACACAGAAAGAAACTCTAGATCCTTTCTCGGCAAAGTCCGGCTCGCGGGAACCTCCTTTGTGCCCTCCTCCGACGCAGCCGTGATGCATTATCCGTTGGAAAACAACATTTTTTTTTCGCGTGTGAGAGGAGAACTAGGGCTCAAAGTTTTCCTCACCGTTGATCCGTCAATCAAAGCTTCAAATCCTCTTCCGGCTATCGATCCTGCTACGAAAATTCATCCTCAGAAACAAGTCTTAAACCCTAAGCCTAATCCGGCTCGAGATCGGTCAGGTGGACCGAGACGTACATTTCATAGTATTGGAAAAGAGGCCCACAATCAGCATGAACATTGTCCTCCGGTCACCACAAGTGAAGAGCCAGTAAAGCGTGATGCAGGCCAATTCAAATGCGAGTCGCCAAAATCCAAATGCGAGTCACCGAAATTTGCGAACATGTGCTCACGGGCACCCTCGAAGAAAGAGGCTGAATATGCACTTAAAAAAACAAACCCATCCCTCGGCTTCGGACAGATCGTTGGTGGCCAAGTCATACACGCGGAGAAGCCAAGCACTATGTATGATCTTGTCGAGCCGATGCAGTATCTTTTCGTCCGAGTTGTCAAAGCAAAGGACCTACCGGAAAGCACTGATCCATTCACTGAGTTGAAGTTCGGGATCTCTAAGGCCGCCACAAAGCAGCTTGAGAAGAAACCGAATCCGGAGTGGAATGAGGTGTTCGCCTTCTCCCACGAATGTATGCACTCGACGATCCTCGAAGTTCAGATCAAAGATAAGAAGCAAGAGAAGGATGAGTACATTGGGCGTGTTCGGTTCAATATAAATGATGTGCCGATGCGAGCTCCGCTGGATTCTCAGTTGGCACCAGAGTGGTACCGTCTCGATGACAAGAAGGAAGAGAAAATCAAAGGCGAGCTGATGCTCGCGGTCTGGATCGGAACTCAAGCTGACGAGGCCTTTCCTGACGCGTGGCAGTCCGATGTGGCGAATGCAGCCGCAGATGCCTCAGTGGCCGCGAGCCCGCATGTGCGGTCGAAAGTCTACCATGCTCCGAGGCTCTGGTACGTGCGAGTCAACATCATCGAGGCGCTAGATATTGAAATAGCCGACAAGGACCGCACTCCGGAGGTGTTCGTCAAGGCGCAGATCGGAGACCAGCACTTCAGGACGAGAAAAATGAAGTCGCGGAGATCCAAATATTTCTGGAATGAAGAAATAATGTTTGTGGCTGCCGAGCCTTTCGAGGATCAGCTAGTTCTGTCGGTTGAAGATCGCGTCAAGCTGGATAAAGATGAGGTGATCGGTCGAGTTACTATTCCGCTTTTTAAGGTGGAGAAGCGAGTCGATGACCGGACATTCCATAGCCGGTGGTTCAATCTTGAAAAGCCGAATGGTGCTGATGCCGATAAACCGAAGAAGGACGCTGCCGATGGCGATAAGGCCAAGAAGGACAAGCCCGCGAGCCGCATTCATTTGCGGGTATTCTTGGAGGGAGGATATCACGTGCTAGATGAGTCGACGCAGTATAGCAGCGACTTTAGACCGTCAGCGAAGCAGCTGTGGAAGCCGGCTGTCGGCGTGCTCCAGGTCGGCATACTCGGGGCAACCGGGCTTCTTCCGATGAAAACAAAAGATGGAAAGGGCATGACCGATGCGTACTGCGTGGCGAAGTACGGGAAGAAGTGGATTCGGACTCGAACCATGATCAACAGCCTGTGCCCACGGTTCAACGAGCAGCACACCTGGGAGATCTATGATCCGGCAACCGTCCTAACGGTCGGCGTTTTCGACAACTGCCAAATTGGCGAGAAGGGAAATGGTAAGAAAGATGCAAAAGATTCAAAAGACGGAAAGGAGGGACAAGACGCTGTTATCGGAAAGATCCGAATCCGGCTCTCGACACTCGAAACGGGTCGCGTGTACACCCATTCCTACCCGCTTCTCGTCCTGCAGCCGTCGGGGTTGAAAAAGATGGGGGAGCTTCATCTTGCCGTAAGGTTCTCCTCGACGTCGTTCGTCGGCATGATGTCGATGTACGCGCGGCCCCTGCTTCCGAAGATGCACTACGTCCGCCCGCTGATGCTATTTCAGATCGACGTGCTCTGCCACCACGCCATCCAGATCCTTGCGGCGCGGCTGGGCAGAATGGAGCCGCCGCTACGAAAAGAAGTCGTGGAGTTCATGTCCGACGTCGACTGTCACTTATGGAGCATGCGGCGGAGCAAAGCAAACTTCTTCAGGCTGACGTCGGTCTTCTCGCGCTTGTTCGAGGCCGGGAAGTGGTTTAAGGCGATATGCACGTGGACGAAACCTGTCGCGAGCGTTCTGGTTCACGTTCTGTTCACAATGCTTGTGTGCTTTCCGGAGCTGATACTCCCGACGGCGTTCTTGTACATGTCCTTCATAGGGCTCTGGAACTATCGGCACCGAGCACAGTACCCTCCACACATGAACACGAAGCTCTCCCAGGCGGAGAACGCCGACCCTGACGAGCTCGACGAGGAGTTCGATGCGCTCCCGACGAGCAAAGAGGCGGAGGTGGTGAAGAAGAGGTACGACCGGCTGAGGTGCGTCGCGGGGAAGGTGCAGATGCTGGTGGGGGACGTCGCGACGCAGGGGGAGAGGATCCAGGCTCTGATCAGCTGGAGGGACCCGCGGGCGACGTCGGTGTTCGTGCTGCTCTGCTCCGTGGCGGCGGCCGTCGCGTACGTGACGCCGCTCCGCGTCGTGGTCGCGCTGCTGGGGTTCTACACCATGAGGCACCCGCGGTTCCGGCAGAAGACGCCCTCCGCGCCGGCAAACTTCTTCCGGCGGCTGCCAGCGAGAATAGATAGCATGCTATGA

>Ancom010265

ATGAGTAATTACAAGCTAGGGGTGGAGGTTACAAGTGCGCACGACCTAATGCCCAAGGATGGGCAAGGTTCTGCTAATCCCTGTGTGGAACTACACTTTGAAGGCCAGAAATTTCGCACCACCATCAAGGAAAAGGACCTCAACCCGGTCTGGAATGAGTGTTTCTATTTCAATGTTGCAGACCCCGAGAACCTCCCCAACCTTGCTCTCGAAGCCTATATCTACAATGTAAGCAAAACCATGCACTCTAAGTCCTTCCTAGGCAAAGTCCGGATTGCAGGCACCTCTTTTGTTCCCTTCTCCGATGCTGTTGTAATGCATTACCCGCTGGAGAAACGTGGGATCTTCTCCCGTGTAAAAGGAGAGTTGGGGCTAAAAGTATTCCTTACTGATGATCCCTCGATCAGACCTTCGGATCCTCTTCCATCTTTTGATCCTGTAGTTAATAATCCTCCTCAAGTACCAAATCCAATATTCGATCCCCCGAAAGAACGGATGTCTGAAAAGAGGCATATGTTCCACACTGTCCCAAAAGAAGTCCATCACCATTCTACGGCTCCAATTAGTGAGCAGTCAGTGAAGTACGCGACTGATCAGATGAAACCTGAGCCGCCCAAAATTGTTAGGATGTACTCGGCTGCATCACAGCAACCGGTTGATTATGCGCTCAAAGAGACCAGTCCATTTTTGGGGGGTGGGCGGGTTGTTGCCGGCCGGGTCATACGTGCAGATAAGCCTGCAAGCACATATGACCTTGTTGAACAGATGCAGTATTTATTTGTTCGTGTGGTCAAAGCGCGGGATTTGCCAGCCATGGACGCATCTGGGAGCCTTGATCCTTTTGTGGAAGTGAGAGTTGGTAACTACAAGGGAATAACTAAGCACTTTGAGAAAAAGCAGAATCCAGAATGGAACGAAGTCTTTGCTTTCTCGCGAGACCGGATGCAGTCCTCAGTCCTTGAAGTCGTGGTCAAAGACAAAGATCTGGTGAAGGACGATTTTGTTGGGCTAGTGCGGTTTGATTTAAATGATATCCCAACCCGGGTCCCTCCTGATAGCCCGTTGGCACCAGAGTGGTACAAACTTGAAGGCAAGAGGGGAGATAAAACGAAGGGTGAGTTGATGCTTGCTGTTTGGATGGGCACGCAAGCTGATGAGGCTTTTTCTGATGCATGGCATTCGGATGCTGCGGCACCAATTGATTCCGCAGTCATAAACGCTCATATCAGGTCGAAGGTCTACCACGCGCCGAGACTGTGGTACCTTCGGCTCAACATCATTGAGGCCCAAGATATTATTATACCAGATAAGACTCGTTTTCCTGATGTGTATGTTAAGGTCCAATTAGGAAATCAGTTTTTGAGGACAAAGGCAGTTCAGGCCCGAACATTTAATCCGCTCTGGAATGAAGACCATATGCTTGTGGCTGCTGAACCATTTGAGGATCCCCTCATTCTTTCTGTCGAAGATCGTGTCGGTCCTAATAAAGACGAGACAATTGGGCGTGTTATTATACCGCTGCAATCTATTGAGAAGCGGGTCGATGACCGTGTTCTCTATGGCCGGTGGTTTAACCTTGAGAAGCCATTTATAGTTGATGTGGATCAGTTGAAGAAGGACAAATTTTCGAGTCGAATTCATCTTCGTGTTTGTTTGGAAGGAGGATATCATGTTCTTGACGAGTCAACTCACTACAGCAGTGACCTTCGACCTACAGCTAAGCAGCTTTGGAAACCATCAATAGGTCTGCTTGAGCTCGGTGTACTTAGTGCGGACGGCCTTCATCCTATGAAAACACGAGATGGAAAGGGAACTTCAGATACGTACTGTGTGGCCAAGTACGGCCAGAAGTGGGTTCGAACCCGAACGATCATCAACAGCCTGAGCCCTAAGTACAACGAGCAGTATACTTGGGAAGTCTATGACCCATCTACCGTCCTCACTGTCGGAGTTTTCGACAATTGCCAACTTGGCGAAAAGGGCGGAAACAGTAACGGTAACAAAGATGTCAAGATAGGCAAGGTCCGCATCCGCCTCTCCACTCTCGAAACCGGGCGGGTGTACACACACTCTTACCCACTTCTGGTCCTGCACCCTTCGGGGGTTAAGAAGATGGGCGAGCTCCACCTTGCCATACGCTTCTCGTCCACATCATTGATCAACATGCTATACATTTACTCGCGTCCTCCGCTGCCAAAGATGCATTACACCCGCCCATTAACCGTGATGCAGCTTGAGATGCTGCGCCACCAAGCTGTTCAGATCGTCGCTGCCCGTCTGAGCCGTATGGAGCCGCCCTTAAGAAAGGAAGTGGTGGAGTACATGTCTGACGTCGACTCTCACCTTTGGAGCATGCGGAAGAGCAAAGCGAACTTCTTTAGGCTGATGTCCGTCTTCTCAGGCCTGTTTGCTGCAAGCAAATGGTTTGGGGACGTATGCGCGTGGAAGAACCCTATCACGACTGTACTGGTCCACGTCCTCTTCATAATGCTCGTTTGCTTTCCCGAGCTGATACTCCCTACAATATTCCTTTACATGTTCCTGATCGGGCTGTGGAACTACCGGTACCGCCCGCGCTATCCTCCCCACATGAACACGAAAATATCGCACGCTGAGGCCGTACACCCCGACGAGCTGGACGAGGAGTTCGACACGTTTCCGACGAGCCGCAGCGCGGAGTTGGTTAGAATGAGGTACGACAGGCTGAGGAGCGTGGCAGGAAGGATACAGACAGTGGTGGGCGACATGGCGACGCAGGGGGAGAGGATCCAGGCGCTGCTGAGCTGGCGGGACCCGCGGGCCACTGCGATTTTCGTGCTTTTCTGCCTGATCGCCGCAATCGTGCTGTACGTGTCGCCGTTCCAGGTCTTGGCCGCACTGGTGGGGTTCTACATCATGAGGCACCCGAGGTTCCGCCACAGGCTGCCCTCGGCGCCGGTGAACTTCTTCCGGCGACTGCCTGCGCGGACTGATAGCATGCTGTAA

>Ancom011245

ATGAAGGTGGCGGTGGAGGTGGCGGACGCGAGCGACCTCATGCCCAAGGACGGGCACGGATCCGCGAACCCGTTCGTCGAAGTGGATTTCCAGGGGCAGCGCCAGCGCACCCGAACCAAGTTCCGCGACCTCTCCCCCTCCTGGAACGAGACCCTCGTCTTCAACCTCTCCGACCCCTCCCTCCTCCCCTCCCTCCCCATCGACGTCACCGTCTACCACGACCGCCGCGCCGCGGCGGCGCCGGAGGGGGGAGGAGGGGGGCACCACCGCAACTTCCTCGGCCGCGTCCGCATCTCCGGCGTGTCGGTGGCGCCCTCCCCCGCCGAGGCCGTCGTGCAGCGGTTCCCCCTCGACAAGCGCGGCCTCTTCTCCANGAGGCGGAGAAGAAGAAGACGAAGAAGAAGAAGGCCGAGCAGCAGGGCGCAGGAGGGGGAGGCGGAGGGGAAGGAGCCGCCGGCGCCGGCCCCGCGCGTGTTCTTCTCCGTCGGCGCCGCCCCTCCTCCCGCCGCCGCCGGCGCGCAGAAGCCCCCGACCAACGTGTTCTACGACGAGAAGCCCGCGCGGGCGGTGCCGCCGCCGCCGGGGGCGACGGTGGTGCAGGCGCGGCCTCCGGGGGGCGCCGGAGCGCCGGGGGCGCCGCGGCCGGAGTTCGGGCTGGTGGAGACCCGGCCGCCGCTGGCCGCGCGGCTGGCCCGCAGCGGCCGGCTGGGCGCCCGGGACAAGATCTCGTCGACGTACGACCTGGTGGAGCCGATGCACTACCTGTACGTGAGCGTGGTGAAGGCCCGCAACCTCCCCGCGAAGGACATCACCGGGTCCCTGGACCCGTACGTGGAGGTGAAGCTGGGGAACTACAAGGGCATCACGAAGCACCTGGAGAAGAACCAGAACCCTGTGTGGCACCAGGTGTTCGCCTTCTCCAAGGACCGCATCCAGGCGAACATGGTGGAGGTCACCGTGAAGGACAAGGACCTCGTGAAGGACGACTTCGTGGGGCGCATCGTCTTCGACCTCACCGACGTGCCCCTCCGCGTGCCCCCCGACTCCCCGCTCGCCCCGCAGTGGTACAGGCTCGAGGACAAGAAGGGCGACAAGCTCACCACCGGCGGCGGGCCCGAGCTCATGCTGGCCGTGTGGATCGGCACCCAAGCCGACGAGGCCTTCCCCGAGGCCTGGCACTCGGACGCGCACTCCGTCTCCCTCGACGCCCTCGCCAACACCCGCTCCAAGGTCTACTTCTCCCCCAAGCTCGTCTACCTCCGCGTCCTCGCCATCGAGGCCCAGGACCTCGTCCCCGCCGACAACTCGGCCACCAGCTCCGTCAACCCCGTCTGGAACGAGGAGTTCCTCTTCGTCGCCGCCGAGCCCTTCGACGAGCCGCTCGTCATCACCGTCGAGGACCGCGTCGCCCCCGGCCGCGACGAGCCCCTCGGCCGCCTCACCCTCCCCGTCTCCGTCGCCGTCCCCCGCAACGAGCACAACAAGCCCGTCGAGCCCAAGTGGTTCAGCCTCTCCCGCCCCACCGCCCTCCTCGACGAGGAGAAGAAGGAGTCGACCTCCAAGTTCTCCAGCAAGGTCCACCTCCGCCTCTCCCTCGACCTCGGCTACCACGTGCTCGACGAATCGACGCACTACAGCAGCGACCTCCAGCCCTCGTCCAAGAACCTCCGCAAGCCCAGGATCGGCATTCTGGAGCTGGGCATCCTCAGCGCCCGCAATTTGGTCCCCATGAAGGCCAAGGACGGCCGCACCACCGACGCCTACTGCGTCGCCAAGTACGGCAACAAATGGGTCCGCACGCGCACGCTGCTCGACACGCTCGCGCCGCAGTGGAACGAGCAGTACACTTGGGAGGTCTTCGACCCCTGCACCGTCATCACCATCGCCGTTTTCGACAATTGCCACGTCGCCGGCCACAACGGCGGCGGCGACGTGCGCGACCAGCGAATCGGCAAGGTCCGGATCCGGCTCTCGACCCTCGAGGCCGACCGGATATACACGCACTTGTACCCCTTGCTGGTTCTCCAAACCTCCGGCCTCAAGAAGACCGGCGAGCTCCACCTCGCCGTCCGGTTCACGTGCACCGCGTGGGTCAACATGGTGACGCTCTACGGGAAGCCGCTGCTCCCGAAAATGCACTACGTGCAGCCGATCTCCGTCCTCCAGCTGGACTACCTCCGCCACCAGGCGATGCAGATCGTGGCGGCCCGGCTGGTCCGGGCGGAGCCGCCGCTCAGGCGCGAAGTGGTCGAGTACATGCTCGACGTGGATTCGCACATGTTCAGCCTGAGACGGAGCAAGGCCAATTTCTACCGCATCACGTCTCTCTTCTCCGGCATGGCGGCGATCGGGAGATGGTACAACAGTATTCGGAACTGGCGGAATCCGATAACTACGGTGCTCGTCCATGTCCTCTTCTTGATACTCGTGGGCTACCCGGAGCTGATCTTACCGACCATCTTCCTCTACCTGTTCATGATCGGCATATGGAGCTACCGGTTCCGGCCGCGCCACCCGCCGCACATGGACACAAGGTTATCCTACGCCGAGATGGCGCACCCGGATGAGCTTGATGAGGAGTTTGATACGTTCCCGACGTCCAAGGCGGCGGATGTGGTGAGAATGAGGTACGATCGGCTGCGGAGCGTGGCGGGGCGGGTGCAGACGGTGGCGGGCGACCTTGCGACGCAAGGGGAGCGCGCGCAGGCCCTCCTGAGCTGGCGGGACCCACGGGCCACCGCAATCTTCATAATCATCTCCCTCATCATAGCCATAGTGCTCTACGTCACGCCGTTCCAGGTCATCGCGGTGATCGTCGGGCTCTACCTGCTCCGCCACCCCCGGTTCCGGAGCAAGATGCCCTCCGTGCCGTACAATTTCTACCGGAGGCTACCCTCAAAGTCCGACATGCTGCTCTGA

>Ancom011746

ATGAGCAGCAATCTCACACTGGGGGTCGAAGTCGTCAGCGCCCGCGAACTTGCCCCGCCCCGAGACGGCGACGGCACCGCCAACGCCTTCGCGGAGCTCCACTTCGACTCCCAGCAGTCCCGCACCACCGTAAAGGACCGCGACCTGAGCCCGGTCTGGAACGAGGCCTTTCGCTTCCGCGTCTCCTCCGACCCCTCGTCCCTCCCCGAGCTCCGCCTCGACGCCCTCCTCCACTTCGACTCCCAGCAGTCCCGCACCACCGTAAAGGACTACGACCTGAGCCCGGTCTGGAACGAGGCCTTTCGCTTCCGTGTCTCCGACCCCTCGTCCCTCCCCGAGCTCCGCCTCCACGCCCTCGTCTTCCACCTCAACCGGTCCACCGGCTCCAGGTCCCTCCTCGGCAAGGCCTGCATCTCCAGCACCTCCTTCGTTTCCTTCGCCGACGCCGCCACTCTTTACTACCCCTTGGAGAAGCGCTCCCTCTTCTCCCGCGCCCGGGGGGAGCTCGGCCTCAAAGTGTACTTAACTAACTCTTCAATCCCAGCCGTTGATCCTCTGATCCAGCCCGGCGCTGCTTTTAATCAGGACCCTCCATCACTTGCGGACAAAACAGAGACCGTTGCTCGTAATAACAGCTTCCTCCACCTCTCCAGAGAGCAAGTGGCGGCGGAACCGGCGTTGACGTTTGCGGTGAAAGAGATGAAGCCGGAGCCGCTCCGCGCCGTGTCGGTGCCGGCGTTCCCGTCGGCTTCGTTCCGGCAGCCGTTCGACTTCCAGCTGAAGGAAACCAACCCTCCGCTCGGCGCCGGCCGCGCCTTCGGGGGGCGGGTCTTCCCCGGCGGCCGGACGGCCGGGACCTACGACCTCGTGGAGCGGATGCACTACCTCTTCGTCCGCGTCGTGAAGGGCCGGGAGCTCCCCCCGCGCGACGTCGCGGGGAGCATCGACCCGTTCGTGGAGGTGCGGCTCGGGAACTACCGGGGGACGACGAAGCACTTCGAGAAGAAGCACAACCCGGAGTGGGACGAGGTGTTCGCGTTCTCGCGGGAGAGCGTGCAGTCGTCGCTGGTGGAGGTGGTGCTCAAGGATAAGTCGCTCGCGAAGGACGGCGTCGTGGGCCGGGTGCACTTCGACCTGAACGAGGTGCCGATGCGCGTCCCGCCGGACAGCCCGCTGGCTCCGGAGTGGTACCGGCTGGAGAACGTCAGGGGGGAGAGGATCAAGGGCGAGCTGATGCTGGCGGTGTGGATCGGGACGCAGGCCGACGAGTGCTTCCCGAACGCGCTGCACGCCGACTCCGCTCCGGTCGATCCGATCCTCGCGAGCGTCCACATCCGGGGGAAGGTCTACCACGCGCCGCGGCTGTGGTACGTCCGGGTGCACGTCGTGGAGGCGCAGGACGTGTTCCTGTCGGACCGCAGCCGGGTGGGCGAGGTGTTCGTCCGGGCGAAGCTGGGGAGCCAGGTGCTGAAGACGCGGGTGGCGCCGTGCCGGACGCCGCCGTACTACCGGTGGGACGAGGATCACCTGTTCGTGGCGGCGGAGCCCTTCGAGGAGGAGCTGATCCTGTCGGTGGAGGACCGCGTGGGGCCGAACAAGGAGGAGGTGATCGGCCACTGCCGCGTGCCGCTGGCGCAGCTGGAGCGGCGGTTCGACGAGCGGGAGGTCGCGTCGAGGTGGTTCGGCCTGCAGAGGCACGGCGGCGGCGCGGAGGCGATCAAGGAGGAGAAGTTCTCCAGCAAGCTCCACCTCCGGCTCTGCCTCGAGGGCGGCTACCACGTGCTGAGCGAGTCGACGCACTACAGCAGCGATCTCCGCCCGACGGCGAAGCACCTGTGGAGGGATCCGATCGGGCTGCTCGAGCTGGGCGTCCTGAACGCCGACGGCCTCACCCCGATGAGGACGCGGGACGGCAGGGGCACCTGCGACCCCTACTGCGTCGCCAAGTACGGCCAGAAGTGGGTCCGGACGCGCACCGTCGTCGACCGGCTCTCGCCGCGGTTCCACGAGCAGTACACGTGGGACGTCCACGACCACGCCACCGTCCTCACCGTCGGCGTCTTCGACAACTGCCAGCTCGAGAGGCGGCCGGCCGGCGCGGAGGCCGTCAGGGACGCGATCGTCGGGAAGGTGCGGATCCGGCTCTCGACCCTCGAGACCGGCCGCGTGTACACGCACTCCTACCCGCTCCTCGTCCTCCACAACTCCGGCGTCAAGAAGATGGGCGAGCTCCACCTCGCGATCCGCTTCTCCGCGACGTCCTTCTCCGACACGCTGTACGCGTACTCGCGCCCGCTGCTGCCGCGGATGCACTACGTGCGCCCGCTGTCGATGGTGCAGCAGGAGGTGCTGCGCCACCAGGCGGTGCAGATCGTGGCGCTGCGGCTGAGCCGGATGGAGCCGCCGCTCCGCCGGGAGGTGGTCGAGCACATGTCGGACGCGCACGCGCACCTCTGGAGCATGCGGCGGAGCAAGGCCAACTTCTTCCGGCTCATGTCGGTCTTCTCCGGCGTGTTCGCGGTCGCCAAGTGGTTCGCCGACGTCTGCTGCTGGACGAATCCGGTGACGACCGTTCTGGTGCACCTTCTGTTCGTCATGCTGGTCTGCTTCCCGGAGCTCATCCTCCCGACCTTCTTCCTCTACCTCTTCCTGATCGGCCTGTGGAACTACCGCCGCCGGCCGCGCCACCCGCCGCACATGAACACGCGGATATCGCACGCCGACGTCGCGCACCCGGACGAGCTCGACGAGGAGTTCGACACCTTCCCGACCAGCCGCAGCCCGGAGATCGTGCGGATGAGGTACGACCGGCTGCGGAGCGTGGCGGGGAGGATACAGACGATCGTGGGGGACGTCGCAACGCAGGGCGAGCGGTGCCAGGCGCTGGTCAGCTGGAGGGACCCGCGCGCGACCGCCGTGTTCCTGCTCTTCTGCCTCTGTGCTGCGCTGGTGCTCTACGTGACGCCGTTCCCCGTGGTGGCGCTGCTCCCCGGCTTCTACTGCATGAGGCACCCGCGGTTCCGGAACCGGCTGCCGGCCGTGCCCATGAACTTTTTCCGCCGATTGCCGGCGAGGACCGACTGCTTACTATGA

>Ancom015662

ATGAGCCACGACGACCTGCACCACGAGGACTACCAGCTGAAGGACACGAACCCGCTGCTCGGCGAGCGATGGCCCACGGCGGGAGGCGGAACCCGACAGGGCCTGGGAGGCGGTGGTTTCAGCGGGTGGCTGAGCAGCGACAAGCTGACGAGTACGTACGACCTCGTGGAGCAGATGCACTACCTCTACGTCCGCGTCGTGAAGGCGCGGGACCTCCCTCCCAATCCGCTCACGGGAAGCTGCGACCCTTACGTTGAAGTCAAGCTCGGCAACTACAAGGGCACCACAAGGCACTTCGACAAGCGGACTAATCCTGAATGGAACCAGGTTTTCGCGTTCTCCAAGGACCGGATCCAATCGTCGACGCTCGAGGTTTACGTGAAGGATAAGGAGATGGTGGGGAGAGATGACTACATGGGCAAGGTGGTGTTTGATTTGAATGAGGTGCCGACACGGGTCCCGCCTGACAGCCCATTGGCCCCGCAGTGGTACCGGCTGGAGGACAGGCGGGGTGAGGGGAAGGTGCGGGGGGAGGTCATGCTGGCAGTCTGGATCGGGACGCAGGCCGACGAGGCTTTTCCTGAGGCCTGGCACGCAGATGCAGCCTCTGTCCACCCGGAAGGGGTGGCCAATATCCGCTCCAAGGTATATGTCTCCCCTAAGCTTTGGTACCTCCGAGTTAACGTCATTGAGGCCCAGGACGTGCAGCCTAACGTCCGGGGCCGGACACCAGAAGTTTTCGTGAAGGCGCAAGTCGGAAACCAAGTGCTCAAGACGAAGAGCTGTGTGGCGGCCACCCTCAACCCACTGTGGAACGAGGATCTTATCTTTGTTGTGGCCGAGCCATTTGAGGAGCAACTTATTATTAGCGTCGAGGATAGGGTGAGCCCGAGGAAGGATGATCTGCTTGGCCGGGTAGCATTGCCATTAACCTTGTTCGAGAAGCGGCTTGACCACCGGCCGTTCGTCCACTCTCGCTGGTTTGACTTGGAGAAATTCGGGATCGGCGTGCTCGAAGGCGAGACACGAAGGGAGCTCCGGTTCGCAACCCGGGTCCACCTCCGTGTCTGCTTAGAAGGGGCCTACCACGTGATGGACGAGTCAACAATGTACATCAGCGACCAGCGGCCCACGGCCCGCCAGCTATGGAAGCCGCCGGTTGGAGTGCTGGAGGTGGGCATTTTGGGCGCGAAGGGGCTGCTGCCGATGAAGATGCGGGATGGAAGGGGGACCACCGATGCCTATTGCGTTGCAAAGTATGGGCAGAAGTGGATCCGGACCCGGACGATGGTCGGGTCCTTTAGCCCTAGCTGGAACGAGCAGTACACATGGGAGGTGTTCGACCCGTGTACGGTGATCACCATCGGGGTCTTCGACAACTGTCACCTGGGCAACGGGGGCGGCGGAGCTGTGAAAGATTCGAGAATTGGGAAAGTCAGAATCCGGCTCTCAACCCTAGAGACCGATAGAATCTACACACAGTCGTACCCTCTCATCGTGCTCCAGCCTTCGGGAGTAAAGAAGATGGGTGAGCTCCAGCTTGCGGTGCGGTTCACGTGCCTCTCTTTCACTAACATGATCTACCTCTACGGCCAGCCGCTCCTACCCAAGATGCACTACCTCCATCCCTTTACTGTAAACCAGCTCGACAACCTCCGCTATCAAGCCATGAGCATTGTTGCGGCCCGGCTTGGCAGGGCCGAGCCACCATTGAGAAAGGAGGTCGTTGAATACATGCTCGATGTGGAGTCACACATGTGGAGCATGCGAAGGAGCAAGGCCAACTTCTTCCGGATCATGTCACTGCTCTCCGGAACAATCGGCACGTTCAAGTGGTTCAGCGACGTTCAAAGATGGAAAAACCCGATAACGACCGTGTTGGTCCACGTGCTGCTTTTGATCCTCGTCTGCTATCCGGAGCTGATATTGCCCACCATATGCCTGTATATGTTCTTGATAGGGTTGTGGAATTACAGATTTAGACCAAGGCAGCCGCCGCATATGGATACAAAACTCTCGTGGGCGGAGGCAGTGCACCCGGATGAGCTGGACGAAGAGTTTGATACTTTTCCTACTTCGAGGCCGCAGGATGTAGTTTTTATGAGGTATGACAGGCTGCGGAGTGTGGCAGGGAGGATACAGACAGTGGTGGGTGACATAGCGACGCAGGGGGAGAGGGTGCAGTCGCTGCTGAGCTGGCGGGACCCGAGGGCAACATGTCTGTTCGTATTTTTCTGTCTCTGCGCGGCGGTGGTGCTCTACGTGACTCCCTTCAAAGTGGTGGCTCTGGTGGCAGGACTCTACATGCTCCGCCACCCTCGGTTCCGGAGTAAGCTGCCATCGGTGCCGAGTAACTTCTTCCGACGTCTGCCGTCGCGGGCCGACAGCATGTTGTGA

>Ancom017114

ATGAAGAAGCTCCGGGATCCTTCTCCGCCGCACTCGCAGCTAGGGTTTCACCTCCTTCGATCGATCGAGGTTTCGCGACAGTTTGAGGAGAAGATGCAGAGAGTAGTAGTGCCGCCTCGTCCCGAGGAGTACTCCTTGAAGGAGACCACCCCGCACCTCGGCGGCGGTGGGGCCGCGGGCGACAAGCTCACGACCACCTATGATCTCGTTGAGCAGATGCAGTATCTCTACGTTCGGGTCGTGAAGGCCAAAGACCTGCCCACGAAGGACGTGACGGGCAGCTGCGACCCCTTCGTCGAGGTCAAGCTCGGCAACTACAAGGGAACCACCCGCCATTTCGAGAAGAAGACCAACCCCGAATGGAACCAAGTCTTCGCTTTCTCGAAAGAGCGGATTCAGGCGTCGCACTTGGAAGTCGTGGTCAAGGACAAGGATTTCGTGAAGGACGACATTATCGGGCGGGTGGTTTTCGACCTCACTGAGATCCCTAAGCGGGTCCCGCCCGACAGCCCGTTGGCCCCCCAGTGGTACCGGTTGGAGGACCGGAAGGGGGATAAGGTGAAGGGGGAGCTCATGCTGGCTGTTTGGTGGGGGACTCAGGCCGACGAGGCTTTTCCTGATGCCTGGCATTCTGACGCCGCGACCGTCCCCAGCGACGGCCTCGCGAATATTAGGTCGAAGGTGTATCTCACTCCTAAGCTCTGGTACGTGCGTGTTAATATTATCGAAGCCCAGGATCTTCAACCTAGCGATAAGAGCCGGTTCCCCGAAGTCTATGTTAAGGCCATATTAGGGAACCAAGCTCTTAGGACCCGTGTATCTCCGAGCAGGACGATTAATCCGATGTGGAACGAGGATTTGATGTTCGTGGCGGCCGAGCCGTTTGAGGAGCACCTGGTTCTAAGCGTAGAGGATCGGGTCGCACCCAACAAGGACGAGGTATTGGGGAAGGCAATCATCCCGTTGCAAAATGTGGACAGAAGGCTTGATTATAAGGCCGTAAACACCCGGTGGTACAATCTTGAAAAGCATGTGACAGTCGATGGCGAGCAGAAGAAGGACACCAAGTTTTCTAGCCGGATTCATGTGCGAATTTGCTTAGAAGGCGGGTACCATGTATTGGATGAGTCAACTCACTATAGCAGCGACCTAAGACCCACCGCGAAACAGTTGTGGAAGCAGAGCATCGGAGTTCTCGAATTGGGTATTTTGAGTGCTCAGGGGTTGCTGCCGATGAAGACTAAAGATGGGCGTGGGACGACAGATGCTTATTGTGTTGCCAAATACGGGCAGAAATGGGTCCGCACGAGAACCATCATAGATAGTTTCACTCCCAAATGGAACGAGCAGTACACGTGGGAGGTCTACGATCCGTGTACTGTTATAACGATCGGAGTGTTCGACAACTGTCACTTACAAGGGGGCGAGAAGGCGGCGGGTGCGCGGGATAATAGAATCGGGAAGGTGCGGATCCGTCTCTCTACTCTCGAGACAGATCGGGTTTACACCCATTCTTACCCTCTCATCGTGTTACTTCCTTCGGGAGTGAAGAAGATGGGAGAGGTTCAATTGGCCGTCCGATTCACGTGCTCCTCTCTCCTCAACATGTTGCACCTGTACTCCCAGCCGTTGCTGCCGAAGATGCATTACCTTCACCCTCTGTCCGTGATGCAGCTTGATAATCTGAGGCACCAGGCCATTCAGATAGTCTCGATGAGGCTAGGCCGTGCTGAGCCACCTCTGAGAAAGGAGGTAGTGGAGTATATGCTCGACGTGGATTCACACATGTGGAGCATGCGAAAGAGTAAAGCCAACTTCTTCAGAATCATGGGCGTGTTGGGCCCTTTGATCGCCGTGGGGAAATGGTTTGATCAGATTTGCCACTGGAAAAACCCTTTGACTACAATACTAATCCACATCCTCTTTGTGATATTGGTTTTGTACCCCGAACTGATTCTCCCGACGATCTTTCTCTACCTCTTCTTGATCGGGGTTTGGTACTACAGATGGAGGCCGAGGCAGCCGCCCCACATGGACACGCGTCTCTCCCACGCGGAGACCGCCCACCCAGATGAGCTCGATGAGGAATTCGACACTTTCCCGACATCTCGGCCACCCGATATCATCAGGATGAGGTACGACCGTCTGAGGAGCGTCGCGGGGAGAGTACAGACGGTCGTGGGCGATTTGGCTACGCAGGGAGAGAGGCTGCAGTCGCTCTTGAGCTGGCGGGACCCGCGAGCCACCGCGCTGTTTGTCGTGTTCTGCCTGATCGCCGCCATCGTGCTCTACGTCACGCCGTTCCGTGTTGTAGCCTTTTTGACGGGCTTATATGTGCTAAGACATCCGAGGTTTCGCCATAAGCTCCCGTCGGTTCCGCTCAACTTCTTCAGGAGATTGCCCGCGCGAACCGATAGCATGTTGTGA

>Ancom019606

AATCAAATAAACACAATGAGCAACAACCTGAAGCTCGGGGTGGAAGTGGTGAGCGCCCATGACCTGCTTCCCCGCGACGGCGACGGCACCGTCACGACGTTCGTCGAGCTCAGCTTCGACGGGCAGCGCCACCGCACCGCGGTCAGGGACCATGACCTCAACCCCTACTTCAACGAGCGCTTCTTCTTCGCCGTCTCCGACCCATCCTCCCTCCCCTCCCTCGTCCTCGACGCCACCGTCTTCCACCTCCACCGCCCCTCCCTCTCCCGATCCTTCCTCGGCCGCGTCCGCATCCCCGCCTCTTCCGTCCCCGCGTCCCCCTCCGATTCCCCCGTCCTCTACTACCCTCTCGAGAAGCGCGGCGGCGGCAGAGGCGGCGGGGTCTTCTCCCGCATTCGCGGCGAGCTGGGGCTCAGAGTGTTCCTCACCGACGACCCGTCGGTTCAGCCCTCCGGCCAGAACCTTCGGCAGGATCAGTTCGCCGACATCCCACCGGAGCCGGACATGTCGACCTTCTTCCGCCTCTTCCGATCACAGCAGCGCCAGCAGCAGCGGTCGGATCAGGCACCGCCGTCGAGGGTTATGCGGATGTTCTCGTCACTGTCGTCGCGGCAGCCGGCCGAATTCCAGATCAAGGAGACGAGCCCGGCGTTGGGCGGCGGCCGCATCGTGCGCGGCCGCGTTGTCGTCCCGGGGGACAAGCCGGGCGCCTTTGACCTAGTGGAGAAGATGGAGTTCCTCTTCGTGCGCGTGGTGAAGGCACGGGACCTCCCGGCGAAGGATGTGACCGGCAGCCTCGACCCCTACGTCGAGGTGATCATCGGCAACTACCGGGGGACGACGTGGCACTTCGAGAAGAACCAGAACCCGGAGTGGAATGAAGTGTTCGCGTTCCCACGCGACCGCCTGCAGGCGTCGGCCGTGGCGGTGGAGGTCAGGGACCGGGACCTCGTCCGCGACGACTATGTTGGCGCGGTGCAGTTCGAACTCAACGACGTGCCGCTGCGGGCCCCGCCGGACGGCCCCCTGGCGCCGGAGTGGCACCGGCTGGAGGACAAGAGGGGGGGGCGGGACGTCGGCGAGCTGATGTTGGCTGTGTGGTTCGGGACGCAGGCCGACGAGTGCTTTCCCAGCGCTTTGCACGCCGACACGGCGGCAATCGACCCTGCGACCGCCAACACGCACGCCCGCGGCAAGGTCTACCAGGCGCCGACGATGTGGTACCTCCGGGTGAACGTCATGGACGCGCATGACGTGTACATGCCGCAGGGGGACCGGTCCCCGGAGGTGTTCGTCCGCGGCCGTGTCGGGAACCAGATGCTCAGGACGAAGATGGGCCGGCCCGGCGCTGCTGGGATCTTCAAGTGGAACGAGGTACATTTCTTTGTCGTGGCCGAGCCGTTCGACGACGAGCTGACCGTGTCGGTAGAGGACCAGGCCGGGCCGGACCAGGACCGGGTGATCGGCTACGTGAACATCCCGATTGCCACCGTCGACAAGCGGCCCGACGACCGCCGCACCTGGCCGAAATGGCTCGACCTCCGGAAGCCAACGCTGATCGACGTCGACCGCCTCCGCGAGGACAAGTTCGCCACCAAGCTCCAGCTCCAGATCAGCCTCGACGGCGGCTACCACGTCGTTGACGAGCTCATCCACTACAGCAGCGACTTCCGGCCCTCGTCGAAGCACCTGTGGAATGCAAAGAAGCCGATCGGCATGCTCGAGCTCGGCATACTCAGCGCCGTGGGGCTGCAGCCGATGAAAACTGCCCGCGACGGGCGGCCGACGTGCGACGCCTTTTGCGTCGCCAAGTACGGCCGGAAATGGGTCCGGACGCGCACTGTCGTCGACTCGCTGAGCCCCAAATTCAACGAGCAGTACGCGTGGGACGTCTACGACCACGCGACGGTGATCACCGTCGCCGTATTCGACAATAGTCAAGTACTTACAGGGAGCAGTACTAGAGACACGGCGATCGGGAAGGTCCGGATACGGCTGTCGACCATCGAGACGGGCCGTGTCTACGCGCACGCGTATCCGCTGCTGGTGCTGCACCCGTCGTCCGGGGTGAAGAAGATGGGGGAGCTGCACCTTGTGGTGCGGTTCACTGCGACATCTTTCGCAAGCATGTTGCACGCGTATGTGCGTCCGGTCCTGCCAAAGATGCACTACACGCACCCGATCACGCAGCTGCAGCAGGAGAACCTGCGGCTGCAGGCGGTGCAGATACTGGCGACGCGGTTGGGGCTGGCGGAGCCGCCGCTGCGGAGGGAGGTGGTGGAGTACATGTCGGAGGCGCAGGCGCACATGTGGAGCATGCGGCGGAGCAAGGCGCACTTCTACCGCCTCACGGAGGTGTTCGGTGGGGCCGTCGCGGCTGCCAAGTGGCTCAGGGACATCTGCCGGTGGGCGAATCCGGTCACGACGGTTCTCGTCCACCTTCTGCTCGTGATTCTCGTGTGCTTCCCGCATCTCATCCTCCCGACGGTCCTACTCCACTTGTTGGCCGTCGGATTGTGGAACTTCTGGTACCGTGCGCGGTACCCGCCGCACATGAACACGAAGATTTCTCACGTGCATGGGGTGAGCCCGGACGATCTTGACGAGGAGTTCGATCTCTTCCCCACGACCCAGAGGCCAGAAGTGGTTCGGAGTCGATACGATCGACTCCGATTCATAGCGGGGATGATGCAGAAGACGCTCGGTGAGTTTGCGACACAAGGGGAAAGGGTGCAGTCCCTCCTCAGCTGGCGGGATCCGCGAGCCACCGCAATTTTTCTCGTGTTCTGTCTCGTCACGGCGTTCGTGTTTCTAGTTGTTCCGTTCAAAGTGTTGGTGATTTGGCTAGGGTTTTACTGGATGAGGCACCCACGGCTGCGGCATAAGATGCCGTCCGTGCCGACGAACTTCTTCCGGCGGTTGCCGGCAAGGACTGATAGCTTGCTGTAG

>Ancom027649

ATGCTCGAAAGGAGTACCCAGNACCACCCGGCCCCCGCCCCCGCCCCCGCCCCCGGCCCCGCTCACGCGCCGCGGGTGATCTCGGGCCGGTTCGTGTCGTCGGCCGAGCCGGTCGAGCGGGTCCAGACCACCTACGACCTCGTGGAGCCGATGCAGTACCTCTTCGTCCGCGTCGTCAGGGCGCGGGGCCTCCGCCCGTGCGAGTCGCCCTACGTGAAGGTCCGGACCGGGTCCCAGTCGTTCCGGTCGAAGCCGGCGCGCGACTCCGGCTCGGGCGAGCCGGAGTGGAACCAGGTGTTCGCGCTGAGCCACGCGAAGCCGGAGCCGACGCTGGAGATCTCGGTGTGGGACGGCGCCCCCGCCGCGGCGGACGCGTTCCTCGGCGGGGTCTGCTTCGACCTCTCCGACGTGCCGGTCCGGGACCAGCCCGACGGCCCGCTAGCCCCGCAGTGGTACCGGCTCGAGGGTTCCGAGCCGGGCCCCCGCTCGGTGTCGGGCGACATCATGGTGTCGGTGTGGATCGGGACCCAGGCCGACGACGCGTTCGCGGAGGCGTGGATCTCGGACGCGCCGTACGTGAGCCACACCCGGTCCAAGGTGTACCAGTCCCCCAAGCTGTGGTACCTCCGCGTGTCGGTGATCGAGGCCCAGGACCTGCGGCTCCCCGCGGCCGCGGCCCCGGCCCCGGCCCCCTGCACGCCGTTCGACGTGCGCGTGAAGGTGCAGCTGGGCTTCCAGTCGGCGCGCACCCGCAGGTCGACGGTGAGCGGGAGCGGGTCGACGTTCTCGTGGGCGGAGGACCTGATGTTCGTGGCCGCGGAGCCGCTCGACGATCAGCTGGTCGTGCTCGTCGAGGACCGGTCGGCGGCCAAGGACCCGGCGCTGCTGGGCCTCGCGGCGGTCCCCGTCCCCTCCGTCGAGCAGCGCCTGGACGAGCGCCACGTGCCGGCCTCCAGGTGGGTCAACCTCGAGGCCGAGGCCGAGGGCGGGTACCGCGGGCGGGTCCACCTGCGGCTCTGCCTGGAGGGCGGGTACCACGTGCTCGACGAGGCGGCGCACGTCTGCAGCGACTACAGGCCCACGGCGAAGCAGCTGTGGAAGCCCCCCGTGGGGGTGCTGGAGCTGGGGATCCTCGGGGCGCGGGGCCTGCTCCCGATGAAGACCAAAGGAGGCGGAGGAGGCAAGGGCTCCACCGACGCCTACTGCGTGGCCAAGTACGGAAAGAAGTGGGTCCGGACGCGGACCGTGACGGACAGCCTCGACCCGCGGTGGAACGAGCAGTACACCTGGCAGGTGTACGACCCCTGCACGGTGCTGACGGTGGCGGTGTTCGACAACTGGCGCATGTTCGCCGATGCGGCCGGCGAGGAGCGGCCCGACTACCGCATCGGCAAGGTGCGCATCCGGGTGTCGACGCTGGAGAGCAACCGCGCGTACACGGCGTCGTTCCCGCTGCTCGTGCTGCTGCGGTCGGGGCTGAAGAAGATGGGGGAGGTGCAGCTGGCGGTGCGGTTCGCCTGCCCGGCGCTGCTGCCCGACACCTGGGCCATGTACGCGCAGCCGATGCTGCCGCGCATGCACTACCTGCGCCCCATCGGGGTGGCCCAGCAGGAGGCGCTGCGCGGGGCGGCAATTCGCACCGTCGCCGGGTGGCTGGCGCGGGCGGAGCCGCCGCTGGGCCCGGAGGTGGTGCGCTACGTGCTGGACGCGGACGCGCACGGGTGGAGCGTGCGGCGGAGCAAGGCGAACTGGTTCCGGATCATGGGGGTGGTGGCGTGGGCCGTGGGGCTGGCTCGGTGGGTGGACGACGTGCGGCGGTGGAGGAGCCCCGTGACGACGGTGCTGGTGCACGTGCTGTACCTGGTGCTGGTGTGGTATCCGGAGCTGGTGGTGCCGACGGGGGCGCTGTACGTGTTCCTGATAGGGGTGTGGTACTACCGGTTCAGGCCAAAGGGCCCGGCCGGGATGGACGCAAGGCTGTCGCAGGCCGACACGGTGGAGCAGGACGAACTGGAGGAGGAGTTCGAGCCGGTGCCCACGTCGGTGGAGGTGCTGCGGGTGCGGTACGAGAGGCTGAGGACGCTGGCAGGGAGGGTGCAGAGGGTGATCGGGGACCTGGCGGCGCAGGGAGAGCGGCTGCAGGCGCTGGTCAGCTGGAGGGACCCCAGGGCCACCAGGATATTCATCGCCGTCTGCCTCGCGGTGGCGCTGGTGCTCTACACGGTGCCGCCGAAGATGGTGGCGGTGGCGCTCGGCTTCTATTTCCTCCGCCACCCCATGTTCAGGGACCCCATGCCGCCCGCCGCCGTCAACTTCTTCCGCCGCCTGCCCAGCCTCTCAGACAGACTTTTGTAA

>AT1G04150

ATGACTGAAGCTAAAACCGGCACTGGCAATGAGAGGCTCGTGGTGGAGATAGTAGGGGCCCACAATCTCATGCCAAAAGATGGCGAAGATTCTTCGTCACCTTTTGTGGAAGTGCAGTTCGAGAACCAACGTCTCCGGACTAAGGTCAAACCCAAAGACTTGAACCCAATCTGGAACGAGAAGCTCGTCTTCCACGTTATTGACGTCAACGACCTCAGACACAAAGCCCTAGAAATCAACGTATACAACGAGAAGCGTTCTTCTAACAGTAGAAACTTCTTGGGAAAGGTTAGGGTTTTGGGTTCCAGCGTTGGAAGAGAAGGAGAGTCAGTCGTTCAGCTCTACACTCTTGAAAAGAGAAGCCTTTTCTCTTCCGTGCGTGGAGAAATCAGCGTCAAACACTACATGACCACAACGGCGGAGAACGGTGAGAATGTCCGTCGGGTTAACAGGTCGGGTGGTTCAAAGAAAAGCAAAAAAGTACAGAACGTGAGCTCTTCCATGGCGATTCAACAACAGCAGCAACAACAACAGCAACAAATTTCTCTTCACAATCACAACAGAGGAAATCAACAACAAAGTCAGCAGAACGGTCAAGGACAGAGGATGCTACCGTTTTATCCACACCAAAGTGAGATAAAGCCTCTCGTGATAACCGCTCTTCCCAGTCCAATGCCCGGACCCGGACCCAGACCCATCGTTTACTCTAACGGGTCGAGTGAGTTTTCGCTTAAAGAGACAAAGCCATGTCTTGGAGGAACAAGTAATGGTCTTGGCGGTTTGAGTAGTCACAAGGACAAAACAAGCTCCACTTATGATCTAGTTGAGCAAATGCAGTACCTTTATGTTAATATCGTGAAAGCTAAGGACTTGTCGGTATTAGGCGAAGTCGTGTCGGAAGTGAAATTGGGTAATTACAGGGGAGTTACTAAAAAGGTGAGTTCAAATTCCAGTAATCCAGAGTGGAATCAAGTATTTGTCTTTTCCAAAGAAAGAATTCAATCATCCGTAGTTGAGCTTTTCGTTAAAGAAGGTAATAAAGATGAGTATACGGGTAGAGTCTTGTTTGATTTAAGCGAGATCCCAACCCGCGTTCCTCCGGATAGTCCGTTAGCTCCGCAATGGTACAAGATAGAGAATAGAAACGGCGGTAGAGGTAATGGAGAGCTTATGGTTTCGGTGTGGTTCGGGACTCAAGCTGATGAGGCTTTTGCGGAGGCGTGGCATTCAAAGGCTGGAAATGTTCACATCGAGGAACTCTCATCGATCAAATCAAAGGTTTACTTGTCTCCTAAGCTATGGTATCTTAGGATATCAGTGATTGAGGCTCAAGATGTTGCAATTATGGACAAGGGATCAAGTCTCATGAGGTTTCCAGAGCTTTCGGCGAAGCTTCAAGTAGGGAGTCAGATACTGAGAACTGCAATCGCATCTGCAATCCCCACAAAGAGCTTTTCAAATCCTTATTGGAATGAGGATTTGATGTTTGTAGTTGCAGAGCCGTTTGAGGATTGCGTTACAGTTGTTGTAGAGGATCGTCTTAATGGTGGTGCAATAGGAGGACAAAACGATGTGGCTGTTGGGAGGGTTCAGATTCCGATTTCCGCTGTTGAAAGACGAACAGGAGATACACTGGTTGGTTCAAGATGGTTTAGCCTAGATAACGGCAACAATAACAACCGGTTTGGTTCTAGGATTCATCTTAGATTGTCATTAGACGGTGGCTACCATGTTTTGGATGAGGCAACAATGTATAACAGTGATGTTAGACCGACTGCGAAAGAGCTTTGGAAACCGCAAGTAGGGCTTTTAGAGATTGGGATTCTTAGTGCAACTGGTTTGATGCCTATGAAGGTTAGGGACGGGAAATGCGGTGGAATCGCGGACTCGTACTGCGTGGCTAAGTATGGGCCTAAATGGGTCAGAACAAGAACTGTTGTGGACAGTCTTTGTCCTAAGTGGAACGAGCAGTACACTTGGGAAGTGTATGACCCGTGCACAGTTGTTACCGTCGGTGTATTCGATAATGCACGTGTTAATGAAAATAACAACTCGCGGGATGTGCGGATTGGAAAGGTTAGGATTAGGTTGTCTACGCTAGAGACAGGACGGGTTTACACGCATTCGTATCCATTGATTGTGTTGCATCCCTCTGGAGTAAAGAAAACAGGTGAGCTTCATTTAGCGGTTAGATTATCTTGCGGTAACGCTGTTAACATGCTTCATATGTATGCGTTACCATTGCTTCCAAAGATGCATTATACTCAGCCGTTAGGCGTCCATATGCTTGAACGTTTGCGGTATCAAACGCTAAACGCGGTTGCGGCCAGGCTGAGTAGAGCTGAGCCACCATTGGGTCGTGAGGTTGTGGAATATATGCTAGATCATGATTTCCATGTTTGGAGTATGAGAAGGAGCAAAGCTAATTTTTTCCGACTGGTTAACGTGATTTCCGGTTTGGTCGCGGTTGCTAAGTTGGTTGAAGTTATGAGAAGTTGGAGTAAACCGGTTTACTCGACGGTTTTTGTTTTGGCTTTCTTGTTTATGGTTCTGTTTCCGGAGCTTCTATTGCCGTGTTTGCTTCTTTACACGGCTGCGGTTGGCGTTTGGCGTTTCAGGCGGCGGTCTAGATATCCACCGCACATGGACGCGCGTATCTCTCACGCAGAAACTGTGTTTCCCGATGAACTTGATGAAGAGTTTGACACATTTCCGACAAGCAGAGGATTTGATGTTGTGAGAATGAGATACGATCGAGTTAGGAGCATCGCCGGGAGGGTTCAGACAGTGGTTGGTGACATGGCAAGTCAGGGAGAGAGGGTACAAGCTTTGCTAAGCTGGCGTGATCCACGTGCTACGTTTCTCTTCTTGATGTTCTGTTTATTGGCGGCAGTTGGATTTTACACTGTGCCGGTTAAGTTGACAGTAGCTATTTCCGGTTTGTATTATCTGAGACCTCCTCGGTTTAGGAGGAAACTTCCGTCGCGAGGGCTTAGCTTCTTTAGACGGTTGCCGTCTAGAGCCGATAGCTTGCTCTAG

>AT1G22610

ATGAATAAACTAGTTGTAGAAATCGTTGACGCTAGCGATTTGATGCCAAAAGACGGACAAGGCTCAGCGAGTCCTTTCGTAGAAGTTGAATTCGACGAACAGCGTCAGAGAACGCAAACGCGGTTTAAAGATCTAAACCCTCAGTGGAACGAGAAGTTGGTTTTTAACGTCGGTGACTTGAAGCGTTTGAATAACAAGACTGTCGACGTCACTGTTTACGATGACCGCCGTGATAATCAGCCTGGGAAGTTTCTCGGCCGTGTTAAAATTGCCGGTGCGGTTGTTCCGTTATCGGAATCTGAATCCGGTGTTCAGAGATATCCTCTTGATAAACGAGGTTTGTTTTCTAACATTAAAGGTGATATCGCTTTGAGAATCTACGCCGCTCCTATCGACGGCGGCGATTTCGTCTCTCCTCCGCCGGATTTTGCTGAGAAAGTGATGAAAGAAGATAAGAGATTCGAATCTCAGGAGTTTCAATTTCAAAATCAGAATCAGAATCAGAATCACTACGAGCAGTTTGAAGATGAGATTAATAACATGGAGACGTTGAAGCCGACGAAGAAGAAAGAGAAAGAGTCGAGAACTTTTCACTCAATCGGTGCTCACGCCGGAGGAGGAGGAGGAGCTCCACCGATGTCACAAGCGAAACAAGCTTATCCTCCACCTCCGAATCAACCGGAGTTTAGGTCAGATTTCATGAGAGCTCCAGGACCACCGACTGGAGCAGTAATGCAGATGCAACCTCCGAGACAGCAGAATCCGGAGTTTCAGCTAATTGAAACTAGTCCTCCTTTAGCAGCTCGTATGAGACAATCTTACTATTACAGAAGCAGTGGAGATAAAACGTCGAGCACATACGATCTCGTTGAGCAGATGCATTACCTCTACGTTAGCGTCGTGAAAGCTCGAGATCTTCCTGTCATGGATGTTTCCGGTAGCTTAGATCCTTACGTTGAAGTGAAGCTAGGGAACTACAAAGGTTTAACGAAGCATTTGGAGAAGAATTCGAATCCGATCTGGAAACAGATCTTTGCCTTCTCTAAAGAGAGATTACAATCGAATCTTCTTGAAGTCACTGTGAAAGATAAAGATCTTTTGACTAAAGACGATTTCGTTGGAAGAGTTCATATTGATCTCACTGAAGTTCCTCTTAGAGTTCCTCCAGATAGTCCTTTGGCTCCACAGTGGTACAGGTTAGAGGATAAGAAAGGTATGAAGACGAACAGAGGAGAGATTATGCTTGCTGTTTGGATGGGAACTCAAGCTGATGAGTCGTTTCCAGATGCTTGGCACTCGGATGCGCACCGAGTGAGCCATTCGAATCTTTCAAATACGCGGTCCAAAGTTTACTTCTCGCCGAAGCTGTATTACTTGAGAATTCACGTAATGGAAGCTCAGGATCTTGTTCCGTCTGATAAAGGTAGAGTACCTGATGCGATTGTGAAGATTCAAGCGGGGAATCAGATGAGAGCGACGAGGACGCCTCAGATGCGGACGATGAATCCGCAGTGGCATGAGGAGCTTATGTTTGTTGTGTCTGAGCCGTTTGAGGATATGGTGATTGTTTCTGTTGATGATAGGATTGGTCCAGGGAAAGATGAAATATTGGGGAGGGTGTTTATACCTGTGAGGGATGTTCCGGTCAGGCAAGAGGTTGGGAAAATGCCTGATCCACGGTGGTTTAATCTACAAAGACATTCAATGTCTATGGAGGAAGAGAATGAGAAAAGAAAAGAGAAGTTCTCTAGTAAGATTCTGCTTCGGGTTTGTATAGAAGCAGGGTACCATGTCCTTGACGAGTCCACTCATTTCAGCAGCGATCTTCAACCTTCTTCAAAGCATTTGAGGAAGCCGAGTATTGGGATTCTTGAGCTTGGGATTCTCAGTGCTAGGAACTTGATGCCTATGAAAGGTAAAGATGGGAGAATGACTGATCCCTATTGTGTGGCGAAATATGGTAACAAATGGGTCAGAACCAGGACTCTTTTAGACGCACTTGCGCCTAAGTGGAACGAGCAATATACTTGGGAAGTTCATGATCCTTGCACGGTCATAACAATTGGAGTCTTTGACAATAGTCATGTCAACGATGGTGGTGACTTTAAGGACCAGAGGATTGGGAAAGTTAGAGTCAGGCTATCTACGCTGGAGACAGACCGGGTTTACACTCATTTCTACCCGTTGCTGGTTCTTACACCTGGTGGTTTAAAGAAGAATGGTGAACTTCAGCTAGCTCTGAGGTACACCTGCACCGGCTTTGTTAACATGATGGCACAGTATGGAAGACCGTTGTTGCCCAAAATGCATTATATTCAACCAATACCAGTCAGGCATATTGACTTGCTTAGGCATCAGGCGATGCAAATAGTTGCAACAAGACTATCAAGGTCCGAGCCACCTCTGAGACGAGAGGTTGTGGAGTACATGCTTGATGTAGACTACCATATGTTCAGTCTCAGGAGAAGCAAAGCTAATTTCAGCAGAATCATGTCGCTTCTCTCCTCGGTCACTCTGGTCTGCAAGTGGTTCAACGATATCTGCACATGGAGAAACCCGATCACAACGTGTCTGGTTCACGTTCTGTTCCTGATTCTTGTCTGCTACCCAGAACTGATTTTACCCACAGTCTTCCTCTACCTCTTTGTGATAGGCATGTGGAATTACCGGTACAGACCAAGACACCCACCTCACATGGACGCTCGAGTGTCCCAAGCAGACAATGCACACCCAGACGAGCTAGACGAAGAGTTCGACACTTTCCCGACCAGCAGACCGGCAGACATTGTCAGAATGAGGTATGATAGGCTTAGGAGCGTCGGTGGTAGAGTTCAGACAGTCGTAGGCGATCTGGCGACTCAAGGGGAGAGAATTCAAGCTCTTCTTAGCTGGAGAGACCCGAGAGCAACTGCTTTATTCATCGTCTTCGCATTGATCTGGGCCGTGTTTATCTATGTCACACCGTTCCAGGTGATTGCAATCATAATCGGTCTGTTCATGCTGCGTCATCCACGGTTCAGGAGCAGAATGCCTTCAGTACCTGCCAATTTCTTCAAGAGATTACCAGCCAAGTCAGATATGCTACTGTAA

>AT1G51570

ATGCAGAGACCACCTCCTGAAGATTTCTCCTTAAAGGAGACAAAACCTCATCTCGGTGGAGGCAAAGTCACCGGAGATAAGCTCACAACCACATATGACCTCGTCGAGCAAATGCAGTATCTCTATGTTCGTGTCGTAAAGGCTAAAGAATTACCAGGTAAAGACTTAACTGGTAGTTGTGACCCTTACGTTGAAGTTAAGCTTGGTAACTACAGAGGAACCACTAGACATTTCGAGAAGAAATCTAATCCTGAGTGGAACCAAGTTTTCGCCTTTTCTAAAGATAGGGTACAAGCTTCTTATCTTGAAGCTACTGTCAAGGATAAAGATTTAGTTAAAGATGATTTGATTGGTCGTGTTGTGTTTGATTTGAATGAGATACCTAAGAGAGTTCCTCCTGATAGTCCTTTAGCTCCACAATGGTATAGATTAGAAGATGGGAAAGGCCAAAAAGTTAAAGGAGAGCTTATGTTAGCTGTTTGGTTTGGTACTCAAGCTGATGAAGCTTTTCCTGAAGCTTGGCACTCTGATGCTGCAACTGTTAGTGGAACTGATGCTTTAGCTAACATCAGATCTAAGGTTTATCTCTCTCCTAAGCTTTGGTATCTTAGGGTTAATGTCATTGAAGCTCAGGATTTGATACCGAGCGATAAAGGGAGATATCCTGAAGTCTTTGTGAAAGTGATAATGGGAAATCAGGCATTGAGGACTAGAGTGTCACAAAGCAGGAGTATTAATCCGATGTGGAATGAGGATTTGATGTTTGTTGTAGCTGAGCCGTTTGAAGAGCCTTTGATTCTAAGTGTGGAAGATAGAGTTGCGCCGAATAAAGATGAAGTCTTGGGGAGATGCGCGGTTCCGTTGCAGTATTTGGACAAAAGGTTTGACTATAGACCGGTGAACAGCAGGTGGTTTAACCTTGAGAAGCATGTTATAATGGAAGGAGGAGAGAAGAAAGAGATCAAATTCGCCAGCAAGATCCACATGAGGATTTGTTTGGAAGGAGGGTATCATGTGCTTGATGAGTCTACACATTACAGTAGTGACCTAAGACCAACTGCAAAGCAACTGTGGAAGCCTAACATCGGTGTGCTTGAGCTAGGGGTCTTAAATGCGACCGGTTTAATGCCCATGAAAGCTAAAGAAGGTGGTAGGGGAACCACGGACGCGTATTGTGTGGCAAAGTATGGGCAGAAATGGATCAGAACTCGGACAATTATTGACAGCTTTACCCCAAGATGGAACGAGCAATATACTTGGGAGGTTTTTGATCCGTGTACTGTAGTTACAGTTGGTGTGTTCGATAACTGCCATCTACACGGTGGTGACAAAAACAACGGAGGAGGAAAAGATTCAAGAATTGGAAAGGTGAGGATAAGGCTCTCTACTCTCGAGGCTGATCGAGTCTACACACATTCATATCCTCTTTTGGTGCTACATCCTAGTGGAGTCAAGAAAATGGGAGAGATTCACTTAGCGGTGAGGTTTACTTGCTCTTCATTGCTCAACATGATGTATATGTATTCTATGCCTCTCTTACCTAAGATGCACTACCTCCATCCCCTCACGGTTAGCCAGCTCGATAACTTAAGGCATCAAGCAACTCAGATTGTGTCGACAAGGCTAACACGAGCAGAGCCGCCTTTGAGGAAAGAAGTTGTTGAGTATATGCTTGATGTGGGATCTCACATGTGGAGTATGCGTAGAAGCAAGGCGAATTTCTTCAGGATAATGGGAGTCTTGAGTGGGATAATCGCTGTAGGGAAATGGTTTGAACAGATCTGCGTATGGAAAAATCCGATAACAACGGTTTTAATCCACATTCTCTTCATTATCCTTGTTATCTACCCCGAGCTCATCTTGCCCACCATTTTCCTCTACCTCTTCTTGATTGGAGTTTGGTACTATCGCTGGAGGCCGAGACATCCTCCACACATGGACACACGTCTCTCACACGCTGACTCAGCACACCCTGATGAGCTCGACGAGGAGTTTGACACTTTCCCTACTTCCCGACCATCAGACATCGTCAGGATGAGATATGACCGACTCAGAAGCATTGCTGGTAGGATTCAAACAGTTGTTGGTGATCTTGCAACTCAAGGAGAGAGATTTCAGTCGTTGCTAAGCTGGCGTGACCCGCGTGCCACTGCTCTCTTCGTCTTGTTCTGTCTGATAGCTGCAGTGATTTTATATATAACTCCCTTCCAGGTTGTGGCTTTTGCCATTGGCTTGTATGTGCTTAGACACCCGAGGCTCAGGTACAAGCTCCCATCAGTTCCACTTAACTTCTTCAGGAGGCTTCCAGCAAGAACTGATTGCATGCTCTGA

>AT1G74720

ATGAACACGACGCCGTTTCACTCGGATCCTCCGCCGTCGAGGATCCAGCGTAAGCTCGTTGTCGAAGTTGTTGAAGCTCGTAATATTCTCCCTAAAGATGGTCAAGGAAGCTCTAGCGCTTACGTCGTTGTCGATTTCGATGCTCAGAAGAAACGAACCTCCACTAAGTTCCGTGACCTAAACCCTATTTGGAACGAGATGCTTGATTTCGCCGTCTCCGATCCCAAAAACATGGATTACGACGAGCTCGATATCGAGGTTTATAACGATAAAAGATTTGGTAACGGAGGTGGCCGGAAGAATCATTTTCTCGGTAGGGTTAAGATCTATGGAAGCCAGTTCTCGCGAAGAGGTGAAGAAGGTCTTGTGTATTTCCCTTTGGAGAAGAAGAGTGTGTTCAGCTGGATTCGCGGCGAGATTGGACTCAAAATCTACTATTACGACGAAGCCGCCGACGAAGACACGGCGGGTGGAGGTGGAGGACAGCAACAACAACAGCAACAGCAACAATTTCATCCGCCGCAACAAGAAGCCGATGAACAACAACACCAGCAACAATTTCATCCTCCGCCGCAGCAGATGATGAATATACCACCGGAGAAACCTAATGTAGTTGTGGTTGAAGAAGGTAGGGTTTTCGAATCGGCTCAGAGTCAGCGCTATACAGAGACACATCAGCAACCTCCGGTGGTTATTGTTGAAGAATCACCACCGCAGCATGTAATGCAAGGTCCAAATGATAACCATCCTCACCGAAATGATAACCATCCTCAACGGCCACCGTCTCCGCCGCCACCTCCATCGGCTGGGGAAGTACATTATTATCCACCGGAAGTGAGGAAGATGCAAGTAGGAAGACCTCCCGGCGGAGATAGAATTAGGGTTACGAAGAGACCACCGAATGGAGATTATTCACCTAGGGTTATCAATAGCAAAACTGGAGGAGGAGAGACGACGATGGAGAAGAAGACTCATCATCCTTACAATCTTGTTGAGCCAATGCAGTATCTCTTCGTTCGGATTGTGAAGGCGCGTGGCTTACCACCTAACGAGAGCGCGTATGTTAAGGTACGGACGTCGAACCATTTCGTCAGGTCTAAACCGGCCGTTAACCGGCCCGGCGAATCGGTTGATTCACCGGAGTGGAATCAGGTTTTTGCTCTTGGTCATAACCGGTCTGATTCCGCTGTAACTGGTGCGACTCTTGAGATCTCTGCTTGGGATGCTTCGTCGGAGAGTTTTCTCGGAGGAGTTTGTTTTGATCTCTCTGAGGTTCCGGTTCGTGACCCGCCGGATAGTCCGCTTGCTCCTCAGTGGTATCGGCTCGAAGGCTCCGGCGCGGATCAGAACTCTGGGAGAATTTCCGGTGACATTCAGCTCTCTGTTTGGATTGGTACTCAGGTAGATGAGGCATTTCCGGAGGCTTGGAGCTCTGATGCTCCGCATGTAGCTCACACGCGTTCTAAGGTGTATCAATCGCCGAAACTTTGGTACTTGAGAGTGACGGTTCTTGAGGCACAGGATTTACACATAGCTCCTAATCTCCCGCCGTTGACTGCGCCTGAGATTCGTGTGAAAGCTCAATTAGGGTTTCAGTCGGCGCGTACAAGAAGAGGCTCAATGAATAACCACAGTGGTTCGTTTCATTGGCATGAGGATATGATCTTTGTTGCTGGAGAGCCGTTGGAAGATTGCTTGGTTCTGATGGTGGAAGACCGGACGACTAAAGAAGCAACACTTCTAGGACATGCCATGATCCCAGTGAGCTCCATCGAGCAGCGAATTGATGAGCGTTTTGTGCCGTCGAAATGGCACACTCTGGAAGGAGAAGGTGGAGGTGGAGGTGGAGGAGGAGGACCTGGAGGTGGTGGTGGTGGTGGACCTTATTGTGGAAGGATTAGCCTTAGACTTTGTCTCGAAGGTGGGTATCATGTGCTTGAAGAGGCGGCGCATGTATGCAGCGATTTCCGTCCGACGGCTAAGCAGCTATGGAAACCGCCGATTGGAATACTTGAGTTGGGGATTCTTGGAGCTCGTGGGTTGTTGCCGATGAAGGCGAAAAACGGAGGGAAAGGTTCCACTGATGCTTATTGTGTTGCTAAGTACGGGAAGAAATGGGTCAGGACTCGAACCATAACAGACAGTTTTGACCCGAGGTGGCACGAGCAGTATACGTGGCAGGTTTATGATCCTTGCACCGTGCTAACTGTTGGAGTCTTCGACAATTGGAGGATGTTCTCTGACGCCTCCGATGATAGACCTGACACACGGATTGGGAAGATACGGATCCGGGTGTCGACGTTAGAGAGCAACAAAGTGTACACCAATTCATATCCTCTGTTGGTTTTGTTACCTAGCGGTATGAAAAAAATGGGTGAAATTGAAGTGGCAGTCCGGTTTGCATGCCCGTCTCTGCTGCCTGATGTTTGTGCAGCTTATGGACAGCCGCTTCTGCCTCGGATGCACTACATAAGGCCTCTAGGTGTAGCACAACAAGATGCATTAAGAGGGGCCGCCACGAAAATGGTAGCAGCTTGGCTGGCTCGAGCAGAACCACCATTGGGACCAGAGGTAGTTCGATATATGTTAGATGCAGATTCGCATGCATGGAGCATGAGGAAAAGCAAAGCGAATTGGTACAGAATTGTTGGTGTTTTAGCTTGGGCAGTGGGTTTAGCTAAGTGGTTGGATAATATCAGGCGGTGGAGGAATCCAGTGACGACGGTGCTAGTCCATATTCTATATCTGGTTCTTGTTTGGTACCCTGATTTGGTAGTCCCGACTGCATTCTTGTACGTGGTGATGATCGGAGTTTGGTACTACCGGTTTAGACCCAAGATACCGGCTGGTATGGATATCCGCTTATCACAAGCTGAAACCGTCGATCCTGATGAGCTAGATGAAGAATTCGACACCATACCAAGCTCAAGGCGACCAGAAGTAATCCGAGCTAGGTACGACCGATTAAGGATCTTAGCAGTGAGGGTTCAGACCATTCTAGGAGATTTTGCAGCGCAAGGAGAACGGATTCAAGCGTTGGTTAGCTGGAGAGATCCGAGAGCGACAAAGCTGTTCATAGCAATCTGTTTGGTAATCACAATAGTTCTGTATGCAGTTCCTGCGAAAATGGTGGCGGTGGCTCTAGGGTTTTATTATCTTCGGCATCCGATGTTCCGAGACACAATGCCTACGGCTAGTCTCAATTTTTTCCGGCGGTTGCCAAGCTTGTCCGATCGACTCATCTAA

>AT3G03680

ATGGCAGACAATGTTCTGAGGAAGCTGATTGTTGAGATCTGTAGCGCAAGAAATCTGATGCCTAAGGATGGTCAAGGCACCGCGAGCGCTTACGCAATCGTGGATTTCGACGGCCAACGGCGACGGACGAAGACGAAATTCCGAGATCTGAATCCACAGTGGGACGAGAAGCTCGAGTTCTTTGTGCACGACGTGGCGACTATGGGGGAAGAGATTCTTGAGATCAATCTTTGCAACGATAAGAAGACGGGAAAACGGAGCACGTTTCTCGGGAAAGTTAAGATCGCCGGAAGTGCGTTTGCGTCGGCTGGATCTGAAACTCTTGTTTATTATCCGCTTGAGAAGAGGAGCGTTTTCTCTCAGATCAAAGGTGAGATTGGATTAAAGGCTTATTACGTCGATGAGAATCCCCCGGCGGCGCCTGCTGCGACGGAGCCGAAACCTGAGGCTGCAGCTGCGACGGAAGAGAAGCCGCCGGAGATTGCAAAAGCAGAGGATGGGAAGAAAGAAACAGAGGCGGCGAAAACAGAGGAGAAGAAGGAAGGAGATAAAAAAGAAGAGGAGAAACCGAAGGAAGAAGCTAAACCGGACGAGAAAAAACCCGATGCACCACCGGATACGAAGGCAAAGAAGCCTGATACCGCCGTAGCTCCTCCTCCTCCTCCGGCTGAGGTTAAGAATCCGCCTATACCGCAGAAGGCAGAGACTGTGAAGCAGAATGAATTAGGAATTAAACCGGAGAACGTAAACCGGCAAGATCTAATCGGGTCTGATCTCGAGCTTCCTTCACTAACCAGAGATCAGAATCGTGGTGGTGGTTACGATCTCGTCGATCGAATGCCGTTTCTATACATTCGTGTAGCTAAAGCGAAGCGAGCGAAGAACGATGGAAGCAATCCGGTTTACGCGAAGCTCGTGATCGGAACAAATGGCGTGAAAACCAGAAGCCAAACCGGTAAAGATTGGGATCAAGTGTTTGCTTTTGAGAAAGAGAGCTTAAACTCGACCTCGCTTGAAGTTTCCGTTTGGAGTGAAGAGAAGATTGAGAAGGAAGATAAAACAACGACGACTACTGAGAGTTGTTTAGGAACGGTGTCGTTTGATCTTCAAGAGGTTCCTAAGAGAGTGCCTCCGGATAGTCCTTTGGCTCCTCAATGGTACACATTGGAATCTGAGAAATCACCGGGGAATGACGTCATGCTCGCTGTTTGGCTCGGGACTCAAGCTGATGAAGCGTTTCAAGAAGCTTGGCAATCTGATTCCGGTGGGTTGATTCCGGAGACGAGATCCAAAGTTTACTTATCTCCGAAGCTTTGGTATTTGCGTCTAACGGTCATACAAACCCAAGATTTGCAGCTAGGTTTGGGATCCGAAGCTAAGTCAAAGATCCCCACCACTGAGCTTTATGTGAAAGCTCAGCTTGGACCTCAAGTTTTCAAAACGGCTAGGACTTCGATTGGGCCGTCGGCTTCTTCGTCCGGATCCGGTAACCCGACTTGGAACGAGGATCTAGTTTTCGTAGCTTCCGAGCCGTTTGAGCCGTTCTTGATAGTGACTGTGGAAGATATAACCAACGGGCAATCAATTGGTCAGACTAAGATACATATGGGAAGTGTTGAGAGGAGAAATGATGACCGGACTGAACCGAAATCGAGGTGGTTTAATCTCGCCGGCGATGAGAAGAAACCGTACTCCGGGAGAATCCACGTGAAAGTTTGTCTTGAAGGTGGGTATCACGTGCTAGACGAAGCAGCTCACGTGACGAGTGATGTCAGACCCTCTGCTAAACAGTTAGCCAAGCCACCTATTGGTTTATTGGAAGTGGGTATCCGTGGAGCTACTAATTTACTTCCTGTGAAAACCAGAGACGGTACACGTGGCACCACCGATGCTTATGTGGTTGCTAAATATGGACCGAAATGGATTCGGACTCGTACGATCCTAGACAGGTTTAATCCGCGGTGGAACGAGCAGTACACATGGGATGTTTATGATCCGTGCACGGTCCTTACTATTGGAGTTTTCGACAATGGAAGATACAAACGGGACGAATCCGGAAAGCAAGGGAGAGATGTTAGGGTCGGGAAAATCCGTGTACGTCTCTCGACACTCGACATGAACCGCATATACCTTAATTCCTACACCTTAACCGTGATCTTACCAAGCGGAGCCAAGAAAATGGGAGAAGTTGAAATCGCGGTTCGATTCTCTTGTCCATCTTGGCTAAGCATTATTCAAGCCTATGTCACACCAATGCTACCTAGGATGCACTATGTCCGTCCGCTTGGTCCAGCTCAACAAGACATTTTGCGTCACACGGCTATGCGTATCGTGACAGCCCGGCTTGCGCGGTCTGAGCCTCCATTAGGTCAAGAGGTGGTTCAGTACATGCTAGACACAGACAATCATGTGTGGAGCATGAGGAGAAGCAAAGCTAATTGGTTCCGTGTCATCACGTTCTTGTCACGGGCCGCAACTATAGCCCGTTGGATTCACGGGATTCGAACTTGGGTTCACCCACCAACAACCGTTCTCGTTCACTTGCTTCTCGTGGCCATCGTCTTGTGTCCACATTTAGTCCTCCCAACCGTATTCATGTACGCGTTCTTGATCTTAGCCTTACGATTCCGTTACCGTGGCAGAGTCAAAGTCAACAGCGTTGACCCACGACTATCTTGCGTTGACTCCGTTGCTCCCGACGAGCTTGATGAAGAGTTTGATGGTTTCCCAACAACGAGACAACCGGAGGTCGTACGTATAAGGTATGACCGGTTAAGGGCATTGGCCGGTCGGGCACAGACTTTATTAGGTGACGTGGCGGCTCAAGGGGAGCGTGTTGAGGCGTTGTTCAACTGGAGAGACCCACGCGCCACGTGTATATTCGTAGTGTTCTGTCTTTTCGCGTCGTTTTTGTTCTACATCGTTCCGTTTAAAGTTTTCCTTTTGGGATCTGGTTTTTACTACATTCGACATCCGAGGTTTAGAGACGATATGCCTTCCGTTCCGGTCAACTTTTTCCGGCGACTTCCTTCAATGTCGGACCAGATTCTGTGA

>AT3G57880

ATGCAGAGACCACCTCCTGAAGATTTCTCCTTGAAGGAGACGAGACCTCATCTAGGTGGAGGAAAACTCTCTGGAGATAAGCTTACCAGTACTTATGACCTTGTTGAGCAAATGCAGTATCTCTATGTTCGTGTGGTTAAGGCAAAGGAGTTACCTGGCAAGGATATGACTGGTAGTTGTGACCCTTATGTTGAGGTTAAGCTTGGAAACTACAAAGGCACCACTAGGCATTTCGAGAAGAAATCTAATCCTGAGTGGAACCAAGTTTTCGCCTTTTCTAAAGATAGGATTCAAGCTTCTTTCCTTGAAGCTACTGTCAAGGACAAGGATTTTGTCAAAGATGATTTGATTGGTCGGGTTGTCTTTGATTTGAATGAGGTACCTAAGAGAGTTCCTCCTGATAGTCCCTTGGCTCCGCAATGGTATAGGCTCGAGGATAGGAAAGGTGATAAAGTCAAGGGAGAGCTTATGTTGGCTGTTTGGTTTGGTACTCAAGCTGATGAAGCTTTCCCTGAAGCTTGGCACTCTGATGCTGCAACTGTTAGTGGTACTGATGCTCTTGCCAACATCCGTTCCAAGGTTTATCTTTCTCCTAAGCTTTGGTACCTCAGGGTTAATGTAATTGAAGCTCAAGATTTGATACCAACTGATAAGCAAAGATATCCCGAGGTTTATGTGAAGGCTATAGTGGGAAACCAGGCGTTGAGGACTAGAGTATCGCAGAGCAGGACTATTAATCCCATGTGGAATGAGGATTTGATGTTTGTCGCAGCAGAGCCATTTGAGGAACCTTTGATCCTCAGCGTGGAAGATAGAGTTGCTCCAAACAAAGATGAAGTCTTGGGGAGATGTGCGATCCCGTTGCAGTATTTGGACAGAAGATTTGACCATAAACCGGTGAACAGCAGGTGGTACAATCTCGAGAAGCATATTATGGTCGATGGAGAGAAGAAGGAGACCAAATTCGCTAGCAGGATTCACATGAGAATATGTTTGGAAGGAGGGTATCACGTTCTCGATGAGTCTACGCATTACAGCAGTGATCTTAGACCAACAGCGAAGCAACTATGGAAGCCTAATATCGGTGTACTGGAGTTGGGGATACTGAATGCTACAGGTCTGATGCCTATGAAAACCAAAGACGGCCGGGGAACCACAGATGCATATTGTGTGGCGAAGTACGGACAGAAATGGATTCGAACTCGGACAATCATTGACAGCTTTACACCAAGATGGAATGAGCAATATACTTGGGAGGTTTTTGATCCGTGCACCGTTGTTACTGTTGGAGTTTTTGATAACTGCCATCTCCATGGAGGTGAGAAGATTGGAGGAGCAAAAGATTCAAGAATCGGGAAGGTAAGAATCAGGCTTTCTACTCTCGAGACTGATCGAGTTTATACACATTCATACCCTCTTTTGGTGCTCCATCCTAACGGAGTCAAGAAAATGGGAGAAATTCACTTAGCTGTGAGATTCACTTGCTCTTCATTGCTCAACATGATGTATATGTACTCACAGCCTCTCTTACCGAAGATGCATTATATCCATCCACTGACGGTTAGCCAGCTTGATAACTTGAGGCATCAAGCGACTCAGATTGTATCGATGAGGCTGACCCGAGCAGAACCACCTCTAAGGAAAGAAGTAGTTGAGTATATGCTTGATGTGGGTTCTCACATGTGGAGTATGCGGAGAAGCAAAGCAAACTTTTTCAGGATTATGGGAGTTTTGAGTGGGTTAATCGCAGTAGGAAAATGGTTTGAACAAATCTGCAACTGGAAGAATCCAATCACAACAGTCTTGATCCATCTCTTGTTCATCATCCTTGTGCTCTATCCCGAACTAATCTTGCCCACCATCTTCCTCTACCTCTTCCTCATCGGTATCTGGTACTACCGTTGGAGACCGAGGCATCCTCCTCACATGGACACACGTCTCTCTCACGCTGACTCAGCCCACCCCGACGAGCTAGATGAAGAGTTCGACACTTTTCCTACTTCCCGACCATCTGACATAGTCAGGATGAGGTATGACAGGTTGAGGAGTATTGCTGGTAGGATTCAGACAGTGGTAGGTGATCTAGCAACTCAAGGAGAAAGGCTTCAGTCTCTGCTAAGCTGGCGTGATCCGCGTGCAACAGCGCTCTTCGTCTTGTTCTGCTTGATTGCCGCAGTCATTCTCTATGTGACACCTTTCCAGGTTGTGGCTCTTTGTATTGGAATCTATGCTCTGAGACATCCGAGGTTCAGATACAAACTACCATCCGTTCCTCTCAATTTCTTCAGAAGGCTTCCTGCAAGAACTGATTGCATGCTCTGA

>AT3G61300

ATGATGAGTAATCTGAAACTTGGCGTTGAAGTCATAAGCGCAAGGCTCAAACCTAGAGAGGACTATGGCGGTGTTAACGCTTACGTCGAGCTACGATTTGACGATCAGAAAGTCATAACTATGACTAAGATCGATGACTCTAGTCCGGTGTGGAACGAGAAGTTCTTCTTCAACATCTCTGATACAGAGGATTTATCAAATCAATTTCTTGACGCTTACGTTTACAATAAAACCAGCAGCATAACAAAATCTTGTCTAGGTAAGATTCGAATATTGGGAACTGCGTTTCTCCCGTACTCAGAAGCTGTTGGATTGCCTTATCCTCTAGAGAAAGAGAAATGGAGCATGTTCTCTTCCGCTGCCGCCAATGGAGGTGAGCTCGCTCTCAAAGTTTTCCTCACTGATAACCCCTCTCCCAAGGTACCGAATCTAATCTCTACAAAGAAGATTCCTTCTAAATCAAGGCACAAATTTCACAACATCCCCACAAATGAGAGTAACCATAGCCCACGAGGGAATCAGCAATCGTTTCAACCGCAACCGCCTCCGCCTCAATCGCAAACGGCTCTACCGCCACCGATGATGGAGTCTTCTCTCTATCAAGCACCGAGGTTTGGTACTCCGATACCTACAACTATGGGGTTTAATCCGAATCCACCGGATTATTCTATCAAAGAGACGAAACCGATTCTCGGAGGAGGCAAACGAGCAAGATCAAGTGATCATGATCTCGTCGAGCCAATGGAGTTTCTCTTCATCAAAATCGTGAAAGCTAGAAATCTTCCATCCATGGACTTAACCGGAAGTCTAGATCCATACATCGAAGTCAAACTCGGAAACTATACCGGCAAAACCAAACATTTTGAGAAGAATCAAAACCCGGTTTGGAACGAAGTCTTCGCTTTCTCCAAATCCAATCAACAATCAAACGTTCTTGAAGTCATCGTCATGGACAAAGACATGGTCAAAGACGATTTCGTCGGTTTAATCCGGTTCGATCTCAATCAGATTCCGACACGTGTCGCACCAGACAGTCCTTTAGCTCCAGAATGGTACAGAGTCAACAACGAGAAAGGAGGCGAGATTATGTTGGCTGTTTGGTTCGGTACTCAAGCCGACGAAGCGTTCTCAGACGCGACATACTCAGACGCTTTAAACGCTGTTAACAAATCGAGTTTGCGTTCTAAAGTCTATCATTCTCCAAGGCTTTGGTACCTCCGGGTTAATGTCATCGAGGCACAAGACTTAGTTATTGTACCAGACCGGACTCGATTGCCAAATCCATATGTAAAAATCCGGTTGAATAACCAAGTGGTTCGAACCAAACCGAGTCACTCGCTGAATCCAAGATGGAACGAAGAGTTCACTCTCGTCGCGGCTGAACCGTTTGAAGATCTGATCATCTCGATTGAAGACCGAGTCGCACCTAACCGAGAGGAAACGCTGGGAGAGGTACATATACCGATTGGTACAATTGATAAACGGATTGACGATAACCGGACAGTGCCTAACCGTTGGTTTAGTCTTAAAACTGAGAACCAGAGAAGAGTCAGATTCGCGACGACGAGACTTCATCTAAACGTTTGTCTAGAAGGAGGCTACCACGTTCTTGATGAATCTACTTACTACAGCAGCGATTTCAGACCGTCGATGAAAGAGTTGTTGAGTCATAAACAACCGTCTTTTGGTGTTCTTGAATTAGGAATCTTGAGAATTGAAGGTTTGAATTTGAGCCAAGAAGGGAAGAAGGAGACGGTGGATGCTTATTGTGTAGCTAAGTATGGAACCAAATGGGTCAGGACTCGAACCGTTACGAACTGTTTAAACCCGCGGTTCAATGAGCAGTACACATGGGAGGTTTATGAGCCAGCAACTGTAATAACAATCGGTGTTTTCGATAACAATCAGATCAATAGTGGTAATGGTAACAAAGGAGATGGGAAAATTGGGAAAATCAGAGTTAGAATCTCGACTCTCGAAGCCGGAAGGATCTACAGTCACTCGTATCCGCTTCTTGTTCTTCGACCTTCAGGTTTAAAGAAAATGGGGGAGTTGCATTTAGCGATTAGGTTTTCCTGCTCATCGATGTTTCAGATGCTAATGCAGTACTGGAAACCGCTTCTTCCCAAGATGCATTACGCAAGACCGTTGAAAGTAGTGCAGCAGGAGATTCTTAGGCAGCATGCGGTTAACTTAGTGGCAGCTCGGTTAAGTAGAGCCGAGCCGCCACTTAGAAAAGAAGTAGTTGAATACATTTCGGATTCAAATTCGCATTTATGGAGCATGAGGAAGAGCCGTGCAAATCTCTTCAGATTGAGTTCTGTTTTCTCTGGTTTACTAGGAACCGGAGAATGGTTTCAAGATATCTGCAGGTGGAAGAAACCGGTAGAAACTACAGCTATTCATATAATCTTCTTGGTGTTAGTGTGTTCGCCGGAGATGATTCTTCCAGTGATGTCTCTTTGCTTGTTCATGCTTGGGGTATGGAACTACAGGCTCCGTCCAAGACAACCTCCACACATGGACACAAGACTATCATTTGCAGACAATATCCACCCGGAAGAGCTTAACGAAGAGTTCGATACCTTCCCTTTTTCGTCTCAAGACCCGGGGATTGTAAAAATGCGGTACGAGCGGTTAAGGAGTATCGCTAGTAGAGCTCAAACGGTTGTGGGAGATATAGCAGGTCAAGGAGAACGAGTTCAGGCTCTGTTAAGCTGGAGAGATCCAAGAGCAACTTCGATCTTCATGGTGCTTTGTTTGGTTTCTACTGTCGTTTTGTATGTTGTGCCGTTTAAGGTTTTTGTTCTTCTCGCCGGACTATACATCATGAGACCTCCAAGATTCAGGGGTAAGACACCTCCTGGACCTATTAATTTCTTCCGACGACTTCCTGCTAAAACAGATTGTATGCTGTAG

>AT3G61720

ATGGCTGCCAATAAAGATGAATTCTCCGTCAAGCAAATCTTTCCAAAGCTGGGAGGAGAAAGAGGAGCTCGAAACCCTCGTTACGGTCCAACTTCTTCACACGACCTCGTCGAACAGATGGAGTTTCTTTACGTACAAGTAATCCAAGCCATCAACAACTCTGTTGTAAACCCTAGTGCCCGCATATGTTGCCCCGTCGTCGAAATCACTCTCGGGAACTACAAATCCTCTACCAAAAATCTGCCAATGGGACCAAATATGGATTGGAACCAAGTCTTTGCTTTCGACAAATCCAAAGGTGATGTCTTGTCGGTTACTCTTAAAGACGGTCCGACCAATACCGTAATCAACAAGCGCAACTTCAAGCTGGCCTCGGAAATTCCGACTAGGGTTCCTCCCGATGCAAGAATCGCACCGCAGTGGTACTCTATGCATAATACCGAAACCGACTTTTACATGGAGTTGCTGATGTCAGTTTGGTTCGGTACACAAGTTGATGAGGTTTATCCAGAGGCTTGGTTTTCAGACGCCTGTGAAGTGTGTGCCTCTCGCGTGATCAACACGCGACCTAAGGTCTACCTAGCCCCAAGGCTCTGTTATGTTAGGGTTACGATTGTTTCAGGTCATGATTTGATTTCCAAGGATAAAAACAAGACTCCTTCGGTTTATGTGACAGCTACTCTAGGTAAAGTCGCTCTAAAAACGAAAGTTTCTTCCGGTACAAACCCGTCATGGAACCAAGATCTAATCTTTGTTGCATCAGAGCCGTTAGAAGGAACTGTCTACATTAGGTTAATCGACAGAGAAGACGAACAACACGAAGGATGCATAGGGACGTTGAAGAAGAAGCTGACAGAGATGACTCCGCTCAAAGTTCCAAGTTCCGCACCGGCTTTGTTCTACGACATTGAAATGCCGACTGAAGTCAAACCGGCTGGCGATTCGAGGAGGTTCGCTAGCAGACTCAAGATGAAACTAGCCACTGATCAAGCTTATCACGTCGCCGAGGAATGCACCCAATACTCGAGCGACAATAGAGCTTTTGTAAAAGGGTTATGGCCCGGTCTGCTCGGGAAATTAGAAATCGGGATCTTGGGAGCAACCGGTTTAAAGGGAAGCGACGAGAAAAAACAGACTATCGACAGTTACGTAGTGGCTAAATACGGGAATAAATGGGCCAGGACTCGAACCGTGGTTAATAGTGTCTCACCCAAGTGGAACGAGCAGTATTCGTGGGATGTTTACGAGAAGTGTACGGTGTTAACCCTTGGAATCTACGATAATCGACAAATCTTGGAGGACAAGAATAAGGCGAACGATGTTCCGATCGGGAAAGTGAGGATTCCATTGAACCGGGTACAAAGTGACTGGATTTACACTTGCTCGTATCCTATTCTGAAGTTGGGAAGCAGCGGACTGAAGAAGATGGGAGAGCTTCAGCTAGCAGTTCGTTTTGTTTATGTTGCGCAAGGGTATGCACGGTATTCAGCTCCTTTCCGTTGGATGTTACCGAAAGCTCATTACAAATCTCCATTGTCCATGTATCAGATCGACAAGTTGAGAGCACAAGCTGTTGAGATTAATTGTGCGAATCTTGCGAGAACAGAGCCGGCTTTGAGAAGCGAAGTTGTGTCAGATATGTTGAAACCAAAGAGTAGGAACTTCAGTATAAGAATATCCAAAGATAATTTTGATAGGCTTTATACAGTCGTTAAGATGGTCTTGTGGTGTGTTTCGGTCATTGCATCGGTCAGATCCACGACAGCCTGTACTCCGAAATTCATTGCGCTTGGAGTCAGTTTTGTGTTTCTTTTTTGGGAGTACTACATATATTGGTTGGTGACTTCGTGGCTAGTTGCGTATTGCATTGTGCTTTGCATTGTGGTTATACTTTTACGGGAAATCCTCAAGTCCCCGAGGCAAACATATAACTGGTTGTTTTATCGGAACGTCACTCCGCCTCCTTTGATTCTCGTCGATTTGAAGCTACGGAAACTTGACTCGATCAACCTAGACGAGTTAGCCGAAGAGTTTGACTCGTTTCCGTCGTCGGAGAACGATTTGAACATTCTTAGGATGAGGTACGATAGGTTGAGGAAGATTATGGAGAACGTCATGTTATTAATGGGGGATGCTGCTACACAAGGCGAACGGTTACTAGCGGCTTTTACGTTGCTTGAGCGGCCATTCGTTTTGATTATCTTATTGGCCCTCTGTTATTGCTCCATGCTCGTCGTTTGCCTTGGTTGGGATCTACATGTTCGTAAGTGTTTGATTTTCGTATTTATTTGCTACTGGGTGCAACTACCGTGGTTCCGCAATAATTTACCCGATGGGAGCCTCAACTTCTTCCGAAGATTGCCGAGCAACGAAGACCTCATGTTCTGA

>AT4G00700

ATGAGCAATATAAAGCTAGGAGTTGAAGTGATAAGCGCACAGGGTCTGTTACAGAGAGATAAGCATAACTCATGTAGCCCTTTCGTGGAGCTCAAGTTCGACAACCAGATATTCCGCGCAACGACCAAACACAACGACCCAAATCCTGTCTGGCACGAGTGTTTTTACTTCGTTGTCTCTGACCCTTCCGTCTTGTCAACTCGTACGCTTGAAGCCCACGTTTACAGCTACCAGAACGAATTCGATGCTAAGCCCTTTCTTGGCAAAGTTCGAGTCAACGGAACATCTTTTGTTCCTCGCTCTGAAGCAGCTCCTTTCAATTACCCTTTAGAGAAACGCAGTGTTTTCTCTCGAGCCCGTGGAGAACTTTGCTTGAGAGTTTTTATCACAGACGACCCTTCTGTTACACCTTCTGTCCCAACCCCGGTTCCAGAATCTCCTCAAGCTTATAGCCCAAGTCCGCGTAAAGAACATGTGAAGTCCTTAATCACAGCCGATGCAAGTATGGCTACTGATGAGAGAAGAGAGCTAAAGCCTAAAACACGTACGTTTCATAACTCTGCCCCTTTAGTGAAACAACAGCCAATGATGAACTATGGCATCCACGAGATGAGAGCTGCACCGATGCCTCCAAGAGTGGTTCAAGTCAACGGTCCAGGTCCATCCTTGCACCAGCTGCCACCAGATTTCTCTGTCAAAGAAACAAGCCCTTTGCTCGGAGGTGGACGGATCGTTGGAGGACGAGTGGTTCGTGGTACAGAGAGGCCAACAAGCGGGACATATGATCTCGTTGAAGAGATGAAATTTCTCTACGTGAGAGTTGTGAAAGCGCGTGATCTCCCCAACAAAGACCTTACAGGAAGCCTAGACCCGTATGTCGTAGTAAAAATCGGAAACTTCAAAGGTGTTACCACACATTTTAACAAGAATACAGATCCCGAGTGGAACCAAGTCTTTGCCTTCGCTAAAGATAATCTTCAGTCGAACTTTCTTGAAGTTATGGTCAAGGATAAAGACATTCTCCTTGATGATTTTGTTGGGATCGTTAAATTCGATCTCCGTGAGGTTCAGTCTCGCGTTCCTCCTGATAGTCCTTTAGCTCCCCAGTGGTACAGACTGGAGAACAAGAGAGGCGAGAAGAAGAATTATGAGATAATGCTTGCTGTGTGGTCTGGAACTCAGGCTGATGAAGCTTTCGGGGATGCGACTTTCTCAGACTCATTGGTAGATTCAGACAGCTCCAACATTATATCCGCTAACCTGCGCTCAAAAGTGTACCATTCGCCTCGGCTATGGTACCTAAGGGTGCAAATATTGGAAGCTCAGGACGTCATCATCGTGTCTGACAAATCCCGTGTTCCAGAGGTCTTTGTGCGAGTAAAAGTCGGTAATCAGATGTTAAGGACTAAATTTCCCCAGAGAAGCAATAATCCAAAATGGGGTGATGAGTTCACGTTTGTAGTCGCGGAGCCGTTTGAGGATAACTTGGTCCTCTCCGTGGAAGATCACACTGCTCCTAACAGAGACGAGCCAGTAGGAAAAGCTGTGATTTTGATGAATGACATAGAGAAACGTATAGATGATAAACCTTTTCATGACCGATGGGTTCATCTTGAAGACTCCATCTCAGACGCTATGGATGTTGATAAAGCCAAGAAAGTGAAGTTCGCTACTAGGCTCCGGTATAAGGCGGTTCTCGATGGAGGCTACCACGTTTTTGATGAGTCCATGTATAACAGCAGTGATCTCAGACCTTCGTCGAGAAAGCTCTGGAAACCAGCAATTGGTGTCTTGGAGCTCGGGATACTTAACGCAAATGTGTTCCATTCGATGAAAACTCGGGAAGGGAAAGGTACTTCGGACACGTACGTTGTTGCCAAATACGGTCACAAATGGGTTCGGTCACGAACTGTGATCAACAGCATGAATCCAAAGTACAATGAGCAATACACATGGGAGGTTTTTGATCCTGCAACGGTTTTAACCATCTGCGTCTTTGACAACGCTCATTTCGCAGCAGGAGATGGCGGAAACAAGAGAGATCAGCCGATTGGGAAAGTCCGCATTAGACTCTCGACTCTTCAGACGGGCCGTGTGTACACGCATGCTTACCCTTTACTTGTTCTACAACCTACTGGTCTTAAGAAAAGAGGAGAGCTTCACTTAGCGGTGAGATTCACCTGCACATCGGTCTCCAGTATGCTGATGAAATACACAAAACCTCTCCTGCCTAAAATGCATTACATACTACCCTTATCCACAAACCAACAGGAAGCTCTTAAGATGCAGGCTATCAACATAATTATTGTTCGTTTAGGCCGGTCCGAGCCACCGCTGAGACGGGAAGTGGTTGACTATTTGACAGATTGGAAAAGTCAGCTGTTCAGCATGAGGAGAAGCAAAGCCAACTTCAATAGGTTCACGACAGTGTTCTCCGGTGCGCTGTCTGTTTGGAAATGGATGGAACAAGTGTGCACATGGAAGACTCCCGTGACGACGGCTCTGGTGCACGTGCTCTACACTATGCTTGTGACGTTCCCGGAGATGATTTTGCCGACTGTGTTTCTGTATATGGCTGTGATTGGGATGTGGAATTACAGGTTTAAGCCTAGATTTCCTCCTCATATGGACGCCAAACTATCTTATGCAGACAATGTAAATTCTGATGAGCTTGACGAAGAGTTTGATACCTTCCCGACCGTAAGGGCTCCGGACATTGTGAAAATGAGGTACGACCGGCTGAGATCTGTGGCCGGGAAGGTACAGTCGGTGGCTGGAGACATAGCCGCACAAGGAGAGCGAGTGCAAGCGTTGCTGAGCTGGCGTGATCCACGCGCCACCGCAATATTCGTGACGTTCTGTTTCATTATTGCCATGGCTCTCTACATTACCCCTTTTAAACTTGTCGCTCTGTTATCCGGTTACTACTTCATGCGACATCCAAAGCTCCGACACCGCATTCCCTCCGCTCCCGTCAACTTTTTCCGGCGACTTCCAGCCATGACCGACTCTATGCTGTGA

>AT4G11610

ATGATGATGAGCAATCTAAAACTCGGCGTGGACGTGATCGGAGCACACAATCTCTTCCCAAAAGATGGGCAAGGAACATCGAACGCTTACGTTGAGCTTTACTTCGATGGTCAAAAGCACCGAACAACGATCAAAGATCGAGACTTAAACCCGGTTTGGAACGAGAGTTTCTTCTTCAACATCTCAGATCCATCTCGTCTTCACTATCTCAATCTTGAAGCTCAAGCTTATAGTCACAACAGATCTACTAATGGACGTTCCTTCCTTGGCAAGGTATCTCTCTCAGGGACCTCTTTTGTTCCTCATTCTGATGCTGTTGTTCTTCACTTTCCCATGGAGAGACGTGGGATCTTTTCTCGTGTGAGAGGTGAGCTCGGCTTGAAAGTTTACATAACCGACGAGGCTTCTTTAAAATCCTCTGCTGCTTCTAATGATCATCCAGATAATCTAGATCCGGCGCTCCCAAGAGCAATGAATGTAGAACATAGATCCGACAAACGACATGTGTTTTACAATCTCCCAAACAGTGCTCAAGAACATCAACACCAACATCCACAAGGGCCTAACCAATCCTCTTCCTTAGCCGCTGAACAAGATAATCATAACGAACATCATCACCATTACGTACCAAAGCATCAAGTAGACGAGATGAGATCCGAACCAGCGCGGCCCTCGAAGCTAGTCCATGCGCATTCCATTGCTTCGGCTCAGCCTGCTGATTTCGCGCTTAAGGAGACAAGCCCGCATCTCGGTGGTGGAAGAGTCGTTGGTGGGCGTGTTATACACAAAGACAAAACCGCTACGAGTACTTATGATCTTGTCGAGAGAATGTATTTCCTTTATGTCCGCGTTGTCAAAGCTCGTGAGCTTCCTATAATGGATATAACAGGAAGTGTTGATCCATTCGTCGAGGTGAGAGTGGGGAACTACAAAGGAATCACAAGACATTTTGAGAAGAGACAACATCCGGAATGGAACCAAGTGTTTGCATTCGCGAAAGAGCGAATGCAAGCCTCGGTGTTGGAAGTAGTGGTTAAGGACAAAGATCTTTTAAAAGACGATTATGTCGGGTTTGTTAGGTTTGACATTAATGATGTCCCTCTTCGTGTGCCACCAGACAGCCCTCTTGCTCCGCAATGGTATAGATTAGAGGACAAGAAAGGAGAGAAGATCAAAGGTGAGCTTATGCTTGCTGTTTGGATCGGTACACAAGCTGATGAAGCTTTCTCTGATGCTTGGCATTCTGATGCTGCAATGCCCGTTGATTGTTCTCCAGCTATCTCCGCCGTTCTCCGCTCAAAGGTCTATCATGCGCCTCGTCTATGGTACGTCCGTGTGAACGTGATCGAAGCACAAGACTTGATTCCGACAGACAAAACTAGGTTCCCTGATGTTTACGTCAAGGCGCAGCTAGGAAACCAAGTCATGAAGACCAGACCATGCCAAGCTCGAACCCTTGGTGCCGTATGGAACGAAGACTTTTTGTTTGTTGTTGCGGAGCCATTTGAAGACCATTTAGTTCTCACCGTCGAGGACCGGGTTGCTCCAGGAAAGGACGAGATAGTTGGTCGTACTTACATTCCTTTGAACACGGTAGAGAAGCGAGCTGATGATCATATGATACATGCGCGATGGTATAATCTGGAGAGACCGGTTATTGTAGATGTGGATCAGCTCAAGAGAGAAAAATTCTCTATGAGGATTCATCTGCGAGTTTGTCTTGAAGGAGGATACCATGTTTTGGATGAGTCAACTCATTACAGCAGCGATCTCCGCCCATCCGCTAGACCACTCTGGAGACAACCGATCGGAGTTCTTGAGCTCGGGATCTTAAACGCTGTTGGACTTCACCCAATGAAAACTAGAGAAGGCAGAGGAACCTCTGACACTTTTTGCGTTGGGAAATATGGACAAAAGTGGGTTAGAACAAGGACAATGGTGGATAACTTGTGTCCCAAGTATAACGAGCAGTATACATGGGAAGTCTTTGACCCAGCGACGGTTTTAACCGTTGGTGTGTTTGATAACGGACAGCTAGGTGAAAAAGGAAATAGAGATGTGAAGATTGGGAAGATTCGTATAAGGTTATCTACTTTAGAGACTGGACGGATCTACACACATTCTTACCCGCTACTTGTTCTTCACCCTACTGGGGTGAAGAAAATGGGCGAGCTACATATGGCCGTGAGGTTCACATGCATCTCGTTCGCAAACATGCTTTACCAATACTCAAAACCACTTTTGCCTAAAATGCATTATGTTAGACCGTTTTCAGTAATGCAGCAGGACATGCTTCGACATCAAGCGGTGAATATAGTGGCTGCGCGGTTAGGTAGAGCAGAGCCTCCCCTGAGGAAAGAGATCATTGAGTTTATGTCGGACACGGATTCGCATTTGTGGAGTATGCGTAAGAGCAAAGCTAATTTCTTCCGGATGATGACAGTTTTCTCCGGTGTTATCGCGGTTGGAAAATGGTTCTCGGACATTTGCTCGTGGCGAAACCCGATCACGACCGTTCTTGTTCATGTTCTGTTTTTGATGCTTGTTTGTCTCCCAGAACTGATCTTACCAACAATGTTTCTCTACATGTTTTTGATCGGGCTCTGGAACTACCGGTTTAGACCACGTTATCCACCACACATGAACACAAAAATCTCTCAAGCCGAGGCGGTCCATCCAGACGAGCTTGATGAAGAGTTTGATACGTTTCCAACCACTAGAAACCCTGACATGGTGCGTTTAAGGTATGACCGGCTTAGAAGCGTGGCCGGGAGGATTCAGACCGTGATCGGAGATCTTGCAACACAAGGAGAACGTTTTCAAGCACTTTTAAGCTGGAGAGATCCACGCGCTACCGCGATATTTGTTATATTGTGCTTTATTGCGGCCATAGTTTTCTTCATAACACCGATTCAGATTGTTGTAGCATTGGCTGGATTCTTCACGATGAGGCATCCGAGGTTCCGACATCGGCTTCCTTCTGTTCCAGTTAACTTCTTTCGCCGGTTGCCTGCTCGAACGGATAGTATGCTCTAG

>AT4G20080

ATGGCGGTGAACGGTACCGGTAACGGAACCGGTGACGGCGATTTCTCATTGAAAGAGACATCTCCCAATATCGGCAATGGTGGTGTTAACGGCGGCGAGAAGCTGACGTCATCGTTCGATCTTGTGGAGGCGATGCATTTCCTCTACGCTAGAATTGTCCGTGCTCGAGCCTTACCGGTCAACGACTCTTTCGTCGCAGTCAAAATCGGAAGCTACAAAGGAAGAACAAAACAGATCTTAAACTCAAACCCTAACCCTGAGTTTCACGAAACCTTCGCATTCACGAAAACTCGTCTTCAAGGGGATATCTTAGAAGTGGTCGTGCGAAACAGAGATAACCCAAACGAAGACGATATTGTCGGGAAATGTAAGTTCGATGTTGCGGAGATTCCGACACGTGTTCCTCCTGATAGTCCATTGGCTCCTCAATGGTATAGATTGGAAGATAGAAACGGTGTCAAGATCGGTGGAGAGATTATGGTATCTGTTTGGATTGGGACACAAGCTGATGAAGTTTTCTCGGAGGCTTGGCATTCGGATTCGGCGAGTGTTACCGGAGAAAACGTGGTGAACACGCGATCTAAAGTTTATCTTTCACCGAGGCTTTGGTACTTGCGAGTTAATGTAATCGAAGCTCAGGATTTGGTTCTGTTGCACCCGAACCGGATCAATCCAGAGATTCTGATTAAAGGGTTTCTTGGAAACGTTGTTGTGAGGAGTCGGATTTCGCAGACGAAGAGTGTGAGTCCTGTGTGGAACGAGGATATGATGTTTGTAGCGGTTGAGCCGTTTGATGACAGTTTGATACTTAGCGTGGAAGATAAGGTAGGGCCGAGAGAAGAGTGTTTAGGAAGGTGTGAGATAAAGTTATCTCAGGTTGAGAGAAGGGTGCTTCCTGGTCCGGTGCCGTCATTGTGGTACAACGTTGAACACATTGGTGAGACCGGAGAAGGGAGGAGATTTGCTGGGAGGATTCATTTGCGGGTTTCTTTAGACGGAGGTTATCACGTTCTTGATGAGTCGATTCAGTATAGCAGCGATTACAGAGCTTCAGCTAAGCTTCTATGGACTCCACCTATTGGTGTGTTGGAGCTAGGAGTTTTAAATGCTACGGGTTTAATGCCGATGAAGTCAAGAGGTGGCCGAGGAACTACCGATGCGTATTGTGTGGCGAAATACGGAACAAAATGGGTGAGAACGAGGACCATTGTGGACACTTTCGATCCAAAGTGGAATGAGCAGTATACTTGGGAAGTCTATGATCCTTACACGGTGATAACAATCGGTGTCTTCGATAATCTGAAATTGTTTGGTGCGGGTAACGAAAACCGTTTGATCAACGATTCAAGGATTGGGAAGATCAGGATACGTTTATCGACCCTTGTAACCTCGAAGATCTACACACATTCTTATCCTTTAATGGTTTTAAAACCAGACGGGGTTAAAAAAATGGGTGAGATTCAGCTCGCGGTTAGGTTCACCGCGACATCTATGATGGATATGCTTCAGAAGTACACAGAGCCGTTGCTTCCAGAAATGCATTACATATCTCCATTGTCTATATACCAATTAGACAGTCTTAGGCACCAAGCGACCCACATTCTCTGCATCAACTTAGGGCGCAACGAGCCAGCACTTGGAAGAGATGTTGTAGAGTATATGCTCGATGTGGGATCAAACATTTGGAGTCTACGAAGAGGAAGAGCGAATTTCGAACGTCTTGTCTCGTTTTTCGATGGCTGGATCGATGCTTGGAAATGGTTTGATGAGATTTGCAAATGGAAAAGTCCCGTGACGTCGGTTTTAGTCCATATTGTCTGCTTGTTCGTTGTTTTTCTCCCTAAGTACTGTGTTTTCTCGATGCTACTGTACTGCTTCGTGTTTGGGTTATACAGGTTCGGTTTGAGACCGAGACATCCTCCACATATGGATATAAAGCTGTCTAAAGCTGATTCAGCTTTGCCCGATGAGCTAGACGAGGAGTTTGATGTGTTTCCAAGCTCGAAATCCGGTGATGTACTGAAAAGAAGGTATGATAGGTTGCGTGGGATCGCAGGGAGGATGATGATTGTGTTGGGAGATTTGGCTACACAAGGAGAAAGAGTGAAAAGTTTGTTGAGTTGGAGAGATCCAAGAGCAACGTCTCTGTTCTTGACGTTCTGTTTTGTTAGCTGTGGAGTGATTTGTTTCGTCTCCATGAAGTTACTGCTCACATTTCTTGCGTTCTACGTCATGAGACATCCTAGGGTTAGAGTTTTCGATATACCTTCTATTCCTCAGAATTTCTTCAGGAGATTACCATCAAGAGCTGATAGCATCTTGTGA

>AT5G03435

ATGGCTGCCAATAAAGATGAATTCTCCGTCAAGCAAATCTCTCCAAAGCTGGGAGGAGAAAGAGGAGCTCGAAACCCTTACGGCCCAACTTCTTTACACGACCTCGTCGAACAGATGGAGTTTCTTTACGTAGATGTAATCCGAGCCATCAAGAACTCTGATGTAGACCCTGGACCATGTGACCCCGTCGTCGAAATCACTCTCGGAAACTACAAATCCTCTACCAAAGATCTGCCAGTTGGACCAAATATGGATTGGAACCAAGTCTTTGCTTTCGACAAAACCAAAGGTGATGTCTTGTCGGTTACTCTTAAAGACCGTCTGACCAATACCGTAATAAACAAGTCCAACTTCAAGCTGGCCTCGGAAATTCCGACTAGGGCTCCTCCCGATGCAAGAATCGCGCCTCAACGGTACCCTTTGCGTAATACCAAAACCGGTTTTTACTTGATGATGTCAGTTTGGTTCGGTACACAAGTTGATGAGGTTTATCCAGTGGCTTGGTTCTCAGACGCTTCTGAAGTGAGCACTTGCGTGATCAACACGCGACCTAAGGTCTACCTAGCTCCAAGGCTCTGTTATGTTAGGGTTACCATTGTTTCTGGTCACGATTTGATTTCCACGGATAGAAACCGGACTCCTTCGGTTTATGTGACAGCTACTCTAGGTCAAGTCACTCTAAAAACGGAAGTTTCTTCCGGGACGAACCCGTCATGGAACAAGGATCTAATCTTTGTTGCATCAGAGCCGTTAGAAGGAACTGTCTACATTAGGTTAATAGACAGAGTCGACGATCAACACGAAGAACGCATAATAGGGAAGTTAGAGAAGAAGCTCTCAGAGATGACTCCGCTCAAAGTTCCAAGTTCCGCACCGGCTTTGTTCTACGACATTGAGGTCGAACCAGCAGGTGATTCTAGGAGGTTCGCCAGCAGACTCAAGATGAAACTAGCCACTGATCAAGCTTATCACGTCGCTGAGGAAAGCATTCAATACTCGAGCGACTATAGACCTTTTGTTAAAGGGTTGTGGCCATGTCTGCTCGGGAAATTAGAAATTGGGATTTTGGGTGCGACGGGTTTAAAGGGAAGCGACGAGAGAAAACAGGGCATTGACAGTTATGTAGTGGCTAAATACGGGAATAAATGGGCCAGGACTCGAACCGTGGTTAATAGTGTCACACCCAAGTGGAACGAGCAGTATTCGTGGGATGATTACGAGAAGTGTACGGTATTAACCCTTGGAATCTACGACAATCGACAAATCTTTAAAGAGGATCAGGCGAACGATGTTCCGATCGGGAAAGTGAGGATTTCATTGAACCGTGTAGAAAGTGACTGGATTTATGCTTGCTCGTATCCAATTCTGAAGTTGGGAAGCAGCGGACTGAAGAAAATGGGAGAGCTTCAGCTCGCGGTTCGCTTTGTTTATGTTGCGCAAGGGTATGCACGGTATTCAGCTCCTTTCCGTTGGTTGTTACCGAAAGCTCATTACAAATCTCCATTGTCGGTGTATCAGATCGAAGAGATGAGAGCAGAAGCTGTTAAGATTAACTGTGCGAATCTTGCGAGAACAGAGCCGGCGTTGAGAAACGAGGTCGTGTGGGATATGTTGAAGCCGAAGACGAATACCAGATACTCAACATGTGATATGCGGAAAGTTGCTGCGCTTGCTTTCTTTGATCTATTTCTTTATTGGCCCTCTTTGATAGTTTGGTTGGCGATTTACTTAGTTGTTGTGCCTTGCATAGTACTTGTAGGTCTTTCGGGATTACACAAGTTCCTGACACGAAAGTTTTGGAACAAGAGAGAAAATCCTCGTTCTCCATTGATTGTCAACGACTTGAAGCTCTGGAAACTCGAATCGCCAAACCTAGATGAGTTAGAGGAAGAGTTTGACTCGTTCCCGTCGTCGGTGAGCGATGTGAACATTCTGCGGATGAGGTACGATAGAATAAGGATGGTTTGTCAGAGACCGATGATATTACTGGGCGATGCTGCGTCACAAGGTGAAAGGCTTTACGCTCTCTTAACGTTTAATGGCGATGATCAATTGGCTTCGTTTTACTGCTGGCTCATATGTGTTTTGGTCGCGCTCTGCTGGTACAATATACCGATGTGGCTTTGGTCTCTCTACCCTATCGCATATTGGCTTAATTTCACGCCCTTACGCAACGATATGCCCTGTGGAGTCAGCAACTTCTTCCGAAGATTGCCCACAAACGAAGTATTGTTCTGA

>AT5G06850

ATGGCAGCCAAAGATGGAGCTAAGAGCCAAGAAGACTATAAGCTTAAAGACATGAAACCAGAGCTTGGAGAGAGATGGCCACATGGAGGACAGCGTGGAGGAACCGGATGGATTGGCAGTGAACGAGCTGCAAGCACATATGACCTTGTTGAGCAAATGTTTTATCTCTATGTTCGGGTCGTCAAAGCCAAGGATCTTCCTCCAAACCCTGTCACCAGCAACTGTGATCCTTATGTGGAAGTGAAAATAGGAAACTACAAAGGAAAGACTAAACACTTCGAGAAAAGAACCAACCCGGAGTGGAATCAAGTTTTCGCATTTTCAAAAGACAAGGTCCAATCCTCTACTGTTGAAGTCTTTGTGCGCGATAAGGAGATGGTTACAAGAGATGAATACATCGGGAAGGTTGTGTTTGATATGCGTGAGGTTCCTACAAGAGTTCCTCCTGATAGTCCACTTGCACCTCAGTGGTACAGATTAGAAGATCGGCGAGGTGAGAGTAAGAAAAGAGGAGAGGTTATGGTAGCGGTTTGGCTTGGGACGCAAGCCGATGAAGCATTTCCTGATGCTTGGCATTCGGATGCTTCCTCTGTTCAAGGAGAAGGTGTCCAAAGCGTGAGGTCTAAAGTCTATGTATCTCCCAAATTGTGGTATCTGCGGGTTAATGTCATCGAGGCGCAAGATGTTGAGCCTAGTGACAGGAGCCAACCACCTCAAGCTTTCGTTAAAGTACAAGTTGGGAATCAGATTCTGAAAACAAAGTTATGTCCTAACAAGACGACGAATCCGATGTGGAACGAAGATCTTGTTTTTGTAGCTGCAGAGCCATTTGAAGAACAGTTCTTCTTGACGGTCGAAAACAAGGTGACACCAGCTAAAGATGAAGTCATGGGGAGGCTGATCTCACCTTTGAGCGTATTTGAGAAAAGGTTAGATCACAGAGCTGTTCATTCCAAATGGTACAACCTCGAAAAGTTTGGGTTTGGGGCTCTGGAAGGAGACAAGAGACACGAGCTCAAGTTCTCCAGCCGAATCCACCTACGAGTTTGCCTTGAAGGAGGTTACCATGTAATGGACGAGTCAACTCTGTACATCAGTGACGTGAAACCTACGGCAAGGCAACTATGGAAGTCTCCTATTGGCATCCTTGAAGTAGGAATCTTGAGTGCTCAAGGGCTATCGCCTATGAAAACAAAAGACGGTAAAGCGACAACAGATCCGTATTGTGTTGCCAAGTACGGTCAAAAATGGGTGAGAACAAGAACCATCATTGACAGCTCTAGCCCTAAATGGAATGAGCAATACACATGGGAAGTGTATGATCCTTGTACAGTTATCACACTGGGAGTTTTCGATAACTGTCACCTTGGCGGAAGTGAGAAATCGAACAGTGGAGCCAAAGTGGATTCGAGAATCGGGAAGGTAAGGATCCGGTTGTCGACTCTAGAGGCTGATAGAATCTACACTCACTCCTACCCTCTTCTTGTTCTGCAAACAAAAGGGTTAAAGAAAATGGGAGAAGTCCAATTAGCTGTGAGATTCACTTGTCTATCCTTGGCTCACATGATCTATCTTTATGGCCATCCTCTGCTTCCAAAGATGCATTATCTCCATCCATTCACTGTGAACCAATTAGACAGTCTAAGATACCAAGCAATGAGCATTGTCGCAGCACGGTTATCTCGAGCAGAGCCTCCTTTAAGGAAAGAGAACGTAGAGTACATGCTTGATGTTGATTCACACATGTGGAGCATGAGAAGGAGCAAAGCTAACTTCTTCAGGATAGTGTCTGTGTTTGCAGGCTTGATTGCTATGAGTAAATGGCTCGGAGACGTCTGCTACTGGAAGAATCCGTTGACGACGATATTATTCCACGTTCTCTTCTTTATATTGATTTGTTATCCGGAGCTGATACTCCCAACCACATTCCTCTACATGTTCTTGATAGGGCTTTGGAATTTCCGGTTCAGGCCGCGTCATCCTGCTCATATGGACACAAAGGTATCTTGGGCTGAAGCGGCTAGCCCTGATGAGCTAGATGAGGAATTTGATACTTTCCCAACTTCAAAGGGACAAGATGTTGTGAAGATGAGGTATGATAGGTTAAGAAGCGTTGCGGGTCGGATCCAGATGGTTGTTGGAGACATTGCAACGCAAGGAGAGAGGTTTCAGGCTTTGTTGAGTTGGAGAGATCCGCGTGCGACTTGCTTGTTTGTGATTTTTTGTCTTGTTGCTGCGATGATTTTGTACGTGACGCCTTTCAAGATCATAGCTCTTGCGGGTGGGATGTTCTGGATGAGGCATCCTAAGTTCAGAAGCAAGATGCCTTCTGCTCCAAGTAACTTCTTCAGGAAATTACCTTCAAAAGCAGATTGTATGCTTTGA

>AT5G12970

ATGCAGAAACCAGGGCAAAATATAGATTTTGCACTGAAGGAAACTTCACCAAAGATTGGTGCAGGTTCTGTGACAGGTGATAAACTCTGCAGCACTTATGATTTGGTTGAGCAGATGCATTACCTCTACGTACGGGTCGTTAAGGCGAAGGAGTTACCGGGGAAAGATGTGACCGGTAGTTGTGATCCATATGTGGAAGTGAAGCTCGGAAACTACAGAGGGATGACTAAGCATTTCGAGAAGAGGTCGAATCCGGAGTGGAAGCAAGTGTTTGCGTTTTCAAAGGAGAGGATCCAAGCATCAATCTTGGAGGTTGTCGTTAAAGACAAAGATGTGGTGTTAGATGATTTGATTGGTAGAATCATGTTTGATCTCAATGAGATTCCTAAACGGGTACCGCCTGATAGTCCATTGGCTCCTCAGTGGTATAGGTTGGAGGATCGACATGGGCGTAAGGTTAAAGGAGAGCTTATGTTAGCTGTTTGGATGGGGACACAAGCAGATGAAGCTTTTTCTGATGCTTGGCATTCAGATGCAGCCACGGTCGGACCTGAGGGTGTGACACATATCCGTTCAAAGGTTTATCTTTCGCCAAAGCTTTGGTATGTCAGGGTCAATGTAATTGAGGCTCAAGATTTGATACCTCATGATAAAACCAAGTTCCCTGAGGTTTACGTGAAAGCAATGCTCGGAAACCAGACTCTGAGAACACGTATTTCTCAGACCAAAACATTGAATCCGATGTGGAATGAAGATCTGATGTTTGTTGTTGCTGAGCCTTTTGAGGAAGCTTTGATTCTCGCGGTGGAAGATAGGGTTGCACCGAATAAAGATGAAACGTTAGGCCGGTGTGCAATCCCGTTGCAGAACGTCCAAAGAAGGTTAGACCATAGGCCGCTTAACTCGAGGTGGTTCAACCTGGAGAAACATATTATGGTGGAAGGAGAGCAAAAAGAGATCAAATTCGCAAGCAGAATTCATCTAAGAATCTTTCTTGAAGGTGGGTACCATGTTCTTGATGAATCAACTCACTACAGCAGCGACCTAAGGCCAACCGCAAAACAGCTATGGAAACCGAGTATCGGACTGCTTGAAGTAGGGATCATAAGTGCACACGGGCTGATGCCAATGAAGTCAAAAGACGGGAAGGGGACAACAGATGCATATTGCGTGGCTAAGTATGGGCAGAAATGGATTAGAACAAGAACAATCGTGGATAGTTTTACGCCTAAATGGAATGAGCAGTACACATGGGAAGTGTTTGACACCTGCACTGTCATAACTTTTGGAGCATTCGACAACGGACACATTCCAGGCGGAAGCGGAAAAGACTTGAGAATCGGGAAAGTGAGAATCCGGCTCTCTACCCTTGAAGCTGACCGTATCTACACTCATTCATATCCGCTACTCGTCTTTCATCCTTCTGGGATCAAGAAGACAGGTGAAATACAGTTAGCTGTGCGGTTCACTTGCCTATCTCTCATCAACATGCTTCATATGTATTCTCAGCCATTACTACCCAAAATGCATTACATCCATCCGTTATCGGTTCTCCAGCTGGACAGCCTGAGACACCAGGCGATGAACATTGTCTCGGCAAGGCTGAACCGCGCAGAGCCGCCTCTTCGCAAAGAGATTGTGGAGTACATGCTCGATGTTGACTCCCATATGTGGAGCATGAGGAGGAGTAAAGCTAACTTCTTCAGAATCATGAATGTTCTGAGTGGTCTCATTGCTGTTGGAAAATGGTTTGATCAGATCTGCAACTGGAGAAACCCAATAACCACAATTCTCATTCATGTTCTTTTCATTATCTTAGTTCTTTACCCGGAATTAATCCTCCCAACGGTTTTCTTGTACCTTTTCTTGATTGGGATCTGGAATTTCCGGTGGAGACCAAGGCACCCGCCACACATGGACACACGGTTGTCCCATGCAGACGCTGTTCACCCCGACGAGCTTGATGAAGAGTTTGATACTTTCCCGACTTCCCGATCCTCTGAGATTGTGCGGATGCGATATGACCGGCTCAGAAGCATAGGAGGACGGGTTCAGACCGTGATAGGCGATCTAGCAACACAGGGAGAGCGGTTTTTATCGCTGCTGAGCTGGAGAGACCCGAGGGCAACCACATTGTTTGTGCTTTTCTGTCTCATAGCTGCTATCGTGTTGTATGTTACGCCGTTTCAGGTTGTTGCACTTCTTGCTGGGATCTATGTGCTGAGGCATCCAAGGTTCAGGCACAAGCTACCCTCTGTGCCGCTCAATCTCTTCAGGAGGCTACCTGCAAGATCTGACAGTCTATTATAG

>AT5G17980

ATGGCAACAACCCGGAAACTGGTAGTAGAAGTAGTAGACGCAAAGGATCTTACACCAAAAGACGGTCATGGAACGTCAAGTCCGTACGTTGTCCTCGACTATTACGGCCAAAGAAGACGGACACGAACCATAGTACGTGACTTAAACCCTGTTTGGAACGAGACACTCGAGTTCAGTCTCGCAAAGCGACCATCTCATCAGCTCTTTACCGATGTTCTTGAGCTCGACATGTACCATGACAAGAACTTTGGACAAACCCGGAGGAACAATTTTCTTGGTAGGATCCGACTTGGGTCCGATCAGTTTGTTGGTCAAGGAGAAGAGGCTTTGATATATTATCCTTTGGAGAAGAAGAGTTTGTTTAATCTTGTACAAGGTGAGATTGGGCTTAGGGTTTACTACGCCGATGAAAAGCCTCCTCCTTTGAAACCGACGGTAGCTCCTCTTGAGACGGTGGTGGAGGAAAAGACGGAGGAGACGAAGGCGGAAGGTCCAGACGAGTCTAAGCCACCACCAGAGACTAATGACATTCCTGCTGAAGTGAAAGAAACCGTGAAACCACCACAACCACCACCGGAGGAGAGTTCACCAGCCGAGGGTCCCAAGCCAGACGAGGAAGCCTCACCACCTTTACAAGAAAACGCTACGGTTGGTGGAGAAGAACCACCGGCGTCGGAATCAGATAAGAATGAAGCAGAGGCGAAACCCGTGGAGGAGCCACCGCAGAATCAACCAGATGGTGAAGATATTGTGCTTGAGTCAGAAGATACAATGAGTTGGGCTTCGGCGCCGCGATCACCGTTACCGGAAGTTATCATTTCAAGATCTGTATCCGGTTCCATACCCGAAACCAAAAACGGACCACAACCACTACGGAGAAGCGTATCGGAAACTGCAAGCTACACATCAGAGATCTCCGACGTATCAACGATCGAACGATCCACGTTTGATTTGGTGGAGAAGATGCATTACGTCTTCATCCGCGTCGTGAAAGCTCGGTCTTTACCAACCTCCGGGAGTCCAGTCACAAAGATCTCACTCTCCGGCACAATGATTCAATCAAAACCGGCTAGAAAAACCTCTTGCTTCGAGTGGGATCAAACGTTCGCCTTCCTCCGTGACTCACCGGATTTATCTTCCTCTCCAATTCTGGAGATATCCGTGTGGGATTCATCGACGGGAATCGAAACCAGCCAATTCCTTGGTGGAATTTGCTTCGACGTGTCAGAGATTCCGCTCCGAGACCCGCCGGATAGTCCATTAGCGCCGCAATGGTACCGTCTCGAAGGTGGTGGAGCTCACAACAGTGATCTAATGCTTGCCACGTGGACAGGAACTCAAGCAGACGAATCATTCCCCGACGCGTGGAAAACTGATACAGCCGGAAACGTGACCGCACGAGCGAAGGTTTACATGTCGTCGAAGCTGTGGTATCTACGCGCTACCGTGATAGAAGCACAAGACTTGTTACCGCCTCAGTTAACGGCGTTTAAGGAAGCGTCATTTCAGTTAAAAGCTCAGTTAGGTTCCCAAGTTCAGAAAACCAAATCCGCCGTTACTCGTAACGGAGCTCCGTCATGGAACGAAGATCTACTCTTCGTAGCCGCCGAACCTTTCTCCGATCAGCTTGTTTTCACGTTGGAGTATCGAACATCGAAAGGACCAGTCACCGTCGGAATGGCTCGCGTGCCACTCAGCGCAATCGAGAGACGCGTCGACGATCGCTTGGTTGCTTCGAGATGGTTAGGATTGGAAGATCCAAACGACGAGAAGAGAGGAAACAGATCTAGAGTTCACATTAGGCTTTGCTTCGACGGAGGTTATCACGTGATGGACGAGGCGGCGCACGTGTGCAGTGATTACCGACCTACGGCGAGGCAGTTATGGAAACCAGCGGTGGGAATCGTCGAGCTCGGTATAATCGGTTGCAAAAATCTATTACCGATGAAGACGGTTAACGGAAAAGGATCAACGGACGCGTACACGGTGGCGAAATACGGTTCTAAATGGGTCCGTACCCGAACCGTGTCGGATTCTTTAGATCCGAAATGGAACGAACAGTACACGTGGAAAGTATACGATCCGTGTACGGTTTTGACTATAGGAGTCTTCGATAGCTGGGGAGTATACGAAGTCGACGGTGGAAAAGAAGCCACGCGTCAAGATCTAAGAATTGGAAAAGTACGTATACGTATATCGACGCTGGAGACTGGTAAAGCGTATAGAAATACGTATCCGTTACTGATGTTAGTTAACGGCGGCGTTAAAAAATTGGGAGAGATAGAATTGGCCGTTAGATTCGTGAGAACAGCTCCACCGTTGGATTTCTTACACGTGTATACTCAACCGTTATTGCCTTTGATGCATCACATTAAACCGTTGAGTTTGTTTCAAGAAGATATGTTGAGGAACACTGCAGTGAAGATCTTGGCCGCACATTTGTCACGATCTGAGCCGCCTTTAAGGCCGGAGATAGTACGGTATATGCTCGACGCCGATACTCATACTTTCAGTATGCGTAAAGTCAGAGCAAATTGGCTTCGGATAGTGAATGTAGTCGCCGGAATGGTCGACGTGGTGCGGTGGGTTGATGACACCCGTTTCTGGAAGAATCCGACATCAACTTTACTAGTTCACGCACTTGTCGTGATGCTGATTTGGTTCCCGGATCTTATCGTACCGACATTAGCGTTTTACTTGTTCGTGATTGGTGCGTGGAATTATAGGTTCAGGTCACGTGCTGCTCTACCACATTTCGATCCAAGACTCTCGTTAGCTGATGCAGCTGATAGAGACGAGCTCGACGAGGAGTTTGACGTCGTACCGAGCAACCGACCACCGGAGATGGTTCGGTTGAGGTACGATAAGCTACGAAACGTCGGAGCTAGAGTTCAAACGATTCTTGGTGAAGTGGCTGCGCAAGGGGAGAAGATGCAAGCTTTGGTGACGTGGCGTGACCCACGAGCGACTGGTATATTCGTGGGGCTGTGTTTCTTTGTGGCGTTGGTGTTGTATCTTGTGCCGACGAAGATGGTGGCTATGGCGTCAGGGTTTTATTACTTCCGGCATCCTATTTTCCGTGATCGGAAACCTTCTCCGGTGTTAAATTTCTTCCGGCGACTACCATCATTATCTGATCGGCTCATGTAA

>AT5G48060

ATGAGAAACACCACCAAACTAGTCGTGCACGTGGTTGATGCTCAATATCTTATGCCCAGAGATGGCCAAGGATCAGCAAGTCCTTTTGTAGAAGTTGATTTCCTTAACCAGTTAAGCAAAACAAGAACCGTACCTAAAAGCCTTAACCCTGTTTGGAACCAAAAACTCTACTTTGATTATGACCAGAGTGTGATAAACCAGCACAACCAACACATTGAAGTCTCGGTTTACCACGAAAGAAGACCGATTCCCGGTAGAAGTTTCCTCGGTAGAGTCAAGATTTCTCTCTGTAATATTGTTTACAAGGACGACCAAGTGTATCAACGCTTCACGTTGGAGAAGAAATGGCTTCTCTCTTCTGTTAAAGGTGAGATTGGTCTCAAATTCTACATTTCATCGTCAGAAGAAGACCAAACATTTCCTCTTCCTTCTAAACCTTACACCAGCCCAACACAAGCCTCTGCCTCTGGAACTGAAGAAGATACTGCTGATTCTGAAACAGAAGATTCTCTAAAGAGTTTTGCATCAGCAGAGGAAGAAGATCTTGCTGATTCGGTCAGTGAGTGTGTCGAAGGGAAAAAGAGTGAAGAAGTTAAAGAACCAGTTCAGAAACTACATAGACAAGAAGTTTTTGCTCGGCCAGCACCTATGCAGTCCATTCGCCTACGGTCTCGTGAAAACCCGCATGAAGCCCAAAAACCGATGTCTCGTGGAGCTAACCAGCTTCACCCTCAAAACCCCAACCACTTGCAATCATATGGTGACACTGACCTAGACGACTTTAAGGTGAAAGACATGAACTTGGATCTTGGAGAGAGATGGCCAAATCCAAATGCTGGAGAAAGATTTACAGGCACATACGATCTTGTGGAACAGATGTTTTATCTCTATGTTCGAGTTGTTAAAGCAAAAGAGCTACCACCTGGTTCAATAACTGGCGGATGTGATCCTTACGTGGAGGTCAAGCTTGGGAACTACAAGGGAAGAACGAAAATTTTTGATAGGAAGACAACAATTCCTGAATGGAACCAAGTTTTTGCATTTACCAAAGAACGGATTCAATCATCAGTTCTTGAAGTTTTCGTGAAGGACAAGGAAACACTAGGTCGAGATGATATTCTTGGAAAGGTAGTGTTTGATCTGAACGAGATCCCAACAAGGGTTCCACCAAACAGTCCTTTGGCTCCGCAGTGGTACCGATTAGAGGATTGGAGAGGAGAAGGCAAAGTAGTACGAGGAGAGATAATGCTTGCTGTCTGGATGGGAACTCAAGCCGATGAAGCTTTTCCTGAAGCATGGCACGCCGACTCTGCTTCTGTTCATGGAGAAGGCGTGTTCAACATTAGATCCAAGGTATATGTATCTCCTAAGCTGTGGTACTTAAGAGTCAATGTTATCGAAGCTCAAGACATGATCCCGAGTGATAGAAACCGGCTTCCTGATGTTTTCGTCAAAGCAAGTGTTGGAATGCAAACCCTAAAGACAAGTATCTGTTCTATAAAGACCACAAATCCACTCTGGAAAGAAGATCTTGTCTTTGTGGTTGCTGAGCCATTTGAAGAGCAGCTAGTGATCTCTGTCGAAGACCGCGTTCACACGTCTAAAGATGAAGTAATTGGCAAGATTACCCTGCCGATGAACGTGTTCGAAAAGCGTTTAGACCACCGTCCAGTCCATTCCCGTTGGTTCAATCTTGATAAGTACGGTACCGGAGTTCTTGAGCCAGACGCAAGAAGAAAAGAACATAAGTTCTCGAGCCGGATTCACCTACGAATCTGTCTTGAAGGAGGCTACCATGTGATGGACGAGTCAACAATGTACATTAGCGACACAAGACCTACAGCTAGACAGTTATGGAAGCAGCCGGTTGGAATGTTAGAGATTGGGATTCTTGGAGCAAATGGGTTGGTGCCAATGAAGTTAAAAGACGGTAGAGGAAGCACTAACGCTTATTGTGTTGCTAAGTATGGTCAAAAATGGGTGAGAACCAGAACCATTCTTGACACATTAAGTCCTAGATGGAACGAGCAGTACACATGGGAAGTATATGATCCTTGTACTGTCATTACTCTCGGAGTTTTCGACAATAGTCATTTGGGCTCTGCACAATCCGGAACTGCAGATTCTAGAGATGCAAGAATCGGAAAGGTTCGAATAAGGTTGTCTACACTAGAGGCTCACAAGATCTACACACATTCATTTCCGCTACTTGTTCTTCAACCGCACGGTCTCAAGAAAACGGGGGACCTACAAATATCTGTCCGTTTTACAACTCTCTCTTTAGCCAACATCATCTACAATTACGGCCACCCGCTTCTCCCCAAAATGCACTATCTTTTCCCTTTCACGGTCAACCAAGTAGATGGTTTAAGGTACCAAGCCATGAACATTGTTTCAACCCGCCTTGGCCGAGCTGAGCCACCACTTAGGAAAGAGGTCGTCGAGTACATGCTGGATGTAGATTCTCACTTATGGAGCATGAGAAGAAGCAAAGCCAACTTTTTTCGCATAATGTCGCTTCTTTCAGGTTATTTCTTGGTCGGGAAGTGGCTCGAAGATGTTTGCAACTGGAGATACCCGGTTACTTCGGTTCTGGTCAATGTCTTGTTCTTCATTTTGGTCATGTACCCTGAACTGATTTTGCCTACCATGTTTCTCTACATGTTCTTCATTGGATTGTGGAACTTCAGGTCCAGGCCGAGACACCCTCCTCACATGGATATGAAACTCTCATGGGCCGAGGCTGTAGGTCCTGATGAGCTCGATGAAGAGTTTGACACATTCCCAACGTCTCGGTCACAAGAGTTGGTGCGTCTGAGATATGACCGGCTCAGAAGCGTGGCAGGTAGGATCCAGACTGTTGTTGGAGACATTGCAGCTCAGGGAGAGAGAATCCAGTCACTTTTAAGCTGGCGAGACCCCAGGGCTACAAGCCTCTTCATTTTGTTCTGCTTGGCTGCGTCGGTGGTCCTATACGCAATGCCGTTCAAAGCAATAGCACTAGCCAGTGGGCTATACTATCTAAGGCATCCAAAGTTTAGAAGCAAGTTACCTTCTCTGCCAAGTAACTTCTTCAAGAGATTGCCGTCTAGTACCGATAGTCTTCTTTGA

>Amtri00004.74

ATGGCGGAAGGCAGTCACCGGAAACTTGTGGTGGAAATAAGTCAAGCTAGAAACCTGATGCCGAAAGACGGTCAAGGAACCTCGAGCCCTTATGTTCAGGTGGACTTCGATGGCCAGAGAAGACGCACTAAAACGAAGTTCAGAGAGCTCAACCCTCAATGGGATGAGAAGCTCGAGTTCCTCGTAAACGATCCCGAATCTATGGCCTCTGAAACCCTAGAACTCAACATCTACAACGACAAGAAGACCGGAAAACGAAGCACATTTCTAGGGAAGGTCAAGCTTTCAGGTAATTCTTTCCCTAAAATTGGATCTGAAAGTTTCACCTACTTTCCCTTAGAGAAACGCAGCGTTTTCTCTCAGATCAAAGGTGAGTTAGGGTTAAAGGTCTACTACATAGATGAGGAGCCGCCAGAAAAGGCTCCAGCTCCGTCCAATCCAGAAGAGAAACCGCCAGAAAATGCTCCAGAACCGGCCAAAGAAGAGGAGAAACCACAGGAAAAAGCAAAGACAGAGGAGGCACCCCCAGCCAAAGAAGAGGAAAAGCCACCGGAAAACAAAGATCCCAGCGAGAAGCCGGCGCCAGAGGCCGAAGATTCAAAGAAGGAGGAAGAAAAACCGCATGAAAAGCCACCAGAAAAGTCGCCAGAAAATGCAAGTCAGAAAGTTTTGGCCACCACGAAAACGGTATCCGGCGTGAACACCTTACAGACTCGAATTATAGGAGACGGTGTGAGGGGTGCATACGATCTTGTAGAGCGCATGCCGTATCTATACGTGCGTGTCGTAAAGGCGAAGAGGACTGCAGACAATGGCTCGACACCGTGTCTGTATGCTAAGCTGGGCATAGGTACTCAAAGTGTAAGCACTCGAACCGTGCAAAACACGGAGTGGGATCAGGTATTCGCCTTCCAGAAAGAGAAGCTCAACTCGGCGAGCCTCGAGGTCTCGGTACACGAAGAGAAGAAGCCCGACGCAGCGGCTGACACACCCAAGGACACTCCTCCAGCCACCGACACCTGCCTGGGTACGGTGTCGTTCGATCTGCAAGAGATTCCGAAGAGGGTTCCTCCTGATAGCCCTTTGGCCCCTCAGTGGTACACGCTCGAAGGAGGGCTTCCGGAAGGTGACGTCGCCTTGCCAGGAAATGATGTCATGCTCGCTGTTTGGTATGGGACTCAGGCGGACGAGGCATTCCCGGCTGCTTGGCAATCGGATTCCGGCGGCCTCCTGGTGCATACCCGAGCCAAGGTCTACCTCGCTCCGAAGCTGTGGTACCTCCGCCTAACGGTCATCCAAACCCAGGACCTTCAGTTCCCCAGCACGCCCGAACCAAAGGCCCGGGCCCCTGAGCTTTACGTTCGGGCCCAGCTAGGTCCCCAAGTTCTGAAGACGGTTAAGAGTGTCGGCTTGTCGGCTAACGCGCCGAACCCAAGCTGGAATGAAGACTTGATTTTTATTGCAGCCGAGCCGTTTGAGCCGTTTTTGGTAGTCGGCGTGGAGGACCAACCTAGCGGGCAGCAGGTTGGGAAGGCCAAGATCCACGTGGCGAGCCTTGATAGGAGGATCGACGAGCGGGAAGAGCCCAAGTCGAAATGGTATAACCTTGGCTCAGATGAGAATAAGGCATATGCAGGCCGTATACACGTGCGAGCTTGCTTAGAGGGTGGTTACCACGTGTTAGATGAGCCGCCTCATTTGGTTAGCGACTCGCGGGCCTCGGCTAAGCAGCTTTGGAAGCCGCCGATCGGCTCATTGGAGCTGGGCATTTTGGGGGCGACCAATCTCATGCCGATAAAGACCATGGACGGGACACGTGGCACCACCGATGCGTATTGCGTGGCTAAGTACGGAACCAAGTGGGTACGGACAAGGACCGTTTTGGACAATTTTAGCCCAAGGTGGAATGAACAGTATACGTGGGAGGTGTACGATCCGTCTACGGTTCTTACTCTCGGCGTGTTTGATAATGCACAGTATGGGAAAGATGAAGGAAAGGGAGCTTCTAAGGACACACGTATAGGCAAGGTACGTATCCGGCTTTCTACGCTAGACACAAACAGGGTATATACGAATTCTTATACACTCACAGTGCTTCAACCTGGTGGAACCAAGAAAATGGGTGAGCTCGAGCTTGCAGTGCGCTTCTCTTGCACCTCTTATCCAGCCCTAATGGCTTCTTACTTCACTCCCCCTCTTCCCCGTATGCATTATGCCCGCCCTCTCCCTCTTGCGGATGCCCTGCGCCATGCTGCTGCCCGCACCCTCGCTGTCCGCCTCTCTCGCTCAGAGCCCCCACTCCGTCCGGAAGTTGTCCACTATTTACTGGACTCTGACGCCAACTCTTGGAGCATGCGTAGGACCAAGGCTCACTGGTTTAGACTGGCTTCGAGCTTGTCGAAGCTTGCAGCCTTAGCACATTGGCTCGACTCTGTGAGATATTGGAGCCAACCGTATACAACCATTTTGGTGCATGTCCTCTTTGTAATTCTCATCTTGTTCCCTAACCTGTTCTTACCAACATTCTTCCTGTATTTGTTCTTTATTGGGGTGTGGAGGTACAGATACAGGAGAAACCTGCCTGAAAATATAGATATTCGGCTATCGTGTGTTGATTTGGTCAGTTTAGATGAGCTTGACGAGGAGTTTGAGGGGTTTCCTGCAAATAGGGCGGCTGAGGTGGTGAGGGTGAGATATGATAGACTGAGGGCAGTCGCTGGGCGAGCCCAGACCTTGTTGGGTGAGGCAGCAGGGTTTGGTGAGCGGCTCGAAGCATTGCTCGATTGGCGGGACCCGCGTGCCACATTGATGTTTGTGGTTGCATGTTTGGGGGTCTCAGGTGTGCTTTATGCTATACCATCTAAGGTAGTAGCAATTGGACTGGGGCTCTATTTCTTCAGGCACCCTCGGTTCAGAGGAGACTCACCACCGGCTGTTTTGAACTTTTTCCAACGATTTCCATCACTTGCCGACCGAATTCTTTGA

>Amtri00009.279

ATGAGCAACCTGAAACTGGGTGTGGAGGTAGTAAGTGCCCTCAACCTCATGCCCAAAGATGGTCAGGGATCCTCCTCCCCCTTTGTCGAGGCCCACTTTGATGGCCAAAAGTTTCGCACCACTATCAAAGAGAAAGATCTCAACCCTGTATGGAATGAGAAGTTTGTATTCACTGTAACTGATCCCGTATCTCTACCTCACAAATACATAGAAATTTACGTTTACAATGATGTGAAACCCTCTCACGCCAAGTCTTTCCTTGGATATGTTCGCCTCTCTTGCACAACGCTAGTCCCAGACTCAGAAGCCCTTGTTCAACACTACCAACTCGAGAAACGATGGGTTTTATCTCGTGTGAAAGGAGAGGTAGCCTTGAAAGTCTATCTGATTGATCCCTTTATGTCACAAATGCACGACCCGCAAAAATTGTCTGACCCACTGTTTGATCTTCTTCAGCAAAATGATCCACTTCATCTCAATGAACCGCTTCATATCAATCCCAATGTAAACACCACTGCCACCACAACGAACACTAGGCAATTTTATCACCTTAACCGAAATGAATACCACTCAGAACCCACAAAGGGTGAGGTACAAAGTGACCTAAGGCCCCCGCCATTGGTTGTAAGGCCGCAGCAATCAGCTTATCAAGGTCCTATGTCTTCGACCTCAGGTGACTATGCATTAAAAGAAACAAGCCCGTTTTTGGGAGGGGGAAGAGTGGTAGGGGGGAGAGTGGTGCAAGTTGGGTCTAGTGGGGGTAAGCCAACAAGCACATATGACTTGGTTGAGAGCATGCAATATTTGTTTGTGAGAGTAGTGAAGGCTCGAGACCTCCCTTCTGCTGATATGACTGGGGGTTGTGATCCTTTTGTGGAGGTGAAGGTTGGGAATTACAAAGGCATCACGACTCACTTCGAGAAGAAGCAAAACCCCTATTGGAATGAGGTCTTTGCCTTCTCAAGAGAGCGGTTGCAAGCATCAGTGGTTGAAGTGGTGGTGAAGGACAAGGATTTGGTAAAGGATGATATAATGGGTTGTGTGAGGTTTGACCTTAATGAGGTCCCAACACGTGTGCCCCCCGATAGCCCCCTTGCCCCTGAATGGTATCGCCTTGAAGACAAGAAGGGAGAGAAGATAAAGGGTGAGCTTATGCTTGCTGTTTGGCTTGGGACCCAAGCCGATGAGGCCTTTTCAGAAGCGTGGCACTCAGATGCAGCTACTTTGAAGACTGATAATGTCAATAGCATTCGGTCAAAGGTGTACGTTGCACCCAGACTCTGGTATGTGCGTGTGAATGTGATTGAGGCCCAAGATCTGCACATTCCAGACAAAACCCGGCTCCCAGATGCCTTTGTGAAGGTTCAGCTTGGCTCTCAATTCCTCAAGACCAAGCCAGTCCAAAGCCGAACCTTAAGCCCAGTTTGGAACGAGGACCTCCTGTTTGTAGCTGCTGAGCCATTTGAGGAACCACTCATATTCACTGTAGAAGACCGAGCTGGCCCCAACAAGGATGAAGTCTTGTGTCGAGCTGATCTCTCTCTCACCTCTGTCGAGCGTAGAGCCGACGATCGGCTCATCCACTCTCGCTGGCTCTACCTAGAGAAGCCTAAGGATGCCATCTTGATCGATGGGGATGCAGTTAAAGAGGCAATTAAAAAGGAGAAGTTCTCGAGCCGACTCCATGTAAGGGTTTGCTTAGATGGTGGATACCATGTGTTGGATGAGTCGACTCATTATAGTAGTGATCTAAGGGCAACTGCTAAACAGCTTTGGAAGCCATCAATTGGGGTATTAGAACTTGGGATTTTAAGTGCTCAAGGCCTAAACCCAATGAAATCTAGGGAAACTCGAGGCACTTGTGATGCATTTTGCGTGGCTAAGTATGGGCAAAAGTGGGTTCGTACTCGTACAATTGTGGATAGTTTGGCACCCAAGTGGAATGAGCAATACACATGGGAGGTATATGACCCTTGCACTGTGTTGACCATTGGAATGTTCGATAATGGGCAGCTTGCACATGGTTCAGAGAAGGCTGGGGAGAAAGCGGGAGAGAAGGCAGCAAGTTTGGGCAAGGATTTGAAGATAGGGAAGGTGCGAGTGCGGCTATCAACGTTGGAGACAGGGAGGGTGTACACACACTCATACCCATTGCTTGTCTTGCACCCATCAGGGGTAAAGAAAATGGGGGAGCTTCATCTTGCTATTAGGTTCTCATGTACATCCATGTTGAACATGATGTACCTATACTCCAAACCGCTTTTGCCTAAGATGCACTACATTCGCCCGCTCTCTGTTATCCAGCTAGACTCACTAAGGTACCAAGCTACGGCCATTGTGGCTGCTCGCCTTAGCCGCTCTGAACCCCCCCTCAGAAAGGAGGTTATCGAGTACATGTCTGATGTTGATTCCCACCTATGGAGCATGCGTCGGAGCAAGGCCAACTTCTTCAGACTCATGTCTGTCTTCTCTGGCCTCCTTGCTACCGGAAAATGGCTCGGGAATATTTGTGCATGGCGGAATCCAATTACCACGGTGCTTTTTCACGTGCTATTCCTCATCCTCGTTTGCTTCCCTGACCTCATCCTCCCAACCGCCTTCTTGTACATGTTCTTGATTGGGTTATGGAACTTCAGGTATCGACCGAGGTACCCACCCCACATGGACACGCGACTCTCGTACGCTGAGGCTGTACACCCAGACGAGCTCGACGAGGAGTTTGACTCATTCCCCACATCTCGGCATGCTGACATAGTGCGCATGCGATATGACAGGCTACGGAGTGTCGCCGGGCGGATACAGGCGGTGGTTGGGGATGTGGCCACACAAGGGGAGAGGCTCCAGGGATTGCTCAGCTGGAGAGACTCTAGGGCCACCGCTATCTTTGTGGCCTTTTGCTTTATTTCTGCTGTGGTTCTTTATGTTACACCACTGCAGGCAGTGGTCGTGTTTTATGGATTCTACCATATGAGGCACCCGAGGTTTAGACACAGGCTTCCATCTGCTCCTCTCAACTTCTTTAGGCGCTTGCCAGCTCGAACTGATAGTTTGTTATGA

>Amtri00022.254

ATGCAGAGGCCGCTTCAATCAGAGGACTATTCCTTAAAGGAGACATCACCCCATCTCGGAGGAGGTGGCGTTCATGGAGATAAGCTCACGAGCACTTATGATCTTGTCGAGCAAATGCAGTACCTCTATGTCAGGGTTGTAAAAGCCAAAGACCTCCCTACAGATGTTACAGGAAGTTGTGATCCCTATGTGGAGGTGAAGCTTGGAAACTACAAGGGAACAACCAGGCATTTTGAAAAGAAGACAAACCCTGAATGGAACCAGGTATTTGCATTCTCTAAAGAACGAATCCAATTCTCTGTGATCGAAGTCACTGTGAAAGATAAGGACCTGGTAAAAGATGATTTTATTGGGAGAGTTACGTTTGATCTCAATGAAATCCCTAAAAGAGTCCCCCCTGATAGTCCATTGGCTCCACAGTGGTATAGGCTTGAGGACCGAAAGGGAGATAAGGCTAAAGGCGAGCTTATGCTCGCCGTTTGGATGGGTACACAGGCTGACGAAGCTTTTCCTGAAGCTTGGCACTCTGATGCAGCAACCATACATGGTGAAGGTCTTAGTAATATAAGATCGAAGGTTTATTTGTCACCCAAGCTCTGGTATCTTAGAGTTAATGTTATCGAAGCCCAAGATTTGGTGCCTAGTGATAAGGCTCGGTTCCCTGAAGTTCATGTCAAGGTTATGCTTGGGAGCCAGGGTATGAAAACTAGGGTTTCTCAGAGCAGGAGCTTGAGCCCACTTTGGAATGAGGATTTGATGTTTGTTGCTGCTGAACCCTTTGAGGAACACCTGGTTATTAGTGTTGAAGATAGGATTGGACCCAATAAAGATGAGCTAATGGGTAAGTGTGTGATTCCATTGCAAAGTGTAGAGAAAAGGTTCGATCATAAGCCTGTCAATTCGAGGTGGTATAATCTGGAGAAGCATGCCATTACGGAGGGGGAGAAGAAGAAGGAGGTCAAGTTCGCAAGTAGGATTCATCTGAGGATCTGTTTGGATGGTGGCTATCATGTTTTGGACGAGTCCACTCATTATAGTAGTGATTTGAGGCCCACTGCTAAGCAACTTTGGAAACCCAGTATTGGGGTTCTTGAATTGGGTATTTTGAATGCTCAAGGCCTCTTGCCCATGAAAACAAAGGATGGGAGGAACACTACTGATGCTTATTGTGTTGCCAAATATGGCTCAAAGTGGATTCGAACCAGAACGATCATAGACAGTTTCACTCCCAAATGGAATGAGCAATACACATGGGAAGTTTATGATCCGTGTACTGTGGTTACAGTTGGGGTTTTTGATAATTGCCATTTAGCTGGGGACAAAGCTAATGGAACCAAGGACTCGAGAATTGGTAAAGTTAGGATTCGACTCTCTACTCTCGAAACTGATCGGGTTTACACCCATTCTTACCCCCTTTTGGTTTTGCATTCTTCTGGGGTTAAGAAAATGGGTGAGGTTCAATTAGCAGTTCGGTTTGCTCGCTCGTCTCTGCTTAATATGCTGCATATCTATTCCCAACCTTTGCTTCCTAAAATGCATTATCTTCACCCCTTATCAGTGACGCAGCTTGATAGCCTGAGGCACCAAGCTACCCAAATTGTTTCGATGAGGCTGAGTAGGGCCGAACCCTCTTTGAGGAAAGAGGTTGTTGAGTATATGCTAGATTATGATTCTCATATGTGGAGTATGAGGAGAAGCAAAGCGAATTTCTTTAGAATCATGTCGGTTATGAGTGGGATGATTGCTGTAGGGAAATGGTTGGACAAAATTTGTAATTGGAAGAATCCCCTAACAACTATATTGGTACACATCCTCTTCCTCATTCTGGTTCTCTACCCTGAACTCATACTCCCGACCATCTTCCTCTACATGTTTTTGATTGGTATATGGCACTATCGATGGAGGCCAAGGCAGCCTCCTTACATGGATACTCGACTCTCTCATGCTGAAAATGTTCACCCTGATGAATTGGATGAGGAATTTGATACTTTTCCCACTTCGAGGCATTCTGATATAGTGAGGATGAGGTATGATAGACTGAGAAGTGTGGCTGGAAGAATACAAACTGTAGTGGGGGATATGGCCACTCAGGGTGAAAGATTGCAGTCCTTATTGAGCTGGAGGGACCCAAGGGCCACTGCCCTGTTCGTTACCTTCTGTTTTCTGGCAGCAATTGTTCTTTATGTGACTCCTTTTAGAGTGGTGGCCATTTTAACAGCCTTTTATGTGTTGAGGCACCCGAGATTTCGCCACAAGCTCCCCTCTGTGCCTCTGAACTTCTTTAGGAGGCTCCCTGCAAAGACTGATAGCATGCTATGA

>Amtri00022.265

ATGAAGCTAGTGGTTGAAGTAATAGAAGCCCATGATCTTATGCCCAAAGATGGTGAAGGCTCAGCCACCCCCTTTGTTGAAGTAGACTTCGAAAACCAACGCATTCGAACCAAATCCCTCGAAAAAACACTAAACCCCCACTGGAACGAGCAGCTCATTTTCAACGTTACCGACCCCAAAAACCTCCATAACAGAGGCATCGAGGTTTACGTCTACAACGAGAAGAGGTCCCTGCACAGGCGCAACTTCCTCGGAAAGGTAAGAGTCTCAGGCTCTAGTGTAGTCAGAGAGTCCGAAAAGGCTCTCCAAACCTTCCCCTTGGAAAGAAGAACACTCTTCTCATTTGTCAAGGGAGATATCACCCTCAAAATCTACCTGTCCACCAATAACAAACTTGAAACACCACCACCCTTGATTAATAATACCAGTACTAGCCCCACAACCTTAGCCAAACCACCACCACCATCCACCACTGATAGTCCTGATAACAAGGCTCTAACTTCACCACCATCTACTACTGATAGTACTGCTAAAACTACTGATAATACCCCAACTACACCACCACCAACTGATTCCAATACTAGTAATTCTATTAGTACTAAATCTGTTGGTGAAGTTGCACCGCCTGAGGAGATCAAAGAACCAATAGAAGTGAAAATCTCGACAACTCAAATTGTAAACAAACAAGAAGTTACTCAACAACCAAGTAGAGTGGTCGAACAGAGAAGGCCACAGGGCATCCCAGTTGTAACCATGAACAATTACCAAAATATTGTTCCCCCTCCACCAGGGACTCACCATCATGGGGACTTTGGGCTCAAGAACACGAGCCCTCACCTTGGCACGGGGTGGTCGAACCGAGAATTTGCTAGCACATATGACTTAGTGGAGCAGATGCACTATCTTTATGTGCGCGTGGTTAAGGCCAAGGACCTCCCTGCTAGCTCAGTGACAGGTAGTTGTGACCCTTATGCCGAGGTCAAGCTTGGTAACTATAAGGGTACCACAAAGCATTTTGAGAAGAAGCTTAACCCTGAGTGGAACCAGGTTTTCGCTTTCTCAAAGGAGAGAATTCAGTCCTCCATGCTCGAGGTCTTTGTTAAGGATAAGGAGATGGTGGGCAGAGATGATTACCTAGGAAGGGTTGTGTTTGACCTAAATGAAGTCCCCAAGCGGGTTCCACCTGATAGCCCTTTAGCTCCACAGTGGTACAGGTTGGAAGATAGAAAGGGCGAGACCAAGGTTAGGGGAGAGATGATGCTTGCAGTTTGGATGGGAACACAAGCTGATGAGGCTTTCCCTGAAGCATGGCATGCTGATGCAGCAACAGTTCAAGGAGAAGGGGTTTTCAGCATAAGATCGAAGGTTTATGTGTCCCCTAAGCTTTGGTACCTAAGAGTTAATGTGATCGAGGCTCAAGATCTGCAACCGAGTGACAGGGCTCGGGTTCCTGAAGCTAGTGTAAAAGTTCAGGTGGGACACCAAGTGCTCAAGACCAAGCCTTCGCCAGTTCGAACCCCGAACCCACTCTGGAATGAAGACCTAATCTTTGTGGTAGCTGAGCCCTTTGAAGAGCAGCTTGTTCTCATGGTGGAAGACAGGGTTAGCCCATCAAAGGATGATGTTTTGGGGAAGATTGCACTCCCCTTAACAATATTCGATAAGAGATTGGACCATAGGCCAGTACATTCTAGATGGTTCAACCTCGAGAAATTTGGGTTTGGAGTTTTAGAGGGAGACAAGAGGAAAGAGCTCAAGTTCTCTAGCAGGATCCATTTGAGGGTGTGCCTAGAAGGAGGTTATCATGTGTTGGACGAGTCAACGATGTACATAAGTGACCTTCGCCCAACTGCAAAGCAACTATGGAAGCCCCCGATTGGGATTTTGGAGGTGGGTATATTGAGTGCTCAAGGGTTGCTTCCAATGAAAACCAAGGATGGGAGACAAACAACCGATAGCTACTGTGTGGCCAAGTATGGACCAAAGTGGGTTCGAACCAGAACAATTATAGACAGCCTCAGCCCCAAATGGAATGAGCAATACACATGGGAGGTCTATGACCCTTGCACTGTCATTACCCTAGGAGTCTTTGATAACTGCCATTTGGGCACACCAACCAGCTCAGCCAGCGATTCAAGAATCGGAAAGGTAAGAATTCGTCTCTCCACCTTAGAAACAGACCGAATATACACCCACTCCTACCCACTCTTGGTTCTTCACCCATCTGGCCTAAAGAAGATGGGTGAGCTTCAACTAGCCGTTAGGTTCACATGCACATCTCTTGTGAATATGGTCCATCTCTATGGCCATCCCCCACTTCCCAAGATGCATTACATACATCCATTCAGTGTGAACCAGGTCGATAACCTAAGGTACCAAGCAATTAACATAGTTGCCATGAGGCTTGGGAGAGCAGAGCCCCCACTGAGAAAAGAGGTTGTCGATTACATGCTAGACTTTGATGCTCATGCATGGAGCATGAGGAGAAGCAAGGCCAACTTCTTTAGAATTATGTCACTCCTATCAGGCGTGATATCGGTGGGAAAGTGGTTCGATGACGTGTGCCATTGGAAGAACCCCATAACCACAGTGTTGGTTCATATCCTATTCCTCATATTGATCTGGTACCCAGAACTCATACTACCCACCTTGTTCCTATACATGTTCTTGATAGGGATTTGGAACTTCAGGTTCAGGCCTAGGAACCCACCTCATATGGACACCAAGCTCTCCTGGGCTGAGGCAGTGCCCCCCGATGAGCTCGATGAAGAATTTGACTCTTTTCCAACTTCAAGGCCTCATGATGTTGTAAGGATGAGGTATGATAGGCTGAGGAGTGTGGCAAGAAGGATTCAAACTGTGGTGGGAGACATGGCTACACAAGGAGAGAGGTTTCAAGCCCTTCTGAGTTGGAGGGACCCTAGGGCTACAAGCCTTTACATTCTCTTCTGTTTAATTGCAGCAGTTGTGCTCTATGTAACTCCCTTTAGGGTTGTAGCTCTCCTCTTGGGGCTCTTTGTGCTTAGACACCCGAGGTTTCGAAGCAAACTACCTTCCGTTCCAAGTAATTTCTTCAAGAGACTGCCAGCTAAAGTTGACAGTATGCTATGA

>Amtri00050.35

ATGAGTAAAGATAAGCTGGTGATAGAGGTAGTAGCGGCCCACAATCTCCAGCCCAAAGATGGACAGGGCTCTTCGAGCCCCTTTGTCGAAGTCCACTTCGAGAACCAGAGGCTCCGCACCCAGTCCAAGTTGAAGGACCTTAACCCTGTGTGGAATGAGATGCTTGTCTTCAACATCGGCGACCTCGCCGACCTCCCCTACCGGACGATCGAGCTCAACATATACAATGAGAAGAAATCAGGGCTTAGCCGAAACTTCCTCGGAAAGGTCAGGATATCAGGTTCAAGTGTTCCCCCTCATGGCCAAGAGGTGGCTCAGCTCTGCACCCTCGAGAAAAGAAGCCTCTTCTCTCACATCAGGGGTGAAATAACCCTCAAGGTCTACCACACAAACGACCCGAAGATTGTAGCAGCATCACCGGGTGGTGGTTCTGTTGTTTCGGCAGAGGAGCCGAAGCCTTCCGATACGAAGAAAGAAGCCAAAGCTCAAAAGCAGCAGCAACAACAACTTTCTTCTAGCTTGAATCAGCAGCAAGCGATGCAGCAACAAGGGAAGGCTCCGGAGCAAAAACAGAGCAATGAGAAGCCGCCTGTGATAGTTGCTGCTGCGGGTCCTATAGCTTCACAACCGCCACCCCAGCAGCAACAGCAGCCAAGCGAATACAGTCTCAAGGAGACGAAGCCACAGTTAGGCGGAGGGGCTTTGTTCAGGGACAAGAGTAGCTGGACCTATGACTTGGTGGAGCAAATGCAGTATCTCTACGTACGCGTGGTGAAGGCGAGAGACCTTCTGACCAATCTTACTGGCGGGTGTGATGCTCACGTTGAGGTGAAGGTGGGGAATTACAGAGGGGTCACGAAGCCGCTTGATAAGAGGGGAGGGAACAACTTGGAGTGGAACCAGGTGTTTGCATTTTCAAAGGATTGCATTCAGTATTCAATGGTGGAGGTGGCAGTGAAGGAGAAGGATGACACATTGATGGGGAGAGTGGCATTTGATTTGAGCGAGGTGCCCAGAAGGGTGCCACCGGATAGTCCGTTGGCTCCTCAGTGGTACAGGCTCGAGGGCAAGGGCAGGGGTGAGGTGATGGTCTCAGTGTGGATGGGGACACAGGCCGACGAGGCCTTTTCAGAGGCTTGGCATTCGAAGGCAGCTGCTGTTCATACAAGTGATGGGCTTGCAGTCATCAAATCAAAGGTTTATCTCTCTCCTAAACTCTGGTATTTGAGGGTCGGAGTGATTGAGGCACAGGACCTGTTGCAGGCTGGAAGTAAAAATGGGGCCGTTAGGTACGCGGAACTGAGTGTGAAGCTGCAATTAGGCAACCAGGTTGCGAAGACCAGGCCTTCTGTTTTGAGGAACTCGAGCCCCCGGTGGAATGAGGACCTGATGTTTGTGGCTGCCGAGCCCTTCGAGGACAGCCTTATGATTGTGGTGGAGGATAGGGTGAGCCCAAACCAGGATGAAGTGCTCGGAGGGGTGCTCATACGCCTTGCCACAGTCGAGCAGCGAACTGATGATAGGACGCCATTGTCAAGATGGTTCACCCTTGACAAGCAGCAAGGCCTAAAGCCCTCCTCGCAGGCCAGTGGGGGTGGCCGGATTCACTTGCGCATGTGCTTAGATGGCGGCTACCATGTGCTTGATGAGTCTGCAATGCATAGCAGCGACCTCAGGCCGACTGCCAAGCAGCTGTGGAAGCCACACATTGGGGTCTTAGAGATGGGAATTCTTGGTGCCGATTCCCTGTTACCTATGAAAATCAAGGATGGTAACGGCACCACTGATGCTTACTGTGTGGCCAAGTATGGCCAGAAGTGGGTTCGAACCCGGACAGTTGTCGATAGCCTCATGCCCAAATGGAATGAGCAGTATACATGGGAAGTATTCGACCCCTGCACCGTGGTTACAATTGGTGTCTTCGACAATGGCCACCTTCATACCAGTCCCGGCAATGGCACAATGCTCCGTGATTCAAGGATTGGGAAGGTGAGAATCAGGCTATCGACTCTCGAATCAGACCGAGTCTACACCCATTCTTATCCTCTCCTACTCCTCCATTCCTCTGGTGTGAAGAAGATGGGCGAGCTCCATTTGGCCTTGCGCTTCTCCTGCTCCTCACTCCCCAACGCTTTACATCTCTATATGATGCCCCTTCTTCCGAAAATGCACTACCTTCACCCCCTCTCTGTGGCACAAGTTGAAGCCCTGCGCTACCAAGCCATGAACATGGTGGCCATGCGTCTAGGCAGGGCTGAGCCCCCCTTGTGGCGTGAGGTGGTTGAGTACATGCTAGACCTCGACTCCCATATGTGGAGCATGCGTAGAAGCAAGGCCAACTTCTTCCGCCTCATGTCGATTCTCTCCTGTGTGGTCGGGGTCGGTCGTTCGGTTGAGTCGGTGTGCGGTTGGAGGCGCCCAGTGGTCTCCACCCTTGTTCTGGTGATCTTTCTTATAGTGGTCTGTTACCCAGAGCTTGCTCTCCCAACCCTTCTAATGTACCTCTTCATGCTCGGGCTTTGGCGTTTCAGGCGAAGGCCGAGGCATCCCCCTCACATGGATACACGCCTCTCACATGCTGACTCCGTGTCGGTTGACGAGCTCGATGAGGAATTCGATACATTTCCGACGAGCCGGAGCTCAGACGTGGTGAGGATGAGGTATGACAGGCTGAGGAGTGTGGCGGGTAGGATACAGACAGTGGTGGGGGACATGGCGACGCAGGGGGAGCGGCTGCAGTCGCTGCTCAGCTGGAGGGACCCGAGGGCGACCTCGCTGTTCCTCACATGGTGCCTAGCGGCTGCAATCGTGTCTTATGCTGTTCCATTTCAGCTGCTGCTCACTGCGGCTGGTTTGTATGCACTGCGCCCGCCCAAGTTCAGGAGTCGTTTACCTTCGCCTGCACTCAATTTCTTCAGGCGGTTGTCTGCAAGGGCTGATAGCTTGCTGTGA

>Amtri00078.73

ATGAGCGGGAGAAGGCTCATAGTCGAAGTGGTTGATGCTCGAGACCTGCTGCCGAAAGACGGGTTGGGGAGCTCGAGCCCTTATGTAGTCGTGGATTTCGATGGCCAGAGAAAGCGAACAAAGACTGTTTTCAGAGACCTCAATCCTTCATGGCACGAGAAGCTTGAATTCGATGTCACTGATCCTGAAACAGTGGCGTATGAAGACCTCGAAGCCGAGGTTTACAATGATAAAAGGACTGGAAATGGAAGAAAAAGCCATTTTCTTGGTCGTGTGAGATTAAACGCGGGCCAATTTCCAAGGAAAGGGAATGAGGCCTTGGTGTATTTTCCATTAGAGAAGAAGAGCTTTCTCAGTTGGATAAGAGGGGCATTGGGGTTGCGGATTTATTACACCGACGAGCCGGAAAACCCAGTTCCAGCGCCGGAAGATGAGAAGTCTCCGGAGAATGAGACAAAGCCGCCAGAAGATTACACAGAACCGCCAGAAGATGACACAAAACCGCCAGAGAGTGAGCTCTGTCCGATGCCCTCAAGCCACGCCACCCAAATAGAGGAGCAGAACAGCCCTCCGGAAAACACAGCTCCACCGCCGGAAAATGCAGTTCTGGCGACGGAAGCCTTTATTCCGGGGCCTGAAACCCAACCTCCACCTCTGGAACATGCACCCCCACCCCCAGAAACTCGGATTCCAGTACGCCCACCAATAGGAATGACTCCCCGGAGAGTGTACGGATCCGGCGCGTTTACCGGTGAAACGAAGATTTATCCGGCGCATGATTTGGTCGAGGCCATGAAATACCTCTTTGTGAGAATCGTGAAGGCTCGAGGCTTAGCTCAGGGTTCGGGACTCGAACCGGTGGTCAAGATCAGGCTTGGTAGTTATAGTGTACGTAAGAGACCAGATTCTCGGAACTCTTTCGAGTCGAATCCAGAATGGAACCAGGTCTTCGCTTTTGGACGTGAGAGACTCGATTCGAGCGCCAACACCTTAGAGATCTCGGTTTGGGCTCCTTCTCCAACAACAGGAGGTGGTGGGTTTCAGGAGGGCGCCGGAAATCCCGGTGCCGGAGATGGGATTTACCGCAATGCACCAGGTGAATTTCCCGGCAATGTACAAGCTGAGTTTCCCGGCTGGGGACATGGCTCCTCGGGTGATGTGCCAGGTGGGTTTCCCAGCACTGGAGGTGAGCCTCCTATGGACGGTGGATTTCCCGGCTCCGAAACTGGTTTTCGTGGCAATGCAGCTGGTGGATTTTCTGGGAATGCACCAGCTGGTTATCCCAGTGCCGGAACTGGTTTTTCTGGCCAACTTTTAGGTGGGGTGTGCTTTGATTTGTCAGAGGTCCCGGTACGATCACCATCTGATAGCCCTTTGGCTCCACAATGGTACAGATTGGATGGCGATGGCCACGTCTGCGGCGATATAATGCTCAGTGTGTGGATCGGAACCCAAGCCGATGACTCGTTCTCAGAAGCCTGGCAATCCGATGCGCCCCACTTAGCCCACACTCGCTCCAAAGTGTACCATTCTCCGAAGCTTTGGTACCTTCGACTTACTGTAATTGAAGCCCAAGACCTTCCAATCTCGAGGTTTTCAGAGACCCGTGTCAAGGTCCAACTCGGGTTCCAGTCCTCCAGGACACGTCCTTCTCCACCGAGTCAGGGGGGTTCGGTGTCTTGGCATGAAGACCTCCTCTTTGTGGCTTCCGAGCCATTGGAGGATGCAGCGATTCTATTGGTTGAAGAGAGGTTTTCGGGCAAAGAACCGAGTCTTGTAGGCCATGTAATGATACCATTGGCTCCGGTTGAGCAGAGGCTTGATGAGCGGCTCTTGCCTTCGAGGTGGTTCGTGCTCGAGAGCCCCACTGCATTTGGTGGTGAGAGTGGCGGGGGCACAAATGCGACCAACTATAGAGGGAGAATGCACCTACGGTTGTGCCTCGAAGGTGGGTACCATGTTTTGGACGAGGCTGCACATTTTTGCAGTGATTTCAGGCCTACGGCGAGGCAGCTATGGCGCCCGCCGATAGGCACACTCGAACTTGGGATCTTAGGTGCTCGAGCCCTCTTGCCTATGAAGACCCAAACCCACGGTCCGCTGGGCTTGGGTCCAGGCGCAATGAAGGGCTCCACCGATGCATATTGTGTCGCTAAGTACGGGCACAAATGGGTACGTACCAGAACCCTAACCGATTCGTTCGATCCTCGATGGAACGAGCAATACACCTGGCAAGTATACGACCCTTGTACGGTGCTCACGATTGCCGTCTTTGACAATTGGCATATGTTCTCCGACAAGTGTCCGATACGTCCCGACACTCGCATTGGCAAGATACGTATTCGTGTCTCGGCCCTAGAGGGCAACAAGGTCCATACACGAGCATACCCATTGCTCGTGCTTTTGCGCTCTGGTCTTAAGAAGATGGGTGAGCTCGACCTAGCCATACGATATACATGTGGACCATTGATAGACACCTGTGGAGTTTACACACGCCCAATGCTCCCTCGAATGCACTATACACACCCGATCGGTCTAGCCCAGCAAGACGCACTCCGTGCTGCAGCAGCACGGTCGGTGGCAGCGTGGCTCACACGCTCAGAGCCTCCCCTGGGCCCTGAGGTGGTCCAATACATGCTCGACTCTGACTCCCACACATGGAGCATGCGTCGGTCGAAGGCTAACTGGTTTCGAGTCATGTCCGTTCTTGCTTGGCTAGGTGGGCTGGCTCACTGGGTCCACGACGTGCGCCAATGGCGAAACTCGGTCCAAACCATCTTGGTCCATGTGCTATTCTTAGTTCTAGTTTGGTACCCTGAGCTTGTAGTACCCACTATCTTCTTCTATATAGCCATTGTGGGGGCTTGGTACTACAGGTTTAGGCCAGTGGGGCCAATGGGGATGGATACCAGGCTGTCTCAAGCCGAGTCATCGGATTTCGATGAGCTCGAAGAGGAGTTCGATCTGATACCCAGCTCAAGACCGAGTGAAGTGGTTCGGGCTCGGTACGATAGGCTCAGGGTTTTGGCGGGACGAGTGCAGAGTGTGCTTGGCGACCTTGCTGCTCAGGGGGAGCGAGCCCAGGCCTTAATTGCGTGGCGTGACCCTCGAGCCACGAGGCTCTTCGTGGGCTCATGTTTGGCTGCCTCTTTTGTGATGTATGTCTCTCCTCCTAAGGTGGTGGCTGTGGCTTTGGGGTTTTACTTCTTGAGGCACCCTATGTTTAGGGACCCTATGCCACCTCCTAGCTTGAACTTCTTTAGGAGGCTGCCAAGCTTATCGGATAGGATGCTATGA

>Bradi1g13460

ATGGTGGAGGAGGGGGCGAAGCGGCGGGTGGTGGTGGAGGTCTGCAACGCGCGGAACCTCATGCCCAAGGACGGGCAAGGGACGGCGTGCGCCTACGCCGTCGTCGACTTCGACGGCCAGCGCCGCCGCACCGCCACCCGGCCGCGGGATCTCAATCCGCAGTGGGGGGAGCGCCTCGAGTTCCTCGTGCACCACCCCGACGCCATGACCGGGGAGACGCTCGAGCTCAACGTCTACAACGACAAGAAGGCCATCGCCGGCGGGGGAAGCGGCGGCGGTGGGAGCGGCCGCCGCGGTGGCACGTTCCTCGGGAAGGTCAAGGTGGCCGGCGCGTCATTCGCCAAGGAAGGGGACGAGACGCTCGTCTACTACCCGCTCGAGAAGCGGAGCGTCTTCTCGCAGATCAAGGGGGAGATCGGGCTCAAGATTTGGTTCGTCGACGAGCCGCCCCCGCCGCCGCCTGCCCCTGCAGCTGAAGAGAAGGCCGATGCGTCGGCTGAGAAAAAAGACGCGACCGAGGAGAAGGGCAAGGAATCAGCCGCTGCCCCTGCGGCTGAGGAGAAGAAGCCTGAGGAAGCCGCTGCCGAAGCAAAGAAAGCAGAGGAGGCCAAACCAGAGGAGAAGAAACCGGAGGCGGGCAAGAAGGACGGCAAGAAAAACTCGCCGGAGAAGGGGAAGAAGGACGGCGAGAAACCCAAGGAGGAAGCTAAATCCAAAGAGGAGAAGAAGGAGGCAGCGCCTCCTTCCCCGTCGAAGGCCCCGCCGCCCTCGCCGTCGAAGATGCAGCTGTCCAACGCCGGAATCGCCGGCGACCTCGAGATCCGTCCGCAGAGCGCTGCCGAGCGGAGCATGACCGCCTCCGGCGGCAGCGCGTCGTACGACCTGGTGGATCGCGTGCCTTACCTGTTCGTCCGGCTTCTGAAGGCAAAGCATCAAGACGACGGCAACAAGCAGCCGCTGTACGCCCAGCTGTCCATTGGCGCTCACACCGTGCGGACTCGATCCGCCGCGGCCGCCGGCGAGTGGGACCAGGTGTTCGCCTTCCACAAGGCGAGCCTCACCGCCTCCTCGCTGGAGGTGACCGTCCATGAGGAGGCCAAGAAGCCGGAGAAGGAGGGGGAGCCCGTTCCGGCTGACCCCAACCTGGGCTTCGTCTCCTTCGACCTCCAAGAAGTCCCCAAACGATCGCCGCCCGACAGCGCGCTCGCTCCCCAGTGGTATACCCTGGAGGGCCACGCCGATGACGGCACCTCGGCCTGCGACGTGATGCTCGCCGTGTGGGTCGGCACGCAGGTCGACGAGGCATTCCAGGAAGCATGGCAATCTGACTCCGGTGGCAACCTGGTCCACACCCGCTCCAAGGCATACCTGTCCCCCAAGCTCTGGTACCTCCGCCTCAGCGTCATCCAAGCGCAGGATTTGCGGCTGCCGTCCCCGCCGGACGCCAAGGCGAAGCAGTACGCGCCCAGCTTCCCGGAGCTGTACGTGAAAGCGCAGCTGGGCGCCCAGGTGTTCAAGACCGGGCGCATCGCGCTGGGCAGCGCGGCGGCCGGCGCGTCCAACCCGAGCTGGAACGAGGACCTGCTGTTCGTCGCCGCGGAGCCGTTCGACCCTTTCCTGACCGTGGCCGTGGAGGACATCTTCTCCGGGCAGCCGGTCGGCCAGGCGCGCGTGCCGCTGTCCACCGTGCACCGGCGTTCCGACGACCGGGCCGAGCCGCCGTCGCGGTGGCTCAACCTCTGCGGCGACGAGGCCCGGCCGTACGCCGGGCGGGTGCACGTGCGCGTGTGCCTGGAGGGCGGGTACCACGTGCTGGACGAGGCGGCGAACGTGGCGAGCGACGTGCGCGCGGCGTCGAAGCAGCTGTCGAAGCCGCCGGTGGGCATGCTGGAGGTGGGCGTCCGCGGCGCGGCGAACCTGGTGCCGATGAAGATCGCCAAGGACGGGGCGAGCGGGTCGACGGACGCGTACGTGGTGCTCAAGTACGGGCCCAAGTGGGCGCGCACGCGCACCATCCTGGACCAGTTCAACCCGCGGTGGAACGAGCAGTACGCGTGGGACGTGTTCGACCCCTGCACCGTGCTCTCCATCGCCGTCTTCGACAACGCCCGCTACTTGAACGGGAAGCTGCCGCCCAAGGACGCCCGCATCGGGAAGCTCCGCATCCGCCTCTCCACGCTGGATACCAACCGGGTGTACGTCATCAACTACGCGCTCACGGCCGTGCACCCGGTCGGCGTGCGCAAGATGGGGGAGCTGGAGCTGGCCATCCGCTTCACCTGCCCGTCCTGGCTCACGCTGATGCAGGCCTACGGGAGCCCGCTGCTGCCGCGCATGCACTACGTCAAGCCGCTCGGCCCGGCGCAGCAGGACGTGCTGCGGCACACGGCCATGCGCATCGTGTCGGGCCGGCTGGCGCGGTCGGAGCCGCCGCTGGGGCCCGAGGTCGTCCAGTACCTTCTGGACACGGACACCCACACGTGGAGCATGCGCCGGAGCAAGGCCAACTGGTTCCGCGTGGTGGGCTGCCTGTCGCACGTGGCCACGGCGGTGAAGTGGGGCCACCGGGTGCGCACCTGGGAGCACTCGCCGACCACCGTGCTGGTGCACATGCTGCTGGTCGCCGTCGTGCTCTGCCCGGAGATGATCCTGCCCACCGTCTGCCTCTACCTCTTCCTGGTCCTGCTCTGGCGCTACCGCTCGCGCCCGCGGGAGCCGACGGGCATGGACCCGAGGCTGTCCCACGTCGACAGCGTCAGCCCCGACGAGCTCGACGAGGAGTTCGACGGGCTCCCCTCGGGCCGCCCCGCCGACGTCGTCCGGATGCGCTACGACCGGCTGCGCGCCGTGGCCGGGCGCGCGCAGACGCTGCTCGGCGACGTGGCGGCGCAGGGGGAGCGCGTCGAGGCGCTGCTGTCCTGGCGCGACCCGCGCGCCACGGGGGTGTTCGCCGTGGTTTGCCTGCTCACCGCGCTCGTGCTCTACGCCGTGCCGTTCAAGGTGCTGCTGCTCGGGATGGGCTTCTACTACCTCCGTCACCCCAGGTTCCGCGGCGACATGCCGTCCGCCGGGTTCAACTTCTTCCGCCGCCTGCCCTCGCTCTCGGACCGGGTTCTTTAG

>Bradi1g27740

ATGAGCAATCTTAAGCTCGGTGTTGAAGTTGTCAGTGCTCATGACCTCATCCCGAAAGAGCAGGGGACAGCCAATGCCTTTGTAGAGGTCGAATTCGATGACCAGAAGTTCCGCACAGCCATAAAAGACAGGGACATTAACCCTGTCTGGAACGAGCAGTTCTTCTTCAACATATCCGATCCTTCACGTCTTCAGGAGAAAGAGCTCGAGGCCTATGTGTACCATGCAAACCGTGTCAGCAATAACAAGACATGCCTCGGCAAGGTTCGCATCTCCGGTACATCGTTTGTCAGCCAATCTGATGCAGCACCCCTGCATTACCCTCTGGAGAAGCGCACAATCTTGTCACGTGCTCGTGGCGAGCTTGGTCTTAGAGTCTTCCTTACAGATGATCCGTCAGTGAGGGTGTCCGCTCCAGGTCAGGATTTTAATTTTGCAAGCACACCCACCACTGCCCAAGAGCAGGCAACTGTCAATTCTATTCCAAATCCTTTCCAAGAAACCAGGACAAATGAAGTGAGGCAATTCCAACACTTACCACGGGAACAGCAGCGACCAGCCCCGATGGCGGGGCAGCAATACTACGCTCAGGGACAGGGTTCATATGGAGATCAGCAGCAGAGAAACTACGCCGCTGCTGGGAACAAACCTGAAGCCCCTCAAGTAAGGATGTATTCTGCAGGTCCACAGCAGCCTGTAGACTTTCAGCTGAAGGAGACAAGCCCAACGCTTGGTGGTGGGCGTATTGTTCATGGTCGGGTGATGCCTGGTGAAAAGGCTGGGGCATATGACCTTGTGGAGAAGATGCACATCCTCTTTGTGCGTGTAGTCAAGGCCCGTGAGCTGCCCCACATGGACCTCACAGGGAGTCTTGACCCTTATGTTGAGGTGCACCTTGGGAACTACAAAATGAAAACAAAGTTCTTCGAGAAGAACCAGAGGCCTGAGTGGGATGAGGTGTTTGCATTCCCTAAGGAAGTAGTGCAGTCATCAACACTTGAAGTTGTTGTGAAGGATAAGGACATCCTTCGGGATGACTATGTCGGCCGAGTAATGCTTGACCTGAATGAGGTGCCTGTAAGGGTCCCTCCTGACAGTCCATTGGCGCCAGAGTGGTATCGCCTTATGGGCAAGGATGGGATGAGGGACAGAGGGGAGTTAATGCTCGCAGTATGGTATGGAACTCAAGCAGATGAATGCTTCCCAAGCGCCATTCATGCAGGATCAACACCAATTGATTCCCATTTCCACAACTATATCCGTGGTAAGGTCTACCCTGCACCAAGAATGTGGTATGTAAGGGTCAATGTAATTGAGGCACAGGATATATTCACAATGGAGCACCACCACATACCTAATGTGTTTGTAAAGGTGAGGATTGGTCACCAATTGCTGAAGACAAGGCAAGTTCGCTCACCAACCAAAAACTTCATGTGGAATGAGGAGATGATGTTTGTCGCAGCAGAGCCTTTTGAGGATGACTTGATCATACAAATAGAAGATCGTGTTGCACAGGACAAAGATGAGGTAATTGGTGAAGCTATCATACCTATCGCAAGGCTTCAAAAGAGGGCTGATCACAAGGCGATAGTACGCCCAGTGTGGTTTGATCTGAGGAGACCAGGACTGATTGACATGAACCAGCTAAAGGAAGACAAATTCTATGCAAAGATAAGCCTCCGGGTTTGCCTTGAAGGTGGTTATCATGTCCTTGATGAGTCAACACAGTATTGTAGCGATCTCCGACCAACAATGAAGCAGCTGTGGAAGCCACCAATCGGGTTGCTTGAAGTTGGCATTCTAAGTGCAAATGGTCTTACCCCAACAAAGACCAGGCAAGAAAGAGGGTCATGTGATCCATATTGTGTTGCCAAGTATGGTCATAAATGGGTGCGTACTCGCACAATAGTTGACAACTTGAACCCTCGATTCAATGAGCAGTATACATGGGATGTCTTTGACCATGGAACAGTACTCACCATTGGTCTGTTTGACAACTGCCACATTGGAGGAGACAACCACGACCACGGCCACGGCCACAGCCACAGCCACAGCCACAGCCACAGTTCCCCCAGCAGTATGGATAAACCCATTGGGAAAGTGCGAATCAGGATCTCAACCCTTGAGACTAGGCGGGTGTACACGCACACATATCCACTGCTTGTTCTCCACCCATCAGGAGTTAAGAAGATGGGTGAACTTCACCTTGCTATCAGGTTCTCAGTCACATCCCTGCTCAATGTGTTCCTTACATACTCACATCCACTCTTGCCCAAAATGCACTACTCCCAGCCATTGTCAATAGTCCAGCAGGAAATGCTACGTCACCAGGCTGTCCAGGTTGTTGCACAGCGCCTGGGGCGCATGGAGCCACCAGTTCGCAGGGAAGTTGTTGAGTACATGTCAGACGCCCGCTCTCACCTGTGGAGTATGCGACGAAGCAAAGCAAACTTTTTCCGTCTTATGCAAGTATTCTCTGGATTCATTGCTGCAGGGAAGTGGTTTGGTGATGTTTGCCAGTGGAAAAATCCAGTCACCACAGTCTTGGTTCATGTGCTCTTCATCATGCTCGTCTTCTATCCAGACCTCATCCTTCCGACAATCTTCCTGTACATGTTCTTGATTGGGTTGTGGAATTACCGGTTCCGGCCACGTGTTCCACCACATATGAACACGAGGATATCTTATGCTGATGTTGCCCATCCAGACGAGCTTGATGAGGAATTTGACACGTTCCCAACCTCAAAGAGCCCAGACCTGATTAGGATGAGGTATGACAGGCTCCGGCATGTTGCCGGGAGGATACAGACAGTTGTTGGTGACATCGCAACTCAGGGTGAGAGAATACAGTCACTGCTGAGCTGGAGGGACCCAAGAGCAACAGCTATGTTCCTATTATTTTGCCTGTTCACCGCAATTATTTTGTATATTACACCATTCCAAGTGATTGCACTCTGCCTTGGGTTCTTTTGGATGAGGCACCCACGGTTTCGCCACAAGGTGCCTGCAGCGCCGGTAAACTTCTTCAGGAGGCTACCTGCAAAGACAGATTCTTTGCTGTAA

>Bradi1g35960

ATGATGCAGAGGCCATTCCGTCCAGAGGAGTACTCCTTGAAGGAGACTTCTCCTCACCTTGGAGGTGCAGCTGCAGGTGACAAACTCACCACCACCTATGACCTGGTCGAGCAGATGCAGTACCTTTATGTTCGGGTGGTAAAAGCAAAGGAACTGCCAAGTAAAGACATCAGTGGAAGCTGTGACCCATATGTTGAGGTGAAGCTTGGGAACTATAAGGGCACAACTCGGCATTTCGAGAAAAAGACCAATCCTGAGTGGAACCAGGTATTTGCCTTCTCAAAGGAGCGCATTCAGGCATCAGTAGTGGAAATCATCGTCAAAGATAAAGACTTTGTCAAGGATGACTACATTGGACGAGTTTTATTTGACCTGAATGAAGTCCCGAAGCGAGTGCCACCTGACAGCCCGTTGGCCCCACAATGGTATAGGTTGGAAGAACGGAATGGGCACAAGGTGAAGGGAGAGTTGATGTTAGCTGTTTGGATGGGTACTCAAGCAGACGAAGCATTCCCTGAAGCATGGCATTCTGATGCGGCGTCAATCCCTGGCGATGGACTTGCAAGCATTAGATCAAAAGTTTATCTTACTCCCAAGCTTTGGTATTTGCGTGTCAATGTCATTGAAGCACAAGATCTTATGCCAAATGATAAGACCAGGTTCCCTGAGGTCTACGTCAAAGCAATGCTAGGCAATCAAGCTCTTAGAACAAGGGTATCACCAGGCAGAACACTGAACCCGATGTGGAACGAGGACTTGATGTTTGTTGCAGCTGAACCATTTGAAGAGCACCTGATTTTGAATGTAGAAGACAGGATTGCCCCAGGGAAGGATGATGTGATTGGGAGAACTGTCATTTCCCTGCAGCATGTAGCTCGGAGGCTGGATTACAAGTTGCTGAACAGCCAATGGTATAATCTTGAGAAGCACGTGATTGTGGATGGTGAGCAGAAGAAGGAGACCAAGTTTTCAAGCAGGATTCACTTGAGAATTTGCCTTGAAGGTGGATATCATGTCTTGGATGAGTCGACACATTACAGTAGTGATCTAAGGCCAACTGCAAAACAGCTGTGGAAGCATAACATTGGTGTCCTTGAGCTGGGTATCTTGACAGCTCAGGGCCTGTTGCCTATGAAGACAAAGGATGGGCGTGGCACAACTGATCCTTACTGTGTGGCTAAGTATGGACAGAAATGGGTCCGGACAAGGACTATCATTGATAGTTTCACACCCAAGTGGAATGAACAGTACACTTGGGAGGTGCATGATCCATGCACTGTGATCACAATTGGTGTATTTGACAATTGCCACTTGAATGGTGGGGAGAAAGCTAATGGTGCCCGGGATACCAGAATTGGGAAGGTCCGCATCCGCCTCTCAACACTTGAAACTGATCGAGTATATACCCACTCATACCCTCTTATTGTTTTGACACCCGGTGGCGTTAAGAAAATGGGTGAGGTACAACTTGCTGTCCGGTTCACATGCTCGTCGCTGCTAAACATGATGCATCTTTATTCACAACCCTTGCTTCCAAAGATGCACTATATACAACCACTGTCTGTGATTCAGGTAGACAATCTAAGGCGCCAAGCCACCAACATTGTCTCAACAAGGCTGAGCCGTGCAGAACCACCGCTCCGAAAAGAAATTGTGGAGTACATGCTGGATGTGGATTCCCATATGTGGAGCATGAGAAAGAGCAAGGCAAACTTTTTCCGTATCATGGGTGTCTTGAGCCCTCTGATTGCGGTGGCTAGATGGTTTGATCAGATCTGTCACTGGAGGAACCCACTGACCACTATACTGATCCATGTCCTGTTTGTGATATTGGTCTTGTATCCTGAGTTGATATTGCCTACAATCTTCCTGTACCTGTTCCTGATTGGAGTGTGGTACTACCGGTGGCGGCCGAGGCAGCCTCCGCACATGGACACTCGCCTCTCACATGCGGAGACTGCGCATCCTGATGAGCTTGATGAAGAGTTTGACACCTTCCCAACATCTCGTCCACCTGACATTGTCAGGGTGCGTTATGACCGCCTCCGCAGTGTTGCTGGGAGGATACAAACTGTTGTCGGTGATCTTGCGACACAAGGAGAAAGGCTGCAGTCTTTGCTGAGCTGGAGAGACCCAAGAGCCACTGCACTGTTTGTGACCTTCTGCTTCATCGCTGCGATTGTCCTGTATGTCACTCCGTTCCGTGTTGTGGTCTTCCTTGCAGGGCTGTACACCTTGAGACACCCGAGGTTCCGCCACAAGATGCCGTCTGTCCCGCTGAACTTCTTCAGGAGACTGCCAGCAAGAACTGATAGCATGCTCTAA

>Bradi1g35970

ATGGGCGGGAACCAGGAGGCGCACCACGAGGACTTCCAGCTCAAGGACACGAACCCGCTGCTGGGCGAGCAATGGCCCAAAGGCGCAGCAGGCCCGGCACGCCCAGCCGTGGGCGGCGGCATCGCCGGTTGGCTGGGCATGGACAAGCCATCAAGCACGTACGACCTTGTGGAGCAGATGTTCTTCCTGTACGTGCGCGTGGTGAAGGCCAAGGACCTGCCCCTGAACCCGGTGACCGGCGCTCCCATGGACGCCTACGTGGAGGTGAAGCTGGGCAACTACAAGGGCACCACCAAGCACCACGACCGGCGGCTCAACCCGGAATGGGACCAGGTGTTCGCCTTCTCCAAGTCCCGCGTCCAGTCCAACGCCCTGGAGGTGTTCCTCAAGGACCGCGAGATGCTGGGCCTGGGCCGCGACGACTACGTGGGCCGCGTCGTCTTCGACCTCGGCGAGGTCCCCACCCGCGTCCCGCCCGACAGCCCGCTCGCCCCGCAGTGGTACCGCCTCGAGGACCGCCGCGGCGGCAAGGTGCGCGGCGAGCTGATGCTGGCCGTCTGGATCGGCACCCAGGCCGACGAGGCGTTCCCCGAGGCCTGGCACTCGGACGCCGCCACCGTGCGCGGCGAGGGCGTGGCCAGCGTGCGCTCCAAGGCCTACGTCTCCCCCAAGCTCTGGTACCTCCGGGTGAACGTGATTGAGGCCCAGGACGTGCAGCCGCAGTCCCGGGGACGCGCACCCGAGGTGTTCGTCAAGGCGCAGGTCGGGAACCAGGTCCTCAAGACGTCCGTGGCACCGGCGGCCGCGACGCTCAACCCGCGGTGGAACGAGGACCTGGTGTTCGTCGTGGCCGAGCCCTTCGAGGAGCAGCTGGTCATGACCGTGGAGGACCGCGTGTCGGCGCGCAAGGACGACTTGCTTGGGCGCGTCCAGCTCCCGCTCTCGATCTTCGAGAAGCGGCTCGACCACCGGCCCTTCGTGCAGTCCCGGTGGTTCGACCTCGAGAAGTTCGGCATCAACGCCATGGAGGGCGAGACGCGGCGTGAGCTCCGGTTCGCCAGCCGCGTCCACGTCCGCGCCTGCCTCGAGGGCGCCTACCACGTCATGGACGAGTCCACCATGTACATCAGCGACACCCGCCCCACGGCGCGCCAGCTCTGGAAGCCGCCCGTGGGGGTGCTCGAGGTCGGCATCCTCGGCGCGGCCGGGCTGCAGCCCATGAAGAACCGCGACGGCCGCGGCAGCACGGACGCCTACTGCGTGGCCAAGTACGGGCAGAAGTGGGTGCGCACGCGCACCATGATCGGCACCTTCAGCCCCACCTGGAACGAGCAGTACACCTGGGAGGTGTTCGACCCTTCCACCGTCATCACCATCGGCGTCTTCGACAACTGCCACCTCGGCAACAACAACAACAACAACAATGCAACCGGAGCGCCACCGCCGCCACCGGCGAGGGACGCGCGCGTGGGCAAGATCCGGATCCGGCTGTCGACGCTGGAGACGGACCGGGTGTACACGCACGCGTACCCGCTGATCCTGCTGCAGCCATCGGGGGTGAAGAAGATGGGCGAGCTCCGCCTGGCCGTGCGCTTCACCTGCCTCTCCATGATGAACATGCTGCACCTCTACACGCAGCCGCTGCTCCCCAGGATGCACTACCTGCACCCGTTCACGGTCACCCAGCTCGACGCGCTCCGGTACCAGGCCATGGGCATCGTGGCGGCGCGCCTCGCCCGCGCCGAGCCGCCGCTGCGCCGGGAAGTGGTCGAGTACATGCTGGACGTCGAGTCCCACATGTGGAGCATGCGGCGGAGCAAGGCCAACTTCTTCCGCGCCGTGTCGCTCTTCTCGGGCGCGGCCGCCGGGGCCCGGTGGTTCAACGACGTGTGCCACTGGAAGAACGTGGCCACGACGGCGCTGGTGCACGTGCTTCTGTTGATACTGATATGGTACCCGGAGCTCATCCTGCCCACGGTGTTCCTCTACATGTTCATGATCGGGCTGTGGAACTACCGGAAACGGCCGCGCCACCCGCCGCACATGGACACGAAGATGTCGTGGGCGGAGGCCGTGCACCCGGACGAGCTGGACGAGGAGTTCGACACGTTCCCGACGTCCAGGCAGCAGGACGTGGTTTACATGCGCTACGACCGGCTGAGGAGCGTGGCCGGGAGGATACAGACGGTGGTCGGCGACATGGCCACGCAGGGGGAGCGGCTGCAGTCGTTGCTCGGGTGGCGTGACCCGAGGGCCACCTGCCTGTTCGTGGTGTTCTGCCTCCTCGCCGCCGTCGTGCTCTACGTCACGCCGTTCCGGGTGGTGGCGCTCGTTGCCGGGCTGTATCTGCTCCGGCACCCGCGGTTCCGCAGCAAGCTCCCGTCCGTGCCGAGCAACTTCTTCCGGCGGCTGCCGTCGCGGGCTGACAGCATGCTCTGA

>Bradi1g56630

ATGGCGCCGGCGGGGACGGTCCGGAAGCTGGTGGTGGAGGTGGTGGAGGCGCGGGACCTGCAGCCCAAGGACGGCTTTGGCACGTCCAGCCCGTACGCCCGCGCCGACTTCGACGGGCAGCGCCGCAAGACCCGGACCGTGGTCCGCGACCTCAACCCGGTCTGGAACGAGCCGCTCGAGTTCACCTTCCCCGGCCCCGGCACCGGCGTGCTGGACCCCGTGGGAGGCGGGGAGCCGCTGGAGGTTTCCGTGCTCCACGATTTACGCGTCGGGCCCAGCCGCCGGAGCACCTTCCTGGGCCGCGTCCGCCTCGACGCGCGCCGCTTCTTCGTGCGCAAGGGCGAGGAGGCGCTCATCTACTTCCCGCTCGAGAAGAAGAGCTTCTTCGGATGGGTCCGCGGCGAGATCGGCCTCAAGGTCTACTACGTCGACGAGCCCGCTCCCGTTCCCGTCGCGCCGGAGCCGGAGCCGAACGCTGGCGCTGATCCTCCTCCGGCTGCTGCTGCACCCGACGCTGCCGAGGCGGTAGAAGAAGCGCCGCCAACGGCTCCTGATGCTGCTCCCGCGTCTGCCGACCCACCGCCGGTGCCGACAGAGGAGGCGGAGGCGCCAGAGGAAGCAGCACCACCGGCCGGCGAAGAAGCCGGCCCGGAGAAGCCACCGGATCAGGCTGACACGGCTGAGACAGTGGATGCCCCTGTAATGACATCAGAGGCGGTGCCGGCGTCCTCGGCGGAACCTGCGGCAGAGACCCCGCCCCCAATGCCGATGCCGATGCCGATGCCGAGGCAGGTGCTGCTCCCGGCGCGTCCGGCGCCGGCGCCGGCCGAGGTCCTGCCGGTGGAGCCATCGAAGCACGACCTGGTGGACAAGATGCCGTACCTGTTCGTCCGCGTGGTGCGCGCGCGGGGGCTTCCCGCAGGCGCGCACCCGCACGTGCGCGTGGCCGTGGCCGGCGGCGGCCGCCACGCGTCCACCCGGGAGGCGCGCCGGGGTGCCTTCTTCGAGTGGGACCAGACCTTCGCCTTCGCGCGCGACCCGGCGGACTCCCAGACAGGCCCCACGATGGAGGTGTCCGTGTGGGACCTCCCGCCTGACGCCGACGTGTCCGTCGCCGACGACCGCAGCTTCCTCGGCGGGCTCTGCTTCGACACCGCCGACGTGCACGCGCGGGACCCGCCCGACGGCCCGCTCGCCACGCAGTGGTACCGGCTGGAGGGCGGCCGCCGCAACGAGCGGGCCGCCGACCTCATGGTTGCCACGTGGGCCGGCACGCAGGCCGACGAGGCCTTCGCCGACGCCTGGAAGGCCGACTCCCCCCCGGCCCATGCCTCCTCCTCCACGGCCACCGCGTCCTCCTCTGCGTCCTCGAGCGCCAAGGTGTACGTCTCGCCCAAGCTCTGGCTCCTCCGGCTCACCGTCATCGAGGCGCAGGACACGCTCATGGCGGCCCGTGCTGACGCCGGTATCGCCGTGCGGGGCACGCTGGGCTTCCAATCGCTCAAGACCCGGACCACGGCAGCGGTGACACGCAACGGCGGTCCATCGTGGAACGAGGACCTGCTGTTCGTCGCCGCAGAGCCCTTCACCGACGGCGACTGCTTCGAGATCTCCCTCGAGGTGCGCCACGGCAAGGACGCTTTCACCGTGGGCTCGGCTAGCGTCTCGCTGGGGAGCATCGAGAGGCGCGTGGACGACCGCAAGGTGGCCTCCAAGTGGCTCGACCTTCTCCCCTCCGACGAGGCCGCCGCCACGAGAAAAGCCAACGGCAAGTTCAGGATGCCGGCGCACGTGCACGGCGGGCGGCTGCACGTGCGGGTTTGCCTGGACGGGGGCTACCACGTGGCGGACGAGCCGCCCTACGCGAGCAGCGACTTCCGGCCCTCGGCGCGGCAGCTCTGGCGGCAGCCTGTGGGCCTCGTGGAGCTGGGCGTGGTGGGCTGCAAGGGCCTCCTCCCGATGCGGGCCGCGGACGGGAAAGGCTGCACGGACGCGTACGCCGTGGCGAAATACGGGCCCAAGTGGGCCCGCACGCGCACCATCTCCGACAGCTTCGACCCGGCCTGGAACGAGCAGTACACGTGGCCCGTCTACGACCCTTGCACCGTGCTCACCGTCGGCGTCTTCGACGACCCGCCGCCTCCGCCGTCCGATGACGCCGACGCCGCCGTGACGCCGTCGAGGCCGATGGGGAAGGTGCGGATCCGGCTGTCGACGCTGGAGAACGGGCGCGTGTACCGGGGCTCGTACCCGCTCCTCATGATGCTCCCCACGGGCGCCAAGCGGATGGGCGACGTGGAGCTCGCCGTGCGCTTCGCCACTTCCGGAACCTTCCTCGACACGCTGCACGGCTACCTGCAGCCATCTCTGCCGCCGATGAACAACCTCCGTCCGATCCCGGCGGCTCACCGCGAGCCGCTGCGGCTGGCCGCGGCACGGATCACGGCGGGCCACCTGGCGCGCGCCGAGCCGCCGCTCAGGCGGGAGGTTGCCACGTGGATGCTGGACGCCGGGCCCGGGTCCGGCTCCAGCTCCAGCTTCAGCATGCGGAAGCTGCGGGCGAACTGGAACCGGGCAGCATCGGCGCTGACGTGGGTGTCCGGCGTGGCGAGGTGGGCGGAGGAGACGCGGACGTGGCGGAGCCCCGCGGCGACGGGGATGGCGCACGCCGTGCTGGTGCTGCTGGCGTGGCACCCGGACCTGGTGATCCCCACGCTGGCGCTCCACGTGGCGGCCGTGGGGGCTTGGAGGTACAGGCGGCGGCCACGGGCACCGGCGCCGCACCCGTGTGTGAGGGCGTCCATGGCGGAGGCGCCGGCCGAGAGGGAGGAGCTGGACGAGGAGTTCGACCCGGTGCCCAGCGCCAGGCCGCCGGAGACGGTGCGGGCCAGGTACGACCGGGCCAGGGTCGTCGGGGCCAGGCTGCAGGCCATGGTCGGCGACGTCGCCACGCAGGCCGAGAGGGTCCAGGCGCTCGTGTCCTGGCGAGACCCGCGCGCCACGGGGATGTTCGTCGCGCTCTGCGTTGCTCTGGCCATGGTGTTGTACGTTGTGCCGCTCAAGGTAGTCGTTGTTGTCGCGGGGTTCTACTACCTCCGACACCCCATGTTCAGGGACAGGACGCCCGCGCCGGCCGTCAACTTCTTCCGCCGCCTGCCTTCCATGGCCGAACGGATCATATAG

>Bradi2g16690

ATGGCAAGCCATAAGTTGGTGGCAGTGGTTGTCCGCGCAGAAGGTTTGTCGGCAGCAAGTGGAACTGTTGTCTTCGTCGGGCTGCGCTTCAACGGTGACACGCAGTACACAACCAGGAAAACCCACACGGTGAGTCCAAGTTGGAAGGAGTGCTTCTCATTTGACGTATCAGATCCGGAGAGGCTTGACGATCTTTCCCTCGATGCCAATGTATACAGCATTGATGAAAGAAGCAGCAGGTCCAGAAAGGTCAACCTTGGCAAGGTCCGGCTCCAAGGCACACGCTTCGTTCCATTAGATGAGGCGGCATCTGAGGCCTACACCTTGAAGAAACGGAGGAAGCTGTCCTGGGGTGGTAAAGGGAAGCTGGTCCTTAAAGTTTCCCTTGAGAATGTCAACCGCAGCAGTGATTCCACCACAGGGGATGCTGCTGCCGACGATTCTACTGTGAATAATGTGCGGGCTCAGGTCTTAACGGACGGTCAAAACAATGAGCTCCGCCGTTTGGCGCAGGTGTCCAGACGACCAGACTTCGCAGTGAGTTCTATTAGTCCATCCCTTGGTGCTGGACAAATGGTGGATGGCCGGCTTTCGCCTGTTGGGCACAGGGATCCAGTACCTGCTAACGATCTTGTGGAGGTGATGTGGTACATATTTGTCTCTGTCGTCAAAGGTAGAAACTTGCCGGCCATGAGCTCCCAGGGGTCTCTTGATCCTTATGTTGAGGTGGAGTTCGGCAGTTACAAGGTGGAGACGGAGAACCGTACCGGAGATCAGAACCCGGAGTGGGGGGTTGTTTTTGCCTTTTCCGACGAACACATCCAGTCATCAAAAGTACAGGTTATTCTCAAGAGTAGAGATGAAGTCAGACCTGATGTTTTGGGGAAGTTATCCATCGATCTCAGAGACATCCCCATGCATCAGCCACCAGAGAGTGCATTGACCGCCCAGTGGTACAAGTTGATGAATGAAAGAATGGAGACAACAGATGGCGAACTGATGCTTTCTATCTGGAAAGGCACCCAAGCCGACGAGGCTTTCCGTGATGCTTGGCACTCGGACTCTGCAACTCATGTCCATCCCAGTCCAATAACCTCCGAGCTCAGGTCAACAGTATACTCTGCTCCAGTAATGTGGCATGTAAGGCTGGACATAATACGAGGTGTGGTCCCTGCTTCTGCCGGCAATACACGTCTTTCAACTCTACGTGTGAAGTCACAGATAGGACGCCAAATTCACAGGACGAGGCCAGCTGACATAATCAATCGTTCTTGGAGTGATGAGCAAACATTCTTCTTCATGGTTGCCGAACCATTCGAGGATGACCTAATTTTGTCCATTGAGAGCTTTCAAGTAAACGAAGATATCAGCTTTGTGGTACCTTTAGCTTCGATACAGAAGCAAACCGACGGCCGAGAAATCAATACCCAGTGTATTGAATTTCAGAAGTTAGATGGCTCAAATGGTAATAAGACGGTCGCCAAGGTTGATATTCGTCTCTGTTTGGAGGGACGGTATTGGGTACCTGTCGATTCCATTTGCTACAGTGGCGACTTAAGGTCGACGTTGGATCAGCACTCGAGCAGTAAAAAGATCGGTTTGGTTGAGCTGGGCATCATCAGAGCGGAGGCGCTTGCGCCCATGAGGACCATCGGTGGGAGAGGGACATACTGTGTGATCAAGTATGGTCGGAAATGGGTGCGAACTCGTACCATTAAGGATAGCCAAAGTCCGAGGTTTAACGAGCAATACTCATGGGATGTCTATGACCCATGTACTGTTGTTACCATTGGCATTTTCGACAATGGCCACATCATTGAAGGCAGCAGTACTGATGTTCCCAGTTCCAAGCACACCATGATCGGCAAGGTCCGCATTCGCCTCTCCACTCTTATGCGTGGCCGGCTGTATGCGCTCTCGTATCCGCTCACCGTCGTCAGTCCAGTTGGCGTCAGGAGAATGGGTGAGCTCCATGTAACCATCAGATTCTCCTATAAGACATTTCCCAGCATGTGCCGCGCGTACTTAAGGCCACTGTTACCGGCGCTGCATTACACCATCCCGATAGACGCCATGACGACTGGTCTTCTTCACACTGAAGCCATATACACCGTGGCAACTTGTCTAACCCGGCAGGAGCCACCACTACGGAAGGAGGTCGTCCAGTCCATCTGTGAGGGGGATTGCGACATATTTAGGATGCAGAAGACCAAGACCGATTCAACGCTCTCCAGGTTTGTTGCATTCTGCCGAGATATAGCTATGTGGAAGGACACCGCCACTACAGTGCTCTGCCATGCTATTTTCCTTATGGCTTTGAGTAATCTGGAATTTCTTATCGCAACGGTTGCGGTCTCTCTCTTTATGCCTATGAGCTCGAACATTGGGCTGAGACATACGCTCCCAGAGCACCTGGATCCATCCATTTCTGGCGTAGGAGATGCGCACCTCGGTGACCTTGACGAAGAGTTTGACCAGTTTCCTGGCATCAAGACACAGGAAACTGTTACAATGTGGTATGAGTACGAACGATTGAGGACCCTGACAGAAAGGTTACGGAAGGATGCCAGATCTATAATGGTTCATTTGGAGAGAGTGGAAGCATTATTTAGCTGGAGGGATCCCACAGCCACGAGCATTTTCTTCTTTTTCTGCATGGCAATGTCAGCTGCATTGGTAATTTCCCCGACTGCGGTCATGTGCATGGGTGGACTTTATGTTATGAGGCATCCAAGGTTCCGGGGAGACACCCCGTCGGCGCTTCTCAACTTGTACAGTCGGTTGCCGTGCAAGCACAAATGCATGATGTAA

>Bradi2g24410

ATGAAGGGAGCAATGCAGCCGCGGCCGTTCATGATGCACGGCCCGATGCCTCCGCCGCCGCAGCAGTTTGGCCTCGTGGAGACGCGCCCGCCGCTGGCCGCCATGCTGCGGCCCCGCTTCAACATCCCGGGGCTGAACCCTTCCGCCGCCGCGGCGTCCGCGGCGGGCAAGATCTCTTCGACCTACGACCTCGTGGAGCCGATGAGGTTCCTCTACGTCCACGTGGTCAAGGCGCGGGACCTCCCCGCCGTCTCCCCCACCGGCTCCATCGACCCCTTCGTCGAGGTCAAGCTCGGCAACTTCAAGGGCCACACATCCGTCCACGGCGCCAACCACAACCCGTCCTGGCAGCAGGTGTTCGCCTTCTCGGCCACGCACCTCCAATCGCATCTGCTCGAGGTGTCCATCAAGGCCAAAGATCTCGCCGGCGGAGACGACCTCATCGGCCGCATGGCGTTCGACCTGTCCGAGGTGCCGGTCCGCGTGCCGCCTGACTCCCCGTTAGCCCCCCAATGGTACAGGCTCGAGGGGAAACGGGGTGAGAAGCTTCCCCGCGGCGAGATCATGCTCTCCGTGTGGCTCGGGACCCAAGCCGACGAGGCGTTCCCAGAGGCCTGGCATTCCGACGCCCATGGGGCGGCTGGCCCGGCGGCCGTCTTGTCCACTCGCGCCAAGGTATACTTCTCGCCCAAGCTCGTGTACCTCCGCGTCGCCGCCATCGGGGCGCAGGACCTCATGCCCCACGACACGTCCCGCCCCATGAGCGCCTCCGTCAAGCTGCAGCTCGCCGGGCAGGTGCGTCGCACGCGTCCTGGGGGCCCGCCGGGGACGCCCAACCCAATGTGGAACGAAGAGTTCATGTTCGTCGCCTCCGAGCCCTTCGACGAGCCCCTGGTCGTCACCGTGGAAGACCGTGTCGCGCCCGGCCGCGACGAGCCGCTCGGGCGCATTATACTGCCCCTCAACGCCGCAATGCCACGCCATGACCACTTCGGCAAGCCTGTGGAGCCGCGCTGGTACAGCCTTGGGCGTCCCAGTGACGATGGTGAGAAGAAGGAAGGCAAGTTCGCGAGCAAGATACAGCTTCGGATGTCACTGGACTTTGGATACCATGTTCTCGACGAGTCCACTTACTACAGCAGCGATCTCCAGCCATCGTCAAAGCACACGCGGAAGCCAAGCATTGGGATCCTTGAGGTGGGAGTTTTAGGTGCACGCAACTTGATACCGATGAAAGCCAAGGATGGGCGCACCACCGACGCTTACTGTGTTGCCAAATATGGACCGAAATGGGTGCGCACAAGAACAATCCTTAACACACTGAATCCGCAGTGGAATGAGCAGTATACTTGGGAGGTGTTTGATCCATGCACTGTGATCACAGTGGTAGTGTTTGACAATAGCCAGATTGGGAGCAAGAGTGCTGATGCCCGGGATGAGAGTATTGGCAAGGTCAGGATCCGTCTCTCGACACTGGAGACTGACCGGGTGTACACCCATTTCTACCCTTTGCTGGCTCTTAAGCCAAGTGGCCTCAAGAAGACCGGTGAGTTGCATTTGGCTGTGCGGTTTACATGCACGGCATGGGTGAACATGATGGCGATGTATGGCCGTCCCCTGCTGCCAAAGATGCACTACACACAACCAATATCAGTGATGCAGTTGGACTACCTGAGGCATCAGGCGATGCAAATTGTTTCAGCGAGGCTTAGTCGTGCTGAGCCTCCACTGCGCAGGGAGGTGGTGGAGTACACGCTCGACGTGGGATCCCACATGTTTAGTCTCCGGCGCAGCAAGGCCAACTTTTATCGGATCACCTCTCTGTTCTGTTGTTTTGCGGCAATGGCCAAGTGGTATGATGGCATCAGGAGCTGGCGAAACCCGATTACAACTATGCTGGTGCACATGTTGTTCCTCATACTGATCTGCTACCCGGAGCTCATCCTGCCTACCATCTTCCTCTACATGTTCATGATCGGACTGTGGAACTATCGTTATAGGTCAAGGCATCCACCGCACATGGACACTAAGCTATCACAAGCTGAGTTTACACACCCTGATGAGCTTGATGAGGAGTTCGACACATTCCCAACCAACAGATCAGCTGACATTGTAAGATTGCGCTATGACAGGCTGAGGAGTGTTGGAGGTCGTGTCCAAACAGTGGTTGGGGACCTGGCGACACAAGGTGAGAGAGCTCATGCATTGCTGAGCTGGAGAGACCCTCGGGCCACTGCCATCTTCATATTCTTATCCCTAGTTGTAGCCATTGTGCTGTATGTGACGCCATTCCAGGTTTTGCTGGTCATTACCATGCTTTACTTGTTGCGCCATCCCCGGTTCCGCAGCAGGATGCCATCTGTCCCATTCAACTTCTACAGAAGATTGCCTGCCAAGTCTGATTTGCTCCTTTGA

>Bradi2g42110

ATGGCGGCCGGGGGGCCGCCGCCGCAGCAGATGGTGCGGAAGCTGGCCGTGGAAGTGGTGGACGCACGGGACCTGGTGCCCAAGGACGGTCTCGGCACGTCGAGCGCCTACGCCGTGGCGGACTTCGACGGGCAGCGGAAGCGCACGCGCACCGTGCCGCGGGACCTCAACCCGCAGTGGCACGAGCGGCTCGAGTTCGCCGTGCCCGACCCGGCCACCATGCACGCCGAGGCTCTCGACGTCTCGCTCTACCACGACCGCCGCTTCAACCCTTCCGGCGGCGGGGGCAAGAACAACTTCCTCGGCCGCGTCCGCATCTACGGCTCCCAGTTCTCGCGCCGCGGGGAGGAAGGCATCGTCTACTTCCCGCTCGAGAAACGAAGCCTGCTCAGCTGGATCCGCGGAGAGGTCGGGCTCAAGATTTACTACTACGACGAGCCGGCCGTGCCGCCGCCGCCTCCTCCTGAGGATAATAAGCCGCCGGAGGGGGGTGACAACGCGCCGCCGCCTGAGGTTCCCCCGGAAGCGCCCAGGGAGCTCCCCCCGGAGGTCCCCGAGCCTACGGAGGCCGCCGTCGAGGTGCAGCAGCCACAGTTTCAGCCTCCGGTCGTCATCGTGGAGGAAGCTCCGATGCACGGGCCACACGGCCCGATGATGATGCCGCCAATGCACGGCCCGTATGGCCCGATGATGCCGCCGATGCAGCACGGCCCGATGATGCCACCCCCGGTGACGATGCACGCCCGAATGATGCCGCCGCAGCCGGAACCGGAGCCAGAGCCGGAGCCGCAGCGTGAAGCAGGTGGGCCGGATGGCGCAGAGCATTACCCGCCTGAGTTGCGGAAGACGCGGATGGCGTCCAGCACGGAGCGTGTCCGCCTCCCGCGACACCCGAGCGGCGGCGGCTACGGGCCGCCCGATTACTACGCCGCCTCGCCCCGCGTCATTTCCGGGCGGTTCGTGTCCACCGGTGAAGCCGTCGAGCCGGTGCAGTCGACGTACGACCTGGTGGAGCCGATGCGGTACCTCTTCGTGCGCATCGTCAGAGTGCGCGGCATCCGCCCCTGCGAGGGCCCTTACGTGAAGATCCAGGCGGGCCCGCACTGCCTCCGCTCCCGGCATGGCCGCGACGTCTCCGGCACGGGCAGCCCCGAGTGGAACCAGGTGTTTGCCATCAGCCACGCCAAGCCGGAGCCGACGCTGGAGATATCCGTGTGGGACGGCGGCGCGCCGTCTCCCGCGGACGCCTTCCTCGGCGGCGTCTGCTTCGACCTCTCGGACGTGCCGGTCCGCGACCAGCCGGACGGCCCGCTGGCGGCGCAGTGGTACCGGCTGGAGGGCGGGGACCCCGGCATGGTCACGGGGGACATCATGGTGTCCGTGTGGATCGGCACGCAGGCGGACGACGTGTTCCCGGAGGCGTGGAACACGGACGCGCCCTACGCCGCGTACACGCGCGCCAAGGTGTACCAGTCTCCCAAGCTCTGGTACCTGCGGGCGTCCGTCATCGAAGCGCAGGACCTGCGCGTGCCGACGCCGCCTCCTGGGCTGCCGTTCGACGTGCGTGTCAAGGTGCAGCTCGGGTTCCAGTCGGCGCGCACCCGCCGGTCAGTGGCCAGCAGCAGCGGCTCGGCGTTCGCGTGGGCAGAGGACCTCATGTTCGTGGCTTCCGAGCCGCTGGACGACACCCTTGTCCTGCTCGTGGAGGACCGGTCCATGATCAAGGAGCCTTCTCTGCTCGGCCATGCCACCATCCCCGTCAGCTCCGTGGAGCAGCGCCTGGACGAGCGGCAGCTGGTCGCGTCAAGATGGTTCAATCTCGAAGGCGGGATGGGCCATGGTCATGGTCACGGAGACGCCGGGGATCATCCGCATGGACAGCCTGCAGGGTTCTACTCCGGGCGGCTGCACCTTCGGCTTTCCCTGGAAGGAGGGTACCACGTGCTGGACGAGGCGGCGCACGTGTGCAGCGACTACCGGCCGACGGCGAAGCAGCTGTGGAAGCCGCCGGTGGGCGTGCTGGAGCTGGGCATCGTGGGCGCGTGCGGCCTGCTCCCGATGAAGACGAAAGGAGGGTCCAAGGGGTCGACGGACGCCTACTGCGTGGCCAAGTACGGCAAGAAGTGGGTGCGCACGCGCACCGTGACGGACAGCTTCAGCCCGCGGTGGAACGAGCAGTACACGTGGCAGGTGTACGACCCGTGCACGGTGCTGACGGTGGCCGTGTTCGACAACTGGCGCATGTTCGCGGGCGCCGGGGACGAGCGACAGGACTACCGCATCGGCAAGGTGCGGGTGCGCGTGTCCACGCTGGAGAGCAACCGCGCCTACACGGCGTCGTACCCGCTGCTGGTGTTGCTGCGGTCGGGGCTCAAGAAGATGGGCGAGGTGCAGCTTGCCGTGCGGTTCACGTCGCCGGCCCACCTGCCGGACACCTGGGCCACGTACACGTCCCCTCTCCTGCCGCGGATGCACTACCTGCGCCCGATCGGCGTGGCGCAGCAGGAAGCCTTGCGTGGCGCGGCCGTGCGCACCGTGGCGGCGTGGCTGGCGCGGTCGGAGCCGCCGCTGGGTCCCGAGGTGGTGCGCTACATGCTGGACGCGGACGCGCACACCTGGAGCGTGCGCCGCGCCAAGGCCAACTGGTTCCGCATCATGGGGGTGCTCGCCTGGGCCGTCGGCCTGGAGCGCTGGCTCGATGGCGTGCGCCGCTGGCGGAACCCTTCCACCACCGTCCTCGTCCACGTGCTCTACCTGGTCCTCGTCTGGTACCCGGAGCTGGTGGTGCCCACGGCCTCGCTCTACGTGTTCCTCATCGGCGTCTGGTACTACCGGTTCAGGCCGCGGGCGCCCGCCGGCATGGACGCGCGGCTGTCGCAGGCGGACACGGTGGAGGGCGACGAGCTGGAGGAGGAATTCGAAGCCGTGCCGGCGCCCGACGTCCTCAGGCTGCGCTACGAGAGGCTGCGGACGCTGGCCGGGCGCGTGCAGCGCGTCATGGGGGACGTGGCGGCGCAGGGCGAGCGGCTGCAGGCGCTCGTCAGCTGGAGGGACCCGCGGGCCAGCCGGATCTTCGTCGGCGTGTGCCTCGCTGTCGCAGTCGCGCTCTACGCGATGCCGCCCAAGATGGTGGCCGTGGCCACGGGATTCTACTACCTCCGGCACCCCATGTTCCGGGACCCCATGCCCGCGGCCGCCGTCAACTTCTTCCGCCGCCTGCCCAGCCTCTCCGACAGGATGCTCTGA

>Bradi3g50990

ATGGCCTCCGGCGTCTTGCCGAGCTGGTTTGGCCGTTTCGGTCCACAGCCGCCATACGACGAGTTCGGGATCAAGGAGACGAGGCCACGCCTCCCCGGCGGGCGGACCGGGGGTTACGACCTGGTGGAGAGGATGGAGTACCTCTACGTGCGCGTCGTCAAGGCGCGTGAGCTCCGGTGGGGCGGCGGCGAGTTCGACCCGCTGGCGGAGCTGAGGCTCGGGAGCTACTCCTGCACGACGCGGCACATCGAGAAGACCGTGGCGCCCGAGTGGAACGACGTGTTCGCCTTCTCCAGGGAGCGTGTCCAGGCGTCGTTCCTGCACGTGGCCGTCCGCGGCCGGGGCTTCGCCGAGGGGGACTACGTCGGGAGCGCGCCCCTCGACCTGGCGGACCTCCCCGTGCGCGTGCCGCCCGACAGCGCGCTGGCCCCGCAGTGGCACCACGTCTTCGACCGGAACGGGGAGCGCGCCGGGGAGGTGATGCTGGCCCTGTGGATCGGGACGCAGGCCGACGAGTGCTTCCCGCTGGCCGTGCACGCGGACTCGGCGTTCGCCGTGGACGCCGACCTGGCCACGCACATCCGCTGCAAGCAGTACGCCGTGCCCCGGCTGTGGTACGTGCGCGTGAACGTCGTCGAGGCCCGCGACGTGGTCTTCGCGGACAAGACCCGCGCCGCCGGCCAGCTCTTCGTGCGCTCGCGGATATCCACGCAGGTGCTCAGGACCAAGACGTGCGCCTCCCGGCTGCCCTCCTACGGCTGGAACGAGGACCACCTGTTCGTCGCCGCCGAGCCGTTCGAGGATCACCTGACCATCTCCGTGGAGGACCGCGTCGAGGTCGATAAGGAGGAGGTCATCGGCCACGTCCACATTCCCTTCACGGAATTCGAACGCCGGTGGGACACACGCCCGATCCGCCCAAGATGGTACAATTTGCTGCAACCGGAAGGAGCCACGAAAATCGAGAAATTCTCTACCAAGATTTGCGTCCGGCTCTGCCTGGAAGGCGGGTACCGAGTGCTGTCAGAGCCTATCCACTACTTGAGCGACGTCCGCCCTGCGGCGAGGGAGCTGTGCCACAGGCGGCCGCCCATCGGCCTCGTGGAGCTCGGCATCCACAACGCGTTCGGGCTGAGCGCCCTGCGCGCGCGCAACGGGCGGGGCTCCTGCGACGCCTACTGCGTGGCCAAGTACGGCGCCAAGTGGTTCCGCACGCAGACCGTCATCGACAGCCTCGCGCCGCGGTTCCACCAGCAGTGCTTCTGGGAGGTGCACGACCACTGCACCGTGCTCACCGTCGCCGTCTTCCACAACTGCCAGATCGGCGAGAAAGGCGGCCTCGCGACCGGCGACCCCGTCAAGGACGTGCTCCTCGGCAAGGTGCGCATCCGGCTGTCCACGCTCGAGACCGGCCGCGTCTACACGCACGCGTACCCGCTCGTGTCCCTCCACGGTGGCGGCATCAAGAAGATGGGGGAGCTCCACCTGGCCGTGCGCTTCTCGGCCACATCCACGCTGGGCCTGCTCCAGACGTACGCGCAGCCGCACCTGCCGCCGATGCACTACCACTGCCCACTCTCCGTGGTGCAGCAGGAGACGCTGCGGCGCGAGGCGGTGGCGCTCATCGCGCACCGGCTGGGCCGGATGGACCTGCCGCTGCGCCGGGAGTGCGTCGAGCACCTCTGCGAGGCGCACGCGCTGCGGTGGAGCATGCGCCGCAGCAAGGCGCACTTCTTCCGCATCATGTCCGCGCTCGCGCCGCTCTTCGCGGCGCTCAAGTGGTTCGTCGACGTCTGCCACTGGAGGAACCCGGTGACCACGGTGGCCGTCCACATCATCTACGCCATGCTCGTGTGCTGCCCAAACCTCATAATGCCCACCTTCTTCCTCTACAAGTTCTGCATCGGCCTGTGGAACTACCGGCGCCGGCCCAGGCACCCGTGGCACGTCGACACCAAGGTGTCGCACGCCCACACGGCGCACCCGGACGAGCTGGACGAGGAGTTCGACGAGTTCCCGACGGCGCGGCACCCGGACGTCGTGCGCATGCGATACGACAGGCTGAGGAGCCTCGGGGCGCGGATACAGGAGATGGTCGGCGACGTCGCGGCGCACGTCGAGCGAGCGCGCTGCGTCATGACCTGGCGGGACCCCCGCGCCACCACTGTGTACCTTATGGTTTGCCTGTGCCTTGCTGTCATCACGTTCGCCGCGCCCTTCCAGGCGGTGGCGCTGCTGACGGGGTTCTACCTGATGCGGCACCCGAGTCTCCGGCAGAGGCTGCCCGATGTGCCAGCCAACTTCTTCCGGCGCCTCCCCTGTAAGGTCGATTGCCTGCTTTAA

>Bradi3g55250

ATGGGCAAGGCGGAGAAGCTGGTGGTGGAGGTGGTGGCGGCGCACAACCTGATGCCCAAGGACGGGCAGGGCTCCTCGTCGGCGTACGTGGAGGTGGAGTTCGAGCACCAGAAGCGCCGCACCCGGCCCAGGCCCAGGGAGCTCAACCCGGTCTGGAACGAGCGCCTCGTCTTCCCCGTCGCCGACCCCGGCGACCTCCCCTACCGCGCCATCGACGTCGCCGTGTACAACGACCGCGCCCTCGCCGGTGGGGCCGGGAGCGGCGGGCGGAACTTCCTCGGCAAGGTCCGCGTCCCGGCCGCCGGCGTGCCGGCACCGGGCGAGGAGGCTGTGACGCAGCTGTTTACTCTCGAGAAGCGCAGCCTCTTCTCCCACATCCGCGGCGAGATCACGCTCAAGGTTTACCGCATCGGCGGCGGCGGCGGCGGCGGCTCCGGGGACAATGTTGTTGCCAAGGCGTCGGCGTCCAAGCAGGAGAAGCCGACCAAGGTGGCGGTGAGTGGGCCGGAGGTGGTCGCCGCGCCGCATGCTAACGGGGGAAAGAAGCAGCACCATCCGCATCAGCATCAGCAGCAGCCGATTGTGGCCGTGCAGCCCCCGCCGCCGCAGCAGCAGCGGCAAGCGCCCATGGCCATGGACATTCTCCCTCAGCCGCAGCCGCAGGTTCCCATGGCAATGAAGCCGCCGGTGATGTTCGCGGACCACCACCACTACCCCGTCCCGACTGCCATGTTCTCCGGCCGCCCAGGCGACTTCTCCCTCAAGGAGACGCGCCCCCGGCTCGGCGGCGGCGCGAGCGCCGACAAGGCGAGCGCCACCTACGACCTGGTCGAGCAGATGCAGTACCTCTACGTGCGCGTCGTCCGCGCTCGCGGCGCGGCGGCGCCGGCCGAGGCCGTCGCGGAGGTCAAGCTCGGTAACTACCGCGGCCTGACCGCGGCCACTTCCGCCGGCTCCGGCGGCCACCACCACTGGGACCAGGTGTTCGCCTTCTCCAAGGAAACCATCCAGTCCTCCTTCGTCGAGGTCTTCGTCCGCGCCGCCCGCGCCGGCGGCGACGACCACGCCGGCCGCGTCTGGTTCGACCTCTCCGAGGTGCCCCGCCGCGCGCCGCCGGACAGCACGCTGGCGCCGCAGTGGTACGCCATGGAGGACCGCAAGGGCGAGCGCGGCGGGGTGGAGGTCATGGCCGCCGTCTGGTACGGCACCCAGGCCGACGAGGCCTTCGCCGAGGCCTGGCATTCCAAGGCCGCCGGCGTCCAGGGCCCTGGCCCGTTGGGCTCCATCAAGTCCAAGGTCTACGTCGCCCCCAAGCTCTGGTACCTTCGTGTGTCCGTCGTCGAGGCGCAGGACCTGCTGCCCATGGACAAGGGGCCCATGACCATGAGCCGGTACCCGGAGCTCTTCGTGCGCGCGCAGGTCGGCAACCAGATGCAGCGGACACGGCCCTCGTCGGTCGTACCCAACCGGGGCCCGTCGAGCCCGTTCTGGAACGAAGACCTGATGTTTGTGGTTGCGGAGCCATTTGAGGAGTTCTTGGTGTTGCAGGTGGAGGATCATGTGTCGCCCGGAAGGGACGAAATTCTTGGCCGCCTTGTCGTCCCAGTGTCCAACATTGAGAGACGCTGGGATGAGAAGCTTGTTGTCTCCAGGTGGTATGGGCTGGACCGTGGCACCGGCGGCGGCAACGTGGCCATCAACAATCCAAACAGGTTTGGCAGCCGTGTTCATCTCCGGTTGAGTCTCGATGGTGGCTACCATGTCCTGGATGAGGCGACAGCATATAGCAGTGATCTCCGGCCCACGGGGAAGCAGCTGTGGCAGCCGCATGTTGGGGTTCTTGAGCTCGGCGTGCTTGGCGCCACTGGACTGATACCAATGAAGGCTCGTGATGGCAGGGGTGCCACGGCAGATTCGTATTGTGTTGCAAAGTATGGCCAGAAGTGGATCCGCACTCGCACCGTCGTGGACTCTGTTTGCCCGCGGTGGAACGAGCAGTACACCTGGGAGGTGTTCGACCCTTGCACTGTCATCACCATCGGTGTGTTCGACAACTGCCATGTTGACAAGCCACAATCAGGGAACACATCTGTGGTTGTCCGAGATAATTGCGTTGGCAAAGTCCGGATCAGGCTCTCGACATTGGAGACCGACAGGGTGTACACCCATGCATACCCACTCCTTATGCTGCATCCGTCGGGGGTCAAGAAGATGGGGGAGCTCCACCTTGCTGTGCGCTTCTGCTGTGGCAATGCCGGCAACATGTACCATGCCTATGTACGCCCACTGCTTCCGAAGATGCACTATGTTGAGCCGCTGCTCGTGCGCCAGGTTGAGAGCCTTCGGTTTCAGGCGACGAGTGTCGTTGCTGCCCGGCTGGGCCGTACCGAGCCACCGCTTGGCAAAGAGGTTGTGGAGTATATGCTGGATCACCGCTCACACCTATGGAGTATGCGACGCAGCAAAGCAAATTTCTTCCGCCTTGTCGCTGTGCTCTCTGGTCTGATTGCCATTGGCAAGTGGTTTGAACTTGTTCGCTCCTGGCATCGCCCGGTGCATTCATGCCTTGCTGTTTTCACATTCCTGGTGTTCGTCCTGATGCCAGAACTGATCCTTCCAACGGCCTTCCTGGTGATGGCCTTCACTGGGCTATGGAGGTACCGGGTCCGTCCGAGGCACCCACCTCACATGGATATGCGGCTATCTCATGCAGATGCAGCCACCGTGGACGAGCTTGATGAGGAATTCGACACTTTCCCTTCAAGCCGGGGGGATGTCGTGCGCTTCCGGTATGAACGCCTCCGCAGCGTGGCTGGGAGGGTGCAGACAGTGGTGGGTGACATAGCGACGCAAGGTGAGCGGATGCAGGCCGTACTCAGCTGGCGTGACCCAAGGGCGACGCTCCTGTTCTCCATTGCCTGTGTCACAGCGGCGGTCATTGCATACGCCGTGCCGATGAAGGTGCTGATCGGGCTGTGGGGCCTGTACGCTATGCGCCCACCGAGGTTCCGCAGCAGGATGCCGTCCCCGCTGATGAACTTCTTCAGGAGGCTTCCTTCCAAGGCCGACATCCTGCTCTGA

>Bradi4g27090

ATGCAGCGTCCGCGGTCAGAGGAATACTTATTGAAAGATACATCGCCTCACCTTGGGGGTTTCATGGCAGCAGGTGACAAGCTCACAAGAACATATGACCTTGTGGAGCAGATGCAATATCTCTATGTGCATGTTGTCAAGGCCAAAGACCTCCCTTTCAAAGATCTGACTGGGAGCTGTGATCCATACGTGGAGATTAAGCTTGGCAATTACAAGGGCATCACCCACCACATGGAGAAGAATACTAGCCCAGAGTGGAACCAGGTATTTGCCTTTCCCAAGGAACACATACAGTCCCCCTATGTTGAGGTCGTTGTCAAAGACAAGGACTTATTCATTCAGGACGATTTCATTGGGCGAGCTGTGTTTGACCTCAGTGAAATTCCAAAGAGGGTCTCACCTGACAGTCCACTAGCACCAGAGTGGTATAGTCTTGAGGGTTGGAATGGGGGAAAGTTCGGCGAGCTCATGTTGGCTGTCTGGATGGGTACACAAGCAGATGAAGCTTTCCTAGAAGCATGGCATTCTGATGCTGCAACAGTCCCTAGTGATGGTCTAGCAAGCATAAGGTCTAAGGTGTATTTGAATCCTAAGCTTTGGTATCTCAGGGTCAATGTTATTGAGGCGCAAGATTTGGTACTAAGTGACAAGAGCCGTTGTCCAGAAGTTTATGTGAAAGCTACACTTGGGAGCCAGTCATTGAGGACAAAGGTATCCCCAAACAAGAATGTTAATCCCTTGTGGAATGAGGATCTGATGTTTGTGGCCGCTGAACCATTTGAGGAACACTTGATTCTTAGCGTTGAGGATTGGATTGCACACAACAAGGATGAAATATTAGGGAAAGCAATTATTCCACTGCAGAATGTAGATAGGAGGCTCGACCACAGGCCAGTGGTCAGCAGGTGGTGTAATCTTGAGAAGCATGTAACTGGGGATGGAGAAAAAAAGAAAAAGGATTTCAAGTTCTCTAGTCGTATTCATCTTAGGATTTCTTTGGATGGTGGGTATCATGTTCTAGATGAGTCATCATACAACAGTAGCGATCTGAGGGCCACTGCAAAACAATTGTGGAAGCATTGGGATTCGTATTGTGTTGCGAAGTATGGACACAAGTGGGTTAGAACAAGGACTATTATTGACAGTTTCAACCCAAAATGGAATGAGCAATACACTTGGGATGTTTATGATCCATGTACTGTTATAACAATTGGTGTGTTCGATAACTGTCACTTCCAAGGTGAGAAGGCCAAGGGAAACAAGGATGGCAGAATTGGAAGGAAGATGGGTGAAGTGCAGCTTGCTGTGCGGTTCACCTGCTTCTCACTTGTGAACATGATGCAGCTGTACTCTCAGCCACTACTGCCAAACATGCATTATATTTACCCTCTATCAGTCCCACAGTTTAACAATCTGAGGTTCCAGGCTACTCAGATGGTCTCGATGAGATTAAGCCGTGCTGAGCCACCTCTCAGGAACGAAGTTGTTGAATACATGCTGGATCTGGATTCTCACATGTGGAGTATGAGAAAGAGCAAGGCAAACTTCTTTAGGATTGTGAATATATTGAGCCCCCTGGTTGCAGCTTGTAAATGGTTTGATCAGATTTGCACATGGAAGAATCCTCTGACTACTGTGCTGATTCATGTTCTATTCATGATATTGGTGGTGTATCCGGAGCTGATCCTTCCAACAATTTTCATGTATTTGTTCTTGATTGGGATCTGGTACTACCGTTGGAAGCCAACGCAGCCACCTCATGTGGACATCCATCTTTCACATGCAGAGACTTCCGAACCAGATGAGACTGATGAAGAGCTTGATACTTTCCCATCTCGCACACCTGATGTTGTTAAGATGAGGTATGATCAACTGAGGAGTATCTCTGGCAAGGTACAAACAATTATTGGTGACATGGCAACACAAGGTGAGAGGCTGCAGTCCCTGCTGAGCTGGCAGGATCCAAGAGCAACTGCCATCTTCATGACTTTCTGCTTGATCGCTGCTGTCGTGTTGTACCTGACGTCATTCCGCATAGTTGCATTCTTTGCTGGGTTATATCTGTTGAGGCATCCTAGGTTCCGTTACAGGCTTCCATCTGCTCCAGTCAACTTCTTCAGGAGGCTCCCTACAAGAACAGACAGCATGATGTGA

>Bradi4g40830

ATGGTGAGCTATACACTGGTTGTTGATGTTGTAAGTGCTAATGGTCTTTCAGGCAGTCATGATTCTCTGAATCTCTGTGTTGAGCTTCGGTTTGCTGGGCAAAGAGCCACGACGTCAGTCAAGAACAAAGATTGCCGTCCTGTATGGAATGAGACGTTCCGATTCTCTGCACTGGACAAAGATAAGGTTGGTTATGGCACTCTTGAAGCTTATGTGTATAATATTGTCACTGCTGGCCGCAAGTCTTTACTGGGCAGGGTCCGGCTATCTGGGTCTGTGGTTCCGGACTCCTCCGCTGATGTTGCTGCTGGCCCCTATCCATTGAGGGGTGGGATATTCCCACGATCGAAAGGAACATTGCATCTCAAAGTTGTACTTGAGAACGAAACCCCCATTGCAACCTCCGATCCTCTCTTAGCAGTTATTCCTTCCAGTTTTTTCACAATAGGAAACCGGGAGTGTGCGACGCCTGTGGATGCTGCTGTCAAGGAAATCACGCCAAGCTTTCAACACGGGATGATTGTTGAGCTTATGCCCTATGTTTTTGTGCATGTGGTGAAGGCTCGACACTTGGCAGGTGCCGATGCCAGAGGAAGACTTGACCGCTATGTTGAAGTAAAGGTTGGCGATTACGGAGGCACCACCGAGTACATGGACATGGAGCAGAATGCTGAGTGGAACGCAACTTTTGCATTCTCAAAACTAGAGATGGACCAGAACCAGCTGGCAATGGTCTATGTTATCGTAAAGAATACGGACATGGCAAGGGATGACTCTGTAGGAATGGTGTGGTTTGACGTAAACAACATTCCTAGGCGTACCCCCCAATCTCATGAACCATTACTACCAGAGTGGTATCCTCTTCGTGATGAAAGCGGGACTAGTACTGAAGGTGAGTTGCTGCTTAAAGTTTGGAGAGGATCCCAGGCTGATGAAGCATTTCCTGACGCTTTCAAGACGGATTCTCGCATTGGGCCCCAAGTGTATCATTTGCCACGCCTCTGGTACCTGAGGATCCAGATAATTGAGTTTAAATGTGTTGCAGTAGCAGGTAGGGCAAAGGTTGTGGAGTTGGATGTCACGATAGCGCATGGTGTTCAGCATCGGATTACGAAAAAAGTAAAGAAGCCGCTAGGCCACCATGTCTGGAACCAGGAATTTATGTTAGTAGTTGCGGAGCCATTTGAGGATGGCGTTCAAATTTCTGTCCGAGCACATGTTGGGCCTCGTAGCAGGCACGTGATTATGGGCGAAGTCACTATACCCCTTGAAACATGTCAGAGGCAAGTCGAGGGCCGTCATATCAAATCTCAGTGGTTTGATCTTCAAATGCCAAGACAAGCACATGATGTTCATGGTGGTAGGTCCAGGGATGATGAATTTGCTGCCTCATCATGCCACATTCGTCTCACCAGCTGCCTGGAGGGTGGATACCATGTCCTATATGATAGTACGTACTTTGTTGATGATTACAGGCCTTCAGCTATGGAAATCCCGGATCCTCCTACCGTTGGCTTGCTTGAGATTGGAATCCTCGGAGCGAAGGGCCTCCATCCCAGGAAAAGGATAAATGGATCCTCGACACTACACCCATATTGTGTTGCAAAATATGGCAGGAGGTGGATACGAACTCGTACCATCAACAACAGTTGCAATCCTGTATTCAATGAGCAGTACAACTGGGATGTCTATGACACATCAGCCGTCTTGACCATCGGTGTTTTCGATAATGCCCAACTCCAGGGGTATAGCTCAGAAGAAGACAAGTCTGTCAAGATAGGGAAAGTAAGAATTCGCCTCTCCGATCTCCAACCTGGTCGAACATATGCTCACTCATACCCTCTACTTGTTCTACGTCCTAAGGGGCTCAAGAACATGGGCGAACTGCACCTGGCAGTAAGATTTTCAGGAGAGTCAATACTGAAAATGGTGCGCATGTACTCTAATCCCAAGCTGCCAGAGATGCATTACAAGCACCCAATTTCAGTAATGCAGCTAGATTATTTGCGGCACCACGCTCTCGGGATTGTGGCTGCTAGATTCAGCCGGATGGAGCCACCTCTATGGAAGGAAGCTGTGGAGTACATGTGTGATGTGAGCGGACACATGTGGAGCTTGCGCAAGAGCAAGGCTAACTTCTATAGGATAATGGGCGCCTTCTCATTCTTTTTTAGGTTTATCAAATGGTTTCACGGTGTATGCTTATGGAAGAACCCTGCCACAACTCTGCTTGTCCACGCCATCTTTGCGATGCTTGTTTTGTATCCACAGCTCATACTCCCAGCGGTGCTCCTATATGTCTTCTTCATTACCGTGCGGAACTACCGGCATCGCCCTACTTATCCTCCACATGTTGATACGAAGTTATCTTATTCAGAGGGAGCACATCCAGACGAGCTTGATGAGGAATTTGATACATTCCCAACCTCGCGGAGTCTGGATTTAGTAAGGATGAGGTATGACAGACTAAGGAGTATTGCCGGCAGGGTACAGACAGTCATAGGAGATGTAGCAACGCAGATTGAGAGGATACAAGCATTGGCAAGCTGGAGGGATACTACAGCGACGGCAATATTTGGTCTCTTCACTCTTGTGGCTGCTATTGTCATATTTTTTACACCATGGAGAGTTCTCGTTGCAATAGCCGGATTGTACACCATGAGGCCCCCCATGCTTCGGCGCTACAGTGTGATGCCGTCATTTTTCGCGAACTTCTTCCTGCGTTTGCCGCAGAAGACAGACAGCCTGCAGTAA

>Bradi5g13230

ATGGGGATCCTGGGCCCTGAGAACCACGTTGTTGCAGCCGCCACCTCCCCTAAAGTTGCTATCAAGACCACAATGCTCAATTTTGATGAGAGAACGCCGGCGCCGGCAGTGAGAGCGGAGGAATACAAGGCCAAGGACGCGATGCCGCAGCAACAGGCGAGGGCTCAGTGGCCGGCCGGCAGCGGCGGCAGCGGCAGCACGAGCGCGCGTGGCGAGTGGATGGGCATTGGCTCCGGCGAGAAGCTTGCGAGCGCTTATGACCTCGTGGAGCGGATGCATTACCTGTACGTGCGCGTCGTCAAGGCGCGCGGGGTCCCCGTGGCCGTGGGCAGCCCCGGCGTCGTCGAGGTCCGCCTGGGAAACTACCGGGCCACGACGCCTCACCGCGAAGGGATCCATGAGTGGAACCAGGTGTTCGCCTTCTCCAGGGAGCGCGTCCAGGCCTCGGTGCTCGAGGTGTTCGTCCGGGACAAGGACGCCGCCCTGGCCTCGGCGCCAGACTACTACATCGGCAAGGTCGCGTTCGACGTCGCCGAGATCCCCGTGCGCGTGCCGCCCGACAGCCCGCTCGCGCCGCAGTGGTACCGCCTCGGGAACGCCGGTGGCAACGGCAAGATGGCGCACGTCGAGGCCATGCTCGCGGTGTGGGTCGGCACGCAGGCCGACGAGGCGTTCGCCGACGCATGGCATGCCGACGCGGCGTCGGTGCGCGGCGGCGATGGCATGGCCGTGCAGCAGAGCACGCGGTCCAAGGTGTACGTGACGCCCAAGCTGTGGTACCTCCGGATAAACGTGCTGGAGGCACAGGACGTCGTGACCACTGCCCGTGTCGGCGCCGGCAGCAGGCACGTCGAGGTCTTCGCCAAGGTGCAAGTAGGCGGCATGACGCTCCGGACGAAGCCGTGCTCCGTGAGGAGCGCCACGAGCCTGTCCTGGAACGAGGAGCTGGTCTTCGTGGTGGCGGAGCCGTTCGAGGACCCGGCGGTGCTCATCGTCGAGGCCCGGGCGCACCCTGGCAACAACAACAACAAGGACGAGATCGTGGGCCGCGCCGTGCTGCCTCTCACGATCTTCGAGAAGCGGCTCGACCGCCGGACGGTCCACTCGCAGTGGTTCAGCCTGGAGCCGTTCGGGCATCCGCTGACGTTCGCCGGCCGCGTCCACATCCGGGCGTGCCTGGAGGGCGCGTACCACGTCATGGACGAGCCGGCCATGTACGCCAGCGACACGCGGCCCACGGCGCGGCAGCTGTGGCGCCCGCCCGTCGGCGTGCTCGAGGTCGGCGTCCTCGGCGCGCAGGGCCTGACCCCCATGAAGACCACCGACGGCCACGGCGGCAGGGGCAGCACCGACGCGTACTGCGTGGCCAAGTACGGGCACAAGTGGGTGCGCACGCGGACCGTGGTGGACTCGTGCAGCCCGCGGTGGAACGAGCAGTACACCTGGGAGGTCTACGACCCGTGCACCGTCCTCACGCTCGCCATGTTCGACAACTGCCACCTCGGCAACGCCCCCGGCGCCGTCACCAGAGACCAGAGGATGGGCAAGGTGAGGATACGGCTGTCGACGCTGGAAATGGACAAGGTGCACGCCAACGCGCACCCGCTGCTCGTGCTGCACCCATCTTCCGGCGCGCTGCGCAAGACCGGCGACCTCTGCCTCGCCGTGCGCCTCACGTCAGTCTCCCTCGCCAGCGTCGCGTGCCTCTACGCGCAGCCGCTCCTCCCCAAGATGCACTACCTCCAGCCCTTCACCATCCCTCAGCTCGACGCGCTCCGGCGGCAGGCCATGGGCCTCGTGGCGGCGCGGCTGGGCCGGGCCGAGCCGCCGCTGCGCAGGGAGGTCGTGGAGCACGTGCTGGAGGCCGGCTCGCACGCGTGGAGCATGCGGCGGAGCAAGGTCAACTTCTTCCGGGTCACGGCCCTCCTGTCTGGGGCCGCCAGCACGGCGCGGTGGCTCCTGGACGTGTGCCACTGGAGACGCCCGGCCACGACCGTCCTCGTGCACGTGCTCTTCGTCACGCTCACGTGCTTCCCGGAGCTCGTCCTGCCAACCGCGTTCGCGTACATGGGCTTGGCGGGCCTCTGGAACTACCGGCGCAGGCCGCGGCGCCCGGCCAACATGGACGCGAGGCTTTCCTGCGCGGACACGGCCCAACCCGAGGATGTCGACGAGGAGATGGACACGTTTCCCACGTCGAAGCCCAACGGCGTGGTGCGGCTGCGGTACGACCGGCTGCGGAGCGTGGCCGGGAGGATCCAGACGGTGGTCGGGGACGTGGCCACGCAGGGGGAGCGCGTGCGGTCGCTGCTCGCCTGGAGGGACCCGAGGGCCACGGCGATGTTCACGGCGCTCTGCCTCGTGGCAGCCGTGGCGCTTTATGTCACGTCGTTCCGGGTCGTCGTACTCGTCGCCGGGCTCTACGTGCTCCGCCACCCGCGGTTCCGGAGCCAGATGCCCTCCGCCGCTGCCAACTTCTTCAAGAGGATGCCCTCCCGGGCCGACACCATGCTGTAG

>Bradi5g26730

ATGGCGACGTATAAGCTGGGTGTGGAGGTTGCGAGCGCTCATGACCTGATGCCCAAGGACGGGCATGGTTCTGCGAGCGCCTGTGTCGAGCTCAACTTTGATGGCCAGCGGTTCCGGACAGCTATCAAGGAGAAGGATCTGAACCCGGTGTGGAACGAGCACTTCTACTTCAACGTGTCAGATCCATCAAATCTCCCTGAGCTTGCTCTTGAGGCGTATGTCTACAATGTCAACAAATCCGTTGAAAGTTCCAGGTCATTCCTTGGCAAGGTCAGGATTGCTGGGACCTCATTCGTGCCCTTCCCTGATGCCGTCATCATGCATTATCCCCTGGAGAAGCGCGGGATGTTCTCACGGGTGAGAGGAGAACTGGGTCTGAAAGTGTACATCACCAACGACCCCTCTATCAGAGCTTCAAACCCTCTTCCGGCAATGGACCCTGTTTCGAATCATTCTCCAAGTCAAGCTGAGCAAATAGCAGCTGATATAACTGGTACTAATCTGAACACCTCTCGGGAACACAGGAATGAAGCGAGAACCTTGCATACCATAGCTAAGGACGCACATCATCATCAGCACCATGGCCATCTTCCAGCTTCTTTTTCCGAGCAACCTTCCAAGTATGGTATTGAGCAAATGAAACCTCAACCTCAACAGCCCAAGATTGTCAGGATGTATTCAGCAGCCTCACAGCAGCCCATGGACTACGCCCTTAAAGAAACTAGTCCATTTCTTGGTGGTGGACAGATTGTTGGTGGTCGGGTCATACGTGGTGAGAAGCATGCTAGTACCTACGACCTGGTGGAGAGAATGCAGTATCTGTTTGTACGTGTGGTCAAGGCACGGGACTTGCCTGACATGGACATCACTGGTAGCCTGGATCCTTTTGTGGAAGTGAGAGTTGGCAACTACAGGGGCATAACTAAGCACTTTGAGAAGCAACGGAATCCTGAGTGGAATGCAGTATTTGCTTTTGCTAGAGATCGTATGCAGGCATCTGTCCTTGAAGTGTTGGTCAAAGATAAAGATCTTGTTAAGGATGATTTTGTTGGTATGGTCAGGTTCGATTTGAATGATGTACCAATACGTGTGCCTCCTGATAGTCCGCTAGCTCCAGAATGGTATCGGCTTGTTCATAAGAGTGGGGATAAGTCAAGGGGTGAGCTGATGCTGGCAGTTTGGGTTGGCACCCAAGCTGATGAGGCATTTCCTGACGCATGGCATTCGGATGCCGCAACACTTGATGACGCATCTGCTGTAACACACATGAAGTCGAAAGTTTACCATGCTCCCAGATTGTGGTACCTGCGAGTTAATATAATTGAGGCCCAAGATATTCTCATACATGATAAGACCCGCTATCCAGATGTTTTTGTGAGGGCACAGGTGGGGCATCAGCATGGGAGGACAAAACCTGTTCAAGCTAGAAACTTCAACCCATTTTGGAATGAAGACCTTATGTTTGTGGCTGCTGAACCTTTTGAGGATCACCTTATCCTCACGCTTGAAGATCGTGTAGGTCCTAACAAAGATGAGATGCTTGGCCGCATAATTATACCATTGACGATGGTTGAAAGACGGGCTGATGACCGTATTGTCCATGGGAAGTGGTTTAATCTCGAGAAGCCTGTACTTGTTGATGTGGACCAACTGAAGAAGGAGAAGTTCTCTAGTCGGCTTCATCTCCGTCTCTGTCTTGATGGAGGGTACCATGTTCTGGACGAGTCCACAAACTACAGCAGTGACCTCAGACCAACAGCCAAGCAACTCTGGAAGCCATCGATTGGTTTGCTCGAGCTTGGAGTCCTTGGTGCTCAAGGGATTGTCCCTATGAAGACTCGAGATGGAAAAGGTTCATCAGACACCTATTGTGTTGCGAAGTATGGGTCAAAGTGGATTCGTACACGTACTATCATGAACAATCCAAACCCCAAATTCAATGAACAATACACTTGGGAGGTCTATGATCCGGCAACTGTCTTGACTATCGGTGCTTTTGACAATGGCCAGCTTGGTGACAAGAATGGGGAGAAGACGTCCAATGGTAAAGATGCGAAAATCGGCAAGGTTCGAATTCGCCTTTCCACACTTGAAACTGGCCGTGTCTACACTCACTCCTATCCTCTCCTGGTTCTACACCCATCAGGGGTGAAAAAGATGGGTGAGCTGCACCTAGCCATACGGTTTTCCTCAACGTCATTGGTGAACATGCTGTACCTGTACTCTCGACCTTTGCTACCGAAGATGCACTATGCACGTCCGATACCAGTCCATCAGGTTGACATGCTGCGCCATCAAGCTGTCCAGATCGTGGCTGCCCGACTTAGCCGAATGGAACCACCTCTGAGAAAGGAAGTTGTTGAGTACATGTCAGATTTTGATTCTCACTTGTGGAGCATGAGGCGAAGCAAAGCAAACTTCTTCAGACTCATGTCAGTCTTCTCAGGCTTGTTTGCGGTCAGCAAGTGGTTTAGTGGTGTCTGTGCATGGAAGAACCCTATTACCACTGTGCTAGTCCACATCCTCTTTATAATGCTGGTGTGTTTTCCAGAGCTCATACTTCCAACAGTGTTCTTGTACATGTTCCTGATAGGGATTTGGAACTATCGTTATCGGCCTCGCTATCCCCCACACATGAACACCAAGATCTCTCATGCAGAGGCTGTTCACCCAGATGAACTTGATGAAGAATTCGATACTTTCCCGACAAGCCGGAGCCAGGAGATTGTAAGGATGAGGTATGATAGGCTGAGGAGTGTTGCTGGAAGGATACAGACTGTTGTTGGTGATATAGCAACACAAGGGGAGAGAGTTCAAGCACTGCTTAGTTGGAGGGATCCTCGGGCTACAGCAATATTTGTCCTATTTTGTTTCACTGCAGCAATAGTCCTGTATGTTACACCACTCCAAGTTCTTGCAGCATTAGGAGGGTTCTATGCCATGAGGCACCCAAGGTTCAGACACAGGCTGCCCTCAATACCAGTAAACTTCTTCAGACGCATGCCAGCAAGGACCGACAGTATGCTATAA

>Bradi5g27530

ATGAAGCTGGCGGTGGAGATCGCCGACGCGGCGGACCTGTCGCCCAAGGACGGCTCCGCCACCTGCAACGCCTTCGTCGAGGTCGACTTCGACGGCCAGAAGCAGCGCACCGCCACCAAGCCCGCCGACTGCGCGCCCCAGTGGAACCAGACGCTCGTCTTCTCCGTCGCCGACGCTTCCCTCTTCCCGTCGCTCCACGTCGAGGTGTCCGTGTACCACGACCGCCGCCTCAACGACCACAACGCGCTCCGCCCCCACGCCTTCCTCGGCCGCGTCCGCCTCTCCGCCGCCGCCTCTGTTGCCAGGTCCGTCGGCGAGGCAGTGCTGCAGCGGTACCCGCTTGATAAGAGGGGGCTGTTCTCGAGGGTCTCCGGCGACATCGCCCTCCGCCTCTACCTCATCAACGAGGACGGCGATCCTGCTGCTGCTGCTTCGGGGGCGGCCGTGGATCAGCCTTCTGAGCCGGTCGCCATGGACCCTGAGAGGACTGTCAGGAACGTCTTTGCCAACGAGGCGCCTTCTTCTTCTTCCTCAGCTCCGGAGGCAGCGGCGGCGGCGGAATCCAAGGGGAAGAGCAGCCACGACCACGAGCTTCCTCCGCCGCGCGAGTTCCGCGCCGAGCCACGCCGCTTCACGCTGCACGCCATGGCGGCGCCGAGCGCGCCCCCGGGCCAAACAGTCGTCATGCCGAAGCCCCCCGCCGCGGCGGCGCAGCAAGCGGCGGCGCCAGGCTCCCAGTACGGGCTAGTGGAAACAAAGCCACCCCTCCCGGCAAAGCTCGGCCCGCGCGGGTCGGCGCTGGCGGCGTCCAAGGTGTCGTCCACCTACGACCTCGTAGAGCCCATGTCGTACCTCTACGTGACCGTCGTGAAAGCCCGGGACCTCCCGACCAAGGACATCACCGGAGCGCTGGACCCCTACGTGGAAGTAAAGCTCGGCAACTTCAAGGGCACCACAAAGCACCTGGAGAAGAACCCGAACCCGGTGTGGCGGCAGACGTTCGCCTTCTCCAAGGAGCACCTCCAGGCCAACCAGCTGGAGGTCATCGTCAAGGACAAGGACGTCGTCAAGGACGACTTCGTCGGCCGCGTGCTCTTCGACATGTCCGACGTCCCCAGCCGCCTCCCCCCCGACAGCCCGCTCGCGCCGCAGTGGTACAAGCTCGCCGAGGCCGGGGGAGACAAGCTGCGGCACGGCGGCGAGATCATGCTCGCCGTCTGGCTCGGCACCCAGGCCGACGAATCGTTCCCGGAGGCCTGGCATTCCGACGCGCATGGCGTGGCGTCGCAGGAAGGGCTCGCCAGCACGCGCTCCAAGGTCTACTATTCCCCCAAGCTGATTTACTTGAAGGTGAACGTCATCGCCGCCCAGGATTTGGTGCCTGGGGAGAAGGGCCGGGCAATGGCGCCGGCGATCGCGAAGATCCACATGGGGAGCCAGATCCGGCGGACGAGGCCGCAGCAGTCGGCGAACCCTGGGTGGAACGAGGAGTTCTTCTTCGTGGCCGGCGAGCCCTTCGAGGACCCGCTCGTGGTCACCGTGGAGGAGAAGCTCTCCGGCCGGGACGAAGCCATCGGCCGCGTCATCATCCCCGTCGGCGCGCCGTTCGTGGCCCGCAACGACCTCGCGAAGTCGATCGCGTCGCGGTGGTTCAGCCTGTCCCGGGGGATGACGGTGGACGAGGCGTCGGCGGGGGTCACGGAGAAGATGAAGGACAGGGAATCCTCCAAGACGTTCACCAGCAAGATCCACCTGAGGCTCAGCCTCGAGACCGCCTACCACGTGCTCGACGAATCGACACACTACAGCAGCGACCTGCAGCCCGCGGCGAAGAAGCTCCGGAAGAGCGCCATCGGCATCCTGGAGGTGGGCATCCTGAGCGCCAAGAACCTGGCGGGGAAGAAGAACCCCTACTGCGTGGCCAAGTACGGCGCCAAGTGGGTCCGCACGCGGACGCTGGTGGGCACGGCGGCGCCGGCGTGGAACGAGCAGTACACCTGGGAGGTGTTCGACCTCTGCACCGTCGTCACCGTCGCCTGCTTCGACAACGCCGCCGTCCATGGCGGCGACAAGGACGCGCGGATCGGCAAGGTGAGGGTCCGCATATCGACATTGGAGTCGGACCGCGTGTACACCCACTACTACCCGTTAATGGCGCTGACCCCGTCGGGGCTGAAGAAGACGGGCGAGCTCCACCTCGCCGTGCGCTACACGTGCACGTCCTGGGCAAACATGCTGGGGCAGTACGGCAAGCCGCTGCTCCCCAAGATGCACTACACGAACCCCATACCCGTGCTCCAGCTCGACTACCTCCGGTTCATGGCGATGCAGCTGGTGGCGGCGCGGCTGGGGCGCTCGGAGCCGCCGCTGAAGAGAGAGGTGGTGGAGTACATGCTGGACGTGGACTCGCACATGTTCAGCCTGCGGCGGAGCAAGGCCAACTTCCACCGGATCACGTCCCTCTTCTCCGGCGCCGTCGCCGTGGGGAAGTGGTTCGAGGGGATCTGCAAGTGGAAGAACCCGCTGACGACGATCCTCGTCCACGTGCTGTTCCTGATCCTAGTGTGCTACCCGGAGCTCATCCTGCCGACGGTCTTCTTATATTTGTTCATGATCGGGGCGTGGAACTACCGGCGGCGGCCGAGGAAGCCGCCGCACATGGACACGGTGCTGTCGTACGCCGAGCTCGCGCACCCCGACGAGCTGGACGAGGAGTTCGACACGTTCCCGACGTCCAAGCCGGGGGACGTCGTCAGGATGAGGTATGACAGGCTGAGGAGCGTCGCCGGCAGGGTGCAGACGGTGGTCGGGGACTTGGCGATGCAAGGCGAACGCGCCCAGTCGCTGCTCAGCTGGAGAGACCCCAGGGCGACGTCCATCTTCGTCACGCTCTCGCTCATCGTGGCCATTGTGCTCTACGTGACGCCGTTCCAGGTCGTGGCAGTCATTGCTGGGCTTTACCTCCTCCGGCATCCAAAGTTCAGGGGCAAGCAGCCCTCCGTGCCCTTCAACTTCTACAAGCGCCTTCCCGCCAGGGGCGACATGCTCATATGA

>Cicle10000127

ATGGCCAGAGTGGTAGTAGAAGTTGTTGATGCAAGTGACCTCACACCCAACCATGGACAAGGATCTGCTAGTCCCTTTGTTGAAGTAGACTTGGATGATCAGAAACAAAGAACTCAAACCAAGCCTAAAGACGTCAATCCTTACTGGAATGAAAAGCTTGCTTTCAACATTAACGATCTCAGAGATCTTCCCAACAAGACCATTGATGTAACTGTTTTCAATGATCTCAAAGGCAGCCACGATCGTGATCACCACAAGAACTTTCTCGGACGTGTCAGAATTTCAGGTGTTTCTGTCCCTTTTTCGGAGTCAGAAGCCAATGTTCAGCGATACCCTCTTGACAAACGCGGCCTTTTCTCTCGTGTCAATGGTGATATTGCCCTCAAGATATACGCACACCCACTTCATGATGCCTCTCACTTTACAACTCCACCAACCAACGCTACTACCACTGCCAGTAGTCTTGAGACCGAAGAAACCCCACTCCAAGAAATCAACACCAACAAGTTTGGCGATCATGATGTTAAATTAATGTTTGATCACGAGAAGATCAAAAAGAAAAAGAAGGAAAAAGAAGTAAGGACTTTTCACTCAATAGGGACGGCAGCTGGGGGTCCTGGTCCGGCTCCTGCTGCTCCGCCACCGGTGTCATCCACGTTTGGTTTTGAGACTCATCAAAAGCCTCCAGTGGCTGAGACGAGAATGGATTTTGCCAAAGCTGCTGCGCCAACACCATCTGTAATGCAAATGCAAATGCCAAAGACGAACCCAGAATTCCTTCTGGTGGAGACTAGTCCGCCGGTAGCAGCGCGTCTGCGATACAGAGGAGGAGACAAGACAGCAAGTACTTACGATTTGGTGGAGCTGATGCATTATTTATACGTGGATGTGGTTAAGGCAAGAAATCTGCCTGTAATGGATGTTTCAGGGAGCCTTGACCCTTACGTTGAAGTGAAGCTTGGAAACTACAAAGGCATCACCAAACACTTGGAGAAGAACCAAAACCCGGTATGGCATCAAATTTTTGCCTTCTCTAAGGAAAGATTGCAATCCAATTTGGTTGAAGTTACTGTCAAAGACAAGGATATTGGGAAAGATGATTTTGTGGGGAGAGTTACTTTTGATCTATTCGAAGTCCCTCATCGTGTTCCGCCTGATAGTCCATTGGCTCCTCAATGGTATAGATTGGAGGACAGAAAGGGTGATAAAATTACTAAAGGGGAAATAATGCTTGCTGTTTGGATCGGGACACAAGCTGATGAGTCTTTTGCTGCGGCCTGGCACTCTGATGCACACAACATCAGCCAGAAAAATTTAGCTAACACCAGATCAAAGGTTTACTTCTCACCCAAGCTTTATTATCTTCGGGTTTTTGTATTTGAAGCACAAGATCTTGTTCCTTCTGACAAAGGGCGAGCACCAGATGCTTGTGTAAGGATTCAGCTTGGAAATCAGCTGAGGGTTACCAGGCCTTCGCCTGTGCGAACTGTTAACCCGGTATGGAACGAAGAGCATATGCTTGTTGCATCTGAACCTTTTGAGGATCTGATTATTGTGACTGTTGAAGATAGGATTGGACCTGGCAAGGATGAGATTTTAGGAAGAGAATTCATACCAGTTAGAAATGTGCCACATAGACATGAGACCGGTAAGCTCCCAGATCCTCGATGGTTTAACCTTCACAAACCTTCGTTGGCAGCAGAGGAAGGTGCTGAAAAGAAGAAGGAGAAATTCTCAAGCAAGATTCTAATTCGCTTCTGTTTGGAAGCAGGTTACCATGTTCTTGATGAATCCACACATTTTAGCAGTGACCTACAGCCCTCGGCGAGGAGCCTGAGGAAGGATAGCATTGGGATTCTTGAGCTTGGCATTTTGAGTGCAAAGAAGTTGATGCCAATGAAGAGCAAGGATGGTAAACTGACTGATGCATATTGCGTGGCCAAGTACGGGAACAAATGGATTCGAACCAGAACAATCCTTGACACACTGGATCCTAGATGGAATGAGCAGTATACTTGGGATGTTTATGATCCATGTACTGTAATCACAATTGGAGTATTTGACAATTGCCACGTCAATGGAAGCAAAGATGACGCGATTGATCAGAGAATCGGAAAGGTGAGAATTCGATTATCAACTTTAGAAACCGATCGTATATACACCCATTTCTATCCCTTGTTGGTACTCACGCCTTCTGGTTTGAAGAAAAATGGGGAACTTCATTTGGCTTTAAGGTTTACATGTACAGCTTGGGTAAACATGATGACAAAATATGGAAGGCCGTTGCTTCCCAAAATGCATTATGTCCAACCAATACCTGTTATACTCATTGATAGGCTACGGCATCAAGCAATGCAGATAGTTGCGGCAAGGCTGGGAAGAGCAGAGCCACCCCTGAGGCGCGAGGTAGTGGAGTATATGCTGGATGTGGATTACCATATGTGGAGTTTGAGGAAAAGCAAAGCCAATTTTTACAGAATTATGGAACTTCTTTCGGGGCTTACAGCTATTTGTAGATGGTTCAATAACATATGTACATGGAGAAACCCAGTGACAACAATCCTTGTACATGTATTGTTCTTGATACTCGTTTGCTACCCTGAGTTGATACTGCCAACAATTTTCCTGTACCTCTTCGTAATTGGGATGTGGAACTATCGGTTCAGGCCAAGGCACCCGCCTCACATGGATGCTAAGCTCTCGCAAGCAATAAATGCACACCCAGATGAGCTAGACGAAGAATTTGATTCGTTTCCAACAAAACGACCATCAGACATCATTAGGATGAGATATGACAGGCTACGCAGTGTGGGAGGGCGGGTGCAGACAGTGGTTGGAGATTTGGCATCGCAAGGCGAAAGAGCTCAAGCGATCCTCAACTGGCGTGATCCAAGAGCTACGTCTATCTTCATCATCTTCGCATTGATTTGGGCAGTGTTCATTTATGTTACTCCATTCCAAGTGGTTGCAGTTCTGATTGGCCTTTACATGCTGCGCCATCCCCGGTTCAGGAGCAAGATGCCTTCGGTACCTGTCAACTTCTTCAAAAGATTACCGGCCAAGTCAGATATGCTGATATAA

>Cicle10000346

ATGCCGAAGACGAACCCTGAATTCCTTCTGGTGGAGACTAGTCCGCCGTTAGCGGCGCGTCTGCGATACAGAGGAGGAGACAAGACAGCAAGTACTTACGATTTGGTGGAGCTGATGCATTATTTATGCGTGAATGTGGTTAAGGCAAGAAATCTTCCTGTAATGGATGTTTCAGGGAGCCTTGACCCTTACGTTGAAGTGAAGCTTGGAAACTACAAAGGCATCACCAAACATTTAGAGAAGAACCAAAACCCGGTATGGAATCAAATTTTTGCCTTCTCAAAGGAAAGATTGCAATCCAGTTTGCTTGAAGTTACTGTCAAAGACAAGGATATTGGGAAAGATGATTTTGTGGGGAGAGTTAGTCTTGATCTATCACAGGTCCCTCTTCGTGTTCCGCCTGATAGTCCATTGGCTCCTCAATGGTATAGATTGGAGGACAAAAAGGGTGATCAAACTACTAAAGGGGAAATAATGCTTGCTGTTTGGATGGGGACACAAGCTGATGAGTCTTTTGCTGAGGCTTGGCACTCTGATGCACACAACATCAGTCAGAAAAATTTAGCTAACACCAGATCAAAGGTTTACTTCTCACCCAAGCTTTATTATCTTCGGGTTTTTGTATTTGAAGCACAAGATCTTGTTCCTTCTGACAAAGGGCGAGCACCGGATGCTTGTGTGAGGATCCAGCTTGGAAATCAGCTGAGGGTTACCAGGCCTTCGCATGTGCGAAGTGTTAACCCGGTATGGAACGAAGAGCATATGTTTGTTGCATCTGAACCGTTTGAGGATCTGATTATTGTGACTGTTGAAGATAGGATTAGACCTGGCAAGGATGAGATTTTAGGAAGAGAATTGATACCAGTTAGAAATGTGCCACAAAGACATGAGACCACTAAGCTCCCAGATCCTCGATGGTTCAACCTTCACAAACCTTCGTTGTCAGCAGAGGAAGGCGCTGAAAAGAACAAGGAAAAATTCTCAAGCAAGATTCTAATTAGCTTCTGTTTGGAAGCAGGTTACCATGTTTTTGATGAATCCACACATTTTAGCAGTGACCTGCAGACCTCATCGAAGAGCCTGAGGAAGGGTAGCATTGGGACTCTTGAGCTTGGCATTTTGAGTGCAAAGAATTTGATGCAAATGAAGAGCAAGGATGGTAAACTGACTGATGCATATTGCGTGGCCAAGTACGGGAACAAATGGATTCGAACCAGAACAATCCTTGACACACTGGCTCCTAGATGGAATGAGCAGTATACTTGGGATGTTTATGATCCGTGTACTGTAATCACAATTGGAGTATTTGACAATTGCTACGTCAATGGAAGCAAAGATGACGCGAAAGATCAGAGAATCGGAAAGGTGAGAATTCGATTATCAACTTTAGAAACCGATCGTATATACACCCATTACTATCCCTTGTTGCTACTCACGCCTTCTGGTTTGAAGAATAATGGGGAACTTCATTTGGCTTTAAGGTTTACGTGTACAGCTTGGGTAAACATGGTGACAAAATATGGAAGGCCGTTGCTTCCCAAAATGCATTATGTCCAACCAATACCTTTTATACTCATTGATAGGCTACGGCATCAAGCAATGCAGATAGTTGCAGCAGGGCTGGGAAGAGCAGAGCCGCCACTGAGACGCGAGGTAATGGAGTACATGCTGGATGTGGATTACCATATGTGGAGTTTGAGGAAATGCAAAGCCAATTTTCAAAGAATTGTGGAACTTCTTTCAGCTATTTGTAGATGGTTCAATGACATATGCACATGGAGAAACCCAGTGGAGACAGCCCTTCTTCATGTATTGTTCTTGACACTCGTTTTCTACCCTGAGTTGATACTGCCAACAATTTTCCTGTACCTCTTCCTAATTGGGATGTGGAACTATCGGTTGAGGCCAAGGCACCCACCTCACGTGGATGCTAAGCTCTCGCAAGCAATAAATGCACACCTAGATGAGCTAGTCAAAGAATTTGATACGTCAGATGAGCTAGATGAAGAATTTGATTCGTTTCCAACATCACGACCATCAGACACCGTCCGGATGAGATATGAAAGGCTACGCAGTGTGGGAGGTCAGTTGCAGACAATGGTTGGAGATTTGGCCTCGCAAGTCGAAAGAGCTCAAGCGATCCTCTGCTGGCGTGATCTAAGAGCTACGTTTATCTTTCTCATCTTCTCATTTATTTGGGCAGTGTTTAGTTATGTTACTCCATTCGAGGTGGTGGCAGTTCTGATTGGCCTTTACATGCTGCGCCATCCCCGGTTCAGGAGCAAGATGCCTTCAGTACCTGTCAACTTCTTCAAAAGTTTTCCGTCCAAGTCAGATATGCTGATATAA

>Cicle10030764

ATGAGACCACCACTGGAAGAGTTTTCTTTGAAAGAGACGAAACCCCACTTGGGTGGGGGAAAGATAACCGGTGACAAGCTCACTAGCACCTATGACCTCGTTGAGCAAATGCAGTACCTTTATGTCCGGGTTGTAAAGGCCAAGGACTTGCCCCCGAAGGATGTCACTGGTAGTTGTGATCCTTATGTCGAAGTTAAGATGGGGAATTATAAGGGTACAACTCGGCATTTTGAAAAGAAAACAAATCCTGAGTGGAACCAGGTGTTTGCTTTCTCGAAGGATCGTATTCAGTCTTCTGTGCTTGAGGTTACTGTGAAGGATAAGGATTTTGTTAAGGATGATTTTATGGGTAGGGTTTTGTTTGATTTGAATGAGATTCCAAAACGAGTTCCACCTGATAGTCCATTGGCACCACAGTGGTATAGGTTGGAGGATAGAAAGGGGGATAAGGTTAGGGGAGAATTGATGTTGGCTGTGTGGATGGGCACTCAAGCAGATGAGGCATTTCCTGAGGCGTGGCATTCCGATGCGGCTACTGTCACTGGAATTGAGGGTCTTGCGAACATACGGTCAAAGGTTTATCTCTCGCCTAAGCTTTGGTATTTGAGGGTTAATGTGATTGAAGCTCAGGATCTGCAACCCACCGACAAGGGTAGATTCCCAGAAGTTTATGTGAAGGCCCAACTGGGAAATCAGGCTTTGAGAACAAGAGTTTCTGCTAGCAGGACCATTAATCCAATGTGGAATGAGGATCTGATGTTTGTGGCAGCTGAACCATTTGAAGAGCACTTGATTTTGACTGTGGAAGATAGAGTTGCACCTAACAAGGATGAAGTTCTGGGGAAATGTATGATCCCATTGCAGTATGTGGACAAGAGGCTTGATCATAAACCAGTGAACACTAGGTGGTATAATCTTGAAAAGCATATCGTTGTAGAAGGGGAGAAGAAGAAAGATACAAAGTTTGCTAGCAGGATCCACATGAGGATTTGTTTGGAAGGTGGTTATCATGTTTTAGATGAATCAACACACTACAGCAGTGATCTTAGGCCAACGGCAAAGCAGTTATGGAAGTCCAGCATAGGGGTCTTAGAATTGGGGATTTTGAATGCTCAAGGGTTAATGCCAATGAAGACTAAAGATGGTCGAGGAACTACAGATGCGTATTGTGTGGCAAAGTATGGCCAGAAATGGGTTCGAACCAGGACAATCATTGATAGCCCTACTCCCAAGTGGAATGAGCAGTATACATGGGAAGTTTTTGATCCGTGTACTGTTATAACCATTGGGGTATTTGATAATTGTCACTTGCATGGAGGAGATAAAGCTGGTGGGGCAAGGGATTCAAGGATTGGAAAGGTAAGGATCCGTCTTTCTACCCTTGAAACAGATCGGGTTTACACTCATTCGTATCCACTGCTGGTGCTATACCCAAATGGTGTCAAGAAGATGGGTGAAATTCATTTGGCTGTGAGGTTTACATGCTCTTCATTGCTTAATATGATGCACATGTACTCGCAGCCACTGTTACCAAAGATGCACTATCTGCATCCATTGACTGTCAGCCAGCTCGATAGCTTGAGACACCAGGCCACTCAGATTGTGTCCATGAGACTTAGCCGAGCTGAGCCGCCGTTGAGGAAAGAAGTAGTCGAGTACATGTTGGATGTGGGCTCTCATATGTGGAGCATGAGAAGAAGCAAAGCTAACTTTTTCAGGATTATGGGAGTTTTGAGTGGGATAATTGCTGTTGGAAAATGGTTTGATCAGATCTGCAATTGGAAAAACCCCATTACGACAGTGTTGATTCACATCTTGTTCATCATACTGGTGCTGTATCCAGAGCTTATCTTACCAACAGTGTTCCTCTATCTCTTCTTGATTGGTGTTTGGTATTATAGATGGAGACCAAGACATCCTCCTCACATGGACACCCGTCTCTCACATGCAGATTCTGCTCATCCTGATGAACTTGATGAAGAATTTGATACATTTCCAACTTCTAGGCCTTCTGATATTGTAAGGATGAGATATGATCGACTGAGAAGTATTGCTGGAAGAATTCAGACTGTGGTTGGTGATTTAGCTACTCAAGGGGAGAGGCTGCAGTCTTTACTGAGCTGGAGAGACCCAAGAGCCACAGCTCTGTTTGTAATTTTCTGTCTGATTGCAGCCATTGTTCTTTATGTTACACCATTCCAAGTTGTGGCTCTTCTAACGGGATTTTATGTGTTAAGACATCCAAGGTTCCGTCACAAGCTTCCTTCCGTACCGCTCAATTTCTTCAGGAGGTTGCCAGCAAGAACTGATTGCATGCTATGA

>Cicle10030600

ATGAAGCTGGTTGTGGAAGTAGTAGATGCTTATGATCTTATGCCCAAAGACGGTGAAGGTTCAGCTAGTCCATTTGCAGAAGTTGATTTCCTAAACCAACTTAGCAAAACCAAAACAATTCCAAAGAACCTCAACCCTGTTTGGAACCAGAAACTTCTCTTTGATTTTGACCAAACCAAAAGCCATAACCACTTACGTATTGAAGTCTCTATTTACCATCACGAAAGAAGGCCAATACCGGGCAGGCACTTCCTTGGCAGAGTTAGAATTCCTTGCTCAAATCTTGTCAGAAAAGGCGAGGAAGTTTACCAAAGATTCCCACTTGAAAAGAAGTGGCTTCTCTCATCTGTCAAAGGTGAGCTTGGCCTCAAAATCTACATTTCACCACAATCTGAAACGACACAGCCCCCAACTTCAAGTTTACCAAAACCAAAATCACCAAAAAATACCACCAATCTTGATAGCAAAACTTTTACTGCTCTTCCCAAAGTTGAAGAGCTAGCTGCCGTTGATGCTCCAAAATCTCTGTCCGAAGAAGAAATTTCTAGAATTTCTTTAAAAGAGGATATCAAAGAGCCTGCCAAAGTAACAGTAGAGCCTATCCAAGAGTTCCTCAAACAACAAGTTGTTCTGCAACCAGGCCAATCAGTGGAGAAACAACCACAAGGTGTTCCATTCACCATGCATTCTATGAATCTACAGCAAGGGCGTCCTGGTGACCAAGAGGAGTACAATCTGAAGGACACCAACCCCCAACTTGGGGAGCGGTGGCCAAATGGTGGAGGATATGGAGGAAGAGGATGGATGAGTGGTGAAAGATTCACGAGCACGTACGACCTTGTTGAGCAGATGTCCTATCTGTATGTTAGAGTGGTGAAAGCTAAAGATCTTCCCCCCAGTTCCATTACTGGGAGCTGTGATCCATACGTAGAAGTAAAGATGGGAAACTACAAAGGAAGGACTAAGCATTTTGAGAAGAGAATGAATCCAGAGTGGAACCAGGTCTTTGCTTTCTCCAAAGAACGCATTCAGTCATCAATGCTTGAAGTTTTCGTGAAGGATAAAGAAATGTTAGGCAGAGATGATTATCTTGGAAGGGTTGCTTTTGACTTGAATGAAGTTCCAACAAGAGTTCCTCCTGATAGTCCACTGGCTCCTCAATGGTACAGGCTAGAGGATCGACGTGGTGAAGGAAAAGTAAGAGGACAGATCATGCTTGCAATTTGGATGGGAACTCAAGCTGATGAAGCATTTGCCGAGGCTTGGCATTCAGATGCTTCGTCAGTATATGGAGAGGGTGTTTTTAACATCCGATCGAAGGTTTACGTATCGCCAAAGCTGTGGTATCTAAGAGTGAATGTGATTGAAGCACAGGATATTGTGCCTAATGACAGAAACCGGCTTCCTGAAGGTTTTGTGAAAGTTCAAGTTGGAAACCAAGTACTCAAAACCAAGATATGCCCAACTCCAACTACTAACCCACTTTGGAATGAAGATTTAGTTTTCGTAGCAGCTGAGCCTTTTGAGGAGCAGCTATTTCTAACAGTTGAGGATCGAGTTCATGCTTCGAAAGATGAGGTGCTGGGGAAGATAAGCTTACCACTGAACATATTTGAGAAACGGCTAGACCACAGGCCAGTTCATTCACGTTGGTTCAATCTTGAGAAATTTGGTTTTGGTGCGATAGAAGCTGACAGAAGGAAAGAGCTCAAGTTTTCAAGCAGAGTTCACCTTAGAGTTTGTCTTGAAGGTGGGTACCATGTGCTAGATGAATCAACAATGTACATAAGTGATCAGAGGCCAACAGCAAAGCAGCTCTGGAAACCGCCAGTTGGGATATTAGAAGTTGGCATTTTGGGTGCACAAGGACTTCTACCTATGAAGATGAAGGATGGCAGAGGAAGCACAGATGCTTACTGTATTGCTAAGTATGGTCAGAAATGGGTTCGGACAAGAACAATTCTTGACACATTTAATCCCAAATGGAATGAGCAATACACTTGGGAAGTTTATGATCCCTGCACAGTGATCACGCTGGGAGTATTCGACAACTGCCACTTGGGTGGAGGTGAAAAACAAAACGGTTCCAGTGCAGTAAGAGACTCGCGAATAGGCAAGGTACGAATAAGGCTCTCAACACTTGAAGCACATCGAATTTACACGCATTCTTATCCACTCCTTGTTCTTCATCCACATGGAGTTAAGAAAATGGGTGAGCTTCAGCTAGCAATTAGATTCACTATATTCTCTTTAGCTAGTATGATATATGTGTATGGGCATCCATTGCTGCCTAAAATGCATTACCTGCATCCTTTCACTGTGAATCAAGTGGACAATTTAAGGCACCAAGCCATGAATATTGTTGCAGTGAGGCTTGGTAGAGCTGAGCCACCTCTGCGAAAAGAGGTGGTGGAATACATGTTAGATGTTGATTCCCATATGTGGAGCATGAGAAGAAGCAAAGCTAACTTTTTTCGGGTTATGTCACTGCTTTCGGGCATGATATCTGTCAGCCGATGGTTCACTGATATATGCAACTGGAGAAATCCAGTAACAGCAGTTCTAGTTCATATCCTCTTCCTGATACTAATTTGGTATCCTGAGTTGATTCTCCCAACCGTATTTTTATACATGTTCCTCATTGGACTCTGGAACTATAGGTTCCGGCCAAGACACCCCCCTCATATGGATACCAAACTTTCATGGGCAGAAGCTGTCCATCCAGATGAGCTAGATGAAGAATTTGACACTTTTCCAACTTCAAAAACTCACGATATTGTTCGAATTAGGTATGATCGGCTTAGAAGTGTTGCAGGGAGGATTCAAACAGTGGTGGGTGACATAGCAACTCAGGGTGAGAGGTTTCAGTCATTGCTAAGCTGGAGAGACCCAAGAGCAACTGCCCTCTTCATACTGTTCAGTCTGTGTGCAGCTATGGTTCTGTATACAACCCCATTTAAAGTGGTGGCTCTGCTTGCCGGTTTGTATTATTTACGGCACCCAAGATTCCGTAGCAAGCTGCCTTCCGTACCCAGCAACTTCTTCAAGAGAATGCCAGCTCGTACTGACAGCTTGCTGTGA

>Cicle10033973

ATGAATCCATTATCGGCTCCTTATTATCAAGAAGACTACAAATTAAAGGACACAAAGCCCCAGCTTGGGGAACGATGGCCACATGGAGGAATACGTGGTGCGGGTGGGTGGATAAGCAGTGAAAGAGCTACAAGCACATATGATCTTGTTGAGCAGATGTTTTATCTGTATGTTCGAGTTGAGAAAGCCAGAGATCTTCCCACAAATCCTATGTCCGGTAGCTGTGATCCTTACGTTGAAGTAAAGCTTGGAAATTATAAAGGGAAAACAAGGCACTTCGAGAAGAAATCAAATCCTGAATGGAAACAGGTTTTTGCATTCTCCAAGGAGAAGATTCAGTCTTCGGTTCTTGAGGTGTTTGTCAGAGACAGAGAAATTGTGGGCAGAGATGACTACATAGGGAAAGTGGTGTTTGACATGAATGAAGTGCCTACAAGGGTTCCACCAGATAGCCCTTTGGCACCACAGTGGTACAGATTAGAGGACAGGCGAGACGATAGGAAGGTGAAAGGAGAGGTTATGCTTGCAGTTTGGATCGGAACACAAGCTGACGAAGCTTTCCCAGAGGCCTGGCATTCGGATGCTGCTACAGTTGAAGGAGAGGGTGTTTTCAATATCAGATCAAAAGTTTATGTTTCACCAAAACTTTGGTATCTCAGAGTTAATGTGATTGAAGCTCAGGATGTTGAGCCACTTGACAAAAGCCAGTTACCACAGGCTTTTGTAGAGGCTCAAGTTGGAAACCAGGTGCTTAAAACCAAGCTATGCCCAACCAGAACAACCAATCCTCTCTGGAATGAGGATCTGATATTTGTTGCAGCTGAACCGTTTGAAGAACAGTTGGTACTAACAGTTGAAAATAAAGTCACCCCTGCTAAAGATGAACCTCTGGGGAGATTACGTTTGTCACTAAATGTCATTGAGAGGCGCCTGGATCACCGGCCAGTTCATTCTAAATGGTTCAACCTCGAAAAATTTGGATTTGGTGCTTTGGAGCTAGACAAGAGGCATGAGCTCAAGTTCTCCAGCAGAATTCATCTCAGAGTCTGCCTAGAGGGTGCTTATCATGTAATGGATGAATCAACAATGTATATAAGCGACCAGCGCCCAACTGCTAGGCAGCTGTGGAAGCAGCCGATTGGGATCCTTGAAGTAGGCATCTTGAGCGCTCAAGGACTCCTTCCAATGAAGACAAGGGATGGTAGGGGAACCACAGATGCCTATTGTGTTGCCAAGTATGGGCTGAAATGGGTGCGAACCAGAACATTAGTTGACAACTTCAATCCGAAATGGAATGAACAGTACACTTGGGAGGTTTATGACCCCTGCACAGTGATCACACTGGGGGTATTTGACAACTGTCACTTGGGTGGCAGTGGAACAAAACCAGATTCAAGAATTGGGAAGGTAAGAATCCGGCTATCAACACTAGAAGCAGATCGAATCTACACTCACTCTTATCCACTTCTTGTTCTAAACCCATCAGGAGTGAAGAAGATGGGAGAACTACAACTAGCAGTTAGATTTACCTGTCTATCTCTAGCAAGTATGATCTACCTCTATGCACACCCCTTATTGCCAAAAATGCATTATCTGCATCCATTCACTGTCAACCAATTAGACAGCCTGAGATATCAAGCTATGAACATTGTAGCAGTGAGGCTTGGCCGTGCTGAGCCACCACTGAGGAAAGAGGTGGTCGAATACATGCTGGATGTAGATTCTCACATGTGGAGCATGAGAAGAAGCAAAGCTAACTTCTTCAGAATAGTCTCACTGTTTTCAGGTGCAATCTCTATGAGCAAGTGGCTCGGTGAAGTTCGCTACTGGAAGAACCCAGTGACTACTATTCTTGTTCATGTTTTATTTCTGATATTGATCTGTTACCCAGAATTGATCCTGCCAACAATTTTTCTATACATGTTTCTCATTGGTATCTGGAACTACCGGTTCCGGCCTAGGCATCCACCTCACATGGATACCAAACTTTCATGGGCAGATGCAGTTCATCCAGATGAATTGGATGAAGAATTTGATACTTTTCCTACATCTAAGCAGCAAGATGTAGTGAGGATGAGGTATGATAGACTCAGAAGTGTAGCAGGAAGGATTCAGACGGTGGTCGGCGATATGGCAACACAAGGGGAGAGATTTCAGGCTCTGCTTAGTTGGAGAGACCCAAGAGCAACTAGCCTGTTTGTAATTTTCTGCCTAATTGCAGCTGTTGTGCTATACGTGACGCCATTCAAGATAATCACATTAGTTGCAGGTTTATTCTGGCTGCGGCATCCCAGATTTCGCAGCAAGCTACCGTCAATACCAAGCAATTTCTTCAGGAGATTGCCATCGCGTGCCGACACCATGCTCTGA

>Cicle10033918

ATGACAGAGCTGAAAGAAGACTTTTCATTGAAGGAGACCTCACCGAAAATTGGTGGAGGGAGAGTCTCTGGTAGAGAAAGGCTCACATCATCATTCGACCTTGTTGAACAAATGGAGTTTTTGTATGTAAGGATAGTGAGAGCCAGAGATTTGCAAGTCAATCAAGTGACCGGAACTTGTGACCCTTATGTTGAGGTAAAGATAGGAAACTATAAGGGAACAACTATCCCCTTTGAGAAGAAACTAAACCCAGAATGGAACCAAGTTTTTGCCTTTACTAAAGAGCGTCTCCAGGCCATATCTGTAGAGTTATTGGTTAAGGATAAGATGATTGTCAATGGTGATTTTATTGGTAAGATTAAGATTGACATGCCTGATATTCCTAAACGTGTTCCACCGGATAGTCCTTTGGCGCCAGAGTGGAAAAGATTGGAGGCTAAAGATGGCAGCAGGGCTAGAGGAGAGTTGATGTTTGCTATTTGGTTTGGAACTCAAGCAGATGAAGCATTTTCCAGTGCTTGGCATTCAGATACAGCTGTTGTTAGTGGTGAGAATATTATGAATTGTCGTTCAAAAGTGTATGTTTCACCTAAACTTTGGTATCTTAGGGTTAATGTGATTGAAGCTCAAGATTTGGTACCTAAACAAAGGAATAGAAACCCTGAAGTTTTCATCAAAGCAATTTTTGGGAATGTGGTTCTGAAGACTACTGTTTCAGCCAAAAAAACTGCGAACCCAACTTGGAATGAGGACTTGATGTTTGTTGCAGCAGAGCCATTTGATGACCCTTTGATTCTGACTGTGGAGGATAAACTTGGAGATAATAAAGAGGAATGTCTGGGGAGATTAGTGTTGCCCTTATCTAAGGCTGGTAAGAGGTTCCTGCCATTGCCTGCCTCTGCTATATGGTATAATCTTGAGAGGAATATTGCCGATGGTGAAGAGAAAAAAGATGTCAGATTCGCTAGCAGAATTTGTTTGAGATTCTCTTTGGACGGTGGGTATCATGTTTTCGATGAAGCCACTAACTATAGCAGTGATTTGAGGTCCACGATGAAGCAGTTGTGGCCACCAGTGATTGGGGTTTTGGAGTTGGGGATTTTAAGTGCTAAAGAATTGCTGCCAATGAAATCAAGAGATGGAAGAGGAACTACCGATGCTTATTGTGTGGCTAAATATGCTAACAAATGGGTGAGAACAAGGACTGTTGTTGATAGCTTTGATCCAAAATGGAATGAGCAATACACTTGGGAGGTTTATGATCCTTACACTGTCATTACCTTAGTAGTTTTTGATAATTGTCATTTACATCCTGGAGGAGCCAAGGATTCAAGAATTGGCAAGGTAAGGATTCGACTGTCTACGCTTGAAACTGATAGGATCTACACTCACTCTTATCCTCTTGTAGCCTTGCTACCGAATGGGGTGAAAAAGATGGGAGAAGTTCAGCTGGCTGTGAGGTTTACTTGTTCTTCTTTTGTCAACCTGCTCCAAACTTACTCACAACCTCTCTTGCCCAAAATGCATTACATTAACCCGCTTTCTGTTTTTCAGATTGATAGTTTAAGGCACCAGGCTACTCATTTGCTCTCCTCAAGGCTGAGTCGTGCTGAGCCACCATTAAGGAGAGAGGTTGTTGAGTATTTACTCGATGTAGGTTCACAGATGTGGAGCATGAGAAGAGGCAAAGCCAATCTTGCAAGACTCATGAGATTTTTGAATGCATTTGGTGTTGCTTGGATTTGGTTTGATCAAGTACGCCGATGGAAGAACCCAATGACAACAATTTTTGTTCATGTCTTCTATGTGATTATGGTTCTTTTCCCACAAATGATTTTGGCCATGTTCTTTTTTAGCTTATTTGGTGTTGTGATTATGAAGTTCAAAAAGAGGCCAAGACTTCCTCCTCATATGGACATTAAATTATCATTTGCTGACAAAGCTCATCCTGATGAGCTGGATGAAGAGTTTGATACATTCCCATCTTCCAAGCAAGGTCACATTCTAACAACCAGATATGATCGATTGAGAAGTATAGCAGCAAGGATGGTTACATTAAATGGTGACTTGGATAGTCAATTGGAGAGGCTGCAGTCATTAATAGATTGGCGGGATCCGAGAGCCACAGCTATGTTCTCAATTTTTTGTTTGATGGCTGCTGTAGTGTTTTACATAGTTCCCCTTTGGATTTTACTCCTATTTGCAGGTCCTTTTGTGATGAGGCATCCAAGATTCCGTATTGACATTCCTGCATTACCACAAAACTTCTTAAGGAGATTACCTTCAAAGGCAGAGAGTTTGTTGTGA

>Cicle10024404

ATGGGCGACAGGAAAGAGAAGCTAGTAGTGGAGGTGATAGCAGCACACAACTTGATGCCTAAAGATGGAGAAGGCTCATCATCCCCGTTTGTAGAAGTTGAGTTTGAGAAGCAAATACTAAGAACCCAAGTGAAGTACAAAGATTTAAACCCAATATGGAACGAGAAGCTTGTGTTTGACGTTCCAGATATCGCGGAGCTGCCTTACAAACACATTGAGGTTAATGTGTTCAACGAGAGAAGGTCAAGCAACAGCAGAAATTTTCTGGGTAAAGTCAGGGCTCCTTGTTCACAACTCTGTAAGAACGAAGGTGAAGCCACTGCTCAGTTGTACACTCTAGAGAAAAGGAGCTTATTTTCTCACATAAGGGGGGAGATAAGCTTGAAGCTTTTCGTGTCTACGACAGAGGAGGTGGTTAAAAAAGGAGGCTTTGTTTCTTCTTTGACTCCGTCCTCTGCGTTTTCTAAGAAGAACAAGAAACTGCAGCAACAGAGTCCTGTTATGCAGGTGCAGCAGCAGCATTTTGGCCATCAAGACATGATGAGCAAGCCAACTCATCAGCAGCAGAGTCAAAATCACGTGAAGCCCATGGAGCCAAACCCGGGTGAGTTAAAGCCTGTTGTGATTACTACTGCTCCTCGTCCAGTCATTCCCGGAGCCAGAGGTGGTCCAACCTCCGGCGGCGGAGGCGGCGGAGGTGGTGGTGGGGTTTACGTTAATGGGTCAGGCGAGTTTTCTTTAAAAGAGACAAGTCCTCATCTTGGTGGTGGTCCTCTCAACAAGGACAAAACTAGTTCTACTTATGATCTTGTTGAGCAAATGCAGTATCTTTATGTTAGAGTCGTGAAAGCTAGAGATATTTCTTTGTTTGGAGGTGGTGAGATTGTAGCTGAAGTGAAACTAGGGAACTACAGAGGAATTACAAAGAGGGTGAGTTCAAATCATTTAGAATGGGACCAAGTCTTTGCATTTTCAAAAGATTGTATACAGTCCTCAGCGGCTGAGATTTTTGTCAAAGAAAGCGACAAAGATGATTTCTTGGGGAGAATTTGGTTTGATTTAAATGAAGTTCCTAGAAGGGTACCTCCTGATAGCCAGCTAGCACCACAATGGTATAGGATGGAAGATAGAAGAGGTGATAGGTCAAAAGGAGGTGAAGTTATGGTCTCAATATGGTTTGGAACTCAGGCTGACGAGGCATTTGCGGAGGCATGGCACTCAAAGGCAGCAAATGTTCATTTTGATGGGCTTTGCTCTCTTAAATCCAAGGTTTATCTGTCACCAAAACTTTGGTATTTGAGAGTCTCGGTTATTGAAGCGCAAGATATTGTTCCTGGGGATAAAGGGTCTGCTATGATGAGGTTTCCGGAGCTTCATGCAAAAGCACAGGTGGGGAATCAATTTTTAAAGACTAGAATTGCAGCGCCTAGTGCAACAAGAAGCTTGTCAAATCCTTGTTGGAATGAGGATTTGTTGTTTGTGGTTGCTGAGCCATTTGAGGATTATCTTTTGATTTCAGTTGAGGACCATGTTGGGCCTGGTAAAGATGAGATCGTGGGTAAGGTTTTGATTCCAGTGAGCACAGTTGAGAGAAGAACTGATGATAAACAAGTGGTATCCCGGTGGTTTAATCTTGAAAACCATTTTGGCAACCAGGGGGAGTCAAAAGTTGTGACAAGATTCGGATCTAGAATTCATCTTAGGGTCTCTCTTGATGGTGGCTACCATGTGCTTGATGAGGCCACACTGTATAGCAGTGATGTGAAGCCAACGGCAAAGCAGTTGTGGAAGCCTCACATTGGTGTGCTTGAAATGGGCATTCTGGGTGCAACAGGGCTTATGCCAATGAAGTTCAAAGAGGGGAAAGGAGGATCTGTTGATGCTTACTGTGTTGCGAAGTATGGCCAGAAATGGGTGAGAACACGAACTGTGGTTGATAGTTTGTCTCCCAAATGGAATGAGCAGTATACTTGGGAAGTTTTTGATCCTTGCACAGTCATCACGGTTGGAGTGTTTGATAACTGCAGCCTTGATAAGAACATAATTAACAACAGTGGTGGTCGTGATTCTCGAATTGGGAAAGTCAGAATCAGGCTGTCTACACTTGAATCTGATCGCGTTTACACTCACTCATATCCACTGCTTATGTTGCATCCTTCAGGTGTGAAAAAAATGGGTGAGCTTCATTTGGCTGTGCGATTTTCCTGTGCTAATCTGGTTAATATGTTGCATATGTACGCAATGCCGTTGCTACCAAAGATGCACTATGTGCACCCTTTGAGCGTGCATCAACTTGAAAGCTTGAGGTACCAAGCTCTGAATGTGGTGTCATCCCGGCTCAACCGAGCAGAGCCACCACTGGGGCGAGAGGTGGTTGAGTATATGCTTGATTATGACTCTCACATGTGGAGCATGAGAAGGAGCAAGGCTAACTTTTTTCGGCTAATGAATGTGATCTCTGGTCTCATAGCAATGGTTCGTTATGCGGAGTCTATGCGCAATTGGCACAAGCCAATATACTCAACCTTGTCTTTGGCATTTTTCTTCTTGCTGGTTCTAATGCCTGAGCTTGTAATCCCTGCCATATTGCTATACTTGTCACTTCTGGGGCTCTGGCGATATAGGTCCAGGTCACGGCACCCTCCTCACATGGATATCAGGCTCTCTCAGGCTGACAGTGTCTTCCCTGATGAATTGGATGAGGAATTTGATTCCTTTCCGACAAGTCGAGGTGCTGACATTGTTCGAATTAGGTATGATAGGCTAAGAAGCGTGGCTGGAAGAATTCAGACCGTGGTTGGAGATATGGCCACACAAGGTGAACGGTTTCAAGCATTAATAAGCTGGAGAGACCCGCGGGCCACATTCTTGTTTGTGATTTTCTGCTTGTTTGCTGCAATTGGGTTTTACGCCGTGCCACTTCGCGTAGTGTTTGCATTGTCGGGGGTGTATGTGCTGAGGCCACCACGGTTCAGAAGCAAATTGCCTTCTCCAGCTTTGAGTTTCTTCCGGAGGTTGCCCTCAAAAGCTGATACCTTGTTGTAG

>Cicle10018633

ATGGCTGCAATCCAAAAACTGATCGTTGAAGTTGTTGATGCACGCAACCTTTTACCAAAAGATGGGCACGGGACCTCCAGTCCCTACGTCGTGATTGACTACTACGGGCAGAGAAGAAAAACCCACACAGCGGTTCGTGATTTGAACCCGACATGGAACGAGGCACTCGAGTTCAACGTTGGTAAGCCACCTCAAGTGTTCACTGACATGTTTGAACTTAACATATTTCATGATAAAGCGTACGGTCCTACTACAAGGAATAATTTTCTTGGTAGGATTCGGTTGAGTTCAAGCCAGTTTGTTAAGAAAGGTGAAGAAGCCTTGATTTATTACCCTCTTGAAAAGAAAAGCTTGCTTACTTGGATCCAAGGTGAAATTGGGTTGAAAATTTATTATGTTGATATTGTTCCGACGCCGCCACCTGCGGCGCCAGCGCCAGCGCCAGTTCCACAGCCAGATCCTCCTGCGAAGGAAGTGAAACCTGATCCAACTGTCGAAGCTAAAGCTGAAGCCGCGAAGCCCAATGTAGAGCCAGCCGCCGATCATGAGGCTAAAGTTGAAGCTGAAGCAGTACCAGCACCAGAAAATAAAGAGCCTGCAGGGGATAAAGAACTCCAGTGCGACACGTCATCAGCACCAGAGCAAGTACAAGCAAATGAAGAACAAGCTCGACAGCAGCCATCCATGCAGGAGCAATCTGGCCATAGTGAGTTTGATCTTACAACGGCCAAGGCGGGCCCGGAAGCTCCAGCGGCACCGTCTGATCACGTGATGGCGGCATCAATCTCCGGGTCTGTGCCGGAGGTCAAAGTAACTCCTCCGAGCAGCAGTCCACAGCCAATCTCAAGGTCAGCGTCAATGGCGATCTTCGCTTCAGCCACATCAGGTAACATTCCGATCAACGGTCCACAGCCAATCTCGAGGACAATGTCAACGGCAAGCTTCGCATCAGACATAACAGATAACATTCCGATCGAACGGTCGTCGTTTGATCTTGTGGAGAAGATGCATTACCTCTTCGTACGAGTAGTTAAAGCTCGGTTCTTACCCACCAAAGGCAGCCCGGTTGTAAAGATAGCCGTTGCGAACAGCCGCGTCGAATCGAAACCCGCCCGTAGAACCTCGTGCTTCGAGTGGGACCAGACGTTTGCTTTTGGCCGAGACTCGCCCGAATCCTCCTCATTCTTAGAAGTCTCGGTGTGGGACCCGCCGAGAGGTGACGTGGCAGCTCCTCCTGGTTTCTTAGGTGGCATTTGCTTCGACGTTTCGGAGATTCCATTGCGGGATCCGCCGGACAGCCCATTGGCCCCTCAGTGGTACAGGATGGAGGGTGGTGGGGCCTACAGTGGAGACTTGATGCTCGCCACTTGGGTTGGTACCCAGGCTGACGATTCATTCCCTGACGCATGGAAAACCGACACAGCCGGTAATGTTAACTCTAAAGCCAAAGTTTATGTCTCTCCAAAATTGTGGTACCTTAGAGCTACTGTAATTGAAGCACAAGATATTTTGCCGCCAGTAGCGGCGTTAAAAGAAGCCTCGTTTACAATCAAAGTCCAGTTAGGGTTTCAAGTACAGAAAACCAAAGTCTCCGTTACGCGCAACGGAACTCCGTCGTGGAACGAAGATTTACTCTTTGTAGCTGCCGAACCCTTTACTGATCAACTGAGTTTCACGTTAGAAAACCGACAACATAAAGGATCAGTAGCACTCGGAGTCACTAGAGTTCCACTAACGGCTGTTGAAAGGCGAGTTGATGACCGGAAAGTGGCGTCGAGATGGTTCACGTTTGAAAACACAAATGATGAAAAGAGGGCATACAAAGGTAGGGTCCACCTGAGGCTGTGTTTCGATGGTGGGTATCACGTGATGGACGAGGCGGCGCACGTGTGCAGTGACTATCGACCTACAGCTAGACAGCTTTGGAAACCGCCGGTAGGAACCGTTGAACTTGGTGTGATTGGGTGCAAGAATTTGTTACCGATGAAAACCGTAAATGGTAAAAGTACCACGGATGCTTACGTGGTGGCAAAGTACGCGTCCAAGTGGATTCGGACCCGTACGGTTTCGGACAGCTTGGAGCCACGTTGGAATGAGCAGTACACGTGGAAGGTTTATGATCCTTGTACGGTGTTAGCACTGGGAGTCTTTGATAGTTGGGGAATATTTGAAGGTGAAAACGGTAGCATGGAAACGACACGCCCTGATTGTCGTATTGGTAAAGTACGAATACGTATTTCTACTCTGGAGACCGGTAAAGTTTATAGAAATACGTATCCTTTGCTTTTGTTGGGAAGTAATGGGATGACAAAAATGGGTGAAATAGAAGTGGCCGTACGGTTCATACGTACAAGTCCGACGTTGGATTTCTTGCACGTGTACTCGCAACCTTTGTTACCATTGATGCATCATATCAAGCCACTCGGGATGGTCCAGCAAGAGATGTTAAGGAGTGGGGCTGTTAAGATTATAGCCGCACACTTGGCAAGATCAGAGCCACCGCTGAGGCGTGAGATAGTGCTATGCATGCTTGACGCGGATTCACACGCGTTTAGTATGCGGAAAGTGCGGGCTAATTGGTTTAGGATCATCAACGTGCTTGCGGGTGTGATTGATATCTTACGGTGGGCGGATGATACGCGATCGTGGAAGAATCCTACGGCTACCATTCTTGTGCATGCATTGTTGGTGATGCTGGTGTGGTTCCCTGATCTGATCGTGCCCACATCAGCATTTTACGTGTTTGTGATTGGCGTGTGGAATTACAGGTTCCGGAAGAGGGACCCACTGCCGCACTTTGACCCGAAGATCTCGCTGGCCGATACAATCGAACGTGACGAGCTTGATGAAGAGTTTGATACAGTACCAAGTGCAAGACCAAACGAAATCGTACGGGCCAGGTATGACAAGCTGCGTACGCTCGGGGCACGTGTGCAGACACTGTTGGGTGATTTTGCAGCGCAGGGGGAGCGGGTGCAAGCATTGGTGACGTGGAGGGACCCACGTGCCACGGGTATCTTTGTGGGGTTATGTTTTGTCGTGGCGATGATATTGTATTTGGTTCCATCCAAAATGGTGGCGATGGCATTTGGATTCTACTATCTGCGCCATCCGATGTTCCGTGATCGGATGCCATCACCAGCGTTGAATTTCTTCAGGAGGCTGCCTTCACTGTCCGATCGAATCATGTAG

>Cicle10018672

ATGAGTCATCTCAAGCTAGGAGTAGAAGTTGTGAGTGCCTACGAACTCATGCCCAAAGATGGGCAGGGCTCATCCAATGCTTTTGTGGAGCTCCATTTTGATGGTCAGAAATTCCGCACAACCACCAAGGAGAAAGATCTCACTCCTGTTTGGAATGAAAGTTTCTACTTCAACATTTCTGATCCCCACAATCTATCAAACCTTGCTCTTGATGCCTACGTCTACAATCATAATAGAACAACCAACTCCAAATCTTTCCTTGGTAAAGTCCGTCTTACTGGAACGTCATTCGTCCCTTATTCTGATGCTGTTGTTTTACATTATCCTCTGGAGAAGCGTAGCATCTTTTCTCGTGTTAAAGGAGAGCTTGGATTGAAAGTTTTTGTTACTGATGACCCATCAATAAGATCTTCAAACCCTCTTCCTGCAATGGAATCATTTGGGCATTCAGATTTGCGCTCAACCAAATCTCAAGCACCAGAACAAGTTCCAAGCTCAGCGCCTGACCCATTCTCAGATGATAAAGCTCGGAGAAGACACACATTTCATCATCTTCCAAATGCAAATATCTCGCAACAACAGCAACATTCATCCCCATCTGCAGCACAGCCATCAATGAATTATGGTGCTTATGAGATGAAATCTGAACCACAAGCTTCTAAAATTGTGCATACATACTCAGGGTTATCTTCACAACCTACTGATTATGCACTTAAAGAAACAAGTCCTTTTCTTGGAGGGGGACAAGTTATTGGAGGGCGAGTTGTGCGTGGTGACTTGCGAGCTAGCACCTATGATCTTGTTGAGCAGATGCGGTACCTTTTTGTACGAGTTGTGAAAGCCCGTGACCTTCCTTCCAAGGATGTGACTGGGAGTCTTGACCCATTTGTTGAAGTAAAGGTTGGGAACTACAAAGGGATTACAAAGTACTATGAGAAGAAACAGAATCCTGAGTGGAATGAAGTGTTCGCCTTTTCGCGGGAGAGGATTCAGTCATCTGTGCTGGAAGTTGCTGTCAAGGATAAGGATGTGGTGAAGGATGATTATGTTGGATTGGTCAGGTTTGATCTGAACGAGGTTCCCACACGAGTTCCACCTGATAGTCCATTAGCTGCAGAGTGGTATCGTCTTGAAGACAGGAAAGGGGAGAAGAAAAAGGGTGAACTGATGCTTGCTGTCTGGTATGGTACGCAAGCTGATGAGGCTTTTCCTGATGCATGGCATTCAGACGCTGTTACTCCCACTGATAGTCCTTCAAATGTCTCCACACACATCCGGTCTAAAGTTTATCATTCTCCAAGACTATGGTATGTCCGTGTTAATGTGATGGAGGCACAAGATCTTGTCATATCTGACAAGAATCGGTTTCCTGACGCATATGTTAAAGTGCAGATTGGTAACCAGGTTCTAAAGACAAAATCTGTTCAATCTCGAACTCTGAATCCAGTTTGGAATGAGGATATGATGTTTGTTGCTTCTGAACCATTTGAGGATCACCTGATTCTCACAGTTGAAGATCGTGTGGGCCCCAATAAAGATGAGACTATCGGTAAGGTTGTGATACCATTGCACTCTGTTGAGAAGCGTGCTGATGACCGCATTGTCCACACCAGGTGGTTCAATCTTGAAAAGTCCGTCTCAGCTGCGTTGGATGGAGATAATGCAAAGAAAGATAAGTTCTCTAGTAGACTGCATCTTCGTGTGTGTCTTGATGGAGGTTACCATGTGCTTGATGAGTCAACTCACTACAGCAGTGATCTCCGACCAACAGCAAAGCAGCTCTGGAAACCATCTATTGGTGTGTTGGAGCTTGGCATTCTAAATGCCGATGGCCTCCACCCGATGAAAACAAGAGATGGAAGAGGCACGGCTGACACATATTGTGTTGCAAAGTATGGTCACAAATGGGTTAGAACTCGAACCATAATTAATAGCTTGAGCGCGAAATATAATGAGCAGTACACCTGGGAAGTCTATGATCCAGCCACAGTTCTTACCGTGGGAGTTTTTGATAACAGCCATATTGGTGGTTCAAGTGGTAGCAAAGACGTGAAAATTGGTAAGGTTCGGATTAGAATCTCTACTCTTGAAACTGGTCGTGTATATACACACTCTTATCCCCTTCTAGTGCTCCACCCCTCTGGTGTCAAGAAGATGGGTGAATTGCATCTGGCAATACGGTTTTCTTATACATCATTTGCAAACATGATGTTTCTATACTCACGACCCCTCTTGCCGAAGATGCACTATGTCAGGCCATTGACCATGGCTCAACAGGATATGTTGCGTCACCAGGCTGTCAACATTGTGGCAGCTCGACTTAGTAGGGCAGAACCTCCTCTGAGGAAGGAAGTAGTTGAGTACATGTCCGACGTGGACTCTCATCTCTGGAGCATGAGGCGCAGCAAGGCAAATTTTTTCAGACTGATGTCAGTTTTCTCAGGATTATTTGCTGCTGGAAAATGGTTTGGGGAAGTTTGCATGTGGAGGAATCCTATTACGACAGTTCTAGTACATATTCTCTTTGTGATGCTTGTGTATTTCCCAGAACTGATTTTGCCAACTGTATTTCTGTACATGTTCATGATTGGGCTTTGGAATTACCGTTATCGTCCAAGATACCCTCCTCACATGAACACGAGAATCTCGTATGCAGATGCAGTGCACCCTGATGAGCTAGATGAGGAGTTCGACACATTTCCTACAACAAGGAGTCCTGACATAGTTCGCATGAGGTATGATCGGCTCAGGAGTGTGGCTGGAAGGATACAGACAGTCGTGGGTGATGTAGCTACTCAAGGAGAGCGAATTCAAGCGCTATTGAGCTGGCGGGATCCTCGGGCCGCAGCTATATTTGTGATTTTCTGTCTTGTGGCTGCTGTTGTGTTGTATGTGACACCTTTCCAGGTGCTTGCTCTTTTAGCTGGATGTTATATCATGAGGCATCCCAGGTTCCGGCATAAGACACCCTCTGCACCAATTAATTTCTTCAGGCGGCTTCCGGCTAGAACTGATAGTATGCTTTGA

>Cicle10023869

ATGAGAAACCTTAAGCTTGGAGTACAAGTGGTCGGTGCTCACAATCTTTTACCTAAAGATGGGAAAGGTTCATCCAGTGCCTTTGTGGAGCTCTACTTCGACGGCCAGAGGTTTCGTACCACTATCAAAGAAAATGATCTGAACCCTGTTTGGAATGAGAGCTTTTACTTCAACATCTCTGATGCTTCCAAACTCCATTATCTCACTCTTGAAGCCTACATCTATAATAATATTGGAGATACCAACTCCAGATCATTTCTTGGGAAGGTTTGCCTCACTGGAAATTCCTTTGTTCCCCTTTCTGATTCTGTTGTTTTGCACTATCCTCTAGAAAAGCGTGGCATCTTCTCGCATGTAAGAGGAGAGCTTGGCCTGAAAGTCTATATCACTGATGACCCTTCCATAAAATCTTCTACTCCACTCCCAGCAGCTGAAACCTTCTCAACTAAGGATCCCAGCATTACTCATACACATGCCCAACCAGTAGCAAATCCAGTCACTGGTGACACAGTTGAGTCAAGACACACCTTTCACCATCTCCCAAACCCAAATCACCATCAACATCACCATCAACATCATCCTTCCACTACAGTAGTTAACCGTCATGTGCCAAAGTATGAGGCTGATGAGATGAAATCTGAACCACAGCCTCCAAAGCTAGTTCACATGTACTCAGCAGCATCATCACAATCTGCTGACTATGCACTTAAAGAGACGAGCCCTTACCTTGGCGGTGGAAAGGTTGTTGGGGGTCGTGTTATACATGCAGACAAGACTGCAAGCACTTACGATCTCGTTGAACGGATGTACTTTCTCTACGTAAGAGTTGTTAAGGCTCGTGAACTTCCTGCCATGGATCTTACTGGGAGTATTGATCCATTTGTGGAGGTGAAAATTGGAAACTACAAAGGAATCACAAAACATTATGAGAAAAACCAAAATCCGCAGTGGCACCAGGTTTTTGCCTTTTCCAGGGACCGAATGCAGGCTTCTGTCCTAGAAGTTGTAATTAAGGACAAGGATCTAGTAAAGGATGACTTTGTGGGCATTGTGAGATTTGACATCAATGAGGTGCCATTGCGAGTCCCACCAGATAGCCCTCTAGCTCCAGAGTGGTACCGACTTGAGGATAAGAAGGGAGAGAAGATAAAGGGTGAGCTAATGCTCGCAGTTTGGATTGGGACTCAAGCAGATGAGGCCTTTTCCGATGCGTGGCATTCTGATGCAGCAACTCCTGTTGACAGCACACCAGCTATCACTGCAGTGATACGTTCAAAAGTCTATCATTCACCAAGGTTGTGGTATGTGCGTGTTAATGTTGTCGAGGCACAAGATCTGGTCCCAACAGAGAAGAACCATTACCCAGATGTCTATGTTAAGGCTCAAATAGGAAACCAGGTTCAGAAAACAAAGATATGTCAGGCTCGGACACTGAGTGCTGTTTGGAATGAGGATCTACTGTTTGTTGCTGCTGAACCTTTTGAAGATCATTTAGTTCTAACAGTAGAGGATCGTGTGGGTCCTGGAAAGGATGAAATAATTGGAAGGGTCATCATACCATTGAGTGCAATAGAGAAGCGAGCTGATGAGCGAATAATCCATTCCCGTTGGTTCAACTTGGAAAAGCCAGTTGCTGTGGATGTGGATCAGTTGAAAAAGGAAAAGTTCTCTAGCAGGATCCATCTGAGAGTATGTCTAGATGGTGGTTATCATGTTCTGGATGAATCTACTCACTACAGCAGTGATCTCCGTCCGACTGCAAAACAGTTATGGAGGCCATCCATTGGGATATTAGAACTTGGCATTCTAAATGCGGTGGGGCTTCACCCCATGAAAACACGAGATGGGAGGGGCACATCAGATACATACTGTGTTGCAAAATATGGTCACAAATGGGTCAGGACACGCACCCTCGTTGACAACCTGAGTCCAAAATATAATGAGCAGTACACATGGGAGGTTTTTGATCCTGCTACAGTTCTCACTGTGGGTGTATTTGACAACAGCCAGCTTGGGGAGAAGTCGAATGGGAACAAGGACCTAAAGATTGGCAAGGTTCGGATTCGCATTTCCACACTTGAAACTGGCCGCATTTATACACACTCTTATCCTTTGCTGGTTCTTCATCCTACTGGAGTTAAAAAGATGGGAGAATTGCATTTGGCAATACGATTTTCATGCACATCCTTTGCAAACATGCTTTATTTGTACTCACGACCACTTTTGCCAAAAATGCATTACGTGAGGCCCTTTAGTATCATGCAGTTAGACATGTTACGCCATCAAGCTGTTAACATTGTGGCAGCAAGGTTAGGCCGAGCAGAACCTCCACTTCGGAAGGAGGTGGTGGAGTACATGTCTGATGTGGATTCACACCTCTGGAGTATGCGCCGAAGCAAGGCAAATTTCTTCAGGCTTATGACTGTGTTCTCGGGATTGTTTGCTGTTGGGAAATGGTTTGCAGATATTTGCATGTGGAAAAACCCTATAACGACAGTGCTGGTTCATGTGCTCTATCTTATGCTTGCCTGCTTTCCAGAGTTGATTCTGCCCACAGTGTTCCTTTACATGTTTTTAATAGGGATATGGAACTACCGATATCGACCAAGGTACCCTCCCCACATGAATATAAAAATCTCACAAGCTGAAGCAGTGCACCCTGATGAGCTTGATGAGGAATTTGACACATTTCCCACAAGCCGAAGCCCTGAGCTGGTGAGAATGAGGTATGATCGACTGAGGAGTGTGGCTGGTAGAATTCAAACCGTAGTTGGTGACGTGGCAACCCAGGGGGAGAGGCTTCAGGCACTGATAAGCTGGCGAGACCCACGTGCCACTGCCATCTTTATTACATTTTGCCTTGTAGCTGCATTGGTGTTGTTTCTGACACCATTTCAGGTGATAGCAGCTCTGGCGGGGTTCTGGGTGATGAGGCATCCAAGATTTCGCCGTAGGCTGCCGTCCGTGCCAATCAACTTCTTCCGCCGATTACCAGCAAGGACAGATAGTATGTTGTAA

>Cicle10018651

ATGACGACGCCGTCTCAACCACCGCCGCAGCAACAGTTTACCGTACGCAAACTTGTCGTGGAAGTTGTGGACGCACGTGACCTTCTCCCCAAAGACGGCCAGGGAAGCTCCAGCCCCTACGTCATCGCTGACTTTGACGGTCAGCGGAAGAGAACGTCCACCAAATTTCGCGACCTAAACCCAGTATGGAACGAGCCGCTGGAGTTCATAGTCTCGGACCCCAAGAACATGGACTGCGAAGAGCTCGAAATCGAAGTCTACAACGACAAAAGGTACTGCAATGGCAGTGGCCGTAAGAACCATTTCCTCGGAAGGGTCAAGTTGTGCGGCAGCCAGTTTGCTAGGCGAGGAGACGAAGGCTTGGTGTATTTTCCGTTGGAGAAGAAGAGTGTTTTCAGCTGGATTCGAGGTGAGATTGGGTTGCGAATTTATTACTACGATGAGTTATCTGAGGAGGAGCATCAGCATCCACCACCGCCTCAAGACGAGCCGCCGCCGCCGCAGCCGCCTCAGCAACAGCCGGGGGTGTGCGTAGTTGAAGAGGGACGAGTTTTTGAAGTTCCAGGAGGACACGTGGAGGTTTGCCATCCTGTTCCAGAAATCTATCATGGCCAGCCGCCGCCACAGGCTCCTATCATCGAGGAATCTCAACCGCACGGGGTGCACGTGCAACCCGAACCGGTCCAAATTCCGCCTCATGATGAGCCGATTCCGACGGCGGTTCCGGCGGCCGAGATTAGGAAGATGCAAAGTGGATGTGCTGAGAGGGTTAATGTTTTGAAGAGACCAAACGGCGATTATTCACCTAAAGTGATTAATTCGAGTAAGCCTAATGGAGAAGTTCCAACGGAAAGGATCCATCCGTACGATCTCGTTGAGCCGATGATGTATTTATTTGTCAAAATCCGAAAAGCGCGTGGGTTGGTTCCAAACGAGGCCCCGTACGTGAAAATAAGAACCTCCAGCCACTACAGAAAATCAAAACTAGCGAGTTACCGAGCCTGTGACCCACACGACTCGCCCGAGTGGAACCAAGTTTTTGCGCTTTTTCACAACAAGAACGACTCCGTCAGCGCGACCTTGGAGATCACGGTGTGGGACTCGCCGACGGAAAATTTTCTTGGTGGTGTTTGTTTTGATCTCTCAGATGTTCCAGTGCGGGACCCACCGGATAGTCCCCTGGCCCCACAGTGGTATCGCCTTGAGGGCGAAGCTTCAGATCAGAATAACAGAGTGTCCGGCGACATTCAGCTCGCAGTGTGGATTGGAACTCAAGCCGACGAAGCGTTCCCTGAAGCGTGGAGCTCAGACGCTCCCTACGTGACTCACACGCGCTCCAAGGTTTATCAATCGCCCAAGTTGTGGTACCTGAGAGTTACTGTGATGGAAGCTCAGGACCTTTGCATTGCTCATAATTTGCCTCCGCTAACGGCGCCGGAGATTAGAGTTAAAGCACAGTTAGCGTTACAGTCTGCACGGACGCGGCGTGGCTCCATGAACAATCATAGTTCGTCGTTTCACTGGCACGAGGACGTGTTCTTCGTAGCCGCCGAGCCGTTTGAGGACTCGCTGATTTTGCTAGTCGAAGACCGTACAGCCAAGGACGCAGCGGCAGTGATACTGGGACACGCTGTGGTTCCTGTGAGCTCGATTGACCAACGAATTGATGAGCGCCACGTGGCAAGTAAATGGTTTCCATTGGAGGGTAGTTGTGGCCGGGGATGCGCACGGTCCTACTGCGGGAGAATTCAGCTAAAATTATGTTTGGAGGGTGGTTATCACGTGCTTGATGAGGCAGCTCACGTGTGTAGTGACTTTAGACCCACGGCTAAGCAGCTGTGGAAGCCGCCCGTGGGGATTTTGGAGCTGGGGATTTTAGGCGCGCGTGGATTGTTACCGATGAAAACCAAAAACGGAGGCAAAGGGTCCACTGATGCTTACTGTGTGGCCAAGTATGGTAAAAAGTGGGTGCGTACCCGAACCATTACTGACTGCTTTGATCCACGCTGGAACGAGCAGTACACGTGGCAGGTGTATGACCCTTGTACCGTCCTCACCGTTGGAGTATTTGACAACTGGCGAATGTTTGCTGACGCGTCGGAGGAGAGACCCGATTACCGCATTGGAAAAATTCGCATCCGCGTATCAACACTGGAGAACAACAAGGTGTATACAACTTCGTATCCATTGCTGGTATTGTTGCGGACCGGATTGAAGAAAATGGGCGAAATTGAATTGGCGGTTCGGTTTGTGTGCCCTTCAATGTTACCCGAAACTTCCTCCGTATATGGGCAGCCACTGCTTCCAAGAATGCATTACTTACGGCCGCTAGGGGTGGCCCAGCAGGAGGCATTGCGTGGAGCTGCTACAAAGATGGTGGCTGCGTGGCTAGACAGGTCGGAGCCACCACTGGGGCCTGAGGTTGTGAGATACATGCTGGATGCGGACTCTCATGCATGGAGCATGAGAAAGAGTAAAGCCAATTGGTTTCGGATTGTTGCGGTTCTTGCTTGGGCTATTGGGTTGGCTAAATGGTTGCACAATATAAGAAGGTGGAAGAATCCGGTTACTACGGTTTTAGTGCACGTGTTGTATTTGGTTCTGGTTTGGTACCCGGATTTGATTGTCCCAACTGGGTTCTTGTATGTGGTCTTAATTGGGGTGTGGTATTATAGGTTTAGGCCCAAGATACCGTCGGGTATGGATACAAGATTATCACAAGCGGAAACGGTAGACCCGGACGAGCTGGATGAGGAGTTTGATACAATACCGAGTTCAAAACCACCGGAAATCATCAGAATGAGGTATGACAGGTTACGCATGTTGGCTGCAAGAGTACAGACGGTATTGGGTGATTTTGCAACGCAGGGAGAGCGGGTGCAAGCTTTGGTGAGTTGGAGAGACCCAAGGGCCACTAAATTGTTCATTGGGGTATGCACTGTTATTACATTGGTACTCTATGTGGTGCCACCCAAGATGGTAGCCGTGGCATTGGGATTCTATTACTTGAGACACCCTATGTTTAGAGATCCCATGCCACCGGCTAGCTTGAATTTTTTCCGGCGGCTGCCGAGCTTATCGGACAGATTGATGTAA

>Cicle10018674

ATGGCGGAAAGTTGCAATAGAAAGCTGATCGTCGAGGTCTGCAACGCGAAGAACTTGATGCCGAAAGATGGCCAAGGGACGGCGAGTGCCTATGTGATAGTGGACTTCGACGGACAGAGAAGAAGAACGAAGACCAAGTTCAGAGATCTCAACCCGCAGTGGGACGAGAGGCTCGAGTTTCTCGTTCACGACGCGGAGTCCATGCCTACGGAAATCTTGGAGATAAATCTTTACAACGATAAGAAGACTGGCAAGAGAAGCACTTTTCTCGGAAAAGTTAAAATTGCTGGAAGCACTTTTGCGAAAGTTGGATCCGAGAGTTTGGTTTACCATCCCTTAGAGAAGAGGAGCGTTTTTTCGCAGATTAAAGGAGAGATCGGGCTCAAGGTTTATTATATAGACGAGGATCCACCGGCGCCCGCGCCGGAAGCTGCTGCTGTTGCAGAACCTGCGACAAAGCCGGAGGCCGCGGTGGAGGATAAGCCACCGGAGAAAGCTGTAGGAGAGGAGAAAAAAGAAGAGAAACCAGCGACGGTGGAGGGGAAGAAAGAAGAAGAGAAACCTAAAGTGGAGGAAAAACCTAAAGAAGAAAAACCACCGGAGGAGAATACAAATCCTAAGCCAGCAGAGGCTCCTCCGGCGGCAGCGGCAGTAGCGGCTACACCGGTTGAGTTGCAGAATCCTCCTTTAGCTCAAAGCGATAAACCGAGCAATGCAAAAGATAAAACCACAGTAACGGAGACAAAAACTCAAGAGCTTCGCCTTAACGAGCATGAGCTCCGGGCTCTAACAAGCGATCGGAGTCGCAGCGCTTACGATCTTGTTGATCGCATGCCGTTTTTGTATGTTCGAGTGCTCAAAGCTAAGCGAGCCGGTAACGTATCTAACGGCTCGTTGTACGCTAAGCTGGTGATTGGTACTCATAGTATCAAAACTAAAAGCCAGCCTGATAAAGACTGGGACCAGGTTTTTGCATTTGATAAAGAAGGCTTGAATTCGACCTCGCTTGAGGTTTCAGTTTGGAGCGAGGAGAAGAAAGAAAATGAAGAGTGCACTGAGAATTGTTTGGGAACGGTGTCGTTTGATTTGCAGGAGGTACCCAAGAGGGTGCCTCCGGATAGTCCTTTAGCTCCACAGTGGTACAGTTTGGAATCAGAGAAGTTACCGGGAAATGACGTTATGCTCGCCGTTTGGATAGGGACTCAGGCTGATGAAGCGTTTCAGGAGGCCTGGCAATCGGATTCCGGTGGGTTGATACCGGAGACTCGAGCCAAGGTGTACCTCTCGCCCAAGCTTTGGTATTTGAGATTAACGGTTATCCAAACCCAAGATTTGCAGCCTGGTTCGGGATCCGAGCCTAAGGTTAGGAGCCCTGAGCTCTATGTCAAAGGTCAGCTTGGAGCCCAACTTTTCAAAACGGGCCGGACTTCTGTGGGGCTTTCCCCATCCAGCTCCGCTAACCCGACTTGGAATGAGGATTTGGTTTTTGTTGCCGCTGAGCCGTTTGAGCCGTTTTTGGTGGTGACTGTTGAGGATGTGACCAATGGGTGCTCTGTGGGCCATGCCAGAATTCAAATGTCAACAGTGGAACGTAGGATTGATGATCGAGCGGAGCCAAAATCAAGATGGTTCAATTTGGTTGGTGATGAGACACGGCCGTACGCAGGGAGGATACACCTGCGAGTCTGTTTGGAAGGTGGCTACCACGTGTTAGATGAGGCTGCTCACGTGACTAGTGATGTTCGAGCCGCCGCTAAACAGTTAGCCAAGTCTCCTATTGGTTTACTGGAGGTGGGTATTCGAGGTGCCACTAATCTGCTTCCTGTGAAGACTAAAGATGGTACACGTGGCACTACTGATGCTTATGTGGTGGCCAAGTATGGGCCAAAATGGGTCCGTACCCGTACGATCCTCGATCGATTTAATCCACGGTGGAATGAACAGTACACTTGGGATGTCTATGATCCATGTACGGTGCTCACGATTGGTGTATTTGATAATGGACGGTACAAGCGTGATGAAGCAGGGAAGCCAGGGAAAGATGTTAGAGTGGGAAAAATACGTGTACGGCTGTCGACGCTTGACACAAATCGGGTGTATCTCAATTCATATTCCCTCACTGTATTGCTGCCAGGTGGGGCCAAAAAGATGGGAGAGATTGAGATTGCTGTTAGATTTACTTGTTCATCATGGCTAAACTTGATTCAAGCATATGCAACACCAATGCTACCGAGAATGCATTATGTGCGCCCACTGGGTCCAGCTCAACAAGACATACTGCGCCACACGGCTATGCGTATAGTCACGGCTCGGCTCGCACGCTCCGAGCCACCGTTGGGACAGGAAGTTGTTCAATTCATGTTGGATACAGACACACACGTGTGGAGCATGAGGAGAAGCAAGGCTAATTGGTTCCGGGTCGTGGGCTGTTTGACGCGTGCCGCAACTTTGGCGCGTTGGTTGGACGGGATACGCACGTGGGCACACACACCCACTACAATTTTAGTGCACGTGCTGCTGGTTGCAGTCGTGTTGTGTCCACATCTAGTGCTCCCCACCGTATTCATGTACGCCTTCTTGATCGTAGCGTTGAGATTTCGTTACCGCCAAAGGGTCCCACAAAACATGGACCCAAGGCTCTCTTACGTAGACGTGGTTGGCCCTGATGAGCTTGATGAGGAATTCGATGGGTTCCCCACATCGCGGCCCTCCGAAGTAGTCCGTATCCGATACGACCGGTTAAGGGCATTGGCAGGGAGGGCCCAGACCTTATTAGGTGACGTGGCAGCACAAGGGGAGAGATTGGAGGCATTGTTTAACTGGAGGGACCCACGAGCCACGTGGATTTTTGTGGTGCTTTGCTTAGTAGCTTCTTTGGTGTTTTATGCGGTGCCGTTTAAGCTGTTCGTCCTCGGTTCTGGATTTTATTACTTGCGTCATCCGAGGTTTCGAGGCGACATGCCGTCTGTTCCCGTCAACTTTGTACGGCGACTGCCGTCTCTTTCTGATCAAATTCTCTAA

>Cicle10014352

ATGCAGAAGTCTCCACAAGCTATAGACTTTGCTCTGAAGGAAACCTCCCCCAAGATTGGTGCGGGGTCTATCACAGGCGATAAGCTTTCATGCACCTATGACCTTGTCGAACAAATGCAATACCTTTATGTCCGGGTTGTCAAGGCAAAGGATTTACCTGGAAAAGATGTTACTGGTAGCTGTGATCCCTATGTTGAAGTAAAACTCGGAAACTATAAGGGAACTACGAAGCATTTTGAGAAGAAGTCCAATCCTGAATGGAATCAGTGTTTTGCTTTCTCGAAAGATAGGATTCAGGCTTCAGTTTTGGAAGTGTTGGTGAAGGACAAGGATGTTGTTTTGGATGATTTAATTGGCAGGGTAATGTTTGACCTCAATGAAGTGCCGAAAAGGATTCCTCCAGACAGTCCTTTGGCACCCCAGTGGTACAGATTGGAAGATCGTAAGGGAGATAAGGTTAAAACTGGAGAGCTGATGTTGGCTGTTTGGATGGGAACTCAAGCAGATGAGGCGTTTCCTGATGCATGGCACTCAGATGCAGCAACAGTCAGCGGTGAAGGAGTTGCAAATATTCGATCAAAGGTATATCTATCCCCCAAGCTTTGGTATGTGAGGGTCAACATTATTGAAGCTCAGGACCTGCTACCTAGTGATAAAAGTAGGTTTCCTGAAGTTTTTGTGAAGGCTATCCTCGGAAATCAGGCATCGAGAACTAGAATTTCTCAAAGTAAGACTATAAACCCAATGTGGAATGAAGATTTGATGTTTGTAGCAGCCGAGCCTTTTGAGGAACCCTTGATTCTGACCGTCGAAGATAGGGTAGCACCAAACAAGGATGAAGTGTTAGGTAAGTGTCTGATCCCATTGCAGGCAGTGCAGAGGAGGTTGGACCATAAGCCTGTGAACACTAGGTGGTTTAACCTTGAGAAGCATGTTATTGTAGACGGGGAAAAGAAAGAGACTAAATTTTCCAGCAGAATTCATCTGAGGATATGTCTGGATGGTGGATATCATGTTTTGGATGAATCGACCCACTACAGCAGTGATCTGAGGCCAACTGCAAAACAGTTGTGGAAACCCAGCATTGGAATATTGGAGCTTGGCGTTCTCAGTGCTCATGGGCTGACGCCAATGAAGACAAAAGATGGACGAGGAACCACAGATGCTTATTGTGTGGCCAAATATGGGCAGAAATGGGTGCGAACACGGACAATTGTTGACAGCTTCGGGCCACGATGGAATGAGCAGTACACTTGGGAAGTTTTTGACCCATGTACTGTCATTACTGTAGGGGTATTTGATAATGGCCACATCCATGGACAAGGTGGCGGTGGGAAGGATTCAAGAATTGGTAAAGTGAGGATTCGATTATCAACACTTGAAACTGATAGGGTTTACACACACTCTTATCCTCTTTTAGTTTTGCATCCTTCAGGGGTGAGGAAAATGGGCGAGGTTCAATTGGCCGTGAGGTTTACTTGCTCGTCTTTGATTAACATGCTGCATATGTACTCACAACCATTGCTGCCCAAAATGCACTACATTCATCCATTGTCTGTTATTCAGCTTGATAGCTTGAGGCACCAGGCTATGCAGATTGTCTCCATTAGGCTGAACCGAGCTGAGCCACCATTGAGGAAAGAGGTTGTGGAGTACATGCTTGATGTAGACTCTCATATGTGGAGTATGAGGAGAAGCAAGGCTAATTTCTTTAGAATAATGGGTGTTCTAAGTTCGTTGATTTCTGTTGGTAAATGGTTCGATCAAATCTGCAATTGGAAAAACCCTCTTACAACCATTTTGATTCACATCCTTTTCATCATATTGGTCCTTTATCCTGAGCTCATACTCCCCACCGTATTTCTCTACCTTTTCTTGATTGGCATTTGGAACTTCCGTTGGAGGCCAAGGCACCCTCCTCACATGGACACTCGATTGTCGCATGCCGAAGCTGCTCACCCTGATGAACTAGATGAAGAATTCGACACATTCCCCACTACCAAGGGATCAGACATTGTTCGCATGAGATATGACCGCCTGAGAAGCATAGCAGGGAGGGTTCAGACAGTGATTGGTGACCTTGCAACTCAAGGGGAAAGGTTTCAGTCTCTGATAAGCTGGAGAGACCCAAGAGCAACCACTCTTTTTGTCACTTTCTGCTTGATTGCTGCTATAGTCCTCTATGTTACTCCATTCCAAGTTGTTGCCCTCCTTGCAGGCATTTACGTGCTAAGGCATCCCAGGTTTCGCCACAAGCTTCCATCGGTTCCTCTCAACTTCTTCAGGAGGTTGCCAGCAAGGTCAGACAGCATGCTATAA

>Cusat038090

ATGAGTTCACCTGCAGCGGGCGACAAGGAAGCGGATTACAAGCTGAAGGACACGAAACCAAACCTTGGTGAGCGATGGCCGCATGGAGGGATACGTGGAGGGGGTGGGTGGATAACGAGCGAGAGAGCGACGAGCACATACGATCTTGTGGAGCAAATGTTTTATCTATACGTTCGGGTGGTGAAGGCAAAGGACCTACCGCCGGACCCGGTGACTGGGAGTTGTGATCCATATGTGGAAGTGAAGTTGGGAAATTACAAGGGAAGGACTCAACATTTTGAGAAGAAAACAAATCCCGAATGGAACAATCAAGTATTTGCTTTCTCTAAAGACAAGATTCAATCAACAGTTCTTGAAGTTTTTGTTAGAGATAAAGAGATGGTTCCAAGAGATCAATATGTTGGAAAAGTGGTGTTTGATTTGAATGAAGTGCCTACAAGAGTCCCACCGGATAGCCCATTGGCACCACAATGGTACAAGTTAGAAGATCGAAAAGGCGACACCAAGGTGAAAGGAGAGATAATGCTAGCGGTTTGGATGGGAACGCAGGCAGACGAAGCATTTCCTGACGCATGGCACTCTGATGCAGCATCGGTCCACGGGGAAGGCATCTATAATATTAGATCAAAAGTGTACGTTTCTCCAAAACTATGGTACCTAAGAGTGAACGTCATAGAAGCACAAGATGTTGAGCCACAGGACAAAAGTCAACCACCCCAAGCATTTGCAAAAATTCAAGTAGGAAAGCAAATATTGAAAACAAAGCTATGTTCAACAAAAACCACAAACCCAGTTTGGAACGAAGACCTAATCTTCGTCGTAGCTGAGCCGTTCGAAGAACAACTCGTTTTAACGGTCGAAAACAAAGTATCATCTGCAAAAGACGAGGTCGTGGGAAGATTAATAACACAACTAAACGGATTTGAGAGGCGTTTGGATCATAGAGTAGTTCATTCACGTTGGTTCAATCTTGAGAAGTTTGGATTTGGAACATTAGAGGGAGATAAAAGGCATGAATTGAAATTCTCAAGTAGGGTTCATTTAAGGGTATGTCTTGAAGGAGCTTATCATGTAATGGACGAATCAACAATGTATATAAGTGATGTAAGGCCAACAGCAAGGCAACTTTGGAAACAGCCAATTGGGATTTTTGAAGTCGGGATTTTGAGTGCTCAAGGGCTTCAACCAATGAAGAAGAATGATGGGAAAGGAAGTACAGATGCTTATTGTGTGGCAAAATATGGCCAAAAATGGGTTAGAACAAGGACAGTAACTGATAGCTTTAATCCAAAATGGAATGAGCAATACACTTGGGAGGTTTATGATCCTTGCACTGTCATCACAATTGGGGTTTTTGACAATTGCCATTTGGGTGGGAATGATAAGAATGATTCAAGAATTGGGAAGGTAAGGATAAGACTTTCAACACTTGAAATGGATAGAATCTACACCCATTCATACCCACTTCTTGTGTTGCAACCATCCGGATTGAAGAAAATGGGAGAGCTCCAACTAGCCGTGAGATTCACATGCCTTTCCCTTGCTCACATAATCTACCTCTATGGCCATCCCCTCTTACCTAAAATGCACTATCTCCACCCTTTCACCGTCAACCAACTAGATAGCTTAAGATTTCAAGCAATGAGCATTGTAGCCACGAGGCTCGCTCGAGCCGAGCCGTCTCTACGAAAAGAAGTTGTAGAATACATGTTAGATGTGGATTCTCACATGTGGAGTATGAGAAGAAGCAAAGCCAACTTCTTTCGAATAGTTGCTCTATTCTCAGGCATCATCTCTATGAACCGATGGCTCGGAGAAGTTTGTCAATGGAAAAACCCTATTACTTCTGTCCTCGTCCACATCCTCTACTTCATCCTTATATGTTTTCCTGAACTCATCCTCCCAACCACTTTCCTTTACATGTTTCTCATTGGCATTTGGAACTTTCGGTTTCGACCACGACATCCACCACACATGGACATAAAACTTTCTTGGGCTGAGGCAGTTCATGCAGATGAACTGGACGAGGAGTTTGATACTTTTCCAACTTCGAAGACGCAAGACGTTGCACGAATGAGGTATGATAGGTTGAGAAGTGTCGCCGGACGGATCCAAACGGTGGTCGGGGACATTGCAACTCAAGGGGAGAGGTTTAAGGCTTTACTTAGTTGGAGAGATCCAAGAGCTACGAGTCTTTATGTCGTGTTTTGCTTACTTGTGGCTATTGCATTGTATATTACACCGTTTAAGATTGTGGCTTTGGTTGCAGGTGTTTATTGGCTCAGACATCCTAAGTTTAGGAGCAAAATGCCATCAGTTCCTTCTAACTTCTTTAGGCGACTCCCGTCTCGAGCTGATAGCTTGCTTTAA

>Cusat050080

ATGGTGATTCCTCCGTATTTCGCTCTCAAAGAGACCTCCCCCAACATTAATGGGGGAAAATCATCTGTTGGCATTAGTACTGCTTTTGATCTTGTCGAACAAATGTTGTTTCTTTACGTCAAAGTCGAAAGAGCAAGAGATTTAACGGAAACTTGTGACCCTTATGTTGAAATCAAGCTTGGAAACTACAGAGGGACCACGAAAGCTTTTGAGAAAACACCAAATCCAGAATGGGGTACTGTTTTTGCCTTTGTGAAAGATCGAATTCAGACAACTGACGTTGAAATCTCTTTGTTTAATAAGTCAGGGGCCAATGCAGAGATCGGCAGCATTGTTATGAGCATTGCCGACGTTCCATTGAGGATACCACCCGACAGTCAGTTGGCTTCGCAATGGTATAAACTCGAAAATCGAAACAGCAATGGCAGTCGAGTCAGGGGTGAGTTGATGCTCTCTGTTTGGATGGGGACTCAAGCCGACAACCATTACTCAATAGCGTGGCATTCTGATGCAGCATCGGTCAGTGGCGACGGTGTCATCAACACACAATCGAAAGTTTACCAATCACCAAGGTTGTGGTACTTGAGAGTGAACATAATTGAAGCTCAAGATTTAGTAATCAATGACAAGAACAGAAAGCCAGAGGTTTTGATTGAAGCAAGACTTGGGATTATACAAATGATAAGCAGAATATCAGAGAGTAAGAATTTGAATCCAGTATGGAATCAAGACATGTTGCTTGTTGCAGCAGAGCCATTTGAGAAGAATTTAGAGCTTCGTGTGGTTGATAAGATTGGCCCTAATCAAATTGATGTTCTAGGTGTTTGTTATATTCCTCTAGAGAAAATTGAAGTGAGAAATGATAGTTCATCTGTTGAGAACAGATGGTACAATCTAGAGAGGCCCAATGGATTCAAGGCAGGGGACGAAGCAAAGGAAGTGAAGTTTGCAAGCAAGTTGCATTTGAGAGTCTCTTTAGATGGAGGGTATCATGTTCTTCATGAGCAGATTCAATATGCGAGTGATCTTCGAGCAACGTCGAAGTTGTTATGGCCGAAGTGCATTGGTGTTTTGGAGTTAGGTATCCTGAGTGCTTCGGGGTTGTCGCCAATGAAACAAAGAGAGAATCAAACTGACGCATTTTGCGTAGCAAAATACGGGCCGAAATGGGTGAGGACTAGAACAATTACTAATACTTCTGCTCCCAAATGGAATGAGCAATACATTTTTGAGGTTTATGATCCGTGTACTGTTTTAACTATTGGAGTGTTTGATAATGGTTATCTCCAAGGAGGGGATATAGGAAAGGATAGTCGCATTGGGAAGGTTCGGATTCGATTGTCCACACTCGAGACCAATCGAGTTTACACACATTCTTACCCTCTTGTGGCATTGCAAGCTTGTGGTGTGAAGAAGATGGGTGAGATTCAATTGGCAGTGAGGTTTTCTTGCTTATCTTTTATCAACATGTTGCAAACTTATGCTCAACCAATGCTACCTGAAATGCATTACACTCTCCCTTTGTCCATTTACCAAATAGAGCACTTGAGAGATCAATGCTTCAACATTCTTTCAGACCGACTAACACGTGCTGAGCCAAAGCTAAGGAGGGAGGTCATCTACTACATATTGGATGCAGACTCACACTTATGGAGCATAAGAAAATCCAAAGCCAACTTCAACCGAATTGCAGCGCTTTTCAAGTGGTTGGTTTTGTTTTGCAAATGGTTTGGTTGCGTACAAAGTTGGACAAATCCTACCTTAACCGTTGCAGTTCACATAATGTTTATACTCGTTGTGTTCTTTCCTAAACTAATCTTTCCCACTATCTTTTTTTACGGTTTCTTGATGGGTGTGTGGCGGTACCGTTATAGGCCGAGGCATCCACCACACATGGATACTGAGCTTTCGTACGCTTATGCGGTGACACCTGATGACTTAGAAGAGGAATTTGACACATTCCCGAGTAGAGCGAATGGAGGAGCATTGAGAAGACGATACGACAAGCTTCGATATATCGGGGGGAGAATGCAAGTGTTGATGGGGGATTTAGCAACACAAGGGGAGAGGATTGAAGGGGTTCTAAGTTGGAGGGATCCAAGAGCAACTGCACTTTTTATGATGTTTTGCCTTGTTGCAGCTGTGGGAATGTATGTAATTCCTTTCAATGTTCTTATCCTTTTAATGGGTCTTTATGCAATGAGACACCCAATATTTAGGATCACTTTACCTTCTTTCCCTCAAAACTTTCTTAGGAGAATGCCTGCTAGAATTGACTCTTTACTTTGA

>Cusat061600

ATGACCAAGCTCGTAGTAGAAATCCTCGACGCCGGCGACCTCATGCCCAAAGACGGCGACTCTGCCAGCCCCTTTGTCGAGGTTGATTTTGACGACCAAAAACAGAGAACTCATACCAAACATAGAGATCTCAATCCTTACTGGAATGAGAAGCTCCTCTTCAACATCTCCCACCCTAAAGATTTTCCCAACAAGACCGTCGATGTTGTGGTTTATAACGAGAGGAAATCCGGCCACCGCCGGGATTTTCTCGGCCGTGTTAGAATCTCCGGCATGTCGGTGCCTCTTTCTGAACAGGAAGCTAATGTTCAGCGATACCCACTTGACAAACGTGGCCTTTTCTCTCATATCAAAGGCGATATTGGATTTCGAATGTATATGATTCACGATGATGATTCGTCTTCCTTTTCTCCTCCTCCTCCTACCCATCCAGCGCCACCACAACCTCCCCATTTCGAAACGCCCCTGCAAGAAATCAATCCCAACATATTCGATCAGGAGGAACTGCAAGTCCCCACTAATGGATACGAGAGTGCGAAGGTCAAGAAGAAGAAGGAAAAGGACGTCAAGACCTTCCACTCCATAGGAACAGCTCCGGCTGCGGCTGCGACCTCGGTGGCTCCACCACCGACAGAATTCAAGCGGCCACCACCAATGGCAACCCGGATGGACTTCGCTCAAGCAGGTCCATCTCCGGCGACAGTAATGCATTTACCAATTCCAAAGCAAAATCCAGAGTACTCTTTGGTGGAGACCAACCCCCCGTTGGCAGCAAGATTGCGGTACGGCTACAGAGGAAAAGACAAGATCATCAGCACCTACGATATGGTGGAGCAGATGCATTTTCTGTATGTGAACGTGGTTAAAGCTAAAGATCTCCCTGTCATGGATGTTTCAGGGAGTTTAGACCCTTATGTGGAAGTGAAGGTGGGGAACTACAAAGGAGTCACAAAGCACTTGGAGAAGAATCAAAACCCAGTTTGGAAGCAGATTTTTGCCTTCTCAAAAGAGAGATTGCAAGCAAGCTTACTGGAAGTGATTGTGAAAGACAAGGATTTGGGGAAGGATGATTTTGTGGGTAGAGTTTTCTTTGATATCCCTGAGGTTCCATTGAGAGTTCCTCCTGATAGTCCATTGGCTCCTCAATGGTACAAACTAGTGGACAAGAAGGGAATCAAAGCAAAAGGGGAAGTGATGCTTGCTGTTTGGATGGGAACTCAGGCTGATGAGTCCTTCCCTGATGCTTGGCATTCTGATGCTCACAGCATCAGCCACAGCAACCTCGCCAACACAAGATCAAAGGTTTATTTCTCTCCTAAACTCTACTATCTAAGAGCCCAAGTAATCGAAGCTCAAGACCTCATTCCATCGGACAAATCCAAGCCCCCAGATACATTCGTGAGAATACAATTCTCCAATCAGGGTAAAGTAACCAAACCTTCTCAGATGCGAGTGATCAACCCAGTTTGGAACGAGGAGCTAATGTTTGTAGCATCCGAACCATTTGAAGATTTCATCATCATCTCTGTTGAAGATAGAGGAACAGGGGAGATTCTGGGAAGAGTGATAGTGCCGTCAAGAGATGTTCCACAGAGAATCGAGTCCACAAAACTCCCAGACGCCCGCTGGTACAATCTCCACCCTCCATACATCGCTAAATTAGAGGAAACAGAGAAAAAGAAGGAAAAGTTCTCCAGCAAGATCCATGTCCGTCTCTGGATCGATTCCGGGTACCATGTTCTAGATGAATCAACGCACTTCAGCAGCGATCTTCAGCCATCCTCCAAAGTCCTCAGAAAAGACAGCATCGGAGTACTCGAATTAGGGATTTTGAGCGCTCGGAATCTGCTACCAATGAAGAGCAAAGAAGGAAGAATCACAGATGCTTACTGCGTTGCCAAATATGGCAACAAATGGGTTCGAACCAGAACTCTATTAGACACCCTCGCCCCTCGCTGGAACGAACAGTACACTTGGGAAGTTTACGATCCTTGCACTGTAATCACAATTGGGGTTTTTGACAACGCTCACACAAACGGAAGCAAAGAAGACGCAAAAGACCAGAGAATTGGAAAAGTAAGGATTCGATTATCCACATTAGAGACAGACAAAGTATATACGCATTATTACCCTTTATTGGTTCTTCAGCCCTCTGGCCTGAAAAAACATGGAGAGCTTCAATTGGCTTTGAGATTCACCTGCACGGCCTGGGCCAACATGTTGACACAATACGGGAAGCCATTGCTCCCCAAAATGCACTACCTGCAACCAATCCCAGTCCGCCACATCGATCTACTCCGCTTCCACGCGATGAACATAGTGGCTGCAAGGCTGTCTCGAGCCGAGCCCCCACTCCGGCGAGAAGCGGTGGAGTACATGCTCGACGTCGATTACCACATGTTCAGTCTCAGAAGAAGCAAAGCGAATTTCAATCGCATAATGTCGCTTCTCTCAGGAATCACCGCAATTTACCGATGGTTCAACGATGTGTGCATCTGGAAAAACCCAATCACAACCTGCCTCGTGCATGTTCTGTTCTTGATTCTCGTTTGTTACCCCGAATTAATCCTCCCGACCGTCTTCCTGTACCTATTCGTAATCGGAATCTGGAATTACCGGTTCCGGCCGAGGTATCCGCCGCACATGGACGCGAGATTGTCGCAGGCGGAGCACACTCACCCAGACGAACTGGATGAAGAGTTCGACAACTTTCCAACGACGAAGCATATTGACACGGTGAGGATGAGGTACGACAGGCTGAGGAGTGTGGCCGGAAAAGTGCAGACGGTTGTGGGAGATTTGGCGACGCAAGGGGAAAGGGCTCAGGCGATTCTGGGGTGGAGAGATCCGAGGGCCACAGCACTGTTCATCATCTTCGCGTTGATGTGGGCGGTTTTCATCTACGTTACGCCGTTTCAGGTGGTGGCGATTCTGATCGGACTATACCTGTTCCGGCACCCGAGATTGAGGAGGAAGTTGCCATCAGTTCCTGTGAATTTCTTCAAGCGGCTGCCATCAAAAGCCGATATGATGTTATTATAG

>Cusat069790

ATGGCGGACAGTTGCGGCCGAAAGCTCTTCGTTGAGGTCTGTAACGCCAAGAATTTGATGCCCAAGGACGGCCAAGGAACGGCCAGTGCTTATGCGATTGTCGATTTCGAAGGCCAGCGGCGACGTACCAAGACGAAATTTCGAGATCTCAATCCTCAATGGGACGAGAAGCACGAGTTTCTTGTTCACGACATGGAGGCTATGGCTTCCGAGATTTTGGAGGTTAATTTGTATAATGATAAGAAGACGGGGAAAAGAAGCACGTTTCTTGGTAAGGTTAAGGTCGCTGGAACTTCTTTTTCCAAATCTGGATCGGAATCGCTTATTTATTATCCTTTAGAGAAACGTAGTGTGTTCTCTCAGATTAAGGGAGAATTAGGTCTCAAGGTTTATTATGTCGATGAAGATCCACCTGCTGGTGGTGCTGTTGCTGAATCTGAGCAGAAACCCGAGACTACTCCAGTAGCTGAGGAGAAACCCCCTGAGAATCAGGAGGGAAAGGAATCGAAAGTCAAGGAAGAGGAGAAGAAAGAAGAAGAGAAGCCGAAGGAAGAACCAAAAGCAGAGGAAAAGTCTAACGAAAATCCACCGGAAAATCCTAAACCAGAGGAATCCCCGGCTGTTGAACCTGAGAAGCCCGTAGAAGTGGAGAATCCGCCAATTGCACATACGGAAAAGCCAAAGCAAATGCGGAAGGCGAAATCGGAAACAGAGAAACTTGCCGATTTATCCGTCAATGATCTTGAGCTTCGTTCTGATCGAAGTCGTAGGGCGTACGATCTTGTTGATCGAATGCCGTTTCTCTACGTTCGAGTCGTAAAGGCAAAAAGAGAATCCTCCGATGGTGGATCCTCCTCCATGTACGCAAAGCTCGTGATCGGAACCCACAGTATCAAAACGAAGAGCCAGAGCGAGAAAGATTGGGATCAAGTATTCGCGTTCGACAAAGAAGGTTTGAACTCCACATCTCTTGAAGTATCTGTTTGGGCTGAGGAAAAGAAAGAGAACGAAGATCAGAAAGCGGAGAATTGTTTAGGAACAGTATCGTTTGACCTGCAGGAAGTTCCGAAAAGAGTACCGCCAGACAGTCCTCTAGCTCCGCAATGGTACTCTCTAGAATCTGATAAATCGCCGGGAAATGACGTCATGCTCGCTGTCTGGTTAGGAACTCAGGCTGACGAGGCGTTTCAAGAGGCTTGGCAGTCAGACTCCGGCGGGATGATACCGGAGACCCGAGCCAAAGTCTACCTCTCTCCGAAGCTGTGGTATCTGAGATTAACGGTCATCCAAACCCAGGATTTGCAGTTCGATTCGGCTTCCGAACCTAAGTCTCGAAACCTCGAGCTTTACGTTAAAGGTCAGCTTGGTCCACAAGTTTTCAAAACGGGAAGGACTGCTGTCGGCTCAGCTAACCCCACATGGAACGAGGATTTGGTATTTGTAGCAGCCGAGCCGTTTGAGCCGTTTTTGGTAGTCACGGTAGAGGACGTGACAAATGGAAAATCAGTGGGCCAAGCGAAAATCCACATGGCAAGCATCGAGAAGAGAACCGACGATCGGACAGAGACAAAGTCACGATGGTTCAATTTAGTCGGAGACGAAACCCGCCCTTACACTGGCAGAATCCACCTCCGAATCTGCTTAGAAGGCGGGTATCACGTGCTGGACGAAGCAGCGCACGTGACAAGCGATGTCAGAGCCGCAGCGAAACAATTAGCAAAGCCACCAATCGGACTCCTGGAAGTCGGGATTCGCAGCGCTAGTAATCTTCTCCCGGTGAAAACAAAAGATGGTACTCGTGGCACCATAGACGCTTACGTGGTAGCCAAATACGGCCCGAAATGGGTTCGAACACGCACGATCCTGGACCGGTTCAATCCACGCTGGAACGAACAGTACACGTGGGATGTATACGATCCTTGCACCGTCCTCACCATCGGCGTATTCGACAACGGTAGATACACCCGCCAAGAAAACGACGGCGTTTTAAAACAGCCCGGAAAAGACCTACGTGTCGGAAAAGTACGAATCCGATTGTCGAGTCTCGACATCAATCAGGTGTACTCAACAGCATACTCCCTTACGGTGTTACTCCCTACCGGCGCCAAGAAAATGGGAGACCTTGAAATTGCCGTCCGATTCTCCACCTTCTCATGGCTAAGCTTAATCCAATCATATTCAACCCCAATACTTCCAAGAATGCATTACATCCGTCCGTTGGGCCCAACCCAACAAGACATCCTCCGCCACACCGCTATGAGAATCGTTACAACCCGACTAGCCCGGTCCGAACCAGCCATGGGTCATGAAGTGGTTCAATACATGCTCGACTCAGACACACACGTGTGGAGCATGCGAAGAAGCAAAGCGAATTGGTTCCGTGTTATCGGTTGTCTCTCACGCGCCGTCGCCATCGCACGTTGGTTTGACGAAATCCGCACGTGGGTCCACCCACCAACCACCGTCCTCATGCACATTCTCTTAATCGCCGTCGTCTTATGCCCTAACTTAATCCTCCCAACTCTCTTCATGTACGCTTTCTTAATCCTGACCTTTCGATTCCGTTACCGTCACCGGACTTCCCACAACATGGACCCCAGATTATCGTACGTGGACTTCGTAAGCACCGACGAACTGGATGAGGAGTTCGATGGATTCCCGTCCGCACGATCTGCTGACCAGATTCGGGTGAGATACGACCGACTGAGAGCACTTGGAGGTAGGGCCCAAGTGTTGTTGGGTGACGTGGCGGCTCAAGGGGAGCGTTTGGAAGCGTTGTTTAATTGGAGGGACCCTCGAGCCACGGGAATATTTGTGGTCATTTGTTTGGGAGCCTCGCTTTTGTTCTATGCGGTGCCCTTTAAGGCTTTTCTCTTGGGATTTGGATTTTATTACTTCCGCCATCCCCGGTTTCGTGGCGATATGCCATCCGTTCCGGCTAATTTTTTCCGGCGTTTGCCCTCTCTTTCCGATCAAATGATCTGA

>Cusat097930

ATGACGACGACGACGACGCCGCAGCCACAGTCACAGCCACCGCCATCGCCATCGCCATTGCCGCCGGCGCTGGTCAAAACAGTTAGAAAGCTTGTGGTCGAAGTTGCTGATGCTCGCAACCTTCTTCCTAAAGATGGCCAAGGAAGCTCCAGTCCGTATGTTGTCGCTGATTTTGATGGCCAGAGGAAGCGTACCGCCACTAAGTTTCGTGAGCTTAATCCTGTGTGGAATGAGCCGCTGGAATTCATTGTCTCTGATCCTGACAATATGGACTACGAGGAGCTTGATATCGAAGTTTTCAATGATAAGAGGTATGGCAATGGGAGTGGCCGGAAGAATCACTTCTTGGGGAGGGTGAAGTTGTACGGAAGTCAGTTTGCGAAGAGAGGGGATGAAGGTTTGGTTTACTATCAATTGGAGAAGAAGAGCGTGTTCAGCTGGATTAGAGGCGAAATTGGGCTTAGAATCTGTTATTACGATGAGTTGGTGGAAGAAGCTCCGCCGCCGCCTCCGCCGCAGGAGGAGCAACCACCTCCTCCAACTGAGAAGCCTAAAACTCCGGAAGCTGTAGTCGAGGAAGTGAGGATGTTCGAGCTTCCGCCACAGGGGGAGGTCGGTCGCGATGATTCGAATTCACCCCCGGTGGTGGTCATAGAAGAGTCGCCGCGGCAGGATATGCCGGTACATTCTGAGCCACCGCCGCCGGAGGTAAATGGTCCTCCGCCAGGGGAGGGGCAATTTGCACCGGAAATGAGAAGGATGCAGAGTAACAGAGCAGCAGGATTCGGGGAAGGGATTAGGGTTTTGAGAAGGCCGAATGGAGATTATTCTCCGAGAGTAATCAATAAGAAATACATGGCTGAGACGGAGAGGATTCATCCATATGATCTTGTGGAGCCGATGCAGTACCTCTTCATCCGTATTGTGAAAGCTAGAAATCTCGCTCCTAATGAGCGCCCTTACTTACAGATTCGCACATCAGGCCATTTCGTGAAATCGGATCCAGCTAATCATCGGCCTGGTGAGCCGACTGAATCGCCGGAATGGAACCGTGTCTTTGCCCTCCGTCATAGCAGGCTTGATACGGCAAATACAACGCTGGAGATTGCCGTCTGGGACACGTCATCGGAGCAGTTCCTCGGCGGCGTTTGCTTTGATCTTTCCGATGTACCAGTACGAGATCCGCCCGATAGCCCTCTGGCCCCTCAGTGGTACCGCCTCGAAGGCGGCGCCGGAGACCAACAACCATCCAAAATTTCTGGCGACATTCAGCTCTCTGTTTGGATCGGAACTCAAGCCGACGACGCATTTCCAGAAGCTTGGTGCTCCGATGCGCCACACGTGGCTCATACACGCTCGAAGGTCTATCAATCTCCCAAGCTATGGTACTTGCGAGTATCAGTGATAGAAGCGCAGGATCTTCACATTGCTTCAAATCTGCCTCCATTAACGGCACCGGAAATCCGAGTCAAAGCCCAACTGAGTTTTCAGTCGGCTCGGACCAGGCGAGGTTCCATGAACAACCACAGCGCCTCATTTCACTGGAACGAGGACCTTGTCTTCGTTGCCGGTGAGCCTCTTGAAGATTCCCTGATCTTACTTGTTGAAGACCGAACAAGCAAGGAGGCCATACTCCTCGGCCACGTCATGATTCCAGTGGACACAGTGGAACAACGGTTCGATGAGCGATATGTGGCGGCGAAATGGTATTCCTTAGAAGGCGGCAATGGTGGTGAAACATACAGCGGCAGAATCTATCTCCGACTCTGTTTGGAGGGTGGATATCACGTGCTAGATGAGGCGGCACACGTGTGCAGCGATTTCCGGCCAACGGCGAAGCAGCTGTGGAAGTCGGCTGTTGGAATTCTGGAGCTGGGGATTCTCGGAGCACGGGGGTTGCTTCCGATGAAGACGAAAGATCCAGGGAAGGGGTCCACCGACGCTTACTGCGTCGCAAAGTACGGGAAAAAGTGGGTCCGAACCAGAACGATGACGGACAGCTTTGATCCACGTTGGAACGAACAGTACACGTGGCAAGTTTATGACCCTTGCACTGTTCTCACCATTGGCGTCTTCGACAACTGGCGAATGTACTCGGACGCGTCGGAGGACAAGCCCGATTACCACATTGGAAAAGTTAGGATTCGGGTGTCAACCCTTGAAAGCAACAAAATCTACACAAACTCGTATCCTCTGTTGGTGTTGCAGAGAACAGGGTTGAAGAAAATGGGTGAGATTGAGCTAGCCGTCCGGTTCGCTTGTCCGGCATTATTGCCGGATACATGTGCAGTTTATGGCCAGCCATTACTTCCAAGAATGCACTATCTCCGTCCTCTAGGGGTGGCTCAACAGGAGGCTTTACGCAGAGCCGCCACAAAGATGGTGGCAACTTGGCTAGGCCGGTCGGAGCCACCATTGGGCTCGGAGGTGGTTCGATACATGTTGGATGCAGATTCACACGCTTGGAGTATGAGAAAAAGCAAGGCGAATTGGTTTAGAATTGTGGCAGTTTTGGCATGGGCGGTTGGATTGGCCAAATGGTTGGATGATATCCGAAGATGGAGGAACCCCATCACCACAATGCTTGTTCATATACTGTATTTAGTGCTCGTTTGGTACCCGGATTTGATTGTCCCAACTGGGTTTCTCTATGTGTTCTTAATTGGAGTGTGGTACTACCGGTTCAGACCGAAGATACCCGCTGGAATGGACACTCGGCTGTCGCATGCTGAGGCAGTGGATCCAGACGAACTAGACGAGGAATTCGACACAATTCCGAGCTCAAAGCCACCGGACATAATCCGGGTAAGGTACGATCGACTAAGGATACTAGCTGCTAGAGTTCAAACAGTGTTAGGGGATCTTGCTACTCAAGGGGAGAGGGTACAAGCGTTGGTCAGTTGGAGGGACCCTCGAGCCACAAAGCTGTTCATTGGGGTATGTTTCGCCATCACATTGATCCTCTATGCGGTGCCGCCCAAAATGGTGGCAGTTGCACTCGGATTCTACTACTTACGTCACCCCATGTTCCGGGATCCTATGCCATCGGCGAGTCTGAACTTTTTCCGACGACTTCCGAGCCTATCGGACCGGTTAATGTAG

>Cusat135440

ATGGGTAACCTCAAGCTAGCTGTTGATGTAGTGGGTGCTCATGATCTTATGCCAAAAGATGGACAGGGCTCAGCCAATGCTTTTGTAGAGCTTCACTTCGATCGTCAAAGAGTTCGTACCACGACAAAGGAGAAGGATCTCAATCCTGTTTGGAACGAGAGCTTCTACTTTAACATATCAGATCCACAAAATTTGGCTAACCTTATTCTGGAGGCCTTTATCTTTACCTATAACAAATCAAGCATCAGCTCGAAGCCCTGCTTTCTTGGAAAAGTTCGTCTTACTGGGACCTCATTTGTTTCGCACTCTGATGCTGCTGTTTTTCACTATCCTTTGGAAAAACGAGGCATTTTTTCACGAATAAAAGGAGAACTTGGCCTTAAGGTCTATGTAACTGACGATCCTTCTCTAAAGCTTTCAAATTTACTTCCTGCAGCGGAACCTTCTGTGGAAAAGGATCCTCTTCCAGTTCCAATCACATCTGAACACCAATCAACAATACGAAAAGTTCCAAAGTTTGTAGCAAGTTTATTTTCTACCGACAAAACTGAATCAAGACAAACATTCCACCACCTTCCCAATGAGAAGCAGTCTCAACAAGATACGCCACAAGCTAGTGTACCAGCTGTGACCTATGGAGGATACGGTATGAATTCTAATCCAATGGTAGTAAATAATGTTCAGGCATATCCAGGGTCACCATTTCATTATAATGATTATTCAATAAGAGAGACGAGTCCTTACCTTGGCGGTGGAATGGTTGTTGGGGGTCGCCTTGCACTTCGAGACAGGCCTACAAATACCTATGACCTTGTAGAAAAGATGCATTATCTTTTTGTTCGAGTTGTGAAAGCCCGTGATCTTCCCACCAAGGATTTGACTGGAGGCTTGGATCCTTATGTTGAAGTGAAACTAGGGAACTTTAAGGGAACTACAAAGCATTATGAGAAAAATTCAAGTCCCGAATGGAATGAGGTATTTGCCTTCTCAAGGACGGATGTGCAATCAACAGTTCTGGAAGTTACTCTTAAAGACAAGGATCACATAAAGGATGATTATGTTGGACGTTTGTACTTTGATCTTCATGAAGTTCCTACTCGAGTTCCACCTGATAGTCCATTGGCTCCTGAATGGTATCGCCTCGAAGACAAGAGTAGATCAAAGAAAAAGGGAGAGCTAATGCTTGCTGTATGGTACGGCACACAAGCTGACGAGGCTTTTCCAGATGCTTGGCATTCTGATGCTATCTCCCCTACTGATTATACTTCGGTCATCCCAGCATATATTCGCTCCAAAGTTTATCATTCACCTAGATTGTGGTACGTCCGTGTCAATGTTGTTGAAGCTCACGATTTGGTTGTACAGGAGAAGTCTCGTTTCCCAGATGCATATGTGAAGGTACAAATTGGCAACCAAGTTCTACGAACAAAACCGGTGAAAACTCAATCAATGAATGCCTTCTGGAATGAAGATCTAATGTTTGTTGCTGCTGAACCCTTCGATGATCATTTGATCCTTTCTGTTGAGGACCATGTTGGTCCCAACAAGGATGAAACGCTTGGGAGGGCTGTTATTCCGCTGAGTTCTGTAGAAAAGCGTGCTGATAGTCGACCTATCCGCAGCCGATGGTACGACCTTATGAAGTCTATGTCAGATGCCGTGGAAGCAGGGGAGGGGAACAAAGACAAGGATAAGGATAAGGATAAGTTTCATAGTAGACTCCATCTTCGCATTTGTCTGGAGGGTGGATACCATGTGCTCGATGAGTCGACTCACTATAGCAGTGACCTCAGACCCTCACTGAAGCAACTTTGGAAGCCACCAATAGGTATATTGGAGCTTGGCATCCTGGCGGCGGATAAGCTTCATCCAATGAAAAATAGGAATGGGAAGGGCACAACTGATACATTTTGTGTGGCGAAGTATGGTCAGAAATGGGTTCGAACTCGAACAATAATTGACAATTTAAGCCCCAAGTTCAATGAGCAGTACCATTGGGAGGTTTTTGATCCTTCCACAGTCTTGACTGTTGGTCTTTTTGACAATGGTCATATTGGTGAATCCAGCAGTAACAGGGACACAAAAATTGGGAAGATTCGAATTCGTATTTCAACTCTCGAAACCAGTCGCATTTATACACATGTATATCCATTGCTTGTTCTTCACCCTTCTGGTGTCAAGAAGATGGGTGAACTGCACCTTGCTCTAAGATTTTTGTGCCCATCGGTTATGAATTTGATGTCTATGTATTCACGGCCTCTATTGCCAAAAATGCATTACATAAGGCCATTAGCGTTGTCTCAACAAGAACCACTTCGACATCAGGCAGTCAACATTGTAGCTGCTCGATTTAGCAGAGCAGAGCCTTCTCTGAGGAAGGAGGTAGTCGAGTACATGTCTGATGTAGACTCTCATCTTTGGAGCATGAGACGAACCAAGGCCAACTTCTTCCGGATTGTAGCAGTTTTTTCGGGATTACTTGCAATTGGAAATTGGTTTGGAGAAGTGTGCATGTGGAAGAACCCCATTACTACAGGGCTTGTTCATCTTCTTTTTTTGATGCTAGTTTGTTTCCCTGAGCTGATCTTGCCCACAGTTTTCCTCTACATGTGTGTTATAGGAATTTGGAACTATCGGTACCGTGCTCGGAACCCTCCACATATGGACACGAAACTCTCTCACGCAGAGGCAGTGAACCCAGACGAGCTTGACGAAGAATTCGACTCGTTTCCAACAAGTCGAAGCCCAGACATAATTCGAATGAGGTATGACCGGATGAGAAGTTTGGCAGGAAGAATCCAAACTGTAATGGGAGACGTGGCAACACAAGGAGAACGAATTCAAGCACTCTTAAACTGGCGAGATCCTCGTGCAACATGCATATACATAATATTTTGCTTCATTGCAGCTCTTGTGCTGTATGTAACACCATTCCAGATGTTGTTCCTTCTAACTGGTTTCTACGTAATGAGGCATCCTAGGTTCAGAAACCGAATGCCACCGGTGCCGATGAACTTCTTCCGCAGGCTGCCTGCTAGGACGGATAGTATGTTGTAA

>Cusat153570

ATGCAGAAGATTCTTCAACCTCATGATTTTGCTCTGAAGGAGACGTATCCTAAAATAGGTGCAGTCTCGATAACGGGAGACAAGCTCTCTTCCACGTATGATCTTGTGGAGCAAATGCAATATCTTTATGTCTATGTGGTCAAAGCTAAAGATTTACCTGGAAAAGATGTTACTGGTAGTTGTGATCCCTATGTGGAAGTGAAACTTGGAAACTATAAGGGAACAACCAAACACTTTGAGAAGAAGTCCAACCCTGAGTGGAAACAAGTTTTTGCTTTCTCGAGGGAACGAATCCAAGCCTCTCTTTTGGAAGTGGTGGTGAAAGACAAGGATTTTGTAGTAGACGATTTCATGGGGCGGGCTATTTTCGATCTCAATGATGTTCCAAAACGTGTCCCTCCTGATAGTCCACTGGCACCGCAATGGTATAGGCTGGAGGACCGAAAAGGGGATAAGGTAAAAGGAGAGCTTATGTTGGCTGTGTGGATGGGGACTCAAGCAGATGAAGCATTTCCTGATGCCTGGCATTCAGATGCTGTAACCGTGGGTGCTGATGCCATTGCTAGCATCAGATCAAAGGTTTATCTTTCTCCCAAACTTTGGTATGTTAGAGTGAACATCATTGAAGCTCAGGATTTACTACCGAGCGATAAGAGTCGGTATCCAGAAGTTTTTGTGAAAGCTATTCTTGGGGCCCAGGCTCTAAGAAGTAGAATATCTCAAAGCAAGTCTATAAATCCAATGTGGAATGAGGACTTAATGTTTGTGGCTGCTGAACCATTTGAAGAGCCACTACTTCTGACAGTTGAAGACAAGGTAGCATCAAATAAAGACGAAATTCTTGGGAGGTGTTTGATTCCCCTGCAAAATGTGCAGAGGAGATTAGATCATAAACCTGTAAATACTAGATGGTTCAATCTTGAGAAACATATCGTTGCAGATGGTGAAAAGAAAAAGGAAGTCAAGTTTGCCAGTAGGATTCATCTAAGGATTTGTTTGGATGGTGGGTATCATGTGTTGGATGAATCAACCCACTACAGTAGTGATCTTAGGCCTACTGCAAAACAGTTGTGGAAGTCTAGCATTGGGATTCTAGAGATGGGGATTCTAAGTGCTCAAGGGCTGATGCCGATGAAGACGAAAGATGGCAGAGGGAAGACAGATTCGTACTGCGTTGCAAAATATGGACAGAAATGGATTCGGACAAGGACTATTGTGGACAGCTTCAGTCCAAAGTGGAATGAACAGTACACTTGGGAGGTTTTTGATCCCTGTACTGTTGTTACTGTTGGGGTCTTTGACAATGGTTATATAGGTGGAGGAAGTGGAGTAAAAGATTCAAGGATTGGAAAGGTGCGGATTCGGCTATCGACCCTTGAAACTGATAGGGTTTACACTTATTCATATCCACTTCTGGTCCTTCATTCTTCAGGAGTGAAGAAAATGGGTGAAGTGCAGTTAGCTGTAAGGTTTACTTGTTCATCTTTGGTTAACATGTTGCATATGTACTCCAACCCATTGTTGCCAAAAATGCATTACATTCATCCATTATCAGTGATTCAACTTGATAGCTTAAGGCACCAGGCTATGCAAATTGTCTCGATGAGGTTAGCGCGTGCTGAGCCTGCATTGAGGAAAGAGGTTGTGGAGTATATGCTGGATGTAGATTCACATATGTGGAGCATGAGGAGAAGCAAAGCCAATTTCTTCAGAATAATGGGCGTTTTAAGTGGATTCATTGCATTTGGTAAATGGTTTGATCATATTTGCCACTGGAAGAACCCTATAACAACAATACTAATCCACATCCTTTTCATCATTTTAGTTCTTTACCCCGAGCTCGTACTTCCAACCATCTTTCTTTACCTTTTTGTTATCGGTATTTGGAATTTCAAGCATAGGCCTAGACACCCCCAACACATGGACACTAGGTTGTCTCATGCTGATGCAACTCATCCTGATGAACTAGATGAAGAATTTGATACATTTCCTACATCCCGATCTTCCGACACCGTTCGGATGAGATACGATCGCCTACGGAGTATAGCCGGGAGAGTGCAAACTGTGGTCGGGGATCTTGCAACTCAAGGAGAAAGATTTCAGTCACTATTGAGCTGGAGAGACCCAAGAGCAAGTGCTCTCTTTGTAACATTTTGCTTGATTGCTGCTATAATTCTTTATGTGACCCCATTTCAAGTTATTTGCCTTGTTGGAGGTATTTATGTTCTGAGACATCCAAGATTTCGACATAAACTTCCTTCAGTTCCTTCCACCTTCTTTAGAAGATTGCCTGCAAGATCAGACAGCTTGTTGTGA

>Cusat195010

ATGCAGCTGGTGGTGGAAGTAATAGATGCTCATGATCTTATGCCCAAAGATGGTGAAGGATCTGCAAGTCCATTTGTAGAAGTTGATTTTCAAAACCATATAAGTCGAACAAAAACGGTTCCAAAGAGTCTCGATCCCATTTGGAATCAGAAACTATCTTTCGATTTTGACGAAACCCAAAACCATCAGTACCAAACCATCGATATCTCAGTTTATCATGAAAAGAGATTGATTGAAGGCAGAAGCTTTCTTGGAAGAGTTAGAATTTCTTGCTCTAACATTGCCAAGGAAGGTGAAGAAACTTATCAGAGATTCCATTTAGAAAACAACTGGTTTCTTTCGGCTGTCAAAGGTGAGATTGGCTTGAAAATCTATATTTCACCACCAAAAAAATCTCCAATAAATCCACGAGAACCCCCAATTTCTAATCCTCCTCCAACCAGAGTGGTTTCAAATCCTCCTATTTCTTCAGCATTAGCTGCAGTAACTAAAGCAGACGGTGTTCCTGTTAGTGATATTCAAGAAGAACCAAAGAAAGATGTTTTAAAAATCTCACCATCTAAAGATTCAAACTCAACTCTTCCTGTAGTTGAGTTTCGTATCGAAGATCCTGCTAAAGAGCCAAAGGAAGAGATTGAAGAACCAATTGAAGCAAGACAAGAGACAACACAACTACACAAGCAGCAAACAATGCAGCGGCCTCGAATAGTAGTACAGAGACGACCACAAGGCGCTTCATCTTCGATGAACAGAAGCATTCCACCAACAATGAACACAAGCAATTCAGAAGCTAATTCCAGCAATCAGGATGACTATGAGATCAGGGACACTAATCCTCAGCTCGGGGAGCAATGGCCGAATGGAGGAGGGTATGGTGGAAGAGGTTGGCTGAGTGGTGAGCGACACACAAGCACCTATGACCTTGTTGAGCAAATGTTCTATCTCTATGTTCGTGTAATGAAAGCAAGGGATCTACCTTCCAGTTCTATCACTGGAGGTTGTGATCCTTATGTGGAAGTGAAGCTCGGAAACTATAAGGGGAGAACAAAGCATTTTGACAAGAAGCAAAATCCAGAATGGAATCAGGTCTTTGCTTTCTCAAAAGAACGCATACAGTCTTCTGCACTTGAAGTTTTTGTCAAGGACAAAGAAATGCTCGGAAGAGATGATTACCTGGGCCGAGTGGTGTTCGACTTAAATGAGGTTCCTACTCGAGTTCCTCCGGATAGTCCATTGGCTCCTCAATGGTACAGATTGGAGGACCGGCGAGGAACAGGCAAGGTAAGGGGAGAAATCATGGTTGCAGTTTGGATGGGAACACAAGCTGATGAAGCCTTCCCAGAGGCATGGCATTCCGATGCAGCCTCGGTCTTTGGGGAAGGTGTTCATAATGTAAGATCAAAGGTTTATGTCTCTCCAAAACTATGGTACCTAAGGTTAAATGTGATTGAAGCTCAAGATGTAATCCCCAATGACAGAAACCGCCTTCCAGATCTTTTTGTCAAAGTTCAGGTTGGCAATCAGGTCCTAAGAACAAAGATCAGTTCAACAAGCACTACAAATCCAGTTTGGAATGAAGATTTGGTATTTGTGGTAGCAGAGCCTTTTGAAGAACAGTTGGTAATCACTATTGAAGACAGAGTACACCCTTCAAAAGAAGATGTCTTAGGGCAGATCAGTCTCCCTCTTGACACGTTTGATAAGCGGCTAGATTATAGACCAGTCCATTCACGCTGGTTCAATCTTGAGAAGTACGGTTTTGGAGTCCTAGAAGCGGATAGGCGAAAGGAACTCAAATTTTCAAGCAGGATTCACTTGAGAGCTTCTCTTGAAGGTGGGTATCATGTACTAGATGAATCAACTTTATACATCAGTGACCAACGACCAACTGCAAAACAGCTGTGGAAACCACCAGTGGGAATATTGGAGGTAGGAATATTAAGTGCTCAAGGACTACTTCCAATGAAGATGAAGGATGGGAGAGGAAGCACAGATGCCTATTGTATTGCGAAGTACGGCCAAAAATGGGTTCGTACTAGAACAATTCTCAATACTTTCAGTCCCAAATGGAATGAACAATACACATGGGAAGTCTATGATCCATGTACAGTCATTACTTTGGGAGTTTTTGACAACTGCCATTTAGGGGGTGGTGAAAAACACAATGGAAGCAATGGAGCAAAAGATTCGAGGATTGGAAAGGTTCGAATTCGACTATCGACACTTGAAGCTCACAAACTCTACACTCATTCTTATCCCCTTCTGGTTCTACACCCCAATGGAGTAAAGAAGATGGGGGAGCTTCAACTTGCGGTTCGCTTCACCACTCTATCTTTGGCAAACATGATATACATCTATGGAAACCCATTGCTGCCAAAGATGCATTACCTTCAACCTTTTACAGTGAACCAAATAGAAAATTTAAGGTATCAAGCTATGAACATAGTAGCAACAAGGCTCAGTCGAGCTGAACCGCCTCTAAGAAAAGAAGTCATCGAGTACATGTTAGATGTCGATTCACATGTATGGAGCATGAGAAGAAGCAAAGCTAACTTTTTCCGAATTATGTCACTGCTTTCAGGAATGATTTCAGTTACTCGATGGTTTCGTGAAGTTTGCAATTGGAGGAACCCCATCACATCTGTGCTTGTGCACATTTTGTTCCTTATCTTGATTTGGTATCCAGAACTAATACTTCCCACTGTTTTTCTATACATGTTCCTCATTGGCCTATGGAACTACAGGTTTAGGCCAAGGCACCCACCCCACATGGACACCAAGCTCTCCTGGGCTGAAGCAGTAAACCCAGATGAGCTTGATGAGGAATTTGACACGTTCCCAACTTCCAAACCGAATGACTTAGTTCGACTAAGGTATGACAGGTTAAGAAGTGTTGCAGGGAGGATCCAGACGGTTGTAGGGGACATAGCAACACAAGGAGAGAGAGTTCAGTCTCTGCTCAGTTGGAGGGACCCTAGAGCCACCAGTCTTTTCATAGTATTTTGTCTTTGCACTGCTGCTGTGCTATATGCTACCCCTTTCAGAGTGGTGGCTTTGGTTGCAGGCTTATATTGTTTAAGGCATCCGAAGTTCCGCAGCAAGCTACCATCAGTACCTGGCAATTTTTTCAAGAGATTGCCTCCCCAAACAGACAGTTTGCTATGA

>Cusat242860

ATGCAGAGGCCACCACCAGAAGATTTTCTATTGAAGGAGACCAATCCCCATCTTGGTGGGGGGAAGGTCACTGGAGATAAGCTCACGAGCACGTATGATCTCGTTGAGCAAATGCAGTATCTCTATGTCCGTGTTGTCAAAGCGAAAGACTTGCCTGGAAAGGATGTTACTGGCAGCTGTGACCCTTATGTAGAGGTGAAGCTTGGGAACTACAAGGGTACAACTCGACATTTTGAGAAGAAGTCGAATCCTGAGTGGAGCCAGGTTTTTGCCTTCTCAAAAGACCGGATTCAGTCTTCAGTGCTTGAGGTTACTGTAAAGGATAAAGATTTTGTGAAGGATGATTTTATGGGTCGTGTGTTGTTTGACATGAATGAGATTCCAAAGCGTGTTCCACCCGACAGTCCATTGGCTCCTCAATGGTATAGACTTGAAGATAAGAAAGGCGATAAACTGAAAGGAGAGCTAATGTTGGCCGTTTGGATGGGAACTCAAGCTGATGAAGCATTTCCTGAAGCATGGCATTCAGATGCTGCAACTGTCAGTGGAACTGATGGTCTAGCAAATATTCGATCGAAGGTGTATCTTTCACCTAAGCTCTGGTATTTAAGGGTTAATGTTATTGAAGCTCAGGACTTACAGCCTACTGATAAGGGTCGATACCCTGAAGTTTTTGTGAAGGCTGTCCTGGGAAATCAAGCTTTGAGAACAAGGATTTCCCAGAACAGAACAATCAATCCATTGTGGAATGAGGATTTGATGTTTGTAGCTGCTGAACCATTTGAGGAACCCTTGATTTTGAGCGTTGAGGACCGAGTTGCTCCGAATAAGGATGAAACCCTTGGTAGGTGTGCGATTCCTTTGCAATATGTGGACCGGAGATTGGATCATAAACCTGTGAACAGCAAATGGTACAATCTGGAGAAGCATATTATCCTTGAAGGAGAAAAGAAGAAAGAAATCAAGTTCGCTAGCAGAATTCATATGAGGATCTGTTTGGAAGGTGGTTATCATGTTTTGGACGAATCAACACACTACAGCAGTGATCTTCGTCCTACTGCCAAAGTATTGTGGAAGCAGAGCATTGGAGTGTTGGAATTAGGTATCCTCAATGCTCAGGGATTGATGCCTATGAAGACTAAAGATGGTCGTGGGACAACAGATGCATATTGTGTGGCGAAATACGGGCAGAAGTGGGTTCGAACTAGGACGATCATTGATAGCTTCACACCCAAGTGGAATGAGCAGTATACTTGGGAAGTTTTCGATCCATGCACAGTGGTTACGATCGGTGTTTTTGATAATTGCCATTTGCTTGGGGGAGATAAAGGTGGAGGGACAAAGGATTCACGAATTGGGAAGGTGAGGATACGTCTTTCAACTCTCGAAACAGACCGAGTTTACACACACTCGTATCCTCTTCTGGTTTTGCATCCAAATGGAGTGAAGAAGATGGGTGAGATACACTTGGCTGTGAGGTTCACTTGCTCTTCCTTGCTGAACATGATGCACATGTACACACATCCCCTGCTTCCCAAAATGCATTATATTCATCCTTTGACGGTCAGCCAGCTTGATAGCTTAAGGCATCAGGCGACTCAGATTGTATCAATGAGGCTGACCCGGGCCGAGCCACCACTGAGGAAGGAAGTTGTTGAATATATGCTGGATGTCGGTTCCCACATGTGGAGTATGCGAAGAAGCAAAGCAAATTTCTTCAGGATTATGGGAGTTTTAAGTGGATTAATAGCAGTAGGAAAATGGTTTGATCAAATTTGCAACTGGAAAAACCCCATTACCACTGTACTGATACACATCTTGTTCATAATCCTGGTCATGTACCCAGAGCTAATCCTGCCCACCATTTTCCTCTACCTCTTCTTGATTGGTGTTTGGTATTACCGTTGGAGACCGAGGCATCCGCCACACATGGACACTCGTTTGTCACATGCAGACTCTGCACACCCCGATGAACTTGACGAAGAATTCGACACATTCCCAACATCAAGGGGTGGTGACATAGTGAGGATGAGGTATGACCGACTTAGAAGCATTGCTGGGAGGATTCAGACTGTTGTGGGGGACTTAGCGACTCAAGGAGAGAGGTTGCAGTCTTTGCTTAGCTGGAGAGACCCAAGAGCCACTGCCCTGTTTGTTTTGTTCTGTCTTGTTGCTGCCATTGTCTTATACGTCACACCATTCCAAGTTGTGGCCCTGCTTACTGGATTTTATGTTTTGAGACACCCCAGATTCCGCCATAAGCTTCCTTCTGTGCCACTGAATTTCTTCAGGAGGCTGCCTGCAAGGACTGATTGTATGTTGTGA

>Cusat334820

ATGGCCACTGGCCAACTTAGGAAGCTTATAGTTGAAGTTGTGGATGCTCGTAACCTCTTGCCTAAAGATGGACATGGATCCTCCAGTCCTTACATCGTGGTCGACTACTATGGCCAACGAAAACGGACACGAACCATAGTGCATGACTTGAACCCGACGTGGAACGAGGTTCTCGAGTTCAATGTCGGGCCACCATCGAGTGTGTTCGGAGATGTTTTGGAACTTGATGTGATCCATGATCGAAGCTACGGGCCAACAAGACGAAACAACTTTTTGGGACGGATCAGATTGAGTTCTACTCAATTTGTGAAGAAAGGAGAAGAGGCTTTGATTTATTTTCGTTTGGAGAAGAAGAGTCTCTTTAGTTGGATTCAAGGAGAGATTGGCTTGAAAATTTATTACTCTGATTGCGTTACACCTGCAAGGGTTGAGGAGGGAGATGCAATCAATACAGTTGAGCAACCAACGACTGAAGGCGATGCAATTAATACAGTCGATCAACCAACGACTGAGCCAGAACTAAAGCCCAAAGAGCAAAAACCAGAGCCAGACTCAGAACTAAAGCAATCACCTTTGTTGGAACAACAAGATGTCACCCAACAAACGGACGAACTAGCAAGTATTGAAGGCCAAATCGCTCCAACAACAGAAAATTTGGCAGATAAGGGCAATGCAGCTCCGGAAGTAGAAACTTTGGGAGTCGAGAGCAGTACTAGTCCCACAGAAATTCCAACTCCTGCTGTTGAAACGGTGTCATCAGAAACCCATCCACCGGTGGAAGCGATGGAACAAGGCCGGGAGGCGCCACCAAAAACATCATCGGAAGAGAAGCAACCGACAGCAGAGTCAAAAGAAGAAGCAGAAATCAACTTGACGCCACAACCAATTAAAAGATCGATGCCGATACCAAGCTACACATTGGAGGCAACAGAAAGTCGAACAATGGAACAATCCACATTTGATCTTGTAGAGAAGATGCATTACCTCTTCGTGCGAGTAGTAAAAGCACGCTCACTCGCCACTAATAGCCATCCAATAGTGCAAATCGAAGCATTTGGAAAACGTATCAAATCAAACCCAGCCAGAAAGAGCAACGTGTTTGAGTGGGACCAAACATTTGCATTTAGCCGCGGTGCAGCGGATTCTGCCTCCATGATGGAAATTTCAGTTTGGGATGGCAAAGTAAACGACGCCGTATCACCAACTGACGTGGACGGACGCAATTTCTTGGGTGGCTTGTGTTTGGACGTATCAGATATTCTATTGCGTGACCCACCAGATAGTCCACTGGCCCCACAATGGTACAGATTGGAAAGGGAAAGAAACGACGCCGCTTTTGGTGGGTATTTAATGTTAGCCACGTGGATTGGTACTCAAGCCGACGATGCGTTTCCCAACGCGTGGAAAACAGATGCCGGTGGGAATTTTAACTCTAGAGCAAAAATTTACCAATCGCCAAAAATGTGGTATCTACGCGCCACAGTCATTGAAGCACAAGACGTCGTTCCGATCACCGCTGTGAAAGAAGCTTTGTTTCAAGTCAAAGCTCAACTTGGCTTCCAAGTTTCCGTAACAAAACCCGTCGTGACCCGAAATGGCGCTCCGTCGTGGAATCAGGATTTGTTCTTTGTTGCCGCTGAGCCAATGACCGACCACTTGATCTTCACCGTTGAGAGTCCTCGTAGCTCGAAGTCTCCGACCGTCATCGGAGTTGTAAAAATCCCACTCACTGACATTGAGCGGCGAGTGGATGACCGAAAAGTGACAGCACGGTGGTGCACACTCGCCGGAGTTGTGGATGAAAAGGGATCATCTTACACAGGAAGAATTCAGTTGAGGTTGTGCTTTGATGGAGGGTATCACGTGATGGATGAGGCGGCGCACGTGAGTAGCGATTATCGGCCGACGGCGAGACAGCTGTGGAAGCCGCCGGTAGGTGTGATTGAAATTGGTGTGATCGGATGCAGGGATTTGGTTCCAATGAAGTCCACGGCGACCGGAAAAGGATCCACCGATGCGTATTGTGTTGCAAAATATGGGTCTAAATGGGTCCGAACTAGAACGGTGAGCAACAATTTTGATCCCAAATGGAATGAACAATATACGTGGCAGGTTTACGATCCATGTACGGTTTTGACAATTGGAGTTTTCGATAGTATGGAAGAATCTGAAAATGGTGATCGACCTGATTCACGAATCGGCAAAATACGAATACGAATCTCCACCTTAAAAACTGGTAAGGTATATAGAAATTTTTACCCTCTCCTCCTTTTAACCACTGCTGGTACAAAAAAAATGGGTGAACTTGAAATCGCTGTTCGATTCGTTCGTTCGGCACCGCCGTTGGATTTCTTACACGTGTACTCCCAACCATTGCTGCCGTTGATGCACCACGTGAAGCCTCTCGGAGTTCGGCAACAGGATTTGCTCCGAGGCGCGGCAGTGGAGACGGTGGTTGGTCATTTTTCCAGATCGGAGCCGCCGCTTCGACGGGAGATCGTTGTATTCATGCTGGATGCCGAATCACATAGCTTTAGCATGCGAAAAATTCGTGTGAATTGGTACAGAGTCATCAATGTGGCCTCCACCATCATCGCCGCTGTGAAATGGATCGACGATACTCGATCGTGGCGGAATCCGACGGCCACCATACTAGTCCATGCGTTGCTGGTGATTCTGATTTGGTTCCCTGATTTGATCATTCCGACGATTTCATTTTACGTTTTTGTCACGGGCGCATGGAATTACAAATTGCGATCGTCAGAGCATATTCCGAGTTTCGATTCAAAGCTTTCAATGACGGACATCGTGGAACGAGATGAATTAGATGAAGAGTTCGATGACGTACCGAGCACGAGATCAGCAGAAGTTGTACGGATGAGATACGATAAGTTGAGGGTGATTGGGACACGTGTGCAAAGTTTATTGGGGGATTTAGCAACTCAAGGGGAGCGTGTACAGGCGTTGGTGACATGGCGTGACCCACGTGCCACTGGTATTTTTACTGGGATATGCTTTGCGGTGGCAGTGGTGCTCTACGTTGTGTCGTTGAGGATGGTGGCAGTGGCGTTCGGGTTTTATTACCTCCGCCACCCAGTTTTTCGAGATCGATTGCCGTCACCGGCTCTTAACTTCTTAAGAAGACTTCCGTCTTTGTCAGATCGATTAATGTAG

>Cusat357330

ATGATGCAAAAGCCTCCACCAGAGGATTTCTTACTGAAAGAGACCAATCCCCACCTTGGTGGGGGGAAGGTCGCTGGTGACAAACTAGCAAGCACCTATGACCTTGTGGAGCAAATGTATTACCTCTACGTTCGGGTCGTCAAAGCGAAGGATTTGCCAGGGAAAGATGTCACTGGTAGCTGCGATCCGTATGTGGAGGTCAAGCTTGGAAACTACAAAGGTACGACTCGGCATTTTGAAAAGAAGTCGAATCCTGAATGGAATCAGGTGTTTGCCTTTTCGAAGGATCGGATTCAGGCTTCGGTGCTTGAGGTGAGTGTGAAGGATAAGGATTTTGTGAAGGATGATTTCATGGGGAGAGTTCTTTTTGACTTGAATGAGGTTCCAAGAAGGGTACCACCGGACAGTCCTTTGGCCCCACAATGGTATCGGTTGGATGATCGGAAAGGGGACAAGGTGAAAGGAGAGCTGATGTTGGCTGTTTGGATGGGAACACAAGCAGATGAAGCATTTCCTGAGGCTTGGAATTCGGATGCTGCAACTGTAAGTGGAGCCGATGGGCTTGCGAATATTCGGTCTAAAGTGTATCTTTCTCCCAAGCTTTGGTATTTGAGGGTGAATATCATTGAAGCTCAAGATCTGCAGCCAACCGATAAAGGTCGGTATCCAGAGGTTTTTGTCAAGGCTATACTTGGAAATCAGGCGTTGAGGACTAGAATATCTCAGTCTAGAACTATCAACCCAATGTGGAATGAGGATTTGATGTTTGTAGCAGCTGAACCATTTGAAGAGCCTTTGATTTTAAGTGTGGAAGACCGAGTTGCGCCGAACAAGGATGAGGTCTTGGGACGGTGTGCAATTCAGCTGCAATATATAGATAGGAGATTGGACCATAGAGCTGTGAACACAAGATGGTTCAATCTTGAGAAACATGTTGTTGTTGTTGAAGGGGAGAAGAAGAAGGAGATCAAGTTTTCTAGTAGGATTCATATGAGGATATGTTTGGAAGGTGGCTACCATGTCCTGGATGAATCAACACATTATAGTAGTGATCTTCGACCGACTGCGAAACAACTCTGGAAGAACAGCATTGGAGTTTTAGAATTGGGGATTCTGAATGCTCAGGGGTTAATGCCAATGAAGACAAAAGACGGTCGTGGAACGACAGATGCATACTGTGTGGCTAAGTATGGGCAGAAGTGGATTAGAACGAGGACAATCATCGACAGCTTCATACCCAAGTGGAACGAGCAGTACACTTGGGAGGTTTTTGATCCCTGTACTGTCATTACAATTGGAGTTTTCGACAACTGCCATTTGCATGGTGGAGAGAAGGCAGGAGTATCAAAAGATGCTCGGATAGGGAAGGTAAGAATTCGTCTTTCCACGCTCGAGACAGATCGAGTGTATACTCATTCATATCCTCTGCTGGTTCTTCACCCAAATGGGGTGAAGAAAATGGGTGAAATTCATTTGGCTGTGAGGTTTACATGCTCTTCTTTGTTAAACATGCTGCATATGTACTCACATCCACTCCTTCCCAAAATGCACTATATTCATCCGTTAACGGTGAGCCAGCTCGATAGCCTTCGCCATCAGGCAACACAAATTGTCTCAATGAGGCTGAGCCGTGCTGAGCCACCACTGCGAAAAGAGGTTGTCGAGTATATGCTTGATGTAGGCTCCCACATGTGGAGCATGAGAAGAAGCAAGGCTAATTTCTTCAGAATCATGGGAGTTTTTAGTGGCCTGATTGCAGTTGGAAAATGGTTTGATCAGATTTGCAATTGGAGAAACCCCATTACAACTGTTCTTATACATATCTTGTTCATAATCCTTGTCATGTACCCTGAGCTCATTCTTCCCACAATTTTCCTCTATCTCTTCCTAATTGGAGTTTGGCATTATAGATGGAGGCCAAGGCACCCTCCTCACATGGACACCCGTCTCTCTCATGCCGATTCCTCTCACCCCGATGAACTCGATGAGGAGTTTGACACTTTTCCAACTTCTCGCCCTGCTGACATTGTGAGGATGAGGTACGATCGGTTGAGGAGCATTGCGGGGAGAATCCAAACTGTTGTCGGGGACCTAGCAACACAAGGAGAAAGGCTGCAATCTCTACTCAGTTGGCGCGACCCGAGAGCGTCGGCATTGTTTGTGATCTTCTGCTTGGTCTCTGCCATAGTTCTGTACGTTACACCATTCCAAGTTGTGGCACTTCTTTCAGGGATTTATGTGCTTAGACATCCGAGGTTTCGGTACAAGCTCCCGTCTGTGCCGCTCAACTTCTTCCGAAGGCTGCCGGCCAGAACAGACTGTATGCTATGA

>Dacar000606

ATGAACAACCTTAAGCTTGGAGTTGATGTAGCTAGTGCCCACAACCTTCTGCCAAAAGATGGGCAAGGCTCATCCAATGCCTATGTGGAACTCTACTTTGATGGCCAAAGGCACCGCACAACCATCAAAGAGAAAGACCTGAGCCCAGTCTGGGATGAAAGCTTCTACTTTAACATCTCCGATCCATCCAACCTTCACAATCTAACTCTTGAAGCATACATTTACAATAATATCAAAATCGCCCAATCAAATTCTTTTCTTGGAAAGGTTTCCATCAACGGGACATCCTTTGTTCCCCACTCTGATGCTGTAGTCCTGCACTATCCTTTGGAAAAGCGTGGAATTTTCTCACGTGTGAGAGGAGAACTCGGTCTGAGGGTTTATATTACTGATGATGCATCCTTGAAGGCCTCTAAATCACAACATGCAGCTGAAGAGACTGAAATGCATTCCCACGCAGCTGAAGCTCAAGTATCTAGGGCATTTTCTAATATCAAATCTGAATCGAGGCACAGTTTCCATCATCTTCCAAATCCTAGTCATCACATGCAGCAGCAGCATTCCCCTTCTATGTCAGTTTATGAATCAACAAAATATGGGGTTGATAATATGAAAGCTGAACCACAACCCCCAAAACTTGTCCGCATGTATTCTGCTGCATCAGTGCAACCAGTTGACTATGCACTTAAAGAGACGAGCCCATTTCTTGGAGGAGGACGAGTTGTAGGGGGGCGAATACTTCATACAGACAAGGCTGCTTGTACTTATGATCTTGTAGAAAAAATGCACTTTCTTTTTGTACGTGTTGTTAAGGCTCGTGAACTTCCTGCCATGGATATTACTGGAAGTCTTGATCCTTTTGTTGAGGTGAGAATAGGTAATTACAGAGGAGTCACAAAGCATATGGAGAAACAGCAGAACCCAATGTGGAATGTTGTTTTTGCATTCTCAAGGGAGCGAATGCAGGCATCGGTCCTGGAAGTTGTAGTTAAAGACAAAGATCTGCTGAAAGATGACTTTGTTGGCCTTGTAAGGTTTGACCTGAATGAAGTTCCTATGCGTGTTCCACCTGATAGTCCACTGGCTCCTGAATGGTACCGACTCCAGGATAAGAAAGGAGAGAAAATTAAGAGTGAACTGATGCTTGCAGTGTGGATTGGTACCCAAGCAGATGAAGCTTTTCCTGATGCTTGGCACTCTGATGCAGCAACGCCTATTGACAGCTCCGGAGCTGCCTCAACACTAATTCGGTCAAAGGTCTATCATGCACCGCGATTATGGTATGTTCGTGTGAATGTTGTTGAGGCCCAAGACTTGGTTCCAACAGAGAGAACTCGCTTCCCTGACGTATATGTAAAGGCACATATAGGGAGCCAGGTTTTCAAAACAAAGACAGTTCAAGCAAGGTCTCTGAATCCTCTTTGGAATGAGGATCTCATATTTGTTGCCGCAGAACCATTTGAAGATCATTTGGTCCTTACTGTTGAGGATCGTGTGGGTCCTGGAAAAGATGAGATTCTGGGGAGGGTAATCATCCCCTTAAGCATGGTGGAGAAGCGTGCTGATGATCGTCTCATCCATTCTCGTTGGTTTAATTTGGAGAAACCTGTCGCTATAGACGTGGATCAGCTAAAAAGAGAGAAGTTCTCTAGCAGGCTCCATCTTCGAGTCTGTCTAGATGGTGGTTACCATGTTCTTGATGAATCTACACATTACAGCAGTGATCTTCGTCCGACAGCAAAACAACTTTGGAAGCCATCTATTGGGGTTCTGGAACTTGGTATTCTCAATGCAGTTGGACTCCACCCTATGAAAACAAGAGATGGCAGGGGCACATCAGACACATATTGTGTAGCCAAGTATGGTCATAAATGGGTTAGGACGCGCACAATTGTTGATAATTTGTGTCCAAAATACAATGAGCAGTACACTTGGGAGGTGTTTGATACTGCTACAGTTCTAACTATAGGTGTTTTTGACAATAGCCAGCTAGGGGAAAAAGGGGGCAAGGACCTGCAAATTGGAAAGGTACGAATTCGCCTTTCTACACTTGAAGCTGGCCGCGTTTACACACACTCTTATCCTCTACTTGTTCTTCACCCTACTGGTGTCAAGAAGATGGGGGAGGTGCATTTGGCAATAAGGTTTTCATGCACTTCTTTTGTAAACATGATGTACATATACTCTAAACCTCTGTTGCCGAAAATGCACTATGCTAGGCCATTTAGCGTAATGCAGCTTGACATGCTGCGTCACCAGGCTGTGAACATAGTTGCAGCGCGCCTGGGACGTGCAGAGCCCCCTCTTAGGAAGGAGGTCGTGGAGTATATGTCTGATGTGGACTCACATTTGTGGAGCATGCGCCGCAGCAAGGCCAATTTCTTCCGTCTCATGTCAATTTTTTCGGGATTATTTGCTGTTGGAAAATGGTTTGGGGATATATGCATGTGGAAAAACCCCATCACAACAGTGCTTGTCCATGTCCTATATGTTATGCTTGTTTGCTTCCCAGAACTAATTTTGCCTACTTGTTTCCTTTACATGTTTCTGATTGGAATCTGGAACTTTCGATACCGGGCAAGATACCCTCCTCACATGAACACAAAGATCTCACAGGCAGAGGCAGTGCACCCTGATGAACTTGATGAAGAATTCGACACATTCCCCACAAGTCGTAACCCTGAGATTGTCAGAATGAGGTATGATCGCCTGAGGAGTGTAGCTGGTAGAATTCAAACAGTGGTAGGTGATATTGCTACACAGGGTGAGCGTGTTCAATCATTGTTAAGCTGGAGGGACCCTCGCGCTACCTCCATCTATGTCACATTCTGCGTAGTAGCTGCTATAGTGCTGTATGTGACACCATTCCAGGTGATTGCTGCATTGGCAGGGGTATACATGATGAGGCATCCCAGATTCCGCTATCGTCTGCCTTCTGTGCCTGTCAATTTCTTCCGTCGACTTCCTGCAAGGACTGATAGTATGCTCTAA

>Dacar003526

ATGGGGAGGCTTGTAGTTGAAGTCCTTGAGGCCAATGACCTGATGCCGAAAGATGGACAAGGCTCGGCAAGTCCCTTTGTTGAGGTAGATATAGATGATCAGCATCAAAGGACACAGACAAGGATTAAGGATCTTAACCCCTCGTGGCACGAAAAATTTGTCTTTAAATTCGATGACTTGCAGGATCTTTCTAGGAAAACTATTGAAGTTGTCATCTACAATGAAAATACTAGTCATGGCAACCACAATTTTCTTGGTCGAGTTCGGTTATCTGGTCTGTCAGTTCCTAAGTCCGAGTCTGAAGCAACGGTGCAACGATATCCTCTTGAGAAACGAGGTATATTTTCTCATATTCGTGGTGATATCGCGCTCAGGCTGTATCTTGCTGATGGCAAAGATGAGGATGGCGCTTCATCAGATCAAGCTGAATTACCAAAAGAAAAGACGAAGAAAGGCATGGAAAGTAGAGAAACTCCGGTGCAAGAAAATGTTAAAAGGTATGATAAGAAGGCTCAGGATGATGAGTTTAATGATTTTAGCAAACAGAAGATAAAAAGGAAGAAGGAGAAAGAGATTAGGACTTTCTACTCTGTTGGAACTGGTGGCCCAACTGGTCCTATGCCTCCAATGCAGAAGCCAGTAGTTCTTGAAACCAGGCCTCAGTTTATCCCACCAAGGCCACCTATGGTTATGCAAAATCAAATTCCCATGCAGAAGCCTGAATTCGGGTTGGTTGAGACGCGGCCACCAGTGGCAGCAAGGATGGGGTATTTGGGAGGTGACAAGATGGCGAGTACTTATGATTTGGTGGAGAAAGTGCATTACTTGTACATCAATGTGGTGAAAGCTAGAGATCTTCCTGCTTTGGATGTGAATGGAAGTCTTGACCCTTATGTTGAAGTAAAGGTGGGAAACTACAGGGGCTTGACAAAACACTTGGAGCATAACCAGGATCCGATGTGGAACAGTGTGTTTGCGTTCTCTAAAGAAAGGCTGCAGTCAAATGTGATTGAGGTGATAGTGAGGGCTAAAGATTTGGGGAAGGATGATTATGTTGGGAAGGTGGTTTTCGATGTAGTGGAAGTGCCTCTTCGGGTGCCACCTGATAGTCCTCTGGCTCCCCAGTGGTATAGATTGGCTGGTCAGGGAGAGATTATGCTTGCAGTTTGGCTTGGAACCCAGGCTGATGAGGCTTTTCCTGAGGCATGGCATAGTGATGCTCATGATATTAATCATCATAACCTAGCGAGTACACGCTCAAAGGTTTATTTCTCGCCTAAGTTGTACTATCTTCGCGTCCATGTCATTGAAGCCCAAGATCTTGTGCCTTCTGATAGAACAAGACTACCACAGCCATATGTGAGAATACAGCTAGGACATCAGATCAGGGTCACCCGGCCTTCCCAAACGAGGAACATAAATGCTGCTTGGAATGAAGAGCTTATGTTTGTAGCTTCTGAGCCTTTTGAAGACGTCCTTATTGTGAGTGTTGAAGACCGTGGTGAAGGGGGAAGGGAGGATAATATGGGAAGAGCAATGGTACCGGTCAGAGAGATTCCACCAAGGGTGGATTCAAGTAAGCTCCCGGATCCTCGCTGGTTAAATCTTCAGAGACCTTCTCACTCTGTGCATGAAGATGGCGAGAAAGAAGTCAAGTTTTCTAGCAAAATCCGCCTCTGCCTCTGCTTAGACGTCGGGTACCATGTCTTAGACGAAACGACTCATTTCAGTAGTGATCTTCAGCCATCATCCAAGTACTTGAGGAAACAAAGAATAGGAATTCTTGAAGTAGGGATATTGAGTGCTCAAAATCTGCTACCAATGAAAATCCGAGAAGGTGGAAGTACAGATGCATATTGTGTAGCCAAGTACGGAAATAAATGGGTACGTACTAGAACTCTTCTTAACACACTGTCTCCCAGGTGGAACGAGCAGTATACGTGGGAAGTTTATGATCCCTGCACTGTAATTACTATTGGTGTATTCGACAATCATCATGTTAATGGAAACAAAGAGGATGCAAGAGATCAGAGGATCGGGAAAGTAAGGATTCGTCTATCAACTCTTGAAACTGATAGAATTTATACACATAACTATCCCTTGCTGGTTCTGCAACCCTCTGGTTTGAAGAAACACGGAGAGCTTCATTTAGCCATACGCTTCACTTGTGTAGCTTGGGTTAATATGTTGACCCAATATAGCAAGCCATTACTCCCCAAAATGCACTATGTCCAGCCTATTTCTGTCCGACACATTGACTGGCTACGACACCAAGCAATGCAGATAGTTGCAGCACGGTTAAGCAGGGCAGAACCACCTCTAAGACGCGAAACAGTTGAGTACATGCTTGATGTGGACTATCACATGTGGAGCCTCAGGAGAAGCAAAGCAAATTTTTACCGAATAATTTCACTCCTCTCAGGATTTTCAGCTATCTGGAAATGGTTGGATGGAATTTGCAACTGGAGAAACCCTCTGACAACATGCCTTGTGCACGTACTTTTCTTGATCCTGGTTTGCTACCCTGAACTAATCCTCCCTACAATATTTCTCTACTTGTGTGTGATTGGTTTATGGAACTACCAGTTCAGACCCCGAAAGCCTCCACATATGGATGCACGAATCTCACAGGCTGAGAACGTGCATCCAGATGAGCTAGACGAGGAATTTGACACATTTCCATCTTCGCGACCTACAGACCTAATTAGGATGAGGTATGATCGGATGCGTAGTGTGGCAGGAAGAGTGCAAACAGTCCTCGGAGATTTAGCTACACAAGCGGAGAGAGCTTTGGCAATACTTAGCTGGCGAGATTCAAGAGCAACCGCAATCTTCATTATATTTGCTCTCATGTGGGCAACTTTGTTTTACCTCACTCCCTTTCAGGTCATTGCAGTGCTTCACGGGCTGTACTGGATGCGCCATCCGAAGTTCAGAAACAAGATGCCTTCTGTTCCTGTCAATTTCTTCAAAAGACTCCCATCAAAGTCTGATATGCTGTTATCCTAG

>Dacar006920

ATGCGCAATCTCAAACTAGGAGTAGAGGTTGTTAGTGCCCACAACCTTGCACCAAAAGATGGCCAAGGCTCATCTAATGCCTTTGTGGAGCTTCACTTTGATCATCAGAGATTTCGCACCACTGTCAAGGAGAAGGATCTTGACCCTGTTTGGAATGAGTCTTTCTATTTTAACATCTCCAATCCAGAAAACCTATCCAATCTCACCCTTGAGGCCCATGTCTACAGCAACGGTAAAGCCAACAACTCTAAATCTTCACTTGGCAAAGTTTGCATAACAGGGACATCGTTCGTGGCTTACTCAGATGCTGTTGTTTTGCATTACCCTCTTGAGAAACGAGGCATTTTCTCCCGTGTTAAAGGTGAGCTCGGGCTTAAGGTATTCCTCACTGATGATCCATCCATTAAGTCCTTTAATCCAGTTCCTACAATGGAGCCATTCTCGCATACAAATTCATTATCAGCTCAAGCTCAAGCAACTGTCCAACAAGAACAGAATTTGGATCAGGATTGGTCGTTGAAGGAGAAAGACGAGTCAATACGCACATTCCACCATCCGGAAAATCCAAAGCAGCAGCAGCAACAGTACCAACAACAACAACAGTATATCCCTACTGCTACCGTGCAACAACCAATGAGATATGGTGTTGATGAAATGAGATCTGCACCTCCCGCTTCCAATATTGGTTTCATGTCCTCGGGTTCTTCATCACAACCAATGGAATATGCACTGAAAGAGACTAGCCCTTTTCTTGGAGGGGGTAGAGTTATTGGGGGTCGAGTGATACGTGCAAACAAGCCAGCTAGTACTTATGATCTTGTTGAACCAATGCAGTTCCTTTTTGTACGAGTAGTAAAAGCACGCGAGCTTCCTAACATGGATGTTACAGGTAGCCTTGATCCTTATGTGGAAGTACGAGTAGGAAACTATAAAGGAGTAACATCGTACTTCTCCAAAACACATAAACCGGAATGGAATACTGTGTTTTCGTTCTCACGGGAGAGAGTACAAACATCTACCCTGGAAGTCGTAGTTAAGGATAAAGATTTGTTAAAAGACGATTTTGTAGGGATTGTGCTAATTGATGTTAATGATGTTCCTACCCGAGTTCCCCCAGACAGTCCACTGGCACCAGAATGGTATCGCCTAGAAGATAAGAAGGGACGGAAAATCAATGGAGAACTGATGCTTGCTGTCTGGATCGGAACACAAGCTGATGAGGCTTTTCCTGATGCTTGGCATTCAGATGCTGCTATACCTGTTGATAGTTCCATGCCATCTACCCAAATCCGCTCTAAAGTGTACCACTCACCAAGACTATGGTATGTTCGTGTGAACGTAATTGAAGTTCAGGACCTGGTTTTGTCCAATAAGACTCGGTTCCCGGATGTATATGTCAAAGTACAGATAGGTAGCCAGGTTCTGAAAACAAAGCCAATTCAAGCACGTTCTGTTCATGCAGTGTGGAATGAGGACATGATGTTTGTTGCTGCTGAACCCTTTGAAGATCATGTTGTGCTTTCAGTTGAAGAACGTGTTGGTCCCAACAAAGATGAAATTTTTGGAAGGGTCATTATACCATTGAACTCAGTTGAAAAGCGGGCTGATGATAGAATGATTCATGCTCGATGGTTCAGTCTCCAAAAACCGAGTATAACTGATGTTGAAGAGATAAAGAAGGAAAAATTTGCTACAAGGCTCAACCTTAGAGTTTGCCTTGACGGAGGGTATCATGTACTTGACGAATCCACTCACCACAGCAGTGATCTCCGACCAACAGCTAAACAGCTGTGGAAGCCTCCAATAGGTCTTCTAGAACTCGGCATTTTAAATGCTGATGGTCTCCACCCTATGAAAACCAGAGACAAAAGGGGCACATCAGATACATATTGTGTAGCCAAGTATGGCCACAAATGGATTCGGACCCGAACAATCAATGACAGCCTAAATCCAAAATATAATGAGCAATATTCTTGGGATGTTTATGATCTGGCAACTGTTCTCACTGTAGGTGTTTTTGATAACAGTCAGCTTACTGAGAATGGTTCAAGTGGAACCAGAGATATGAAAATAGGTAAGGTTCGCATTAGGCTCTCTACCCTTGAAACTAGCCGTGTATATACACACTCGTATCCTTTGTTAGTCCTTCATCCCTCTGGTGTGAAGAAGATGGGGGAATTACATCTAGCTATCCGGTTTTCAAGCACATCTATGATCAACATGATGTGTATGTACTCGCGTCCCCTTTTACCAAAAATGCATTATGTGAGGCCCTTGACAGTGATGCAGCAAGACATGCTTCGACACCATGCTGTTAACATAGTGGCAGCTAGGCTCAGGCGTGCTGAACCACCACTTCGGCAAGAAATAGTTGAATACATGACTGATGCTGATTCACATCTCTGGAGCATGAGGCGAAGCAAGGCAAACTTTTTCCGCTTGATCTCAGTTTTTAATGGATTGTTTGCTGTTAGTAAATGGTTTGGAGAGGTTTCTTTGTGGAGGAATCCTGTCACGACGATACTAGTACATGCTCTCTTTCTAATGCTCGTGTATTATCCGGAAATGATTCTTCCAACAGTGTTTCTATACATGTTTATTATAGGACTGTGGAACTACCGGTTTCGGGAAAGATACCCGCCTCACATGAACACAAGAATCTCTTATGCCGATTCAGTACACCAGGATGAACTTGATGAGGAATTCGACACATTTCCTACATCTCGAAGCTCAGACCTTGTCCGAATGAGGTATGATCGCCTAAGGAGTGTGGCTGGACGGATTCAGAGTGTGGTTGGTGACATAGCAAGCCAAGGGGAACGTGTGCAAGCACTTCTCAGTTGGCGGGATCCACGAGCCACCATCATATTTCTAACATTTTGTTTAGTAGCTGCCATGGTGTTGTATGTAACTCCTTTCCAAGTACTAGCTGCGTTTGCTGGATTATATGTCATGAGGCATCCTTGGTTTCGTCATAAGTTGCCATCCGCGCCACTCAATTTTTTTCGTCGGTTGCCTGCCAGGACTGACAGCATGTTATAA

>Dacar008603

ATGAGCAGTCTCAAACTAGGAGTGGAGGTTGTCAGCGCCCACAACCTTATGCCCAAAGATGGCCAAGGCTCATCCAGTGCCTTTGTGGAGCTCAACTTTGATCATCAAAGATTTCGGACCACTATCAAAGAGAAGGATCTTGACCCTGTTTGGAATGAGTCTTTCTATTTCAACATCTCTAATCCAGAAAATCTACCCAATCTCACTCTCGAGGCCCATGTCTACAACAATAATAAAGCTAACAATTCCAAATCTTCCCTAGGCAAGGTTTGCATAACAGGGACATCATTTGTCCCCTACTCGGATGCTGTTGTTTTGCATTATCCTCTTGAAAAACGAAGCATTTTCTCCCGTGTTAAAGGTGAGCTTGGCTTGAAGTTATTTCTCACCGATGATCCCAACATAAAGTCCTCCAATCCCCTTCCAATGGAGAACTCTTCGCAGAAAAATTCGTTATCTGCCCAAGCTCAAGCAACTGTGCAGCAGACTCAGAATCGGTCATCAAAGGTCAAAGATGAGTCAGTTCGTACATTCCACAATCTAGCGACTCCAAAGCAGCAGCAGCAGCAGCAGCAACAGCCATATATTCCTAGTACTGCTATGCAACAACCAGTAAGATATGGTGTTGATGACATGAGACATGAATCTCGAGCTTCCAACCTTGCCCGCATGTACTCAGGTTCTTCATCGCAACCAATGGAATATGCACTGAAAGAGACTAGTCCTTTTCTTGGAGGGGGCAGAATTGTTGGAGGTCGTGTGATACGTGCAGACAAACCAGCTAGTACTTATGATCTTGTTGAACCAATGCAGTTCCTTTTTGTACGAGTTGTCAAAGCCCGGGACCTCCCGACTATGGATGTTACGGGTAGCCTTGATCCTTATGTTGAAGTACGAGTTGGAAACTATAAAGGAGTCACACAACACTTCTCGAAAACACATAACCCAGAATGGAATACAGTTTTCTCTTTCTCACGGGAGAGAGTGCAAACATCTGTATTGGAAGTTGTTGTTAAGGATAAGGATCTGTTGAAAGATGAATTTGTTGGCATTGTTCGCTATGACATAACCGATGTTCCTACACGAGTTCCTCCAGACAGTCCATTGGCACCAGAATGGTATCGCCTGGAAGACAAGAAAGGACAGAAAACCAAAGGAGAACTGATGCTTGCAGTCTGGATTGGAACACAGGCTGATGAGGCTTTTCCTGATGCTTGGCATTCCGATGCAGCTATTCCTGCTGATAGCTCAATGCCGTCCACCCACATCCGCTCTAAAGTGTACCATTCCCCAAGACTATGGTATGTTCGTGTTAATGTGATTGAGGTTCAAGACCTAGTTTTGTCTGACAAGACTCGTTTTCCGGATGTATATGTTAAAGTACAGATAGGTAGCCAGATTCTTAAAACAAAGCCAATTCAGGTACGATCTGTGAATGCAATGTGGAACGAGGATATGATGTTTGTTGCTGCTGAACCCTTTGAAGATCATCTTTTTCTTTCTGTCGAAGAACGTGGTGGCCAGAACAAAGATGAAATATTTGGAAGGGTCCTTATACCATTGAACTCTGTTGAAAAGCGTGCTGATGATAGATTGATACACTCTCGATGGTTTAATCTCCAAAAGCCAAGCATCACTGATGTTGAAGAGATAAAGAAAGAAAAATTTGCTACAAGACTCAATGTACGAGTTTGCCTTGATGGAGGGTATCATGTACTTGACGAGTCCACTCACTACAGCAGTGATCTCCGGCCAACAGCAAAACAGCTATGGAAGCCATCCATAGGTATCCTGGAACTTGGCATTTTAAATGCTGATGGTCTCCATCCAATGAAGACAAGAGATGGAAGGGGCACATCAGATACGTATTGTGTAGCCAAGTATGGCCACAAATGGATTCGAACCCGAACAATCAATGATAGTTTAAGTCCCAAGTACAATGAGCAATACACATGGGAGGTTTTTGATCCGGCAACTGTTCTCACTGTAGGTGTTTTTGATAACAGTCAGCTTCTGGAGAATGGTTCAAGTGGAAACAGAGATATGAAAATTGGTAAGGTACGTATTAGGCTTTCTACTCTTGAAACCGGTCGTGTTTATACTCACTCATACCCGTTGCTAGTACTTCATCCCTCTGGTGTAAAGAAGATGGGGGAACTGCATCTAGCAATCCGGTTTTCAAGCACGTCTATGACCAACATGATGTACATGTATTCACGACCCCTGTTGCCAAAAATGCATTATGTGAGGCCATTGACTGTGATGCAGCAAGACATGCTGCGACACCAAGCTGTCAACATAGTGGCAGCTAGACTCAGCCGTGCTGAACCACCACTTCGGAAGGAAATAGTCGAGTACATGACTGATGCGGATTCACATCTCTGGAGCATGAGGCGAAGCAAGGCAAACTTTTTCCGTTTGATGTCAGTTTTTAATGGATTATTTGCTGTTGGAAAATGGTTTGGGGAGGTTTCTATGTGGAGGAATCCTGTCACTACAGTGCTAGTGCATGCTCTATTTCTGATGCTCGTGTGTTTTCCAGAACTGATTCTTCCAACTGTGTTTCTATACATGTTTCTTATAGGACTGTGGAACTACCGATTTCGAGCAAGATATCCCCCTCACATGAACACAAGAATCTCTAATGCTGATTCAGTAAGTCGGGACGAACTTGATGAGGAATTTGACACATTTCCTACATCTCGAAGCTCAGAGGTAGTTCGGATGAGGTATGATCGGTTAAGGAGTGTGGCTGGCCGGATTCAGAGTGTGGTACTAGCTGTGTTGACTGGATTTTATGTCATGAGGCATCCTAGGTTCCGTCATAAGTTGCCATCTGCACCGCTCAATTTCTTCCGTAGGTTGCCTGCGAGGACTGATAGCATGCTGTGA

>Dacar009086

ATGGCGGAGAGTTGCAGCAGAAAGCTGTATGTAGAGATCTGCAACGCCAAGAACCTGATGCCTAAAGACGGCCAAGGCACTGCTAGTGCTTATGTTGTCGTAGATTTTGATGGCCAGAGACGTCGAACACAGACGAAATCCAGGGACTTGAATCCGCAGTGGGACGAGAAACTCGAGTTTTTGGTTCATGATGTAGCGGCAATGCCTTCTGAAGTGCTCCAGATCATTGTTTATAATGACAAGATGTCGGGGAAGAGGAGCACGTTTCTCGGGAAAGTGAAGATCTCCGGGAGTACTTTTATGAAAGTTGGTTCGGAGAGTCTTGTTTATTATCCGCTGGAGAAGAGGAGTGTTTTTTCGCAGATTAAAGGCGAGATCGGATTGAAGGTGTGGTATGTTGATGAGGAGCCGGCGGCGCCGCCGGCTCCGGCGGAAGGCGAGAAGAAAGCGGAAGCTGTTGCGGAGGAGAAGAAACCGGAGGAGGTGAAAAGTGGTGAAGAAGAGAAGAAAATTGAGGAAGTGAAGAAGGAGGATACGGCTCCGGATGCGAAGGAGGGTGAAAAGACGGGAGACATGAAAGTCGAGGAGGCGGCTCAATCGGCCACAGCTGCGGCAGCTGTGGCTGTGGTGGAGAATCCTCCCGTAGCTGAGAAGCCGAAGGCTGAGGAGAAGGCGGTGGAGAAACGAGTGAATTTGAATGTGACTGATCTGGAGCTTCGGAGGCTTGGAAGTGATCGAGGCCGGACTGCTTATGATCTGGTTGATCCAATGCCGTTTCTTTTTGTTCGTGTTTTGAAGGCGAAGCGAGGGGATAAGGACCAGGCTGATAGCTCTGTTTATGCAAAGCTTGTGATCGGTACTCATAGTATTAATACCAGGAAACAAACGGATAATAAGGATTGGGATCAGGTTTTCGCGTTTGATAAGGAAGGATTGAATTCGACTTCTCTAGAAGTTTCCGTTTGTGTTGAGAAGAAAGGGGCGGAGGATAGTGTGATTGAGAGTAGTTTGGGGACGGTGTCGTTTGATTTGTTGGAGGTGCCTAAGAGAGTTCCACCGGATAGTCCATTGGCACCGCAGTGGTATACATTGGATGGAGGTTCGTCTGAGGGGACTACGGATGTTATGCTTGCTGTTTGGATCGGGACTCAGGTGGATGAGGCTTTTCAGGAGGCTTGGCAATCGGATTCAGGTGGATTAGTACCGGAGACACGAGCCAAGGTTTACTTGTCTCCGAAGCTGTGGTATTTGAGGCTAACGGTCATCCAAGCCCAGGACTTACAGCTGGGATCAGGCTCGGAGATTAAAGTTAAGAATCCGGATTTATACGTTAAGGCTCAATTGGGGCCGCAGCTATTCAAAACGAGTCGCACAACGGTTAGTTTGTCCAGCTCATCGTCTAATCCAACGTGGAATGAAGATCTTGTGTTTGTGGCAGCTGAGCCATTTGAGCCCTTTTTGCTGATCAATGTGGAGGATGTTTCTAACGGTCATACGGTGGGGCAGGCTAAGGTACAAATGTCAAGTATTGATAGGCGGAATGATGATAAGTCGGAGGCAAGATCAAGGTGGTTCAATCTGGTAGGCGATGAGACGAGGCCTTATACAGGAAGAGTTCATGTTCGGGTGTGTCTTGAAGGCGGATATCATGTGCTTGATGAGGCTGCTCATGTGACCAGCGATGTCCGGCCTACTGCAAAGCAACTGTCTAAACCACCTCTTGGTATACTAGAAGTTGGTATTCGCGGAGCTACAAATTTGCTTCCTGTGAAAACAGCTGATGGGACAAGAGGCACAACGGATGCATACGTTGTGGCAAAGTACGGGCCTAAGTGGATTCGTACTCGTACAATTCTTGATAGGTTTAATCCGCAATGGAATGAGCAATACACATGGGATGTCTATGATCCGTGCACTGTGCTTACAATTGGCGTGTTTGATAATGGAAGGTATAAGCGTAGTGGGGAAGAAGGTAAACCGGGAAAGGATGTGCGCCTAGGAAAGCTGCGTGTAAGGCTGTCCACGCTGGATGCTAACCGTGTTTACACAGGATCGTATTATCTCACTGTGTTGCTTCCTGGTGGTGCCAAGAAAATGGGGGAGATTGAGATTGCTGTTAAATTTTCGTGTTCCTCCTGGCTATGTTTACTTCAAGCATATGCTAGTCCAATGCTGCCGAGGATGCATTATGTTCGGCCATTGGGGCCGTCTCAGCAAGACATTTTGAGGCACACGGCTATGAGAATTGTGACGGCTAGACTATCTCGGTCTGAACCGGCTTTAGGTCAGGAAGTGGTTCAGTTTATGTTGGATTCCGACACACATATGTGGAGTATGAGGCGTAGCAAGGCTAATTGGTTTCGAGTGGCTGGGTGTTTGTCAAGAGCTGCCACATTAGCTCGGTGGCTGGATGGAATCCGCACCTGGGCGCACCCGCCAACTACAATATTAGTCCATGTCTTGCTAGTGGCCATTGTGCTGTGTCCTCACCTAGTCCTGCCCACAATCTTTATGTATGCATTCCTGGTCATCACATTGCGCTTCCGCTATCGCCAGAGGGTCCCGATCACAATGGACCCTCGGCTTTCTCACGTGGAAGCAGTAGGACCGGATGAACTTGATGAGGAGTTTGATGGATTTCCGACCACAAGGTCACCTGATCATGTTCGCATGAGATATGATCGTTTACGCGCCCTGGCTGGGAGGGCGCAGACACTATTAGGCGATGTGGCAGCGCAGGGTGAGCGTTTAGAGGCGTTGTTCAACTGGAGGGATCCGAGGGCTACTGGCATATTTGTTGTGGTCTGCTTATTAGCTTCACTGGTGTTTTATGTTGTCCCATTCAAGGCATTTGTTCTGGTCTCAGGATTTTACTACCTGCGTCACCCCAGGTTCCGCTATGACATGCCATCGGTTCCTGTCAACTTTTTCCGGCGACTTCCCCCCCTGTCGGATCAGATTCTGTAG

>Dacar013475

ATGGCAGTTGGTGTTCGGAAGTTAATAGTAGAGGTGGTGGACGCTAGAAATTTGTCACCCAAAGACGGGCACGGAACATCAAGTCCATACGTGATACTTGATTTCTACGGGCAAAGAAAAAAGACAAGAAGTGTAATTTGTGATCTCAATCCTGTATGGAACGATATTGTATCTTTCAATGTTGGAAAGCCATCTAGTGTTTTTGGTGACATGCTGGAGGTAGACGTTTATCACGACAAAAATATTGGCCCTACTACCAGAAACAATTTCCTAGGCCGAGTGCGATTGGATTCTCGACAGTTTGTTAAAAAAGGGGAAGAGGCTTTGATTTACTATCCATTGGAGAAGAAAAACCTCTTCAGTTTTATCCAAGGTGAGATTGGCTTGAAGATTTATTTCAGTGACGAGGTCGTTCCACCGCCAGAGCCACCAGCACCATCACCACCACCACCACCACCACCACCACCTGAGGATGTGAAGTCTGATCCACCACCAGAAACAGGAGCGGCAGCAGAAGCTCCAACTGATCTGCCACCACCAAGTTCAAGCGAACAACCTGCTGCAGATGTGCCAAAAACTGAAGAAGTGCCACCACCTGCTGATACTGCGTCACAATTACCACCTTCACCAGATGAAACTGGAGGATTGCCACCAGCTGAAGCTGCGCCGCCACCAACATCACCAGCACAAGCGAAGATGGAACCATCGGATAATGCTACTGTGGAGCCCTCAGCGGAAGCAGTTAATAGTTCAGAAAGAGAAGACCTAAATCCTGATCAGTTGATGGCAAACATGGCATCAGCATCAATACCCGAAGTCAAAGTCAGTGGTATCTACGGACCTCGTCCAATCTCTCGTGCCGCATCAGTCAGTAGCTTTATATCAGATGCATCTGACAATGTTTCAATGGAACGGCCTTCATTTGATCTAGTAGAGAAAATGCATTACCTCTTCATCAGGGTAGTTAAAGCTAGATCATTGCCTACTCCAGGCAATCCTGTGGTTAAGATTGTTGTATCTGGCAGCCAAGTCACGTCCAAACCAGCCCGGAAAAACGTGTTGTTCGAGTGGGACCAAACTTTTGCATTCGCTAACGAATCTGCAGATTCCAGCTCCCTCCTCGAAGTCTCTGTGTGGGACCCACTAATCTCAACCTCAACCACTGACATGGCCGGTCATAACTTCCTAGGTGGCATATGCTTTGATGTCACTGAGATTCCGCTGCGTGATCCACCAGATAGTCCATTGGCTCCACAATGGTACAGACTTGAAGGAGGTGGTGCCCACAGAGGGGATCTCATGCTTGCCACCTGGGTTGGCACACAAGCAGATGAATCTTTTCCAGAAGCATGGAAAAACGACACCCCTGGAAATCCTAGCTCCCGGTCCAAAGTCTACCAATCACCGAAGTTGTGGTACTTAAGATCAACGGTGATTGAAGCCCAAGACATATCAGGATCTGCGCCATTAAGAGACTCTACCTTCCAAATCAAAGCTCAATTAGGCTTTCAAGTACAGAAAACCAAGCTCTCCATGACGCGCAGTGGATCCCCCTCATGGAATGAAGACTTAATGTTTGTTACATCCGAGCCATTTGGTGACCAACAGTTATTGTTATCTTTAGTGGAATACCGGGAACCTAAACAACAGGTTGTCATAGGAGTTGCTAGCATGCCACTCGTATCAATTGAACGCCGTGTTGATGACCGGAACGTGGCATCACGGTGGTTCACATTTGAAGATCCTAATCAAGAGAAGAGGGTGTATAAGGGCAGAGTCCACTTGAGGTTATGCTTTGATGGCGGATATCACGTGATGGATGAAGCTGCTCATGTATGCAGTGATTATCGTCCAACTGCTAAGCAACTCTGGAAACCTCCAATTGGAACAGTCGAGCTAGGGATAATTGGATGCAAGAACTTGTTACCAATGAAAACAATTGATGCCAAAGGATCTACAGACGCATATGCTGTTGCTAAGTATGGTAACAAATGGGTACGTACTCGCACAGTCTCTGATACTCTGGATCCCAGATGGAATGAGCAGTACACGTGGCGGGTCTACGATCCATCCACTGTGTTGACAATTGGTGTGTTCGATAGTTGGGAAGTATTTGAATCAGACAGCTTCAAAGAATCGACACGACCTGATTTTAAAATTGGAAAGGTGCGCATACGGATATCAACATTAGAAACAGACAAGGTGTACAAGAATACATACCCGCTGCTTCTGTTATCTCCTTCTGGATTGAAAAAAATGGGTGAAATAGAGGTGGCTGTACGATTTGCGCGTGCAGCTCCCACACTGGATGTTTTGCACTTGTACTCTACGCCATTGTTGCCTATAATGCACCATATTAAGCCCATCGGTGTAATGCAACAAGATATATTGAGATCAACGGCTGTGAAGATTCTATGCACGCACTTCTCACGATCTGAGCCTCCCCTTCGACGTGAAGTAATCACGTATATGCTAGATGCTGATTCACAGGCATTCAGTATGAGGAAAGTTCGTGCCAACTGGTTCAGAATCATTAATGTGATTGCAGGGCTCATTGACACTGTCAAATGGATTGATGACACTCGTTCTTGGCGAAATCCAACATCTACAACCTTAGTACATGCATTGCTGGTGATGCTTGTCTGGTTTCCTGATTTAATCATCCCTACATTGGCATTCTACATGTTCGTAGTTGGAGTATGGAACTACAGGCTGAGGTCCCGGAACCCACTGCCACATTTTGATCCAAAGATCTCATTAGCTGAAAAGATTGACCGCGATGAACTTGAAGAGGAGTTTGATTCGATGCCGTGTAGCAGAACAAACGAGATGGTTCATGCCAGGTATGACAAGTTACGGGTTCTTGGTGCACGAGTTCAAACCGTATTGGGCGATTTCGCCACACAAGGGGAACGTGTACAGGCGCTGGTGACATGGAGGGATCCCCGTGCCACAGGGATCTTTGTAGGATTGTGCTTTGTGGTGGCATTTATATTGTACTTGGTGCCATCTAAAATGGTAGCTATGGCCTCCGGATTTTACTTTTTGAGGCACCCAATATTTAGAGATAGAATGCCTTCCCCGGCTCTAAACTTCTTCAGGAGGCTGCCTTCACTTTCTGATCGAATCCTTTAA

>Dacar014077

ATGGCTCAGCTGGTGGTGGAAGTTATAGATGCGAGTGATCTGATGCCGAAAGATGGGCAGGGCTCGGCGAGTCCTTTTGTGGAGGTGGAGGTGGAAGATGAGAGGCGGCGTACGCAGACTAAGGTGAAGGATCTCAATCCTTATTGGAATGAAAAGATGGTGTTTAACATGAATGATCAGCAGGATCTTATGAGGAAAACGATTGAGGTTACAGTTTATAATGAGAAAAGCAGTGATGGGCATCACAAGAATTTTCTGGGTCGTGTCAGAATCTCTGGCCTTTCTGTCCCTTTATCCGAATCTCAGGCTCAGGTACAGAGATACCCTCTGGATAAGCGTGGTATATTCTCTCATATTCGCGGAGATATTGCTCTTAAATTGTATGTCATAGGTGGTGCTGGTGGTGGCCATGATCACTTTTCAACGAATTTCAGTGCCTCTCATGATGGTGGCCGTGATCACTTTTCAACTAATCATAGAGCTCAGGATGGCGCTGGCCATGATCAGTTTTCGACGTATAGAGCTCAGGAGACGGAGGAAGTACAAGCAACACGAGTGAAGAAGAGCAGCCATTTGGAGCAGAGTACACATAATCCAGTGCTGCAAGAAATTAACACCAACTATAGGGTTAATGATGATGAGTACAAGGACATTGAAAAGAAACTGAAGAAGAAACACAAGGAAAAAGAAGTGAGAACTTTTTACTCTGTTGGAACAGGTGGTGGAGGACCACCACCTGCTTCGATTCCTATGCAAAGGCCGGTCACTATGGAGCCTCCTAGACCCATGGAGTCTTCCAGAGCTGATTTTGCCCGACAGGGGCCTGGGCCCGGACCTGCCACCGTAATGCAGATGCAGTTTCCAGGACAGAGGCCTGAGTTTGGATTGGTGGAGACTCGGCCACCAGTAGCAGCTCGGATGCGTTATGGGGACAAGATGGACAAGATGTCAAGTACTTATGATTTGGTGGAGAAGATGCACTACATGTATGTGAATGTTGTGAAAGCTAGAGATCTCCCTGCAATGGACATTACTGGAAGCCTTGACCCGTATGTGGAGGTAAAGGTAGGGAACTACAAAGGCTTGACGAAACACCTGGATAAGAACCAGAATCCGGTATGGAACAGTATCTTTGCATTTTCTAGAGAAAGATTGCAGACCAATTTGATTGAAGTAATAGTGAAGGATAAAGATATCGGTAAGGATGACTTTGTTGGGAGGGTGGCATTTGATATTGCAGAAGTGCCCCTTCGTTTGGCACCGGATAGTCCATTGGCTCCTCAATGGTATAGACTAGCAGACAAAAGAGGGGAAAAGCCTACTAGAGGAGAAATCATGCTTTCGGTTTGGATAGGAACACAAGCCGACGAGGCCTTTCCTGAGGCCTGGCACAACGATGCTCACAACATCAGTCACCAGAACCTTGCTAATACACGATCGAAGGTTTATTTCTCACCAAAGTTATACTACCTTCGAGTTCATGTTATCGAAGCTCAGGATCTTGTGCCAATTGAGAAAACTAGACAGCTGGACACACATGTAAAGGTCCATCTCGGAAATCAAATCAGGGTCACCAAGCCTTCCCCAGTGAGGCATATAAATCCTGTCTGGAATGATGAGCTTATGTTTGTAGCCTCAGAGCCATTTGATGAATTCTTAACTCTTACGGTGGAAGAACGGCATGGACCGGGGGAGCCAATAGGGAGAGTGATCATACCTGTAAGAGAGATACCACCGAGAATGGACTGGCATAAGCCCCTGGATCCTCGCTGGTTTAATCTTCACAAGCCTTCTCACGCTGGAGACGAAGTTGACAAGACAAAAGAAATTAAGTTTTCCAGCAAAATACTCCTCAGGCTTTGTTTAGATGCGGGTTACCATGTCCTCGATGAGTCTACTCCTTTTAGTAGTGACTTTCAGCCTTCATCAAAGTTTCTGAGAAAGCCTAGCATTGGAATTCTCGAATTGGGGATTTTGAGTGCAAAGAATTTGCTCCCAATGAAAAGCAGCCATGGAGGAAGTACAGATGCTTATTGTATAGCCAAGTACGGAAATAAATGGGTTCGTACCAGGACACTTCTTGACACTCTCTCTCCCAGGTGGAACGAGCAGTATACCTGGGAAGTTTACGACCCCTGCACGGTGATCACAATTGGTGTATTTGATAACTTCCATGTTAATGGAAACAGAGAGGACGCAAGAGATCAGAGAATAGGTAAAGTACGAATTCGTCTTTCAACTTTGGAAACAGATAGGATTTATACCCATTACTACCCTTTGTTGGTTCTGGACCGTTCTGGTTTAAAGAAGCACGGAGAACTGCAGTTAGCTGTAAGATTCACTTGCGTAGCTTGGGTCAATATGGTGGCACAATATGGAATGCCATTGCTACCCAAGATGCATTATGCCTATCCCATTTCAGTACGTCACATTGACTGGCTTCGACACCAGGCAATGCAGATAGTAGCAGCGCGTCTATCTAGGGCAGAGCCACCTCTCAGGAGGGAGACTGTGGAGTATATGTTGGATGTTGATTACCATATGTGGAGCCTCAGGCGAAGCAAAGCAAACTTCTACCGCATAATGTCACTGCTCTCTGGGGTTTCGGCTGTCTGCAGATGGCTAGATGGAATTTGCAAATGGAGAAACCCCTTAACAACATGTCTCGTGCATGTTCTTTTCTTGATTCTGGTTTGCTATCCAGAACTGATCTTGCCAACAATATTTCTCTACTTGTTTGTGATTGGACTATGGAACTACAGGTTCAGGCCAAGAAAGCCACCACACATGGATGCAAGGATTTCACAAGCTGAGAACACTCACCCGGATGAGCTTGATGAGGAATTTGACACATTTCCAACTTCTAGGCCATCAGACCTTGTGAGAATGAGGTATGATAGAATGCGGAGTGTGGCCGGAAGAGTCCAAACAGTGGTCGGAGATTTGGCAACACAAGCAGAGAGAGCTTTAGCAATACTTAGCTGGAGAGATTCAAGGGCCACGGCAATCTTCATTATCTTTGCATTAGTTTTGGCAGTAGTGCTTTACGTAACTCCCTTCCAAGTCATTGCAGTTCTAGTCGGTCTGGTCATGCTGCGCCATCCTCGGTTCAGAAACAGGATGCCTTCTGTACCTGTCAATTTCTTCAAGAGACTACCATCCAAGTCGGATATGCTTCTATTATGA

>Dacar018531

ATGAACAACAACAAACAAAAGCTTGTAGTGGAAGTGATAGGAGCCCATAACTTAATGCCAAAAGATGGTGAAGGATCATCATCGCCATTTGTTGAAATCGAGTTTGAGAATCAGAGACAAAAGACGCAAGTCAAGTACAGAGACTTGGACCCTGTCTGGAATGAAAAGCTTGTGTTTTATGTCAACAATGTGGCAGATCTTCCTTACAGGACTATTGATGTCAATGTTTTTAATGAGAAAAGGTCGAATAATAGTAGAAATTTTCTGGGTAAAGTTAGGGTTTCGGGTTCAAATGTTTGCAGAGAAGGCGAAGAGATTGCACAGCTTTTTACACTGGATAAAAGAAGCTTATTTTCTCATGTTAGAGGTGAGATAAGTTTGAAGATTTTTTTGTCAAGTTCTGAGCAAGTTAAGCATGTTGTTGGTGAAGGAAATGATGGGTTTTCCAAGAAAAACAAGAAGTTGCAGCAACAGCAGCAGCAGCAGCAACAAGGGATGGCTATGGCAGTGAAGCAACAACAAATGGGGCAAGATAATAAGGTTAGTAATCAAAAGATTCAAACTTTGCAAAACCCAAAAGGTGAGAATCAGAATAATGGTGATTTGAAGCCTGTTATTATTACTAATGTGCCTGTTCAAGTTGGCTCCGGCGCCGGAGCCGGTGCCGGAGGTGGGTCCGGTGGTGGTGGGGTTGGTGGGTTTGGGGTAATCTCAAATGGGTCAAGTGAGTTTTTGCTTAAAGAGACTAGACCTCATCTGGGTGGTGGATCTTTGAGTAAAGATTCAACTTATGATCTTGTGGAGCAAATGCAGTATCTTTATGTTAGGGTAGTGAAAGCTAGGGAACTTTTGGGGTTTGGTGGTGGAGAAGTGGTTGCTGAGGTGAAATTAGGGAACTATAGAGGTATTACTAGGAGGGTTGGTCTTAATAATGCTGAATGGGATCAAGTGTTTGCATTTTCAAAAGATTGTATTCAGTCTTCAGCTGTTGAGTTGTTTGTAAAAGAGAGGGATAAAGATGATTTCTTGGGGAGGGTTTTGTTTGATTTGAATGAGGTTCCGAAAAGGGTTCCTCCAGATAGTCAGTTGGCACCACAATGGTATAGAATGGATGATAAGAAAGGTGATAGGTCGAAAGGGGGTGAAGTTATGGTGGCACTTTGGTTTGGAACTCAAGCTGACGAGGCCTTTTCTGAGGCATGGCATTCCAAGTCTGCAAATGTGCATATGGATGGTTTATGTTCGATAAAATCAAAGGTGTATCTTTCTCCTAAGTTGTGGTATTTAAGGGTATCGGTGTTGGAAGCTCAAGATGTTGTTTTGGGAGATAAAGGTTCATCAATGATGAGATATCCGGAGTTGATGGCAAAAGTTCAAGTTGGAAATCAAGTTTTGAGGACTAGAATAGCTCCAGCCACTCCTAACAGGAGCCTACACAACCCATTTTGGAATGAGGATCTGATCTTTGTAGTTGCTGAACCATTTGAGGATTATGTGTTATTTTCTGTTGAGGATCGGGTTGGCCCGAACTGTGATGAGGTTGTGGGGAGAGTTGTTCTCCCACTTACAAATATCGAGAGGAGGTTGGATGATAAACCAGTGGCATCAAGGTGGTACAATCTCGACCTTCGCTATAACAATCCAAATGAGTCTAAAGCTGTGACTAGATTTTCGTCCAGGATACATCTTTGTGCTACACTTGATGGGGGTTACCATGTGCTTGATGAAGCTACAATGTATAGCAGTGATGTTAGGCCCACAGAAAAACGGCTTTGGAAGCCTCACATTGGGGTACTTGAAATGGGGATTCTGGGCGCCTCTAATCTTATGCCGGTGAAGATCAGAGAAGGCAAAGGAGGCTGCACGGATGCTTATTGTGTTGCTAAGTATGGCCAAAAATGGGTGCGAACTCGTACTGTGGTGGACAGTTTAACGCCAAAATGGAATGAGCAATACACTTGGGAAGTGTTTGATCCTTGCACTGTCATCACCATTGGTGTGTTTGATAATGCTCGTGTTGACAAAAATTCTGCCACCGCTGCTGGAATTAGAGATTCTCGCATTGGGAAAGTTAGGATCCGATTATCGACTCTTGAATCTGATAAAGTCTATACTCACTCCTATCCACTGTTAATGTTGCATCCTTCTGGTGTGAAGAAAATGGGGGAGCTTCATTTGGCTGTTAGGTTTACTTGTGCTAATATGCTCAATGTGCTTCAAATCTACACAATGCCTTTACTTCCAAAGATGCACTACGTTCACCCTCTGTCTGTGAATCAGATAGACAGCTTAAGGTATCAAGCACTGAATGCCGTGTCATCAAGGCTCAGTAGAGCCGAGCCACCTCTGGGTAGAGAAGTGGTTGAATACATGCTTGACCATGACTCTCACATGTGGAGCATGAGAAAAAGTAAGGCAAACTTCTTTCGCCTTATGAACGTACTGTCCTGGTTTGTCGCCTTGAACAGGCTAATGGATTCAATGCGGAACTGGCATAAGCCATTGTACTCAACCCTATTTCTCATCAATTTTCTTGTCCTAGTGATGGTCCCTGAACTCATCATACCAACAATATTACTGATCCTGGCTGTTATGGGTCTGTGGCGATACAGAACCAGGCCACGCCAGCCATCTCACATGGATACACGCCTCTCCTATGCTGAAAGTGTCTACCAGGACGAGTTAGACGAGGAGTTTGATTCATTTCCAACAAGCCGAAGTGCAGAGGTGATCCGAATGAGATACGACCGTCTCAGGAGTGTGGCAGGAAGGATCCAAACCGTCGTGGGAGACATGGCTACTCAAGGCGAAAGGCTTCAGGCTTTGATAAGTTGGCGCGACCCAAGAGCCACATTCTTGTTTGTGATCGTCTGCTTATTGGCTGCTTTCGGGTTCTACTTGGTGCCAATCAGATGGGTTGTGGCTCTTTGGGGACTCTATTTCATGAGGCCACCAAGATTCCGGAGCAAGTTGCCCTCAAGAGCTGTTAGCTTCTTCAAGAGGCTGCCAACTAGAGCTGACAGCATGTTATGA

>Dacar019793

ATGACTTCTCCTAACCAAGATGACTTTAAGCTCAAGGACACAAAACCTCAGCTAGGGGAACGATGGCCACATGGTGGTGTACGTGGTGGAGGGGGTTGGATAAGCAGCGAAAGAATCACCAGCACTTACGATCTTGTGGAGCAGATGTACTATCTGTATGTTCGCGTTGTGAAAGCAAGAGATCTTCCTCCAAATCAGATAACAGGGACATCAGATCCATATGTTGAGGTGAAACTAGGAAACTACAAAGGGAAAACCCAGCACTTTACCAAGAGATCAAACCCTGAGTGGAAACAAGTGTTTGCATTTTCAAAAGACAAAATTCAATCTTCAGTTCTCGAAGTATTTGTGAGAGACAAAGATATGGTGGCTAGAGATGATTATTTGGGAAGAGTTGTTTTTGATATGAATGAAGTGCCTACTAGGGTTCCTCCAGACAGCCCTTTGGCACCTCAGTGGTATAGACTTGAAGATCGTCGAGGCGAGAACAAGCTAAGAGGAGAAGTCATGCTAGCCGTTTGGATGGGAACCCAAGCTGACGAAGCCTTCCCGGATGCTTGGCATTCAGATGCTGCAACTGTTCAAGGAGAGGGTGTATATAGCGTTCGCTCCAAGGTGTATGTTTCTCCCAAACTTTGGTATCTGAGGGTAAATGTGATCGAAGCTCAAGATGTAGAATCAGCAGACAAGAGCCAACTCCCTCAGGTTTTTGTCAAAGCTCAAGTTGGGAATCAGATACTTAAGACCAAGACATGCCCAACAAAAACGACGAATCCCTTCTGGAACGAGGACTTAATCTTTGTAGCCGCAGAGCCCTTTGAAGAACAATTAGTGCTGACAGTGGAGAATAAGACAGCCTCAAGAGATGAAACCGTGGGAAGGATACAATTGCCGCTGAACATTTTTGAGAAGAGACTGGATCATCGGCCTTTTCACTCTCGTTGGTTTAACTTAGAGAAGTTTGGTTTCGGAGTCTTGGAAGGAGACAGGAGAATGGAGCTCAAGTTTTCAACCAGAATTCACCTCAGAGCATGTCTCGAAGGTGCATATCATGTACTAGATGAATCAACTATGTACATTAGTGATCAACGGCCTACTGATCGACAACTATGGAAGCAGCCTATAGGTATTCTTGAGGTAGGTATATTAAGTGCTCAAGGGCTTCTTCCAATGAAAACAAGGGATGGCAGAGGAGCCACCGATGCTTACTGCGTAGCAAAATATGGTCAGAAATGGGTCAGAACGAGGACAATAGTCGGCAGCTCTAGTCCGAGATGGAATGAGCAATACACATGGGAGGTTTATGACCCTTGCACTGTGATCACTATAGGGGCATTTGACAACTGCCACTTGGGAGCGAACGAGAAGCCAGGTGGAAAGGACTCGAGGATCGGGAAGGTACGGATCCGATTATCGACCCTCGAAACTGATCGCATCTACACACATTCCTATCCCCTATTAGTCCTTCAACCATCTGGGGTAAAGAAAATGGGCGAACTCCAGCTCGCTTTCCGCTTCACTTGCTTGTCATTAGCAAACATGATCTACCTTTATTGGCACCCTTTGTTGCCAAAAATGCATTACCTTCATCCTTTCACTGTAAGCCAGTTGGATTTTCTGAGATTTCAGGCCATGAACATTGTGGCAGTGAGGCTTGCCCGAGCTGAGCCACCACTACGGAAAGAAGTTGTTGAATATATGCTTGATGTAGATTCTCACATGTGGAGCATGCGAAGAAGCAAAGCAAACTTCTTCAGGATCGTTTCACTGTTTTCTGGGGCTATCTCTTTGAGCAAATGGCTCGGGGAAGTTTGTCACTGGAAGAACCCCATCACATCAGTTTTAGTCCATATACTATTTTTCATACTAATCTGCTTCCCAGAGCTTATTCTTCCAACTATATTTCTTTACATGTTTCTCATTGGAATATGGAATTTCCGATTCAGGCCACGACATCCACCCCATATGGACACTAAACTATCTTGGGCAGAAGCCGTTCATCCGGATGAACTAGACGAAGAGCTACGGAGTGTGGCAGGAAGAATACAGACTGTTGTAGGTGACATGGCAACGCAGGGAGAGAGATTTGAGGCTCTGCTCAGCTGGAGAGATCCGAGAGCAACATGCCTCTTTGTAGTTTTCTGCCTTGTCACAGCCGTTGCTCTGTATGTAACCCCTTTCAGAATAGTCATTCTGTTGGCAGGGCTGTTTGTACTAAGACACCCCAGATTCAGAAGTAAGCAGCCTTCGGTGCCTAGCAATTTCTTCAGGAGATTGCCAGCTCGAGCAGACAGTATGCTTTGA

>Dacar019833

ATGGGCTCACAAGCAAATCCGAACCAAGATGACTACAAGGTCAAGGACACGAAACCTAAGCTAGGGGAACAATGGCCACATGGAGGGGTGCGTGGTGGAGGAGGTTGGATAAGCAGCGAGAGAATCACAAGCACTTATGATCTCGTCGAGCAGATGTACTATGTTTATGTCCGTGTTGTAAAAGCTAGAGATCTTCCTCCTAATCAAGTCACGGGGAGCTCGGACCCGTATGTTGAGGTGAAGCTAGGAAACTACAAAGGAAAAACTCAACATTTGGAAAAGAGAGCCAACCCTGAGTGGAAACAAGTATTTGCATTCTCAAGAGACAAGATTCAAGCTTCAGTGGTTGAAGTTTTTGTGAGAGACAAAGAGATGGTGGCTAGAGATGATTATCTAGGAAGAGTTGTGTTTAATATGCATGAAGTGCCTACAAGGGTTCCTCCAGACAGCCCCTTGGCACCTCAGTGGTATAGACTAGAGGATCGTCGAGGTGAGAGCAAGATAAGAGGGGAAATCATGCTAGCCGTTTGGATGGGAACGCAAGCTGATGAAGCCTTCCCAGACGCATGGCATTCGGATGCTGCTACAGCTCGAGGAGATGGCGTTTTTAGCGCTCGCTCAAAGGTGTATGTTTCTCCCAAACTCTGGTATCTGAGAGTCAATGTAATTGAAGCTCATGATGTAGAATCAGCAGACAAAAGCCAACTCCCTCAGGTATTTGTCAAGGCTCAAGTTGGGAATCAGATACTCAAGACCAGCATGTGTCCAACAAAAACAACCAATCCTTTCTGGAATGAGGACTTGATCTTCGTGGCCGCGGAGCCTTTTGAAGAAAAATTGGTGCTCACAGTTGAAAACAAGACACCTTCAAGAGAGGAAACTGTGGGAAGGATACAATTGGCGCTCAACGTTTTTGAGAAAAGATTAGATCACCGGCCTATTCACTCTCGCTGGTTTAACTTGGAGAAGTTTGGTTTCGGAGCCCTGGAAGGTGACAGGAGAATGGAACTCAAATTTTCAACCAGAATTCACCTCAGAGTATGTCTTGAAGGTGCATATCATGTACTGGATGAATCATCCATGTACATCAGCGATCAAAGGCCTACTGATCGCCAACTATGGAAGCAGCCTATAGGTATCCTTGAGGTGGGTATTTTAAGTGCACAGGGACTTCTTCCAATGAAGACAGTGGACGGCAGAGGAGCCACGGATGCTTACTGTGTGGCAAAGTATGGTCAGAAATGGGTAAGAACCAGAACTATAGTCAATAGCTCTGCTCCGAAATGGAACGAACAGTACACATGGGAGGTTTACGACCCATGCACTGTCATCACTCTAGGTGCATTTGACAATTGCCACTTGGGAGGAAATGACAGTGGCCCCGGAAAGGACTCGAGAATCGGAAAGGTAAGAATCCGATTATCCACCCTTGACACTGACCGGATATATGCACATTCTTACCCCCTTTTAGTCCTTCAACCATCCGGGGTGAAGAAGATGGGGGAACTGCAATTAGCCTTTCGGTTCACTTGCTTGTCTATGGCAAATATGATGTATCTTTATTGGCATCCTTTGTTGCCAAAAATGCATTACCTGCATCCTTTTACTGTGAGCCAGTTGGATTTCTTAAGATTTCAGGCAATGAACATTGTAGCCGCCAGGCTTGGTCGATCTGAGCCTCCACTCCGGAAAGAAGTTGTAGAATACATGCTTGATATAGATTCTCACATGTGGAGCATGAGAAGAAGCAAGGCCAACTTCTTCAGGATTGTTTCACTGTTTTCTGGAGCAATATCTATGAGCAAATGGGTGAGAGAAGTTTGTCACTGGAAAAACCCCATCACTTCGATTCTAGTCCATGTCCTGTTTTTCATACTAATTTGCTTCCCAGAGCTGATCCTTCCGACAATATTTCTTTACATGTTTCTCATCGGAATATGGAACTTCTGGTTGAGGCCAAGACATCCACCCCATATGGATATTAAACTTTCTTGGGCAGAAGCAGTTCATCCAGATGAACTGGATGAAGAGTTTGACACTTTCCCCACATCCAAGGCTCAAGATGTGACGAGGATGCGATACGACAGACTACGCAGCGTGGCAGGGAGAATACAGACAGTGATAGGCGACATGGCAACGCAGGGTGAAAGGTTTCAGGCTCTGCTGAGCTGGAGGGATCCAAGAGCGACGTGCCTCTTCATACTTTTCTGCCTGATTGCTGCCGTTGTTCTTTATGTCACTCCTTTCAGAATAGTCATTTTAGGGGCAGGCTTGTTTCTGCTGAGACACCCCAGACTGAGGAGTAAACAGCCTTCAATTCCCAGCAATTTCTTCAGGAGATTGCCAGCTCGAGCGGACAGCATGCTTTGA

>Dacar019836

ATGATGCAGAGACCTCCCCATGAAGACTTCTCGCTCAAGGAGACCAAACCCCACCTTGGTGGTGGGAAGGCTACTGGTGATAAGCTCACAAGTACGTATGACCTTGTGGAGCAAATGCAGTACCTATATGTTAGAGTTGTAAAAGCTAAAGATTTGCCTGCCAAGGATCTTACTGGTAGCTGTGACCCGTACGTGGAGGTTAAGCTGGGAAACTACAAGGGCACCACACGCCATTTTGAGAAAAAATCAAATCCGGAGTGGTTGCAGATCTTTGCCTTCTCCAAGGATCGAATTCAGGCCTCTGTACTGGAAGTAACTGTGAAAGACAAGGATGTGTTGAAGGATGATTTCATGGGTAGGGTTCTGTTTGACCTCAATGAGGTTCCCAAACGGGTTCCACCTGATAGTCCTCTCGCTGCACAGTGGTATAGGCTGGAAGATAGGAATGGGAATAAACTTAAGGGAGAGCTCATGTTGGCCGTGTGGTGGGGTACCCAAGCCGACGAAGCATTTCCCGAAGCATGGCATTCAGATGCAGCAACTGTTAGTGGTGCTGACAGTCTTTCAAGCATTCGCTCAAAGGTGTATCTCTCACCCAAGCTTTGGTATCTAAGGGTCAATGTGATTGAAGCACAGGATTTGATTCCCACCGATAAGACGAGGTTTCCGGATGTTTTTGTCAAGGCTCAACTAGGAATTCAAACTCTAAGAACAAGAAGTTCTATGAGCAAATCGATCAATCCAATGTGGAATGAAGACTTGATGTTTGTGGCTGCAGAACCATTTGAAGAGCCCTTAATTTTGAGCGTGGAAGACAGAGTTGCTCCAAACAAGGATGAGGTTCTAGGACGGTGTGCTATTCCCTTGCAGTACGTGGACAGGAGGTTAGACCATAAACCCTTGCATACTAAGTGGTACACACTTGAGAAGCATGTCATTATTACTGAGGGAGATAAGAAGAAGGAGATTAAATTCTCAAGTCGGATTCATATGAGAATCTGTCTGGAAGGCGGTTATCATGTATTGGATGAATCCACACATTACAGTAGTGATCTTAGACCGACTGCAAAGCAGTTGTGGAAGTCCAGCATTGGGGTTCTTGAGGTGGGTATCCTGAATGCTCAGGGTTTATCACCGATGAAGACAAAAGATGGAAGGGCAACAACAGATGCTTACTGTGTTGCCAAGTACGGGCAGAAGTGGGTCCGAACACGGACGATCATAGATAGTGCTGCCCCCAAGTGGAATGAACAGTATACCTGGGAAGTTTTTGATCCTTGTACCGTTATAACTGTTGGAGTGTTTGATAATTGTCATCTTCATGGCGGGGATAAGGCTGGTGGAGCAAGGGATTCGCGGATTGGAAAGGTCAGAATTCGCCTTTCTACTCTTGAAACAGACAGGGTTTACACACATTCATATCCGCTTTTGGTTTTGCATCCAACTGGTGTAAAGAAAATGGGTGAAATTCATTTGGCTGTGAGGTTTACATGCTCTTCTTTGCTAAATATGATGAACATGTACTCGCAACCACTATTGCCCAAGATGCATTATGTCAATCCATTAACTGTGAGTCAGCTCGATAGTTTGAGGCACCAGGCCACTCAGATTGTCTCCATGAGGCTTAGTCGTGCTGAGCCACCTCTTAGAAAAGAGGTAGTGGAGTATATGCTTGATGTTGGATCTCACATGTGGAGTATGAGAAGAAGTAAAGCTAACTTCTTTAGAATTATGGGAGTTTTCGGTGGACTGATTGCTGTTGGCAAATGGTTTGATCAGATTTGCAACTGGAAGAATCCCATAACCACTGTTCTGATACACATTTTATTCTTGATTTTGGTATTATATCCAGAACTAATACTACCAACCATTTTCCTCTACCTCTTCTTAATTGGAGTTTGGTACTACAGATGGAGGCCAAGACATCCTCCTCACATGGACACTCGCCTCTCTTGTGCTGATAATGCACATCCTGATGAACTGGATGAGGAATTTGATACCTTTCCAACTTCACGCCCTGCTGACATTGTTAGGATGAGATATGATCGGTTGAGAAGTATTGCTGGAAGAATACAGACTGTGGTCGGTGACTTGGCAACTCAAGGGGAGAGGCTGCAGTCTTTGTTAAGCTGGAGAGATCCCAGAGCTACTGCACTTTTTGTAATTTTCTGCTTGGTTGCGGCAATTGTTCTCTATGTCACTCCTTTCCAAGTTGTGGCCCTTATTGCAGGATTCTATGTGCTAAGGCATCCCAGGTTCCGTCACAAGCTTCCTTCTGTCCCCCTCAATTTCTTCCGGAGATTGCCAGCAAGGACCGACTGTATGCTATGA

>Dacar019837

ATGATGCAAAGACCTCAACATGAAGACTTCTCCCTCAAGGAGACCAAACCACACCTTGGTGGTGGAAAGGCCACTGGTGATAAGCTCACAAGTACATATGACCTTGTCGAGCAAATGCAGTACCTTTATGTCAGGGTTGTTAAGGCAAGAGATTTACCTGCCAAGGATCTTACGGGTAGTTGTGACCCGTATGTTGAAGTTAAGCTAGGAAACTATAAGGGTACAACTCGTCATTTTGAGAAGAAATCAAATCCTGAATGGTTGCAGATCTTTGCCTTTTCCAAGGATCGGATTCAGGCTTCTGTGCTGGAAGCCACTGTGAAAGACAAGGACGTTGTGAAGGATGATTTCATGGGCAGGGTTTTGTTTGACCTCAACGAGGTCCCGAAGAGAGTTCCTCCTGATAGTCCTCTGGCTGCACAGTGGTATAGACTGGAAGATAGGAACGGGAACAAACTTAAGGGAGAGCTCATGTTGGCTGTTTGGTGGGGTACCCAAGCTGATGAAGCGTTTCCAGAAGCATGGCATTCCGATGCGGCAACTGTTGGTGGTGCGGACAGCCTTTCGAGCATTCGTTCGAAGGTGTATCTCTCACCCAAGCTTTGGTATCTCAGGGTCAATGTGATTGAAGCTCAGGATTTGATTCCCACCGATAAGACGAGGTTTCCAGATGTTTTTGTTAAGGCTCAACTAGGAAATCAAGCTTTAAGAACAAGAAGCTCTATGAGCAAATCAATTAATCCAATGTGGAATGAGGACTTGATGTTTGTGGCTGCAGAACCATTTGAAGAGCCCTTAATTTTGAGTGTAGAAGACAGAGTCGCCCCAAACAAAGATGAAGTTTTGGGTCGGTGTGCTATTCCCTTGCAGTACGTGGACAGGAGATTGGATCATAAACCCCTGCATACTAAGTGGTACACACTTGAGAAGCATGTCATTATTACCGAGGGAGATAAAAAGAAGGAGGTTAAATTCTCAAGTCGAATTCATATGAGAATCTGTCTGGAAGGCGGTTATCATGTGTTAGATGAATCCACACATTACAGCAGTGATCTGAGACCGACTGCGAAACAGTTGTGGAAGTCTAGCATCGGGGTTCTAGAGGTGGGAATTTTGAATGCTCAGGGTTTGTTACTGATGAAGACAAAAGATGGAAGGGCGACAACAGACGCTTACTGTGTTGCCAAGTATGGGCAGAAGTGGGTTCGAACACGGACAATTATAGATAGTGCTGCCCCCAAGTGGAATGAACAATATACCTGGGAAGTTTTTGATCCGTGCACTGTTGTAACTATTGGAGTGTTTGATAACTGTCATCTTCATGGCGGGGATAAAGCTACTGGAGCAAGGGATTCACGGATTGGAAAGGTCAGGATTCGCCTCTCTACTCTTGAAACAGATAGGGTTTACACACATTCGTATCCGCTTTTGGTTTTGCATCCAACTGGTGTTAAGAAAATGGGCGAAATTCATCTGGCTGTGAGATTTACATGCTCCTCTTTGCTGAACATGATGAATATGTACTCGCAACCATTGTTGCCCAAGATGCATTACGTTAATCCACTCACTGTTAGTCAGCTTGATAGCTTGAGGCACCAGGCCACTCAGATTGTGTCCATGAGGCTTAGTCGTGCTGAGCCACCTCTGAGAAAAGAGGTGGTGGAGTATATGCTTGATGTTGGATCTCACATGTGGAGTATGAGAAGAAGTAAAGCCAATTTCTTTAGAATTATGGCAGTTTTCGGGGGACTGATTGCTGTTGGCAAATGGTTTGATCAGATTTGCCACTGGAAGAACCCTATTACAACTGTTCTGATACACATCTTGTTCTTGATACTGGTACTATATCCAGAACTAATACTACCAACCATTTTCCTCTACCTCTTCTTGATTGGAGTTTGGTACTACAGATGGAGGCCAAGACATCCTCCTCACATGGACACCCGCCTCTCTTGTGCTGATAATGCACATCCTGATGAACTGGATGAGGAATTTGATACCTTTCCTACTTCACGTCCTGCCGATATTGTTAGGATGAGATATGATCGTTTGAGAAGCATTGCTGGGAGGATCCAGACTGTGGTTGGTGACTTGGCGACTCAGGGGGAGAGGCTGCAATCTTTGCTAAGCTGGAGAGATCCTAGAGCTACAGCACTGTTTGTAATCTTTTGTTTGGTTGCTGCTATTGTTCTCTATGTTACTCCTTTCCAGGTTGTGGCCCTTCTTACAGGATTTTATGTTTTAAGACATCCAAGGTTTCGTTACAAACTGCCTTCTGTACCGATCAATTTCTTTAGAAGATTGCCAGCAAGAACGGACTGCATGTTATAA

>Dacar021536

ATGACAACGCCGTTTCCTCAGCCTGAACCCCAACAGCCACCGCCCGAACCCCCCACCGTCCGCAAACTCATCGTCGAGATCATCGAAGCACGTGACCTCCTCCCCAAAGATGGCCTCGGCAGCTCCAGCGCTTATGTCGTCGTAGACTTCGATGGCCAGAAGAAAAGAACGAGCACAGTCACACGAAACTTGAACCCTGTATGGAACGAGAGCCTCGAGTTTATAGTCTCGGACCCCAAAACAATGGAGTTCGAGCAAGTCGAAGTTGAGGTGTTTAATGATAAGAAATTGAGCCACGGGAATGCCAGGAAGAATCATTTTCTCGGGAGAGTTAAGCTCTATGGGACTCAGTTTGCAAAGAGAGGTGATGAGGGATTGATTTATTTTGAGTTGGAGAAGAAGAGTGTGTTTAGTTGGATTAGAGGTGAGTTAGGTCTTAGAATTTATTATTACGATGAAATTGTTCAGTCGGAACAATCTCATGAGGCGGGGGCTGCTCCTGATCAGCCACTGCCTCCTGATCAGAAGCCGCAGCTCATGGTGGGAGAAGATGGCAGAGTTATGGAGATTCCGATGCAGATGCCGGTTCATACTATTGATGATTCACAGTCACCGCCTTTGGTGACTGTTCAGGAGCAACCGCCAGCTGTCACGGTGCAAATGGAGCATCACCATCATCATCAGTATCAGAACCAGCATCAGAGGCAAATGCAGTATCATAATCAGCCTCCGCAGAATTATCAGGATGTGGCTCCGCCTGTGATGCAGCAGGATGTTCAGCCTGAGGCGAGGAATGATCACGAGAGAGTTAGGGTCTTGAGACGGCCTAATGGAGGAGATTACTCGCCTCGGGTTATTACAGGGAAGAAGTTGAGTGAATCCGAGAGGATTCAGCCGTATGATTTAGTTGAGCCGATGCAGTACTTATTTATTCGAATTGTGAAAGCTCGGGGACTTTCGCAGAACGAGAGTTCCTATGTCAAGATTCGGAGTTCGAATCATTTAGTTCGGTCTAAACCGGCTAGTTTTAGGCCAGGGGAGCAGGCACCAATTCCAGAATGGAATCAGGTGTTTGCTCTTGGTTATAATAAGGATACGGCTAATTCGACGTTGGAGATCTCTGTTTGGGATGGATCTTCGGAGCAATTTTTAGGTGGAGTTTGTTTTGATCTTTCGGATGTTCCTGTTCGTGATCCGCCTGATAGTCCTCTTGCTCCTCAGTGGTATCGTCTTGAAGGCGGAGATGATCCGAACTCTGCTAGAGTTTCTGGTGATATACAGTTGTCTGTTTGGATTGGTACTCAGGCTGATGATGCTTTTCCAGAGTCGTGGAGCTCGGACTCGCCTTATGTTACTCATACGCGCTCTAAAGTTTATCAATCACCGAAGCTTTGGTATTTGAGGCTCACGATACTTGAAGCTCAAGACCTTCAAATTGCTCCTAATTTGCCTCCGCTGACAGCCCCGGAGATTAGAGTGAAAGTTCAACTGGGATTTCAGTCGTCGGTTAGGACAAGACGAGGCTCGATTAATCATCATGGTTCGTCGTTTTTCTGGCATGAGGATGTCATCTTTGTTGCTGGTGAGCCACTTGAAGAATCATTGATACTCCTGGTGGAAGATCGGACTGGGAGTGATCCAGCACTCCTTGGACATGTCATGGTTCCTGTGGGATCAATTGAGCAGCGTTTGGATGAACGACACGTACCTTCTAAATGGTTTGGGTTGGAAGGCGGATCTGGTGGATCTTATCCAGGTCGAATTCACCTAAGGATGTGTCTTGAGGGTGGGTATCATGTTCTTGATGAGGCAGCTCACGTGTGCAGTGATTTCAGGCCTACTGCTAAGCAATTGTGGAAGCCTGCTGTTGGGATTTTGGCGCTTGGCATACTTGGAGCTCGTGGATTGCTTCCGATGAAATCCAAGGGCGAAGGGAAAGGCTCTACTGATGCTTACTGTGTGGCAAAGTATGGGAAAAAATGGGTTCGTACTAAAACCATTACTGACTGTTTTGATCCACGTTGGAACGAGCAATATACCTGGCAGGTGTATGATCCTTGCACTGTCTTGACAATTGGCGTGTTTGACAACTGGCGTATGTTTGCTGACACGTCGGATGAAAAGCCTGATTATCGGATAGGAAAAGTGCGTATACGTGTTTCTACATTGGAGAGTAACAAAGTGTACACTAATTCATACCCTCTTTTGGTTTTGCAAAGAACAGGGTTGAAGAAAATGGGTGAAATTGAAATTGCAGTTAGGTTTGCCTGTCCGTCGTTGTTGCCTGACACGTGTGCGGTTTATGGACAGCCGTTACTTCCAAAAATGCACTACTTACGTCCTCTTGGGGTGGCTCAACAGGAGGCGTTACGTGGTGCTGCTACTAAGATGGTGGCAGCTTGGCTAACGCGATCAGAGCCACCACTTGGTCCTGAGGTGGTTCGGTACATGTTGGATGCTGATTCCCATACATGGAGCATGAGGAGAAGCAAGGCCAATTGGTTTCGGATTGTGGCTGTACTTGCCTGGGTTGTTGGCTTAGCGAAATGGCTGGATAATATTCGAAGGTGGAAGAATCCTGTCACTACAGTTTTGGTTCATATGTTGTATTTGGTGTTGGTTTGGTATCCTGATCTGATTGTGCCAACAGGGTTCTTGTATGTGTTTATGATCGGTGTATGGTACTACCGGTTTAAGCCTAAGATACCAGCCGGAATGGACACCCGAATATCTCAGGCTGAAACGGTCGACCCTGATGAATTGGATGAAGAGTTTGATACGTTTCCAAGTTCGAGGCCACCTGAAATAATACGCATGAGGTACGACAGGTTGAGAACATTGGCAGCACGGATTCAGACCGTGTTGGGTGATTTCGCCACACAAGGCGAAAGAGCTCAGGCGCTAGTGAGCTGGAGGGACCCGAGGGCCACGAAAATGTTCATAGCAGTGTGCCTGTCTATTACCATTGTGCTATATGTGGTTCCAGCCAAAATGGTAGCCGTGGCACTTGGATTCTATTACTTGAGGCACCCAATGTTCAGGGACCCCATGCCCCCAACTAGCTTAAACTTTTTCAGGAGACTACCTAGTTTGTCCGATCGATTAATGTGA

>Dacar029626

ATGGGGAAGCAAAATCATGATCAGAACAAAAACCCTAATCAAAACAAGAATAGTAACCCAAATCAAAACCAGTCCCAGAACCAAAAGTTTAATCCGAAACCGCATTCGAACCCGAATGAGGAATTTGATCTTAGGGAGACGGCGCCACGCCTTGGAGGGGGCAGGTTTCCGGGGAATGATAGAGTTGGGACTGCATTTGATCTTGTTGAGCAAATGCATTATCTTTTTGTTAGGGTGGTTAAGGCCAAGGAGCTTCCGAAAAAACATAATGATCAATTTCCTGACCCTTATGTTGAGTTGCAACTTGGTAATTTTAGAGGTCATACGAAGCATTTTGAGAAAGTGTGTAATCCGGAGTGGAACCAGGTGTTTGCGATTTTGAAAGATCAGATTCAGGCTACGAGTATTGGGTTTTTTGTGAAGGATAAGAATGATAATAACAAGGATCATTTTATTGGGAATGTATGTTTTGATGTTTGTGAGGTTCCGAAAAGGGTCCCACCTGATAGTCCCTTGGCGCCGCAGTGGTATAGATTGGATAATAAAGAGGGGAAAAGGTCTCAAGGGGAGTTGATGTTGGCTGTTTGGATGGGGACTCAGGCTGATGAAGCGTTTCCGGAGGCTTGGCATTTGGATGCTTCTGCAGTTAGTGGTGATGGTGTAGCAAATATAAGATCAAAGGTTTATTTATCACCGAGGCTTTGGTATGTTAGAGTCAATGTAATTGAGGCACAGAGTTTGCGAATAAGTGATAGAAATTTTAGGCAATTGGAGCTTTTTGTGAAGGTTGAGCTGGGAAAAGTGGTGTTGAGGACTAAAAATTCTTCTAGCAGGACTCCGTTATGGAATGAGGATTTGATGTTTGTGGTGGCAGAACCTTTTGAAGAGCCATTGGTTTTGAGTTTAGTGGATAGACAGTCAGCTAATAAGGATGAAGTACTAGGGAAATGTTTTGTGTCTTTACAAAATGTGGAAAAACGGGTGGATCTTAAATTTGCTACAAGTAGGTGGTTTAATCTTGAGAAGCATGGGACGGGAGATAATGGGGCGAAGAATGAAGGGAAGTTGAATAGTAAGATACATTTAAGGATTTGTCTGGAAGGGGGCTACCATGTTCTAGACGAGTTAACTCATTACAGTAGTGATTTTAGGGCGACGGCTAGGCAGTTGTGGACACCCAGCATTGGTGTACTGGAGTTGGGCATTTTGAGTGCTCAAGGACTGTCAAGCATGAAGCACAAAGGTGGGAATGGAAGTACAGATTCTTATTGTGTCGCCAAGTATGGACAGAAATGGATTCGTACAAGGACAATTATGAATACAGATAGTCCTAAATGGAATGAGCAATACACATGGGAGGTTTTCGATCCCTGTACTGTCATTACTATAGGTGTGTTTGATAATCAGCACCTACAAGGGGTCGACAAGGGTGGGGGTGCAAAAGATTCGAAATTAGGGAAGGTAAGAATTCGGCTTTCTACTCTTGAATCTGACAGGGTATATACACATTCTTATCCACTTATAGTTCTGGAGCCTTCTGGAGTGAAAAAAATGGGGGAAATCCAATTGGCTGTACGGTTCACTTGCACGTCTCTAGTCAATTTGCTTCAAATGTATACCCAACCTCTTCTGCCCATAATGCATTATCTCTACCCCCTATCTATGCATCAGATTGATTCTTTACGACATCAGGCTACTCAAATTGTCTCCACCAGGCTAAGTCGTGCTGAGCCACCACTGCGAAAGGAGGTGGTTGAATATATGCTTGATGTTGGTTCAACAATGTGGAGTGTGCGGAAGAGCAAGGCCAACTATTGCAGGATCATAGAAGTGATGACTCGCTTTACTGCATTTTGCCGATTGTTTAATCAGATATGCATGTGGAAGAGCCCTTTTATTACAGTTCTTGTTCATATACTTCTTCTGGTACTAGTTTATTATCCACACATGATTTTGAGCACAATGTGCTTTACTATTTTCATGATAGGGATCTCCAAGTACAGGTTCAGGCCAAGGCACCCTCCCCACATGGATATTAAACTTTCTCTTGCTCATATAACTCATGGAGATGAGTTGGACGAAGAGTTTGATACATTTCCAACATCTAAGCAAGGTGAAGTACTTAAAATGAGATACGACCGACTAAGATGTATTGGTTCCAGGATACAAGTTGTTATCGGTGACTTGGCCACTCAAGGGGAGAGGGTGCACAATTTGCTCAGCTGGCGAGATCCCAGGGCGACATCTCTCTTTCTGATACTCTGCTTCATTTCTGCTATTGTGCTGTATGTTACTCCCTGTCGAGTTATTGCTGTTTTGATGGGTTTTTATGTTTTAAGGCATCCAAGATTTCGCCATAAGCTTCCCTCCACCCCTCTAAGTTTCTTCAGAAGATTACCTGCAAGATCAGATAGCTTGTTGTGA

>Dacar029937

ATGCAGCGTCCTCCACAAAATGCTGAATTTGATCTCAAGGAGACCTCGCCAAAAATTGCTGGATCAGGGGTGATCACTGGTGATAAGCTCACATGTGCCTATGACCTTGTTGAGCAGATGCAATACCTCTATGTCCGTGTCGTGAAGGCCAAGGATCTACCAGGAAAAGATGTCACTGGTAGTTGTGATCCATATGTTGAAGTAAAGCTGGGAAACTACAAAGGAGTGACAAAGCATTTTGAAAAGAAACCAAACCCCGAGTGGGATTATGTGTTTGCCTTTTCGCAGGACAGGCTCCAGTCTTCGTTTGTTGAAGTAGTGGTGAAAGACAAGGATGTTGTTATAGATGACTTCATTGGGAGGATTCTGTTTGATCTTGTTGATGTTCCTAAGCGCGTGCCACCAGATAGTCCACTCGCGCCCCAATGGTATAGATTGGAAGATAAAAGAGGGCAAAAGATGAAGCATGGGGAGATCATGGTTGCTGTTTGGAAAGGAACTCAGGCTGATGAGGCCTTTCCTGATGCTTGGCATTCAGATGCAGCTAGTGTAGGTAGAGAAGGGATCACCAAGATTCGAGGGAAGGTTTATTTATCTCCTCGCCTTTGGTATGTACGCGTAAATGTTATTGAATGCCAAGATTTGCTTCCTAGTGACAAGGGTAAAGCCCCCGAGGCATCAGTTAAAGTCATTCTTGGAAATCAGGCTTTGAAGACCAGAATTTCTCCAGTCAAGGGTGTTAATCCAATGTGGAATGAGGACTTGGTTTTTGTAGCTGCAGAACCATTTGAGGAACCTTTGGTCATAACTGTGGAAGATAAGGCTAACAAAGACGATTTCATGGGGAAATGTGTGCTTCCTTTGACAAGTCTCCACAGGAGGCTTGACAATAAGGCTGTGCCTTCTAGGTGGCATAATCTTGAAAAATATGCAGTTGTTGAAGGTGAAAAGAAGGAGCTGAAATTCGCCAGTAGGATTCATCTGAGGCTGAGTTTGGATGGCGGATATCACGTTCTGGAAGAGTCCACTCACTATTGTAGTGACCTGAGGCCAACTTCAAAGCTGTTGTGGAAGTCCAGCATTGGCCTTTTAGAGCTGGGAATCATAAGTGCTACTGGACTTTCTCCTATGAAGACGAGGGATGGCAGAGCAACAACGGATGCCTTTTGTGTGGCTAAATATGGTCCGAAATGGGTAAGGACAAGGACTATTATAGACAGCTTTGGTCCAAAATGGAACGAGCAATACACATGGGAAGTTTTTGATCCATGTACTATGATCACAATTGGTGTGTTTGATAACGGACAATTACATGGAGGGGGAAAGGATTCAAGAATTGGGAAGGTCAGGATTCGGTTGTCAACTCTGGAGACAGAACGAGTCTATACACATTCTTATCCGCTTATTGTGTTGCAGCCCTCAGGAGTTAAGAAAATGGGAGAAGTTCAGTTGGCTGTGAGATTTTCATGCACTTCATATGTTAACATGTTGAGCAAATATTCACAGCCAATGCTGCCAAAAATGCACTATGTCCATCCCTTATCCGTCATCCAAATGGATATTTTGAGGCACCAAGCTACTCAAATAGTTTCAGTCAGGCTCAGTCGCGCTGAGCCTGCACTTAAAAAAGAAGTTGTAGAGTTTATGCTTGACGTGGGCTCACATATTTGGAGTGTCAGAAGAAGCAAAGCCAACTTCTTCAGGGTCATGAATGTCTTGAGCAGTGCTGTTGCAATTGGCAAATGGTTTGACAAGATTTGCAACTGGAAAAATCCTATAACAACGGTCTTGATTCACGTACTATTTGTAATTCTAGTTCTTTTTCCTGAACTGATACTCCCAACAACTTTTTTGTACCTTTTCTTCATCGGCTTGTGGAGGTACAGATGGAAGCCGAGGCATCCTCCCCATATGGATATACGTCTGTCACACGCTGATGCAGTTGGTTCTGATGAATTAGACGAAGAGTTTGACACGTTCCCTACTTCCAAAGGCTCAGATACGGTTAGAATGAGGTATGATCGGCTGAGGAGCATTGGAGGGAGGATTCAGACTGTGGTGGGAGACTTGGCAGCCCAGGGAGAGAGGCTGCAGGCTCTGCTGAATTGGAGAGATCCGAGGGCTTCTGCGTTGTTTGTTACGTTCTGTTTGATGGCTGCAATAGTGCTCTATGTTACGCCTTTCCAAGTGGTGGCGCTTCTGATGGGATTCTACGTGTTGAGGCATCCGAGATTTCGACAGAAGCTTCCTGCATTGCCTGCTAATTTCTTCCGGAGGCTGCCTGCGAAAACAGACTGCATGATATGA

>EucgrA02435

ATGACTTCGCCAGCCCCTCCACGCACCCAAGAAGATTATAAGTTGAAGCACACGAAACCTCAGCTCGGGGAACGATGGCCCCATGGAGGTGTCCGAGGTGGGGGCGGATGGATCACGAGCGAAAGAGCAGCAAGCACTTACGACCTTGTCGAACAGATGTACTATCTCTACGTGCGGGTCGTGAAAGCCAAGGATCTTCCCCTGAATCCTATCACGGGAAACTGTGACCCGTATGTCGAAGTGAAGCTCGGAAATTACAAAGGCAAGACGCGGCACTTCGAAAAGAAGACAAACCCTGAATGGAAACAGGTTTTCGCCTTCTCCAAAGAAAAGATCCAGTCCTCTGTTCTGGAAGTATTTGTCCGAGACAGAGAAATGGTCGGGCGAGATGAGTACTTGGGCAAGGTGGTGTTCGACATGCATGAAGTGCCGACTAGAGTTCCACCAGACAGTCCTCTGGCACCCCAGTGGTACAGGTTGACAGACAGGCGAGGAGAGACCAAGGTGAAAGGAGAAGTTATGCTTGCGGTTTGGATGGGAACGCAAGCCGACGAAGCCTTCCCTGAGGCCTGGCATTCGGACGCAGCATCAGTCCATGGAGAGGGAGTATATAATATCCGGTCCAAGGTTTATGTTTCCCCTAAACTGTGGTATCTCAGGGTGAACGTGATTGAAGCTCAGGATGCCGAGCCCCTTGACCGGAGCCAAGTACCGCAAGCTTTTGTGAAAGCACAAGTCGGAAACCAGGTACTCAAATCCAAGCTCTGTCCTAACCGAACAACACATCCATTCTGGAACGAGGACCTGCTATTTGTAGCAGCAGAGCCTTTTGAAGAGCAGTTGGTGCTAACAGTCGAAAATAAAGTGAGCCCTGCAAAGGATGAAGTCATGGGCAGATTGATATTGCCGCTAACAGTCTTCGAGAGGCGCCTGGATCACCGGCCAATTCATTCGCGCTGGTTCAACCTCGAGAAATTCGGGTTCGGTGCCCTGGAAGCAGATAAAAGGCACGAACTCAAGTTTTCGAGCAGGATCCATCTGAGAGTTTGTCTTGAAGGTGCTTATCATGTGTTGGACGAGTCCACCATGTACCTCAGCGACCAGCGGCCAACAGCACGAGAGCTGTGGAAGCATCCTATCGGCATACTTGAAGTGGGTATTCTAAGCGCTCAAGGGCTTCCTCCCATGAAGACGAAGGACGGCCGAGGAACCACCGACGCCTACTGCGTCGCCAAGTATGGACAGAAGTGGGTGAGAACAAGAACTATCCTCGAGAGCTTGAGCCCGAAATGGAATGAGCAGTACACATGGGAGGTCTATGACGCCTGCACTGTGATCACAATGGGAGTTTTCGACAACTGCCACTTGGGTGGGAATGAGAAATCGGCAGGTGGTGGTGGGGCAAAACCTGATTCAAGAATTGGGAAGGTAAGGATTCGGCTATCGACCCTCGAAACAGACCGAATATACACTCACTCCTATCCGCTGCTCGTCCTCCACTCGTCGGGGTTGAAGAAAATGGGAGAGCTCCAATTGGCGGTTCGATTTACCTCTCTTTCGCTCGCGAACATGATCTACCTCTACATGCAACCTTTGTTGCCCAAGATGCACTATCTGAATCCATTCACAGTGAATCAGCTCGACAGTTTGCGGTACCAGGCCATGAACATCGTAGCAGTGAGGCTCGGCAGGGCAGAGCCGCCGCTCAGGAAGGAGGTGGTGGAGTACATGCTGGACGTGGATTCCCACATGTGGAGCATGAGAAGAAGCAAAGCTAACTTCTTCCGCATTGTCTCGCTCTTCTCAGGCGTCATATCTATGGGCAGGTGGCTAGGCGAAGTCCGCCACTGGAAGAACCCCGCCACGACGGTCCTAGTTCACGTCCTGTTCTTCATATTGATATGCTACCCGGAACTAATACTTCCGACTATTTTCTTGTACATGTTCCTGATCGGGATATGGAACTATAGGTTCCGGCCGAGGCACCCGTCTCACATGGATCCAAAGCTATCATGGGCGGAAGCGGTCCATCCAGACGAGCTAGATGAAGAATTCGATACATTTCCTACATCCAAGCCTCAGGATGTGGTGCGGATGAGGTACGACAGGCTCAGGAGTGTGGCTGGGAGGATCCAGACCGTGGTGGGCGACATGGCCACGCAAGCGGAAAGATTTCAGGCCCTTCTCAGCTGGAGAGACACGAGAGCTACCAGCCTCTTCATATTTTTCTGCCTCGTTGCAGCTGTGGTTCTTTACATAACTCCGTTTAAGATAGTGGCCCTGGCCGCGGGCATAGTCTGGCTTCGGCATCCGAGGTTCAGGAGCAAGCTTCCCTCGATCCCGAGCAACTTCTTCAAGAGGTTGCCGTCTCGGGCCGACAGCATGCTCTGA

>EucgrA02437

ATGATGCACAGGCCTCCTCATCTTGAGGACTTCGCTCTGAAGGAGACCAAGCCCCATCTCGGTGGAGGGAAGGTCTCCGGCGACAAGCTCACGAGCACCTATGACCTCGTTGAGCAAATGCAGTACCTCTATGTCCGCGTTGTTAAGGCCAAGGAGTTGCCTGCGAAAGATGTCACCGGGAGTTTGGATCCTTATGTTGAAGTCAAGCTTGGGAACTACAAGGGCACGACCCGGCACTTTGAGAAGAAATCAAATCCTGAGTGGAATCAGGTTTTCGCTTTCTCAAAGGATCGGCTTCAGGCCTCGATGCTGGAGATCATTGTCAAGGATAAGGATCTCGCGAAAGATGATTTCGTGGGTCGAGTTGTGTTTGACTTGAACGAGGTCCCGAAGCGGGTTCCGCCGGATAGTCCTCTAGCGCCGCAGTGGTACAGGCTGGAGGACAGAAAGGGGGATAAGGTGAGGGGAGAGGTAATGTTGGCTGTTTGGATGGGCACTCAAGCTGATGAAGCGTTCCCAGAAGCGTGGCATTCTGATGCAGCTACAGTTAGCGGGACCGATGCCCTTGCGAATATCCGTTCAAAGGTATATCTTTCTCCTAAGCTTTGGTATTTGAGGGTCAATGTTATCGAAGCTCAGGATCTCCAGCCTAGTGACAAGGGTCGATACCCGGAGGTTTATGTCAAGGCTAAGCTTGGAAATCAGGTTTTGAGAACTAGAGTTTCTCAGAGCAGGAGTATTAACCCATTGTGGAATGAGGATTTGATGTTTGTTGCGGCCGAACCATTCGAGGAACCTCTGATTTTGAGTGTGGAAGATCGAGTTGCTCCCAACAAGGATGAGGTGTTGGGACACTGTGGAATCCCGCTGCAGTATGTGGACCGGAGATTGGATCACAAACCTGTGAACACAAGGTGGCATAATCTCGAGAGGTATATCATTGTAGAGGGAGAGCAGAAAAAGAAAGACACTAAGTTCGCAAGCAGGATCCACCTGAGGATATGCTTGGAGGGTGGTTATCATGTTTTGGATGAGTCGACGCATTACAGTAGTGATCTTCGACCCACTGCGAAACAGCTGTGGAAGTCGAGCATTGGGATATTGGAGCTAGGAATTCTGAGTGCTCAGGGGCTGTTGCCGATGAAGACCAAAGATGGACGGGGAACGACTGATGCTTATTGTGTGGCAAAATACGGGCAGAAGTGGGTCAGGACGAGGACCATCATAGACAGCTTTATGCCTAGGTGGAATGAGCAATACACTTGGGAAGTTTTTGATCCTTGCACTGTCATCACAATTGGCGTATTTGATAACTGCCATTTGCATGGAGGGGATAAGAATGGGCCTGCAAGGGATTCGAAGATCGGCAAGGTGAGGATTCGTCTCTCTACCCTTGAGACTGATAGAGTCTATACCCATTCGTATCCGCTCCTTGTTCTGCACCCAAATGGAGTGAAGAAGATGGGCGAAATCCATCTAGCCGTGAGGTTCACTTGCTCTTCTTTGCTCAACATGATGCACATGTATTCACACCCACTGTTGCCCAAAATGCACTATATTCATCCATTAACTGTGAGTCAGCTAGATAGCTTGAGGCACCAGGCCACTCTGATTGTGTCAATGAGGCTGAGCCGGGCGGAGCCGCCACTGAGGAAGGAGGTTGTGGAATACATGCTTGATGTGGGTTCACACATGTGGAGCATGAGGCGAAGCAAGGCCAACTTTTTCAGAATCATGGGGGTCTTGAGCGGTCTAATTGCTGTGGGAAAATGGTTTGATCAGATATGCCATTGGAAAAACCCCATAACAACCGTGCTGATTCACATCCTGTTCATCATACTGGTTATATATCCTGAGCTCATCCTGCCCACGATTTTCCTCTATCTTTTCATGATTGGAGTGTGGTACTACAGATGGAGGCCGAGGCATCCACCTCACATGGACACCCGCCTCTCGCATGCAGATTCTGCACATCCTGATGAGCTTGATGAAGAATTTGACACTTTCCCCACTTCCCGACCAACTGACATTGTGAGGATGAGATATGATCGCCTGAGGAGCATTGCTGGGAGGATCCAGACAGTCGTTGGGGACTTGGCTACTCAGGGGGAGAGGCTTCAATCCTTGCTCAGCTGGCGCGACCCAAGGGCGACTGCTCTGTTTGTCATATTCTGTTTGGTCGCTGCCATTGTTCTGTATGTCACTCCTTTCCAAGTCGTGGCACTCCTCACAGGATTCTACGTGTTGAGGCACCCGAGGTTCCGGCACAAGCTTCCTTCCGTGCCACTCAACTTCTTCAGGAGGCTACCGGCAAGAACCGACTGTATGTTGTGA

>EucgrB00432

ATGGCCACCGCCACGTCGCCGCCGCCGCCGCAGCAGCACCAGCCGAGGGCCGTGCGGAAGCTCCTCGTGGAGGTGATCGACGCGCGGGACCTGCTGCCCAAGGACGGGCAGGGCAGCTCCAGCCCGTACGTCATCGTGGAGTTCGACAACCAGAAGAGGCGCACCTCCACCCAGTACCGCAACCTCAACCCGGCATGGCACGAGGCCCTCGAGTTCGTCGTCTCCGACCCGGAGAACATGGCCTACGAGGAGCTCGACGTCGAGGTCTTCAACGACAAGAGGTACGCCGCCGGCGGCGGGGGCCGGAAGAACCATTTCCTCGGCCGGGTCAAGCTGTACGGGTCCCAGTTCCAGAGGCGCGGGGAGGAGGGCCTGGTGTACCACCAGCTGGAGAAGAAGAGCGTCTTCAGCTGGATTCGCGGCGAGATTGGGCTGAGGATCTGCTACTACGACGAGCTCGTGGAGGAGCATCCGCCCCCACCGCCCCAGACGCCGCCGCCGGAGGAGGCGGCGCAGCATCATCACCCTCCTCCTCCGGGTGCGGAGCTCCACGAGGGGCAGGGGGTGGCGGTGGGGATGAAGCCCCCGCCGGTGATGGTGGTGGAGGAGGGCAGGGTCTTTGAAATGCCGCCGGGGGAATGCTGCCCGCCGGCGGTGCCGCTCCGGCGCCCCGACCGGTCGCCCTCCCCGCCCGTGGTCGTCATCGAGGAGTCGCCGCCGCCGTCCCACACGGTGCACTTCCGCCCCGGGCCGCCCCCGCCCGAGGCGATGCAGCCGCAGCCGCAGGTGACGACGTACCAGCACCTCCCGGGGGCCGCTCACCCCGCCCAGGCGGCGGCTGCGGCGGTGGCGGCGGCGGCGGAGGCGGCGATGCAGTACCCCCCGCCGGAGGTGCGGCGGATGCAGGGCGCGCGGGTCGGGGAGAGGGTCCGGGTCGTGCGGCGGCCGGCGAGCGGCGATTTCTCGCCGCGGGTGATATCGTCGCGCCACCAGAGGTTCGCGTCGGAGACGGAGCGGATCCACCCCTACGATCTCGTCGAGCCGATGCAGTACCTGTTCATCCGGATCGTCAAGGCGCGCGGCCTCGCCCACAACGACAGCCCCTACGTCAAGGTCGGCACCGCCACCCACCGCGTCCGCTCGAAGCCGGCGGTCCACCGCCCCGGCGAGCCGACTGACTCGCCGGAGTGGCACCAGGTCTTCGCCCTCGGCCACAACAAGCCCGAGTCGACCAGCTCGACCCTCGAGATCTCCGTTTGGGACTCCCCATTGGAGAGCTTCCTCGGCGGCGTCTGCTTCGACCTCTCCGACGTGCCCGTCCGCGACCCGCCCGACAGCCCGCTGGCGCCGCAGTGGTACCGCCTCGAGGGCGGCGACGCGGCGGAGCGGAGCCGCGTCTCCGGCGACATCCAGCTCTCCGTCTGGATCGGCACCCAGGCCGACGACGCGTTCCCGGAGGCGTGGAGCTCCGACGCGCCGCACGTGGCCCACACGCGCTCCAAGGTTTACCAGTCGCCGAAGCTGTGGTACCTGCGGGTGACGGTGATCGAAGCTCAGGACCTCCACATCGCCTCGAACCTGCCGCCGCTGACGGCCCCGGAGGTCAGGGTCAAGGCGCAGCTCGGGTTCCAGTCGGTGCGGACGCGGCGGGGGTCCATGAGCAACAACGTGCCGTCGTTCCACTGGAACGAGGACCTCGTCCTCGTCGCCTGCGAGCCCCTGGAGGACTCGCTCATCTTGCTGGTGGAGGACCGGTCGAACAAGGAGACGCTGCTCCTCGGGCACATCCTGATTCCGGTGGCCTCGATTGAGCAGCGGATAGACGAGCGGCATGTCGCCGCCAAGTGGTTCCCGCTCGAGGGCGGCGCCGGCGGCGGATGCGGAGCCGGGCCGGGGCCTTACTTAGGCAGATTGTACCTGAGGCTGTGCTTGGAAGGCGGTTATCACGTTCTCGACGAGGCCGCGCATGTGTGCAGCGACTTCCGGCCCACGGCGAAGCAGCTGTGGAAGCCGCCGATCGGGATCCTGGAGCTCGGGATCCTCGGCGCACGCGGGCTCTTGCCCATGAAGTCGAAAGGCCCCGGCAAAGGGTCCACGGACGCCTACTGCGTGGCCAAATACGGCAAGAAGTGGGTTCGGACCCGCACCATCACAGACAGCTTCGACCCGCGGTGGAACGAGCAGTATACTTGGCAGGTCTATGACCCGTGCACTGTGCTCACCGCCGGGGTTTTTGATAATTGGCGGATGTTTGCCGACCCGTCGACCGATGAGAGGCCGGATTGCTGCATGGGGAAGATCCGGATTCGGGTATCCACATTGGAGAGCAACAAGGTGTACACCAATTCGTATCCGCTGCTGGTGCTGACGAGAACTGGGTTGAAGAAGATGGGCGAGATCGAATTGGCGGTCCGGTTCGCCTGCCCGTCGCTGTTGCCGGATACTTGCATAGTCTATGGTCAGCCCCTGTTGCCGCGGATGCACTATCTCCGCCCACTCGGGGTGGCCCAGCAGGAGGCGCTGCGAGGGGCCGCCACCAAGATGGTAGCCGCATGGCTCGCGAGGTCAGAGCCTCCGCTCGGCCCGGAAGTGGTCAGGTGCATGTTGGATGCCGATTCGCACACGTGGAGCATGAGGAAGAGCAAGGCGAACTGGTTCAGGATCGTGGCCGTGCTGGCGTGGGCGATCGGGCTGGCGAAATGGTTGGACGACATCCGGAGGTGGCGGAACCCGGTCACGACGGTCCTAGTCCACATACTGTATCTGGTGCTCGTGTGGTACCCGGATTTGATCGTGCCTACCGGGTTCTTATACGTGTTCCTGATCGGCATCTGGTACTACAGATTCAGGCCGAAGATCCCCGCCGGGATGGACACCAGGCTCTCGCAGGCCGAGTCGGTCGACCCGGACGAATTGGATGAGGAATTCGACACGATTCCGAGCTCTAAACCCCCCGACCTCATCCGTCAAAGATACGACCGCCTCCGACTGCTGGCGGCCCGGGTCCAGACAGTCCTGGGCGACTTCGCGACCCAAGGCGAGAGGGTCCAGGCCCTGGTGAGCTGGAGGGACCCGAGGGCGACGAAGCTGTTCATCGGAGTGTGCCTCGCCATCACGCTGATCCTGTACACGGTGCCGCCGAAGATGGTGGCGGTAGCCCTGGGATTCTACTACCTCCGACACCCAATGTTCCGGGATCCGATGCCACCGGCGAGCCTCAACTTTTTCCGGCGACTTCCGAGCCTCTCCGATCGGCTAATGTAG

>EucgrB03663

ATGCAGAAACCTCCTCAACCCCAAGATTTTGCTCTGAGAGAGACTTCACCGAACATTGGTGCCGGACAAGTCACACCTGCTGACAAGCTCGCCTGCACCTATGACCTTGTTGAGCAAATGCAGTACCTATATGTGCGTGTTGTCAAAGCTAAGGAATTACCGGGGAAGGACGTAACTGGTACTTGTGACCCCTATGTTGAGGTAAAACTCGGAAACTATAAGGGAGTCACCCGGCACTTCGAGAAGAAGTCGAACCCAGAGTGGAACCAGGTGTTTGCTTTCTCAAAAGAGAGGATTCAAGCATCGAGCTTGGAGGTGGTGGTGAAAGATAAGGACGTCGTGCTTGATGATTTCATGGGAAGGTCAATGTTCGACATCAATGAAATTCCTAAACGTGCTCCACCTGACAGCCCATTGGCTCCTCAGTGGTATAGATTGGAAGACCAAAAAGGGGAGAAGGTGAAAGGAGAACTAATGCTTGCCATTTGGATGGGAACACAAGCCGATGAGGCATTTCCGGATGCTTGGCACTCAGATGCAGCAGCTGTCGCAGGTGAAGCAGTTTCGCGCATTCGGTCTAAGGTTTATTTGTCCCCAAAACTCTGGTATGTAAGGGTCAATGTCATAGAGGCTCAGGATTTGGTACCTAGTGACAAAAGTAGGTATCCTGAAGTTTTTGTGAAGGCGATCCTCGGAAACCAGGCTTTAAGAACTAGAATATCTCAAAGCAAGTCTGTAAACCCAATGTGGAACGAAGATTTGATGTTCGTGGTTGCCGAACCATTTGAGGAACCCTTGATTCTTTCTGTAGAAGATAGACTGGCGGGAAACAAAGATGAAGTACTTGGGAGATGCATGATCCCGCTGCAAAACTTGCAGAGGAGGCTGGATCATAAGCCTGTTAACACAAGGTGGTATAATCTTGAGAAGCACATAGGAGAGGGAGAACAGAAGAAGGTGATTAAGTTTGCGAGCAGGATCCATTTGAGGGTATGTTTGGATGGTGGATATCATGTTTTGGATGAATCAACACACCACAGCAGCGATCTTAGGCCAACAGCAAAACAGTTGTGGAAGTCCAGCATTGGGATTTTTGAACTAGGGATTTTAAGTGCTCAGGGGCTCATCCCGGCGAAGTCAAGAGATGGAAGAGGGAGTACGGATGCTTATTGTGTGGCTAAATACGGGCAGAAATGGGTGAGGACACGAACAATTGTCGACAGTTTTAGTCCTAGGTGGAATGAGCAATACACTTGGGAGGTTTTCGATATATGCAGCGTGATCACCATAGGGGTTTTCGATAATGGCCATATCCATGGGGGAGGAGATAAAGGTGGAGGTGGAAAAGATTCAAGAATTGGCAAGGTAAGGATTCGACTGTCCACGCTCGAAGCTGATAGAATTTACACACACTCCTACCCCCTTCTGGTTCTACATCCTTCGGGGGTGAAGAAAATGGGCGAGGTTCAATTGGCCGTGAGGTTCACATGTTCTTCGTTAATAAATATGTTGCACAAGTACTCACATCCATTATTACCAAAAATGCACTATGTTCACCCTTTATCTGTCATTCAGCTGGATAGTTTGAGACACCAGGCAATGCAGATTGTGTCAATGAGGCTGAGCCGGGCTGAGCCACCACTGAGAAGAGAGGTGGTGGAGTTTATGCTCGATGTAGACTCACACATGTGGAATATGAGGAGGAGCAAGGCCAATTTCTTCAGAATCATGGGCGTCCTGAGAAGTTTGATTGTGGTTGGGAAGTGGTTCGATCAGATCTGCCATTGGAAGAACCCGCTCACTTCTATCTTAATCCACATTTTGTTTATCATCTTGATGCTTTACCCAGAATTGATACTTCCCACGATTTTTCTCTACCTATTCATCATTGGGATTTGGAATTACCGGTGGCGGCCACGACACCCTCCTCACATGGATATTCGACTGTCTCATGCGGATGCAGTTCATCCCGACGAGCTCGACGAAGAGTTCGATACGTTCCCCACATCCAAACCATCAGATATTGTTAGGATGAGGTATGATCGCCTCAGAAGCATAGCGGGAAGGGTTCAAACGGTGGTAGGTGACTTGGCAACTCAAGGCGAAAGGTTTCAGTCTCTTCTGAGCTGGAGAGACCCGAGAGCAACTACTATTTTCGTCACCTTCTGTTTAATTGCTGCGGTCGTTCTGTACGTCACCCCTTTCCAGGTTGTGGCGCTTCTTACTGGATTCTACGTGTTGAGGCATCCCAGATTCCGGCACAAGTTGCCTTCAGTCCCCCTCAATTTCTTCCGGAGGTTGCCTGCAAGATCAGACAGCATGCTGTGA

>EucgrD01859

ATGATGAGTAGCCTCAAACTAGGAGTAGATGTCGTCAGTGCTCACGATCTTTTGCCAAAAGATGGGCAAGGTTCATCCAATGCATTTGTGGAGCTCTCTTTTGACAGGCAGAAGTTTCGGACCACCATTAAAGAAAAGGATCTCAGCCCCGTTTGGAACGAGAGCTTCTACTTCAACATCTCCGACCCTTCCAATCTCCATTACCTCACTCTGGACGCTTGCGTCTACAACACCATCAAGGCCACCAACACCAGAAACTTCCTCGGGAAGGTTTGCCTTACCGGTACTTCCTTCGTTCCCTACTCTGATGCTGTTGTGTTTCACTATCCACTGGAAAAACGGGGCATTTTTTCACGTGTGAGGGGAGAGCTGGGCCTGAAAGTGTACATCACCAACGACCTGAACATAAAGTCTTCCAACCCACTGCCAGCAACCGACATCCCTCAAAAGGATCCGAGTTCAAATGTCCCTAACATGGTTTCTAACACGGGCCATGCTGAGAAAGGTGGGGTGAGACACACATTTCATCACCTCCCGAACACGAATCATCAACAGAAACAGCAAGCCTCTGAGCCAGAAATTCCTCACAGGGAGCATGTGCCTCAACATGATCACGGGCCAAAGTACGGCATCGACCATATGAAAGCAGAACCACATCCTCCCAAGCTGGTCCGGATGTACTCTGCAGCATCGTCCCAACCAGTTGAGTATGCCTTGAAAGAGACGAGCCCATACCTAGGAGGGGGAAGAGTTGTGGCAGGCCGCGTTATTCGTGGTGACAAGACAGCAAGTACTTATGATCTCGTTGAGCAGATGCATTTTCTATATGTGAGAGTCGTTAAGGCTCGTGAGCTTCCGGCCATGGACATTTCTGGTAGTATCGATCCGTATGTTGAGGTGAGAGTGGGGAACTATAAGGGAATTACTAGGCACTACGAGCAAAAGCAAAACCCGGAGTGGAACCAGGTGTTTGCCTTCTCAAGGGAGCGGATGCAAGCGTCCGTGCTAGATGTTGTCATCAAGGACAAGGATCTGGTCAAGGACGACTTTGTGGGCATTGTGCGGTTCGACGTTAATGAGGTCCCTCTTAGGGTCCCGCCAGATAGCCCCCTGGCACCGGAGTGGTACCGGCTTGAAAGCAAGAAGGGAGAGAAGATAAAGGGGGAGCTAATGTTAGCAGTTTGGATCGGCACCCAGGCTGACGAGGCGTTTCCCGAAGCTTGGCATTCTGATGCCGCAACACCTGTCAATGCTACGCCGATGGTCTCGGCCCTGATACGCTCGAAGGTCTATCATGCACCGCGGTTGTGGTATGTGAGGGTTAATATCGTGGAGGCCCAGGACTTAGTTCCGACTGAGAAAAATCGTTTTCCTGATGTTTACGTTAAGGCTCAGATAGGGAACCAGATAATGAAGACAAAGGCAGTTCAGGCTCGAGGCTTCAATTCCTTGTGGAACGAGGATCTTTTGTTCGTTGCTGCCGAACCTTTCGAAGACCATCTGATCCTTTCAGTTGAGGACCGTGTAGCCCCTGGCAAAGATGAGATCCTTGGGAGGGTCATCATCCCTTTGGGGTCGGTGGAGAAGCGTGCAGATGATCGGATCGTCCACACTCGTTGGTTCAATCTAGAGAGGCCTATAGCCGTGGATGTGGATCAGATAAAGAAAGAGAAATTTTCGAGCAAGATCCATCTCCGAGTTTGCCTAGACGGAGGATATCATGTTCTTGATGAGTCCACCCATTATAGCAGCGATCTACGGCCCACAGCTAAGCAGTTATGGAAGCCATCAATTGGTGTTTTGGAACTCGGAATTCTGAATGCCGTGGGCCTTCACCCGATGAAAACACGAGACGGCAGGGGCACGTCTGATACGTATTGCGTTGCCAAGTATGGTCACAAATGGGTCCGCACGCGTACCATTGTCGACAATCTGTGTCCGAAATATAATGAGCAGTATACTTGGGAGGTTTTCGATCCGGCCACAGTTCTAACGGTAGGCGTATTCGATAACTGCCAGCTCGGAGAAAAGGGTGCAAATGGCAACAAGGACCTGAAAATTGGGAAAGTGAGGATCCGCATTTCGACCCTGGAGACGGGTCGTGTCTACACGCACACGTACCCACTCCTAGTGCTCCATCCTACAGGAGTAAAGAAGATGGGAGAGTTGCATCTGGCAATACGGTTCTCGTGCACATCTTTAATGAACATGCTTTGCACTTATTCGCGTCCGCTTTTACCTAAGATGCATTATGTGAGGCCCTTCAATGTGATGCAGCTCGACATGCTCCGCCACCAAGCTGTCACCATAGTGGCGGCGAGACTTGGCAGGGCCGAGCCTCCACTTCGGAAGGAGGTGGTGGAGTACATGTCCGATGTGGACTCGCACCTCTGGAGTATGCGCAAGAGCAAAGCGAACTTTTTCCGTTTGATGTCAGTTTTCTCCGGATTATTTGCTATCGGAAAATGGTTTGGAGATATTTGCATGTGGAGAAATCCCATTACGACTGTGCTCGTGCACGTGCTCTTCGCAATGCTCGTCTGCTTCCCGGAGCTGATACTGCCCACTACATTTCTGTACATGTTTCTGATCGGAGTATGGAATTTCAGGTATCGCCCCAGGTACCCTCCCCACATGAACACCAAAATCTCGCAAGCTGAAGCAGTACATCCTGACGAGCTGGACGAAGAATTCGACACATTCCCGACCACGCGCAGCCCGGATCTGGTGAGGATGAGGTACGATCGGCTCAGAAGTGTGGCTGGTAGGATTCAGACCGTGGTCGGCGATGTGGCGACACAAGGAGAGCGGCTTCAGGCTTTACTGAGCTGGCGGGATCCACGTGCTACGGCCATCTTTGTCACTTTCTGCCTCGTCGCTGCGCTTGTTTTGTACGTAACCCCCTTCCAGGCGGTGGCAGGATTGGCCGGATTTTACATGATGAGGCATCCTAGGTTCCGTCACAGGCTACCTTCAGCTCCAATCAACTTCTTCAGACGCCTGCCAGCCAGAACTGATAGCATGCTGTAA

>EucgrD02276

ATGAGTAGTCTCAGGCTTGGCGTGGAGGTAGTGAGTGCGCATGATCTTGTTCCCAAAGATGGGCAAGATTCTTCCGGTGTTTATGTGGAGCTCCAATTTGAGGGTCAAAAATTTCGCACCTCTTTGAAAGAGAGAGATCTTAGTCCAGTCTGGAATGAAAACTTCTACTTCAACGTCACCAACCCTAGCAACCTCCGCGACCTTACCCTTGATGCCTATGTCTACAGCCACAATAAAGCCACCGACAAGAAGTCCTTCCTTGGAAAAGTTTCCCTTACTGGGTCATCATTCGTGCCACTCTCGGATGCTGTCGTCCTCCATTACCCCCTCGAAAAACAAAGAATTTGGTCACGGGTGAAGGGAGAGCTTGGCTTGAAGGTTTTCGTAACAGATGATTCTTACATGAGCTCCTCAAATCCACGGCATACAACACAGTCCGCCATGAATGTCGACCCACTTTCAGCTGAATCAATTCCAAATACCTTCTCCAATGAAAGAGCCGAGTCAAGGCGTACTTTCCGTCACCTTCCAAACTCAGACCGCCGAAAACAAAATGACAATACATTTGCAGCAGGTCCCCCGCCAAAACCAAACGTGGGGTCTTATGAGATGAGATCCGGACCACGGCCTCCAAATATAGTCAACACTTATGCTGGCTCAATGCCTATGCAACCAGGGGAGTATTCTGTGAAAGAAACAAGCCCCTATCTTGGCGGTGGCCGAATTGTTGGAGGGCGAGTAATACCTTCTGACAGGCCGAACAGTACCTATGATCTTGTTGAAAAGATGCAATACCTGTTTGTTCGAGTCGTAAAAGCCCGTGATCTTCCTGCTAAGGATGTAACAGGAAGCCTTGATCCGTATGTGGAAGTTAGAGTGGGGAACTATAAAGGAACTACAAAGTATTTTGAAAGGAAGCAAAACCCAGAGTGGAATGAGGTGTTTGCGTTTGCAAAGGACCAGTTACAGACATCTGTGCTCGAGGTTGTGGTCATGGACAAAGATCTTGTAATGGATGACTTTGTGGGTTTAGTTCGCTTGGATACTTACGAGATTCCCACTAGAGTTCCACCTGACAGCCCGTTAGCTCCTGAATGGTATAGGCTTGAAGATGAGAATGGGGAGAAGATAAGAAAAGGGGAACTGATGCTCGCTGTTTGGTATGGCACACAGGCAGATGAAGCTTTTCCTGAAGCTTGGCACTCAGATGCTTCTGCTGCAACTGATCCAACTCCAGGTATCTATGGCCATACTCGTGGCAAGGTCTACCACTCGCCTAAATTGTGGTATGTACGTGTTTTGATAATTGAGGCACAAGATCTGCTAGTGTCGGACAAGGGCCGCTTCCCTGATGCATATGTTAGGGTGCAGATAGGTAATCAGGTGTTCAAGACAAAACCAGTTCAGGCTCGGACTATGAATCCCGTGTGGAGAGAGGAAATGCTGTTTGTTGCTGCCGAGCCCTTTGAAGAGCCTTTGGTTCTTACAATTGAAGACCGTTTAGCTTCCACTAAGGAGGAAACTATTGGGAAAGTTATTGTACCACTGACCCAAGTTCACAGGCGTGCCGATGACAAACTTGTGCGCAGCCGCTGGTTCAACCTTGAAAAGTCAACATCGGCTGCCATGGATGCCGATGCAGGAAAGAAGGACAAGTTCTACACCAGATTGCATGTGCAAGTCTGCCTTGAAGGAGGGTATCATGTGCTCGATGAGTCCGCGTACTACAGCACTGACCTCAGACCGACCGCAAAGCCACTGTGGAAGAATTCCATAGGGGTCTTGGAACTTGGGATTCTCAATGCTGACGGGCTTCACCCAGTGAAGACGAGAGATGGTAAGGGTGCATCAGACACTTATTGTGTAGCAAAGTATGGCCAAAAGTGGGTGCGAACTAGGACCATAATCAACAGCCTGAGTCCCAAATACAATGAGCAGTACACATGGGAAGTTTATGATCCAGCAACTGTTCTAGTTGTGGGAGTTTTCGACAATAGTCAGATAAATGGCAGTAACAAAGATACTAAGATCGGGAAGGTTCGGATTCGGCTATCTACTCTCGAGACTGGTCGTGTGTATACACACTCCTATCCACTTCTAGCACTTCACCCGTCTGGGGTAAAAAAGATGGGTGAGTTACATTTGGCGATAAGATTTTCATGCACGTCACTAGTGAATATGATGTATATCTACTCTCGGCCACTTCTACCGAAGATGCACTACATTAGGCCGTTGACCCCAATGCAACAGGACATGTTGCGTCACCAAGCGGTCACCTTGGTGGCGGCTCGTCTCAGTCGGGCCGAGCCTCCCCTAAGGAGGGAAGTAGTTGAATACATGTCTGATGCACACTCCCATTTATGGAGCATGAGGCGGAGCAAGGCGAACTTTTTTAGGCTGATGTCAGTTTTCTCAGGATTACTTGCTTTTGGGAAATGGTTTGGGGAGGTGTGCAAATGGAAAAACCCTGTGACGACCGTCCTTGTGCATGTTCTCTTTGGGATGCTCGTGCTGTTTCCAGAGTTGATCTTGCCAACTGCTTTCTTGTACATGTTCATAATAGGTCTTTGGAATTACCGGTATCGGCCAAGACATCCCCCTCATATGAACACAAGAATATCTTATGCGGATGGTGTTCCTCCAGATGAGCTCGACGAAGAATTTGACACGTTTCCAACCAGTCGAAGCCCGGATGTAGTTCGATTCCGCTATGATCGTTTGAGGAGTGTCGCAGGAAAGGTTCAGTCTGTGGTAGGTGATATCGCAAGCCAAGGCGAGCGATTCCAAGCACTAATAAGCTGGCGAGACCCTCGCGCCACAGTCATCTTCTTGCTATTTTCTCTCGTGGCAGCAACCGTGTTGTATGCAACACCTTTCCAGGTTGTGGCTCTCATTTGCGGGACTTATGTCATGAGGCACCCTAGGTTACGGCACAAGGTGCCCTCTGCGCCTATCAATTTCTTCAAGCGACTGCCTGCTCACACGGACAGTATGTTGTAG

>EucgrF02999

ATGGCGAAGCTGGTGGTGGAAGTTCTCGACGCGAGTGATCTCGGGCCCCCAAAAGATGGGAATGGCTCGGCCAGTACGTACGTGGAAGTAGAATTCGATGAGCAGCTTCAGAGGACTCAGACCAAGCCGAGTGACTTTAACCCACAGTGGAATGAGAAGCTCCTCTTCAACGTGACCACCCCCGCGCAGTTCCCCAACGCCACCATTGAGGTCGTGGTGTACAATGACAAGACAGGCGGCGGCGGCGGCGGCGGCGGCGGCCACCACAAGCACTTCCTAGGCCGGGTCAGGATCTCCGGGGGCTCCGTCGCCTGGTCCGAGTCGCAGGCGATGGTCCAGAGGTACCCGCTCGACAAGCCGGGGCTCTTTTCGCACACCAGGGGCGACTTGGCACTCAGGATCTATGCGATTCAGGGCTTTTCTGAGCCGTTCGTGGCGAATGGCGCGGCTTGCGAAGATGCAGGGAGTCCCCTGCAGGAGGTGAACACCAACAATCTCGAGGGTGATCAGGATTCGGGGGACAAGCAGAAGAAGAAGAAGAAGAAGAAGAAGAAGCAGGAGGAGAAAGAATTGAGGACATTTTATTCCGTTGGGAATGCTGCTACTGCTACAGAGGGTCCGGCAAACCCTCCTCCTATGGCCCCAGGATATGCATTTCAGGCACCACCCAACGTGCAGATGAGGGCTGATTTTGCCAAAGCATCAGCACCTCCAGCAAATGTGATGCACATGCAGGTGCCGAGGCAGAAGCCGGAGTTCGGGCTGGTGGAGACCCCGCGTCCTCCGGTCGGAGCCTGGACGCGATTCAGAGGAGGGGATAAGATGGCGAGTACTTACGACCTGGTAGAGGAGATGCATTTTCTGTATGTGAGTGTGGTTAAGGCCAGGGGCCTTCCTGTCATGGATGTTACAGGGAGCCTTGACCCGTATGTGGAGGTGAAGCTAGGGAACTACAAAGGGGTGACCAGGCACTTGGAGAAGAACCAAAACCCAGTGTGGCATCAGACTTTCGCGTTCTCCAGGGAGCGGATGCAGTCGAATTTTCTGGAAATCACTGTCAAGGACAAGGACGTCGTGAAGGATGATTTTGCGGGTAAGTTCGTGTTCGACACCCATGAAGTGCCTCGTCGAGTGCCGCCCGATAGCCCGTTGGCGCCTCAGTGGTACAGACTGGAGACCAGGAAAGGAGAGAAGAGCCACGGAGAGATCATGCTCGCGGTTTGGATGGGGACGCAGGCGGATGAGTCGTTTCCTGAAGCGTGGCACTCCGATGCCCACAATGTGAGCCATGTAAACCTGTCCAACACTAGATCGAAGGTCTATTTCTCGCCGAACCTGTACTACCTCCGAGTCAACATCATCGCAGCTCAGGACCTCATCCCCTCCGACAGAGGCCGGGTGCCGGACACGTATGTGAAGGTGCAGCTCGGGAGTCAGCTCCGCAGCACTAGGCCTTCGCAGATGAAGACCAACAATCCGGTCTGGGACGATGAGCTAATGTTTGTCGTGTCCGAGCCTTTCGAGGATTATATTATCATCTCGGTGGAAGACAGGGTTGGACCTGGAAAGGACGAGATCTTGGGAAGGCTGATGATGCCGGTTAGAAGCGTTCCTCATAGGGTAGATGCTGTGAAGCTCCAGGACACTTGGTTCAACCTTCTCGCACCATCCAGGGCCGACGAGGGGGAGAAGAAGAAGGAAGTAAAGTTTTCGAGCAAGATTTGTCTCCGGTTTTGCCTGGAATTGGGGTATCATGTCCTCGACGAATCGACACATTTCAGCAGCAATCTTCAGCCATCATCGAAGCATCTGAGAAAGCCGTGTGTTGGATATCTTGAACTTGGGATCCGGAGCGCGAAGAATTTGCACCCCATGAAGGGAAGGGATGGTAGGACAACCGATGCCTATTGCGTGGCCAAGTACGGGAACAAATGGGCTAGAACCCGGACGCTCCTCGACACACTGGCTCCGAGGTGGCACGAGAGGTACACATGGGAAGTGTACGATCCCTGCACGGTGATTACCATCGGCGTCTTCGACAACTGGCACATCAATGGAAAGGATGAAGCGAGAGACCAGAGGATCGGAAAGGTAAGAATCCGGCTCTCTACTCTGGAGACAGACCGGATATACATCCATCGCTATCCCCTCCTGGTCCTCCAACCCTCCGGCTTACAGAAACGCGGAGAGCTGGAATTGGCGTTGCGATTCACCTGCACGGCTTGGTTCAACATGGTGACTCAGTATGGCAAGCCATTGCTTCCGAAAATGCATTACGTGCAACCAATACCTGTTAAGCACATCGATCTGCTCCGCCACCAGGCAATGCGGATCGTGGCAATGAGGCTGTCGCGAGCTGAGCCACCTCTTCGGGGGGAGGTCATCGAATACATGCTGGATGTGGACTACCACATGTTCAGCCTAAGGAGGAGCAAGGCCAATTTTTGCCGCATAATGTCGCTCCTTTCCGGGGTAACAGCAATTTGCCGATGGTACAGTCAGATATGCTACTGGAGCAATCCGATCACTACCTGTCTTGTCCACGTGCTTTTCACGATTCTGGTTTGCTACCCGGAGCTGATCTTGCCCACGATTTTCCTTTATCTTTTCGTGCTCGGGATATGGAACTATCGATTCAGGCCGAGGCATCCGCCCTGCATGGATCCTCGGCTTTCACAGGCAGAGTTTACTAGTCCAGACGAGCTGGACGAGGAATTCGATACTTTTCCATCATCGAAACCGACAGATATCATGAGGGCGAGGTATGATCGCCTGAGGAGCGTCGCAGGAAGAGTACAGTCAGTGGTAGGAGATTTAGCGACGCAAGGAGAAAGAGCTCAGGCTTTACTGAGCTGGCGGGATCCAAGAGCAACGGCTATAGTAATCATTTTCGCGCTGATCTTGGCAATCGTCCTATATGTTACGCCATTTCAGGTAGTGGCGATGCTGGCGGGACTGTATTCGCTAAGACATCCCCGTTTCAGGACCAAGATGCCCTCCGTGCCGGTGAATTTCTTCAAGAGATTGCCTGCCAAGTCCGATAAGCTTCTATGA

>EucgrF04116

ATGCCGAACTTGGTCGTGGAAGTCCTCGACGCGAGCGATCTGAAGCCCAAGGACGGGCAAGGCTCGGCGAGCCCGTACGTGGAGGTGGAGGTGGAGGAGGAGCGGCGTAAGACGCTGACCAAGCAGCGGGACCTCAACCCCCAGTGGAACGAGAAGCTGGTCTTCAGCATCGGCGACCCGAGGAGCCTTCACAGCAAGACGATCCACGTCGTGGTGTACAACGACACCAGAGGAGGAGGCCCCCACAAGCAGTTCCTCGGCCGGGTAAAGATCTCCGGCGCTTCCGTCCCTCTGTCCGAATCGGAGGCCTCGGTCCAGAGATACCCGCTCGACAAGCGCGGCCTGTTTTCGCACATCAGCGGCGATATCTCGCTTCGGATTTATGTCACCCACGACGACGGTTTTGGCATGTCGCCCCCGAACGTCGGTCCTGACGATGGTGTCGGTGCTGCGGAGACGCCTCTGAAAGATGCCGATTTTAGAGATGCCAAGCATGGTAGCGACCACGTCGGCGTCGACCACCATCATCATGCCGATTTCGATGAGAAGAAGAACAAGAAAAAGCATAAGGACCCGGAAGTGAGGACTTTCCGCTCCATCGGAGCTTCGGCTCCGACCGGCGCTCCCCCGGTGACGAGCTTCTTCTCGGGCCCCGGGAGCGGAGCCAATTTCATGAAGGAGAAGGCGCCGGCCGCCGAGACCAGGGCCGACTTCGCCCGCGCGGGCCCAGCTGCCGTAATGCAAATGCGGATGCCGGGGGGCCCGAACCCGGAATTCGCCTTGCAGGAGACTCGGCCGCCGGTGGCGGCGCACTTGCGGTATCGGGGAGGGGATAAGACCTCAAGCACTTATGATCTCGTGGAGCAGATGCATTATCTGTACGTGAACGTGGTCAAGGCCAGAGATCTTCCTATCATGGATGTTAACGGGAGCCTGGATCCCTATGTGGAAGTGAAGGTTGGGAACTACAAAGGAGTCACCAAACACCTGGAGAAGAACCAGAACCCAGTGTGGCGGCAGATATTCGCGTTCGCGAAGGAGCGGCTGCAATCGAATTTCCTGGAGGTGAACGTGAAGGACAAGGATACATTTTCCAAGGACGATTTCGTGGGGAGAGTCGTCCTGGACATTTCTGAGGTGCCGACTCGAGTTCCTCCGGACAGCCCTCTGGCTCCTCAGTGGTATAGGTTGGAGAACAAGAAAGGAGAGAAATTCAGGGGAGAGATCATGATCGCCGTGTGGATTGGGACGCAGGCCGACGAGTCATTCCCCGACGCCTGGCATTCCGATGCGCACAACATTAGCCACACTAACTTGCAAAATACTCGATCCAAGGTCTATTTCTCGCCGAAGCTCTATTACCTTCGAGTTCACGTCATCGAAGCTCAGGATCTTATTCCCACCGAGGGCGGTCGACTGGTCGACACATACGTCAAGGTTCAGCTAGGGAACCAGGGAAGGGTAACCAGGTCTTCCCAAGTGAAGACCGTGAATCCCGTGTGGCAAGATGAGCTAATGTTCGTGGCGTCTGAGCCGTTCGACGAATATATAATCGTGTCGGTCGAAGAGCGGGCAGGGCCTGGGAGGGATGAGACGTTAGGGAGGTTGATATTCCCGGTTAGGGACGTTCCGCACCGAAGTGAAACCGGCAAGATGATGGATGCTCGGTGGTTTAACCTCCACAAGCCATCTATCATCGAGGAAGGAGAGAAGAAGAAGGAACATAAGTTCTCTAGCAAGATTTGCATCCGGTTTTGCCTGGAATGGGGGTATCATGTTCTCGACGAGGCCACGCATTTCAGCAGCGATCTTCAACCGTCGGCGAAGGTGCTAAGGAAACAGAGCATTGGAATTCTCGAGCTCGGGATTCTGAGCGCTCGGAATTTGCTTCCCATGAAGGCGCACGAAGGCCGAGCTACCGATGCCTACTGTGTAGCCAAGTACGGGAACAAATGGGTCAGGACCAGGACCCTGCTGGACACCCTGCATTCTCGGTGGAACGAGCAGTACACCTGGGAAGTTTACGACTTGTGCACCGTGATCACGATCGGAGTTTTCGACAACTGCCACATCAATGGAAAAGACGACGCTAGAGATAAGCGAATCGGGAAAGTGCGAGTTCGCCTTTCCACTTTAGAAGCCGATCGAGTGTACACTCACTACTATCCCCTCCTCGTCCTCCATCCCTCCTCCGGCCTGAAGAAGCACGGCGAGCTCCATTTGGCGCTACGGTTTACTTGCACGGCGTGGGTTAACACGATGGCTCAATATGGCAGGCCTTTGCTTCCCAAAATGCACTACATCCAACCCATACCGGTTCGGTACATTGATTGGCTCCGGCACCAAGCCATGCAGATCGTGGCGGCCCGGCTGGCCCGGGCCGAGCCGCCGCTCAGGCCAGAAGTCGTGGAATACATGTTAGATGTGGATGCCCACATGTTCAGCCTGAGGAGGAGCAAAGCCAATTTCGGCCGGATCATGTCCCTGTTTTCGGGTGTCGCCGCCGTTTTCAAATGGTTCGACAACATTTGCCACTGGAGGAACCCTATCACCACCTGTCTCGTTCACATACTGTTCTTTATACTCGTTTGCTACCCGGAGTTGATCTTGCCGACGGTTTTCCTGTACCTTTTCGTGATTGGCCTTTGGAATTACCGACTCAGGACGAGGCTTCCACCTCATATGGATGCCCGGCTGTCGTACGCGGAGGGTGTCCATCCGGACGAGCTCGACGAGGAATTCGACACATTCCCCACAACCAGGCAATCGGATGTCGTGCGGCTGAGGTATGATCGGCTGCGGAGCGTAGCAGGCAGGGTCCAGACCGTGATCGGGGATATAGCCACACAAGGCGAGCGGATTCAGTCGATATTGAATTGGCGGGACCCCAGGGCCACCTCGATCTTCATCATATTCTCATTGATATGGGCAGTCATCATTTATGTCACTCCGCTCCAGATTATTATCGTGCTCATCGGGCTCTACCTGCTGCGGCATCCACGGTTCCGGGGCAAGCAGCCTCCCGTACCGGTCAATTTCTTCAAGAGATTGCCGGCCAAGTCGGATATGCTACTATAA

>EucgrJ01251

ATGCAGTCAGCTACTCTGCCAGCCTCAGCAACTGATCCAGATGATTATAAGGTCAAAGATGCCAATCCTGAGCTTGGCGAACGGTGGCTGAACAGTGGATCATATGGTGGCAGAGGGTGGATGAGCAGCGAGAGATTCACGAGCACATATGACCTTGTAGAACAGATGTTCTATTTATATGTTCGAGTTGTAAAAGCAAGAGATCTTCCTCCAAGCTCCATAACTGGGAGCTGTGACCCTTATGTGGAGGTCAAGTTGGGGAACTATAGAGGAAGAACGAGGCATTTTGAGAAGAAAATGAACCCAGAGTGGAACCAAGTTTTTGCTTTCTCTAAGGACAGAATCCAATCGTCAACACTCGAGGTTTTTGTGAAGGATAAGGAAATGGTGGGAAGAGATGATTATGTTGGCAGAGTGGTTTTCGACCTGAATGAAATTCCTACTAGGGTTCCTCCTAACAGCCCTTTGGCTCCACAGTGGTATAGGTTGGAGGACAGGAGGGGAGGAGGGAAACTGAGGGGAGAGATGATGCTGGCAGTATGGATGGGTACTCAAGCTGATGAAGCCTTCCCTGAGGCATGGCACTCTGATGCTTCCTCTGTCTATGGGGAAGGTGTACATAATGTTCGATCAAAGGTTTATGTGTCACCAAAGCTCTGGTATCTCAGAGTGAATGTCATTGAAGCTCAGGACGTGCTTCCCAATGACAGAAGCCGATTGCCAGATTTATTTGTCAAAGCTCAAGTTGGCAGTCAAGTACTAAGGACAAAGATATGTCCGACTCGCACAACCAACCCACTGTGGAATGAAGATCTGGTTTTCGTGGCAGCTGAGCCTTTCGAAGAGCTTCTGGTCATCACAGTTGAAGATCGAGTTCACCCTTCAAAAGAGGAAATGATGGGGAAAATAATTCTCCCGCTCGACACATTTGAGAAGCGGCTTGACCACAGGCCAGTTCATTCTCGATGGTTCAATCTTGAAAAGTATGGCTTTGGAGCCTTGGAAGCAGACAGGAGAAAGGAGCTGAAGTTTTCTAGCAGGATTCACCTGAGAGTCTGCCTCGAAGGTGGATACCATGTCTTGGATGAATCAACCTTGTACATAAGTGATCAGCGACCCACGTCAAGGCAGCTTTGGAAGCCGCCAGTGGGCATATTGGAGGTGGGTATTCTAAGTGCACAAGGGCTCCTCCCAATGAAGATGAAGGACGGTCGAGGGACCACAGATGCATATTGTGTAGCAAAGTATGGCCAGAAATGGGTTCGGACAAGGACGATTCTTGATACTATGAATCCTAAGTGGAGCGAACAATACACATGGGAGGTCTATGATCCTTGCACGGTGATGACCTTAGGGGTCTTCGACAATTGCCATTTGGGTGGAGGTGAGAAGTCAGCTACAGGCAGTGCAGCAAGAGATTCACAAATTGGCAAGGTACGAATTCGCCTCTCGACACTAGAAGCTCACCGTATCTACACACATTCTTACCCACTTTTGGTTTTGCAACCACAAGGGGTAAAGAAAATGGGTGAGTTACAACTAGCATTTCGGTTCACTACCCTTTCTCTCGCCAACATGATCTACGTCTACGGACACCCTTTGCTACCAAAAATGCATTACTTGCAACCTTTTACTGTCAATCAAGTAGACAGTCTCAGATACCAAGCAATGAATATTGTCGCGCTAAGGCTTGGGAGAGCTGAACCTCCTCTCAGAAAAGAAGTGGTGGAGTATATGCTAGATGTGGACTCACACTTATGGAGCATGAGGAGGAGTAAAGCCAACTTTTTTCGCATAATGTCCCTCCTTTCAGGAATGATTAATATGGGCAGGTGGTTTGGAGACGTCGGTAACTGGAAGAACCCCATGACATCAGTGTTGGTTCACATCTTGTTTCTGATACTAATATGGTACCCAGAGTTGATACTACCAACTCTCTTTTTCTACATGTTCCTCATCGGATTATGGAACTACAGGTTCCGTCCGAGGCACCCACCTCACATGGATACAAAGATTTCATGGGCAGAGGCTGCGTACCCAGATGAGTTGGATGAAGAATTTGATACTTTCCCTACTTCTAGGCCACACGATGTTGTCCGAATGAGGTATGACAGACTTAGGAGCGTTGCAGCCGTGGTGCTCTATGTCACTCCTTTCAAAGTAGTGGCCCTAGTGACGGGGCTGTATTACTTGCGGCATCCTCGTTTCCGGAGCAAACTGCCTTCAGTCCCAAGCAATTTTTTCAAGAGATTGCCTGCTCGCACAGAC

>EucgrK01506

ATGGCCACCGTTCGGAAGCTGGTCGTGGAGGTGGTGGAGGCCGTCAGCCTCTCCCCCAAGGACGGCCAGGGCACGTCCAGCCCTTACGTGGTCGCCAACTTCCACGGACAGCGCAAGCGCACCCGCACCGCCGTCAAGGATCTCTGCCCCCGGTGGAAAGAGGTCCTCGAGTTCGTCGTCCGGTCCCCCGACAGCTACGGCAACGCGCTCGAGATCGACGTCCTCCACGACAAGGCCCACGGCCCCACCACCCGCAACAACTTCCTCGGCCGCGTCCGCCTCAGCTCCTCCCAGTTCGTCTGGAAGGGCGAGGAGGCCCTCATCTACTACACTCTCCGCGGCAAGGGCTGGTTCAGCTGGGTCCAGGGCGATCTCGGTTTGAAGATCTATTACACGGTTGAGCTCCCGCCGCCGCCGCCGCCTCCGCCGCTGCCTCAGCCCCCGGCGGAGGAACCGAAGCCGGCGGAAGCCGACGCGCCACAGCCACCGCCGCCGGAGTCTGCCGAGCAGCCGCAGCCTCCACCAGATGGGGCGGCAGCTGCTGGTACCACGTCTGAAGCCACACCGCCTGCTGAGGGCGAAAAGCCCGGTGAAGAGGCACCTAACAAAGATGTGCCGCCGCCCGCGCCACCATCTACTGAAAATGCTCCTCCAGACGTCGAAACTACTGCCGAGGCCAAGCCGCCGGAAACAGCAGGGGCAAATCCAGAGGACTCGCAAGTGCCGCCACCGTCGCCGGCACCCTCACGAACTTCCAAGGAGACCGAAATGGATGCAGCATCCCAGTGGGTGCCTCCACCGTCGCCGGCACCCTCACGAACCTCCACGGAGACCGAAATGGATGCAACATCGCAGTGGGAGCCTCCACCGCCGCTGGCGGCGGCTTCTGTCTCGCGAACAGCCTCCCAAGTAAAATTCGCGGGGATGAATAGTTATCATCCTCCGCCGCCGAGGCAGCCGATGAGCAGGACCTACACAATGGATTCGCTGGACAGCATGGTCATGGAAAAGGAGCGTTCGTCGTTCGATCTGGTGGAGAAGATGCACTACCTCTTCGTCAGGGTGGTCAAAGCCCGGCATCTGCCGACCAACGGCAGTCCCGTCGTGAAGATCGTCGTCTCCGGCGCTCACGTCATGTCCAAGCCCGCGCGCAGGAGCGCGTCCACCGCTTTCTTCGAGTGGGACCAGACATTCGCTTTCGGCCGCGATGCCCCCGGCGAGTCCTCCGCCATCTTGGAAGTCTCCGTGTGGGACCCGCCTTCCGCCGACGTGGCGGGCCACAAGTTCTTCGGTGGCATCTGCTTCGATGCCGCAGAGATCCTCCTGCGGGACCCGCCGGACAGCCCGCTGGCCCCGCAGTGGTACCGGCTGGAAGGGGGCGGGGTCAACTATGCTGACCTGATGCTCGCCACGTGGATCGGCACCCAGGCCGACGAGTCCTTCCCCGACGCATGGAAGACCGACACCGCCGGCGACGTGAACGCGCGCGCCAAGGTGTACCAGTCGCCGAAGCTCTGGTACTTGAGAGCCACCGTGATCGAGGCTCAGGGCGTCTTGCCGTCGACCCCGGGGGCGGCGTTGAGGGATACCTCGTTTCAGATCAAAGCGCAGCTCGGATTCCAAATGCAGAAGACGAAGGTCTCCGTCGCCCACGACGGCTCCCCGTCATGGAACGAGGACTTGCTGTTTGTGGCGGCGGAGCCATTCGGCGATAACCTCATCTTCACCATAGAGAACCGGCTGCCCAGAGGGCCAGTCGAGATCGGCAGGGCGCCCGTCCCTCTCATGGCCATCGAGAGGCGTGTCGACGACCGTCAAGTGGCGTCGCAGTGGTTCACGTTCATGGACGGCGAGACGAACATGTTCAAGGGCAGGGTCCACCTGAGGCTGTGCTTCGACGGGGGATATCACGTGATGGACGAGGCGGCGCACGTCTGCAGCGACTACCGGCCCACGGCGAGGCAGCTTTGGAAGCCGCCGATCGGAATGGTGGAGCTCGGGATCATTGGCTGCAAGCACTTGCTACCGATGAAGACGGTGAACGGCAAGGGATCCACGGACGCGTATTGCGTGGCGAAGTACGGCCCCAAGTGGGTCCGGACACGGACCGTGTGCGACACGCTGGATCCCAAGTGGAACGAGCAGTACACGTGGAGGGTCTACGACCCGTGCACGGTCCTGACGATTGGAGTCTTCGATAGCCGGGGAGCGTCCGAGCCGGACGGTCCCCTAGAAGCCCCGCCTCCTGACTGTCGCATCGGCAAAGTACGGATACGTATATCTACGCTCCAGGCCAACAGGGTGTACCGCAATTTGTATCCGTTGCTCGTGCTGTCGAATGCCGGCTTGAAGAAAATGGGGGAAGTGGAGATCGCCATAAGGTTCGCGCGGACGGCTCCGACGCTCGATGTCCTGCACGTGTACTCGCAGCCTCTGCTGCCACTGATGCATCATGTCAAGCCCCTAGGAGTGGTCCAGCAGGAGTTGCTGAGGAGCGCCGCGGCGAGGATCATGGCCGCGCATTTGTCTCGATCCGAACCGCCGGTCAAGCGCGAGGTGGTGCTGTACATGCTCGACGCCGACTCGCAGGCGTTCAGCATGCGCAAGGTGCGCGCGAATTGGTTGAGGATCATCAACGTGCTTGCCGGGCTGATCGATGTGGTCCGCTGGATCGAGGACACGCGCGGGTGGAAGAACCCGACGGCCACGCCGCTCGTGCACGCGTTGCTGATCATGCTGGTCTGGTTCCCGGATCTGATCGTCCCCACGATAGCATTCTACGTGTTTGTGATCGGGGCGTGGAACTACCGGTTCCGGGGCCGGTACCCGGTCCCGCACTTCGACCCAAGGATGTCTCTGGCCGACACTGCAGACCGCGAGGAGCTCGACGAGGAGTTCGACCAGGTGCCGAGCAATAGTCCACCGGAAGTCGTGCGGGTGAGGTACGACAAGCTGCGCGCAATCGGGGCGCGTGTGCAGACGATGCTTGGGGATCTCGCCACGCAGGGGGAGAGGGTGCAGGCGCTGGTGACGTGGCGCGACCCGAGGGCCACGGGGATCTTCATCCTTCTGTGCTTCATGGTGGCGGTGATACTGTACTTGGTGCCGACGAAAATGGTGGCGATGGCGTCGGGGTTCTATTATTTCCGGCACCCGATATTCCGGGACCGGATGCCGGCACCGGCTTTGAACTTCTTCCGGAGGTTGCCTTCGCTTTCTGACCGAATGATGTAG

>EucgrK01536

ATGGCCGAACCGTGCGGCCGGAAGCTCGTCGTGGAGGTCTGCCACGCCAAGAACCTGATGCCCAAGGACGGCCAGGGCACCGCGAGCGCCTACGCCACCGTCGAGTTCGACGGCCAGAAGCAGCGGACGAAGACCAAGTTCCGGGACCTGAACCCGCAGTGGGACGAGCGGATGGAGTTCCCCGTCCACGACGCCGAGTCCATGCCCTCGCAGATGCTGGAGATCAACCTCTACAACGACAAGAAGAACGGCAAGCGCGGCACCTACCTGGGCAAGGTGAAGATCGCCGGGAGCGCCTTCGCCAAGTCCGGGTCGGAGGGGCTCCTCTACTACCCGCTGGAGAAGAGGAGCGTCTTTTCCCAGATCAGGGGCGAGCTCGGGCTCAAGGTCTTCTACGTCAACGAGGATCCCCCGGCCGCCGCGGCGGAGGCGGCGCCGGATCCGCCGGCGGAGAAGGCGACGGAGCCGGAGAAGCCGCCGGAGGAGGCGGCCCCGAAGCCGGAGGAGAAGGAGGACAAGGGCGAAGCGGAGAAGCCTAAGGAGGAGGAGAAGAAGGAGGAGGAGAAGCCGGCGGAGGCGCCGCCGGAGAACTCGAAGGCAGAGGAGGCTCCGGCGACAGCTCCGCCGGCGGCCACAGCGGAAGTGGAGAAACAGCCGATCGCGCAGTCGGAGAAGCCAGCGCAGCAGCCGAAGGAGAGGCCGGAGAACGGGAAAGCGGCCGTAAACGACCTCGAGCTCCGATCTCTATCGAGCGATCGGAACCGCCGGAGCACGTACGATCTGGTCGACAGCATGCCATTCCTCTACGTGCGCGTGGTCAAATCCAAGTGCGGAGACGCCGATTCGAAATCGCCGGCGTACGCCAAGCTCGTGATCGGCACGCACGCCGTGAAAACGAAGAGCCAGAGCGACGGCAAGGAATGGGATCAGGTCTTCGCGTTCGACAAAGAAGGATTGAACTCGACGCAGTTGGAGGTCTCCGTGTGGATCGAAGAGAAGAAGGAAGGCGATCAGACGGCCGAGAGCTGCCTGGGAACGGTGTCGTTCGATCTGCAGGAGGTGCCGAAGCGGGTGCCGCCGGACAGCCCGCTTGCTCCCCAGTGGTACACTCTCGAGTCGGAGAAGTCGCCGGCGGCGGCTGGAAATGACGTCATGCTCGCTGCGTGGTTCGGTACTCAGGCGGACGAGGCATTCCAGGAGGCCTGGCAGTCGGATTCCGGTGGGCTGATCCCGGAAACCCGGGCAAAGGTGTACCTGGCTCCGAAGCTGTGGTACCTGAGGCTAACGGTTATCCAGACCCAGGACCTGCAGTTATCCGAACCTAAGGTCAAGACTCCGGAGCTCTACGTGAAAGGTCAGCTCGGCGCGCAGCTGTTCAAGACGAACCGGACCACAGCCGGCTCACTGTCGTCCGGCTCGTCCAATCCGACGTGGAACGAGGATCTCGTGTTCGTCGCCGCCGAGCCGTTCGAGCCGTTCCTGGTGATCACGGTGGAGGACGCGACGCACGGCCGGCCCGTCGGCCAGGCCAAGGTGCACGTGCCCACCGTCGAGAGGCGGACGGACGATTGCACGGAGCCGAAGTCGAAGTGGTTCAACTTGGCCGGGGACGAGAGCAGGCCGTACACGGGGAGGATCCACGTGAGGGTGTGCCTGGAAGGCGGGTATCACGTGCTCGACGAGGCCGCTCACTTGACCAGCGACGTGCGCGCCTCCGCGAAGCAGCTGGCTAAGGCGCCGATCGGCTTGCTCGAAGTCGGCGTCCGAGGAGCGACGAACCTGTTGCCGGTGAAGACCAAAGACGGAACGCGCGGTACCACCGACGCTTACGTGGTGGCCAAGTACGGGATCAAGTGGGTCCGGACCCGAACCATCCTCGACCGGTTTAACCCGCGGTGGAACGAGCAATACACATGGGACGTGTACGATCCGTGCACGGTGCTCACCATCGGCGTCTTCGACAACGGGAGGTACAAGCGCGACGACGACGGTAAACCCGGGAAAGATTTGAGAATTGGGAAAATACGCGTGCGTCTATCGACGCTCGACGCGAACCGCGTCTACATCAACTCGTACTCGCTGCCCGTGCTGCTTCCCGGCGGAGCCAAGAAGATGGGAGAGATCGAGATCGCCATACGGTTCTCGTGCTCGTCGTGGCTCAGCTTGATCCAAGCCTACACGACGCCGATGCTCCCCAGGATGCACTACGTCCGCCCGCTAGGCCCTGCCCGGCAAGACATGCTCCGGCACACGGCAATGCGGCTCGTGACGGCCAGGCTGGCCCGGTCCGAGCCGCCGCTCGGCCAGGAGGTGGTACAGTACGTGCTGGACTCGGACACGCACGTGTGGAGCATGCGGCGGAGCAAGGCCAACTGGTTCCGGGCCGTCAGCTGCCTGTCCCGCGTGGCGATGCTGGGCAGGTGGCTCGACGGGATCCGGACATGGGCCCACCCGCCGACCACCGTGCTCATGCACGTGCTGCTCCTGGCCGTCGTCCTGTGCCCGCACCTCATCCTCCCCACCGTGTTCATGTACGCCTTCCTGATCCTGATCCTCCGGTTCCGGTACCGCCACCGGGCGGTCCCGAACAGCATGGACCCACGGCTCTCCCACGCGGACATGGTGGGCCCCGACGAGCTCGACGAGGAGTTCGACGGGTTCCCGACGACGCGGCCGTCCGACGTGGTCCGCATCCGGTATGACCGGCTGCGGGCCCTGGCGGGCCGGGCGCAGACCCTCCTGGGTGACGTGGCGGCCCAGGGGGAGCGGCTGGAGGCGCTCTTCAGCTGGCGGGACCCGCGGGCGACGGGGCTGTTCGTGGCCTTCTGCCTGGCGGCATCGCTGGGGTTCTACGTGGTGCCGTTCAAGGTGTTCCTCCTCGGGTCCGGGTTCTACTACATGCGGCATCCCAGGTTCCGGGGCGACATGCCGTCGGTCCCCGTCAACTTCTTCCGGCGGTTGCCGTCGCTCTCCGATCAGATGCTCTAG

>EucgrK03580

ATGAACGGCAACTCCGCCGGCGTCTCCGGCAAGGAGAAGCTGGTCGTGGAGGTCGTGGCGGCGCACAACCTCATGCCCAAGGACGGGGAGGGCTCCTCGTCGCCCTTCGTCGAGGTCGAGTTCGACAACCAGCGGCAGCGGACCCAGGTCAAGCACAAGGACCTCAACCCCGTCTGGAACGAGCGCCTCGTCTTCCTCCTGGCCGATCCCGCCGATCTCCCCTACCGGACCGTCGAGATCAACGTCTTCAACGAGCGGAGGTCCGGCAACAGCCGCAACTTCCTCGGCAAGGTCCGGATCTCCGGCTCCGGCGTCGCCAGGGAAGGCGAGGAGGTGGTCCAGCTCTTCACGCTCGACAAGAGGAGCCTCTTCAGCCACATCCGTGGCGAGATAAGCCTGAAGCTCTACGTGTCGACGCGGGAGGAGGTGAAGAGCCTGGCGGTCAACGGCGGCGGGTTGTCTTCTTCGTCTCTGGTCCAGAGGAAGAGCAAGAAGCTGCACCACCAGCCGAACGCGGCCTTGCTGGCCGTACAGCAGCAGCAGAAGCTGATGCTAGAACAGAGCAAGCCGGCACAGCAAGTGCCAAGCAAGCAGGCTCAGCGGGCGGAGCCGAGCCCTGGAGAGATGAAGCCGGTCGTGATAGCCGCGGCCCCGCAGCCCGTCACGGCGACGGGTGGCGGCGGCGGCGGAGGCGGAGTCCTGGGGGGATTTTTGGGCGGTGGGTCGAGCGAGTTCTTGCTCAAAGAGACGAAGCCAAGCCTAGGCGGTGGACCCACGAGCAAGGACAAGACGAGCTCGACCTACGACCTCGTCGAGCAAATGCAGTATCTTTACGTGAAGGTGGTGAAGGCTAAGGAGGTCTCGACGCCGTTCGGGGGTGGCGGCGAGCTGGTGGCGGAGGTCAAGCTCGGGAATTACAGGGGCATCACGAAGAGGGTCGGTTCGGGTTCTGGCTCGAACGGTGTGGAATGGGACCAAGTCTTCGCCTTTTCAAAGGATTGCATACAGTCCTCGGTCGTGGAGGTGTTCGTGAAAGAGAGGAATAAAGATGAGTTCTTGGGTCGTGTCTGGTTCGATCTTAATGAAGTCCCTAAGAGAGTGCCTCCGGATAGCTCGCTGGCGCCGCAGTGGTACCGGATGGAGGACCGGAAGGGCGAGAAGGCGAAGTCCGGGGAGGTGATGATCTCCATCTGGTTCGGGACGCAGGCTGACGAGGCCTTCGCCGAGGCATGGCACTCCAAGTCGGCGAACGTCAACTTTGACGGCCTGTGCTCGATCAAGTCCAAGGTTTATCTGTCGCCGAAGCTGTGGTACCTGCGCCTGAGCGTCATCGAGGCTCAGGACATCGTTCCATCGGAGCGTGGCCCGGCCATGGCCCGGTTCCCTGAGCTCTCCGTAAAAGCCCAGGTGGGCAACCAGGTCTTGAGGACGAGGGCTTCGCAGGCTGCACCGAACCGGAGCCTCTCGAGCCCGTTTTGGAATGAGGACATGGTGTTTGTCGTCGCGGAACCATTCGAGGATTATTTGATCGTCTCTGTTGAGGACCGGGTTGGGCCTGGCCGTGATGAAGTCGTCGGGCGGGTGCTTCTCCCGGCGACCTCAATCGAGAGGAGGACCGATGATAAGCCAGTGGTTTCTAGTGACCTTAGGCCGACTGCAAAACAGCTCTGGAAGCCTCACATTGGCGTGCTCGAAATGGGAATCCTCGGAGCCACGGGCCTCATGCCTATGAAAATCAAGGAAGGGAAGGGTGGATCGACCGATGCGTACTGCGTCGCTAAGTACGGTCAGAAGTGGGTTCGGACTCGGACCGTGAACATAGCGAGCAATGCTGGCCCCCGGGATGCACGGATTGGGAAAGTAAGGATTCGTCTCTCGACTCTTGAGTCTGACCGGGTCTACACGCACTCGTATCCGCTCTTGATGCTTCACACTTCAGGGGTCAAGAAAATGGGCGAGCTTCACTTGGCCGTGCGGTTCTCTTGCGCTAACATGGCCAACATGCTGCATATGTACGCAATCCCACTGCTCCCGAAGATGCATTACGTCCATCCGTTGTCTGTAAACCAATTGGACAGCCTGAGATACCAGGCCATGAACGTGGTGGCGTCCCGGCTGAGCCGGGCCGAGCCTCCCTTGGGAAGGGACGTGGTCGAGTATATGCTCGACCACGACTCTCACATGTGGAGCATGAGGAGGAGCAAGGCCAACTTCTTCAGGCTGATGAGCGTTCTCTCCGGTGCTGTGGCCATCAACCGTTGGGTCGAGGTGATCCGCAATTGGCACAAGCCGGTGTACTCGGCATTGTTCGTGGTGGTCTTCCTGTGGCTCGTTTGCTGCCCTGAGTTCATTATCCCCATCATGCTTCTATTCATGGCCTTAACTGGTCTATGGCGGTACCGGTCCCGGTCTCGCCACCCCCCGCACATGGACACACGCCTTTCTCACGCAGAGAGCGTTTTCCCTGACGAACTTGATGAGGAGTTCGACTCATTCCCGACGAGCCGCAGCGCGGAGGTAGTGAGGATGAGGTACGACAGGCTCAGGAGTGTGGCTGGGAGGATCCAGACTGTGGTCGGTGACATGGCCACTCAGGGCGAGCGGTTCCAGGCCCTCCTGAGCTGGCGCGACCCGAGGGCAACATTCTTGTTCATAATCTCCTGCCTATTTGCTTCAGTCGGGTTCTATGCGGTGCCTTTCAGGGTGGTGGTGGTTCTGTGGGGTTTGTATGCGCTGAGGCCACCGAGGTTTCGAAGCAAGCTGCCCAGCCGAGCGCTGAGCTTCTTCAGGAGATTGCCTACGAAGGCTGACGGCTTGTTGTAG

>Frves00519

ATGAAATCACAAGCTGGCCCTAATCCCCAAGAAGACTACAAACTGAAGGACACGAAACCCCAGCTCGGGGAAAGATGGCCACATGGAGGAGTGCGTGGTGGAGGAGGGTGGATTAGCAACGAAAGAGCTACGAGCACTTACGACCTTGTTGAGCAGATGTTCTATCTCTATGTTAGAGTTGTTAAAGCAAAAGATCTGCCAACAAACCCTGTAACAGGAAGTTGTGACCCTTACGTAGAAGTGAAGCTCGGAAATTACAAGGGCAAAACACAGCACTTTGAGAAGAAAACAAACCCCGAATGGAAGCAGGTGTTTGCATTCTCAAAAGATAAGATTCAATCTTCCATACTTGAAGTTTATGTCAGAGAAAAAGACATGGTAGCAAGAGATGACTATGTAGGGAAAGTGGTGTTTGACATGAATGAAGTGCCAACTAGAGTTCCACCGGATAGCCCTTTGGCGCCTCAGTGGTATAGATTAGAACACCGGAGAAGTGATACTAAGGTTAGAGGAGAGGTTATGCTTGCAGTATGGATGGGCACACAGGCTGATGAAGCCTTCCCAGAAGCTTGGCACTCAGATGCTGCTTCAGTCCATGGAGAAGGAGTCTTCAGCATTCGTTCCAAAGTTTACGTTTCACCAAAACTTTGGTATCTCAGAGTTAACGTAATTGAAGCTCAAGATGTAGAACCACATGACAAAAGCCAACCACCACAAGCTTTTGTGAAGGCTCATGTTGGGAATCAGACACTCAAGACACAGGTATGTCCAACCAAAACCCCTAATCCGATGTGGAATGAAGACTTGATCTTCGTAGCAGCAGAGCCTTTCGAAGAACTGCTTGTACTTACAGTTGAAAACAAAGTCAGCTCTGCAAAAGAAGTACAAGTGGGGAAAATAAGACTGCCACTGACCATGTTCGAAAGGCGCTTGGATCACAGGCCAGTTCATTCTCGCTGGTTCACCCTCGAAAGATTTGGTTTCGGTGCATTGGAGGGAGACAAAAGGCATGAGCTCAAGTTTTCAACCAGAGTCCATCTAAGAGTCTGCCTTGAAGGTGCTTACCATGTTCTAGATGAATCGACCTTGTACATAAGTGATGTGAGGCCTACTGCTAGGCAGCTATGGAAACAGCCAATTGGGATTCTTGAAGTGGGGATTTTAAGTGCTCAAGGGATTCTTCCAATGAAGACAAAGGATGGTAAAGCAACAACTGATGCCTACTGCGTAGCCAAGTATGGGCAGAAATGGGTGAGGACCAGAACAATCATTGAAAGCTTCAGTCCGAAATGGAATGAGCAATACACATGGGAGGTCTATGACCCTTGCACCGTGATCACACTAGGAGTCTTTGACAACTGCCACCTGGGTGGAAATGAGAAACCAACATCCGGCGGTGGAGCTAAAAATGACTCAAGAATTGGCAAGGTAAGAATCCGGCTATCAACTCTTGAAATGGATAGGATTTACACCAACTCTTATCCACTTCTTGTTCTTCACCCATCTGGGTTGAAAAAGATGGGGGAACTGCAACTGGCATTAAGGTTTACCTGTCTCTCATTAGCAAATATAATCTACCTCTATGGCCATCCCTTGCTTCCCAAAATGCATTACCTGCATCCATTCACTGTGAACCAGTTAGATAGTCTGAGATATCAGGCAATGAACATTGTAGCTGTGAGACTTGGCAGAGCTGAGCCACCACTAAGAAAAGAGGTGGTGGAGTACATGCTTGATGTAGATTCCCACATGTGGAGCATGAGAAGAAGCAAAGCCAACTTTTTCAGAATTGTTTCACTCTTCTCTGGCCTCATCTCCATGAGCAGGTGGTTTGGCGAAGTTCGTCACTGGAAAAACCCAATCACTACAGTTCTAGTTCATGTTCTGTTTTTCCTACTGATCTGCTACCCGGAATTGATCCTCCCTACGGTCTTCCTTTACATGTTTCTGATCGGAATATGGAATTTCCGTTTCCGGCCAAGAGTGCCTCCTAGTATGGACACTAAACTTTCATGGGCAGAAGCTGTTCACCCAGATGAAATGGATGAAGAGTTTGACACCTTTCCTACATCGAAGGCGCAAGATGTGGTGCGAATGAGGTATGACAGACTACGAAGTGTAGCGGGAAGAATTCAAACGGTGGTTGGAGACATAGCAACTCAGGGGGAGAGATTCCAGGCAGTGCTCAGTTGGAGAGACCCAAGAGCAAGCAGCTTGTTCGTATTTTTGTGCCTTATTATTGCTGTAGTGCTCTATGTGACACCCTTCAAGATGCTTGCTCTGATCTCAGGCATAGTTTGGCTACGCCACCCTAGATTCCGAAGCAAGCTACCATCAGTGATGAGCAATTTCTTCAGAAGATTGCCATCTCGTGCTGACAGCATGCTCTGA

>Frves07439

ATGGAAAGGAGCAACCTCAAGTTGAAAGTGGAGGTTGTGAGCGCCAGGAACCTTTTGCCGAAAGATGGACAAGGCTCAGCCGGTGCTTTTGCGGAGCTCCAGTTTGATCATCAAAACTTCCAGACCACCACAAAAGAAAATGGTGTCGATCCTGCTTGGAATGAAAGCTTCTACTTCAACATCTCTGATCGCGAACACTTACCTAGCCTCACTCTTGAAGCAACTGTTTACCACCACAATGAACCAAACTCCAAATCTTTCCTGGGAAAGGTTTGTCTTCCCGGGACTTCATTTGTCTCAGACCCTGATGCTGTTGTTTTGCGGTACCACCCTCTGCGAAAAAGAAGCATTTTAAGCTCGCGTGTGAGAGGAGAGCTTGGTTTAAAAGCTTTGGTTACTTATGATCATGGTCAACCCCTTCCGACCCCAAATCAACCACAGCAGCAGCAGAATGTACCAAGGGCTCCTATAAATGTTCCACATAACTCAGGTACTTCATCATCTAAATCATATGATTGTTTGCTTAAAGAAACTAGTCCTGTACTGGGAGGAGGGGAAATTGTTCGAGGGCGATTCATCGCAGAGAAACAGGTCAGCCCGTATAACCTTGTTGAAACGATGCATTACCTGTTTGTACGAATTGTGAAGGTGCGTGACCTACATTTGAAAAATATAACAGGAACCTCCTATGTTTCACTAAAAATTGGGTACTACAAAGGAAATACAAAGCCTTTATTAGAGCACTCGCACAATCCAGAATGGAATCAAGTATTTGCTATCAAAAGGGAACATATGCAGTCTCCTGTGATGGATGTTCTGGTGAAACATTACAGTCCTGAAAACAACAATGAAGGTGATCAATTTGAAGGGGCATTACAAATAAATATCCATGAAGTTCATCCTCGAGTTTCACCTAATAGTCCATTGGCTCCAAAATGGTATGCGATTGCAGACAAGAATGGGGACAAAAACGGGGATTTGATGCTTGCTGTATGGAAAGGCACACAAGCTGATGAGGCTTTTCCAGATGCCTGGCATTCTGATGCACCTCTTCCTCCCAACGTTTCTTCAGTTACTTATGAACAGATCCGATCAAAAGTGTACCACACACCAAGGTTATGGTACATAAGAGTAAATGTGACTGAAGCACAATTGTTTGAATACGGCATGTCCCGCGTCCCCAATGCATATGCACAGCTACAGATTGGTAATCAGTGTTTAAAGACAAAAGCAGCGGACTCTGAAGGACCGAACTCAATCTGCTGGAATGAGGAAATGGTATTTGTTGCTGCTGAACCATTTGATGATGAGCATCTGATCGTTTCAGTTGAATATGCCGTAGGTCCAGAAGAATACAAGAGCTTTGGAAGTGTTTCCATACCATTGAACTCTGTTGAGAGGTATGAAGATGATCGAACTATATGCGGTAAGTGGTTCAACCTCGAAAAGTCCATATTCGCTGATGTGATGGGAAATGACGCAGAAATCTCTTCCAGTAAAATCCATTTGTGTGTATGTCTTGATGGAGGGTGCAACGTGCTTAATGGGCCAACTAAGCAGCTCGGGAGTCCCATTGGTGTCTTAGAGCTTGGAGTTATAGCTGCTGGAAAGCTATTGCCAATGAAAAGTAGGAAGGGTAGGGGTACATCAGACCCCTATTGTGTAGCAAAGTATGGAGATATATGGTTTCGTACTCGAACCATAGTAGACAGCTTGAACCCGAAATTCAATGAGCCGCACAATTGGGATGTTTATGATCCTGCCACTGTTCTCACAGTGGCTATTTTTGACAACCAGATTGATGGCGACTCAGATGACAACAAACATGTGGAAATCGGCAAGGTCCAAATTCCAGTCTCTACCCTTGAGTCTGGCCGAATCTACACGCTGTTATACCCACTAATATCCCTTAGTTCTTCTGGTCTCAATAAGAAGGGTGAATTACATGTTGCAGTAAGATTTTCATACACATCATTCATTAACATGATGTTCACATACTCTCAACCCCCTTGGCATGTCAACGTATGTGAGCAAGCTGTCTCGGCAGTGGCAGCTCATTTTGGTCAGGCAGAAGCACCTCTTCAGAAGGAAGTAGTTCAATGCATGTACTCTGATGTAAACTCCCATGTTTGGAGCTTGAGGCGTAGCAAAGCAAACTTTTTCAGAGTGATGTCAGTTTTATCGGCATTTCTTGCTGTGGGAAAATGGTTTGAAGAAGTATGCATGTGGAAGAACCCCATTACAACAGCACTAGTACATGTTCTGTTTGTGATGCTGGTATGCACTCCAGAACTGATTTTTCCTACTGTTTTCCTGTACATGTTTGCTATAGGGGCTTGGAATTTTCGGTATCGCCCAACGTGCCCTCCTCACACGGACATAAAGTTCTCACATGCAGATTCTGTGCACCCTGACGAGCTTGATGAGGAGTTTGACACTTTTCCAACATCACGGGAGCGAACGCTCACGATGATGAGGTATGATCGCCTGAGGAGTATTGCTGGAAGGATTCAAACCATGCTGGGTGACATTGTAATCCATGGAGAGCGGATTCAGGAACTACTGAGTTGGCGAGATCCTCGTGTCACTACTTTATGTATAACATTGTGTCTTGTGTGTGCTATCATATCGTATGTGACACCTTTCAAGGCAATTGTTATTCTTGTTGGGTTTTACCTTATGAGGCACCCTTGGTTCAGGAGTAAGACACCACCGATCCTGTTGAACGTCCTCGAAAGGCTGCCGGCTAAAACGGATTGCATGTTGACCAGTAGCCTAGAGAGTTGA

>Frves08371

ATGACAGTGAGCAGCACCACCGGCAAAGAACGCCTGGTGGTGGAGGTAGTGGCGGCACACAACCTCATGCCCAAAGACGGCGAAGGTTCGTCGTCCCCATTCGTCGAAATCGAGTTCGAAAACCAGAGGCTCCGAACCCAGGTGAAGTACAAGGACCTCAACCCGGTCTGGAACGAGAAGCTAGTCTTTCATGTCAAGGACGTGGCTGATCTTCCCTACAGAGCCATAGAGGTCAACATTTTCAACGAGAGGAGGTCCGGCAACAGCAGAAATTTTCTGGGAAAGGTCAGAGTTTCGGGGTCTAATATTGCTAAAGAAGGAGAGGAGATTCCTCAGCTTTATACACTCGACAAAAGAAGCCTCTTTTCTCATATCAGAGGCGAGATTAGCTTGAAGCTTTACCTTTCGACCAGAGAAGAGGTCAAGGAAACCAGGGCTAATGGAATACTGGGTTCCTCTGTTTCCAACTCTGCTTCGTCTTCTGGGTTTTCGAAGAAAAGCAAGAAACTTCAGGGCCAAAGCTCGGTTATGGCTTCACAGAACCAGCTGATACAAGAAGTAAAGCCGACGCAGCAAGGTCAGAGCAACAATAATCACTCAAAGACTGTGGAGAACAACCAAGGAGTGATGATGAAGCCGATTCTGATCAATACAGGCCCTGTTTCTGCAATTTCCGGCGGCGGCGGAGTGACCGGAGGAGGAGGAGGAGGCGGCGGAGGGGTTGTGTACGGAAATGGGATGACGGAGTTTTCGCTGAAGGAGACGAGGCCTCAGCTCGGCGGTGAGTCTTTGAAAAAGGATAAAACTAGCTCTACTTATGACCTTGTTGAGCAAATGCAGTATTTGTATGTGAAAGTTGTTAAAGCTAGAGACTTTTCTGTTTTCGGAGGAGGGGAGGTTGTGGCTGAAGTGAAGTTGGGAAATTACAGAGGGATAACAAAGAGGGTGAGTTTGAACAATGTGGAGTGGGGTCAGGTGTTTGCTTTTTCTAAAGATTGCATACAGTCTTCAATGGTGGAGGTTTTTGTGAAGGAGGGGAACAAAGATGACTTCTTGGGGAGGGTCTGGTTTGATTTGAATGAGGTTCCGAAGAGGGTTCCGCCGGATAGTCAGTTGGCGCCGCAGTGGTATAGAATGGAGGATAAGAAGGGAGACAAGTCGAAATCAGGGGAGGTGATGATCTCCATTTGGTTTGGGACTCAGGCTGATGAGGCATTTGCTGAAGCTTGGCATTCGAAGGCTGCAAATGTGAATTTTGATGGGCTTGGTTCGATTAAGTCCAAGGTTTACTTGACACCAAGGCTTTGGTACCTGAGGGTTAAAGTGATTGAAGCTCAAGACATTGTTCCTGGGGAGAAAGGGTCTGCAATGATGAGGTTTCCGGAGCTTTCTGTGAAAATTCAGGTGGGGAATCAGGTCTTGAGGACTAGAATTGCGCAGCCTAGCAGTGTGAGGAGCCTCTCAAATCCTTTATGGAATGAGGAGATGATGTTTGTGGTGGCTGAGCCTATTGAGGACTACTTACTGATCTGTGTTGAGGATAGGGTTGGGCCAGGGCGAGATGAGGTGGCTGGGAGAGTGGTTATTCCGGTGGCAGCAATGGAAAGGCGAACGGATGACAAGCCTGTTGTTTCGAGGTGGTTCAACCTTGATAACAGCAGCCATTTCACCAATGCAGCTGGAGAGTCCAAAGTGATGACAAGGTTTGGGTCTAGAATTCATTTGAGGGTTTCACTTGATGGAGGTTATCACGTACTTGATGAGGCCACAATGTATAGCAGTGATCTGAAGCCGACTGATAAGAGGCTTTGGAAGCCCCACATTGGTGTGCTTGAAATGGGTATTTTGGGAGCCACTGGGCTTATGCCAATGAAGATCAAAGAAGGGAAAGGAGGGTCTAGTGATGCTTATTGCGTTGCGAAGTATGGACAGAAATGGGTTCGAACTCGAACTGTGGTTGATAGCTTGTCGCCTAAGTGGAACGAGCAGTACACTTGGGAAGTGTTTGATCCTTGCACGGTTGTTACCATTGGGGTGTTTGATAATTCTCGGATTGATAAGAACACGGCCAACAATGCTGGAGTTCGTGATTCTCGGATTGGGAAGGTTAGGATTCGATTGTCCACACTCGAGTCTGATCGAGTCTACACTCACTCCTATCCTCTCTTGATGCTGCACCCTTCCGGTGTCAAGAAAATGGGTGAGCTTCATCTGGCGGTCAGGTTTTCTTGTGCCAATATGGGTAACATGTTGAACATGTACACTATGCCACTGCTTCCCAAGATGCATTTTGTGCAGCCTTTGACTGTGAATCAACTGGAGACTCTGAGGCACCAGGCTATGAATGTAGTGGCATCGAGGCTTAGCAGGACAGAGCCACCGTTGGGGAGAGAAGTGGTGGAGTACATGCTTGACCATGACTCACATATGTGGAGCATGAGAAGGAGCAAAGCCAACTTCTTCAGGCTAGTGAATGTTCTATCAGGGCCTGTTGCTTTTGGAAGATTTGTGGAGCTGATGCGGAGTTGGCAGAAACCAATCTGCTCTGCCTTGTTTGTTGCAACTTTCCTCTTACTGGTTGCATTTCCGGAGCTCATAGTCCCAATGATCTTGCTGCATATGGCATTTGTTGGAATGTGGCGGTTTAAGTCCCGTCCTCGTCACCCATGTTTCATGGACACTAATCTTTCCCATGCTGAGAGTGTCTATGGTGATGAGCTAGATGAGGAGTTTGATTCGTTCCCAACGAGTCGGAGTGCAGAGGTTGTAAGGATGAGGTATGACCGGCTTAGGAGTGTGGCTGGGAGGATTCAAACTGTTGTTGGTGATGTGGCTACACAAGGTGAGAGGTTCCAAGCATTGCTAAGCTGGAGAGACCCAAGGGCAACGTTCTTGTTTGTGATCTTCTGCTTGATTGCTGCTGTAGTGTTCTATGCTGTGCCGATTAGAATGGTTGTGGTTTTGGTGGGATTGTATGTGCTCAGGCCGCCAAGATTCAGGAGCAAGCTGCCTTCTCCACCTTTGAGCTTCTTCAGGAGGCTGCCAACCAGGGCTGATAGCTTGTTGTAG

>Frves12953

ATGGCAGAAACCTCTGGGCGTAAGCTGATCGTTGAAGTCTGCAACGCCAAGAATTTAATGCCGAAAGATGGTCAAGGAACGGCGAGTGCTTATGCCATGGTGGACTTCGACGGGCAGAGACGGCGAACCAAGACCAAACAGAGAGATCTCAACCCGGAATGGGACGAGAAGCTCGAGTTTCTAGTCCATGACATTGAGTCCATGGGCTCCCAGATACTGGAAGTCAATATCTACAACGACAAGAAGAACAGTGGGAAACGAAGCACTTTTCTTGGCAAAGTCAAGATCCCGGGCTCTACCTTTGTGAAAGCAGAGCCGGAGAACACACTCGTTTACTTCCCCTTGGAGAAAAGGAGCGTGTTTTCTCAGATCAAAGGCGAGTTGGGATTGAAGATTTACTATATCGATGAAGACCCACCAGCTGCTGCGGCGGCGGATGACAAGAAACCGGCAGCGGAGGAGAAGCCGCCGGAAAAGCCAAAGGAGGAGGAAAAGAAGCAAGAAGAGGAGAAGCCGAAAGAGGAGATCAAACCAGCAGAAGAAGACAAACCGAAGGAGGAGGCTAATAAACCAGCAGAGGACAAGGCCACTGTAGCACCGGAATCAAAAACAGAGGAAGCAGCTTCTTCAGCGGTTGCTGCTCCGCCGCCAGAGGTTGAGAACCCGCCGATTGCTTACTCTGAGAAGCCAAATCATCAAGACAAGGTTGTAGAGAGGTCGACGGATGTGAGGATTAACGAGATGGAGCTTCAACCATTGGCCCGGGATAGAAATCGGAGCGCGTACGATCTCGTAGTCAGGATGCCGTTTCTTTTCGTGAAAGTTGTCAAAGCCAAAAGAGCAGACGCTACTACAAATCCATCGGCATCTCTTTACGCCAAGCTTGTAATTGGTACGCATACCATCAAAACCAAGACACAAAGCAGCAACAAAGATTGGGACCAAGTCTTTGCTTTCGACAAAGAGGGTCTCAATTCCACCTCTTTAGAAGTGTCTGTGTGGGCCGAAGAGGAGATCAAGAAGGAAGGAGAAGAAGCTCCCACTCTTACAGAGACTAGTCTCGGAATGGTGTCTTTCGATTTGCAGGAGGTGCCCAAGCGAGTTCCACCGGACAGTCCTCTAGCTCCACAGTGGTACACTCTCGACTCTGAAAAGTCTCCGGGAAATGACGTCATGCTCACTGTCTGGATCGGCACTCAGGCCGACGAAGCATTCCAAGAGGCTTGGCAGTCGGATTCCGGCGGGTTGATACCGGAGACCCGAGCAAAGGTTTATTTGTCTCCGAAGCTCTGGTACTTGAGAGTAACGGTCATCCAAACCCAAGATCTCCAGCTAGGCTCGGGATCCGAGGCTAACAAGAAGGTTCGGAATCCCGAACTTTCCGTGAAGGCTCATTTCGGGGCCCAGCTTTTCAAAACGAGTAGGACCTCCGTGGGGTCCACCTTGTCGAGCTCATCCAACCCCACCTGGAACGAAGACTTGGTTTTTGTAGCAGCCGAGCCGTTTGAGCCGTACATGACTGTGACCGTAGAAGATGTAAGCAACGGTCAATCTGTGGGGCACACCAAGCTTCACGTGCCAAGCGTTGAGAAGAGAACAGACGACCGTGCGGAGCCCAAGTCCAGATGGTTCAACTTGATTGGTGATGAGGCGCGTCCTTACGCCGGTAGGATACACCTGCGGGTTTGTCTAGAAGGAGGCTATCACGTGCTGGACGAGGCTGCTCACGTGACGAGTGACGTCAGAGCTGCGGCAAAGCAGCTGGCGAAACCTGCCATCGGCTTACTTGAAGTCGGAATTCGCGGGGCCACCAATCTGCTTCCGGTCAAGGTCAAAAATGGGATGCGTGGGTCAACTGATACTTACGTGGTTGCCAAATATGGGCCGAAATGGGTCCGAACCCGGACGATTCTGGACCGGTTCAACCCGCGGTGGAACGAGCAGTACACTTGGGATGTGTACGATCCATGCACTGTTCTCACTATCGGCGTCTTCGATAATGGAAGGTACAGGCAACCCGAACCCGAGAAAGATATTCGGATCGGAAAGATACGAGTACGGCTATCCACTCTTGATATGAATCGTGTTTACATGAACTCGTATTCTCTTACTGTGTTGCTTCCCGGTGGGGCTAGGAAAATGGGGGAGATAGAGATAGCCGTTAGGTTTTCATGCTCGTCGTGGCTGAGTGTGGTCCAAGCCTACAGCACCCCAATGCTTCCGAGAATGCATTACGTGAAACCAATGGGCCCGGCCCAACAAGACATTCTACGACATACGGCTATGAAGATTGTGACGGCTAGGCTGGGGAGGTCGGAGCCACCGCTGGGTCAGGAAGTGGTTCAGTTCATGCTCGACTCGGATACACACGTGTGGAGCATGAGGAGAAGTAAGGCCAACTGGTTTCGGGTGGTGGCGTGTTTGTCACGTGTGGCGACACTCGCGCGGTGGATGGATGGGATTCGCACGTGGCGGCATACGCCCACGACGGTTTTGATGCACGCGCTGCTGGTGGCTGTGGTGCTATGCCCGCATTTGATACTTCCGACCGTATTCATGTACGTCTTCCTCATCTTATTGGTGAGATTGCGGTATCGTCATAGAGTGCCGTCAAATATGGACCCTCGGATCTCTTATGTTGACGCCGTGAGCCCTGATGAGTTGGACGAAGAGCTTGATGGGTTCCCCTCCACTCGACCCGCAGACACGATCCGAATACGATACGATCGACTGCGGGCTTTAGGTGGGAGGGCCCAAACTTTGTTGGGCGACGTAGCGGCCCAAGGAGAGCGTCTGGAGGCCTTGTTTTATTGGAGGGATCCCAGGGCAACCGGAATCTTTGTGGTGTTCTGTTTCTTGGCGTCTTTGGTGTTTTACGTGGTGCCGTTCAAGGCTTTTGTGTTGGGGTCAGGGTTCTATTACTTGCGCCACCCTAGGTTCCGTCACGATATGCCATCACTTTCCACCAACTTTTTTCGGCGACTTCCATCCTCGTCTGACCAGATCATGTAG

>Frves18501

ATGACTAGTCATGAAGACTTCTCCGTCAAGGAAACAAGGCCGAACATTAGTAGCCGGAGGATCTCCACCGGTCCAGTGAGTAGTTTTGATCTGGTGGAGCACATGCAGTATCTATATGTGAGGGTTGTGAAAGCCAGAGAGTTGCCACTTCCCTGTGACCCTTATGTTGAACTGAAGCTTGGAAACTACACAGGAACCACAATGCCCTTCCAGAAGACTCCAAACCCGGTATGGAACCAAGTGTTTGCTTTCTCCAAAGACAGAATTCAAGATATAACTATGGAGATTTTGGTGAAGGACAGGGCTGTTGTTGTGGACGGTCAAGGTCATCCGACCATTGGCAAGTTCTCTTTCATTGTTCCCGAGGCTCCCATGAGAATTCCCCCGGATAGTGCATTGGCACCGGAATGGTATAGGCTAGAAGGTCTAAATGGGGTTAAGATTCGAGGAGAGTTGATGCTGTCTTTTTGGTTTGGAACACAGGCAGATGAGGCTTTTTCAGAAGCTTGGCACGCTGATGTGGCTGCAGTCAGTGGTGATTTTGTTTCCACTACTCGTTCCAAAGTTTACCTTTCTCCTAGGCTTTGGTATCTTAGAGTGAATGTGATTGAAGCTCAGGATCTGATACTTAAAATAAACAATAACAAGGTAGAGGCATCAGATTTGTTGGTGAAAGCAGGTCTTGGGAACTTGTTCATGAGAAGTAGAGTTTCTAAAAATAAGAGTGTAAACCCGGAATGGAATGAAGACCTAATGTTTGTTGCAGCAGAACCATTTGATGATCCTTTGGTTGTGAGTGTGGTGGAACTGGTGAACAGAAAGGAGGAACATTGTATAGGGATGTGTGTGATTCCTCTAAGCGATGTGGATAGGAGGATAGGTGCTGCCCCTCCTGCGAATAAGTGGTATAATCTTTCCATGATGGTTGAGGGTCAGCAGAAAGATGTCAAGTTTGCTAGTAAGCTCCACATGAGGATTAGTTTGGATGGGGGATACCATGTTCTTGATGAGCCTACTAATAATATCAGTGATCTTCTCCCGTCATCAAAGCTACTATGGAGACCTCCAATTGGGGTTTTGGAGTTGGGAATCTTGAATGCTACTGGACTATCACCTATAATGAACAATAGGCCTTCGGATCAGGTCTGGGCATATTGTGTGGCAAAATATGGGACAAAGTGGGTAAGAACAAGGACAATTGTTGATAGTTTGGAGCCGAAATGGAATGAACAATATACTTGGGAAGTCTATGATCCATGTACAGTCATTACCATTGGAGTTTTTGACAATGGATGTGGGAACAATAAGCTGGACTTGTGTATGGGGAAGGTAAGGATTCGGCTATCTACTCTCGAAACTGATAGGGTATACACAAATTCTTATCCCCTCGTGGTTCTAACACCTTTTGGTTTAAAGAAGATCGGGGAAATCCAATTGGCTGTGAGATTTTCTTGTTCATCTTTGCTTAACTTGCTCAACCAATATGCACAGCCTCTGCTACCCAAATTGCATTACATTCTCCCACTATCAATATATCAGCTTGCTAGCTTAAGGCACCAAGCGGCTTATATCATCGCAACAAGGTTAAGTCGGGCCGAGCCACCATTAAGGAAAGAGGTTGTGGATTACATGCTGGATGCCAATGCACATCTATGGAGCATTAGAAGAAGCAGAGCCAATTTCAACAGGATCATCAAACTTTTTGATGGTATTGTTGCTCTTTACAAATGCTTTGAGAAGATCCGCAAATGGACTAATCCCATTGTCACGGGGATTGTCCACATCATATTCTTGGTTCTGCTTTTCTTTCCTGGTGTGATACTTCCCACTATGTTTTTCTACTTCTGTGGCCTAGGGATCTGGCGCTTCCGGAGAAGGCCTAGACAGATTGCATACATAGATACCGAATTGTCTACTGCCTCCAACGTAACTCCAGAAGACTTGGCTGAAGAGTTTGATCCGTTCCCATCAAGAAAGAATAGCATTGATGATCTAAGAAGGAGATACGATTTACTTCGAAGTCTTGGGGGAAGGATTCAGACAGTGCTTGGTGATATAGCAACTCTAGGGGAGAGAGTGCAGTCTTTGAATCTTAGCGTTTGTTGCATTCATTTACGAGCTAAGGCACCCAAGTTTGCGAATCGATCTCCCTTCATTCCCACGAAACTTCCTCAGGAGGATGCCAGCCAGATCTGA

>Frves19060

ATGAGCAACCTCAAGTTAGGGGTTGAGGTTGTGGCTGCCCATGACCTCATGCCCAAAGATGGAACAGCTAGCACCTTTGTGGAGCTTCACTTTGATCATCAGAGATTCCGAACCACTGTAAAAGAAAGAGATCTCAATCCTGTCTGGAATGAAAGTTTTTACTTCAATGTTACTGATCCAAATGACCTCTCTAACATGAACCTTGAAGCCTATGTTTACAACCATGGTAAAGCCAACACCAAAACATGCCTGGGGAAGGTTTGTCTTACTGGGACATCATTTGTCCCATACTCTGATGCTTGTGTTTTGCACTATCCTTTGGAAAAGAAAGGCCTTTTCTCGCGTGTGAAAGGAGAGCTTGGCCTCAAAGTTTTTGTTACTGATGACCCATTGATCAGATCCTCAAATCCACTGCCAGCAATGGACTCTTCGATGGATAGAGGTTCACGCCACACACATGGTCAAGCACCCTTGCAACAAGTTCCAAATGTTGTCCCAAACCCATTCTCTGATGACAGAGCTGATTCAAGACACACATTTCGTCACCTTCCAAATCCTACTGTAGCACAACAGCAGAATATTCCTTCAGCGGCTACCCAGCCATCAGTGAACTATGGGATGCAGGAGATGAGGTCTGAACCACAAGGACCTCAAGTTGTTCGCATGTACTCAGGTTCATCATCTCAACCTTCTGATTATATGGTTAAAGAGACAAGTCCTTTCCTTGGAGGGGGTCAAGTTGTTGGTGGGCGGGTTATACGTAGCAACAGACCATCCAGCACCTATGACCTTGTTGAAAAGATGCAATACCTCTTTGTACGAGTTGTGAAAGCTCGTGACCTTCCTACCATGGATGTTACAGGTAGCCTTGATCCATATGTTGAAGTAAAAATAGGGAACTACAAAGGAACTACAAAGCATTTTGAGAAGCAGAAGAATCCTGAATGGAATGAGGTGTTTGCCTTTGCTAAGGACAATCTGCAAGCTCACACATTGGAGGTTGTGGTCAAAGATAAGGATCTCATGAAAGATGATTATGTTGGGTTTGTGCGTTTTGATCTCCATGAAGTTCCTACACGAGTTCCACCTGATAGTCCCTTGGCTCCAGAATGGTACCGGATAGAAAACAAGAAAGGGGAGAAGAGGAATGGGGAACTGATGCTTGCTGTATGGTATGGTACACAAGCTGATGAAGCTTTTCCAGATGCATGGCATTCTGATGCAATTGGTCCTGATGATACTTCTTCAGCTACTTATGCACACTCGCGCTCAAAAGTTTACCATTCACCAAGATTATGGTATGTACGAGTAAATGTAATTGAGGCACAGGACTTGATTATATCTGACAGGTCTCGGTTCCCAGATGCATATGCAAAAGTACAAATTGGTAATCAGGTCCTAAAGACAAAAACAGTTCAAACTCGAGTTTTGAACCCAATGTGGAATGAGGACCTGATGTTTGTTGCTGCTGAACCATTTGATGACCATCTGATAGTTTCAGTTGAAGATCGTGTTGGTCCAAACAAAGATGAGACCTTAGGAAGGGTTGCCATACCTTTGAACACTGTTGAGAGGCGTGCTGATGATCGAATTATCCGTGGGAGGTGGTATAACCTTGAAAAACATATGTCAGATGCCTTGGAGCTGGAGGGAGAGCAGCGGAAGAAAGATAAAGAAAAAGATAAGTTTTCCAGCAGAATCCATCTTCGTGTTTGTCTTGATGGAGGGTATCATGTGCTTGATGAGTCTACTCACTATAGTAGTGACCTTCGACCAACAGCCAAGCCACTTTGGAAGTCATCCATTGGTGTCTTGGAGCTCGGAATTCTGAATGCTGATGGTTTACACCCAATGAAAACAAGGGATGGGAAGGGGACAGCAGATACATATTGTGTAGCAAAATATGGGCACAAATGGGTTCGTACTCGGACCATAAATAACAGTCTAAGCCCAAAGTACAATGAGCAGTACACTTGGGAGGTATTTGATCCTGCAACCGTTCTCACAGTGGGGGTTTTCGATAACACTCAGATTTTCAGTAATTCAAATGGCCACAGAGATGTGAAAATTGGCAAGGTCAGGATTCGTATGTCTACCCTTGAGACCGGTCGCGTCTACACACACTCTTATCCACTACTAGTCCTTCATCCTTCAGGTGTCAAGAAGATGGGTGAATTGCATCTTGCAATAAGATTTTCATGCACATCACTGGTTAATATGATGTTCAAATACTCTAGACCTCTTTTGCCGAAGATGCATTATGTAAGGCCATTGACTGTGATTCAGCAAGACATGTTACGTCACCAAGCTGTCAACATAGTTGCAGCTCGGCTTAGTCGTGCGGAACCACCTCTTAGGAAGGAAGTGGTGGAATATATGTCTGATGCAGACTCTCATCTTTGGAGCATGAGGCGTAGCAAGGCAAACTTTTTCAGACTGATGACCGTTTTTGCAGGATTGTTTGCTGTTGGAAAATGGTTTGGAGAAGTATGCATGTGGAAAAACCCCATCACAACAGCGCTTGTACATGTTCTCTTTGTGATGCTTGTGTGCTTTCCTGAACTGATTCTGCCAACTGTTTTCCTCTACATGTTTCTGATTGGGATTTGGAATTTCCGCTATCGCCCAAGGTACCCTCCTCACATGAACACAAGGATCTCATATGCAGATGCCGTGCACCCGGATGAGCTTGATGAAGAATTCGACACATTTCCAACATCTCGGGGTACAGATATAGTTCGGATGAGATATGATCGCTTAAGGAGTGTCGCTGGAAGGATTCAAACTGTGGTGGGTGATGTTGCAACTCAAGGGGAACGGATTCAATCACTTCTCAGCTGGCGGGATCCTCGTGCCACAATGCTATTTATCACATTTTGTCTTGTGGCTGCTATTGTGTTATATGTGACACCTTTCCAGGTTTTGGTCCTTCTTGGTGGGGTTTACTTCATGAGGCATCCCAGGTTCAGGCATAAGATGCCATCAGCCCCAGTTAACTTTTTCCGAAGGCTACCAGCAAGGACAGATAGTATGTTGTAG

>Frves20648

ATGCAGAAGCCTCCGCAGTCTACAGATTTCGCTTTAAAGGAGACCTCGCCCAACATTGGGGCGGGGTCGATCACAGGTGACAAGCTCTCTTGCACCTATGATCTTGTTGAACAGATGCAATACCTTTATGTTCGGGTGGTAAAAGCCAAGGATTTACCTGGAAAAGATGTAAGTGGTAGTTGTGATCCTTATGTTGAAGTGAAACTTGGTAACTACAAGGGAGTCACTAGGCATTTCGAGAAGAAGTCTAACCCTGTATGGAATCAAGTATTTGCTTTCTCCAAAGATCGAATTCAAGCTTCCATTTTGGAAGCTGTGGTAAAAGACAAGGATGTTGTGGTTGATGATTTCATGGGGAGGGTTATATTTGAACTAAATGACATACCGAAACGCATTCCACCTGACAGCCCTTTGGCACCACAATGGTATAGGCTGGATGATCGAAAAGGTGTTAGGGTAAAGGGAGAGCTGATGTTGGCAGTTTGGATGGGAACTCAAGCTGATGAAGCATTTCCTGATGCTTGGCATTCAGATGCTGCAACAGTTGGGCCAGAAGGTGTGAATAACATTCGATCAAAGGTGTACCTCTCCCCTAAGCTTTGGTATGTAAGAGTTAATGTAATTGAAGCTCAGGATTTGTTGCCTACTGACAAGAGTAGGTACCCCGAAGTTTTTGTGAAGGTTGTCTATGGGAATCAGGCATTGAGGACTAGAATATCACAGAGTAAAAGTATCAATCCTATGTGGAATGAGGATTTGATGTTTGTTGCTGCTGAACCATTTGAAGAGCCTTTGTTTTTGACTGTGGAAGATAGAGTGGGATCAGGCAAAGATGAAGTCTTGGGGAAGTGTGTCATTGCTTTACAGAATGTGCAGCGGAGGTTAGACCATAAACCTGTTAACACAAGGTGGTTTAATCTAGAGAAGCATGCAATTGTAGATGGGGAACAGAAGAAAGAGATTAAGTTTGCTAGCAGGATTCATTTAAGGGTTTGTTTGGATGGCGGGTATCATGTTCTGGATGAGTCGACACACTACAGTAGTGACCTTAGGCCTACAGCAAAGCAGTTGTGGAAACCAAGCATTGGGATTTTGGAGCTCGGGGTTATAAGTGCAGTGGGACTGATGCCAATGAAGGCGAAAGATAGCCAAGGAACCACAGATGCTTATTGTGTAGCTAAATATGGGCAGAAATGGGTTCGAACAAGGACTATTGTGGATAATTTTACTCCCAAGTGGAATGAGCAGTACACATGGGAGGTCTTCGACCCGTGTACTGTCATTTCAATTGGGGTTTTTGACAATGGTCATATACATGGGGGTGATAAAGGGGGAAAGGATTCAAGAATTGGGAAGGTAAGGATACGGCTATCTACACTTGAAGCTGACAGGGTTTACACACATTCCTATCCTCTTCTTGTCCTGCATCCTTCCGGCGTGAAGAAAATGGGCGAAATTCAACTGGCAGTGAGGTTTACATGCTCGTCTTTGGTTAATATGTTGCACATGTATTCACATCCTTTGTTGCCAAAAATGCACTACATTCATCCATTGTCTGTAATTCAGCTAGATAGCTTGAGGCATCAGGCTATGCAGATCGTCTCAATGAGGCTCAATCGGGCTGAACCACCACTGAGGAAAGAGGTGGTGGAGTATATGCTAGATGTTGATTCGCATATGTGGAGCATGAGAAGAAGCAAGGCCAACTTTTTCAGAATAATGGGAGTTTTAAGTGGGGTGATTGCAGTAGGAAAATGGCTCGATCAAATCTGCAACTGGAAGAATCCTCTTACAACCATTTTGATTCATGTCCTTTTCATCATATTGGTTCTTTATCCGGAACTAATTCTTCCCACTATTTTTCTTTACCTGTTCCTGATCGGCATTTGGAACTTCAGATGGAGGCCAAGACACCCTCCTCACATGGACACCAGGCTATCTCATGCTGATGCTGCTCATCCTGATGAACGTGATGAAGAGTTTGACACATTCCCAACTTCACGGCCATCAGACATTGTTCGAATGAGGTATGATCGACTGAGAAGTATAGCAGGGAGGGTTCAGACTGTTGTTGGTGATATGGCAACTCAAGGCGAAAGATTTCAGTCTCTTCTGAGTTGGAGAGACCCAAGAGCAACCACTTTGTTTGTTACTTTCTGTCTGATTGCTGCCATTGTTCTCTATGTGACGCCATTCCAAGTTGTGAGTCTTCTAACAGGAATTTATGTGTTGAGGCATCCCAGGTTCCGCCACAAGCTTCCTTCAGCACCACTCAACTTCTTTAGAAGGTTGCCTGCAAGATCAGACAGATTCAGTTTGAACATATCCAATGCAGAACTAGAATTTGGGGTGTCACTGCTAACTAGGTGCATTTAA

>Frves22671

ATGCAGAGGCCTCCACCTGAAGACTTTGCTTTGAAGGAGACCAAACCCCATCTTGGTGGGGGGAGGATCTCCGGCGACAAGCACACGAGCACCTATGACCTCGTTGAGCAGATGCAGTATCTCTATGTCCGAGTTGTGAAGGCCAAGGACTTACCTTCCAAGGATGTTACCGGTAGCTGTGACCCTTATGTTGAAGTTAAGCTGGGGAACTACAAGGGTGCAACTCGGCATTTTGAGAAGAAGTCCAATCCGGAGTGGAATCAGGTGTTTGCATTCTCGAAGGACCGGATCCAGGCTTCAGCTCTTGAGGTGATTGTGAAGGATAAGGATCTTATGAAGGATGACCTCATTGGATATGTTATATTTGACCTGAACGAGGTTCCGAAAAGGGTTCCTCCTGATAGTCCCCTGGCGCCACAATGGTATAGATTGGAGGATAGGAAGGGGGACAAGGCTAGGGGTGAGTTGATGTTGGCTGTTTGGATGGGTACTCAAGCTGATGAAGCATTTCCTGAAGCATGGCATTCGGATGCTGCCACAGTTAGTGGAGCTGATAGTCTGTCAAATATTCGGTCGAAGGTGTATCTCTCTCCCAAACTATGGTATTTGAGGGTTAATGTGATTGAGGCTCAGGACTTGATGCCAAGTGATAAAGGCAGGTATCCCGAAGTTTATGTGAAGGCTATCCTGGGAAATCAGGCTTTGAGAACTCGAATTTCACAAAGCAGGAGTATAAATCCCATGTGGAATGAAGATCTAATGTTTGTAGCATCAGAGCCGTTTGAGGAGCCGTTGATTTTAAGTGTGGAAGATAGAATCGCGCCAAACAAGGATGAAGTTCTGGGGAGGTGTGCCATTCCTTTGCAGTATGTGCCCCGAAGGTATGATCATAAACCTGTGAACACTAGCTGGCACAATCTTGAGAAGCATATTATTGTAGAAGGTGAAAAGAAGAAGGATGTTAAGTTTGCCAGTAGGATTCATATGAGGATCTGTCTAGAAGGTGGTTACCATGTGCTTGATGAGTCAACACACTATAGCAGTGATCTTAGACCAACAGCCAAACCATTGTGGAAGTCCAGCATAGGGGTTCTGGAAGTGGGAATCCTGAATGCTCAGGGGTTGATGCCAATGAAGACAAAAGATGGGAGGGGAACAACAGATGCCTACTGTGTTGCAAAATATGGGCAGAAGTGGGTTCGTACAAGAACCATTATCGATAGCCTTGCTCCCAGGTGGAATGAGCAATACACTTGGGAGGTTTTTGATCCTTGCACCGTCATCACAATTGGGGTATTTGATAACTGTCATTTGCATGGAGGAGACAAGGCTGCCGGGGCAAAGGATTCACGAATTGGGAAGGTCAGGATTCGTCTCTCTACCCTTGAAACTGATCGGGTGTACACGCACTCTTATCCTCTTCTGGTTCTGCACCCAAATGGTGTGAAGAAGATGGGTGAAATACATATGGCTGTGAGGTTCACCTGCTCTTCGTTGCTGAACATGATGCATATGTACTCCCAACCACTGTTACCCAAAATGCATTATCTTCATCCACTAACTGTAAGCCAGCTTGATAGCTTGAGGCACCAGGCCACTCAGATTGTATCAGTGAGGCTGAGCCGTGCTGAACCGCCCTTGAGAAAGGAGGTGGTCGAGTACATGCTGGATGTGGGCTCGCACATGTGGAGTATGAGAAGAAGCAAAGCTAATTTCTTTCGAATTATGAATGTTCTGGGTGGATTAATTGCTGTTGGAAAATGGTTTGATCAGATCTGTAACTGGAAGAACCCCATCACTACTGTGCTTATCCATATCTTGTTCATAATTCTGGTTATGTACCCAGAGCTGATACTGCCTACTATTTTTCTGTACCTGTTTCTGATTGGAGTCTGGTACTACAGATGGAGGCCCAGGCATCCTCCTCACATGGACACTCGTCTCTCACATGCAGATTCTGCACATCCTGATGAACTTGATGAAGAATTTGATACTTTCCCGACTTCACGTCCTTCTGACATAGTGAGAATGAGATATGACAGATTGAGGAGTATTGCTGGGAGAATTCAGACAGTGGTTGGTGACTTGGCTACTCAAGGGGAGAGGCTGCAATCATTGTTGAGCTGGCGTGACCCGAGAGCTACAGCTCTGTTTGTGCTTTTCTGTCTGATTGCTGCTATTGTTCTGTATGTTACGCCATTCCAGATTGTGGCTCTTCTTGCTGGATTCTATGTGTTGAGGCACCCAAGATTCCGCCATAAGCTTCCATCAGTGCCACTGAACTTTTTCAGGAGCCATGAACATGAAGGTGGTGAGGGTCTTCTTTTCTTTCAGTGTTCTGCAGCAGCGTTCTGGTACGATGTATTTATGGCCTTGTCCTGA

>Frves24406

ATGGAACACAAAACTGGTGTTCCGGAAAAGAGAAAGCTAAACCTAACCGTGGCCGCCAGTAACAGCTCCGTTTCTCGGCTGAGTAACGACGAAGTAGGCTTAATTCTAAACTCCGTCACCAACTCAGATGATAGAAAAGCCTTCTTCAAGGTCTGCAAGCAATGGTGTAGAGCAGAGGGTCTAAACCGAACATCCATTCGTCTTCTCCAACTCGATTATCTCCGGCGTGTACTTCCCAGATTCCCGAACTTAGTGACATTCGAAACATCCAAATGTATCACGCGAAACGACCTCGAATTCTTGGCCCAAACATGTCCCAAAATCGAAACCATCGACCTTGGAAAATACCTTGGAAAATCCATAGATGATGTAACAACAGAACCTAGCCATTCTCAGATCGAAGTTCTGTATGCTCTGGCACATAGCTTCCCGAAATTGTCTAAGCTTTCTTTGAGAAGAAATAGACACTACAGTGGGGAAGGTATTACTCAACTCATAAAAGAGCTAGTACAAAACAAGTTGAGGCATTTGGATTTAGGGTATTGCTGTGTGTTAGATGAAACCCTTGAAGCGATTGGGTCGTTGGGTTGTCTTAGCTATTTGAATCTGGATGGGTGTGACAGGATCACAGATCAGGGTTTAGGGTTTCTGGCATATGGGTCTTGCTCAAAAACCTTAAAGACATTGGTGCTTACTGGGTGCAGAGGGATCACCGATTCTGGTGCCGATCCTTTGCATAAGATGGCTTTCTTGGAAGAGCTGTATTTGGATGGGGAGACCGGGTTCAGTTTGGATGGACTTCGAGCATGGTCGCGTCTCTCCGTCAAGGAACTTGGTTTTTCCGGCTCCAATTTGTGGCCTGTGGAGGAGGCCCAAATAAGAAGATGCCCAACAACAAATGTCGGCCCAAGTCCAATACATTTCAAGACTTACCCCAAACCATGGATCACGTCTGAGCCCAAGTCAAAAAAGAAAAAAGAAAAATATCCCCGGTATTTTCTGGCGCCAATTCGTCATCCCAATCCCGTTCGCGACGTGTTTGGTCCAGAAAGCTCCACGTGGCAACACAACAATAAGATTCACACTCACGGACTGTGTGACCTTCAAGCCTCTTCTTCTTCTTCCTTCTTCAATTCAATGGCTCTCAGACCTCACACGGTGACGTCAGCACCGACCTTCCATTCCCGCCAATTCCTCCACAGGCCCACATTCCCGCCCAGAACTACTCTTCGCTTCCGAAACTCTTCGTCGACGCGAGCGAACTCCCTCACCGCCGAACAAGTCTCGTTCACGGGGGAAGAGAACTCCCTCGTCGAAGCCCTCATCGGAATCCAAGGCCGCGGCCGCTCCGCCTCTCCTCAGCAACTTAATGAGGTTGAGAGCGCTGTGAAGGTTCTTGAAGCTCTGAAAGGAGTTCCTGAGCCGACAAGCTCGAGTTTGATTGAAGGGAGGTGGCAATTGATGTTTACTACAAGACCTGGAACCGCCTCTCCAATCCAAAGAACATTTGTGGGGGTGGACTTCTTTAGTGTATTTCAAGAGGTTTATCTTCAGACAAAGGATCAGCGTGTGTCGAATATTGTAAAGTTCTCTGATGCAATAGGTGAGCTGAAAGTAGAGGCAGAAGCATCAATCAAAGATGGAAAACGAATACTTTTTCGATTTGACAAAGCGGCATTTTCGTTTAAATTTTTACCATTCAAGGTCCCATATCCAGTGCCATTCAGACTTCTTGGGGATGAGGCAAAGGGTTGGTTGGATACCACATATCTGTCCGAATCTGGCAATCTTCGTATATCGAGAGGAAATAAGGGAACAACATTTGTGCTGCAAAAGAAAGCTGAACCTAGGCAAAGACTATTGTCAGTCATCTCGACTGGTACAGCAGTTAAGGAGGCAATTGAGGAGTTTATCTCCTTAAATCAAAATGTAGGTGAATCAGAACTCCAGGAGGGAGAATGGAAAATGGTTTGGAGCTCACAGGAAGAGACAGATAGTTGGCTTGAGAATGCTGCCAATGGTCTTATGGGCACACAAATTGTCAAGGGAAATGAACAAATAAAGTTTGTGGTTGACGCGTTCCTTGGGCTCAAATTCTCCATATCTGGAACTCTTGTAAAGTCTGGCTCCCGCACTTATGATGTTACAATGGACGATGCAGCCATCATTGGCGGTGGCTTTGGATATCCCCTAGAAGAGCTAGGAAGCAAGTTTGAACTAGAACTGCTATATAGTGATGACAAGATCAGAATCACCCGGGGATATAATAAAATACTCTTTGTGCATGTACGAACTGATGGTTTGAAACAGAAACATGTACGTAACGACGTAGCAATGGCTAATCAAATGAAAGAAAATTTCTCTGTCAAGGAGACCAAACCCTCTATCCGTGCCAAGAAGCCGTTGCACGGTCCCACCACCTCCTTTGATCTTGTGGAGGAGATGTTATATCTGTATGTCAGGATTGAGAAAGCCAGAGGTGTGCCAGTTCAAAGTCACCTCCAAGTTGAACTGAAGATTGGGAACTACAGAGGAGCCACAACAACCACGGCGCAAAGTCCACAGGGGGAGTTCAATTGGTACCAGGTGTTTGCTTTCACCAAGAGCAGACTTCAAGATACAACTGTGCATGTTTCGGTGAAGGCTGATGGAGGTGTTGTAAGCAAATGTAGCTTTGGTGTATCGGAAGCATTGAGGAGAGTACCACCTGATCCTCAATTGGCAGCTCAGTGGTATCGACTGGCGGATACAAAAGGGAACGGATTTGGAGGAGAGTTGATGATGGCATTTTGGACTGGGACACAGGTTGATGAGGTCTATAATGTTGCTTCGCATTCTGATGCAGTAGCAGCTCTTAGCGATGATGGTTTGTTGAGTATTTGTCCAAGAGTGTATCATACCCCGAGGTTTTCGTACCTCAGAGTAAATGTAATTGCAGCACAGGATTTGGTGATCAATGACATCAACAACAGAGTCCAGCCGGTGCAGGTTTTTGTGGAAGCTAGTGTTCTGGATTGTAAATTTAGAACCAAAGCTTGTTCAACCAAGACTGTGGATCCTAAGTGGAATGAGGACTTGATGTTTGTGGTTGCGGAACCATTTGATGCAACTTTGGTGGTGGAAGTCTATGAGAAGGCAGTTGTTGTTAATGGGGAAGTGAGACATGGGGAACGTTTAGGGAGGTGTGAGATTCCTATAAAGAATGTGGGGAAGAGGACTGATAGAACACCGGCTGCTAGTCTGTGGTATGATCTTAAGCTCGTGCCGGATCAGCCGAGATTTGCTAGTAAGATTAATATGAGGATCAGTTTGGATGGTGGATATCATGTTTTCGATGATCCTACTGACTTTCCTAGTGATCTTAGGCCGTCGGCAAAGGTTCTGTGGAGACCGCCTGTGGGGACTTTTGAACTTGGAATTATGAGTGCCTCTGGATTGTCCCCAATAATGCCTAAGACTAGTGTGGACACTTTCTGTGTCGCTAAATATGGGCCAAAGTGGGTGAGGACTAGAACAGTCATTGATAATCGTTCTCCAAAGTGGAATGAACAATACATGTGGGAAGTCTTTGATCATTGTACAGTCATAACTATTGCAGTTTTCGATAATAAGTATTTGCTCCAAGGGGGAGAAGTAGCTGCTCATCAGGGAATTGGAAGAGTAAGGATTCGACTATCCGATCTTGATTCTGGTAAAGTTTACACAAACTCGCATCCCCTTGTGAGCCTAGAACGTTCTGGGGTGATGAAGAGAGGTGAACTTCAATTGTCTTATCGATTTTGTCCTACATCTAAGTTTAATGTGTACACTAGATATCCAGAGCCCCTTTTGCCCAAGCTGCATTATATTCTCCCACTATCGGTACCTCAGATTCTCATACTAAGGGCTCAAGCTGTTCGGCTTATACAAAAAATGTTGCAGAGGGCTCAATTAAGCGGAGAGGTTGTGCAGTATGTGTTGTTGGACAACAAATATTCTTTTAGTACGCGGAGAGCTAGGGCTAATTTTTTGAGGTGTAAGAAACTTCAAGAAAGTTTCCTAGTTTGCAAGCAGCAATTTGATCGGCTGCTGAGTTGGTCTAATCCACGCCATACTATTGGCTTTATAATTTTCCTCATTGCAGCCCTTTGCTTCCCTAGTTTGGCACTAGCTTCCATTTTTTTCACGATTTCCGGTGTAGGGGCTTGGGGTTACAGGAAGAGGCCTAGACAACTTCCTCACATAGACACCGAATTGTCACAGCTTAATAGTGTCGACCGTGACGACATTGCCGAAGAGTTTGATGGAGTTGTGACGGAGGAGAGACATGTTCAACTTCAAAAGCTTTTAGTGGTTGTTCAGACACTGCTCGGTGACATTGCAGCTACAGGGGAGAGGGTGCAGTCTTTGTTGAGTTGGAGGGATAAAAGAGCTACACTTCTTGTTCTGATACTTTCTTTCATTGCTGGGCTTGTTTTTTACTTCGACTTTTTGGACTTGCGTGAGAATGCCATCAAAGATAGACAGCATGCTATAAGGAGCTGTAGCTTGTCATGGAGGCAGTCTGTTCAAGAAAGATCAAGTAAAAAAGAGAAACCTAAATGCAGCATAATCTCCGTTCTTACCCACAGCCACATGGTGGAGTTTGTAGTAAAAGACTCCATGCAACAAAATAAAGATTGGAAGATCACAGATAGAGCTGATGAAGCAGCTTCCTCAAATGATAAGCATATCTGCTCAGAGATAGACTTGAAACATTCAATCTTTAGACTTTCTGATGATGGAGAAAACAGTCCAACTGCTTATACTCCGCCGGTATGGGCTGGCTCAAATTGCGAACTTGGAGATACCCTCAAAGAAATACAAAAGATTGGCAAGGGTGTAAGCCACTTCCATGAAAGATAA

>Frves25064

ATGGCAACTGTTCGGAAGCTCATAGTTGAGGTGGTCGACGCACGGGACCTTCCGCCCAAGGACGGCCACGGCACGGTGAGTCCATACGTCCAGGTGGACTACTACGGCCAGCGAAAACGGACGCAGACTGTTATAAAGGACCTGAACCCTAAATGGAACGAGCTTCTCGAGTTCAACGTCGGGAAACCTTCTGACGTTTTCGGGGATGTGCTGGAACTCGACGTCTATCATGACAAGAACTACGGCCCCACCACCCGGAACAACTTCCTCGGCCGACTCCGGCTGACGTCGTCTCAGTTTGTGAAGAAAGGCGAGGAGGCTCTTATATACTTCCCCTTGCAGAAGAAGAGCCTCTTCAGCTTTATTCAGGGCGACATTGGCTTGAAGATTTATTACGTCGACGAGGCTCCACCACCTCCTCCTCCGCCGCCGGAGGAACCCAAGGCTCCTGAGCCCGCGCCGCAAGAGGAAGCTAAGCCTGCTAAAACTACGGCTCCACCACCGGCTGAGGAAGCAGCACCACCACCTCCGGAATCTGAGAAGAAAGAAGAAGCGGCGCCACCGGTAACGGAGCTACCGCCGCCGTCAGAAAACGAGAAACCTGCTGAGGAACCACCGGCTGAGGCAGCCGCGCAACCACCGGAGACTGCAGCAGCTGAAACTGCAGAATCCGATCAGTTACCGCCACCACCGCCACCGCCACCAGAAATCTATGCCGAGAAGCAGCAGCTAGGTCAAAATGATCATATTGAGATGATGTCAGCTTCTTCCGTATCGAAATCAGTGCCGGAGATCAAGTTCGTCGGCGGAATCAACGGTCCACAGCCAATGGCCCGCCGCCCCTCAGGAGTCCCAAGCTACACACAGCTGGAGCCAACTGAGAGTATGTCAATCGATCGTCCGACGTCGTTTGATCTGGTGGAGAAGATGCATTACCTCTTCGTCCGAGTAGTCAAGGCCCGCTACCTCCCTGCCAACGGCAGACCCGTCGTCAAGATCTCCGCCTCCAACTACCACGTGACGTCAACCCCCGCCAGAAAAACCAACTGCTTCGAGTGGGACCAGACCTTCGCTTTTGGCCGCCAGTCCCCGGACTCCGCTTCCATCCTCGAAGTCTCCGTCTGGGACCCGCCAATCCCCGACCCAACCGGCGTGGCCTCCGGGCACAACTTCCTCGGCGGAGTCTGTTTCGACGTGGCGGAAATCCCGCTGAGGGACCCACCGGACAGCCCCTTGGCCCCGCAGTGGTACAGATTAGAAGGAGGCGGATCCCGCATCAACGGGGATCTGATGCTCGCCACGTGGATGGGTACCCAAGCCGACGAATCATTCCCCGACGCGTGGAAGACCGACACCGCCGGAAACCCTAACGCACGCGCGAAGGTGTACCAGTCCCCCAAGCTCTGGTATCTAAGGGCAACAGTCGTAGAAGCACAAGACGTCGTCCCCATAACGACGTCGTTAAAGGAAGCCACGTTCCAAGTCAAAGCCCAGCTGGGCTTCCAGTCTCTCAAGACCGAGGCTTCCCTGACCCGAAACGGCACGCCGTCTTGGCACCAGGACTTGATATTCGTGGCCGCCGAGCCCTTCACCGATCACTTGGTGTTCGTATTGGAGAACCGGCAACCGAAAGGGACGGTCACATTGGGGTTCGCAAAGATACCACTCACCGCCATCGAACGCCGTGTCGACGACCGGAAAGTGGCGTCGAAATGGATCAGCTTGGAGGATCCAAAAGACGAGAAGAGAATGTACACCGGAAGATTGCACGTGCGGCTTTACTTCGATGGAGGATATCACGTGATGGATGAGGCGGCGCACGTGTGCAGCGACTACCGCCCCACGGCCAGGCAGCTATGGAAGCCACCGGTTGGCACTGTGGAGCTTGGTATTATTGGATGCAAGAACTTGATACCGGTCAAGACTGTGAACGGTAAAGGTTGTACGGATGCGTATTGTGTTGCCAAGTATGGTTCTAAGTGGGTACGTACACGGACCGTATGCGATAGCTTGGAGCCCAGGTGGAATGAGCAGTATACTTTCAAGGTCTTTGATCCTTGTACAGTGTTGAGTATCGGTGTGTTTGATAGCAGTGGAGTCTTCGAAACCGACGGCCCGAGGGACGCCACGCGTCTCGACTTTCGAATTGGGAAGGTACGTGTACGTATATCGACTCTGACTACGGGTAGAGTGTACAAGCATACGTATCCGTTGTTGGTCTTGTCTCCGGCGGGTTTGAAGAAAATGGGGGAAGTGGAGATTGCCATACGCTTTGCTCGTGTGAGTCCTATCTTGGATTTGGTCCACGTCTACTCGCAGCCTTTGTTGCCTTTGATGCACCACATAAAGCCGCTAGGAGCGGGGCAACAAGAGATGTTGAGGCGAGCGGCGGTAAAAATCGTGGCAGCACATCTGTCACGATCGGAACCGCCACTTGGCCGTGAGACGGTTCTCTACATGTTGGATGCGGACTCACAAGGGTTCAGCATGAGGAAAGTTCGTGCTAACTATTTCCGGATCATCAATGTCGTGGCCGGAGTAATGGACATTGTGGGGTGGATAAACGACACTCGTTCGTGGAAGAAGCCGATGGCGACTATATTGGTGCACGCATTGTTGGTGCTGTTTGTGTGGTTTCCTGATTTGATAATCCCAACTTTGCTATTTTACGTCTTCGCAATCGGCGCGTGGAACTACAGGTTCCGTTCTCGGGTCCCACTTCAACACTTTGATCCAAAGCTCTCACTGGCAGATACAGTTGACCGTGATGAACTTGACGAGGAGATCGATATGGTGCCAAGCAGCAGATCGTATGAGGTGGTGAGGGCCAGGTACGATAAGCTACGGACGCTTGGGGCACGTGTCCAGACGGTATTGGGGGATTTTGCCACGCAAGGAGAGCGCGTGCAGGCGTTGGTGACGTGGCGTGATCCACGTGCGACGGGGATTTTTGTTGGACTGTGCTTTGTAGTAGCAATGGTGTTGTACTTGGTTCCGTCCAAGATGGTGGCAATGGCGTTCGGGTTTTACTACCTGCGCCATCCGATCTTTCGCGACAGAACGCCGTCGCCGGCCCTGAACTTTATGAGGAGGCTTCCTTCACTGTCCGATCAACTTTTGTAG

>Frves25947

ATGACTGTGTCGTTTCAGCAACCCCCACCTCCCCAATCCCAATCCCAATCCCAATCCCAACCCCAACCACAACCCCAGCCCCAACCAGAGCAGCCACCGCAACCGCAACAGCCACATCAGCCGCAGAGGACGATCAGGAAGCTCATTGTGGAAGTGATCGACGCACGTGACCTTCTACCTAAAGATGGCCAGGGCAGCTCCAGCGCTTACGTGGTGGCGGACTTCGACGGGCAGCGGAAGCGGACGGCGACCAAGTGCAAGGACCTCAACCCGGTCTGGAACGAGCCGCTCGAGTTCGTCGTCTCCGACCCGGACAACATGGACTACGAGGAGCTCGAGATCGAGGTCCTCAACGACAAGCGCTACGGCAACAGCGGCACCGCGCGTAAGAACCACTTCCTCGGGCGCGTCAAGCTCTACGGCACTCAGTTCTCCAAGCGCGGCGACGAGGGCTTGGTCTATTTCCAGCTCGAGAAGAAGAGCGTCTTCAGCTGGATCAGAGGCGAAATCGGCCTAAGAATTTACTACTACGACGAGCTAGTCGACGAGGCTCCGCCGCCGCCGCAGCAACCCCCGCCGCAGGAGGATCCTCCGCCGGAGCAGCCGGCGGTGATGGTGGTCGAGGAAGGGAGGGTGTTTGAGGTCCCCGGAGGACACGTGGAGTGTACTAGAATCCACGACGGCTCGTACTCGCCGCCGGTTGTTGTGATGGAGCAACCGCCGCCGCAGATGGTGCATATGCACTCCGAGCCGCCGGGACAGGAAATGCACGGTCATCCGCCGCCGCAGGAGGTTCGTTTTCAACCGGAGGTGAGGAAAATGGAGACTCACCGAGTTGCTCCGATGGGGGAGAGAGTGAGAATTCCCAGGAGGCCGAACTGCGACTACTCTCCGAAAGTGATTTCCGGAAAGTTTGGGGCGGAGAACACCGCGGAGAGGATTCACCCCTGTGAGCTCGTCGAGCCTATGCAGTACTTGTTCACCAGAATCGTCAAGGCGCGTGGCCTCGCGCCGAACGAGAGCCCCTACGTAAAACTCCGTACATCCAGCCACCTCGTCAAGTCGAAAACCGCGGTTCACCGCCCCGGAGAGCCGACCGACTCGCCGGAGTGGAACCAGGTCTTCGCACTCGCTCACAACCGGCCTGACTCAGTGAGTTCGACATTGGAGATCTCCGTGCGTGACTCGCCGTCGGAGCAGTTCTTAGGCGGCATTATCTTCGACCTCTCCGACGTGCCGGTTCGAGACCCGCCGGACAGTCCTCTTGCTCCGCAGTGGTACAGACTAGAAGGCGGCGCCGGTGATCAAAATTCCGGTAAAGTCTCCGGCGATATCCAGCTCTCTGTTTGGATTGGGACACAAGCCGATGACGCTTTCCCTGAAGCGTGGAGCTCCGAGGCCCCTAACGTGTCCCACACGCGCTCCAAGGTGTACCAGTCCCCGAAGCTCTGGTATCTGAGAACGACGGTTATGGAGGTTCAAGACCTCCACATTGCATCGAATCTACCTCCCTTAACGACCCCTGAGATTCGAGTCAAGGCTCAGCTGGGGACTCAGTCGGCTCGGACCAGGCGCGGGTGCATGAACAACCACTGCGCCTCGTTTCACTGGAACGAGGACCTCATCTTCGTGGCGGGTGAACCACTAGAGGACTCACTGATTCTCTTGGTGGAGGACCGCACCAACAAGGATCCGGTACTCCTCGGCCACATCGTTATTCCGGTGAGCTCCATCGAGCAGCGAATCGACGAGCGCTACGTGGCGTCGAAATGGCTCCCTCTTGAAGGCAGAGGAGGAGGCGGGCCCTACAGCGGCAGAATCCATCTACGGCTTTGCTTGGAGGGAGGGTATCACGTGCTGGATGAAGCGGCGCACGTGTGCAGCGACTTCCGACCCACGGCAAAGCAGCTGTGGAAGCCGGCCGTGGGAATTTTGGAACTTGGGATTCTCGGCGCGCGCGGCTTGCTTCCGATGAAGGCGAAATCCGGTGGGAAGGGGTCCACGGATGCTTACTGTGTGGCCAAGTATGGGAAGAAATGGGTCAGGACCCGGACCATTACGGACGGCTTCGACCCGCGCTGGAACGAGCAGTACACGTGGCAAGTATACGACCCCTGCACGGTGCTCACCATCGGCGTGTTTGACAACTGGCGCATGTTTGCTGACGCTTCAGAGGAAAAGCAAGACTTCCGTATTGGAAAAATACGAATACGTATCTCTACGCTGGAGAGCAACAAGGTGTACAAGAATTCATACCCGTTAATGGTGTTGTCCCGGACCGGGTTAAAGAAAATGGGGGAGATAGAGCTAGCGGTCAGGTTCGCCTGCCCGTCGTTGCTGCCCGAGACATGCGCGGTTTACGGGCAGCCATTGCTTCCGAGAATGCACTACCTCCGCCCTCTCGGGGTGGCCCAGCAGGAAGCGCTGCGAGGGGCAGCCACGCGCATGGTGGCCGCGTGGCTTGCACGCTCCGAGCCGCCTTTGGGGACGGAGGTGGTGCGGTACATGCTGGATGCTGACTCGCACACGTGGAGCATGAGGAAGAGCAAAGCGAATTGGTTCCGGATCGTGGCGGTGCTGGCATGGGCGGTCGGGTTAGCTAAATGGTTGGATGATATAAGGAGGTGGAGGAATCCGGTGACGACGGTTTTGGTGCACGTGTTGTATTTGGTTCTGGTTTGGTACCCGGATTTGATAGTCCCGACCGGGTTTCTATACGTGTTTCTTATTGGGGTTTGGTACTACCGGTTCAGGCCCAAGATACCAGCCGGAATGGACCTCCGTCTCTCCCAGGCGGATACGGTCGACCCGGATGAGCTAGATGAAGAATTCGACACTTTTCCGAGCTCAAAGTCGCCGGATGTCATTCGGGTCCGGTATGACCGGTTGAGAATGCTGGCGGCCCGGGTCCAAACAGTTTTGGGCGATTTTGCGACCCAAGGAGAACGGGCCCAGGCCCTTGTGAGCTGGAGGGACCCACGTGCTACAAAGTTGTTCATTGGAGTGTGCTTGCTTATCACTGTGGTGCTATATACTGTGCCACCCAAAATGGTGGCTGTGGCATTGGGGTTTTACTACCTGCGCCACCCTATGTTCCGAGAGCCAATGCCGCCGGCGAGCCTCAACTTTTTCCGGAGGCTTCCGAGCCTGTCGGACCGGTTAATGTAG

>Frves26959

ATGAAGCTGGTGGTAGAAGTTGTAGATGCTCATGATCTTATGCCCAAAGACGGAGAGGGAAACTTGTCTTCGACATCAGCCAAACCGAAAACTTCCATCACAAAACCATTGAAGTTTCTGTGTAATGAGAGGAGAGTCCCTACTCCTGGCCAACATTTCCTTGGAAGTGTAAGAATTCCTTGCTCAAATACTGTCAAGAAAGGCAAGGCAGCTTATCAAAGATTCCAACTAGAGAAGAAGTGGTTTTTCTCATCTGTCAAGGGTGAGATTGGCCTAAGAATCTATATATCACCAGATTCTGAACCAAAATCTCCTCAACCCCAAACACTAACACTAGAAGCCCCACCTTCCAAGTCTCATCAACCACCAGAAGTACACAAAGTCCTATCTCATAGCGAAGTATTTGCTGCTGTTCCTACTGTTTCTTTGATTGGTAACTCCTCTGCTCGAGATCCAAGTGAGGAGACAAAGGAAGTAGTTACATTCCATCACCTACAAAAATATCAAGTGCAGCAACCAGCAGGCATATCTGTCGAGAGACATTGTCAAGGTTTCCCATCAAGCATGCAGCCAGTTCGTCCGCAAGGCCCTCATAGCCATGAAGAAGACTACGACCTCAAGGACACCAACCCTCAACTTGGTGAGAGGTGGCCAAATGGTGGAGCATATGGAGGACGAGGATGGATGAGTGGTGGTGAGAGATTTGCAAGCACTTATGACCTTGTTGAGCAGATGTTTTATCTCTATGTTCGAGTTGTGAAGGCCAAAGATCTCCCTCCCAGCTCTATTACCGCAAGCTGTGATCCTTATGTGGAAGTAAAGCTGGGGAACTACAAGGGAAGAACAAAGCATTTTGAGAGGAAAATGAATCCAGAGTGGAACCAGGTCTTTGCTTTCTCAAAAGACCGCATTCAGTCGTCTGTGCTAGAAGTTTTTGTGAAAGACAAAGAGATGATTGGAAGAGATGATTATCTTGGAAGAGTGGTTTTTGACTTGAATGAGATTCCCACCAGAGTTCCACCTGATAGTCCACTTGCTCCTCAGTGGTACAGACTTGAGCACCGCCGCGGAGAAGGCAAGGTTAGAGGTGAAATCATGGTTGCAGTTTGGATGGGAACACAGGCTGATGAAGCATTTCCAGATGCATGGCATTCAGACGCAGCAGCAGTCTATGGTGAAGGTGTTCACAATATCCGATCCAAGGTATACGTCTCACCAAAATTGTGGTACCTTAGAGTAAATGTGATTGAAGCTCAAGATGTGCTGCCTAATGACAGAAGCCACCTCCCAGAAGTTTTTGTGAAAGCTCAGGTTGGAAACCAGGTCCTCAGAACCAAGATATGCCCAACTCATACTGCTAATCCATTATGGAATGAAGATTTAGTCTTTGTGGCAGCTGAACCTTTTGAAGAGCAACTGATCATCACTGTTGAAGATCGAGTCCACCCTACGAAAGATGAGGTGCTGGGAAAGATGAGCATGCCCATTGACATGTTTGAGAAGCGGCTTGACCACAGGCCAGTTCATTCGCGTTGGTTCAACCTTGAGAAGTATGGCTTTGGTGTCTTGGAACCTGATAGGAGGAAAGAGCTCAAGTTTTCTAGTAGAATTCACCTGAGAGTCTGTCTTGAAGGTGGATACCATGTACTAGATGAATCAACCATGTACATAAGTGATCAAAGGCCAACTGCAAGACAGCTGTGGAAGCAGCCTGTTGGGATTTTGGAAGTGGGGATTCTAAGTGCACAAGGGTTTCTTCCAATGAAGATGAAGGACGGCCGAGGAAGTACAGATGCTTATTGTGTAGCTAAATATGGCCAGAAATGGGTTCGCACCAGAACAATTCTTGACACACTCAGTCCTAAATGGAATGAGCAGTACACATGGGAAGTTTATGATCCCTGCACCGTCATAACGTTGGGAGTTTTCGACAATTGCCACCTTGGTGGCGCTGAGAGACCAACACCAATGGCAGCCAGTGCAGCAAGAGATTCACGAATCGGCAAGGTACGCATTCGACTATCAACACTTGAAGCTCATCGAATGTATACACACTCATACCCTCTCCTCGTTCTGCAACCTAATGGAGTAAAGAAAATGGGAGACCTCCAACTAGCAATTCGGTTCACCACCCTGTCTCTAGCTAACATGATCTATGTTTATGGACACCCTTTGCTGCCAAAAATGCATTACTTGCATCCTTTCACAGTGAACCAAGTAGACAATTTACGATACCAAGCAATGAACATTGTAGCAGTGAGGCTTGGCAGAGCTGAACCCCCACTTAGAAAGGAGGTGGTAGAGTACATGCTAGATGTTGATTCACACATGTGGAGCATGAGAAGGAGCAAAGCCAACTTCTTTCGAATCATGTCACTACTTTCCAGCATGTTCTCCATGAGCAGATGGTTTGGTGATGTATGCAACTGGAAAAACTCCATGACAACAGTTCTCGTTCACATCCTTTTTCTGATACTGATTTGGTATCCAGAGTTGATACTTCCAACTCTTTTTGTCTACATGTTCCTCATTGGAATATGGAACTACAGATTCAGGCCTAGACATCCTCCTCATATGGATATCAAGCTTTCATGGGCTGAAGCAGTTCACCCGGATGAGCTTGACGAAGAATTTGACACATTTCCAAGCTCAAGACCGCATGACATTGTTCGAATGAGGTACGACAGGATTAGAAGCGTAGCAGGGAGGATACAGACTGTTGTGGGTGATATAGCAACACAAGGCGAAAGGTTTCAGTCTCTACTCAGCTGGAGAGACCCAAGAGCAACCAGCCTTTTCATAATATTCAGTCTTTGTGCAGCTGTGGTTCTTTATGCGACCCCCTTCCGAGTGGTTGCTTTGATTGCAGGATTGTATTACTTGAGGCACCCAAGGTTTCGTAGCAAGCTGCCTTCTGCACCAAGCAATTTCTTCAGGAGATTATCAGCTCGAGCAGACAGCTTATTATGA

>Frves31425

ATGATCAAGCTCATAGTCGAAGTTCAGGATGCAAGTGACCTCATGCCCAAGGACGGCGACGGGTTCGCATCTCCCTTTGTTGAGGTCGACTTCGACCAGCAGAGACAGCGGACACAGACCAAACCCAAAGACCTGAATCCTTACTGGAACGAGCAGCTGGTCTTCAACGTCACCAACCCAAGAGACCTTTCCAACAACACCATCGACGTGGTTGTCTACAATGACAGAAAGTCCGGCCACCACAAGAACTTCCTCGGCCGGGTCCGAATCTCCGGCGTCTCCGTCCCTCTCTCCGAGTCAGAAGCCACGCTCCAGCGCTACCCTCTTGACAAAAGAGGCCTGTTCTCCAACATCAAAGGCGACATCGCTCTTCGAATCTACGCAGTTCAAGATCACACTAGTGCTGCTCAACCACAGCAACATGAATATGGTAATGTCGAAACCGGAACTGCTAGCGTCGAGATTCCTCAGATGTTTTCTACTACTCCTCTGCAAGAAATCAATGGTAATAATACTCATAGGATCGATGAGCAAGCCGAGCATCATCATCATCATCAAATGGGTGAGAAGCCGATGAAGAAGAAGAAGGAACATGAAGTAAGAACATTCCACTCCATTGGAACAGGCGGCGGCGGCGGTGGTGGATTCTCTCATTCTCAGCCACCTTCGTCCGGGTTTGGGTTTGAAACCCATCACCAGAAAGCACCCCACGTAGAAACAAGGACGGATTTTGCTCGGGCGGGTCCTGCCACGGTTATGCATATGCAGCAGGGACCTCCGAGGCAGAACCCGGAGTTTGCGCTGGTGGAGACTAGTCCACCACTCGCGGCCCGGCTCCGGTATAGACCCGGCGGGTTTACAGGGGATAAGACCTCGAGCACATATGACTTGGTGGAGCAGATGCATTACTTGTATGTGAGTGTGGTGAAGGCTAGAGATCTTCCGACCATGGATGTGTCTGGAAGCCTTGACCCTTATGTGGAAGTGAAGCTTGGGAACTACAGGGGTGTCACCAAGCACTTGGAGAAGAATCAGAACCCGGTTTGGAAGCAGATTTTCGCTTTCTCCAAGGAGAGGCTGCAGTCGAATTTGCTGGAAGTTAGTGTGAAAGACAAGGATTTTGGGAAGGATGATCATGTGGGGAGAGTTTTCTTTGATCTAACTGAAGTTCCGGTTCGTGTTCCGCCGGATAGTCCTCTTGCTCCTCAGTGGTACAGATTGGTGGACAAGAAAGGGGACAAGGTTAGAGGGGAGATCATGCTTGCTGTTTGGATGGGAACTCAAGCTGATGAGTCATTTCCTGAAGCTTGGCATTCTGATGCACATGACATTAGCCATGTTAATCTTGCTAGCACAAGATCAAAGGTTTACTTCTCACCGAAACTGTATTATCTTAGAGTTCACGTTCTTGAAGCTCAGGACCTTGTCCCTTCAGAAAGAGGCAGACCTTTGGACACATATGTGAAGGTACAGCTTGGTAATCAGATGAGGGTCTCAAGGCCTTCTCAGGTTCGCACTATTAATCCAATTTGGAATGATGAGCTCATATTGGTTGCGTCTGAGCCGTTTGAAGATCTCATAGTCATATCAGTGGGTGACAAGGTTGGACCTGGCAGGGATGACTTGTTGGGAATGGTGTTTCTTTCGGTTAGAGACATTCCACAAAGACATGACACTCATAAGCTTCCTGAGCCTCTTTGGTTTAATCTCCAGAAGCCTTCAGTGGCAGCTGAAGAGGAAAGTGAGAAAAAGAAAGAAAAGTTTTCAAGCAAGATTCATCTCCGCCTCTATTTGGATGCAGGGTATCATGTTCTTGATGAGTCTACACATTTTAGCAGTGATATGCAGCCTTCTTCCAAGCACCTGAGGAAAGCAGGCATTGGAATTCTGGAACTTGGGATTCTTAGTGCCAAGAATTTGCTCCCAATGAAGGGTAGGGAGGGTAGGACTACTGATTCGTACTGTGTGGCGAAGTATGGGAACAAATGGGTGCGAACTAGAACACTCCTCAACACTCTGAACCCTCGCTGGAATGAGCAGTATACATGGGAAGTGCATGATCCTTGTACTGTCATCACCGTTGGTGTTTTTGACAATCATCATATCAATGGAAGCAAAGAAGACGCCAGGGACCAGCGGATTGGGAAGGTGAGAATCAGGTTATCCACTTTAGAAACTGATCGGATTTATACACATTACTATCCTTTGTTGGTTTTGACGCCCTCTGGTTTGAAGAAGCATGGTGAACTTCAGTTGGCACTGAGGTTCAGTTGTACAGCTTGGGTTAATATGGTAGCTCAATATGGAAGACCATTGCTTCCAAAGATGCATTATGTTAATCCTATACCTGTTAGGTATGTTGATTGGCTCCGCCACCAGGCAATGCAGATTGTGGCTGCACGGCTATCTCGAGCAGAGCCACCATTAAGGCGGGAGGCTGTTGAGTATATGCTAGATGTAGACTACCATATGTTTAGTCTAAGGAGAAGCAAAGCCAACTTCCAACGTATCATGTCACTTCTCAGCGGATTCACCATGGTCTGCAGATGGTTCAATGACATTTGCACCTGGAGAAACCCAATCACTACGTGCCTGGTGCATATTTTGTTTGTGATTCTAGTGTGCTACCCAGAGCTGATCCTGCCAACAATTTTCCTCTACCTCTTCGTGATTGGTTTATGGAACTATAGGTTCAGACCAAGGCACCCACCTCACATGGATGCTCGGATTTCACAGGCAGAGTTTGCACATCCGGATGAATTGGACGAAGAATTTGACAGCTTCCCTACAAGCCGACCCTCTGACATTGTGAGAATGAGGTATGACAGGTTGCGTAGTGTGGCAGGTAGAGTGCAAACTGTGGTTGGAGATTTGGCAACCCAAGGGGAAAGAGCTCAAGCTCTACTAAGCTGGAGAGATTCAAGGGCTACTGCAATCTTCATCATCTTCTCGTTGATCTGGGCAGTTTTCATATACATAACTCCCTTCCAGGTTGTGGCAGTGCTGGTTGGTCTCTATATGCTGCGGCATCCACGATTCCGAAGCAAGATGCCTTCTGCACCTGTTAATTTCTTCAAGAGACTGCCTTCCAAGTCAGATATGTTACTATGA

>Frves32454

ATGAACAACCTCAAGCTAGGGGTGGATGTGGTTAGCGCCCACAATCTTTTGCCTAAAGATGGGCAAGGTTCATCTGATGCATTTGTGGAGCTGTATTTTGATGGCCAGAGGTTCCGCAGCACTATAAAAGAAAAGGATCTCAACCCCGTTTGGAATGAAAGTTTCTACTTCAACATTGCTGATCCTTCAAACCTACACTATCTTACTCTTGAGGCCTATGTCTACAACAATGTGAAAGCTACTCACTCCAGGTCCTTTCTTGGAAAGATCAGTGTCACTGGGAATTCATTTGTCCCCTATTCTGATGCTGTTGTCTTGCATTACCCTCTGGAAAAGCGTGGCATCTTCTCACGTGTAAGAGGAGAGCTCGGCTTGAAGGTTTATGTTACTGATGACCCAACAATTAAGTCCTCCACTCCAATGCCGGCTTCTGAATCCCTTACTGATCAGGATCCAGGCCTTGCACAGACCCAAGGAGTTTCAGCTCCTGGCATGAGCTCATTTCGGAGTGAGAAATCTCAGGCAAGACACACCTTTCATCATCTGCCAAATCCAGGTCAAGAGAGCCAACATCAGCATCATGCTTCTGCTGCCCCAGATACTCATTATGTACCTAAGCATGAAGCTGATCAAATGAAATCTGAACAGCAGCCTGCAAAGCTAGTCCGCATGTACTCTGCTTCAGCATCACAACCTGTTGACTATGCACTTAAAGAGACAAGCCCTTACCTTGGAGGTGGGAGGGTTGTTGGTGGACGTGTTATTCATGGAGACAAGACTGCAAGCACCTATGATCTAGTCGAGAGGATGTACTTTCTCTATGTAAGGGTTGTTAAAGCGCGCGAGCTTCCTGCCATGGATGTTACAGGAAGTCTTGATCCTTTTGTTGAAGCGAGAATTGGAAACTACAGAGGGATTACAAAACACTATGAGAAACAGCAAAATCCAGTATGGAATCAGGTTTTTGCTTTTTCAAAAGATCGCATGCAAGCATCTGTGTTAGAAGTTGTGGTTAAAGACAAGGACCTGCTTAAAGATGACTTTGTGGGAATTGTGAGGTTTGACATCAATGAGGTTCCATTAAGAGTTCCACCTGACAGTCCTCTAGCTCCAGAGTGGTACCGACTTGCGGACAAAAAAGGTGAAAAGATTAAAGGGGAGTTGATGCTTGCAGTCTGGATTGGCACTCAAGCTGATGAGGCTTTTTCTGATGCATGGCACTCTGATGCTGCTACACCTGTTGATAGCTCACCAGCTGCATCTGCAGTGATTCGCTCAAAGGTGTATCATGCACCAAGGCTGTGGTATGTTCGTGTGAATGTGATAGAGGCACAAGACCTCTTTGCAACAGAGAAGAATCGTTTCCCAGATGCATATGTCAAGGTACAGATTGGAAACCAGGTTATGAAGACAAAAACACTTCAGGCTCGAAATTTAAACCCACTTTGGAATGAAGACCTTTTGTTTGTGGCTTCTGAGCCCTTTGAAGACCATCTGGTCATTTCAGTTGAGGACCGTGTTGGCCCTGGCAAGGATGAAATCCTTGGGAGGGTTATATTACCACTCAACTCTGTAGATAGGCGAGCTGATGATCGTATGATCCATTCCAGATGGTTCAATCTTGAAAAGCCAGTTGCTGTGGATGTGGATCAGTTGAAGAAAGAGAAGTTCTCAAGCCGCATTCATCTTCGTGTTTGTCTTGATGGAGGATACCATGTTCTAGATGAGTCGACTCATTATAGTAGTGACTTACGCCCCACAGCAAAACAGCTTTGGAGGCCAGCCATTGGAGTTTTAGAGCTTGGAATCTTGAATGCTGTGGGGCTGCATCCGATGAAAACAAGAGATGGAAGGGGCACTTCAGATACTTACTGTGTAGCAAAATATGGCCATAAATGGGTCAGGACAAGAACCCTTGTCGATAATCTGTGTCCGAAATATAATGAGCAGTACACTTGGGAGGTGTTTGATCCTTCTACAGTTCTTACAGTAGGTGTGTTTGACAACAGCCAGCTTGGGGATAAGGATTCCAATGGCCACAAAGACTTGAAAATTGGAAAGGTTCGTATTCGTATCTCAACACTTGAAGCAGGCCGCATATACACACACTCGTATCCGCTGCTAGTTCTTCACCCAGCTGGTGTCAAGAAGATGGGAGAGTTGCATTTGGCTATTCGGTTTTCATGCACATCTTTTGTGAACATGCTTTACACATACTCAAAACCACTGCTACCAAAGATGCACTACGTAAGGCCCTTCAATGTAATGCAGCTTGACATGCTACGTCACCAGGCTGTGAACATAGTTGCAGCAAGGCTAGGGCGAGCTGAGCCACCACTTAGGAAGGAGGTGGTGGAATACATGTCTGATGTGGACTCACATCTTTGGAGCATGAGACGGAGCAAAGCGAATTTTTTCAGGCTTATGACAGTTTTCTCAGGAGTATTTGCTATAGGCAAATGGTTTACTGACATTTGCATGTGGAAGAATCCCATAACCACTGTGCTTGTCCATGTCCTCTTCCTTATGCTTGTTTTCTTTCCGGAACTGATTCTACCCACAGCTTTTCTGTACATGTTTCTGATTGGGGTATGGAACTTCAGATACCGGCCTAGATATCCTCCTCACATGAACACAAAGATCTCACAAGCTGATCTAGTACACCCTGATGAGCTAGATGAGGAGTTCGACACATTTCCCACTAGCAGGAATCCTGAGCTGGTGAGAATGAGGTATGATCGTCTGAGAAGTGTGGCTGGTAGAATTCAGACAGTGGTTGGTGATGTAGCAACACAAGGTGAGCGGCTTCAGGCACTTTTGAGTTGGCGTGACCCGCGGGCTACAGCACTCTTTGTCACATTTTGCCTCATAGCTGCCTTGGTGATGTATGTGACACCATTCCAGGTGGTGGCAGCATTGGCAGGGTTCTTTATGATGAGGCATCCGCGGTTTCGACACAGGATGCCCTCAGCACCCATCAACTTCTTCCGCAGGTTGCCTGCTAGAACTGATAATCATTGTTGGCCAGAACGCAACTGCCCCAATAAGAAGCAAGAAAGGAATGTCGAAACAGCAAGCTTCCTTCCCAAAATGGCGGTTGCCTTATCGAAAAGAAGTTCCAAACACTTGAAGAAAGAAGCCCCGAAAAGGGAGCCTTGTTTCTGTTTGTCACCATGTTTATGTTTCATTGCTCTATTCAAGTTTCTATATAGACTTCCTGCAGCATTTTTGTTGCTTTTTCTTATCTACCTCTGGTCTTCCTCCACCACCATTATCTCTGGCAACATTGTTCATGTCTGTATCTCGTCTCGGAAGCTCAATAATCATTACTGCCTATCTGCAGGTAGTACAAGACCCAAATTTGAACTCCCAAATCTCAATAATGCTACTCATTACCTCCACACTCCTGACCCGAGTTCAAACCTCTCTATCTCGATATCGCCTCCTTTGTCCCAAAATAACAGTCCCATTAGTCCTCCACCTCCTAAAGTCAGTAGTGAGATTGTGATCAGAGAGACTGTTCAAGTTGTCCATGATGAGTCTGATCCAGTGAAGAATGGAGCCAACAAAGAACGAGAAGAAGAGATTGCAAATGCAAAGAAGCAAGTTGAAGAGCAGCTAAGATTGCACAGATCATGGATATCAAATAAGAAGCATGAAGGGTGTGAAGGAAGAGGAGTCTATGTGTATGACTTGCCATCCAAGTTTAATAAGGACTTGTTGGGTCAATGCAGAGACATGGTTCCATGGATGGATTTCTGCGAGTATTTCAAAAACGAGGCATTGGGTGAATCGATTCCCAAGCTTGGAGATGGGTGGTACCACACTCATCAGTACTCATTGGAGCCAATATTCCATCAAAGGATCTTGAATCATCCTTGCAGGGTTCACAGTGAGAATGAAGCAAAGCTTTTCTATGTTCCATTCTATGGAGGTTTGGACATTCTGAGGTGGCATTTCAAGAATGTGTCCAATGATGTCAAGGACACTTTGAGCTTGGAGCTCATAACATGGCTTAAGAAGCAAGACTTTTGGAGGAAGAATTCAGGTAGAGATCATGTGCTGGTGTTGGGGAAGATTTCATGGGATTTTAGGCGAAAAGATGGTTCGTGGGGGACGAACTTCCTCGAAATTGATCAAATGCAAAATCCAATCAAGCTCTTGATTGAGAGACAACCATGGCAACAAAATGACGTTGGAATCCCACATCCAACATTCTTCCACCCGAATTCAGACGATGATATAATGGCATTGCAATGGAAGATCATTAGCTCATATAGAAAAACGTTAGTGAGCTTCGCTGGTGCAGCAAGGCCTAGTGCAGCCGAGAGCATAAGGTCCACATTGATCGATCAGTGCAACTCAGCCGAAAATGGGAAATGTCACTTCCTCAACTGCCATACAGGTGATTGTGATAAGCCAGAGTCGGTCATTGAGCTTTTCATGGAGTCTGAATTCTGCTTGCAGCCACCAGGAGACAGCCCAACTCGAAAGTCCTTATTTGATTCATTAATATCTGGTTGCATCCCTGTGCTTTTCGATCCTTTCACAGCTTATTATCAGTATCCATGGCATCTCCCAGTGGAACATGGAAAGTATTCTGTGTTTATAGACCAGAAAGATGTGAGGGGAATGAAGGTGAATGTGGTGGAGATGCTTATGAAGATTCCTAAGGAGAACAGGGATGATATGAGGAGGCACATTGTGTACGAGTTGTTGCCTGGTTTGGTTTATGGGGATTCAAATGCCCAGTTTGACAAGTTTCAGGATGCATTTTCGATTACAATGAGTAATCTGATGGAGAGGGTCACCAAATTGGAGTTAGCCACAGATAACTCCGTGCTCGCGCCTCCACGTGTGACCTACTACACCACCTACGTGGAAGCCCCAAGCTCCTCCACGTCAACATCCCAAAACCTTGCATCGCAAATGCTCTGTTCTTCTTCTCGTCTTGTCTTCCGTAGAAACCTCGCCGTCGCTGCAGCTCTTGTTTTCCCCAGGCAATTCTCAACCGTTTCCAAGAATACCCTCTTGCGTCCCTCGTGTTTTCCCTCCATCAAAGCACAGTCTTTCAACCCAGTTTCGTCTTTTCAATTCAATCGATCAATGGCTAGCCAATCCTCCCAACCAAAAACCGTCCACGACTTCACTGTCAAGGATGCCAGGGGCAATGACGTTGATCTGAGCACATACAAAGGAAAGGTCCTTTTGATTGTCAATGTCGCATCACAATGTGGGTTGACCAATTCAAACTACACAGAACTGGCTCAGTTGTACGAGAAGTACAAAACTCAAGGTTTGGAGATTTTGGCATTCCCGTGCAATCAGTTTGGAGCTCAGGAGCCAGGGTCTAATGACGAGATTGTGGAGTTTGCTTGCACTCGCTTTAAGGCCGAGTATCCCATCTTTGACAAGGTGGATGTGAATGGTGACAAAGCCACTCCATTGTATAAGTTCTTGAAGTCGAGCAAAGGTGGTCTCTTTGGTGACAGCATCAAGTGGAACTTCTCCAAGTTCCTTGTTGACAAGGAAGGGAATGTTGTCAACCGTTATGCCCCAACAACTACTCCTCTTAGCATCGAGAAGGATGTGAAGAAGCTGTTGGGGGTTGCATGA

>Gibil34570

ATGAGTAAACTCAAGCTAGCAGTGGAGGTTATTAGTGCCCACAATTTGATGCCCAAGGATGGGCAAGGTTCATCTAGTGCTTATGTGGAAGTAGACTATGGAAACCAGAGATTTCGCACTGTTACAAAAGATAAAGATCTAAATCCTGTCTGGAATGAAAAGCTCGAGTTCAATATACCTGACCCTGCTAATCTTTCCAATCAGACAATAGACATGTATGTTTACAATGAGAATAAGTCTGCTCGCGGAAAGAGTTTCTTGGGAAGAGTCAAGATTTATGGAAGCAGCATTGTTAAACAGGGAGATGCAGTCGTGCAGCATTTCCCACTGGAAAAGAGAGGTATATTCTCACACATCAAGGGAGAACTTGGACTGAAAGTATACTATGTGGAGGAGAAAGGGGGGAACCCCTCTAAAGAGAGCAAAGAAAAGGAGGATAAGTCATCTTCTGTTGAAAAGGAAATGAAGCAACATCAACACCAACAAAAGGCGATGCAGGAGCAGGGTAAATCCTCCGAGGAAAAGAAGCCTGAAATAAAATCAGTGCACATGTATAATACACTTTCTCAGCGAGAAGATTATACTCTGAAGGAGACCAATCCTCAATTGGGAAGTGTAGGGATGAATGGAGACAAGCTGACTAGCGCCTATGATTTGGTTGAGCAAATGCAATACCTTTATGTTCGAGTTGTGAAAGCTAGAGAGCTGCCAGCTAAGGACATTACTGGGAGCTGTGATCCTTATGTTGAGGTAAAAATTGGAAACTATAAGGGAACTACCAAACACTTTGAGAAGAAAACGAATCCGGAATGGAATCAAGTATTTGCATTTTCCAAGGACAGGATCCAGTCGTCTGTTCTGGAGGCAGTGGTGAAGGATAAGGATTTGATCAAGGATGACTTCATTGGGCGTGTGACTTTTGATCTTAATGACGTGCCAACCAGAGTACCTCCTGACAGTCCATTGGCACCTCAATGGTATAGGTTGGAAGACAAGAAATCCGAATCAAAATTGAAAGGTGAGCTTATGCTTTCAGTATGGATGGGCACACAAGCAGATGAAGCCTTTCCAGATGCGTGGAATTCTGATGCTGCTACAGTACACATTGATGGGGTTGCAAATATTCGATCCAAGGTTTACCTCTCTCCCAAGCTATGGTATGTCAGAGTCAATGTGATTGAGGCCCAAGATTTGCAGCCGACTGACAAGGGAAGATTTCCAGAGGTTAGTGTGAAGGTACAGCTAGGAAACCAAATGTTGAAAACAAGAGTAGCACAAAATCGAACCACTAGTCCCCTTTGGAATGAGGATTTGGTATTTGTGGCAGCAGAACCCTTTGAAGATCATTTAATTCTCACAGTTGAGGACCGTTTGGGTCCAAGCAAAGATGAAGTTATGGGGAAGGCTATTATTCCTTTAACATCAGTAGAGAGACGCGTGGATCATAGAACTGTTGACTCACGGTGGTTCAACCTTGAAAAGCATTCTGTGGTTGACGGAGAGAAAAAGAAGGAATCCAAGTTTGCAAGCAAGATTCATTTAAGAGTGTGTCTAGATGGTGGGTATCATGTCCTTGATGAATCTACACATTACAGTAGTGATCTGAGGCCCACTGCCAAACAGTTGCGGAAGCCTTACATCGGCATCCTAGAAGTTGGTATACTAAGTGCCCAGAACTTACGTCCAATGAAGACTAAAGATGGCCGAGGGACTACTGATGGTTACTGTGTTGCCAAATATGGACAGAAGTGGGTTAGGACAAGGACAATCATTGATAGCTACAGTCCAAAGTGGAATGAGCAGTACACATGGGAAGTATATGACCCCTGCACTGTCATCACTGTGGGAGTTTTTGATAATTGCCACCTTCAAGGAGGGATCAGTGAAAAAGCATCGGGAGCTAAGGACACAAGAATTGGCAAGGTACGCATTCGCCTCTCCACTTTGGAGACAGATCGGGTGTACACGCATTCTTATCCTTTGTTGGTTCTACATCCTTCAGGAGTTAAAAAGATGGGTGAACTTCAGTTAGCTGTCCGTTTCTCATGTTCATCTTTTCTGAACATGATGCACATTTATTCAAGACCTTTGCTGCCTAAGATGCATTACTTGCATCCTCTCTCTGTGACTCAGCTCGAAAGCCTTCGGTTCCAAGCCATAAATATAGTCGCAGTGAGGCTCAACCGGGCCGAGCCACCTCTGAGGAAGGAGGTGGTGGACTATATGTCAGATGTGGATTCTAACATGTGGAGTATGCGCCGTAGTAAAGCTAACTTTTTTCGTATTATGTCTGTACTCTCCGGCCTGATAGCCATAGGGAAGTGGTTTGATGAGATTTGCAATTGGAAGAATCCTATTACTACTGTATTAGTCAACATTTTGTTCCTTATACTGGTCTGGTATCCAGAGCTGATGCTGCCAACTGTGTTCCTGTATATGTTTCTGATTGGCATATGGAATTACCGTTTCCGGCCTAGTTATCCACCACACATGGATACTCGCCTCTCTAATGCAGAGGCAGTGCATCCAGATGAACTTGATGAGGAGTTTGATACCTTTCCTACTTCAAGAGGTGCTGATATTGTGAAGATGCGGTATGATAGGTTGAGGAGTGTTGCAGGGAGGATACAGACTGTTGTTGGAGATGTAGCCACGCAGGGGGAGAGGCTGCAGGCTCTCTTAAGTTGGAGAGATCCCCGTGCTACTACAATTTTTATCACTTTTTGCTTGATGGCTGCCATAATTTTATATATCACTCCCTTCCAAGTTGTGGCCGTGCTTGTAGGACTGTATCAAATGCGTCATCCAAAATTCCGGTACCGACTTCCTTCTGTTCCCCTGAATTTCTTCAGGAGGTTGCCTGCACGAACTGATAGTATGCTGTAG

>Gibil33902

ATGGGGAAGGTGATGAAACTGGTAGTTGAGGTGGTGGACGCCCACAATCTGATGCCCAAGGATGGTCAGGGGTCGGCCAGCCCTTTTGTAGAGGTAGATTTTGACAATCAGAGGCGTCGAACCCATACTATAGAGAAAGACCTGAATCCCATTTGGAATGAGAAACTGGAGTTCCATGTGAACGACCCTGCAAAACTACAAAACCAGACGGTCGAAGTTTATGTGTACAATGAGAAGAAGTCTAACCACAGTAGAACCTTTTTGGGTAAAGTTGGAATTTATGGAAGCAGCATTGTGAAGCAAGGCGAAGAGGTTCTCCAACGCTTTCCGTTGGAGAAGAGGAGCATTTTTTCCTATATAAAGGGAGAGATTGGGTTGAAGATTTACTATTTGGAGGAGGAGGAGAAGGGTAAGGGTGGAGGTGGAGGTGGAGGAGGAATAGGAGGTGGCGGCGGTGGTGGTGGAGATGGAACTCCATCTATTGATGGGAAGGGGAAGGAGGAGAGCAAGGCCTCAGAGGCGAAGACGGAAACAAAGCAGCATATACATAAACAACAGACATCACAGCAGCCAGGGAAATCTGTTGAGGAGAAACGGGCAGAACCGAAGGTGGTGACAATGCACAATGCTCCTTCCCAACCCGTAGATTATACTTTGAAAGAAACCTCTCCACATTTGGGGGATGGAAGGGTCAGGGGAGGTGTATGGGGCAGTGGTGAGAGGGTTACTAGCACATATGATTTGGTGGAGCAGATGCAGTATCTCTATGTTAGAGTGGTGAAGGCTAGAGATCTTCCAGCCAAGGAGATCACTGGGAGTTGTGACCCTTACGTGGAGGTTAAACTTGGGAACTACAAGGGCACTACCAAGCATTTTGAGAAGAAGACTAATCCTGAATGGAACCAGGTCTTTGCATTTTCGAAGGAAAGGATCCAGTCCTCGTTTGTTGAGGTAGTGGTGAAGGATAAGGATTTTGTCAAGGATGACTTCATTGGGCATGTCACTTTTGACCTCAATGACGTGCCCACTCGAGTGCCTCCAGACAGTCCATTGGCACCTCAATGGTATAGGTTGGAAGATAGGAAAGGGGAGGCCAAGGTTAAAGGTGAGCTTATGTTGGCAGTATGGATGGGTACGCAGGCTGATGAAGCATTTCCAGAAGCTTGGCATTCCGATGCATCCTCAGTAGACAGTGATGGATTAGCAAATATTCGATCCAAGGTATATGTGTCTCCCAAGCTGTGGTATGTGAGAGTCAATGTGATTGAAGCTCAGGATTTGCTGCCAAGTCACAAGGGACGGCATCCTGAGGTCAGTGTAAAGGTGCAGCTTGGAACCCAAATGTTGAAGACAAGGATAGCTCAGAATCGAACCACAAGCCCCCTCTGGAACGAGGATTTGGTTTTTGTAGCAGCAGAGCCCTTTGAAGAGCATTTGATCCTCATGGTGGAGGACCGGGTTGGTCCAAATAAGGATGAAATTTTGGGGAAGACGATCATAAACTTGACAATGGTTGAGAGGCGTGTGGATCACAGAACTGTCAACTCCCGGTGGTTCAACCTTGAAAAGCCCAATGCTGTTGAGGGGGAGAAGAAGAAGGAGATTAAGTTTGCAAGCAGGATTCATTTAAGATTGTGTCTTGATGGTGGATATCATGTCCTTGACGAATCTACACTTTATAGCAGCGATCTGAGGCCCACTGCCAAGCAGTTGTGGAAGCCTTCTATTGGTGTCTTGGAGGTTGGCATACTGAGTGCCCAAACCTTGCTTCCAATGAAGACAAAAGATGGGCGGGGGGCTACCGATGCCTACTGTGTTGCCAAATTTGGTCAGAAATGGGTTAGAACAAGGACTATTATTGACAGCTTCAATCCAAAGTGGAACGAGCAGTATACCTGGGAAGTTTTTGACCCATGCACTGTCATAACAGTGGGAGTTTTTGATAATTGCCATCTTCAAGGAGGAGCCAATGAAAAGGCTAAGGATGCGAGGATTGGTAAGGTACGCATTCGCCTCTCCACTCTGGAGACTGATCGGGTGTACACCCACTCTTATCCTCTGCTGGTGCTGCATTCTTCGGGGGTTAAAAAGATGGGGGAGCTTCATCTGGCTGTTCGTTTCAGCTGTTCATCTTTGGTCAACATGATGCACATGTACACACATCCTTTGCTGCCTAAGATGCATTACCTACACCCTCTCTCTGTAAGTCAACTCGAAAGTCTTAGGTACCAAGCCATGAATATAGTATCGATGAGGCTCAACAGGGCTGAGCCTACTTTAAGAAAAGAGGTAGTGGAGTACATGTTAGATGTGGATTCTCACATGTGGAGCATGCGCCGCAGTAAAGCCAACTTTTTCCGCATAATGTCCGTTCTGTCTGGACTGATAGCAGTCAGCAAATGGTCTGATGAGATATGCCACTGGAAGAATCCTATCACTTCTGTGCTGGTGCATGTTTTGTTCCTTATTTTGGTTTGGTATCCAGAGTTGATACTGCCAACTGTTTTCCTCTATATGTTCTTGATTGGTATATGGCATTTCCGTTTCCGGCCCCGCCTTCCACCCCACATGGATACCCGCCTCTCTCATGCAGAGGCAGTGCATCCTGATGAACTTGATGAAGAATTTGATACATTCCCTACTTCAAAACCTCCTGAGATTGTGAGAATGCGTTATGATCGGTTAAGACATGTTGCGGGGCGGATACAGACTGTAGTTGGAGACATAGCCACCCAGGGAGAAAGGTTACAGGCTCTCTTGAGTTGGAGAGATCCTCGTGCTACGACTGTATTTATTATGTTTTGCTTGTTTGCTGCTATACTTCTGTATGTCACTCCATTCCGAGTGGTGGCAGTGTTCACAGGTCTGTATATATTGCGCCATCCAAAATTTCGGAGCAAGCTTCCTTCCGTTCCTATCAATTTCTTCAGAAGGTTGCCTGCACGAACTGATAGTATGTTGTGA

>Gibil25152

ATGAGTAAGCAGAAGCTTGCTGTAGAGGTGGTGAATGCGAGCAATCTGATGCCCAAAGACGGGCAGGGATCCGCGAGCGCGTTTGTGGAGGTGGATTATGACAGCCAGAGGAATCGAACCCGTACCAGAGAGAAGGATCTTAACCCGTCTTGGAACGAGAAACTGGTATTTTCTGTTAATGATCCTAAAAGCCTTTCAAATCAGACTGTTGATGTTTATGTTTATAATGAGAAGAAAGGGGGTCATGGTAGGAATTTTTTGGGGAAGGTTAGGATTTCTGGTAGCAGTGTTGTGAAGCAGGGTGAGGAGACTTTACAGAGGTTTCCTTTGGAGAAAAGGAGTTTATTTTCGCATATCAAGGGTGAGATTGGGTTAAAGATTTACTATTTTAATGAGAAAGATGGAGGTGGAGGTGGAGGTGGTAAGCAGTCTGGTGAGGGTGGTAAACCCTCTGGTGAGGGTAAGGTTAAAGAGGGTAAAGAAGGGAAGGAGGGAAAGGAGGGGAAGGAAGGAAAGGAGGGGAAGGGGAAGGAAGAGAAGAAATCCTCAGATGAGACGAAGGAAGCACCTCAAATTACAACTACAACTACCACTGTGCAACACCAAAAACAGACAGCTCAACCACAGGGGAAATCGGTAGAGGAGAAGAAACCCGAGGTTAAGGCTGTACACATGCATGCTCATAACCAGCCCGTAGATTATGCATTGAAGGAGACCACTCCTCACCTTGGTGGTGGAGGGATTAATAGGGATAAAAGAACTAGCACTTATGACCTGGTAGAGCAGATGCAGTATTTGTATGTCCGTGTTGTGAAAGCCAGGGATCTCCCTGCTATGGATATAACCGGCAGCTGTGATCCCTATGTTGAGGTGAAGGTGGGGAATTACAAGGGTACCACAAAGCATTTTGAAAAGAAGACAAATCCAGAATGGAATCAGGTTTTCACTTTCTCGAAGGATCGGCTCCAGTCTTCGGTGTTGGAGGTGGTGGTGAAGGATAAGGATTTTGTTAAGGATGATTTTATTGGGCGTCTGTTTTTTGATATGCATGATTTGCCCGTTCGAATGCCTCCTGATAGTCCTTTGGCACCTCAGTGGTATAGGCTAGAAGACAAGAAAGGGGAAGCAAAGGCTAAAGGTGAATTGATGTTGGCAGTGTGGATGGGCACACAGGCAGATGAAGCTTTTCCTGAAGCATGGCATTCTGATGCAGCTGCAGTGCATTCGGATGGATTAGCTAATACCCGATCCAAGGTTTATGTTTCTCCCAAACTGTGGTATGTTAGGGTGAATGTAATTGAAGCTCAGGATTTGATGCCAAGTGACAAAGGTAGGATTCCCGAGGTCAGTGTTAAAGTGCATCTTGGAAATCAATTGTTGAAGACAAAAACAGTACCGAGCAGAACTATGAGTCCGCTTTGGAATGAGGATTTGGTTTTTGTAGCAGCAGATCCTTTTGAAGATCACTTAATCCTCACGGTTGAGGATCGGTTGGGTCCAAATAAGGATGAAGTTTTGGGGAAAACAATAATTCCTTTGTTCATGGTGGAGAAGCGATTGGATCATAGAATTGTGAACTCTCGGTGGTTTAACCTTGAAAAGCCTACTGCTGTTGAGGGGGAGAAGAAGAAGGAAGTTAAGTTTGCAAGTAGGATTCATTTGAGAGTGTGTTTGGATGGTGGCTATCATGTCCTCGATGAATCGACACATTACAGCAGTGATTTAAGGCCCACTGCTAAGCAGTTATGGAAGCCTTTTATTGGTATCTTGGAGGTTGGTATATTGAGTGCACATAGCTTGCTTCCTATGAAGACCAAAGATGGGCGAGGGACTAGTGATGCCTACTGCGTTGCAAAATATGGACAGAAATGGGTCAGGACGAGGACAGTAATTGATAGCTTAAGTCCAAAGTGGAATGAGCAATACACATGGGAAGTTTATGATCCTTGTACAGTTATCACTGTAGGGGTTTTTGATAATTGCCACCTTCAAGGAGGGGCTGCTGAAAAGGCATCGGGAGCTAAGGATGCAAGGATTGGTAAGGTACGCATTCGCCTGTCCACTCTAGAGACGGATCGTGTTTACACCCACTCTTATCCTCTCTTGGTGCTGCATTCTTCAGGAGTAAAAAAGATGGGTGAGCTTCAGCTGGCTGTTCGTTTCTCGTGTTCATATTTGATTAACATGATGCACATTTATACACAGCCTCTGCTGCCCAAGATGCACTACTTACATCCACTGTCTGTAACTCAGCTAGATTTCCTTAGGCACCACGCAATGCATATAGTGGCTATGAGACTCAACCGAGCTGAGCCTCCTCTAAGAAAAGAGGTTGTGGAATATATGTTAGATGTGGACTCTCACATGTGGAGTATGCGTCGCAGCAAAGCTAACTTTTTCCGCATAATGTCTGTACTCTCTGGCCTGATGGCAGTAGGCAAGTGGTTTAATGATATATGCATGTGGAAGAATCCTGTGACAACTGTGCTAGTTCACATTTTGTTCCTTATTCTGGTCTGGTATCCAGAATTGATATTGCCAACTGTCTTCTTGTACATGTTCTTAATTGGCATATGGCATTTTCGTTTTCGACCCCGACATCCACCCCATATGGACACTCGTCTTTCCCATGCAGAGGCTGTGCATCCTGATGAACTTGATGAAGAATTTGACTCATTTCCTACTTCAAAAGGTCCTGATATTGTGAGGATGCGATATGATCGTCTGAGGAGTGTTGCCGGGAGGATACAGACAGTGGTTGGAGATATGGCCACTCAAGGAGAAAGGCTTCAGGCCCTTTTGAGTTGGAGAGATCCACGTGCTACTACTATATTTGTCACTTTTTGCTTGATGGCAGCCATAATTCTGTATGTCACCCCTTTCCAAGTAATTGCAGTGCTTTATGGAATGTATATCTTGCGTCATCCACGGTTTCGAAACAAGCTTCCTTCTGTTCCTCTCAATTACTTCAGGAGATTGCCTGCACGGACTGATAGGATGCATTGTACAGTAGGTGTTTCCTCTCTGTCAATTAATTGCTTTTGGTCAAGTCATGGGGATCTTGTTTGTGGTGTCATTGTACCACCTAAAGAGCTAGAGAAGAACCAATGCTAG

>Gibil22114

ATGACGACGAGAAAATTGATTGTAGAAGTAGTTGATGCGCACAGTCTGATGCCCAAGGATGGCGTCGGAACTTCGAGCCCCTATGTTCTTGTAGATTTCGATGGTCAGAGGAAAAGGACGAAGACAAAGCATAAGGACTTGAATCCTGTTTGGAATGAAAAATTGGAGTTTACTGTGAGCGATCCCAAGACGATGGCCGCAGAAGAGCTGGAAGCTGATGTTTATAATGATAAAAGAACTGGAAATGGGCGGCGGAGTCATTTCTTGGGTCGAGTTAGGATCGGTGGCAGCCAATTCGTGAAGAAGGGCGAGGAAGCTTTAATTTATTTTCCGTTAGGGAAGAGAAATCTATTTTCTTTGATTAAGGGTGAGCTTGGGCTGAGAATATATTATGTAGACGAGCCCATCCCACCACCGGAGGAGGAGCAGCCAGCAGAGCCCTCAACAGAAGCAGCTCAGCCTGTAGAAGCAGCGCCTGCATCAGAAGAAGCAGCTGCTCCACCTGCCGATGCACCGGCAGCCGAAGCTGCTCCTCCTGCAGAAGCGCCGCCCACAGAGGAACCACCCGCCGATGCACCCCCTGCGGAAGCTGCACCTGCTGCAGAAGAGCCGCCCGCAGACGCTCCACCAACGACTGAGGCCGCACCGGCCGATGCAGCGCCACCCGCTGAAGCTCCCTTAGCGGAAACGCCACCTGCCGAAGCTACTGCTGCGGAAGCGGCTCCCTCAGATGCACCACCAGTCGAAGAAAAACCTTCGGAAGAAACCCCTCCTGCAGGGGAAACTACTCAGCCAGATGAGCAGAAGCCGGCTGACGAGGCTCCTCCAGCCGAAGCAAAACCTCCTGACCAAGAAAAACCAGCCGACGAGCCCATACTTGTGCAGAACCCGCCCATGGCGTCGGTTTCTGGCATGCCAGCTGATTACGTGATGAAGGACCTGAGCGGCTCCTTTGAAAGCAACAATGAACGATCGGCCTATGATTTGGTGCAGAAGATGCAGTATTTGTTTGTCAGAATTGTAAAAGCCAGAGCGCTGCCTCCCCGGGATTCAACCACGGGATCGCCAGCGGATCCCTTCGTGAAGATCAGAGTAGGAGGAGAGTCAGTTCGAACGCGTACATCCCCCAAAACGGGTTACCCGGAATGGGACAAGGTATTTGCATTTGGTCAAGATAAGCTTCAATCTGCTCCCACTTTGGAAATTTCAGTTTGGGATGGCGACACAGGAAGCAGAGATGATTTTCTGGGCGGTGTATGCTTTGATCTATCGGAGGTCCCAACTCGGGTGCCTCCTGACAGTCCTTTGGCACCTCAATGGTACAGATTGGAAGGCGAAGGCAGAGTTAAAGGCGACATAATGCTCGCTGTCTGGATTGGAACGCAGGCCGACGAGGCCTTTCCGGTGGCCTGGCAATCTGATGCAGGCAGCGTCATACATACGAGGTCCAAAGTCTATCTGTCGCCCAAGCTATGGTATCTGAGGGTCAATATAATTGAAGCCCAAGACCTGCAGATTCCAGAAAATGTACGTTATCCTGAGCTGAGAGCGAGAGTTCAATTGGGTTTCCAGGTATTGCGAACGAGGCCTGCTAACAACCGAAACAGCAGCCCGTTTTGGAACGAGGATCTCCTTTTTGTGGCAGCTGAACCATTCGAAGAGCAGTTGTTTTTTATTATCGAGGAGCGGTCGACCAAGGAGGCAGGGCTTGTGGGTCATGCCAGAGTCGCACTTTCGTCGATCGAGAGAAGAGTGGATGATCGTATGGTGAGTTCCAGATGGTTTAATCTGGACAGAACTCATGGGGATCCGAACGATAGATACCACGGGAGAATTCACCTCAGAGTTTGTCTTGATGGTGGTTATCATGTCATGGACGAGGCTGCTCATTTGAGTAGTGATCTACGCCCCACAGCCAAGCAGCTTTGGAAACCATCGCTGGGGGTGCTGGAGCTTGGCATTCTCGGTGCCCAGAATCTGCTCCCCATGAAAACCAAAAACGGCAGAGGGGCAACCGATGCATATTGCGTAGCCAAATATGGCCAGAAGTGGGTTCGAACCCGTACCATCACAGATAGTTATAATCCAAGGTGGAATGAGCAATACACATGGGAAGTCTACGATCCCTGCACGGTTCTCACCTTGGGTGTCTTTGATAACTGGCATATATTCAACACTGTGGACGAAAAAGCAGCCAAGCCCGACTACCGCATTGGCAAGGTTCGAATCCGGATATCCACACTTGAAAGCAATAAGGTCTACACAAATTCATATCCTCTCCTGGTTTTGATGCCCTCCGGAGTCAAAAAAATGGGCGAAATCGAGCTTGCCGTTCGGTTCACCTGCCCATCTCTGCTAGACGTTTTGCACGTCTACACGCAACCTATGCTGCCCAAAATGCATTATCTTCACCCCTTGGGACTCCACCAGCAGGACTTGTTGAGAAACACTGCCATGGAAATTGTGGCCAGGAGATTATCTCGCTCAGAGCCACCTCTGAGAAAAGAAGTAGTGCAATACATGCTGGACACCGATTCCAACATGTGGAGTATGCGGCGAAGCAAGGCTAACTGGTTTCGTATCATGAACGTGCTCTCTGGTGTCGTCAATGTTACAAAATGGGTCGATGATATTTGTCTATGGAAGAATCCGGTTACCACAGTACTGGTTCACATTCTCTTTCTTATACTCGTATGGTACCCGGAACTGATAGTTCCCACTGTGTTTTTCTACGTATTTCTGATAGGCTCCTGGTACTACAGATTTCGGCCGCGGATGCCTCCGCATATGGACACTGGTATTTCCCATGCAGATGCAGTTGATCCAGACGAGCTGGACGAGGAATTCGACCCCATCCCCAGTCAGAAACCCCCGGAGGTTGTCAGATTCAGATATGACAGACTTAGAAGCTTGGCTGGACGCATTCAGAGTGTTTTGGGCGATTTTGCCACACAGGGAGAACGGATTCAGGCTCTGCTGAGCTGGAGAGATCCTCGTGCAACGGGGATATTCATTGCTGTTTGCTTTATGATTTCGCTTATCTTATACATTGTTCATTTCAAGATGGTTTCAGTGGTACTCGGATTCTATTTCCTTCGCCACCCCAGGTTCAGAGGACCAATGCCGGCTTCGTCTCTCAACTTCTTCAGACGTTTGCCAGCTCTCTCTGACCGTCTTTTGTAA

>Gibil08022

ATGAGTAAAAGGAAGCTTGTGGTTGAGGTGGTCGATGCACACAACCTTATGCCAAAAGATGGACAAGGTTCGGCTAGTCCCTTTGTGGAAGTAGACTTTGAGAACCAGAGGTTTAGGACTCATTCCAAACAGAAAGACCTTAACCCTGTATGGAATGAGAAGCTGACATTCAATGTGACCGATCCTGCAAATCTTGCCAACCAGAATGTGGAAGTTTTTGTCTACAATGAGAAGTCGTCCAGTCATCAGAGAAACTTTTTGGGGAAGGTGAAGATTTCTGGTACAAGCATTGTCAGGCAGGGCGAAGAGGTTTTACAGAGGTTTGCTTTAGATAAGAGAAGTATTTTCTCGCATATTAAGGGCGAGGTTGGCTTGAAGATTTATTATTTTGATGAGAAGGGTGGAAATCCTTCAGGTGGTGAAAAGAAGGCAAAGCAGGAAAAGAAATCCTCTGAGGAGAACAACAAGGAAGCCAAGCAACAGCAGCACTCACAACAGAGCGCCCAACAACAGCGGAAACCTGTGGAGCAGAAAAAGGCTGAAGTGAAAGCGGTTGCAATGCATACTGCCCCTTCCCAACCAGTAGATTACTCTTTGAAAGAAACCAACCCTCATCTGGGTGGCGGCGGTGTGAACAGGGACAAGACCACTAGCACTTATGATTTGGTAGAACAAATGCTGTATCTTTATGTTCGTGTGGTGAAGGCCAGAGAGCTTCCTGCCAAGGACATGACCGGAAGCTGTGACCCTTATGTGGAGGTGAAGCTTGGGAATTACAAGGGAACCACCAAGCACTTTGAGAAGAAATCGAACCCTGAGTGGAATCAAGTCTTTGCTTTCTCCAAGGAAAGGATTCAATCTTCGTATCTTGAGGCAGTAGTGAAGGATAAGTCTTTCGTGAAGGATGATTTTATAGGGAGAGTGACATTTGATCTCAATGAAGTGCCCACCAGAGTCCCTCCTGACAGTCCCTTGGCACCTCAGTGGTATAGGTTAGAAGACAAGAAGGGAGAGTCTAAGGTGAAAGGTGAGTTGATGCTGTCGGTGTGGATGGGCACACAGGCAGATGAAGCTTTTCCAGAGGCCTGGCATTCGGATGCTGCAACTGTGCATAGTGATGGCTTGCCCAATATTCGATCCAAGGTTTATCTTTCTCCCAAGCTGTGGTATGTCAGGGTGAATGTAATTGAAGCACAGGATTTGCAGCTAAGCGACAAAGGAAGGTTTCCTGAGGTCAGCGTTAAGGTGCAGCTGGGAAACCAAGTGCTGAAGACAAGAATAGCTCAGAATAGGACCACAAGTCCTCTCTGGAATGAGGACTTGCTTTTTGTGGTTGCAGAGCCTTTTGAAGATCATTTAATGCTCATGGTGGAGGACAGGTTGGGGCCCAGTAAGGAGGAAATCATGGGGAAGACAATAATTCCTTTGACAACAGTTGAGAGGCGTGCTGATCACAGAACTGTCAACTCCCGTTGGTTCAACCTCGAAAAGCCAAATGCCTCAGAAGGAGAGAAGAAGAAGGAAGTCAAGTTTGCAAGCAGAATTCACTTAAGAGTGTGTCTTGATGGTGGGTATCATGTTCTCGATGAATCTACACTTTACAGCAGTGATTTGCGGCCCACTGCCAAGCAGTTATGGAAGCCTTATATTGGTGTACTGGAGGTTGGTATTTTGAGTGCCCAAAACTTGCTTCCAATGAAGACAAAAGATGGAAGAGGGGCAACTGATACATACTGCGTTGCTAAATATGGTCAGAAGTGGGTTAGAACCAGAACAATCATTGACAGCTTCAATCCGAAGTGGAATGAGCAATACACTTGGGAGGTCTATGATCCCTGCACTGTCATAACCGTTGGAGTTTTTGATAATTGGCAAATTCGAGGAGCAGGAGCTGAAAAGGGATTGGGCGGTGGTGCCAAGGATACAAAGATTGGGAAGGTGCGCATTCGCTTGTCAACACTGGAGACAGATCGGGTTTACACGCACTCTTACCCTCTGTTGGTGCTGCATTCTTCGGGAGTTAAAAAGATGGGGGAGCTTCAGCTGGCAGTTCGTTTCTCATGTTCATCTTTGGTCAACATGATGCATATTTACGCACAACCTTTGCTGCCCAAGATGCATTACTTACATCCTCTTTCTGTAACTCAGCTCGAAAGTTTAAGGTACCAAGCCATGAATATAGTGGCAATGAGGCTGAGCAGAGCCGAGCCGCCCCTTAGAAAAGAGGTAGTGGAGTATATGTTAGATTTGGATTCTCACATGTGGAGCATGCGCCGCAGTAAAGCCAATTTTTTCCGCATAATGTCTGTATTGTCTGGACTGATAGCTGTTGGCAAGTGGTTTGATGAGATATGCAATTGGAAGAATCCTGTCACTACTGTTCTGGTCCATATCCTGTTCCTCATTCTTGTCTGGTATCCAGAGTTGATACTGCCAACAATTTTTCTGTATATGTTCTTGATTGGCATTTGGCATTTCCGTTTCCGGCCCCGACATCCACCGCACATGGATACCCGCCTCTCTCATGCAGAAGCAGTTCATCCGGATGAACTTGATGAAGAATTTGATACATTTCCTACCTCCAGGGGTGCCGATATTGTGAGGATGCGCTATGATCGGTTAAGAAGTGTTGCAGGGAGAATACAGACTGTGGTGGGTGACTTGGCCACTCAGGGAGAAAGGCTGCAGGCTCTCTTGAGTTGGAGAGACCCTCGTGCCACCACTATATTTATTACGTTTTGCCTACTTACTGCCATACTTTTATATGTCACCCCTTTCCAAGTAGTGGCAGTGCTCACTGGTGTGTACATGTTGCGGCATCCCAGATTTCGCAACAAACTTCCTTCCGTTCCTCTCAATTTCTTTAGGAGGTTGCCTGCACGAACTGATAGCATGCTGTAA

>Gibil38760

ATGACAACAAGAAAGGTGATTGTAGAGGTGATGGATGCACGCAACCTCATGCCGAAGGATGGAGAGGGGTCCTCAAGTCCCTTTGTAACGGTGGATTTTGATGGTCAGCGCAAGCGCACAAAGACTAAGCATAAAGATCTGAATCCAGTATGGAATGAGAAACTGGAGTTCACTGTGAGTGATCCAAAGCTAATGATACGTGAGGAGCTTGAAGCAGAGGTTTGCAATGATAAGAAGACTAGTAATGGTAGGCGCAGCCATTCTTTGGGAAGGATAAAGCTACTTGGGAGCCAGTTTCCTAAGATGGGTGAGGAAACATTGATTCATTATCCTTTGCAGACCAAGAATTTTTTTTCTTTAATTAAGGGAGATCTGGGGCTCAGAATATATTACTTTGATGAGCCCATTAAGCCCCCCGATCCTCCACCAGCAGAGGCTGCACCTGCTGAAGCCCCTCCTGCTGCTGATCCCCCTGCTGAAACCCCGCCTGCTGAAGTGCCCCCTGCAGAAGCACCTGCCGAATCTCCTCCAGCAGAAGCACCTGCTGCTGAAGCCCCTCCAGCTGAAGCCCCACCTGCTGAAGCCCCTCCTGCAGAAACTCCACCTGCTGATTCCCCTCCTGCTGAAGCACCACCTGCAGAAGCAGCACTGGCGGATGCGGAACCAGCTGAAGCCCCAGCCGCTGAAACCCCAGTTTCAGAAGCACCACCAGCTGAAGAGGTAGCTGCACCCACTGGAGAAACACCATTACCAGCAGAAGAAAAGCCCGCTGAGGTGGCCAGTGCCCCTGAAAGTACGCCTCCTGAACAAGAAAAAGTGACAGCAGCAATGGAAGAAACACTACATCGTCTTCAGAGACCGACCATGGCCCCAGCATCAGCTATGTCTGCAGAGTATCTCATGAAGGATTTAAGCAGTTCTTTTGCCGGAAACAATGAGCGATCAGCATATGATTTGGTGGAGAAAATGCCATATCTTTTTGTGAGGATTGTCAAGGCAAGAGCTCTTCCATCTAGAGATTCGTCAGGGAATGCAGCAGATCCCTTTGTGAAGATCCAAGTAGGAGGCGAACTTCTTCGAACACGAACAACCCAAAAGACTGCATACCCAGAATGGAACCAAATATTTGCCTTTGGCAAAGACAAACTTCAATCCGCTCCCACACTGGAGATTTCTGTATGGGTTGACGACACTGGTACCAAGGATGATTTCCTTGGAGGAGTCTGTTTTGATCTCTCAGAAGTTCCAACAAGGGTCCCTCCTGATAGCCCTTTAGCACCCCAATGGTACAGATTGGAAGGAGATGACAGAGTCAAAGGGGACATTATGCTCGCAGTTTGGATGGGAACACAGGCAGATGAGACATTCCCAGAGGCTTGGCAGTCGGATGCAGGAGGTGTAATAAACACCAGATCAAAAGTTTATTTATCTCCAAAACTGTGGTATCTAAGGGTCAACGTAATTGAAGCTCAGGATTTGCAGATTCCAGAAAAAATAAAATATCCTGAACTCAAAGTGAAAGTCGAATTAGGTAGGCAGGTTTTGAGGACTAGGACTTCTAATGCAAGAACATCAAGCCCATTTTGGAATGAGGATTTGATATTTGTTGCAGCTGAACCCTTTGAGGAACAGTTGGTTTTGTTGGTTGAGGATCGGCCTATTAATAGTAGTAATAAAGATGAAGAAGCTTTAGGCCAAGCCAGAGTTGCACTGTCAACAATCGAAAGAAGAGTAGACGACCGTATGGTAAGTTCGAGGTGGTTCAATTTGGAAAAAGTAAATGGAGAACAGAATGATAGGTACCATGGAAGAATTCATCTGCGCTTGTGTTTTGACGGAGGTTATCATGTTATGGATGAGGCTGCTCATTTGAGTAGTGATTTAAGACCAACAGCTAAACAGCTATGGAAACCATCTTTAGGTGTGTTGGAGTTGGGAATTTTGGGAGCCCAAAACATGTTGCCTATGAAAACCAAAGGAGGAAGAGGTTCAACAGATGCATATTGTGTAGCAAAGTATGGACAGAAATGGGTCAGAACAAGAACTATTACTGATAGCTTTAATCCAAGATGGAATGAACAATATAGATGGGAGGTTTATGACCCATGCACTGTTCTAACCTTAGGAGTCTTTGACAATAGACACATGTTCATGAACAATGCTGATCAGAAATCAGCAGAGCTCAAGGATGTTTGCATTGGAAAAGTTCGTATTCGCATTTCGACACTCGAAAGCAATCGAGTTTACACAAATTCTTATCCGTTGTTGGTTCTCCTGCGTCCTGGTGTGAAAAGGATGGGAGAAATTGAGCTTGCAGTGAGATTTTCCTGCCCTTCATTGCTTGATGTTGTTCAGATCTACTCTCAACCACTGCTTCCTAAGATGCATTATCTCCATCCGTTGGGCCTAAGTCAGCAGGACATGTTAAGGAACACTGCAATGAAGATTGTTGGTTTAAGATTGTCTCGTTCCGAACCACCTTTGAGGCAGGAAGTGGTGCAATATATGTTGGACACAGACTCTAACATGTGGAGCATGAGGAGAAGTAAGGCCAATTGGTTCAGAATTATGAATGTTCTCTCTGGTGCTGTATCTGTTGCCAAATGGATTGATGATATTTGTCACTGGAAGAATCCCATCACCACAGTGTTGGTGCATATCGTGTTCCTTATCCTGATTTGGTACCCTGAACTGATTGTGCCAACAGTTTTCTTCTATGTTTTTGTCATTGGTGCATGGCACTATAGGTTTAGGCCTAGGATGCCAGCTCACATGGATACACGAATTTCCCATGCAGATGCAGTTGATCCAGATGAACTTGATGAAGAATTTGATCCAATACAAAGCTCTAAGCCTTCTGAACTGGTCAGGGTGAGATATGATAGATTGAGGAGTTTGGCTGCAAGGATTCAGACAGTGTTGGGTGATTGTGCTGCACAGGGCGAACGAATTCAAGCTCTGCTTAGCTGGAGAGATCCTCGTTCAACTGGAATTTTCATTGCTACATGTTTCATGATCTCTTTTATCTTGTATATTGTCCATTTCAAGATGGTTTTGATAATCCTTGCATTCTACTTTCTTCGTCACCCAAGATTCAGAGAGCCAATGCCTGCTTCCCTTCTCAATTTCTTTAGACGTTTGCCTGCTCTTTCAGACCGCATCATGTAA

>Gibil30312

ATGCAGAGGCCTGCTCAACCAGAAGACTACAGTCTGAAAGAAACATCCCCACATCTGGGAGATGGAAGGGTCAGGGGAGGAGGATGGGGTAGCAGTGAGAGAACCACCAGCACATATGACTTGGTGGAACAGATGAAGTATTTGTATGTCAGAGTGGTGAAAGCCAAAGATCTTCCAGCCAAGGACATAACAGGGAGCTGTGACCCGTATGTGGAGGTGAAGCTTGGGAACTACAAGGGCACCACCAAGCATTTTGAGAAGAAGACCAATCCTGAATGGAACCAGGTCTTTGCATTCTCCAAGGAGAGGATCCAGTCTTCCTATGTTGAAGCAGTGGTGAAAGACAAGGATTTTGTCAAGGATGACTTCATTGGGCGCGTGGCTTTTGATCTCAGCGAGGTTCCCACCAGGGTGCCGCCTGATAGTCCATTGGCACCTCAATGGTATAGGTTGGAGGACAGGAAAGGGGAGTCCAAGGTTAAGGGCGAGCTCATGTTGGCAGTATGGTTAGGTACGCAGGCGGATGAGGCATTTCCTGAGGCGTGGCATTCTGATGCAGCTACTGTACATCCTGACGGCTTAGCGAATATCCGATCCAAGGTATACCTTTCCCCTAAGCTATGGTATGTTAGAGTTAATGTCATTGAAGCGCAGGATTTGCTACCGAGTGACAAGGGTCGTTACCCTGAGGTTAGTGTTAGGGTGCACTTGGGTAACCAGACGTTAAAAACCAAGTTTGCCCAGACCCGGAATACCAGTCCACTCTGGAATGAGGATTTGATGTTTGTGGCAGCAGAGCCCTTTGAGGAGCATTTGGTCCTCACGGTAGAAGATCGCGTAGGTCCTAACAAGGAGGAGGTTCTGGGCAAGTGCTCAATTAATTTGAACACCGTTCCAAGGCGTTTGGATTATAAGACTGTCACTTCTAAGTGGTTTAATCTTGAAAAACCCACTGCTGTGGAGGGAGAGGCCAAGAAGAAGGAAATCAAGTTTGCTAGTAGGATTCATTTGAGAGTTTGCCTTGATGGTGGGTATCACGTTCTGGATGAATCTACACATTATAGCAGTGACCTGAGACCCACCGCCAAACAATTATGGAAACCTTATATTGGTGTATTGGAGGTTGGTATACTGAGTGCCCATAGCTTGCTTCCAATGAAGACTAAAGACGGGCGTGGGACTACCGATGCCTACTGCGTTGCCAAATATGGGCAGAAGTGGGTTAGGACTAGGACAATTATTGACAGCTTCAATCCAAAGTGGAATGAACAGTACACCTGGGAAGTCTATGATCCCTGCACTGTTATAACAGTGGGAGTTTTTGACAATTGCCACCTTCAAGGAGGGGTCAATGAGAAGGCAGCAGGGGCTAAGGATGCAAGGATTGGGAAGGTACGTATACGTCTCTCTACTCTCGAGACAGATCGGGTTTACACCCACTCTTATCCTCTTTTGGTGCTGCATTCTTCTGGGGTCAAAAAGATGGGTGAGCTTCAACTAGCCGTCCGTTTTACTTGCTCATCTTTAATGAACATGATGCACATCTACACTCATCCGTTGCTGCCTAAGATGCATTACTTACATCCCCTGTCAGTAGGTCAGCTAGAAACTCTTAGGTATCAAGCCATGAACATAGTGGCAATGAGGCTGAGCAGGGCTGAGCCTCCTCTGAGAAAAGAGGTAGTGGAGTATATGATAGATGTGGATTCTCACATGTGGAGCATGCGCCGCAGTAAGGCCAACTTTTTCCGCATAATGTCTGTACTGTCTGGACTGATAGCAGTTGGCAGGTGGTTTGATGAGATATGCAATTGGAAGAATCCTGTGACAACTGTGCTAGTGCATATTCTGTTCCTCATTCTGGTATGGTATCCAGAGTTAATACTGCCCACTGTCTTCTTGTACATGTTCTTGATTGGCATATGGCATTTCCGTTGGCGGCCTCGACACCCACCTCACATGGACACTCGCCTCTCTCATGCAGAGGCAGTGCATCCCGATGAACTCGATGAAGAATTTGATACATTTCCGACTTCAAAAGGGGCTGATATTGTCAGGATGCGATATGATCGGTTGAGGAGTGTTGCAGGTAGGATACAGACTGTTGTTGGAGACTTAGCAACTCAGGGGGAAAGATTTCAGGCCCTCCTGAGTTGGAGAGATCCCCGTGCTACTACCATATTTATCACTTTTTGTTTGTTTGCTGCTATTGTTCTGTACGTCACCCCTTTCCGAGTAATGGCAGTGCTTGCGGGTTTCTATATATTGCGCCATCCAAGATTTCGGTACAGACTTCCTTCTGTTCCCCTTAATTTCTTCAGGAGGTTACCCGCACGAACAGATTGTATGTTGTAA

>Gibil20033

ATGACTACCACTCGTAAGCTCATAGTTGAGGTGTGCAATGCCCGTAATTTGATGCCCAAAGACGGCCAGGGGACTTCCAATCCATATGTTTTGGTAGAATTTGATGGGCAGAGGAGCAGAACAAGTACCAAGGCAAAGGATTTGAATCCACAATGGGATGAAAAATTGGAGTTTGTGGTGAATAATCCAGAGCTTATGTCTGCACAGCTGCTTGCAGTTAATGTCTCTAATGATAATAACAATGGTGGAAGGAGCAATTTCCTTGGGAGATTGAAGGTTTGTGGCAGTAGCTTTGTTAAGCAAGGAGAAGAGGCTCTTGTATATTACCCATTGGAGAAGAGAAGCATTTTTTCTCATATTAAGGGAGAGATTGGGCTGAAAATATATTATTTGGATGAGGAGGAGACTAAGGTTGATGAACCAGAGAAGAAGCCCAAGGAAGTGGATTTGAGTCCACAAAAGGAGGAGGTTCAATCCCCACCAGCAGAAGAAAAAAAGGCTGAAGAGAAGGATAAACCCAAGGGAGATGATTCTCCAATAGAGAATAAATCAACAGAGGACCAATTGCCACCAATGGAATCAGACAAGAGTGTGAACATGGAGGAATCAAAAATAGAAGTTTCCGATCCCCTACCAAATATTAGTCCTAGCCCACCTCTGGATTTTTCAATCAAGGATTTTAGCAGTTCCATTGGAGCCATAAATGGTGATAGGATCAGCAGATATGATCTAGTTGAGCAGATGCAATATCTTTATGTTAGAGTAATCAGAGCCAGGGAGCTGGCTGCCAAGGACATGAATGGAAGCTCAGATCGTTATGTTATGCTCACAATTGGTAGCCATAAGGTTCGAACCCGGGCAGTCAGGAACAACCTGAATCCTGAATGGGATGAGGTGTTTGCAATTGGGATAGACAAGCTGAATGCATCCACATTAGAGGTCACAGTTTGGGATGAAGATGTGGGAAAGAAGGATGATTTCCTCGGTACAGTGTCCTTTGACCTACAGGAAGTTCCTACCAGGGTGCCTCCTGACAGTCCCTTGGCTCCTCAATGGTACAGACTTGAAGAGACCTCCGGTACCATGTTTGGAGATGATGGAAGAAATGTCAAGGGTGACATTATGCTTGCAGTTTGGTTGGGCACACAGGCTGATGAAGCTTTTTCACAAGCATGGCAATCTGACACAGGGGGTCTGATGCTTCACACTCGTTCCAAAGTTTATTTGTCTCCTAAACTATGGTATTTGAGGGTCAATATTATTGAGGCTCATGACTTGCGACCCATTGAAAAAATAATTTATTCTGAAGTCAGTGTGAAGGCTCAATTGGGATTTCAAGTGTTTAAGACTAGATTTTGTAAGGGCACTGCAACTCCAATCTGGAATGAAGATTTGGTGTTTGTGTGTGCAGAGCCCTTTGAGGAATCCTTGGTTTTGAGTGTTGAACAGAGAACGAATGGATCCAACAAGGAGGAAATTATCATAGGGGAGACTATGATTTTTCTTAGCAAAGTTGAGAAGAGAGTTGACCATAGGATAGTGGGTTCTGTCTGGTTCAATTTGGAGAAAAAAGATAAGGATGGGGATTCTAAGGAGAAATTGTACAATGGAAGAATTCATGTTCGTTTGTGCTTTGATGGTGGGTACCATGTATCAGATGAGGCTGCCCATGTTAGCAGTGATGTGAAACCCACTGCAAAACAACTATGGAAGCCCCCATTAGGAGTATTGGAGATTGGCATCATAAGTGCCAAGAATTTAATGCCAATGAAAACAAAGGATGGGCGTGGCTCCACTGATGCATTTTGTGTGGCTAAGTATGGGCAAAAGTGGGTTAGAACCAGAACAGTATTGGATAGCTTCAATCCCAAATGGAATGAGCAGTACACTTGGGAAGTCCATGATCATTGCACAGTCTTCACCTTGGGTGTTTTTGATAATTCCCAACTCAATGCCAATGAAGACGTCAAGAGTTCGAATCCCAACCCACCCAAAGACCAACGTATTGGGAAGGTGAGAATCAGGATATCTACATTGGAGTCTGAACGGATTTACACTAACTCGTATCCTCTCCTGCTGTTACAGCCTTCCGGGGTTAAAAAAATGGGAGAAATAGAGCTTGCAGTTCGATTCTCATGCTCTTCATTGACGGACATATTGCATGTTTATTCCCTGCCAATGCTTCCCAAAATGCATTACCTGCACCCCTTTGGAATAGGACAACAGGACCTACTCAGGCACGCTGCTATGAAGATTGTGGTGTCCAGATTAGGTCGTTCTGAGCCACCTCTGAGCCAAGATGTGGTGCAATACATCCTTGACACTGAGGCAAATCTATGGAGCATGAGACGTAGCAAGGCCAATTGGTACAGACTTGTTAATGTATTGTCTGGATTAATGGCAGTTTCTAAATGGGTTGAGGATATTTGCCAATGGAAAAATCCAACAACAACAATGTTGGTTCAAGCATTGTTCCTCATACTTGTCTGGTATCCAGAGCTGATTCTGCCCACTGTGTTTGTCTACATGTCTTTGATTGGTGCATGGCGGTACAGGTGGAGGCCACGTATGCCTCCACATATGGATACACTTCTGTCCCATGCAGATGGTGGAAGTGTAGACTCTGATGAATTAAATGAAGAATTTGATACATTCCCAACATCATGTAGTCCAGAGATCATGAGGATGAGATATGATAGATTGAGGTCTGTTGCAAGTAGAATTCAAACGGTTTTAGGTGACTTGGCCACCCAAGGGGAAAGATTTCAGGCCTTGCTGAGCTGGAGGGATCCTCGTGCAACTGCTGGTTTTTTAATATTTTGCTTGGTTGCTTCTCTAGTTTTGTATGCGACTCCATTCCGTGTGATAGCAGTTCTGTTTGGTTTCTACTTTCTTCGCCATCCAAGGTACAGAGATCCACTCCCTTCGGCTCCTCTTAACTTCTTCAGGAGATTGCCAGCCCTGTCAGATCGTATGTTGTAA

>Glyma01G219200

ATGAACATTTCGTGTCGACATGTCAACTTTCATCTGTTCCTGTGTCTCCTCTTGGTCACCCATCATTCTGACCCTTCTAATCTTCACTATCTCACACTTGATGCCTATGTCCACTGCCATACCAAAGCTACTAACTCCACTTCATTTCTTGGCAAGGTTAGTCTTACCGGGACTTCTTTTGTCCCTTACTCTGATGCAATTGTCTTGCACTATCCATTGGAGAAACGTGGTATTTTCTCTCGTGTAAGAGGAGAGATTGGCCTTAAAGTTTACATCACTAATGATCCAAACATAAAATCATCCATTCCAACTCCTGCTGTTGAGTCCATGCCAACAAATAATTCAAGCTCAACACATGCAGAAGTTCGAGCACCTGCAAGCACAATGACAAACAATTTTCCAAATGAAAAGGTTGATTCAAGACACACATTTCATCATCTCCCTAACACAAGCCATCACCAGCACCAACAGCATTCCTCTGGCTTTGCAGATACTCATTATGTAACAAAGTATGAAGCCGATGCAATGAAATCTGAACCACAACCTATGAAGCTAGTACGCACTGCTACATCAGTACAACCAGTTGATTTTGCACTTAAAGAAACAAGTCCCTATCTTGGTGGGGGAAGAGTTGTTGGCGGCCGTGTTGTTCATAAAGACAAGACTGCTAGTACATATGATCTTGTGGAAAGGATGTACTTTCTGTATGTCAGGGTAGTCAAGGCTCGTGAGCTTCCTGCTATGGATGTTACTGGTAGCCTTGATCCATTTGTTGAGGTGAGAATTGGCAATTACAAAGGTATCACTAGACACTTTGATAAGAATCAGAGTCCTGAATGGAATCAGGTGTTTGCATTTTCAAAGGACCGGATGCAAGCATCTGTACTTGATGTTGTGATCAAGGACAAGGATCTCATCAAAGATGATTTTGTAGGCATTGTGAGATTTGACATCAATGAGGTTCCTCTGAGAGTACCACCAGACAGCCCTTTGGCACCAGAGTGGTATCGCCTTGAGGACAAGAAAGGGGAGAAGATTAAAGGTGAGTTGATGCTTGCTGTTTGGATTGGCACTCAAGCAGATGAGGCTTTTTCTGATGCATGGCATTCAGATGCAGCCACACCTGTTGACAGCACACATGCTATCTCTGCAGTGATGCGTTCAAAGGTCTATCATGCACCGAGGCTATGGTATGTGCGTGTCAATGTTGTGGAGGCACAAGACTTGGTTCCTACAGAGAAAAACCGTTTCCCAGATGTGTATGCCAAGGTGCAGATAGGAAACCAAGTGCTGAAAACAAAGACAGTTCCAGCCCGAACACTAAGCGCTCTCTGGAATGAAGATCTTTTGTTTGTTGCTGCTGAACCTTTTGAAGATCATTTAACTATTTCAGTTGAAGATCGGGTTAGTCCTGGAAAAGATGAGGTCATTGGGAGGATCATCATACCACTGAACTCCGTGGAAAGGCGTGCAGATGATAGAATCATTCATTCTAGATGGTTCAACCTGGAAAAGCTAGTTGCTATAGATGTTGATCAGTTGAAGAAAGAGAAGTTCTCAAGCAGAATTCAGCTCCGGTTATGTCTAGATGGAGGATATCATGTTCTTGATGAGTCAACTCATTACAGCAGCGATCTCCGCCCCACGGCAAAGCAGCTATGGAAGCCACCAATTGGGGTTCTAGAACTTGGAGTGCTGAATGCTGTAGGACTCCACCCAATGAAAACAAGGGATGGTAGGGGCACATCTGACACATACTGTGTAGCAAAATATGGTCACAAATGGGTCAGAACAAGGACCATTGCTGATAATCTGTGTCCAAAATACAACGAGCAATACACTTGGGAGGTTTTTGATCATGCCACGGTTCTTACTGTTGGTGTCTTTGACAACAGCCAGCTCGGGGAAAAGGGTAATGGTTCTTCAAAGGACTTGAAAATTGGGAAGGTCAGAATTCGCATCTCTACACTGGAAACAGGAAGGATTTACACTCACTCATATCCATTGTTGGTTCTTCACCCAACCGGGGTTAAGAAGATGGGTGAACTTCACTTGGCAATACGGTTTTCATGCACTTCTTTAGCAAACATGCTTTACCTATACTCACGACCACTGCTGCCAAAAATGCACTACGTAAGGCCTTTCTCTGTGACGCAGCTAGACATGCTGCGTCACCAAGCAATGAACATAGTGGCGGCTAGACTCGGCCGAGCAGAGCCACCACTTCGCAAGGAAGTGGTGGAATACATGTCAGATGTGGACTCTCACCTTTGGAGCATGCGGCGAAGCAAGGCGAATTTCTTCCGAGTGATGAGCGTCTTCTCTGGTGTGTTTGCTGTTGGAAAATGGTTTGGTGATATCTGCATGTGGAGGAATCCTATCACAACAGCGCTGGTTCATGTGTTGTTTCTCATGCTTGTTTGCTTCCCGGAATTGATCTTGCCCACTGTTTTTCTCTACATGTTCTTGATAGGGGTATGGAACTTTAGATATAGACCAAGGTACCCTCCTCACATGAACACAAGAATCTCACAGGCTGAGGCAGTGCACCCGGATGAGTTAGATGAAGAGTTTGACACTTTCCCTACTAACCGGAGCCCGGACCTAGTGAGAATGAGGTATGATCGTTTGAGGAGTGTGGCTGGAAGGATTCAAACAGTGGTTGGTGATTTGGCCAGCCAGGGAGAGAGGATTCAGGCACTGTTAAGCTGGAGAGACCCGCGTGCTACATCCATATTTATTACATTGTGTCTCCTATCTGCGTTGGTGCTCTATGTTACTCCTTTTCAAGCAGTGGCTGGTTTGGCAGGGTTTTACATTATGAGGCATCCCAGATTTCGCCACAGGCTGCCATGTACCCCAGTCAACTTCTTCCGGCGCCTCCCTGCAAGAACAGATTGTATGCTATAA

>Glyma02G176700

ATGAGTTCTTCTCAAGCAGCACCAAAGGGAAACCAAGAGGACTACAAGCTGAAGGACACAAAGCCGGAGCTTGGAGAAAAGTGGCCACATGGAGGGCAACGTGGAGGGAGTGGTTGGATATACAACGAGAGAGCCACAAGCACCTATGACTTGGTGGAGCAAATGTTCTACCTCTATGTCCGTGTTGTGAAAGCCAAGGATCTTCCACCAAACCCTGTCACCAGCAACGTGGACCCTTATGTTGAAGTGAAGGTTGGCAACTACAAGGGGAAAACAAGGCACTTTGAGAAGAAGACAAGCCCTGAGTGGAAGCAGGTTTTTGCATTCTCAAAGGAGAAGATTCAATCCTCAGTTGTTGAGGTTTTTGTGAGGGACAAAGAGATGGTGGCTAGAGATGATTACATTGGGAAAGTGGAGTTTGACATACATGAGGTGCCAACAAGGGTGCCCCCAGATAGCCCTTTGGCTCCTCAATGGTATAGGCTTGAGAATTTGAGGGGTGAAGCAAGAAGTAGAGGGGAGATCATGCTTGCTGTTTGGATGGGGACACAAGCTGATGAAGCATTTCCTGAGGCTTGGCATTCAGATTCTGCTTCAGTTAAGGGAGATGGGGTTTATAACATAAGGTCAAAGGTTTATGTTAACCCAAAATTGTGGTATTTGAGGGTTAATGTGATTGAGGCTCAAGATGTGGAGCCAAATGACAAAAGCCAGCCACCCCAAGTTTTTGTGAAGGGTCAAGTTGGACAACAAGTGCTCAAGACTAAGTTGTGTCCAACAAAAACACCAAACCCAATGTGGAATGAGGATTTGGTGTTTGTGGCAGCAGAGCCCTTTGAGGAGAAGCTTGTGCTCACTGTGGAGAACAAGGCCTCCCCTGGGAAGGATGAGGTTGCGGCTAGAATAAGCTTGCCATTGAACAAGTTCGAGATTCTATTGGATCACCGAGCCGTGCACTCGCATTGGTACAACCTTGAGAGGTTTGGGTTTGGTGTGTTAGAGGGTGACAAGAGAAATGAGTCAAAATTCTCAAGTAGGATTCACCTAAGGGTGTGTCTTGAAGGTGCTTATCATGTCCTGGATGAGTCCACAATGTATATTAGTGACACAAGGCCAACTGCTAGGCAACTTTGGAAGCAACCAATTGGAATTCTTGAAGTGGGGATATTGAGTGCTCAAGGGCTCCAATCTATGAAGACAAACAATGGTAAAGGGTCAACAGATGCTTATTGTGTGGCCAAGTATGGTCAGAAATGGGTGAGAACTAGGACAATCACTGAGAGCTTTAACCCAAAGTGGAATGAGCAATACACATGGGAAGTGTATGATCCTTGCACTGTGATAACCTTTGGGGTCTTTGACAACTGCCATTTGGGTGGTGGTGGAGGGCAAACTCAAGTAGCCAAAGTTGACTCAAAAATTGGCAAGGTGAGGATTCGTTTGTCAACATTGGAAATGGATAGGATCTACACCAACTCATACCCTCTACTTGTTCTGAAAACCTCTGGATTGAAGAAGATGGGGGAACTTCAATTGGCGATTCGTTTCACATGTCTCTCCATGGCTCACATAATCTACCTCTATGGACACCCTTTGTTGCCAAAAATGCATTACCTACATCCATTCACTGTGAACCAATTGGACAGTTTAAGGTACCAAGCTATGAACATTGTGGTGGTTAGGCTTGGGAGAGCAGAACCACCCCTTAGGAAAGAGGTTGTGGAGTACATGCTTGATGTGGACTCTCACATATGGAGCATGAGAAGAAGCAAAGCCAATTTCTTCAGAATTGTGTCACTCTTTTCTGGTGCAATATCTATGAGCAAGTGGCTTGGTGAGGTGCAACAGTGGAAGAATCCAGTGACCACAATCCTAGTGCATGTCCTCTTTTTCATCTTGATATGTTACCCTGAACTGATCCTCCCCACAATGTTCCTCTACATGTTTCTCATTGGAATATGGAACTTTAGGTTTAGGCCAAGGCACCCTCCACACATGGACACCAAACTTTCTTGGGCAGAAGCAGCACACCCAGATGAACTTGATGAGGAGTTTGATACTTTTCCCACCTCAAAGGCTCAAGATGTGATCAGAATGAGGTATGATAGGCTTAGGAGTGTGGCTGGGAGGATACAAACTGTGGTTGGAGATATTGCAACACAAGGTGAGAGGTTTCATGCATTGCTTAGTTGGAGAGACCCTAGAGCCACAAGCCTATTTGTGATTTTTTGCCTTGTTGTTGCTGTGGCATTGTATGTGACACCTTTCAAGGTTGTGGCTTCAGTTGCTGGCATTTTCTGGCTCAGGCACCCCAGGTTCAGAAGCAAGCTACCCTCGATGCCTAGTAATTTCTTCAAGAGGTTGCCATCTTGTGTTGATGGCATGCTTTGA

>Glyma03G012600

ATGAGCAATCTCAAGCTAGGTGTGGAAGTTGTGGGTGCTCATGACCTTATGCCCAAAGATGGACAGGGCTCATGTAGTACTTATGTGGAACTTCACTTTGGTGGTCAGAAATTTGGGACTACAACTAAAGAGAAAGATTTGAATCCTGTTTGGAATGAGAAATTTTACTTCAATGTTACTGATCCAAGCAAATTGCAGAATCTCACTCTTGATGCTTGCATCTACCACTATAGCAAGAGTAATAATTCCAAAGTCTTCCTGGGTAAGGTTCACCTTACTGGACCCTCATTTGTACCTTATGCCGATGCTGTTGTTTTGCACTACCCTCTGGAAAAGAAAAATGTTTTTTCCCGCATTAAAGGAGAGCTTGGTTTGAAGGTATATGTCACTGATGATCCTTCGATAAAATCCTCAAACCCTCTTCATGATGTGGAACCCTCTGCGCACACAGTCCAACCCTCAACCCCAGATCAATCACCAGTTTCATTCACAAATTCAATCCTGAACGTCTTTTCTCGCAAGAAAAATGAGACAAAGCACACATTTCATACCCTTCCCAATTCAAATGAAGAAAAACAGCATAAATCTTCTTCTTCTTCAGCTGCTGCAAAGACAACTAAAGACTCAGGGATGCATGAGACTAAATCTGGAATGCCTCCTCCAAAAGTTTTGCATGCGTATCCAGGTTTGTCCTCCCCAATGGATTATGCACTCAAAGAGACAAGCCCTTTTCTTGGAGGGGGACAAGTAGTTGGTGGGCGAGTTATTCGCGGGTACAGGCCATCCAGCTCCTATGACCTTGTTGAACCAATGCAATACCTATTTGTACGAGTTGTAAGAGCTCGTTTGGCTGGGAGCATTGATCCATATGTGGAGGTAAAGGTTGGAAATTTCAAAGGAATTACCAAACACTATGAGAAAACTCAAGATCCTGAATGGAATCAAGTGTTTGCCTTTGCAAGGGAAAATCAGCAGTCAACTTTGCTTGAAGTTGCGGTCAAAGACAAGAATATATTACTAGATGAAGTTATTGGCACTGTGAAGTTTGATCTCCACGACGTTCCTACACGTGTTCCACCTAATAGTCCATTGGCTCCTGAGTGGTATAGGATTGACAAGGGTAAGGACAAGAAAAAAGGGGAGCTGATGCTCGCTGTATGGTTTGGCACACAAGCCGATGAAGCTTTTCCTGATGCTTGGCATTCTGATGCTCTCTCCTCTGGTGATATCTCCTCAGCTGCATATGCTCATATGAGATCAAAAGTTTACCATTCACCAAGATTATGGTACGTACGGGTTAAAGTTATCGAGGCTCAAGACTTACATGTGTCAGAGAATTCCCAAATCCATGATGCCTATGTTAAGCTACAGATTGGTAACCAGATTTTGAAGACAAGACCAGTTCAATCAAGGACTATGATTCTGCGTTGGGATCAAGAGCTGATGTTTGTTGCTGCTGAACCCTTCGAGGAACCTCTGATTGTTTCAGTGGAAAATCGAGTTGGTCCCAACAAAGATGAGACTATTGGAGCTGTTGTTATTCCTTTAAACCAAACTGATAAGCGAGCTGATGATAGACTTATCCTTACTAGGTGGTATCACCTTGAAGAGTCCATGCCATCTGCGATGGATGGGGAACAAGGAAAAAAGGAGAAAGATAAATTTTTTAGTAGAATTCACCTTAGTGTCTGTCTTGATGGTGGTTACCATGTTTTTGATGGGTCAACTTATTATAGTAGTGATCTCAGACCAACGTCAAAGCAGCTCTGGAAGAAGTCAATTGGTCATTTAGAAATTGGCATTCTAAGTGTCGATGGACTTCATCCGACGAAAACAAGGGATGGAAGGGGGATAACAGATACATACTGTGTGGCAAAATATGGGCATAAATGGGTTCGCACTCGAACCATTAGTGACAGTCTTAGTCCAAAATACAATGAGCAGTACACTTGGGACGTTTATGATCCTGCTACAGTTCTCACTGTGGCGGTGTTTGATAATGGACAACTTCAAAATTCAGATGGTAACAAAGATCTAAAAATTGGTAAAGTTCGGATCCGGATCTCAACCCTGGAAGCTGGTCGTGTTTACACAAACGCTTACCCATTACTAGTCCTACATCCTTCAGGTGTCAAGAAGATGGGTGAGTTGCACTTGGCCATTAGATTCTCCTGTTCCTCAATGGTTGACTTGATGCAACAGTATTTCAAGCCTCACTTGCCAAAAATGCACTATAAGAGGCCACTTAACTTAATGGAACAGGAAAAGCTGCGACACCAAGCGGTCAATGTTGTTGCTGCTCGACTTAGTAGGGCGGAACCCCCGCTTAGAAAGGAGGTAGTTGAATACATGTGTGACACAGATTCTCATCTCTGGAGTATGAGGCGCAGCAAGGCGAACTTCTACCGTCTGATGACAGTGTTTTCTGGAATTCTCTCGGTGGTAAGATGGTTAGGGGAAGTTTCCACATGGAAGCATCCTATAACAACAGTGCTGGTGCACATTCTTTTTCTGATGCTTGTGTGTTTCCCTGAACTTATACTGCCAACCGTGTTTCTATACATGTTTGTCATCGGCATGTGGAATTGGAGGTTCCGGCCAAGGTGCCCTCCTCACATGAACATCAGACTCTCCTATGCAGAAAGGGTGACCCCGGATGAGCTTGACGAGGAATTTGACACATTCCCCACCTCAAAGAGCCCCGACATTCTACGTTGGAGGTATGATCGGTTAAGAAGTGTGGCTGGGAGGATCCAGAGTGTTGTTGGAGATTTAGCTACTCAAGGAGAGAGGATCCAAGCTCTTGTGAACTGGCGTGATCCTCGTGCCACTGCCATGTTCATGGTATTTTGCTTCGTGGCTGCTATTGCGTTGTATGTCACACCCTTCCAGCTGCCTATTCTTCTGACTGGATTCTACTTGATGAGGCACCCCATGCTTCGAAGCAAGGTGCCGCCTGCTCCGGTTAATTTCTTCCGCAGGTTGCCTTCTCTCACAGACAGCATGCTGTAA

>Glyma03G142500

ATGCAGAGGCCTCCACCAGAAGATTTTCTGTTGAAGGAGACCAAACCCCACCTTGGAGGGGGAAAAGTCTCTGGCGACAAGCTCACTAGCACTTATGACCTAGTCGAGCAAATGCAGTATCTCTATGTGAGGGTTGTGAAAGCTAAGGACTTACCTGCAAAGGATGTCACTGGCAGTTGTGACCCTTATACTGAAGTCAAGCTGGGGAACTACAAAGGCACCACTCGGCACTTCGACAAGAAGTCTAATCCCGAATGGAACCAGGTTTTTGCTTTCTCCAAAGACCGCATTCAGGCTTCCATACTGGAGGTCACTGTGAAGGACAAGGATGTCGTGAAAGATGACTTCATTGGTCGTGTCTTATTTGACCTCAATGAGATCCCAAAGCGTGTTCCACCAGACAGTCCCTTGGCACCGCAGTGGTATAGGCTGGAGGACAGAAAGGGTGACAAGGCGAAGGGGGAGCTGATGCTGGCTGTTTGGATGGGTACACAAGCTGATGAGGCGTTTCCCGAAGCGTGGCACTCGGATGCTGCAACCGTTAGCGGGACTGATGCTCTTGCAAACATTAGATCAAAGGTATATCTATCTCCTAAGCTTTGGTATTTGAGGGTTAATATAATAGAGGCGCAAGACTTGCAGCCAAGTGATAAGGGTAGATACCCTGAAGTTTTTGTGAAGGCTGCTCTGGGAAATCAGACCTTGAGGACTAGAATCTCTCAGAGCAGGACTATCAATCCAATGTGGAATGAGGATTTGATGTTTGTGGCTGCTGAGCCGTTTGAAGAGCCTCTGACTCTGAGTGTGGAAGACAGAGTTGCACCTAACAAAGAGGAGTCGCTGGGGAAGTGTGCAATTCCTTTGCAGATGGTGGATCGGAGACTGGATCAAAAGCCTGTGAATACTAAGTGGTATAATATTGAAAAGTACATTGTTATTATGGAAGGGGAAAAGAAGAAGGAAATCAAGTTTTCAAGCAAGATTCATATGAGGATCTGTCTGGAAGGCGGTTATCATGTTTTGGACGAATCAACTCACTACAGCAGTGATCTTCGTCCAACTGCGAAGCAGCTGTGGAAGTCCAGTATTGGAGTCCTTGAATTGGGTATATTGAATGCTCAGGGTTTGATGCCAATGAAAACAAAAGATGGTAAGGGGACTACTGATGCTTATTGTGTAGCGAAATATGGGCAGAAATGGGTGCGAACAAGGACAATCATTGATAGCTTTGCACCCCGGTGGAATGAACAGTATACTTGGGAGGTTTTTGATCCTTGCACTGTCATTACAATTGGTGTATTTGATAACTGTCATTTGCATGGTGGTGATAAGCCTGGAGGGGCAAAAGATTCCAAAATTGGGAAGGTAAGAATTCGTCTTTCTACCCTCGAGACTGACCGTGTCTATACACATTCCTATCCACTTCTAGTTCTTCATCCAAATGGTGTGAAGAAAATGGGTGAAATCCACTTGGCTGTTAGGTTTACTTGTTCTTCTTTGCTTAATATGATGCACATGTATTCACTACCTTTGTTGCCAAAGATGCATTATATTCACCCACTAACTGTCAGCCAGCTTGATAATCTGAGGCATCAAGCTACTCAGATTGTTTCAATGAGACTGAGTCGTGCCGAGCCACCGCTGAGAAAGGAGATAGTGGAATATATGCTAGATGTGGGTTCCCACATGTGGAGTATGAGAAGAAGCAAGGCGAACTTTTTCAGGATTATGGGAGTTTTGGGTGGATTAATTGCTGTAGGAAAATGGTTTGACCAGATTTGCAACTGGAAAAATCCAATCACAACAGTTCTGATCCATATCCTGTTCATAATATTGGTCATGTACCCTGAGCTTATCTTACCAACAATCTTCCTTTACCTCTTCTTGATCGGAGTTTGGTACTACAGATGGAGGCCAAGGCACCCACCTCACATGGACACTCGCCTCTCCCATGCAGATTCTGCGCATCCTGATGAACTGGATGAAGAGTTTGACACATTCCCAACCACCAGGCCTTCAGACATCGTGAGGATGCGATACGACCGACTTAGAAGCATTGCTGGGAGGATACAGACTGTGGTTGGTGATTTGGCCACTCAAGGGGAGAGGCTGCAGTCTTTGCTCAGCTGGAGAGATCCAAGAGCCACTGCCCTCTTTGTGATTTTCTGTCTGGTTGCTGCCATTGTACTCTATGTCACTCCTTTCCAAATTGTCGCACTTTTCACTGGAATCTATGTGTTGAGACACCCGAGGTTCCGCCACAAGCTTCCTTCGGTGCCTCTGAATTTCTTCAGGAGGCTGCCTGCAAGAACTGATTGCATGCTTTGA

>Glyma05G029600

ATGATGACGACGCCGTTTCAGCAACCGCCCCAAACCGTACGGAGACTGGTCGTGGAAGTGGTGGACGCGCGCAACCTGCTCCCCAAGGATGGGCAAGGCAGCTCCAGCCCTTACGTGGTGGCGGACTTCGACGGCCAGAGGAAGCGAACCACGACCCGGTTCAAGGAGCTGAACCCGGTCTGGAACGAGCCTTTGGAGTTCATCGTCTCCGACCCAGAGAACATGGAGTTCGAGGAGCTCGAAGTGGAGGTGTACAATGACAAGAAGTTCGGCAACGGAAGCGGCCGCAAGAACCACTTCCTCGGGAGGGTTAAGCTCTACGGAACTCAGTTTTCAAGAAGAGGCGAAGAAGCTCTTGTTTACTACACGCTCGAGAAGAGAAGCGTGTTCAGTTGGATAAGAGGAGAAATTGGCCTCAGGATTTATTACTACGACGAGATGCTTATGGAAGAGGAGAAACCGCCGCCGCCGCCGCAGCAGCAGCAGGAGGAGCAAGGTGAGAGAACGGAACAAGACCGGAACAAGCCGCCGCCGGGGGTGGTGGTTGTCGAGGAGGGACGGGTTTTCGAGGCTCCTGGAGCGATGGAGCAATGCGTGCCTCTTCCTTCAGGTCCTCCTCATTCGCCGCGTGTTGTGGTCGTGGCTGAATCTCCGCCGCCGGTGGTTCATGTTTCGCAGGATCCGCCGTTGGCGGAAATGTGTGAGCCTCCGGCGTCGGAAATGCAGTTCCACCCCGAGGTGAGGAAAATGCAGGCCAACAGAGGGAATAGGGTGAAGATTTTGAAGCGGCCGAACGGTGATTACTTGCCTAAAGATATTTCTGGAAAGAAAACTGGTAATGAGTCTGAGCGAGTTCATCCCTTCGATCTGGTGGAGCCGATGCAGTACCTGTTTGTGAAAATCTGGAAGGCCCGTGGGCTCGCTCCTCCCAGTGAAGGCCCAATCGTGAGGGTTAGGATGTCGAGCCAATCCAGGAGATCCAATCCCGCAAGTTACAGGCCCAGCGAGCCCCCCGATTCGCCGGAATGGAACCAGACATTCGCGCTGAGTTACAACAACACGAACGATGCAAACAGCGCCACACTGGAGATTTCGGTTTGGGATTCCCCGACAGAGAATTTCCTCGGTGGCGTTTGCTTCGACCTCTCCGACGTTCCGGTGAGAGATCCTCCGGACAGTCCTTTGGCGCCGCAATGGTACCGCCTCGAAGGAGGAACCGCCGATCAGAATCCCGGGAGAGTCTCCGGTGACATTCAGCTTTCCGTGTGGATCGGGACGCAATCCGACGACGCGTTCCCGGAAGCGTGGATCTCAGACGCACCCTACGTGGCTCACACTCGATCCAAGGTTTATCAATCGCCAAAGCTCTGGTACCTGCGCGTGACGGTGGTGGAAGCGCAAGACCTGAATATTGCTCCGAATCTGCCTCCACTGACAGCGCCGGAAGTGAGGGTGAAGGTGGAATTAGGGTTCCAATCGCAGCGAACAAGGAGAGGATCCATGAACCACCGCAGCTTATCGTTCCACTGGAACGAGGACCTTCTATTCGTAGCCGGTGAGCCACTGGAAGATTCTGTCATAGTTCTGTTGGAAGATCGAACCACCAAGGAACCGGCGCTGCTAGGCCACATCGTGATTCCTCTGAGCTCAATCGAGCAGCGAATCGACGAGCGCCACGTGGCAGCAAAATGGTTCACCTTAGAGGGCGGGCCCTACTGCGGCAGAGTCCAGATGCGCCTCTGCCTGGAGGGAGGGTACCACGTGCTGGACGAAGCGGCGCACGTGTGCAGCGACTTCCGTCCCACGGCGAAACAGTTATGGAAGCCGGCGGTGGGGATTCTAGAGCTTGGAATTCTTGGCGCTCGGGGGCTGCTACCGATGAAATCCAAAGGCGGTGGAAAGGGGTCCACGGATGCTTACTGCGTGGCAAAGTATGGGAAGAAGTGGGTCCGGACCCGAACCGTAACCGATACCTTCGATCCACGGTGGAACGAGCAGTACACGTGGCAGGTATACGACCCCTGCACGGTCCTCACCGTGGGAGTGTTCGACAACTGGCGCATGTTCGCTGACGTGTCGGAGGACCACAGGCCCGATTGCCGCATCGGCAAGGTACGCATACGCGTGTCTACGCTGGAGAGCAACAGAATATACACTAACTCCTACCCCCTCCTCGTCCTCACGCGAACCGGCTTGAAAAAAATGGGCGAAATCGAGTTAGCGGTTCGCTTCGCCTGCCCCTCCCTGCTGCCCGACACGTGTGCGGTGTACGCCCAGCCCCTGCTTCCGCGTATGCACTACCTCCGCCCGCTCGGGGTGGCGCAGCAGGAGGCTCTCCGCGGCGCCTCCACGAAGATGGTGGCGCAGTGGCTGGCGCGCTCGGAGCCGCCGCTGGGGCACGAGGTGGTTCGGTACATGCTGGACGCAGACTCGCACGTGTGGAGCATGAGGAAGAGCAAAGCCAATTGGTTCAGAATCGTGGCGGTGTTGGCTTGGGCGGTTGGACTGGCAAAATGGTTAGATGACATAAGAAGGTGGAAGAATCCAGTTACCACAGTGCTACTCCACATTCTTTACTTAGTCCTCGTTTGGTATCCGGATTTGATTGTTCCCACCGCCTTCTTATATGTTGTTTTAATCGGAATATGGTATTACCGGTTCAGACCCAAGATTCCGGCCGGGATGGATACCCGCTTGTCGCAGGCCGAAGCGGTTGATCCGGACGAGCTGGACGAGGAATTCGACACCATGCCAAGTTCCAAACCACCCGATGTAATTCGAATGCGATATGATAGGTTAAGAATGCTGGCTGCAAGGGTGCAAACAGTGTTGGGTGATTTTGCCACACAGGGTGAGAGGCTTCAAGCATTGGTTAGTTGGAGGGACCCTAGGGCTACAAAATTGTTCATTGGAGTCTGCCTTACCATAACTGTAGCACTCTATGCTATGCCACCAAAAATGGTGGCGGTGGCGTTAGGCTTTTATTACCTCCGCCACCCCATGTTCCGGAACCCAATGCCCTCCGCCACGTTGAACTTTTTCCGGCGACTTCCCAGCCTCTCGGATCGCTTGATGTAG

>Glyma05G098700

ATGGCGGAAGGTGCTGGAAGGAAGCTCATGGTGGAGGTTTGCAACGCGAAGAACCTGATGCCGAAGGATGGTCAAGGAACCGCGAGTGCTTACGCGATCGTTGACTTCGATGGCCAGCGACGGCGAACCAAGACGAAATCGAGAGATCTCAATCCTCAATGGGACGAGAAACTCGAGTTCATCGTCCACGACAAAGACTCCATGCCTTCCGAAACGCTGGAGGTGAATATCTACAACGACAAGAGGACAGGGAAACGAAGCACTTTTCTCGGCAAAGTCAAGATTTCCGGAAGCACGTTTGTGAAATCCGGTTCCGAAGCGATTGTCTACTATCCGTTAGAGAAGAGGAGCGTGTTCTCTCAGATCAAAGGAGAGCTCGGTCTCAAGGTTTGGTACGTCGAGGACGATCCACCGGAAACAGAAAACGCCGGTGAGGAGAAAGCAGAGTCAGCACCAGCGGCGGAGGAGAAGCCGCCGGAAAAGGAAAAGAAAGAAGATGAGAATAAACCTAAAGAAGAATCAAAAGAAGAAAAACCAAAGGAGGAGGCTACGGCGGAGGCGGCGGCACCGCCACCGCCGGAGGTGGAGAATCCACCTATTGCGCAGACGGAGAAGCCAAAGCCGCCGAAGGAAAAGCACGGCGAAGTGCAGAAGCGTGCGGATCTGAACGTGAGCGATCACGAACTGCGATCTCTGAGAGGCGATCGGAGCTGCAGTGCCTACGACCTCGTTGATCGCATGCCGTTTTTATACGTTCGCGTGGTGAAGGCGAAGAGACCTAAACCAGAAACCGGTTCGACCGTTTACTCGAAGCTTGTGATCGGGACTCACAGCGTGAAGACCAGAAGCGAGAGTGAAGGCAAAGACTGGGATCAGGTTTTCGCCTTCGATAAAGAAGGCCTCAATTCGACGTCGTTGGAGGTTTCCGTTTGGTCAGAGGAGGTCAAAGAAGGGGATGAGAAAAGCGAGAGTTCTCTCGGAACGGTGTCGTTTGATCTGCAGGAGGTTCCTAAAAGAGTTCCTCCGGATAGTCCTTTGGCTCCGCAGTGGTACACTCTCGAATCCGAAACCTCGCCGGGAAATGACGTCATGCTCGCCGTTTGGATCGGGACTCAGGCTGACGAGGCCTTTCAGGAGGCTTGGCAGTCCGATTCTGGCGGGCTGATACCGGAGACAAGAGCTAAAGTGTATCTGTCTCCCAAGCTTTGGTACCTGAGACTAACGGTCATCCAAACCCAGGACTTGCAGCTAGGTTCGGGACCCGAGGCTAAGGCTCGAAATCCGGAGCTGTACGTGAAGGCTCAGCTCGGCGCGCAGGTTTTCAAAACAGGAAGAGCCTCACCTGGCTCGGCTAATCCAACGTGGAACGAGGACCTCGTGTTCGTAGCAGCCGAGCCGTTTGAGCCGTTTTTGGTGGTCACAGTGGAGGACGTGTCAAATTCCAAAACCGTTGGCCACGCGAAGCTCCACGTGGCATCCATCGAGCGGAGAACAGATGATCGAACGGACCCGAAGTCAAGATGGTTCAACCTCTCCAGCGAAGACGAGAGCAATTCGTACACGGGTAGGATCCACGTTCGAGTATGCTTGGAAGGAGGGTATCACGTGATAGACGAAACCGCTCACGTGACCAGCGACGTTCGAGCCTCGGCGAAGCAACTCGCGAAGCCCCCAATCGGGTTGCTCGAAGTAGGGATTCGCGGCGCTGCGAACCTTCTCCCGGTGAAGACCAATGACGGGACACGTGGCACCACGGACGCTTACGTGGTGGCCAAATACGGACCCAAGTGGGTCCGAACCCGAACCATCATGGACAGGTTCAACCCGAGATGGAACGAACAGTACACGTGGGATGTTTTTGATCCTTGCACGGTTCTCACCATTGGAGTGTTCGATAACGGGAGATACAAGCGAGGGGAAGATGGAGAACCTAACAGAGATTGCAGGGTAGGGAAAGTTCGCGTGCGGTTGTCCACGCTGGACACCAACCGGGTGTACGTAAACTCGTACTCCTTAGTCGTTTTGCTACCAAGTGGCGCCAAGAGAATGGGGGAGATAGAGATTGCTGTTAGATTCTCATGCTCCTCATGGTTGAGTCTAATGCAGGCCTACGCAAGCCCAATCTTACCAAGAATGCACTACGTGCGCCCCTTCGGCCCGGCCCAACAAGACATCCTACGCCAAACGGCCATGAAGATCGTAACGGCTCGCCTCGCCCGGTCTGAACCGGCATTGGGTCAGGAAGTGGTTCAGTTCATGCTGGACTCCGACACTCACGTGTGGAGCATGCGGCGGAGCAAGGCGAACTGGTTCAGAGTGGTGGGATGTCTCTCACGTGTGGCAACCCTACTGGGATGGGTGGATGGGATCCGCACGTGGGTGCACCCTCCCAGGACGGTTCTGGTGCACGTGCTGCTCGCAGCGATTGTGTTGTGCCCATACTTACTGCTCCCTACTGTGTTCATGTATGCTTTCTTGATTTTGGTGCTGAGATTCCGTTACCGGCATAGGGTCCCACAAAACATGGATCCGAGGATGTCGTACGTGGACATGGTGAGCCTGGACGAGCTGGACGAGGAGTTTGACGGGTTTCCGACCACGCGGCCGGCGGAGGTGGTGCGGATCAGGTACGACAGATTGCGCGCGCTTGCGGGGAGGGCGCAGACGCTGCTGGGTGACGTGGCGGCGCAGGGGGAGCGATTGGAGGCGCTGTTTAGCTGGCGGGACCCACGTGCCACGGGGTTGTTCGCGGTGCTGTGTTTGGTGATGTCGTTGCTGTTCTACGCGGTGCCGTTTAGGGGCTTTGTGCTGGTTGCGGGGTTCTATTACCTGCGCCACCCGAGGTTCCGCGATGACATGCCGTCTATCCCTGCTAACTTCTTCCGGCGACTCCCGTCCTTTTCTGATCAGATTATGTGA

>Glyma07G072500

ATGAGCAACCTCAAGCTAGGTGTGGAAGTTGTGGGTGCTCATGACCTTATGCCCAAAGATGGACAGGGCTCATGTAGTACTTATGTGGAACTTCACTTTGATGGTTGGAAATTTCGGACTACAACTAAAGAGAAAGATCTGAATCCTGTTTGGAATGAGAAATTTTACTTCAATGTTACTGATCCAAGCAAATTGCCGAATCTCACTCTTGATGCCTGTATCTACCATTATAGCAAGAGAAGTAATTCCAAAATTTTCCTGGGTAAGGTCCACCTCACTGAACCCTCATTTGTCCCATATGCTGATGCTGTTGTTTTGCACTACCCTCTGGAAAAGAAAAATGTTTTTTCCCGCATAAAAGGAGAACTTGGTTTGAAGGTATATGTCACTGATGACCCTTCAGTAAAATCCTCAAACCCTATTCATGATGTGGAACCCTCTGTGGACACCGTCCAACACTCAACCCCAGATCAATCACCAGTTTCATTCACTAATTCAATCCTGAACGTCTTTTCTCGGAAGAAAAATGAGACAAAGCACACATTTCATACCCTTCCAAATTCAAATGAAGAAAAACAGCATAAATCTTCTCCTTCAGCTGCTGCAAAGACAAATAAAGACTCTGGGATGCATGAGTCTAAATCTGGACTGCCTCCTCCAAAAGTTTTTCATGCATATCCAGGTTCTTTCTCTCCAATGGATTATGCACTCAAAGAGACAAGCCCTTTTCTTGGAGGGGGACAAGTAGTTGGTGGGCGAGTTATTCGCGGGTACAGGCCATCCAGCTCCTATGACCTTGTTGAACCAATGCAATACCTATTCGTACGAGTTGTAAGAGCTCGTTTGACTGGGAGCATTGATCCATATGTGGAGGTAAAGGTTGGAAATTTCAAAGGAATTACCAAACACTACGAGAAAACACAAGATCCTGAATGGAATCAAGTGTTTGCCTTTGCAAGGGAAAATCAGCAGTCAACTTTGCTTGAAGTTGTGGTCAAAGACAAGAATATGTTACTTGATGAAATTATTGGCACTGTGAAGTTTGATCTCCATGATGTTCCTAGACGTGTTCCACCTAATAGTCCATTGGCTCCTGAATGGTATAGGATTGACAAGGGTAAGGACAAGAAAAAAGGGGAGTTGATGCTTGCTGTATGGTTTGGCACACAAGCTGATGAAGCTTTTCCTGATGCTTGGCATTCTGATGCTCTCTCCTCTGGTGATATATCCTCGTCTGCATATGCTCATATGAGATCAAAAGTTTACCATTCACCAAGATTATGGTATGTACGGGTTAAAGTGATTGAGGCTCAGGACTTACATGTGTCAGAGAATTCCCAAATCCATGATGCCTATGTTAAGCTACAGATCGGTAACCAGATTTTGAAGACAAGACCTGTTCAATCAAGGACCATGATTCTGCGTTGGGATCAAGAGCTGATGTTTGTTGCTGCTGAACCCTTTGAGGAACCTCTGATTGTTTCAGTGGAAAATCGAGTTGGTCCCAACAAAGACGAGACTATTGGAGCTGTTATTATTCCTGTAGACCAAACTGACAAGCGAGCTGATGATAGACTTATCCACACTAGGTGGTATCACCTTGAAGAATCCATATCATCTGTGATGGATGGGGAACAAGGAAAAAAGGAGAAAGATAAATTTTTTAGTAGAATTCACCTGAGTGTCTGTCTTGATGGTGGTTACCATGTTTTTGATGGGTCAACTTATTATAGTAGTGATCTCAGACCAACATCAAAGCAGCTCTGGAAGAAGCCAATTGGTCTTTTAGAAATTGGCATTCTAAGTGTTGATGGACTTCATCCAACGAAAACCAGGGATGGAAGAGGGACAACAGATACATACTGTGTGGCAAAATATGGGCATAAGTGGGTTCGCACTCGAACCGTTAGTGACAGTCTTAGTCCAAAATACAATGAGCAGTACACTTGGGACGTTTATGATCCAGCTACAGTTCTCACTGTGGGGGTGTTTGATAATGGACAACTTCATAATTCGGATGGTAACAAAGATCTAAAAATAGGTAAAGTTCGGATTCGGATCTCGACCCTGGAAGCTGGCCGTGTTTACACAAATGCATACCCATTACCAGTGCTACATCCTTCAGGTGTCAAGAAGATGGGTGAGCTGCACTTGGCCATTAGATTCTCCTGTTCCTCAATGGTTGATTTGATGCAGCAGTATTTCAAGCCTCACTTGCCAAAAATGCACTATAAAAGGCCACTTAACTTAATGGAACAGGAAAAGCTGCGACATCAAGCGGTCAATGTTGTTGCTTCTCGACTAAGTAGGGCGGAACCCCCACTTAGAAAGGAGGTAGTTGAATACATGTGTGACACAGATTCTCATCTTTGGAGTATGAGGCGGAGCAAGGCAAACTTCTACCGTCTGATGACAGTGTTTTCTGGAATTCTCTCTGTTGTGCGATGGTTAGGGGAAGTTTCCACATGGAAGCATCCTATAACAACAGTGCTGGTGCACATTCTTTTTCTGATGCTTGTATGTTTCCCTGAACTTATTCTGCCAACTGTATTTCTATACATGTTTGTCATCAGCATGTGGAATTGGAGGTTCCGGCCGAGGTGCCCTCCTCACATGAACACCAGACTCTCCTATGCAGAAGGGGTGACCCCAGATGAGCTTGATGAGGAATTTGACACATTCCCCAGCTCAAAGAGCCCTGACATTCTACGTTGGAGGTACGACCGGTTAAGAACTGTGGCTGGGAGGATTCAGAGTGTTGTAGGAGATTTAGCTACTCAAGGAGAGAGGATCCAAGCTCTTGTGAATTGGCGTGATCCTCGTGCCTCCGCCATGTTCATGGTATTCTGCTTTGTGGCTGCTATAGTGTTGTATGTCACACCCTTCCAACTGCCTATTCTTCTGACTGGATTTTACTTGATGAGGCACCCCATGCTTCGGAGCAAGGTGCCGCCTGCTCCGGTGAATTTCTTCCGTAGGTTGCCTTCTCTCACAGACAGCATGCTGTAA

>Glyma07G089100

ATGGCAAACCAAAACCAAAACCAAAAGAAGAACAAAGAGGATTTCTCTCTGAAGGCGACAACTCCAAACATCAGTGCTGGAAGAGCAATCAGTAGTGATAGGCTCCCCACTGCATTTGACTTGGTTGAGAAAATGCAGTTTCTGTTTGTCAGGGTCGTGAAAGCCAAAGACTTGTCTGAGAAATGTGAATCTCAACCTTGCAACCCTTTTGTGGAAGTGAATCTTGGAAGCTTCACAGGAAGAACAAGATGCTTGGAGAAAACCACCACCCCAGAATGGAACCAAGTCTTTGCTTTTGCCAAGGAGAGAATTCAAGTGCTAGTTTTGGAGATTGTGGTGAAGGACAAGGATAATGATGACCCTGATGAGTTCATGGGACGTGTTGCATTCACAATTTGTGATGTCCCAGTGAGAGTTCGTCCCGATAGCCCCTTGGCGCCGCAATGGTACAAGCTTGAGGATCAAAATGGGGTGAAGCTTCAAGGAGAGTTGATGGTGTCTGTTTGGATGGGAACTCAGGCAGATGAAGCTTTTTCTGAGGCTTGGCATTCAGATGCATCAGAAACTAGTGGTGAAAGCATTGCTCACACTCGTTCCAAGGTATATATTTCTCCCAGGCTTTGGTACCTTAGAGTTAATGTGATTCAAGCCGAGGATTTGTTGCTAAAAAATAGTAATATTGGAATTTTCATCCAAGGTGTTTTGGGGAACTTAGCTTTGAGGAGCCGTCCTATGAAGTGTAATGCAAGCCCCATGTGGAATGAGGATTTGATGTTTGTTGTGGCAGAACCCTTTGACGATTGCTTGCTTGTGACCATTGAGCAGGGGAATCCCCACAAACACGAAAGCTTGGGAATTTGTGTGGTTCCTTTGAAGAATGTGCAGCAGAGAATTGATGCCACTCCACAAGCTAGTGTGTGGTGCAATCTTCAGAAGCCAAAGGAAAAGGAGGGTGAAGAGGAGGTTGGGTTTTCAAGTAAACTTAACATGAGGATCTCTTTGGATGGAGGGTACCATGTTCTTGATGAGGCCACTCACTACACAAGTGATGTAAGGCCATCTTCTAAGTATTTATGCAAGCCAAGTATTGGTGTTCTTGAATTGGGAATCCTGAATGCTGTGGGGCTCTCTCCAATGAGTAAGGAAAACCGCACCGATGCCTTTTGTGTCGCTAAGTATGGTCCCAAGTGGGTGAGGACAAGGACTATTGTAGATAGTCTTTCTCCCAATTGGAATGAGCAATATACATGGGAAGTGTTTGATCCTTGCACTGTGATCACCATTGTTGTGTTTCATAATGGAAACTTGAATGGAGGCAAAAATGCCGGAGGGAAAAAGGCTGAAGGAGCAATGGATAGGAGAATTGGCAAGGTAAGGATTCGGTTGTCAACACTTGAAAGTGATAGGATTTACTCTCACTCTTACCCTCTTATAAACTTGCACACTCAAGGAGCCAGGAAGATGGGAGAAATTCAATTGGCTGTGAGGTTTTCTTGTTCATCATTGTTGAATGTTTTGCAAACTTATGCACAACCTTTGCTCCCAAGAATGCACTATATAAGCCCATTGTCTATATTTCAGTTGGATAATTTGAGGAACCAAGCTGCTGCCATCGCCACATTGAGGTTCAAAAGGGCTGAACCACCACTCAGTAAAGAGGTTGTGGAGTACATGCTAGACATGGGGACAAATGTGTGGAGCATGAGGAGGGGAAAAGTTCAGTTTTTCAGGATTGCTTGTCTTCTCAATGTTTTGGTTTCTGTTGCTAAACAGTTTCATGAGATACATGCTTGGAAGAATTCAATTACCACGGTTGGTGATAATTTGAAGAAGAGGTATGATCGATTAAGAGCCATTTCGGGGAGGGTGCTAGAAATGATGGCTGACTTGGCAACTCAAGGAGAGAGGGTGCAGGCTCTACTCAGCTGGAGGGATCCAAGAGCAACATTTTTGTTTGTTATTTTCTGTTTTGTGGCTGTCATTGTGACATATTTGGTTCCTTTTCGCATTTTGATGTTCATGTGGGTCACTTATGTCTTAAGGCCTCCAAGGTTTCGATTTGATATGCCTGCAGTCCCACAAAACTTCCTCAGGAGGATGCCGGCAAAATCAGATGGCATGCTATGA

>Glyma08G041800

ATGAGTAGTAGTAAGAAAAGGTTCATTCCTATTAATCTGGAAGATGCTGCAGTGGACTTGTTAAACCATGTGGTGAAGTTGAAGGAGAAGGGATGGATTCCACTCTTCATTCCACTCATTTTGATTGCTTGGGCTATTCACAGATGGCTCTTCTCTTTCTCCAATTGGCTTCCCCTTGTACTTGCTCTATGGGCATCTATGCAATATGGAAATTATCAGCGCAAACTACTTGAGGAAGAGTTGAATAAGAAATGGAAACGGATCCTACTCAACACCTCGCCCATGACACCATTAGAACACTGTGAGTGGTTGAATCTGCTATTGACACAAATTTGGTCCAACTATTTTAACCCAAAGTTCTCAAGAAGGTTAAAAGCCATAGTTGAGAAACGGTTAAAGCTCCGGAAACCAAGATTTATAGAAAAGGTTGAGGTCCAAGAGTTTTCACTTGGATCATGCCCTCCAAGTTTGGGTCTTCAAGGGATGCGATGGTCAACCTCTGGTGGTCAGCGAGTCCTCAAAACGAGTTTCGATTGGGACACGAGTGAAATGAGCATTTTGATGCTTGCTAAGCTTTCCGTTGGAACAGCCAGAATTGTGATTAACAGTCTTCATATTAAGGGTGATCTTCTGGTGACACCAATTCTAGATGGAAAAGCACTTTTGTATTCCTTTCTATCAATACCTGAGGTGAAAATAGGAATTGCCTTTGGAAGTGGAGCAAGCCAATCAGCCACTGAGTTTCCTGGTGTTTCTTCATGGCTGAATAAACTTTTTACTGATACCTTAGCTAAAACCATGGTGGAACCTCGCCGTCGTTGTTTCAGTTTGCCTGTGGTTGATTTGAGGAAAACTGCTGTTGGAGGCATTATATATGTGTCAGTGATTTCAGCAAACAAACTTTCTAGGAGTTGCTTCAAGAGTAGCCCATCTTTGAGGCAACAAAATAGCACAATCAATGGATATTCAGAAAACAACCTGGATGACAATGACCTACAGACATTTGTAGAGGTAGAAGTGGAGGAATTGACAAGGAGGACGGGTCTGAGCCATGGTTCAAATCCAATGTGGGATACAACATTTAATATGGTTTTACATGATAATACAGGAATTGTTCGCTTCAATCTTTATGAGTGTCCCTCAAGTGGTGTTAAGTGTGACCACCTAGCAAGTTGTGAAATCAAGATGAGGCATGTTGAAGATGATTCAACAATAATGTGGGCAATAGGACCTGATTCTAGTGCAATAGCAAAGCATGCAAAGTTTTGCGGAGATGAAGTTGAAATGGTTGTCCCATTTGAGGGGACCAACTCAGTAGAGTTAAAGGTGAAGTTTGTAGTAAAAGAGTGGCAGTTTTCTGATGGTTCGCATAGCTTGAACAGCCTCCGTTCTAATTCTCAGCGATCACTTATTGGATCATCAAGTCTTCTGTCAAAAACTGGAAGGAAACTTAAGATAACTGTTGTAGAAGCAAAGGATCTCGCTGCAAAAGACAAATCTGAAAAAATTAACCCATACATTAAATTGCTATATGGAAAGGTTGTCAAGAAAACAAAGGTTGCTCTTACTACTACTAGTACTACTACAAATCCTGTCTGGAATCAATCATTTGAATTTGATGAGAATGATGGTGATGAATACCTAAATGTAAAATGCTTTAGTGAAGAAATTTTTGGAGATGAAAATATTGGTAGTGCAAATGTAAATTTGGAAGGACTGGGGGATGGGTCAATCAAGGTTGAATGGATCCCTCTTGAAGGAGTGAGTTCGGGTGAATTGAAGCTTAAAATTGAAGTAGTTAAGGTGGAGGACCAAGAAGGATCAAGGGGTTCAACTAATGGTTGGATAGAACTTGTTGTGATTGAAGCGAGGGATCTTATTGCTGCTGATCTTCGAGGGACAAGTGATCCTTATGTGAGGGTAAACTACGGAAACTCGAAGAAAAGGACAAAGGTTATACACAAAACACTCAACCCTCGTTGGAACCAGACATTAGAGTTCCTTGACGATGGCAGTCCCTTGATACTGCATGTTAAGGACCACAATGCTTTACTACCCGAATCAAGTATAGGCGAAGGTGTTGTAGAATATCAAAGGTTGCCTCCAAACCAGATGTCTGACAAGTGGATACCTCTGCAAGGGGTGAAAAGTGGTGAGATCCACATCCAAATTACAAGAAAAGTTCCAGAAATGCAAACAAGACATACTCTAGACTCTCAACCCTCTTCGTTAAGTAAATCACACCAAATTCCTACCCAGATGAGAGAGATGATGAAAAAGTTTCGATCATTAATAGAGGATGAAAATCTTGAAGGATTAACAACAACTTTGAGTGAGTTAGAAAGTCTAGAGGATACACAGGAAGGGTATATAACACAGTTGGAGACAGAGCAAATGCTTCTACTCAGCAAAATAAATGAACTGGGTCGGGAGATCATCAATTCTTCTTCCCGCGCTAGTAGTAGTCCTTCTCAAAGTGGAATTTGA

>Glyma08G152000

ATGATTTCAATAAAGAGAAGACAAAGAGGTGCTCCAGTCACAATGCACCCAGTTGGTCCTCAAGTCCATCCTAGTAGCCATGATGAAGACTACAATCTGAGGGAGACCGATCCACAGCTTGGTGGGGAGCGGTGGCCTAATGCCACAAGAGGGTGGATGAGTGGTGGTGAAAGATTCTCAAGCACACATGACCTTGTTGAGCAGATGTTCTATCTGTATGTTAGGGTGGTGAAAGCCAAAGATCTTTCCCCCAGCACTCTCACCTCAAGCTGTGATCCTTATGTGGAAGTGAAGCTGGGGAACTACAAAGGAAGAACAAAGCACATAGAGAAGAAAACCAACCCGGAATGGAACCAAGTCTATGCTTTCTCCAAAGACAGATTTCAATCTTCTGTTTTGGAAGTCATTGTGAAAGACAGAGAAATGCTGGGAAGAGATGATTATATTGGAAGGGTGGCATTTGATCTCAATGAGGTTCCAACCAGAGTTCCCCCAGACAGTCCACTGGCTCCTCAGTGGTACAGACTCGAGGACCGGCGAGGAGAAGGGAAGGTGAGGGGTGATATCATGCTTGCAGTGTGGATGGGAACTCAAGCTGATGAAGCCTTCTCAGAGGCATGGCATTCTGATGCTGCCACTGTGTATGGAGAGGGTGTTTTCAATGTCAGATCAAAGGTTTATGTGTCACCAAAACTGTGGTATCTAAGGGTGAATGTCATTGAAGCACAAGATGTGATACCAAGTGACAGAAACCGGCTGCCAGAGGTTTTTGTCAAGGCTCAGATGGGGAGCCAAGTGTTGAGGACCAAGATATGTCCAAGTAGAACAACCACACCACTTTGGAATGAAGATTTGGTATTTGTGGCAGCTGAACCATTTGAGGAGCAGTTGACAATCACTGTGGAGGATCGAGTGAACCCTTCAAGAGATGAAGTACTTGGTAAGATAATCTTGCCACTGACCCTCTTTGAGAAGCAGCTAGACCACCGGCCAGTTCATTCGCGCTGGTTCAATCTTCAGAAGTTTGGTTTTGGAATGATGGAAGCTGATAGGAGAAATGAGCTCAAGTTTTCAAGCAGGATTCACCTAAGAATTTCCCTTGAAGGTGGATACCATGTCCTTGATGAGTCCACTTTGTACTCCAGTGACCAAAGACCAACAGCTAGACAGCTATGGAAGCAGCCTATTGGGGTGCTTGAAGTAGGCATCTTAGGAGCAAAAGGACTCCTCCCAATGAAGATGAGGGATGGCCGTGGCACCCTGGATGCATACTGTGTTGCCAAGTATGGCCAGAAATGGGTCAGAACCAGAACAATTCTTGACACTTTTAGTCCAAAATGGAATGAGCAATACACATGGGAGGTTTATGATCCTTGCACTGTTATAACACTTGGTGTCTTTGACAACTGCCATTTAGGTGGAGGGGAAAAAGCCACTGCTGGCACTGCAGCCAGGGATTCAAGAATTGGAAAGGTAAGAATTAGGCTCTCAACACTTGAAGCTCATAGGATATACACTCATAGTTATCCACTTCTTGTTTTGCACCCTCATGGTGTCAAGAAAATGGGTGAGCTTCAGCTAGCAGTGAGGTTCACTAGCCTCTCACTGGCTAACATGGTTTACATTTATGGCCAACCCTTGCTTCCAAAGCTGCATTACTTTCGCCCTTTCACCGTTAACCTAGTAGAGAGTTTGAGGTACCAAGCCATGAACATTGTAGCTGTTAGGCTTGGAAGAGCTGAACCTCCCCTCAGGAAGGAGGTAGTAGAGTACATGTTAGATGTTGATTCCCATATGTGGAGCATGAGAAGAAGCAAAGCCAACTTCTTCCGAATCATGTCACTTTTCTCTGGATTCATCACAATGGGGCAATGGTTTACCCAAGTTTGCCATTGGAAGAACCCCATCACATCAATCCTAGTTAACATTCTCTTCCTAATACTCATTTGCTACCCTGAATTGATACTTCCAACCTTGTTCCTCTACATGTTCTTGATTGGCCTGTGGAACTATAGGTTTAGGCCTAGACACCCACCCCACATGGACACAAAACTCTCATGGGCAGAAGTTGTTCAACCCGATGAACTCGATGAAGAGTTCGATACATTCCCAACTTCTAGACCACATGATGTGGTGAGAATGAGGTATGACAGACTTAGAAGTGTGGCAGGAAGGATTCAAACAGTTGTTGGGGACATAGCAACACAGGGAGAGAGGTTTCAGTCTCTACTGAGTTGGAGAGACACAAGAGCAACCAGCCTCTTTGTAGTGTTCAGCTTTTGTTCTGCTGTGGTTCTCTATGCAACACCACCAAAAGTGGTGGCTATGGTAGCAGGCTTGTACTATCTGCGGCACCCAAAGTTTCGCAGCAAGCTTCCTTCTGTGCCAAGCAACTTCTTCAAGAGGCTTCCAGCTAGAACAGATAGCATGCTGTGA

>Glyma08G239300

ATGATGAACAAGCTGGTGGTAGAAGTTGTGGAGGCGAGCGACCTCATGCCCAAAGATGGGGAAGGGTCAGCTAGCCCCTTTGTAGAGGTCAAGTTCGATGAGCAGCAGCACAGCACTGAAACGAGGCACAAAGACCTGAATCCTTGTTGGAATGAAAAGCTTGTGTTCAACATCAATAACCCCAGAGATCTTGCCCACAAGACAATTGAGGTGGTTGTATACAATAACAATCACAATGATCGGAACCACAATAACTTCCTTGGAAGGGTGAGACTTTCAGGCTCCTCAATCCCTCTATCGGAGTCCCAGGCCAGTGTGGAACGCTACCCACTTGAGAAACGTGGTCTCTTCTCAAATATCAGGGGAGATATTGCTCTTAGATGTTACACGCTGCATGATCATCATCATCATGCTCATGCTGCTGCGGAACACCATCACCATCACCCCCAAGTAGACACTCCTCCTCCTCCTGCTGCTGCTGCTGCTGCTGCTGAGGAGGAGGAGGAGGAGTATCAGGACACTCCCTTTCAAGAAATAAACCCCAACATGAACACGGTGCTAGATGAGGAAAGCGCGGTTGGTGGCGGGGACAAGAAGAAAAAGAAGATGCAAAAGAAAGAAAAGGAAGTCAGGACTTTTCACTCTATTCCAGCAGCACCCGCGATGGAAACGACCCAAAGGAGGGTTGATTTTGCAAAAGCTGGGCCACCCAATGTAATGTTAATGCAGCAGATCCCAAAGCAAAACCCTGAGTATTCATTGGTGGAGACAAGTCCACCACTGGCGGCTCGTTTGCGCTATAGAGGAGGCCGGGACAAGATATCCACCACCTACGACTTAGTGGAACAGATGAATTACTTGTACGTCAATGTTGTCAAGGCTAGGGATCTCCCTGTTAAGGATATCACTGGTAGCCTTGACCCTTATGTGGAAGTCAAGCTTGGTAACTACAAGGGCCTCACCAAGCACTTGGACAAGAACCAGAACCCTGTCTGGAATCAAATCTTCGCCTTCTCCAAGGACAGGCTGCAATCAAATTTGCTTGAAGTCACCGTCAAGGATAAGGACATTGTCAAGGATGATTTTGTGGGGAGAGTTATGTTTGATCTCACTGAGGTTCCTCTTCGGGTACCCCCAGACAGCCCCTTGGCTCCTCAATGGTACATATTGGAGGACAAGAAGGGCCAAAAAATTCATAATAATGGGGAAATCATGCTTGCGGTTTGGATGGGAACACAAGCGGATGAGTCCTTCCCCGAGGCCTGGCACTCTGATGCTCACAACATTAGTCACTCCAACCTTGCAAACACCCGCTCAAAGGTATATTTCTCGCCAAAGCTTTACTATCTTAGAGTTCAAGTAATCGAGGCTCAGGATCTTGTTCCTTCCGATAAAGGAAGGGCCCCGGACGCCATTGTTAGAGTACAGCTGGGGAATCAGATGAGATTCACAAGGCCTTCTCAAATTAGAGGCATCAACCCAGTTTGGAATGACGAGCTTATGTTTGTGGCAGCCGAACCATTTGAGGATTTTATCATTGTGACTGTTGAGGACAAAGTAGGTTCTAGTGTTGAAATCTTAGGAAGGGAGATCATTTCGGTGAGAAGTGTTCCTCCGAGACACGAGTCCAGCAAGAAGCTCCCCGATTCTCGTTGGTTCAATTTGCACAGGCCTAGTGCGGTTGGCGAGGAGGAAACAGAGAAAAAGAAGGACAAGTTCTCAAGCAAGATTCACCTCCGAGTGTGTCTTGAGGCCGGGTACCACGTCCTTGATGAGTCCACGCATTTCAGCAGTGATCTTCAGCCATCCTCCAAACATTTGAGGAAGAAAAACATTGGAATTCTCGAACTTGGGATACTGAGTGCCCGCAACTTGCTGCCCATGAAGGCCAGGGAAGGGAGGACTACCGATGCCTACTGCGTGGCCAAGTATGGCAACAAATGGGTTCGAACCAGAACTCTGCTTGACACTCTCTCCCCTCGATGGAACGAGCAATATACCTGGGAGGTTCATGATCCATGCACTGTCATCACGGTTGGGGTATTTGACAACCACCACATCAATGGGAGCAGTGATGCCAGAGATCAGAGAATTGGAAAGGTGAGAATCAGGTTGTCAACTCTGGAAACTGATAGGGTGTATACTCATTTTTATCCTCTGCTGGTTCTCCAACCCAATGGCCTCAAGAAGAATGGAGAGCTTCACTTGGCGGTGAGGTTCACCTGCACTGCATGGGTTAACATGGTAGCCCAGTATGGTAGGCCTTTGCTTCCCAAAATGCATTATGTCCAACCCATACCCGTCAGGCACATAGATTGGCTCCGCCACCAGGCCATGCAGATTGTGGCCGCTCGCCTGTCTAGAGCAGAGCCGCCCCTTAGGCGCGAAGCTGTTGAGTACATGCTCGACGTGGATTACCACATGTGGAGTCTAAGGAGAAGCAAAGCCAATTTTCATCGCATAATGTCACTCCTCAAAGGAGTTACTGCTGTTTGCAAGTGGTTTGATGACATCTGCACTTGGAGAAATCCAATCACAACCTGCCTTGTTCACGTCTTGTTCTTGATACTGGTTTGCTACCCGGAGTTGATACTGCCCACCATTTTTCTTTACTTGTTTGTAATCGGGATTTGGAATTACCGCTTCAGGCCAAGGAATCCACCCCACATGGATGCTAGGCTTTCACAGGCAGAGACTGCTCACCCAGATGAACTGGACGAGGAATTTGACACTTTTCCAACAACAAAGCCTTCAGATATTGTGAGAATGAGGTATGACAGATTGCGGAGTGTGGCAGGTAGAGTACAAACTGTGGTTGGAGATTTGGCTACTCAAGGAGAAAGAGCTCAAGCCATACTAGGTTGGAGAGACTCCAGGGCCACATCTATCTTCATCATCTTCTCACTCATTTGGGCCGTTTTCATTTACATTACTCCCTTCCAAGTGGTTGCAATTCTAATTGGCCTCTTTATGCTGCGTCATCCTCGCTTTAGGAGCAAGATGCCATCAGTACCAGTTAATTTCTTCAAGAGATTGCCTTCCAAATCAGATATGCTTATATGA

>Glyma09G003400

ATGAAACTGGTTGTGGAAGTTATTAATGCTCATGATCTTATGCCCAAAGATGGCGAGGGATCAGCCAGTCCCTTTGTGGAAGTAGACTTTGAGAACCAGCTTAGCCGAACCAGAACCGTCCCAAAGAACCTCAACCCCACTTGGAACCAAAAACTAATCTTCAATTTAGATGCAACCAAACCTTACCATCGCCAAACGATTGAAGTATCGGTCTACAATGAGAGGCGACTTACTCCAGGCAGAAACTTCCTTGGAAGGGTGAGAATTCCTTGCTCCAATATTGTCAAGGAAGGTGAGGAAGTATATCAGATTTTCCCTCTTGAAAAGAAGTGGTTTCTCTCACCTGTTAAGGGTGAGATTGGCCTCAAAATATACATTGCATCAGAGTCTAATTCCAAACCAAAACCTCTTTCTCCTGTTTTCCCTTCAGAACAAGAAAAACTTCCACCTTCCACTCCACCCCGAGAACCAGAATCCACCATTAGTGACCTTCCTCCGCCACCTCATAGTATCCCCTCAGGCCTAACTGACAGAACATTAGAAGCTGATCTCAGTGAAGAACTTCCTGCATTTGACACCCCAAAAGCAAGCACAGAAGAAGCAGAAGTATATTATGTTGCAGAAGCTCGGTCTAGCAGTGTTGATATCGATCAAGAGCCAAAGAAAGAAAATAGAGAAGCTGTCGTAGAGACCGTCCAACAACTTGACAAGCACCAAGTTCTCCAGCCACAAACGATTTCAATAAAGAGAAGACCACAAGGTACTCCATCCACCATGCACTCAGTTGATCCTCAAGTCCAATCTAGCCATCACGAAAACTATAATCACAATGACACCAATCAACAGCCAAGGATTTCAATAAAGAGGCGACCGCTAGCGCAAGGTGCTCCATTCACGATGCACTCGGGTGATCCACAAGTCCAACCAAGCCATGGTGAAGGCTACAATCATAATGACACTAACCTGCAGCCAAGGATTTCAATTAAGAGACGACCGCGAGGACCGGGTACTCCATCATCAATGCACTCCTTTAATCCACAAGTCCATGCTAGCCGCAACGAAAGCTACAATAACCTCATGGGAACCAACCCACAACAGCCAAGAATTTTAGTAGAGAGACAACCACAGAATACTCCGCTCACCGTGCACCGAGTTAGTCCCCAAGTCCCTACTAGCAATGATGAAAACTACAATCTCAGTGACACCAATGTGCAGCTTGGCGAGCGGTGGCCCAGTGATGGAGCTTATGGTAGAAGAGGGTGGGTGAGTGGTAGTGACAGATTCACTAGCACGTATGACCTTGTTGAGCAGATGTTTTATCTGTATGTTCGTGTTGTGAAGGCAAAAGATCTTCCCCCAAGCACCATCACCTCAAGCTGTGATCCTTATGTGGAAGTGAAGCTGGGGAACTACAAAGGAAGAACAAAGCACTTTGAGAAGAAATTGAACCCAGAGTGGAACCAAGTGTTTGCTTTCTCCAAAGACCGCATTCAGTCTTCTGTTTTGGAAGTCTTTGTGAAAGATAAGGCAATGGTGGGCAGAGATGACTATCTTGGCAGAGTAGTTTTTGATCTCAATGAGGTTCCAACAAGAGTTCCACCAGATAGTCCACTAGCTCCTCAGTGGTACCGGCTCGAGGACTGGCGCGAAGAAGGCAAGGTGAGGGGTGACATTATGCTTGCAGTTTGGATGGGAACACAAGCTGATGAAGCTTTCTCTGAGGCTTGGCATTCTGATGCTGCCACTGTCTATGGGGAGGGCGTTTTCAACGTCAGATCAAAGGTTTACATGTCACCAAAACTGTGGTATCTCAGGGTGAATGTAATTGAAGCACAAGACGTGATCCCAGGTGACAGAAACCGCCTACCGGATGTTTTTGTGAAAGCTCAAGTGGGCTGCCAAGTGCTGACAACCAAGATATGCCCCACCAGAACAACCACCCCATTCTGGAATGAAGATTTGGTCTTTGTAGCCTGCGAGCCATTTGAGGAGCAATTAACAATCACTGTGGAGGATCGTGTGCACCCTTCAAAAGATGAGGTACTTGGGAAGATAAGCCTACCAATGACCCTCTTTGAGAAGCGGCTAGACCACAGGCCGGTTCATTCGCGCTGGTTCAATCTTGAGAAATTTGGTTTTGGAGTGCTAGAAGGTGATAGAAGAAATGAGCTCAAGTTTTCAAGCAGGATTCACATGAGAGTTTGCCTTGAAGGTGGATACCATGTCCTAGATGAGTCCACATTGTACACAAGTGATCAAAGGCCAACAGCAAGACAGCTATGGAAGCAACCTATTGGAATACTTGAAGTAGGCATCTTAGGAGCGCAGGGGCTTCTCCCAATGAAGATGAGGGATGGAAGAGGCAGCACAGATGCATACTGTGTTGCCAAGTATGGTCAGAAATGGGTCCGAACCCGAACACTTCTCGACACTTTTAGTCCTAAATGGAATGAACAATACACATGGGAGGTCTATGATCCTTGCACTGTGATAACGTTGGGAGTTTTTGACAACTGCCATTTAGGTGGAGGGGAAAAAGCTCCTGGTGACAGTGCTGCTAGAGATTCTCGGATTGGAAAGGTAAGAATAAGGCTATCAACACTTGAAGCTAATAGGATTTACACCAATTGTCACCCTCTTCTTGTTCTACACCAACATGGAGTTAAGAAGATGGGTGAGATTCAGTTAGCAGTGAGGTTTACGGCACTTTCACTAGCCAACATGGTTCACATCTATGGCCAACCCTTGCTCCCCAAGATGCATTACTTACATCCCTTCACAGTGAACCAAATAGACAACTTGAGGTACCAAGCCATGAACATTGTAGCTGCGAGGCTAGGCCGAGCTGAACCGCCTTTGAGGAAGGAGGTGGTGGAGTACATGTTGGATGTTGATTCCCATATGTGGAGCATGAGAAGAAGCAAGGCTAATTTCTTCCGAATCATGTCCCTTTTCTCTGGTATGATCACAATGGGAAAGTGGTTCAGTGATGTTTGCCTTTGGAAGAACCATGTTACATCAGTCCTGGTTCACATTCTTTTCCTCATACTGATATGGTACCCGGAATTGATCCTGCCAACCGTGTTTCTCTATATGTTCTTGATTGGTCTGTGGAACTATAGGTTCCGGCCTAGACACCCGCCTCACATGGATACTAAACTCTCGTGGGCAGAAGCCGTTCACCCCGACGAACTCGATGAAGAGTTTGACACGTTTCCGACTTCTAGATCACAGGATGTTGTGAGAATGAGGTATGACAGGCTTAGAACTGTGGCAGGTAGGATTCAGACAGTTGTTGGGGACATAGCTACACAAGGGGAGAGGTTTCAGTCTCTACTGAGTTGGAGAGACCCCAGAGCAACCAGCCTCTTTGTAGTGTTTAGCTTCTGTGCTGCTGTGGTTCTCTATGCAACTCCATTCAGAGTGGTGGCTCTGGTGACAGGTTTATACTTTTTGCGGCATCCAAAGTTTAGGAGTAAGATGCCTTCAGTACCAAGTAATTTCTTCAAGAGGCTCCCAGCTAGAACAGATAGTTTATTGTGA

>Glyma09G187900

ATGGCAAATCAAAACCAGAACCAGAATCAGAACCAAAACCAAAAGAAGAACAAAGAGGATTTCTCTCTGAAGGCGACAACTCCAAACATCAGTGCTGGAAGAGCGATAAGTGGTGATTGGCTCCCAACTGCATTTGACTTGGTTGAGAAAATGCAGTTTCTGTTTGTCAGGGTGGTGAAGGCCAAAGACTTGCCTGAAAAAAGTGAATCTCAACCTTGTAACCCTTTTGTGGAAGTGAATGTTGGAAGCTTCACAGGAACAACCAGATGCATGGAGAAAACCACCACCCCAGAATGGAACCAAGTCTTTGCTTTTGCCAAGGAGAGGATTCAAGTACTAGTTTTGGAGATTGTGGTGAAGAACAAGGGTGAAAATGGTGACCCTAATGATAATGGTGACCTTGATGAGTTCGTGGGACGTGCTGCATTCACAATTGGTGATGTTCCAATGAGGGTTCCTCCCGATAGCCCCTTGGCGCCGCAATGGTACAAGCTTGAGAATCAAAATGGGGTGAAGCTTCAAGGAGAGTTGATGGTTTCTGTTTGGATGGGAACTCAGGCGGATGAAGCTTTTTCTGAGGCTTGGCATTCAGATGCATCAGAGGCTAGTGGTGTTTTGGGGAACTTGGCTTTGAGGAGCCGTTCTATAAAGTGTAGTACAAGCCCCTCGTGGAATGAGGATTTGATGTTTGTTGTGGCAGAACCCTTTGATGATTGCTTGTTTGTGAGCATTGAGCAGGGGAATAACTTCAAGCACGAAAGCTTGGCAATTTGTGCTGTTCCTTTGAAGAATGTGGAGCAGAGAATTGATGCCACTCCACCAGCTAGTGTGTGGTACAATCTTCACAAGCCAAAGGAAAAGGAGGGAGAAGAGCAGGAGGTTAATTTTTCCAGTAAACTTAACATGAGGATCTCTTTAGATGGAGGGTACCATGTTCTTGATGAGGCCACTCACTACACCAGTGATGTAAGGCCATCATCTAAGTATTTGTGCAATCCCAGTATTGGTGTTCTTGAATTGGGAATCCTGAATGCTGTGGGGCTCTCTCCAATGAGCAAGGAAAATCGGACCAATGCGTTTTGTGTAGCTAAGTACGGTCCAAAGTGGGTGAGGACAAGGACTATTGTAGATAGTCTTTCTCCCAAATGGAATGAGCAATATACATGGGAAGTGTTTGATCCTTGCACTGTGATCACAATTGTTGTGTTTGACAACGGAAACTTGCATGGAGGCAACAAAAATGCCGGAGGGAAAAAATGTGAAGGACCAGTGGATAGGAGAATTGGCAAGGTGAGGATTCGGTTGTCAACACTTGAAAGTGATAGGATTTACACTCACTCTTACCCTCTTATAAACTTGCACACTCAAGGAGCAAAGAAGATGGGAGAAATTCAATTGGCTGTGAGGTTTTCTTGTCCATCATTGTTGAATGTTTTGCAAACTTATGCACAACCTTTGCTCCCAAGGATGCACTATTTAAGTCCATTGTCTATATTTCAGTTGGATAATTTGAGGAACCAAGCTGCTGCCATCACCACATTGAGGTTTAAAAGGGCTGAACCACCACTGAGCAAAGAGGTTGTGGAGTACATGCTAGACATGGGGGTAAATGTGTGGAGCATGAGGAGGGCAAGAGCTCAGTTTTTCAGGATTGCTAGTCTTCTTAATGTTTTAGTTTCTGTTGCTAAACAGTTTCGTGAGATACATGCTTGGAAGAATTCAATTACCACGGTGGTTAGTTACTTCATGTTTCTCATTGTCATTTTCTGTCCGCAAATAGTTTTGCCATCAACATTTTCCTTCCTACTCCTGGCTGGGATTTGGGGCTATAGAACAAGGCCAAGGTGTCCTTCCCATATGGATATGAGATTGTCTCAGGCTGACACAGCCAGTGTTGAAGAACTAGAAGAAGAATTTGATTCCTTTCCGTCGAAATTCAGTGGTGAAAATTTGAAGAGGAGGTATGATCGATTAAGAGGTGTCGCGGGGAGGGTGCTAGAAGTGATGGCAGACTTGGCAACTCAAGGAGAGAGGGTGCAGTCTCTACTCAGCTGGAGGGATCCAAGAGCAACAGCTTTGTTTGTTATTTTCTGTTCTGTGGCTGTCATTGTGACATATTTGGTTCCTTTCCGCATTTTGGTGTTCATTTGGGTGACTTATATGTTAAGGCCTCCAAGGTTTCGATTTGATATTCCTGCAGTCCCTCAAAACTTCCTCAGGAGGATGCCTGCAAAATCAGATGGCTTGCTATGA

>Glyma09G261200

ATGAACAATCTCAAGCTAGGAGTAGAGGTTGTGAGTGCTCATGACCTTGTGCCCAAAGATGGGCAAGGATCATCTAGTACTTATGTGGAACTCCACTTTGATGGCCAGAGATTTCGGACAACTACTAAAGACAAAGATTTGAGTCCTTTTTGGAATGAGAGCTTTTACTTCACCATCACTGATCCAAGCAAGTTACCTAGCCTCACTCTTGAAGCCTGTATCTACCACTACAACAAAGGCAATTGCTCCAAAGTACTCCTTGGGAAGGTCCGGCTCACTGGAACCTCCTTTGTTCCGTATTCTGATGCTGTTCTTTTGCACTACCCTCTGGAAAAGAAAAATATTTTTTCGCGCTCCAAAGGAGAGATTGGTTTGAAGGTTTTTGTCACAGATGACCCTTCTTTAAGATCCTCAAACCCTATTCCTGCTGTGGAATCCTTTTTCAATACAGACCAAAATGAAAATCTAACACAAGACCAAACACCACCACCGGTATCATTTACAGACTCAATCCTCAACAGCGTCTCTAGAAAGAAAACCGAGACAAGGCACACGTTTCATAACATTGCAAAATCAAGTAGTGAACAAAAACAGCAGTCTAAGCCTGCAGCAGATGCCAACCCTAGTGTAACATTTGGGATTCATGAGATGAAATCATCACAGGCTCCTCCAAAAGTTGTTCAAGCATTTGCAGGTCCACAAGAGTTTTCGGTGAAAGAAACAAGCCCTACTCTTGGAGGGGGAAAAGTTGTTGGGGGAAGGGTTATTCGCGGGAGCATGCCGGCCACATCCAGCAGCTATGACCTTGTTGAATCAATGAAGTATATTTTTGTAAGAGTTGTAAAAGCGCGCGACCTTCCTTCGATGGATATGACAGGTAGCCTTGACCCCTATGTGGAGGTAAAGGTTGGAAACTTCAAAGGAACTACCAACCACTTTGAGAAAAACCAAAACCCTGAATGGAACAAGGTGTTTGCCTTTGCCAAGGACAATCAGCAATCATTTATTCTTCAAGTTACGGTTAAAGACAAGGACAAGATATCAGATGATGTTGTTGGAACTGTGACGTTTTCTGATCTGCATGATATCCCTGAACGTATTCCACCTGATAGTCCATTGGCTCCTCAGTGGTATAGGATTGAGAACAAGAATGGAGAAAAGAGAGGGGAGTTGATGCTTGCTGTTTGGAGAGGCACACAAGCTGATGAGGCTTTTCAAGATGCTTGGCATTCTGATGCAGTAGTCTCCCCTGATGGAAGCACTATATCTAACTATGCTCAGATTCGATCGAAAGTTTACATGTCTCCGAGATTATGGTATGTACGCGTCAAAGTGATCGAGGCACAGGACTTAGTTTCATCTGACAAGTCTAAAGTTCCAGACGTCTATGTTAAGGTACACATTGGCAACCAGATTATAAAGACGAAGCCATTAAGGGACATGAACCCGCAGTGGAATCATGAGGCGTTGTTTGTCGCCGCTGAACCTTTTGAAGAGCCTTTGGTCTTCACAGTTGAAGAACGCAGTGCCAACAAGGATGAGACTATTGGCAATGTTGTTATTCCTTTAAACAGAATTGAAAAGCGTGCTGATGATAGGCCTATCCGTGATCACTGGTATCTTCTTGAAAAGTCCATGTCATCTGCTATGGAGGACCAAGCGAAAAAGAAAGAGAAAGAGAAGGAGAAGGACAAGTTCTATAGCAGAATCCGTGTCATTGCCTTTCTTGATGGTGGATACCATGTGCTAGATGAGTCAACTTATTACAGTAGTGATCTAAGGCCAACCACAAGGCAATTATGGAAGAAGCCAATTGGTGTGTTAGAACTTGGCATTTTGAATGCTGATGTATTGCCAATTCCAACCAAAAATAGGGATGGAAGGGGAACAGCAGATACATACTGTGTGGCAAAGTATGCCCACAAGTGGGTGCGCACTAGAACCATTGTTAACAATCTAAACCCAAAGTTCCATGAACAGTACACTTGGGAAGTTCATGACACAGCTACAGTTCTCACCTTGGGGGTATTTGATAATGCACAGATTACTAATTCTTCAAATGGTAACAAAGATTCCAAGATTGGAAAGGTTCGGATAAGGATCTCAACGCTAGAAGCTGGTCGTGTTTACACCCACTCTTATCCATTGTTATCAGTGCAAAACTCTGGCCTCAAGAAGAACGGGGAAGTGCACTTGGCCATACGGTTCTCATGCACCTCAATGGCTAACATGATGGCTTTGTATTTAAAACCCCATTTGCCAAAGATGCACTACACAAAGCCCCTTAACATCATGGACCAGGAAAGGCTGAGACTTCAGGCTGTGCTCATTGTTGCATCTAGACTAGGCAGGGCAGAACCCCCTCTTAGGAAAGAAGTGGTTGAGTACATGTCTGACTCAGAATCTCATTTGTGGAGCATGCGGCGCAGCAAGGCCAACTTCAACCGTTTGAAGGAGGTGTTTTCCGGGCTACTTGCATTTGGAACTTGGTTTGGACAAATTGCAACATGGAAGAACCCTTTTGTGACAGTGCTGCTGCACATTCTCTACTTAATGCTTGTGTGTTTTCCTGAACTTATTCTACCAACCGTGTTCCTATACATGTTTGTAATAGGGATGTGGAAATGGAGGTTCCGCCCGAGATACCCTCCTCACATGGATGCTAGTCTCTCTTGTGCATATGTAACCAGCCCTGAGGACTTTGATGAGGAAATGGACACTTTTCCAACCACAAAGAGCTTTGATATCGTTCGTTGGAGGTATGATAGGTTGAGAAGTCTGGCAGGGAAGGTTCAGAGTGTTGTTGGACAGATAGCAACTCAAGGAGAAAGAATCCATGCTCTTATAAATTGGAGGGATCCACGTGCCACTTCGATATTCATGGTGTTTTGCCTTGTGACTGCTATAGTTTTGTATGTGACACCACCACAGATGTTGTTTATTCTATCTGGATTTTACCTAATGAGGCACCCTATGCTTCGGGGTAAGACGCCAGGAGCACCGATCAATTTCTTCCGCAGGTTGCCTGCTCTCACAGATAGCATGCTGTAA

>Glyma10G091800

ATGCAGAGGCCTCCACCTGAAGATTTTCTGTTGAAGGAGACCAAGCCCCACCTTGGAGGCGGAAAGGTTTCCGGTGATAGACTTACCAGCACCTATGATCTCGTTGAGCAAATGCAGTACCTTTATGTGAGGGTCGTGAAGGCGAAGGACTTGCCCGCGAAAGATATCACTGGGAGTTGTGATCCTTATGTTGAAGTCAAGCTTGGGAACTACAAGGGAACCACCAGGAATTTCGCCAAGAACACACATCCTGAGTGGAACCAGGTTTTCGCCTTCTCGAAGGACCGGCTTCAGGCCTCGATGCTGGAGGTTAATGTGATAGATAAAGATGTTCTGAAGGATGACCTCATCGGCCGGGTGTGGTTTGACCTGAATGAGATCCCGAAAAGGGTACCTCCGGATAGCCCTCTGGCTCCTCAGTGGTATAGATTGGAGGATAGGAAGAGCGACAAAGCGAAGGGGGAGCTGATGCTGGCTGTTTGGATGGGTACACAGGCTGATGAGGCTTTTCCCGAAGCTTGGCACTCGGATGCTGCGATGGTTAGTGGGAGTGATGCTCTTGCGAACATTAGATCGAAAGTTTATCTGTCTCCCAAGCTTTGGTATTTGAGGGTTAATGTGATAGAGGCACAGGACCTGATGCCAACTGATAAGGGTAGATACCCTGAGGTATTTGTGAAGGCTATTCTGGGGAATCAGGCCTTGAGGACTAGAATCTCTCAAAGTAGGAGTATTAATCCAATGTGGAATGAGGATTTGATGTTTGTGGTGGCAGAACAGTTTGAGGAGCCGCTGATTTTGAGTGTGGAGGATAGAGTTGCGCCTAACAAGGATGAAGTGTTGGGGAGGTGTGCCATTCCTTTGCAGTATGTGGAGAGGAGACTAGATGAGAAACCTGTGAACACAAGGTGGTTCAATCTGGAAAGGCATATTGTGATAGAAGGGGAGAAGAAGGACACCAAATTTGCAAGCAGAATTCACATGAGGATTTGTCTAGAAGGTGGTTATCATGTTTTAGATGAATCTACTCACTACAGCAGTGATCTTCGCCCAACAGCGAAACAGCTGTGGATGCCTGGTATTGGTGTTCTTGAACTAGGGATATTGAATGCTCAGGGTTTGATGCCAATGAAGACAAAAGATGGGAGGGGGACAACGGATGCTTATTGTGTAGCAAAATATGGGCAGAAGTGGGTCAGGACAAGGACAATCATTGATAGCTTTGCACCGAGGTGGAATGAGCAATATACTTGGGAGGTTTTTGATCCATGCACTGTCATTACAATTGGTGTATTTGATAACTGTCATCTGCATGGTGGTGACAAGGCTGGAGGGGCAAGAGATGCAAAGATTGGAAAGGTACGGGTTCGTCTTTCCACCCTTGAGACTGATCGGGTCTACACACATTCCTATCCACTTCTTGTTCTTCACCCGAATGGGGTGAAGAAGATGGGTGAGATTCACTTGGCTATGAGGTTCACTTGTTCATCTTTTGTCAATATGATGCACATGTATTCACGGCCCTTGTTGCCAAGAATGCACTATATACACCCATTGACTGTTAGCCAGCTTGACAGTTTGAGGCATCAAGCTACTCAGATTGTTTCAATGAGACTGAGTCGCGCTGAGCCACCTCTGAGAAAAGAGGTAGTGGAATATATGCTGGATGTTGGTTCTCACATGTGGAGTATGAGAAGAAGCAAAGCTAACTTCTTCAGGATTATGGGGGTTTTGAGCGGCCTAATTGCTGTAGGAAAATGGTTTGATCAGATTTGCAATTGGAGAAGTCCAATCACTACAATTCTGATCCATATCTTGTTCATAATATTGGTTATGTATCCAGAGCTTATCTTACCAACAATTTTCCTTTACCTCTTCTTGATTGGAATTTGGTACTATAGATGGAGGCCAAGGCACCCTCCTCACATGGACACGCGTCTCTCTCACGCGGATTCAGCGCATCCTGATGAACTTGATGAAGAATTTGACACATTCCCGACCTCCCGGCCAAATGACCTCGTGAGGATGAGATATGATAGACTTAGAAGCATTGCTGGTAGGATTCAAACTGTGGTTGGGGACTTGGCCACTCAGGGTGAAAGGCTGCAATCTTTGCTGAGTTGGCGTGATCCTAGAGCTACATCACTATTTGTGATTTTCTGTCTGGTTGCTGCTACTGTACTGTATGTCACTCCATTCCAAGTTGTGGCCCTCTTTACTGGAATTTATGTTTTGAGACATCCAAGGTTTCGTTACATGCTTCCTTCGGTGCCGCTGAATTTCTTCAGGAGGCTGCCTGCAAGAACTGATTGCATGCTTTGA

>Glyma10G092200

ATGAGTTCTTCTCAAGCAGCAGCAAAGGGAAACCAAGAGGACTACAAGCTGAAGGACACAAAGCCAGAGCTTGGAGAAAAGTGGCCACATGGAGGGCAACGTGGAGGGAGTGGTTGGATATACAGCGAGAGAGCAACAAGCACCTATGACATGGTGGAGCAAATGTTCTACCTCTATGTCCGTGTTGTGAAGGCCAAGGATCTTCCACCTAACCCTGTCACAAGCAACGTTGACCCTTATGTTGAAGTGAAGGTTGGTAACTACAAGGGTAAAACTAGGCACTTTGAGAAGAAAACAAGCCCTGAGTGGAAGCAGGTTTTTGCATTCTCTAAGGAGAAGATTCAATCCTCAGTTGTTGAGGTCTTTGTGAGGGACAAAGAGATGGTGGCTAGAGATGATTACATTGGGAAAGTGGAGTTTGACATGCATGAAGTGCCAACAAGGGTGCCCCCAGATAGCCCTTTGGCTCCTCAGTGGTATAGGCTTGAGAATTCGAGAGGTGAAGCAAGGAGTAGAGGAGAGATCATGCTTGCTGTTTGGATGGGGACACAAGCTGATGAAGCATTTCCTGAGGCTTGGCATTCAGATTCTGCTTCAGTTAAGGGAGAAGGGGTTTATAACATAAGGTCAAAGGTTTATGTTAACCCAAAGTTGTGGTATTTGAGGGTTAATGTGATTGAGGCTCAAGATGTGGAGCCAAATGACAAAAGCCAGCCACCACAAGTTTTTGTGAAGGGTCAAGTTGGACAACAAGTGCTTAAAACCAAATTGTGTCCAACAAAAACTCCAAACCCTATGTGGAATGAGGATTTGGTGTTTGTGGCAGCAGAGCCCTTTGAGGAGAAGCTTGTGATAACTGTGGAGAACAAGGCCTCCCCCGGAAAGGATGAGGTTGTGGCTAGAATAAGTTTGCCGTTGAACAAGTTTGAGATCCGTTTGGATCACCGGGCAGTGCACTCGCATTGGTACAACCTTGAGAGGTTTGGCTTTGGTGTGTTGGAGGGTGACAAGAGGAATGAGACAAAATTCTCAAGTAGGATTCACCTAAGGGTGTGTCTTGAGGGTGCTTATCATGTGCTTGATGAGTCCACAATGTATATTAGTGACACAAGGCCTACTGCTAGACAACTTTGGAAACAACCAATTGGGATTCTTGAAGTGGGGATATTGAGTGCCCAAGGGCTCCAATCTATGAAGAAAAACAATGCTAAAGGGTCAACAGATGCTTATTGTGTGGCCAAGTATGGTCAGAAATGGGTGAGAACTAGGACTATCACTGAGAGCTTTAATCCAAAATGGAATGAGCAATATACATGGGAAGTGTATGATCCTTGCACTGTGATAACTTTTGGGGTCTTTGACAATTGCCATTTGGGTGGTGGTGGTGGAGGGCAAAATCAAGGAGCCAAAGTTGACTCAAAAATTGGCAAGGTGAGGATTCGTTTGTCAACATTGGAAATGGATAGGATCTACACCAACTCATACCCGCTACTTGTTCTGAAAACCTCTGGATTGAAGAAGATGGGGGAACTTCAATTGGCGATTCGCTTCACGTGTCTCTCCATGGCTCACATAATCTACCTTTATGGACACCCTTTGTTGCCAAAAATGCATTACCTACATCCATTCACTGTGAATCAGTTGGACAGTTTGAGGTACCAAGCTATGAACATTGTGGCAGTTAGGCTTGGGAGGGCAGAACCACCCCTTAGGAAAGAGGTTGTGGAGTACATGCTTGATGTGGACTCTCACATATGGAGCATGAGAAGAAGCAAAGCCAATTTCTTCAGAATTGTGTCACTATTTTCAGGTGCAATATCTATGAGCAGGTGGCTTGGTGAGGTGCAACAATGGAAGAATCCAGTCACAACAATCCTAGTGCATGTCCTCTTTTTCATCTTGATATGCTACCCTGAGCTTATCCTCCCCACATTTTTCCTCTACATGTTCCTCATTGGAATATGGAACTTTAGGTTTAGGCCAAGGCACCCTCCACACATGGACACAAAACTTTCTTGGGCTGAAGCAGCACACCCAGATGAACTTGATGAGGAGTTTGACACTTTTCCCACTTCAAAGGCTCAGGATGTGATCAGAATGAGGTATGATAGGCTTAGAAGTGTGGCTGGGAGAATACAAACTGTGGTTGGAGACATTGCAACACAAGGTGAGAGGTTTCATGCATTGCTTAGTTGGAGAGACCCTAGAGCCACAAGCCTATTTATGTTTTTCTGCCTCATTGTTGCTGTGGCATTGTATGTGACACCTTTCAAGGTTGTGGCTTCAATTGCTGGAATTTTTTGGCTCAGGCATCCCAGGTTCAGAAGCAAGCTACCCTCAGTGCCTAGTAATTTCTTCAAGAGGTTGCCATCTCATGCTGATGGCATGCTTTGA

>Glyma11G024300
[truncated: 1,022,282 more chars]
