# Supplementary material for: Characterization and phylogenetic analysis of multiple C2 domain and transmembrane region proteins in maize
Source: BMC Plant Biol. 2022 Aug 3;22:388. doi: 10.1186/s12870-022-03771-x (PMC9347167; doi:10.1186/s12870-022-03771-x)
Supplement: Supplementary file 2 — Additional file 2. [file 12870_2022_3771_MOESM2_ESM.doc]

All the promoter sequences of *ZmMCTP* genes.

>ZmMCTP1

ACATGTTGACTCGTACCTAGTCTAAAGGAAAAGCCCACATTCTCGCGGGTGGTCTGGAGCTAGGTAGCCGCTTAGCGTGGTCCCGTACTGTAAGCCTACACCTTCCACTACCCTAGAATAGTTGACTTAGTACTTTTCGGACTAGGTACTTGAAAGGTCTACGCCTCGTTCAAAATCAAGGGACGGGTCCCATAAACTCTGCCTGTCAAAAAATCGGCTACATTGTTCGATGGAATGAGTACAACAAACCTAACATCCTAAGCGTACAACCGCTAATCACTATTGCTGATCCATTCTTGGAATACCGTATGCTCCTAAGCACTATTCCTATGCCAAACTCAGAAGTATCTCCCTGGACCAAAAGCAACCCCCACGACTGGGAAAAACTCTAGGAAGCCTTAGCGACTGGGGGGCGAGAAAGAAGAAAACCCTTGTCTTCCCATAATTGGATAATCATGAGGGTAGAACCACTTTCTTTGTCTTCATGCAACAATGATAGTGTGTCGACAAGCAACCCACTCAACGTGAAGGCTACAGGCACGAAGCTGCTACACAATGCTGAAATTTCTGGAACAAGACACATGTATGGCCCCATTAGCGGGTTCGTCGCCTCACCTGAGGTGGGCCTCGGGGCCACTGTCGGTACCCCGAATATGGGGTACCCACTCTTGCTATGTTAAGGTGCGTTCGGGGGCCACCGTTAAGGATGTCGTGGGAAGCCTACCCACCACGAGGCTAAGGCCCACCGTATGGAGTGATGACCAGGCCAGACCCGCCTTCTAGACTGGACCACTGTCTCCATCAAGCTCCTTAAAGGGAGGAATCAACAACCTCAACTATAGGTATGGGAACCTGCAAATAGGACATGGACCTCCCTGTGTCTCAACGGATCCCTAGCCAAAGGGATCCGGAGGAGACATGGGAGAAGGCACTCCCTGGTTACAACACCAAGTCTTCAGAATAGCTCGGGGCATGAAAGATACCATCTAAACCTTAGGCCAGGTGAGGTTCGGAGTCACCACATTTCTAGGTTGGGGTTCCAGTAGCCCGGATCTCCCTAGGTGCGTCTCGAGCTCATGGTCAAGAAAGACTTGGAAAATAGTGTCACCACTTATCCCCACCCAGCGTGGATGTCTGGTGCTAACACGTGGCATACTTGGCCGCATCAGTAGCCGGTAGCGTGTCCCAGGCGTTAAATGCACACAACGTCTTAGGCCTGTGACGTACATGTTTTACTGTTACCTGCATGTTAATGAGACAAGTGTTTGACCCAATGCACCATTTGGGCGCCCTGCATTATAACTGGTACATCACAGAGACACGAAGAGATAGGTGGAGAGGACTAGACAGTATCGCCCGTCTACGCAGAACAACATATCAGATTACGCCGCAGCCTACATCAAGGCCCGTATTCCGATTAGACGGGACAAGATGGTAAGATAAGGACGCCAGAAGATATGCAGCGCCCACGAAGGGATACAAAATCTAGCATTGTGAGCTGAGCCCTATGTAGTTGTTTTGCACTCTATATTGTAAAATCTCCTAGGCCTATTTATAAATTTTAATTAATAAATCTTAAAAAAACATACATCTTATCGTAATATGTTTACTCTGTAACAACACGGTTTAGTCATATCTGACTTGCATATGTGGAGCTGAGGCCGGATTGGTATTAGATGCACGCGTCCATGGTTTTGATTTTTGCTTGGACGGCAATGCACTCCACCGAAACACAAAGCACAACTCTACGGAAAGCCTAGTAGATTTGGATGCACGCCACGTAAAAATAGGGAAAAGGGCACCGGTACGCCATATTGCCGTCGGTGGCAGTCGGCAGAGGATGGCCGTCGTAGGATGGCAGTCATCGGCCGTAAGATTTTGCAAGGCATTTGACCGTTGGTAATAATTCATAATAATCACGGCGTACTCACACCAGGTCAAATTCCGCGGGCGTCACGTCATCAAACCCACGCTTCCAATTCTACTACGGAGGAGAGGTA

>ZmMCTP2

CGAGGTCGACGTCCGGCGTGCCTTCCGTCGGGGCGTCGGGGGCGTCGATTCGCTCGACGGCCGACGAAGCGCGGCCTCCCGTCTGGCCTTGACGGCTCCGCCTCCTCCTCCGTTGGCGGGGGAGAGAACGGAGCGAGCCCGAATATTGCTGTTCCACCACGCGGGGAAGACGTCGTCGATTCCACCGCCGGCGGGCGGGTTGTCGGCCGCCATTGTCGTAGTCGCGCGGCGGTGGAAGAAGTGTCATGTCGTAGCTGCCGTCGAAGGACATGAACTCGAGAGTCCCGAAACGAAGCACCGTCCCGGGCCGGAGAGGTCGCTGGAGACTGCCCATCTGGAGCTTGACGGGGAGCTGTTCGTCAGCACGCAGCAGGCCCCTACCTGGCGCGCCAACTGTCGGCGTTTCGAGACAGGGGGGTCCCTAAGCCGACGAGTGAGTGTGCTGCGTGCCCCAGCCCAGATGGGTCGAGCGCGTGGGCGAGCGCGAAGGGGGGAGAGGCGAGGCGGCCGGAGCCGAGCGTGAGAGAGGTGGAAGTCCCGCGGCCTTCGTGTTCGTCCCGCGCCCAGGTCAGGTGCGCTTGCAGTAGGGGGGTTACAAGCGTCCACGCGGGTGAGGGAAGCGAGCGGCCCCAAGAGAGCGCCTGTCCCGTCCTCGGTCCCGTGCGGCCAACCTTCTCTGAGAAGGCCCTGGTCCTTCCTTTTATAGTCGTAAGGAGAGGATCCAGGTGTACAATGGGGATGTAGGAGAGTGCTACGTGTCTAGCGGAGGGAGAGCTAGCGCCCTAGGTACATGCCAATGTGGCAGCCGGAGAGATCTGGGCACCCTGCTGGCGTGATGTCGTGGCTGTCGGAGGTGCGGCGGAGCCTGACGGAGGGATAGCTGTTTAGCGGTCGAGTCCCTGCTGACGTTGTCCTGCTTCCGTAAGAGAGCGGACGAATCTCAGAACGTCAGCCCCGTAATAACCGNNNNNNNNNNNNNNNNNNNNNNNNNNNNNNNNNNNNNNNNNNNNNNNNNNNNNNNNNNNNNNNNNNNNNNNNNNNNNNNNNNNNNNNNNNNNNNNNNNNNCCGAAGTCCGAGCCCTGAGGGTCGGGGCGGAGCGGAGTTCGCCGTCTTCCGGGTCTTAGCCCGAGTCCGAGCCCTGGGGTCGGGCGGAGCGGAGTTCGCCGTCTTCCGGGTCTTAGCCCGAGTCCGAGCCCTGGGGTCGGGCGGAGCGGAGTTCGCCGTGGCGCCTTTGGCAAGGCCTGATTGCCTGTCAGACTCACTCTGTCGAGTGGCACTGCAGTCGGAGTGGCGCAGGCGGCGCTGTCCTTCTGTCAGACTGGCCAGTGGAGCGGTGGAGTGATGGCGGTCACCTCGGCTCTGCCGGGGGCGCGTGTCAGGATAGAGGTGTCAGGCCACCTTTGCGTTAAATGCCCCTGCAATTTGGTCAGTCGGTGTGGTGATTTAGTCAAGGTTGCTTCTGAGCGAAGCCAAGGCCTTGGGCGAGCCGGTGATGTGTCCGCCATAAAAAGGGGGCCTCGGGCGAGACGGAAGTCTCTCGAGGTCGGCTGCCTTCGGCCGAGGCTAGGCTCGGGTGAAGCGTGATCGAGTCACTCGTGTGGACTGATCCCTGACTTAATCGTGCCCATCAGGCCTTTGCAGCTTTATGCTGATGGGGGTTACCAGCTGAGAATTAGGCGTCTTGAGGGTACCCCTAATTATGGTCCCCGACACCAGTATAAATAGGGATGACAATGGAGAATTTCCCGTTGAGAATAGCTCCCCATACTCGTTCCCACGACGAATTTCCCCCCGCGAGAATCCCCACGAACGCTTGCGGGGAAATTTCTCACCACCCCCGTTCCCCACGGGGATAAATGCTCGACGAGGATCTCCGTCCCGCTTAAATTATAATTGGGACATGCGTCCTTTGTCATTAATGCAAAATATTGTCACTTATATACATTGTTACATGTAAAAATTCATTATGTGTACATCAAAATAAACACATTTGTGCATT

>ZmMCTP3

CAGAACCGAACCTAAGATATTGCTCATCATGAGAAAGTCCCAGGGTTCGTTACTGTTTGGCTCCAAAGATGGGGTGGACATCATACTCAAAATCTTCAACTTTATTTAACCCTACATTATCTAAAGGTGCCAAGTACAGTCAGACAATTCATTCTAGTGTTTGGTGACCAAATCGGCGCCAACCGTCGCCTTTCAGCACTACAGTGATAATAATATCCAGGCCTCATGATTTTTCTTCAAAGTAAAGAAATACAATCACATATTTTCGGACAAGCGGTAGAGCTGGAAAAAAAGTTCGAGGCTCGTGAGCCGGCTCGGGCTCACTCAGGCGCAACTCGGTTTGGAGAGGCTCGCGAGCCTGAACAAGCCCGAGTCGAGACTGCTTTTGTGGCTCGATCAAGTAGCGAGCTTAGCCTGTCAGGCTCTCGAGACGTGGCCAATGTCACGTGTTATATTATTACACCATTACATTAATATGTTATCAATTTATTTATTTAATTTGTATGGACTTATTGCTAGATATAGCTTAAAATATGTTGGCAGATTTTTTTGCATAGTTTTAAATTTTATATATGATAAAAATATTTTTTTTGCTGTAAATAGGATTTAACATGGCGACTCGCGAGAACGAGCCCGAGTCGAACCACTTTTTTCCATCTCGCAATTGACCGAGCCGAGCTCGTTGGTTTACCGAGCTGAGCATGGCTCGTTTCCAGCCCTAAATGGGAGCATGTAACCTTTGTAAAGCAGGCATAAACTATACTAAGAGCAGTGAGCAAAACGAGAGGAAAAGAGGCTCCTTGCCATGCTGAAACATTAAGAAAGAACCAAGAAACCAAGGGACCAGCAAGGTCCCAAGGCGGCAAGGCCTGAATGAAGATCGCTTTTTTTTTCTTTTTGAAAAAAAGAAAACAAGACCCTACACTGAGCTGGGGTTTGCTGCCGAACGTGAGCGAACAAAATGTAAATGCCGTAAGCCCACAGCACACCGTCATTGCCACCGGTCAATAGTATACTTCAATTGCAACGAGGAGATTAGGCCCTGTTTCAATCTCACGTTTAGCTTTCTGCTAAACTTTAGCTATATGAATTGAAGAGCTAAAGTTTAGCTTCAATTACCACCATTAGCTCTCCTGTTTAGCTTACAAATGGCTAAAAGTAGCTAAAGAAAAGCCGCTAAAGTTTATCTCGCGAGATTGAAACAGGGTCTAAAGCTCCAGTGTGAAAAATAAAATAAGAGAAAGGGGTTTCTTTGGTTAATAAAGCAAGCAAGGCCACAAAAAGCCTCTGAATTGCTTTTGCTTTCTAGTTGAATACGCACTTTGCTCTTTTTCCCCCTCTTGGTTCCATCACGTAATAATTGAGTATATATCCCAGCTGAGAACCTTTTGTTTTCTGGATCACATGTAAACGTTGTTATCTGATTTCACCCCCCTCCCCCTACTACGTTGAAGACCAACTTCCGAATTCATGAAACATTACATGACAACATTTTTTTATGTATGCATATGGCTAGTTTGAAAACTCAAATTCTCTTGAGTCAATGATTTTTTAAGGTGAGCAATCCCATAAAATATCATTGAATAAGACATCTAGTTATTTATTATAGGAAAATTTCTCTCTCTTCGCTTATAGAATTCAATCTTACATGTTACAGACGAGCTAGGCAGCTAAACACTTTACCTTCCAAACCCGACAATGTTTGTTTCATTTCATAATTTCCATTGACCGGAAAAAAATTCATAATTTCCACAGATAAGTAAGTATTAAGAAAAGATCCTTGACTGTCATAGATGCATCAAGAATGATGATATCAACTTTAATTAAGATCAGCATTGAGTCTTTCATCGCCAGTGATTATTTACTCGGGCAGAAATACAGGATACAGAAGCGAATCAGCAGATACAGGCATACCGCATACAGCCATACAGTCAGCTTCACCAGCACTCCTCTTCCAGTCTTTCCTCATCCTAGCTCCTACTCAAATCCAAGCAGATGCA

>ZmMCTP4

CCTAAGTACATGCCATCGTGGCAGCCGGAGAGGTTTTGGCACCCGGTTCGTGTGGTGTCGTGGCTGTCGGAGGAGCGCTGGAGCCTGGCAGAAGGACAACTGTCGGGGCTATCGGGTCCTTGCTGACGTCCTCTTGCTTCCGTAAGGGGGCTGAGAGCCGCCGTCGTCATGGAGCATGCGGGACGCCATCATTACTTGTTTAGCGGAGCGAGCCAGATGGGACGCCGGTCTTGTTCCCCGTAGCCTGAGTCAGCTTGGGGTAGGGTAATGATGGCGTCTCCTGTGACGTGGTCGGTCCGAGCCCTGGGTTGGGCGAGATGGAGGCTCCTCCGAGGTCGAGGTCGAGTCTGTCTTTCAAGGCCAAGGTCGAGTCCGAGCCCCTGGGTCGGGCGAGGCGGAGACCGTCGACTGAGGCCAGGGCTGAGTCCGAGCCCTGGGGTCGGGCGAAGCGGAGTTCGTCGTCTTCCGAGGCTGAGCCCGAGTCCGAGCCCTGGGGTCGGGTGAAGCGGAGTTCGTCATCTTCCGGGGCTGAGCCCGAGTCCGAGCCCTGGGGTCGGGCGAAGCGGAGTTCGTCGTCTTCCGGGGCTGAGCCCGAGTCCGAGCCCTGGGGTCGGGCGAAGCGGAGTTCGTCGTCTTCTGGGGCTGAGCCCGAGTCCGAGCCCTAGGGTCGGGCGAAGCGGAGTTCGTCGTCTTTCCGGGGCTGAGCCCGAGTCCGAGCCCTGGGGTCAGGCGAAGCGGAGTTTCCTGTGGCGCCTGAGGCCGGACTTGGCTGCTGTCAGCCTCACTCTGACGAGTGGCACAGCAGTCAGAGCGGCGCAGGCGGCGCTGTCCTCTTGTCAGGCCGGTCAGTGGAGCGGCGAAGTGACTACGGTCACTTCGGCTCTGTCAACTGGAGGGCGCGCGTCAGGATAAAGGTGTCAGGCCACCTTTGCATTAAATGCCCCTGCGATTTGGTCGGTTGGCGTGGCGATTTGGCCATGGTTGCTTCTTGGTGAAGACTGGGCCTCGAGCGAGCCGAAGGTGTGTCCGTTGCTGGAGGGGGTCCTCGGGCGAGACGTAAATCCTCCGGGGTCGGCTGCCCTTGCCCGAGGCTGGGCTCGAGCGAGGCGTGATCGCGTCCCTCGAATGGACCGATCCTTGACTTAGTCGCACCCATCAGGCCTTTGCAGCTTTGTGCTGATGGGGGTTAACAGCTGAGATTTAGGAGTCTTGAGGGTACCCCTAATTATGGTCCCCGACATGTGCATATTGTTATAACCATCATGTGAGATGAACAAATAACCAAACAACAAAAATAAACTTTGTAGATCTTGAGAAGTTATAGAATTTTGTAGTCGGCAACCTTTTCATTTAAAGTCATCTTATCGTATGAATTAAAAAAATTGAAAATTTTAATTTTGAAAACGACCTCAAAAGAAAAAACCACCAGCATGAAAGTTGTAGATATTGAAAAGTTATGAAACTTTGTAGTTGACAATGTTTTGATTTGAAATCATCTTGTCATGCAAAACTATGTTTGAATTTTAAAATTTGATTTTTTCAAACTACCTCGAATGGAAAAACCACCAAAATAAAAGTTGTAGGTCTTGAAATGTAATGAAACTTTATAATTGATAATTTTTTTATTTGAAATCATCTTATCATTGAAAATTCGTGTGAAGTTTTCAAATTTGAAATTCAAATGTTATAAACGACCTCAGATATAGAAACTATCAAAATAGAACTTGTATATCGCGAAAAGTTATGCAACTTTTTAGTTGGTCACATTTTCAAATGAATTTATTTAGTGCCTTAAATAATCAAATTACTTTCGGTTTGTTATAGTACATGAGAATGAAAACGTAATATAGACATAATTGATGTATTAGTGAAGTGGTAGAGAAAGATATGTGCGAAAACAGGTTGCGAGTTTGAATCTTACCATTCACAAAAGCATGTAAATTTGTTTTAAAATGTGGTTAGACAGAGTTGTTTCTATAATTTTAAAAAATATTTTGCTG

>ZmMCTP5

ATAGATGCGAGGACTAGACCAAAGAGGATGGAAGTTGTGAGAACTGCTCAGGGCAATTTTAGCCTATCCAAACCTTTTTTATATACTTTACAGTATGTTAACATTAGGTATAAGAGGTTATAAAAAATTGAGGGGGTCATTCTATGGTTCCGACACGATCGAAAGAATGAGACTGAATGAATAGACGTGAGGATTAGACTAAGTAAAAGGACGAGAGGATAGAATTAGACTAATTCGACGATGAGACCCATAACACAATATTTTAAATATACTTAACAGTAAATAAACATGATATATAAAAGGTCATAAAAATTGAGGGGACATGGCCCACTTTACCCCTGGTTCATAGACTCATAGTTAAGCAACAGTCTTTACTATGGCCCTGTTTGGGAGGGCTCTGGCTTCTCTAAAACTGGCTCCAACCGTTGCTTTTTTAAAGGAGCAGCTTCTCTGCTAGAGCCGATGTCTTTTACAAATCGTTTGACAAAACGGCTCTTTATTGAACGCTAGTGAGAGGCTGCGCGTGGTGGGAGAGGCTGCACGTGGTGCCTGCGCACTGGGATGGGCGAGGAGCCAGTGCGAGAGCAGGTTTTTTGGGCTCCGGCTCTTAGGAGCTAAACTGGATTCTGCTCTCTTTAAAGAGTCATTTCAACAAACTTGTTTGGGAGAGCTCCGTCGCGGAGCCGGAGCTGGAGCCACGGGAGAGCCGTCCCAAACGGCCTCTATATTTGCTATCGACAAGTGATTTTTAACAAATTCCCGCAGCAACGCGGGATATCACCTACTTCAACAAGGCGCGCTAAAACGTTCTGTACGTTAAATATAGCGGACGAGGACGTACGCTACGTCCCCAGCAAGAACCGGTAAAGCGCACGCTAAATTATAGCGAGCCTTCATCGTCCGTCAAATGTGGCGGACTCGTTATCGTCCGCTATCGTCTGTTTGCTCCTGCGTCGCCGCCTATCGGTTCACTCCCGCGCTTGCTTGGAAGTCACTCGTCGCTCCGTTCTCTCCCTCGCTTGCCCAGAAGCTTTGAGTCCTTCAGTGACCTGCACCGTCGTGAAGATGAAGGAGTCGTCCGCCTCCGATGGTGGGAACCGCCGCACCAGCGTGTATAGCCGGACGATGTCCCTTGGCCCGCCTCCTGACAGCTCGTCAAAGTCGTCAGAGCTATCAGCGACAGTGAGCTTGACGGCCTCGTCGTCGTTCCCATTGGTGCCCCCTCCTACGGAGTAGAAGAAGTCATCCTCGCCGCCGTCGTCCAGATCACGCGGGCTAGTGCGACCAAACATCAGGTTGGTGCTCGTTGAAAATGTCTGGGAAGCAGCAGACATGAACGATGATATGTCCAATATAGAAAACAGGGTCCGCTGATTTTTTCTTAAATATGTAATAATATTATTTTCACCATAAAATCAATATGCATTGTGTAGCGTATTTAACATATGAACTGTTTTTAAGCGACCAATGGAAATAATAAAATCTGGTAACACTGTAGATATAGAGGACTTAATTTAGAGGACGTTGCTGGAGACGATGAAAATATAGAGGACGGAACCTTTTAGAGTGTGCTGTAAAGTACGAGGAATATTCCTTTAGAGGATGAAATTTAGGGGATATTGCTGGAGACAGTCTTAGGGCCTCGGTTGTAGCGGTTCCTGTGTACTAGTATCATTTTGCAACCGACCGTACTCCTAAGCGATGGACCAAGGTTTAGACGTCCTACTAATAGGCCAGACGCTAGCTAACAAGGTAGCTCATCTTAGTTGATCTTCCCACTCACAGTCTCACACCCACCTTCACCTCTACGTTGCTGAAGTCAAGCCCCATGGTATTGTACTCGAACTGAACCCGGCAGATCACCAACGTCCCCCAGCAGACACACCAACTGGGTCGATCGCCAACCCAAAAAGAGGCCATTCCTATCCTACGCGGCCAGCACGCTCAACGTCATGATCGTTCGGTGGCCAGAAGGCACATAAGAGCTCCTTCCTCACGCA

>ZmMCTP6

TCCGTTGTTCCCTGCAGCTGACCCTACAGAGTTCACTACTCCTGTGAGTATCAAAATTTTAGTACTGCATGATATATATTCATATGTTCTCACACATACAATCTCTTCTCTGTGCAGGGTCAATCGACGGCGTCGAACAACCCTCATGCTTCGTCCAGCCCTTCGTCGAACCAGTCCAGATGCCCACCTCATTGATGATCTGTTTTGTGATGATCCAGACTTACCTTTATGAGTGTTGGTGATGTTAGTTAGACTTTATCTTGTGAGATACTTGGTGATGTTAGTGATGATGTAGACTTATATCTGTTTTGTGATGATCCTGACTTATATTTGTGAGACTTTGTGATATATATGGTGTTGATAATAAATGTGTTCATTCTGTGATGCGATTGATATATAATGTGATGTGAATGATATATATCTTTTGTATGTTTGGATGGAACAACAAAAGCAAATAAAAAAGGGACATCCTGGTCACTTTGCCGAGTGTAACACTCGGCAAAGGGCCGCTTTGCCGAGTGCTATGACCATGGCACTCGGCAAAGAAGAAAACCTGGGAACCGGTAAAGCCATCTTTGCCGAGTGCTGACAATGGCACTCGGCAAAGAAGGAAACCTGGGAACCGGCAAAGACATCTTTGCCGAGTGCTGTTGCAAGGCACTCGGCAAAGATACTGGCAAAGGGGCCCACTGGAGGGTTCTTTGCCGAGTGCCAGTCCACCAGACACTCGGCAAAGAAGCCTCCTTTGCCGAGTGCCTACTAGAGCTCTTGGCACAGGGACTGGCGGTGGGGCTCACTGGACCAGTCTTTGCCGAGGGCCTCACACTCGGCAAAATTGGCCTCTTTGCCGAGTGCCACAGAAGGCACTCGGCAAAGGATACGTCACCGTCACTTGGCGCCGTGACGGTTACTTTTCTTTGCCGAGTTCCAGATGGCACTCGGCAAAGTCTTTGCCGAGTGCCCGACAAAAAGTACTCGGCAAAGAGACTGTTGCCGATGTACAGTTCGCCGAGGGTTCTTTGCCGAGTGTTACACTCGGCAAAGGCTTTGCCGAGTGTAAAATAGTCTTTACCGTGTGTTTCAGACACACGGCAAAGGAGCTGATTCCGGTAGTGAATTCATTGTAACTGAAAATTACCGTTTTCGTTTCACCCCTAATCGAATGCATTGCGCCTCAGCAACGTCACAACGCAAAATGATGCATGTATGCTTACAACACACACGGGCACCAGTGCACGAGCAAGTGTAAGACCAATGGGTGGTGACCTGGTCACACGAACTTAGTTTAGCATTGCATAAATGAACACCAGATTCGGATGAAATTTCCTATATTGTTTTATATCATCAATTGGGTTTCTTGTGTCAACTTGCACTTGACTAACTTCCTTTCTATTCGCCAATTAGTCATCATCGCCATTTCTAATCTTAGCCAACATGGCAATACAACTATGCTTGTGTCACCATCGCAGGCATATATTTAGCCAATGGATTACAATTATACTAAATACATGATCAATTTGCACATGAAAAGGACATCCTCTCTAATCAGGCTACGAAATCGGAACCTGACTGAAACAGGACTTGGTAAAATTTAGGCGCTTGAATTTTAGTTTTATTTTAGATGTTGATTATTACATATGTTTTATACTAAATTTATAGTTTTAGTTCAATTTGGAACCAATAGGTTAGGGTATCAATTAGCTGGGTACTAGTTGAGAGTGTTTGGAACCTTTGCAGCTAATTTTCGCCGCTGTCGATTAGCTCTAAAGTTTCCAAGCAGAGTAATAATAACATTGATTTATTATAACACGAAACTAATAACAAATCATAAAAGTAATAATATGCTTCCATCAGCTAATGATATAATAGTGTTAACTAAATTTTTAAAATTTTGGCACAAGAAATTGAAAGTGTTCAGTGAACGAAATATACACCATGCAGAAGTAGGTAACATTTGACATTAAACGGAACGCTTCGAGACTACTCTCGTGTACTACAGC

>ZmMCTP7

ATAGTGCACTATGGTTTAAATATGTATGGGGAGAAGCATGCTTCTGTTGTGGTGTGCAATAAATTGGGGTATGATGTGGAACAGTAGGCTCTACGTATGCGTATCCGGACTGTTCGGACCAGGCGGTCGGACGGTCCGACGCTAAGTCGGACTGTTCGATGGTAAACCTGGACTACTCGGTTGTGTTTGCTACTATCGAGGCACCACATTGTGGTCCTAGTGTAGCCCCAAACTGTCTGGTAGGGAAATACGGACGGTCCGCCTAAGGATCAGATTGTCCAGGAAAAGACTCGGATGGTCCGACCGTGTCCAGGGCGCCGATTTGCCAAGCAGGGATGGCGGTGGTGGTATTTGCCCTGGATATGAGTTCATCGGCAATACCATATAATGGTTGGGGCTGCGAATCACCATTTTTTGTTGATGTAATAGACATATACATCATGTAACTAGTTTCAAATGATGGAAAACTAGAAGCAACATATTTCTTTAATGAACATGTTAGTTTTATAATTGAGTCTTCTAACCCTCCAATATGTTGTTTCATTTTATTCTGTTGTTGATATACATATTGTTTAATAGATTAGATGTTGTCGGGTTTACTTACGTTGGGGATTTGAAGAGAAGATAGAAGATATGCGACATCGATCTCTCCGTATTTGATAACCTTCTGGTGGCGATCCACTATGAATTGTGACAAGAATTTCTCCTTCGCTTAACGCATGTAGTCTTCGTATTGTTGTTGCTCATCGGCCGACAGGCTTTCGATAGCCGGTTTTAGGATATTGACTGGGGAGATATCAGTGTGATTTTTAGAACCAGTCATTTGAGGATCTGATTTTTAGTAGATCTAGACACCTGGTCCCCAGCGGGAGTCGCGCAAAAGTGCGTTGGTGTCGATTTGGGCGCCAATCACCAGTAGATTAACCGCCTGATGGTGCTCTTTGCGGAGGCGAGCACGGTCCGCGGCTCTGGGCCGGACGGTCCGCGACTCCTTTTCTGCGTATGTCCGAACAGTTTGCGTCTGGGGCTCGGACGATCAGCAATGGTGCAGAGGGTAGTATTCTTCGCATCAAATCTAGATCTGGCCTCCCGGGAGCAACCCTTTCTTAGAGGAGAGATCCTAAGGTGTGTCTTGGTGTCGGCAGGCCACCCAAGATGCATCTAGTCGACGTAGAGCCGAATAGAGATGAAGATTTGAGGTAAAGAAAGGCTAAACTAGGGTTACTCCTAATCCATAAGGTAAAATGATAAGTAGAATTGATTGGATCGATTGTGGGGGTTCAATCGTACGTATCATTTCATCTATATAAAAGGATGGAGGTATAGACCCGTTACAAGTTGGTTTTCAAGTTAATCCCACATGTTTAGATAAAAATACGGTAATAAATTCAAAACTATAGCTGATTCTGCACACGCAGATCGTCCACATTCCCCTGAGAATAATTCGGACCATAGACCATCTTCAGGTGCGGACAGCAGTACAACACTGCTGTGGATCACAAGGCTCCACACCTCACCAACCACCGCCATTGCTGGGACGGTGGCACCGGGAGAGTGAGCTACAACAGCAGTGACCGGGACGCAACGGAGTACAACAGGACGTGCGGCGCGACAACCCCGAAAAGGATCTGGACGCAAAACACGGCACGCGAACAGGAACAGGCTCCCCGCGAGGCGATACCGTGCACGCAGCCCGGGGGCGCGGGATTTCTGCCGCCGCCGTTGCCGCTCGTGGTCCGTGTCGCGTCAATGCCGTTGCCGTTAAACGTCCCTGTCGGCTGTCGCGCTGCCTCGCCGCAGTCCTCCGATGTCGCCGTTCGCGTTCCGTCCGGGTCCAGGCATTCATCCACAGCTACGGGACAAGTCACCGTGCCCGCAGGCCGCAGCCACACGTGGGGCGCACCACCGTCCGACGTGGAACCTGGCCCCACCTCCCCCGCCCGTTGCCTACGGCCCGCCTACCCTTTTAAACCCCCGCACCGCCCGCCTACGCTACGCCC

>ZmMCTP8

TTCCGGGACTTAGCCCGAGTCCGAGCCCTGGGTCGGGCGGAGCGGAGTTTGCCGTCTTCTGGGACTTAGCCCGAGTCCGAGCCCTGGGTCGGGCGGAGCGGAGTTCGTCGTCTTCTGGGACTTAGCCCGAGTCCGAGCCCTGGGTCGGGCAGAGCGGAGCTTCCTATGGTGCCTTCGACCGGGCCTGACTGCCTGTCAGTCTCACTCTGTCAAGTGGCACCGCAGTCGGAGTGGCGCAGGCGGCGCTGTCCTTCTGTCAGGTCGGTCAGTGGAGCGACGAAGTGACGACGGTCACTTCGGCTCTGCCGGCTGGGGGGCGTGCGTCAGGATAAAGGTGTCAGGCCACCTTTGCATTAAATGCTCCTGCGATTTGGTCGGTCGGTGCGGCGATTTGGTCAGGGTTGCTTCTTGGCGAAGACAGGGCCTCGGGCGAGCCGGAAATATATTCGCCGCTTGGAGGGGGGCCTCGGGCGAGACCGAAATCCTCCGGGGTCGGCTGCCCTTGTCCGAGGCTAGGCTCGGGCGAGGCGTGATCGAGTCCCTCGAATGGACTGATCCCTGACTTAATCGCACCCATCAGGCCTTTGCAGCTTTATGCTGATGGGGATTACCAGCTGAGAATTAGGAGCCTTGAGGGTACCCCTAATTATGGCCCCCGACACATTTGTAGCTAGATTATTTAGGATTGAAACTTAGACTTTACGATAGAGATAGCCCCTGTTTGTTTCGGCTTTTCGCAGCTTCTGGCCACCAAAAGCTGCTGCGCACTGCCAAACACTCTGCTTTTCAGCCAGCTTATATAAAATTCGTTTGGGTAAAAACCATTCAAAATCAACATAAACATATAATCGGTTGAGTCGTCGCAATAGTAGTAAGCTGTCACTTTATAGATTCTAAGCCCCATGAACAACTTTATCTTCCTCCGCACGTAATTCTAATGATACTCAGATTCTCTACACAGCCATATTCTCTCCACAGCTAGATTCTCGGAAAAGCTAGTCAGAAAAAAGCTGAACCAAATAGGCACATAATAACACCTACGACAGTCTTTTTAGTTAGGTGAGACTAGCTTATTGATTTTTAAGAAACTAAAAATACAGTTTTTATAAATTAGGATATAAGCTGTTATGTTTAAAATCATCTCGGCTTTTATAAACTAATTTTTTAAAAACTGGGTGCTTCCAAACGGCCCCCAACAACGCAAGACTTGAGCAGAAATGTACACCGTGCTACGAATGAGTCGTAAATAAAGCGCTTTTGATATTACGAAAAGCGCAAAACGATATTTTGCACCGCTAAAAACGCAAAACAATATTTTGCACACACATGTACGCCGTGTGCTCATTTTATCTTCGCTATCTTATCTCACAGTCTCATCCCCACGCCCATGGGCCGCCACCAAAAATCAATTCAAGTAACGCCATTGGAGCTAGCGGAACAGTGGCATCCTATGCGGGATGAGCAGTGACGTCACCACCCTTCGCCGCCCATCTCCTGCAAACAGTCAACAGAGAGGGGGTAGGCCGCAAACTGCAAGCACCAAGCAGGAGAAGACCGCAGCGCAGAGAGAGAGAGAGGGGTGGCAGCGAAAACCCAAACGGCAACGGCAACGGCAACAGAAACGAGAACCCAAAACCCGCGCGGCGCCTCAAAAACCACCCAAAGCGAAGAGCGCGGGGCAGAGAAACCAGAGCAAAGCAGCCCAGGCAGGAAGCAGAGAGCCACCACCACCGCCGCGGCGGCATCGGCGACACACTACGCTGCCCAAGCAGCGAGCAGAGCCCACACCGCCTCGGCACCTCCCCCATCCGCTTCCTGACCCAGCGCCTCATCGATTGCGATGATGAAGATTCCTTCACGCGCGCCTGCCCCCTACTCGCCGTCGTTCCTATCCGTATAAACCCTCCCCATTTCTCTCCTCCTATTTCCCCCCAACCCCAACCCCTCCCTCTTGGAGATTTTGGGGAAAATTTTGAATTTTGAATAGGAGTAGAATCGGC

>ZmMCTP9

GAGATTGAAAGGGAAATGGGCTTAAGACATTTCCTATAATCAATTTTGGTGTTTGACGTCCATCACAAACCACGTGGACTAACTAGTTTGCCTAGTTGTTATTTATCTCAGGTGCATAAAGTTCAACATAAACCAATAAAGAAAACGAGTTGGAAAACTCTACAAAGTTTGGAGTAAAAACAAGTATTGGTGCAGCTAGTGGCGCACCGGACACTGTCCGGTGCCTAGGCCGAGGCACCTCGTGAACTGGCCGCTCTCGGGTTTTCTGAGAGTCACTCCGCTATAATTCATCGGATTGTCCGGTGTGCACCGGATAGTGTCGGGTGAGCCAACGGAGCAACAGTCAACTTCTCCAACGGTCGACTGCGACGATGAGTGAACAGTGCACATAGCAGAAGCCAGAAGTCAGAACTGCAAAGTCAGAACGCACCAGACTGTTCTGTGTGCCACCGGACTGTCCGGTGCCGCTAGAGGACAAAGAACTTCAACGATCAACCACTCCAAACCTCAACGATCGGCTGGCGTGACACGCTCCGGACAATGAACATTGTCATGTCCGGTGCACCACCGGACTGTCCGGTGTGTCCATCGACAGGAACGACTGGAATAATGGTTGGGGCTATAAATACCCCAACTACCATCATTCAAGCCATCCAAGTTTTCAGACCTTCACATTCAATACAAGAGCAAAAGCCTACACTCCAAGACAAAATCAAAAGATCAAATCCTCTCTAAGCCTCAAAATCAACTCAATTGCTTAGTGACTTGAGAGAGGGTGTTTTGTGTTTCTTTTGTTGCTCTTGTTGCTTGGATTGGTTTATTCTTCTCACTCTAATCTTTCTAAGTGCTTTGTAAAGCTTGCAAGAGACACCTAAGTGTGTGGTGATCCTTGCGTGGTATTAGTGACCCAAGTGATTAAGGAGAAGCACTCGACCGGTCTGTGTGACCTATTGAGAGAGAGAAAGGGTTGGAATAGACCTGGCCTTTGTGGCCTCCTCAACGGGGACTAGGTTCTTTGGAACCGAATCTCAGTAAACAAATCGCTGTGTTCATTTGTGTTGATCTATACTCGATTTGTTTCCCCCTCCTTTCCTCTCTCTAAAGTTTCCTTGCTCATATTGATTTGAGTTGGCTCCCAAAGTTATCTGCATTGATTGAGCAACTCTTAGCAAGGAGAACTATCTTCCGCACTCCGAATTATTTCTAACACTAACCCCGAGTGTAGTGCGTGTTTAAAGTTTGTAAAATTCAGATTTCGCCTATTCACCCACTTTAGGCAACTTTCAACCTATCGCGACTAGGCATGAGTGCGCATGATGGTGGTAGAGCGCAACACGACAAGCCGTACTGAGCCCCAGGCTACGAAGACAAGGGGTTGACAAATTAGACATGCAAGATCTAGGTAAAAAAAAATTTGTGCTGAAGAGAGATCAAATTTACAATGTCTTATCTTGCACCTTCCTACGCTATAAGGTCCTGCCACTTATCTATTAGGCTGTTCGCAACCATCATATAAAATAAGAGGATTTATATTGTAGATGACACTGTTTGCAAAGTAAAATTTGAAATCTAAGATGTGATAAGAGATTAGATAAAATAACTGCTCGGGACAACCTTAAGTTGCAGATCCGTGAGGACCACCGTGCAAAAAAAGTTTAATTTTTTATGATAAGATCCCTGTATCCTATTGGTTTGACGGTAGACATATATTTTTTCAGTTCCAAGAATAATTTTGAAACTCTAAATATTTTTAAATATAAAATTGAAAAAGGTGCGGAAGAAGCCCACGACCACGGTCCAAAGCTAAAAAAAACGACAATGCAGATAGTGCTGGCACGGGAGCCTAGGCAGACGGCCTGTCCTTCCACCGAAATCCTACCAAGCACGAGGGTCTTCGCGGATAAGCGCCGACCGGGTAGGGGCTTTTGCCGTTTTGCGCCCCCAAGGCCCAAGCCAAGTCCCAACCCCCCCCCCCCCACCCACATACACTCTGGAGTCTG

>ZmMCTP10

CAAAAATATAATTCAAGAATTTCGGTGATACTTATCTACTACTACGCATTGTGCAAGGTTAGCAGGTGAGCTTGGGGAGAGAGATAGTAGATGTGTTTACTGTTATAGATGTAGACATAAACACGCATGTGGTGTAGTGGTAACTATTGTTGGTATTTGATTGGAGCGGTTGTGAGTTTGAATCCCCTTGGGGTCATTTGTATTTTTTAAATTTTAGCTAGACGTCGGCGGACAGAACACAGGAATGGACTATGCGCGGGGAGGAGCGAATCGGAAAGGCAGAACATGAAATAGCAGACTACTATTGTAGCCTTAATAAGTAGTAGAGATATCCTCTAATATAAGAAAAAATAATATTTTAATTAGAAGATAAAGATACTGTAGATCCACACCACTAATTGCCCAGTCGGCCAGTCTGATTACCAACATTTTTATTGGCCTTATTCCGCTCCATTTTTCCTTCCTGCAATGGCTGTACAACGATGGGGTCATCCGCTACCGGACATTACTTGGCCCCATTCCCCCAGATTAGATGCCACGGACTGGACGCATTGGAGTGGCGAGATGACATGGGAATATATTCTCCCCCCGGCTTTGCCTTTTGACACATTAATTTGAGAATAAAGGTAACTTCCGTCTGCTTCCTCGATAAGCAGCTATACTATGTCTCATTGTTTAATTATTAACCCTGCGCGCCAGGTGGAGATTGATAGCACCACTGACTGTTTCCATCCTTATTACGAACGTGTTGCCGCCACTGGACTGTGGAGCCGTCGCCGCCGCTCATCAAAAGAGAGGCGGTAGGCCACAACCAAGGCTAGTTTGGAAGCCATAAAAACGAAAAAGATTGGAGGGCTTAAAATTCCCTTCTGATTTTGTAGAGATTAAAGAGTCTAAAATTTCCTACCGGATTTATGGTTTTCAAACTAGTACTAAATTAGAGTGGCAGGATGAGGCATAGGATGCACATGCCACACCTGACATCGACGTCCTGTACACACTCAGACAGGGCGAGACCTAGGATGCACAGGCAGGTTATGAAGGGAGTTGTACGTCATATATCTGTCATATGTCTGTCATATGTCTATCGAGGGGAGGAGATACCGAGTAAAAAAGACAGATAGCCAATCTACTGAATGCGTTTTGAATCATTGAATCAGGTTTTTCTTTCTTTTTTTAATGGACTCTAAACATTTTATCAACTATAAACATTTGTTATCTGCCAAGCAAACACAAATATTATTGTGTCTCCGGGTTTCAGAAACCACTAAATCACGACACAGTTGGTTTACTAATATAGACAACATATATGTTATGAGTTTCGTTGACTGAAACTGAAACAAGATTATTATTTTTTAAAAAAAATAAACAGTAGTGTATCTGCGCCGCGCGTGCAGAGAAACATAACTTTGGTTTTATTTTAAGAGAGAGGGGGAAAGAATCATGGCTTTTTGACACTTGTTTCTCGACCATGTTGCTAGCTGTCTACGGAAGAGTAAGCAGCAGGAAAGCTGGAAATTTACCATGGCATCATGGGTACCAGTACCACTACCACAGCAGATGGGTCCTGGCCTGGCCCACGAACTCTGCAAGGTTCCTTCTCCCTTGCGCCCTGCTAGACCAGACCAGAGGACGAGATGTGAGCACGCCATTTCAATTCAATTCAAGGGCGCATGAACGAAGCCCACTCGGCGCTCCAAAGAATCGATTCCCACGACCGATCGATCGATCGCCAGCCAGCTTTTAGATTTGCCCCTGGCACTGGCAGGCACCGTCGTTCGATCGCCTTTGCACACATATGCACGATCCCTCGTGCTTTCCTCTCGTTTGCCAGCTTCCCGCGAAAGAACACTCCCTTCGGCTGCAGCCTCACGCTCTCCTCCTCTCAACAAGAAGGCGCAGGCAGGGGGGCAGGGTTTAGGCTAGCTAGCTAAGCTTGGTAGAGGCCTCAAGTTCAAGTATACCTTCCTTGTCGCCGGGTTCACCACCGACGCGGCCGC

>ZmMCTP11

ATAGAAATTACAGCTCCTTGTTTTTGTTGGAATTAGAATTTGTTTCAGTTCTGTTCCTAATACATATGGATTTTTGGTACAAGTATTGTTGATTTCTTTGGAATCAGAACCAATTAGGTTTTAGTTCATGAGTTAGGCACTGATTCTGTAAATTCTTTCTATTTGTTTTAGATGTCACCGAGCCGCTTCTTGTGGAAGTTGACCAAATCTATCACCTTGCTTGCCCTGCTTCACCAATATTCTACAAGCACAACTCTGTTAAGGTATGCTAGATCTCTGCTGCATGAGAGGTGGTATATTTTCATAGTTTTTCCTGATATTGGTGTTTGTGATTGTCTGTAACAGACCATCAAGACAAATGTTATTGGTACCCTGAACATGCTAGGACTTGCAAAGAGAGTTGGAGCTAGGTCAGTGTTCATCATGCCATTGATTCTTCCAGCGCCTCATGTGAAAAACTCAGTTACTGAATTTATTTTTGAATTGCAGGATTTTATTGACATCAACCTCTGAAGTTTATGGTGATCCACTTGAGCATCCTCAAATTGAGGCCTACTGGGGCAATGTTAATCCGATTGGTAAATTTGTTCATCTGAAATTTGAAATTTATTTGCTTTGGTTTTTCATAAATTATCTTTCAAGTTCTCTTACAGTTTTTATTTAACTTTTTTTTCTATCAGGTGTTAGGAGTTGTTATGATGAGGGTAAGCGTGTAGCTGAGACGTTGATGTTTGACTATCACAGGCAGCATGGCATTGGTAACACTTCATCTTTCACTTAAACTTCAATATTAGTAGCTCATAGTCCTCTTTGTTATGTGATAACTCTGGGCTTATGATTTTTTTTAACAGTGTTTATCATCTATGTCTGTAGAAATCCGGATTGCCAGGATTTTCAACACCTATGGGCCTAGGATGAACATTGATGATGGCCGTGTTGTTAGCAACTTCATTGCTCAGGCTGTGCGGTAATGCTTTGTTGAATGTCTTTTATTTTCTCCTCAGCTACTGCAGATATATTGGCGGCTAATCTTTTCACAATCTTTAGCGGTCAGCCCCTGACTGTCCAGAGGCCAGGAACACAGACTAGGAGTTTCTGCTATGTTGCCGATATAGTTTGTCTTCCACCGCGTTCTCAATTGGTTTGTTGTTGGTCAAAAGACTGTTAGTGCACTATATCAACTATGTCATTCATTGCACTATATCAACTATGCCATTCCACTGCATTCCCATCCATTTACTGTCAGTGCCACCCGTTGCAACGCACGGGTATATACCTAGTAGCTAAAGAAAAAGAAAAGGCGAAAGCGTGAAGCCGCAGCACGCTTGCACGTTCACGCTGCGGGCCTACCATCTCTAGCCCCAGATCGACGGATGACAGCACAAAAGCATCCGGCACAGCAAGCTGGCCAGCAACAAGGCCCAACCGACAGCGTCCGTTCCGTCCACATCCGCCCCCCTCCCGCCTCCCACACAAGGCAGAAGGCACGCCTGACCTGACCCCAGCAGAACCAAGATTTTCCATGCCGCGCCCCGTTGACACGTTTGCCCCGCGGTCCGCGGCCCCCGCATGTCGGTCGAGTGCGTCGTTGTCTTAAAAGTGTGTTTGGTTTGAGGTCAAAGTAGAATGAGTCGGGGGCGCCACCGTCCCCTCGATTTTTTGAGATCACGCCGCCCCCAAAAATCACTCCGGTGTTCACCTATCCCTGCTTGATTCTCAACCAAACACTATAGAAAATGTGGCCGCCTCATTCCATCCCTCTTTGTACATTCAACCAAACACATCCTAAACGGTGGCCGCAGCCCGCAGCCGCGGGTTAGTGATACGATTCATCGCAGGAAGATTCGGCCTGATGCGGAGACGTTGGATCGGCATCCAACGACTGCGTTCCGCTACGGTGCGCCGCCCACGGCGACATGCGCTGCATCCACACCACTCCACGCAAACACGTTGCGCCGGCAGGGGCAGCAGTAGCCCCGCTCCGCTCCGTTCACAACACT

>ZmMCTP12

TTTCCGAAGTAAAACAAAAAATCATCTTTCAAAACTAACCACAGAGTCAAATTTATCAGGTATATAGTGCATAGTTTGCTGCTTATTAATTCCTAATTTTCGAGTTGTGTGGCTAAAGTTAAAATAATGCAATAGTTGGATACTTGGATGTCCAAATGAAATTCTTGTGCGATGGAAATAAATCTACACATGTTAGACGTACTATATATTGTACACGTTTTACCCTGAAAGAATGAATATACGTTTGAATAGTTATACGGGTCTAATCTTTGTTGTGGCATGATTGACATGCTCTTTATTTACACAGCTTGTTCGCTGCGGCTTGCTGCGGCTGCAATCCGGCTGCGGCTGCGGCTTCTACAGTATTTTTCTCTCTATGGATTGCAGCTGCAGCAGTCCAAACAGCTGCAACAGTGTCGGCTTGCAGCCAACCGAACACGCCCACAATATAAAAACATATCATCAGCTGCTTGCGCATGAAGGTAAGCATATATATATATCTGGGTCTTTACTTCACGCCCTAGTACTACACCTAGCTAAGCCGTTCATCTCATGTATTATTGCCTTTAACTCTTTCTCCTTCACGTAAAGATGAAGCAAGGAATTGGCATCAGCTACTATTCCGCCTCTTATTAATCATTTCCAATGTTTGTAAACCGCGTGACTGTGTGCCATGAAATTCTCTCACAAATTATTGAGAGAAAAAAAGAGACAGAGGAATTTCTCTTCCTATCTAGCTAGCAAATATATATATTCCCCTATTTATAATGATGAGCAAATCTGTTCAATGCCAATGAAATTTATAACCTTTTTTATATGCCATTGAAAATTAAAAGTTCCCTTCTTGCTATTAAATTGCATTTTTCTTTTTCCTTATCTATACAACTATATAATTCACCAATTAAACTTTGCAAAACCCATCCAACCAAATCACCCCACACACATAACCTCCACGAAATCCACAAACACAAATTACATGCAGACTTAACACACCATCAAAACCAACGGTTTAGATCGTTGGATAATCTCATCCCTTACTCAAATCCAATGGACAAAACTATTTGGATTATCCCTCCCCCCTCTCCCCGCGTCCTCGCCGTCGTCAACCCCTCCTCCACCCTCCACAAGATTTGTAGCGTTAGCATTGGCTATATGCTAGCATGTCATTAGATGAATGCTACATGATGAAAATGACAATTATACCATATTTACTAGCAGAGATATAACCTAGAAAATGTAATGATTAGGATATGATTATCTTTTTATCCTCTAACATTTCTTTAACGCCCCGTTTGGATCACTAGAATTGAATTCCATTCTAATATAGTAATTTAAGCATATATCAATTAAGCTAATTCGCTTTTATACAAAATATATTTGTATACTATTATTAGCAAGATGTCCTAGATATTTATGTGCTACATTTTTATTATAGAGGAGTAGAACGAAGAGTGTCATGTAAGTTACAGATTAGAAACAAATTCTAATCATGCATAAAATCATTTCCTATTCTTCACCCTATGAATTTTAGATAGGCTTATATCTGAACTTTGGAAAGTGGTGGAATGCCAAATTCCAAACTAAATAAGTTACTTTATTGAGTGAATTCTAATTTCTTTAAAATGAAGGGATCCAAACGCCTCGTAACTGATTTTACGTGTCGATGACATACAAGAGAAATAAAAATACAATTCAATGACAATAAAGAAAGATCTTAATTTCTAGTGGTTTATACGAAAATAATATAGGTTTCGATAGCATTCAACAAATTTTCTCATTTATATATAGTACCCACTTTCACTTTTAAAGCCTTCTAACTCGCTACGCACCCACGCTCACACTGACCCATCTCGCTAATCGCCATAGCACCATATGCATCTGAAGGCTGAAGCTGCGAGGTGTCTCGCCGTGGTTGCACTTGCACCGACCGAAACAGCAGTGGATTCAACTTTGGCACGTTGCCGTGCTGAGCAGCTGGTGCCAATCGATCGTGAATCGTG

>ZmMCTP13

TTAGGATCTAACTTCAGCTTGGAAGGTTATCCACGTAGTTGACTGGTCGGCTAAGGAATACTGGTTTACTTATGAATTATTACCTTTGCTAAGATTTACCACAAGTCCAAAGCTGGTGTGGAAGACTCTCTGCTTTTGCTTACTCACCTTTTCTGTATCTCGTCTTTCTTTGCAACAGTAACAAGAGGTCTGATGCTGTCTACAACACATCTTTTTTTTATCATCAAAGTGAAGCGATTTGCGCGGGATGACATTGTGCGTGTTTGTTTAGCACTGTCAAACGGTCAGCTTTTTAATCCGCTTTTGTGAGAATCGATTTGATAAAAATAATATAAAACTAACTTGAACATATAATTGACAGAGTTGCGGTAATAGTAAAAATCCGTCACTTTCTAGATCTTAAACCATATAGACCAATTTATTTTATTCTACACGTAATCTTCATAATCTTCATGACATTTAGATTATCTCCTCAGTCAAATTCTTATTTAGAAAAAAAAAATTCAACTAAACAGGTCTACTTATTGAGACATTGGCAACGGCTTTCTCCGGTCTTCAGTCTTCAGTCTGACGTTGGACGCAAAATATTCCTCGTTTGTCTCTTTCCTTAGACATTAGTGCGAAAAACTCCGAATTTTATATATTTTCTAAAACGCGTGAAGCTAGCGCGTGATTGTTGTGCATTGTTCTGCTTCTTCTTCCCCCCACACACACAAAAAAAAAAAACTATATAACAGTATGCTAATTGGCTAATTGCTGTGGAGGGTGGGGTCCATCCAGGTCAACGTCCGCTGCAGTAGTAGTTGCAGACTTGCAGCAGCCGTGCTTTCAGGTCTGGAATCCACGCGCTCCTGCTCCTTCTCTGTCCTTGCATTGCAAATCACAAGTGCGCGACCTCCTTTTTCCTTGCAATTTTACAAGCCAAGCCATCTTCTGCGGTTGCACTGTTAACAGACAGCTACTTCGATTCCATTATCATGCCAAACGCACTGGAAATTACGAAAACGGTGAACAGAAATAATCGATAGAAATGGGTACTAGTTGTTAGTGTGTTTCCTTCTTTTGGTAAGACTTAATAGACACTGCAAACTTGTTTTTTTTTTGTTGCAAACTACAAGCGCACATGCCACATGGCGAGACAGTGACACATGTCATGGGAGATCATTGTGAAGGCTGAGGTGGATAAGTTTAAGAAAGCACAACAAAACTAAGGACACGAGCAAAACTAGTGACACGAGAGATCATTGTGAAGGCTGAGATGGATTATTTGAGATTATTAGGGCTAATTTGGGAATCTCATTTTCTCAAGAGATTTTTATTTTTTCAAGTAAATTAATTTATTTTTTATTGAGAAAATAAAAATCACTTTAAAAAATGGAGTTCCTAAACTAGTCCTTAAGTTTATTTTTCTTCTTCTAGCGTTAAAACTAGATACGGCGCGAGAAAGCGATACTAGCTATGACTTGGTAAGCAACGATACCCGGGGAAAAATCTTGACGATGACCATCGCAGGAGGACCCGGAACGTAAAAAAAAGACGAGAGATGAGAGGAACCAGAGGAGGAAAGAAAAAAGATAGGATGAGAAGCGAACAAAGCAATGGTGGGCGCACCGCCGCGATGAGCAGTGGCCAATTAAGGGCGGGAGGCGATAGAAAATGACGCCACCAAGGCGCCAACGCAGCAAAACGCCCGCCATTACCAAGCCGTCACACGTACGGTCACCTGTCGCAAACCTTGCTTCTAGTTCTAGGCCTGTAGCTGCGTGTTAGGAGTGATTAATTAAACCTAACTGCTCCTCATTAGCATATCAATGTCACCACCCCGCGCTATATTAATACTTGCAGTGCTTTAATCTCTCCCTCTCTCTCTCTCTCTCTGTCTGAGTTCACACTCACGCCGTCACGCGCTCGCGCTCGGCTCGTTCTCCTCTCTTTCCGGCTAAATTGTAAACGTCCAACACACGGCACCACCCAGCAGCGACGACCGCACCAAACAGATA

>ZmMCTP14

CTACTACATCTCTCACCAAGCTCACTTGTTACCCTTGCACAATGCGTAGTAGTAGATAAGTATCACAGAGATTCTTGAATTATATTTTTGGATGAAGGTAATATTATTTAAAGAAGAGCTTTACATGTAATTTTTTTGAATAATGTTTATACTTAAATTCCCTTTTTGCATACGTGACATTTATTCTTCGTAATATTTTTCCATGGAACAGACTTGTGGGTCATTTTCATAAACCAACGCCAAACATGAATCCATGTTTCTTACTTGAAACCCCGAACAAACACCATGAGCAGGAGGCACTCGCGTTAGCGTCGCTCATCGATGACGAGAAGAAGCTTGGTGACTATCAGTTCGACTTCTAGAAGCAGTCCTACGTGGTCGCATGCGTCGCTGTGTACGACAAGCTCCCGCGAAGCCGCCGCTTGGACGCGGTCCAGAGCAGCTGTACCGCGCTGTCCATCGTTAAGCAGGGGGCCTCATGGTTGTCGTCAATGTCGACGACTCTAAGGTTGTTCTGGACACCGCATCCGACGACGACGTCATCACGTCGTCCAGCTCATCGTCCACCTGAAGCCCAACCTGTCACGTAAGTCGCTACTGACCGGACATGAGTTCTTGTGCGCTGGGACGATGAACGTTGCTGACGTTGTGTGAGTGTCCTCGAACATGATGTATGTAGTGGAGTAGCGCATCCGGTAGTGCAACGGCTAGGGGTACTACCTCGCTGATGAGCCCAGGGTGCACTTCGTTTGACAGCCCAACCAAGAGTCATCGGTACTCGTCATGTCGTGCGCGTTCGACGACTACTATATCGAGGATTGTGGCGTCATCATGGCGCCAGAGGAGGATCGACAACAGTGACCAGTTCGTCATCCTCGCCACCGTTAGGGTAGCTTTTGTGCCGGCATGCATTTAATTCTCTTCGCAAACACACACACTTACCTTGTTGTTTAAGCAAGAGCGTATAGCGGTGCATGTCTGATGACTAACGATGTACGAGGGAATTTCTTCCGGCATGTGTGGAACGTGCTCTCTAATGATGAGACCATATAAATCGTGACATATATCGAAGTCTGCATGATATTGATAATTGCAATATAGTGGTGAAACAACTAGATTAAAATAACAAAATTTATGTATAGTTAGGATCATAAATTGATTATGAAATATTTTTTTCATAACAGTATAATATACATTTTGTATATAAATTATCGTAGTATACTAATATTATATATTTCGGTTGTAGGTCACTCAGCTCGGACAGGGAGAGGCGGAGAGCGCATGCGTCCTTGTTCCCCATTACCTAGCTAAAGAAAAAAAGCGAAAGCGTGAAGGCGCAACACGCTGCGGGCCTACGATGTAAAGATCCCCACTCCCAGCTCCAGATCGACGGATGACAACAACACAAAGGATCCGGCATAGCAAGCTGGCCAGCAACAACATTGTGCCCATGCGAACCGAGAGCGCCCGAGCCCCTCCCGCCCAGGGGTCCCACCTCCCGGAGTCCGTCCACATCCGCCTCCCATTCCCACACACAAGGCACGCCTGCCCCCAACACCAAGATTTTTCATACCACCACTGTCTGCAGCAGGCATCACTGACATCTTTGCCCCGCGGTCTGCGACCCCGCATGTCGGTCGAGGGTGTCGTTATCAAAATAGTGGCTGCAACCGCCGGTTAGTGATACAGTTCCTCTCAGAAAGATTGGGCCTGATGCGAGACGTTGGATCGGCATCCAACGACTGCGTTCCGCTACGGTTCGCCGCCCACGGCGACATGCGGTGCAGATCTCGGCTTGGCCCGTACGCCGCCCCCACACTCCTCCACGCAAACACGTCACGCAGGCAGTGGTGGTAGCCCCGCTCCGCTCACAGCACTGTTCGGTGGAATGCCTCACTAGTCACTCACTACTCACTAGTAGGGCTCCTGCTGCCTCCAGGTCCAGCCCACGCTACCCCACACCGCCGTTCCTCCACTGAGCAAACCCCGCCGCCGACGAG

>ZmMCTP15

AATGTTATTTTTACCTATTACATGTCTATATATATGTATTGCTGCACATAGAACCGAGGTGACGAGGAATCTGAATCCCAATTATTGGGAAAGCACCTGAGTACCTGAGATCACAAGTAAAAGATAAGTTGTGTTCTTGATCAGTTTCTTTGACCTAATAATGTTCATGATTCTTAATCATTGTGGCACTGTTAGGTTGAGCTGACGGGGTCTAATAGGTTACCTTGTTTACCCTACACCTTGTATACAAATAAATTGTTGGGTAGTCTTGGTATTGCTCTACCTGGTTTTGAGATTAATACTATATATGAATCATGATCCTGTTTATCATTATTGTTAATAGATTATTTATTGTTCATGATAAGATCACTTTGTTAATTGAAACATGGAGCGACCACCTGAAAAATATATTGCTACCACAAGGGTGACCACCTTATTATAGCCCTCTAGGACGTCATCCAGCCCGTCATCGGACAGAGACAGGGAGGACCAACGACATCTTGAAAACTGCAGCATGACCAAAGCCTGTCGCCGAGACATGGAAGGGGAGTCGGTCCACCTTCCACTTATAACCTACACAAAGGCAACGTAAATAGGTCTTACCTCAGGGAATCTGGATCGGTGGTCGTTGAAACCAAGATTGGGGGAGGAGAAGTAGATCTCGCCCCCGGCCACGACTTCTAGAGGGGGTAAGACGACAACGACGACGACGAACTACGATGGCGGCGATGGTTGTCGCGGTTGTAGGTCACAATCCACCACTTCTGCCCCCCACCGAAACACCACAGCCGCCTTCTCTTGTTGCTCCTCCGCGCCGGCGTCGTCCACCGGATCCGACTCCGCCTTCTTTCACCAACCGTGCTGTCGCCTTGCCTTCGGTCTCCTGAAGTCGTTGCTCGACTTCAACGCCAATGGAAGGGAGCAGGATGTTTCTCCGCGTGCGCGCGGGCGTTTCCGTTCGCGAGAAGCCACCGACTAGGGATGAATAGGGCTCCTCCTCGCCTGGTGTGAGAGAGAAGGGAACGTCACTAAGGGGGCGAGGAAGAGGAGGCGATGCAGGAGTAAGGTAATATTGTATCCCCTATAGAGGCTAGGGACATGAGGTGCGGACAGTCTTAGACCATCTGCACTACAGTGCGTGAAAACTCACGGTAAACTGATACAATGAATGGTAGCGTAAAAAAACTTGCACCGCTGCACGCTACATTTTCCTGTATAGCTATGTAGCTAGAGGTGACAATAGACTCTAAATTTTACACTATAAGATTTAAGGATCGGATCGAATTAGAATCGAACTCTATTTCTATTCATTTTTGAACTAAAATTTATTTAGGGTCATACCATTTTGTGAAGAAGCATTTGAATCGTGATCCATTTTCACCCCTATCTAGCTAAAGTAAAGCACTATGTACAGCGCACACAAAATCTTCATGTATCCATATCGGCTGCAGCAAAAGCCCTGTGTTATATAGAGAGAAAAAATAGTTAGGTTAGAATGTGTATTAGTTGATATAAAGGGAATATGGAGGGATATATGTACGAATGATTGTAATATAGAGTAAGAAATTTTGATGACTAAATTATGAATTGTGATATAGAGCAAGAATATAGAGTAATGTACACTGCGCTGCTGGTTCTGGGTCTGGGCGCAGCCGCGCACAGAACAGAGCATGTATACATGGAATGCTCTGTTAACTGTCGGCCTGTGTTTAGGAACGCGATATAAAACAGAAAAGGGCAGGGGGAGCTGGGAAGAAGTTGCCAACTAGCGTATCGGCGGCGCCACCACCCAACCGCTCAACCACATCAGTGCACATTCAAAAGCGCCAAGAACAGATCAGATTCTGCCCGCACAAAGCTGAGAGCTTCCACGTCAAGAAAGACACCAACTTCTCCTCTCTTCCTCTGCGCCTCTCTCACTCTAGCACAGATTCTCACTTCTCCTCGTCCCTCTCCAAAGCACACTAGCTCTCTGCTCTCCCCAAAAGCAAAAGGAGCCA

>ZmMCTP16

TTTTTAAAACTAAAGCTCGTGGAGCTGGACCTGTTTGGGTTGAAGAAGTTCGAACGAAATTGGAATTCTTGAAGTGAAGCTCTCCCAAACAGGGCCTAAATTAATATAAAATATATTTTAGTTTTGTCATGTCATATAATAAATTGTAGTATTATAGGCTATGGTTTTAAGAAACGGAGTTTCTGAACAGGCCCAAGTTGAAAAAAAGTATCACGTCTAATGCGTGTAACAATTAGATCTAGAATTTCATACCTAAACTTTCCTATTGTACAATTTTTTTTTGAATAAATGTACATCTACTTAGAAAGTAGTAAACGAGTCTAACCATCCAACCTAATTAAATGAGCGTTTACAGCATCCGTAGTTCAGGGTTTGTACCATACTAACAATGTTCCTTTTATTTTCCGCAGTTTTTTTTTTGCCAATGATTTTGCTTTTCTTTTCTGAGAATGTGAATGGTTTCACTCCGTTGCAGACCTGACAGTAGAAAAAATTACCCTGTTTCTTCTCACGGGCCTGGTCGTGCCGCCCTTCTCGGGCCTCGCCGGTTTTTAAAAGCAGGCCAGAGCTAAACCCTGCGCCAGGCCCACGCCGAGTTGGCCGGGGGCGTCTGGGCTCGGTCCAAGCGCGGCAAGACGGAAACAACACGTGCCCGAGCCCGACCGCGGTCCATGGCTCGGCAAATCCGCGCACGCCCGTCCTCGACGCGAGCGCACGCCGGTTTCGGATTTCAAAGCGAGGGAGAGAGAGAGAAGTGGAACGGGCAGACCGACGCGAATATACACGGCGGGCCCCCGAGCTGTCGCGTGGGGGCGGGGGCGCGGGGCACGGTACCATCGAGTGAGTGCTGTCTCGACCTCCCGTGGTCGTGCAGGGCGGGCGCGCGGTTATGGATATTTTGCGCCAAGGCCCTCCATGCTTACGCCTAATTCGTTTATGTGCGGTGCGTGACTCCCGGGCCGTTTGCTGCTACGGCGAGTATCTGTTGGTAATACCATCAATAAAAATTATACTATTTGGGTTATCCATATACGCTTGCACGTATTAAATCTGTTCTATATCTAAACTCGTGCGAGTAATTTTAATAACCTATACCCACTAAAAAGATCTTATATTCTAATATTTCTATTTCTTAAAATGTCAAATAGACACACAAATATAAGCTAATCTTAAAATATAGCAAACATGTGATTATATAACTTAAGGGCTAGTTTGGGAATCCAAAAACCGGAGGGGATTGAAGGGACTAAAATCTCCTTTTTATTTAAAATTGAATAAAGAGGAGATTTTAGCCCCTTCAATCCCCTCCGGTTTTGAGGTTCCCAAACTGGCAGTAATAGTTAAACAAATAATATCCGGAGAAGGATTTTCTTTTAGCTAAGATGTGCTCTATTATTGATAATAATAGTATGATGTGAGTATATTTTACCCACTGGTACACCACCAATTCTCATACATGTCTACCCAACGGGTAGAGATTTTTACCCACTAACATACATGCAAGTAGAGAAACCATCATATTACTAGCTACAAATCAGAGATTTTCGGGTTTCGGGCCCATTGCCATATTTAACGACGAAGCAGTTGGTCGAACTCGTTGTAAATTGACACTAACAAGAGAAGCAAGTAGATAGCGTGCTCGGAAGTTGATTGTAAGTAGTACATTAAACTATTCAATATGGGTAGTACAAAGAATGTGCATTATAATTAATGACTCTTATCTCTTGTTACATGAGATTATGTCCAAAACGCCAAGAATTTTAGAAGAATTTTAGAACGGGAGGGAGTATACCATGCAACAAATGGCGACGAAGCCCACCCCACGTCAAAAGCTACAGAAAACGACAATGCAGATTGAGCTGGCACGGAACCCTAGGCAGACGACCGGTCCTTCCACCAAAATCCTACCAAGGCCGAGGGCCTCCGCTGAAAAGCGCCGACCGGTGAGGGGCTTTTGCGCTCCCAAAACCAAAACCCCGCCCTCACTCCACTTCGTCCCC

>ZmMCTP17

TATGGTGCATGCTCGTAGGAATGTAGCACGGCTATCGCATGAGCTAAACCAACAGCAGACATCATTTAGCACAACCGAATAACATGACGTATCTGTGAATAGTGCTGAGAATTAGGTTAGGTTGGGCTGACCCGGCCTATCGGCTTAAACGGGATCAGACCAAGAAGGTTCAAAAGACTTTTGGGTTGTGCCGTGCCAATCTAAAAATAAAAATAGTTAGGTCATCCTGATTTAAAACTAAACTAAATATATCAAACAATGATAGCATTATTTAACCATATAAAATCTAAATATTCAACAATATAAAGTACATAATTTAATAAATTAAAAGAAATTAAACAATATAAACTTTATTTGGCCATCTCAGTCTAAGACCGACCCATGTATGTGGGCCGTGTCAGGACCCAACTAATCAAAATTTGAGGCTCGACCCATATTATCCGTGTCGGATTGTGTTTTAGTCTTCTGTTTGTGTACATGCTCGTGTTTAATGTTTATGGCAAAAAGGCGAACATATTCTCAACACTATATGTGATGCAATGGCGCGCTGGTTCGACCGCTCGATCTTTTCTCTTCTGTGGGTTGGTCCGTCGGTGACCTCGCCTGTGTTGGTTTAGGGTTTCGATTATCTAAAAAAAAAGGAAACACTAGAATTTTAAATCATCTATGCATTTAATTTCTACCGTTACAGTGTATACTATATTGCAATATATTTGGATCATGTATTTTACGTTATTCATGCATCTTATATCAAATTTAGATGGTTTTGAGTTCCTTCGGGCAATGGACTCAGATTCGGCAGGTGACCATATTAATTAAAGTTAAACATCACTTGCTTTATATTTTTTTTACCGTCAACAACGCAGAGGAAGAGTATCCCCACATAAAATCCAAAATCCATTGAATTCGGCATGACTGATATTGAGTATCGTACTTAGGGGTACCTATATTACGCCTCTAATACATGTTTAACCTCTAGACCATCATCAAGGCACATTCTCCGAGGATGAAGGATCATAAGCCGCGCTTCACCCGAGGCCCCTCTTAGGGGTCACCATAACTCCGGCTCGCACGAGCCTACCCTCGGACAGGGCGTGTTCTCGGGAAAATCATCGTCCTAGCTAGGCCCCTTCTCCCGAGAACGACAGTCTTCGCCTCGCTCGATCCCGTCTCAGGTAAGGAAGACAAACCCAGGACAAGACTCAGCCGAAGTCCGCAGGGGAGAGAAGCATTCAATGCACATACCTACCCCACACAGGATTGCAGGTGAACTGGAGTAACAAGACTGCGGTCCTGTCAAGCTTCAACAACAATGATGACGCGTGCGACCACTATTCCCGCACACCGTATGCCAACCCCCCGATGGGACGTACAATACGACAAGAGTGCAGGCTGGCCCTCGGGCGCAAACTCTGCCTCGCCTGAAGTAGACCCTGATTCGGGGCAAACTCTGCCTCGCCCGAGCCCAGCCTCGGTTACCTACTCCCCGCGAATCCCACAGGAGGCAATACTTCAACTGTGGCGCGCATGTCTTTGAATCCAGGAACGAGCTCGGTGGTGACCTCGGGCGGACTTCCGCCTCGCTCGAGCATGGCTCTGACCTCGATATCCGCAACGGAAAGGCGCCCAACGTCACCATATACTACAGAGCTGACGTATTACTTAGGAGCTTTTCTTATACTTAGTACTGTGTCAACCACTATGGTATGGCAAACCCCTTGCCAGAGAGGCTCCGACGACGATCTCACTTTGTGCAACCAGAAACACCCGACGACGCATCTGGGCTCCGCTTGCCATCATCCAACGCTATGCTAGAGGAATCCTCGCGGGTGATTTTCGTGCTCCTAGTAATAAGGAGAGCGTCGACGGTGCAGAGGCGCCAAAAGGAGGCACTTGTTGCATGACAACAGCTCTCGAAACGCCAGTGCTAAGCTCAACGACACAAGGGACCTGCAAGGACATGAAAATCATTGACGCGACATGAGCTCCCCAAGACGCAA
