# Supplementary material for: Characterization and phylogenetic analysis of multiple C2 domain and transmembrane region proteins in maize
Source: BMC Plant Biol. 2022 Aug 3;22:388. doi: 10.1186/s12870-022-03771-x (PMC9347167; doi:10.1186/s12870-022-03771-x)
Supplement: Supplementary file 3 — Additional file 3: Figure S1. Correlation analysis between MCTP numbers with genome size (A) and Gene Loci No. (B), respectively. Figure S2. The maximum-likelihood (ML) phylogenetic tree was built by MCTPs from 32 species. Figure S3. Conserved motif compositions of MCTPs in five clades. Table S1. Genome information of 33 plant species and five outgroup species. Table S2. The homolog pairs of MCTP genes between maize and four other plant species. Table S3. Ka, Ks and Ka/Ks ratio of WGD/SD and LD duplicated gene pairs in surveyed angiosperms. Table S4. Primer sequences used for RT-qPCR analysis. [file 12870_2022_3771_MOESM3_ESM.pdf]

Figure S1. Correlation analysis between *MCTP* numbers with genome size (A) and Gene Loci No. (B), respectively.

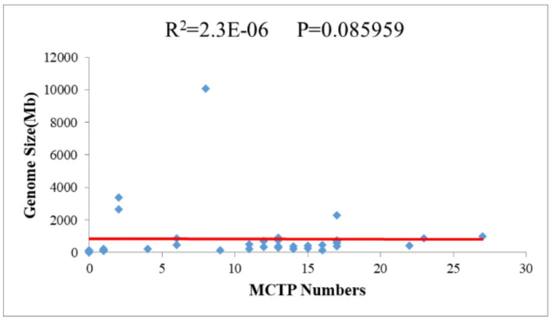

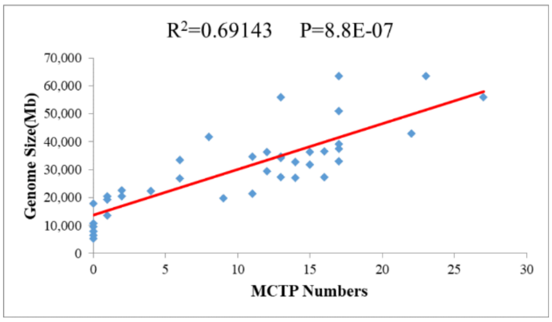

Figure S2. The maximum-likelihood (ML) phylogenetic tree was built by MCTPs from 32 species.

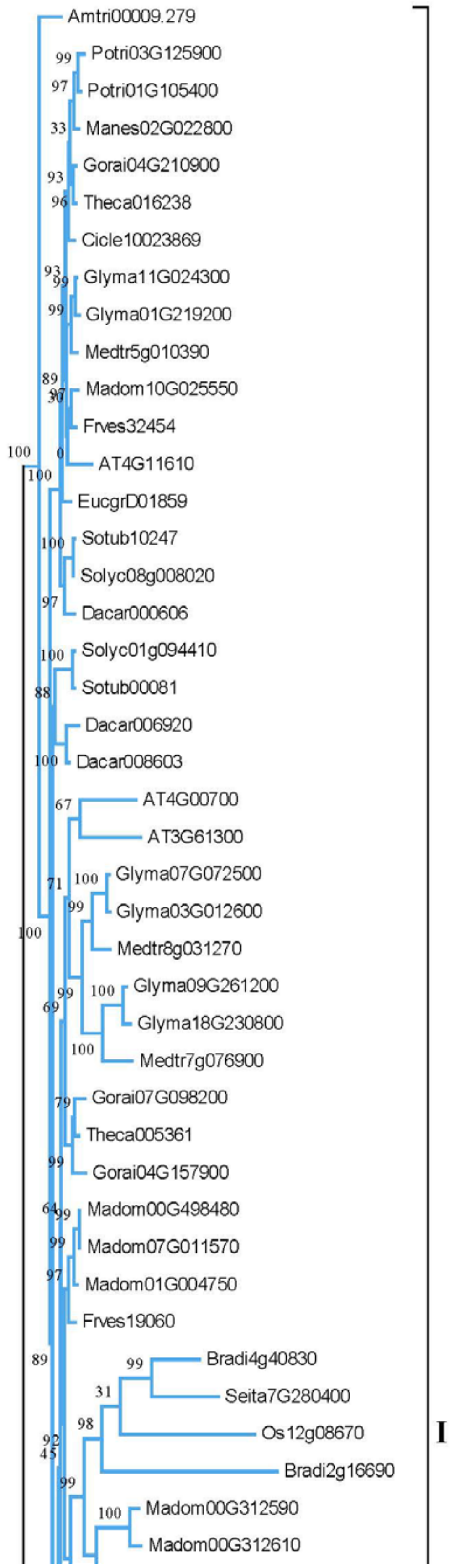

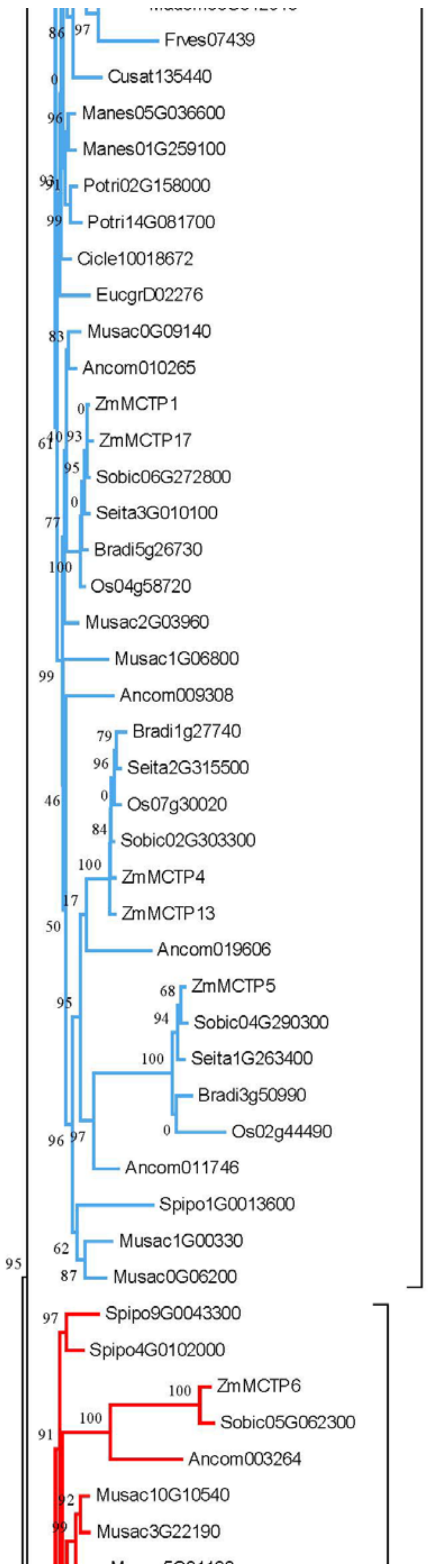

Frves07439
Cusat135440
Manes05G036600
Manes01G259100
Potri02G158000
Potri14G081700
Cicle10018672
EucgrD02276
Musac0G09140
Ancom010265
ZmMCTP1
ZmMCTP17
Sobic06G272800
Seita3G010100
Bradi5g26730
Os04g58720
Musac2G03960
Musac1G06800
Ancom009308
Bradi1g27740
Seita2G315500
Os07g30020
Sobic02G303300
ZmMCTP4
ZmMCTP13
Ancom019606
ZmMCTP5
Sobic04G290300
Seita1G263400
Bradi3g50990
Os02g44490
Ancom011746
Spipo1G0013600
Musac1G00330
Musac0G06200
Spipo9G0043300
Spipo4G0102000
ZmMCTP6
Sobic05G062300
Ancom003264
Musac10G10540
Musac3G22190

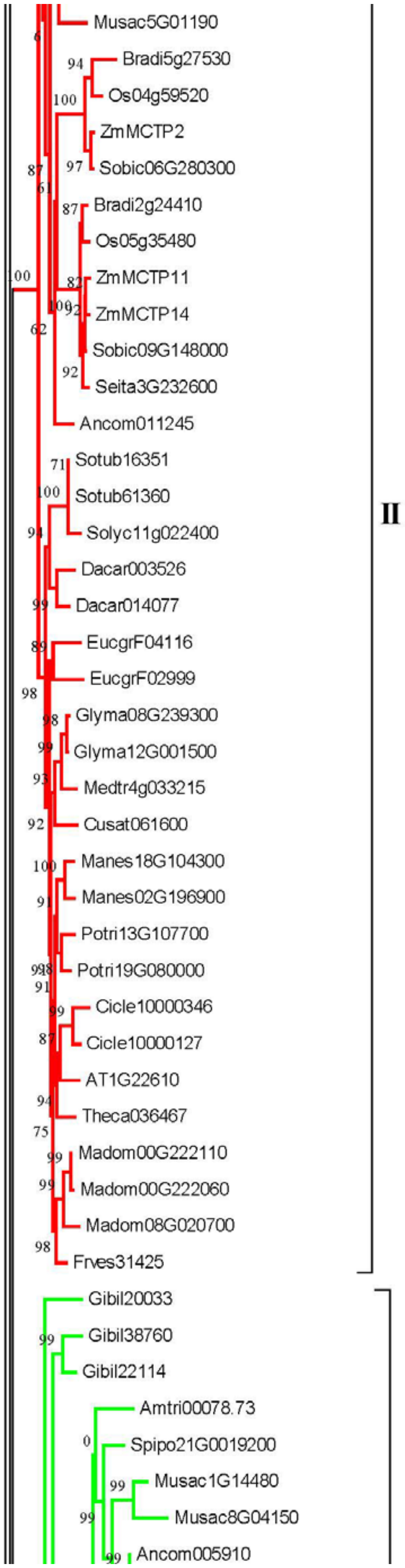

Musac5G01190
Bradi5g27530
Os04g59520
ZmMCTP2
Sobic06G280300
Bradi2g24410
Os05g35480
ZmMCTP11
ZmMCTP14
Sobic09G148000
Seita3G232600
Ancom011245
Sotub16351
Sotub61360
Solyc11g022400
Dacar003526
Dacar014077
EucgrF04116
EucgrF02999
Glyma08G239300
Glyma12G001500
Medtr4g033215
Cusat061600
Manes18G104300
Manes02G196900
Potri13G107700
Potri19G080000
Cicle10000346
Cicle10000127
AT1G22610
Theca036467
Madom00G222110
Madom00G222060
Madom08G020700
Frves31425
II
Gibil20033
Gibil38760
Gibil22114
Amtri00078.73
Spipo21G0019200
Musac1G14480
Musac8G04150
Ancom005910

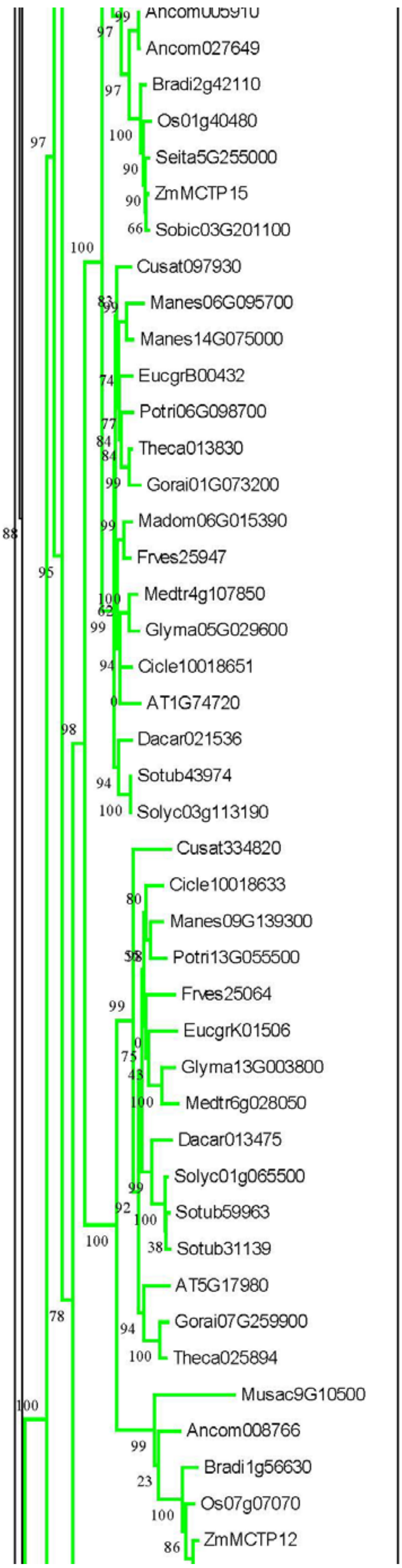
Ancom027649
Bradi2g42110
Os01g40480
Seita5G255000
ZmMCTP15
Sobic03G201100
Cusat097930
Manes06G095700
Manes14G075000
EucgrB00432
Potri06G098700
Theca013830
Gorai01G073200
Madom06G015390
Frves25947
Medtr4g107850
Glyma05G029600
Cicle10018651
AT1G74720
Dacar021536
Sotub43974
Solyc03g113190
Cusat334820
Cicle10018633
Manes09G139300
Potri13G055500
Frves25064
EucgrK01506
Glyma13G003800
Medtr6g028050
Dacar013475
Solyc01g065500
Sotub59963
Sotub31139
AT5G17980
Gorai07G259900
Theca025894
Musac9G10500
Ancom008766
Bradi1g56630
Os07g07070
ZmMCTP12

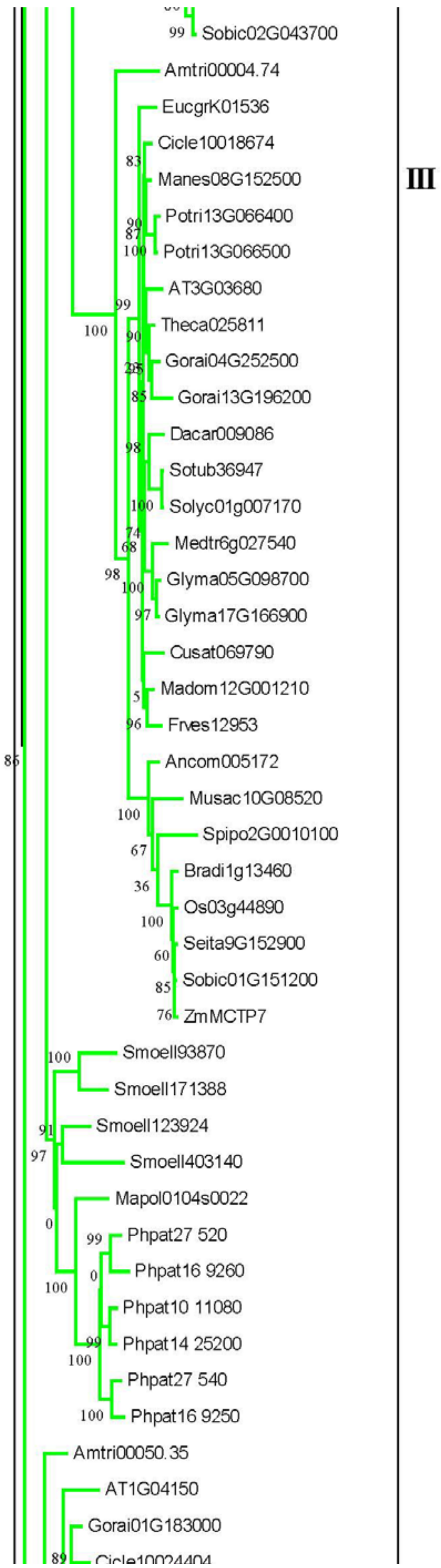
Sobic02G043700
Amtri00004.74
EucgrK01536
Cicle10018674
Manes08G152500
Potri13G066400
Potri13G066500
AT3G03680
Theca025811
Gorai04G252500
Gorai13G196200
Dacar009086
Sotub36947
Solyc01g007170
Medtr6g027540
Glyma05G098700
Glyma17G166900
Cusat069790
Madom12G001210
Frves12953
Ancom005172
Musac10G08520
Spipo2G0010100
Bradi1g13460
Os03g44890
Seita9G152900
Sobic01G151200
ZmMCTP7
Smoell93870
Smoell171388
Smoell123924
Smoell403140
Mapol0104s0022
Phpat27 520
Phpat16 9260
Phpat10 11080
Phpat14 25200
Phpat27 540
Phpat16 9250
Amtri00050.35
AT1G04150
Gorai01G183000
III

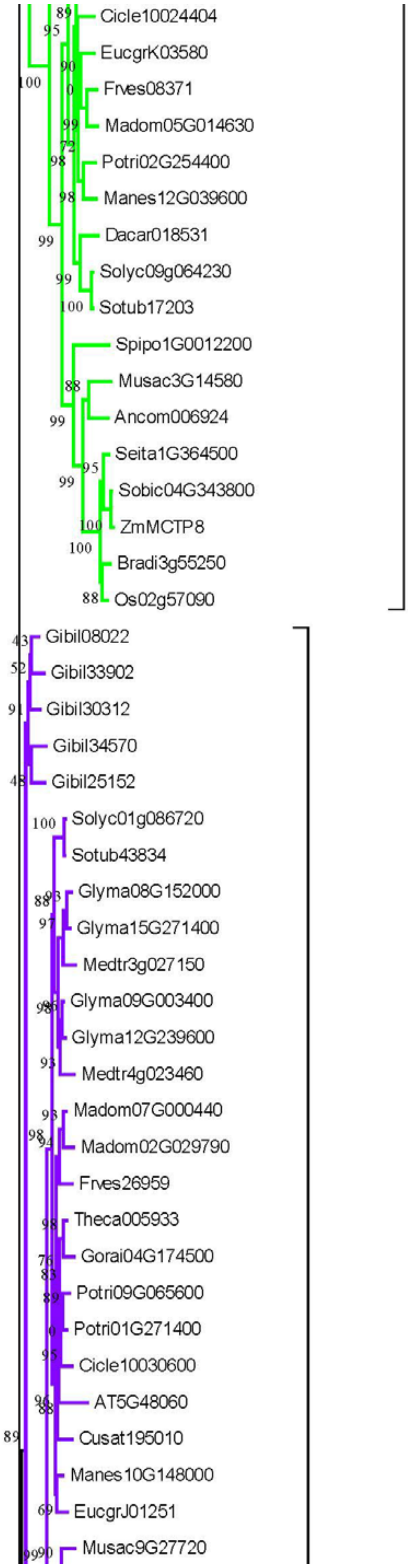
Cicle10024404
EucgrK03580
Frves08371
Madom05G014630
Potri02G254400
Manes12G039600
Dacar018531
Solyc09g064230
Sotub17203
Spipo1G0012200
Musac3G14580
Ancom006924
Seita1G364500
Sobic04G343800
ZmMCTP8
Bradi3g55250
Os02g57090
Gibil08022
Gibil33902
Gibil30312
Gibil34570
Gibil25152
Solyc01g086720
Sotub43834
Glyma08G152000
Glyma15G271400
Medtr3g027150
Glyma09G003400
Glyma12G239600
Medtr4g023460
Madom07G000440
Madom02G029790
Frves26959
Theca005933
Gorai04G174500
Potri09G065600
Potri01G271400
Cicle10030600
AT5G48060
Cusat195010
Manes10G148000
EucgrJ01251
Musac9G27720

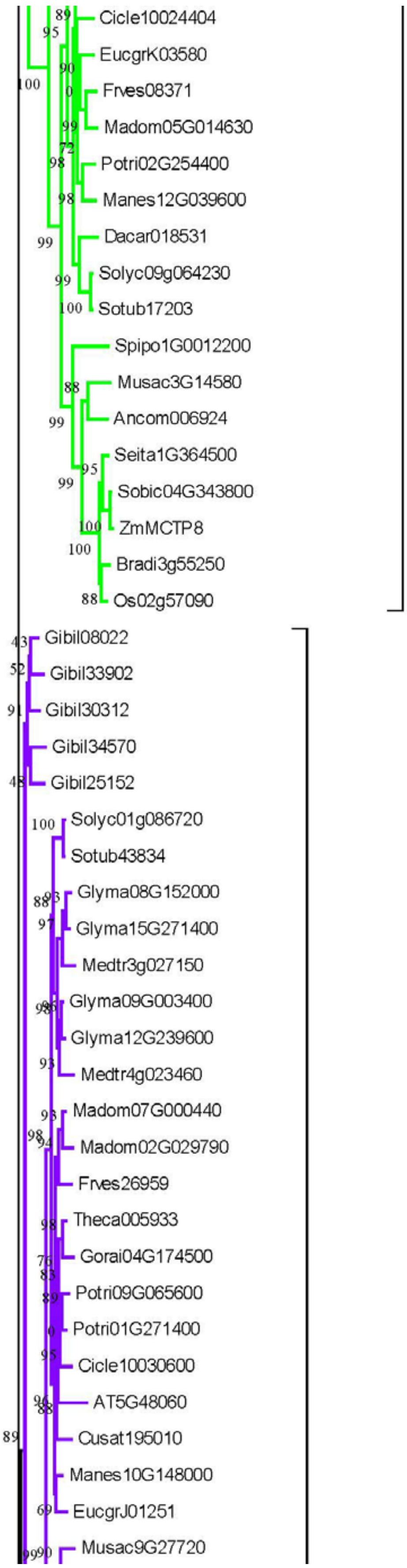
Cicle10024404
EucgrK03580
Frves08371
Madom05G014630
Potri02G254400
Manes12G039600
Dacar018531
Solyc09g064230
Sotub17203
Spipo1G0012200
Musac3G14580
Ancom006924
Seita1G364500
Sobic04G343800
ZmMCTP8
Bradi3g55250
Os02g57090
Gibil08022
Gibil33902
Gibil30312
Gibil34570
Gibil25152
Solyc01g086720
Sotub43834
Glyma08G152000
Glyma15G271400
Medtr3g027150
Glyma09G003400
Glyma12G239600
Medtr4g023460
Madom07G000440
Madom02G029790
Frves26959
Theca005933
Gorai04G174500
Potri09G065600
Potri01G271400
Cicle10030600
AT5G48060
Cusat195010
Manes10G148000
EucgrJ01251
Musac9G27720

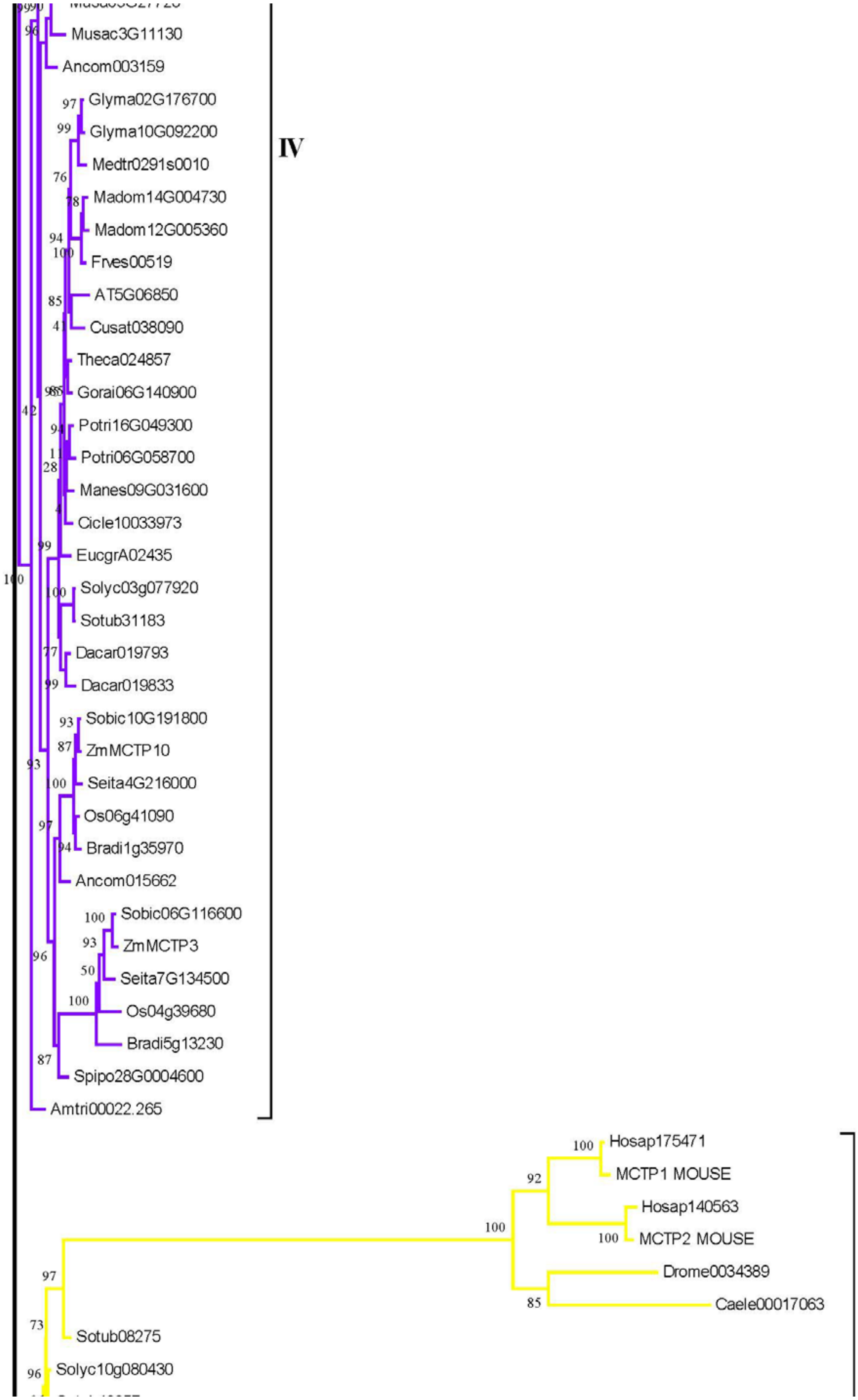

Musac3G11130
Ancom003159
Glyma02G176700
Glyma10G092200
Medtr0291s0010
Madom14G004730
Madom12G005360
Frves00519
AT5G06850
Cusat038090
Theca024857
Gorai06G140900
Potri16G049300
Potri06G058700
Manes09G031600
Cicle10033973
EucgrA02435
Solyc03g077920
Sotub31183
Dacar019793
Dacar019833
Sobic10G191800
ZmMCTP10
Seita4G216000
Os06g41090
Bradi1g35970
Ancom015662
Sobic06G116600
ZmMCTP3
Seita7G134500
Os04g39680
Bradi5g13230
Spipo28G0004600
Amtri00022.265
IV
Hosap175471
MCTP1 MOUSE
Hosap140563
MCTP2 MOUSE
Drome0034389
Caele00017063
Sotub08275
Solyc10g080430

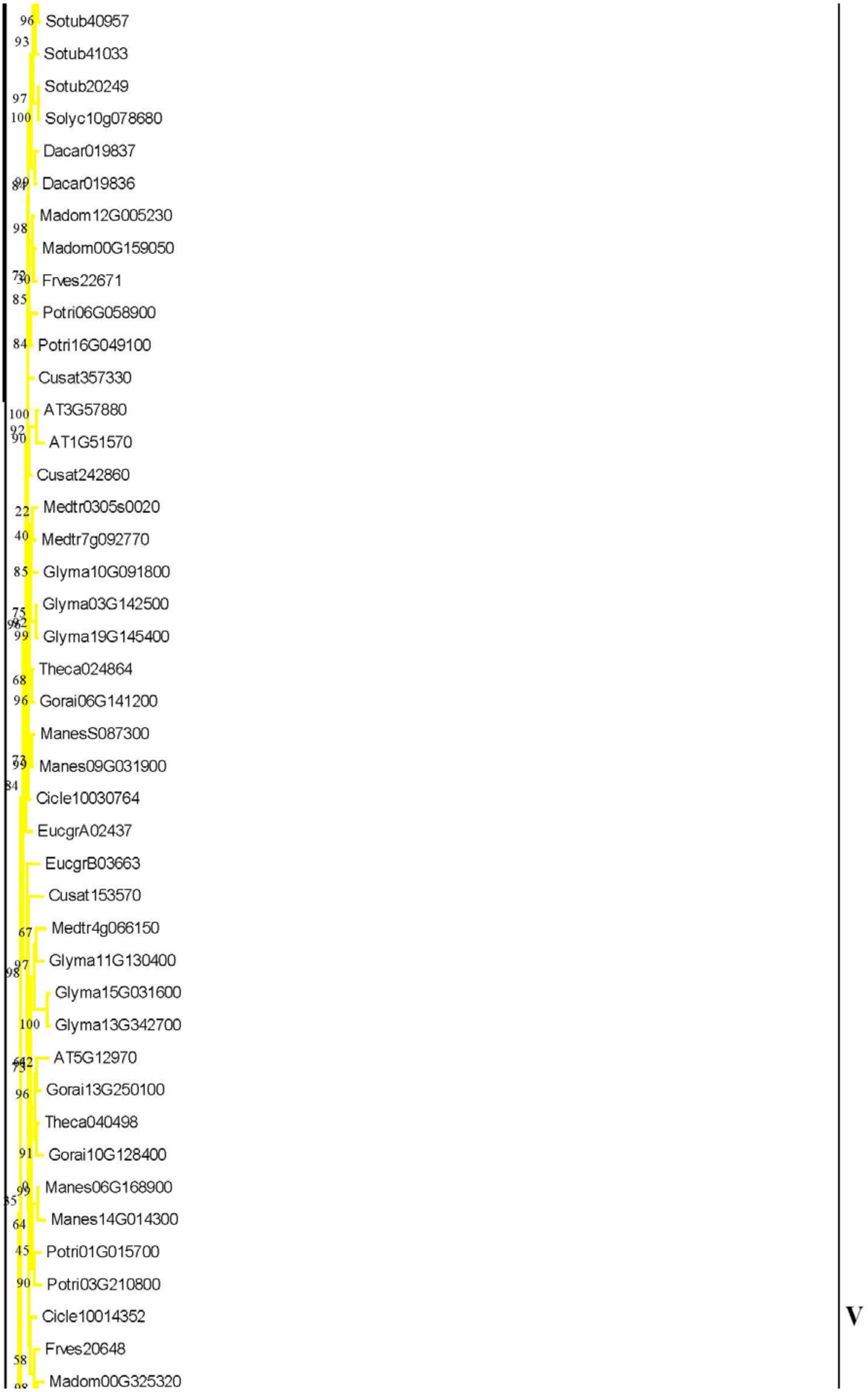
Sotub40957
Sotub41033
Sotub20249
Solyc10g078680
Dacar019837
Dacar019836
Madom12G005230
Madom00G159050
Frves22671
Potri06G058900
Potri16G049100
Cusat357330
AT3G57880
AT1G51570
Cusat242860
Medtr0305s0020
Medtr7g092770
Glyma10G091800
Glyma03G142500
Glyma19G145400
Theca024864
Gorai06G141200
ManesS087300
Manes09G031900
Cicle10030764
EucgrA02437
EucgrB03663
Cusat153570
Medtr4g066150
Glyma11G130400
Glyma15G031600
Glyma13G342700
AT5G12970
Gorai13G250100
Theca040498
Gorai10G128400
Manes06G168900
Manes14G014300
Potri01G015700
Potri03G210800
Cicle10014352
Frves20648
Madom00G325320
V

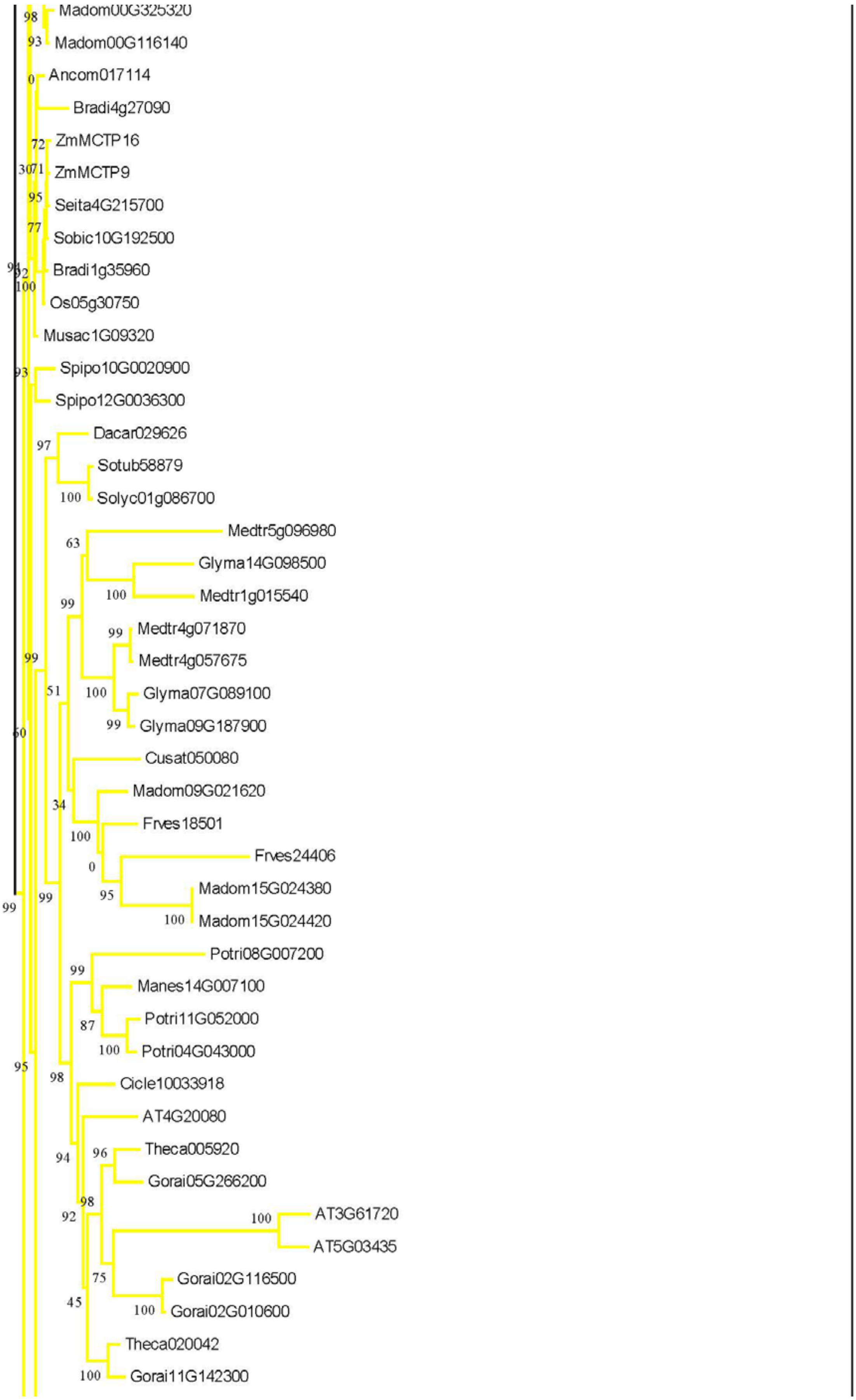
Madom00G325320
Madom00G116140
Ancom017114
Bradi4g27090
ZmMCTP16
ZmMCTP9
Seita4G215700
Sobic10G192500
Bradi1g35960
Os05g30750
Musac1G09320
Spipo10G0020900
Spipo12G0036300
Dacar029626
Sotub58879
Solyc01g086700
Medtr5g096980
Glyma14G098500
Medtr1g015540
Medtr4g071870
Medtr4g057675
Glyma07G089100
Glyma09G187900
Cusat050080
Madom09G021620
Frves18501
Frves24406
Madom15G024380
Madom15G024420
Potri08G007200
Manes14G007100
Potri11G052000
Potri04G043000
Cicle10033918
AT4G20080
Theca005920
Gorai05G266200
AT3G61720
AT5G03435
Gorai02G116500
Gorai02G010600
Theca020042
Gorai11G142300

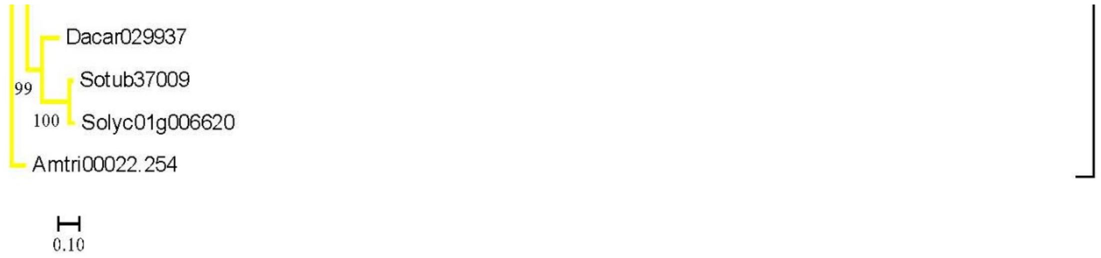
Dacar029937
Sotub37009
Solyc01g006620
Amtri00022.254
99
100
0.10

Figure S3. Conserved motif compositions of MCTPs in five clades.

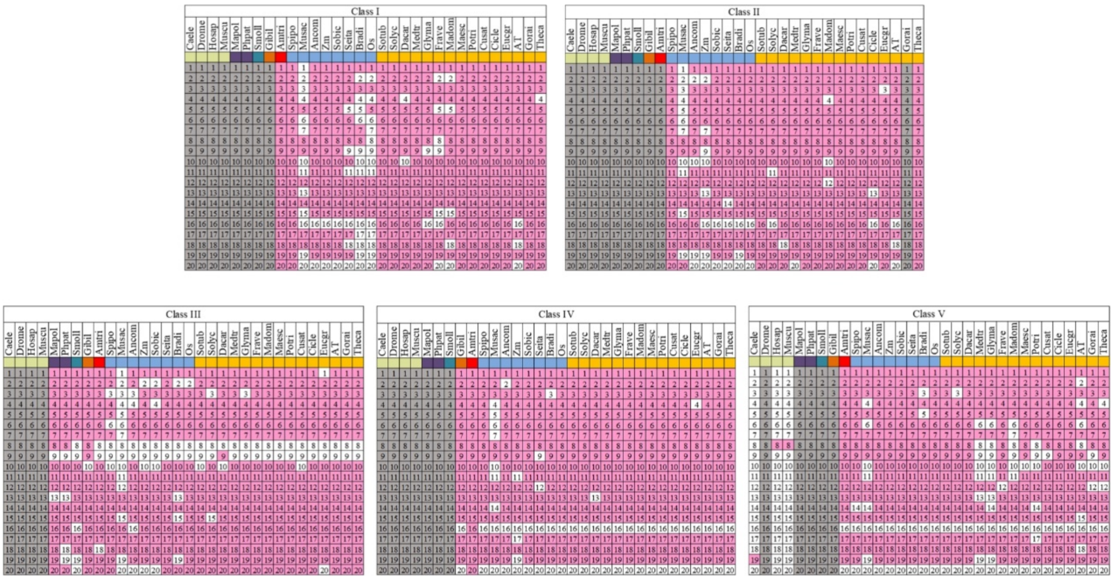

Table S1 Genome information of 33 plant species and five outgroup species.

Taxonomy	species	Abrr.	Genome Size(Mb)	Gene Loci No.	Chr No.	MCTPs homologs No.
Rhodophyta	*Chondrus crispus*	Chcri	104.8	9,603	-	0
Rhodophyta	*Cyanidioschyzon merolae*	Cymer	16	5,331	20	0
Ascomycota	*Saccharomyces cerevisiae*	Sacer	12	6604	16	0
Bilateria	*Caenorhabditis elegans*	Caele	100	20512	6	1
Bilateria	*Drosophila melanogaster*	Drome	120	13600	8	1
Bilateria	*Homo sapiens*	Hosap	3400	20440	46	2
Bilateria	*Mus musculus*	Muscu	2662.4	22619	21	2
Chlamydomonadaceae	*Chlamydomonas reinhardtii*	Chrei	120	17,741	17	0
Bathycoccaceae	*Ostreococcus lucimarinus*	Osluc	13.2	7,796	21	0
Mamiellophyceae	*Micromonas pusilla CCMP1545*	Mipus	22	10,660	19	0
Marchantiaceae	*Marchantia polymorpha*	Mapol	225.8	19,287	10	1
Bryophyta	*Physcomitrella patens*	Phpat	473	33,362	27	6
Selaginellaceae	*Selaginella moellendorffii*	Smoll	212.5	22,273	27	4
Ginkgoaceae	*Ginkgo biloba*	Gibil	10061	41840	24	8
Amborellaceae	*Amborella trichopoda*	Amtri	870	26846	13	6
Araceae	*Spirodela polyrhiza*	Spipo	158	19,623	32	9
Musaceae	*Musa acuminata*	Musac	472	36,542	12	16
Poales	*Ananas comosus*	Ancom	382	27,024	25	14
Poaceae	*Zea mays*	Zm	2300	63,480	10	17
Poaceae	*Sorghum bicolor*	Sobic	732.2	34,129	10	13
Poaceae	*Setaria italica*	Seita	515	34,584	9	11
Poaceae	*Brachypodium distachyon*	Bradi	272	31,694	5	15
Poaceae	*Oryza sativa*	Os	372	55,986	12	13
Solanaceae	*Solanum tuberosum*	Sotub	723	39,028	12	17
Solanaceae	*Solanum lycopersicum*	Solyc	900	34,727	12	13
Solanaceae	*Daucus carota*	Dacar	421.539	36,244	9	15
Fabaceae	*Medicago truncatula*	Medtr	390	50,894	8	17
Fabaceae	*Glycine max*	Glyma	978	56,044	20	27
Rosaceae	*Fragaria vesca*	Frave	240	32,831	7	14
Rosaceae	*Malus domestica*	Madom	881.3	63,514	17	23
Euphorbiaceae	*Manihot esculenta*	Maesc	582.25	33,033	18	17
Salicaceae	*Populus trichocarpa*	Potri	423	42,950	19	22
Rutaceae	*Cucumis sativus*	Cusat	203	21,491	7	11
Rutaceae	*Citrus Clementina*	Cicle	301.365	27,326	9	13
Myrtaceae	*Eucalyptus grandis*	Eucgr	691.43	36,376	11	12
Brassicaceae	*Arabidopsis thaliana*	AT	135	27,416	5	16
Malvaceae	*Gossypium raimondii*	Gorai	761.4	37,505	13	17
Malvaceae	*Theobroma cacao*	Theca	346	29,452	10	12

Table S2. The homolog pairs of *MCTP* genes between maize and four other plant species.

Gene 1	Gene 2
Zm00001d001785	*Seita.3G010100*
Zm00001d003321	*Seita.7G134500*
Zm00001d051389	*Seita.1G263400*
Zm00001d013654	*Seita.9G152900*
Zm00001d018473	*Seita.1G364500*
Zm00001d036801	*Seita.4G215700*
Zm00001d038340	*Seita.3G232600*
Zm00001d018871	*Seita.2G043200*
Zm00001d021494	*Seita.2G315500*
Zm00001d010202	*Seita.3G232600*
Zm00001d011239	*Seita.5G255000*
Zm00001d046749	*Seita.4G215700*
Zm00001d026650	*Seita.3G010100*
Zm00001d001785	*Os04g58720*
Zm00001d002939	*Os04g59520*
Zm00001d003321	*Os04g39680*
Zm00001d021494	*Os07g30020*
Zm00001d051389	*Os02g44490*
Zm00001d013654	*Os03g44890*
Zm00001d018473	*Os02g57090*
Zm00001d036801	*Os06g41090*
Zm00001d038340	*Os05g35480*
Zm00001d018871	*Os07g07070*
Zm00001d010202	*Os05g35480*
Zm00001d011239	*Os01g40480*
Zm00001d026650	*Os04g58720*
Zm00001d026650	*Sobic.006G272800*
Zm00001d002939	*Sobic.006G280300*
Zm00001d003321	*Sobic.006G116600*
Zm00001d021494	*Sobic.002G303300*
Zm00001d051389	*Sobic.004G290300*
Zm00001d053062	*Sobic.005G062300*
Zm00001d013654	*Sobic.001G151200*
Zm00001d018473	*Sobic.004G343800*
Zm00001d036801	*Sobic.010G192500*
Zm00001d010202	*Sobic.009G148000*
Zm00001d018871	*Sobic.002G043700*
Zm00001d006371	*Sobic.002G303300*
Zm00001d038340	*Sobic.009G148000*
Zm00001d011239	*Sobic.003G201100*
Zm00001d046749	*Sobic.010G192500*

Table S3. Ka, Ks and Ka/Ks ratio of WGD/SD and LD duplicated gene pairs in surveyed angiosperms.

Duplication pairs	Gene1	Gene2	Ks	Ka	Ka/Ks
Aco_WGD1	*Aco003264*	*Aco011245*	49.4147	0.4047	0.0082
Aco_WGD2	*Aco010265*	*Aco009308*	1.7074	0.2176	0.1274
Aco_WGD3	*Aco010265*	*Aco011746*	34.6468	0.2247	0.0065
Aco_WGD4	*Aco015662*	*Aco003159*	6.0394	0.1045	0.0173
At_WGD1	*AT4G20080*	*AT5G44760*	1.2352	0.2029	0.1643
Bd_LD1	*Bradi1g35960*	*Bradi1g35970*	59.6025	0.2098	0.0035
Bd_LD2	*Bradi5g26730*	*Bradi5g26690*	2.8457	0.2579	0.0906
Cicle_LD1	*Ciclev10000346*	*Ciclev10000127*	0.0786	0.05	0.6359
Cicle_LD2	*Ciclev10030600*	*Ciclev10033918*	5.2305	0.2949	0.0564
Cicle_LD3	*Ciclev10033973*	*Ciclev10030764*	3.9227	0.1708	0.0435
Cicle_WGD1	*Ciclev10018672*	*Ciclev10023869*	2.1119	0.138	0.0654
Cicle_WGD2	*Ciclev10033918*	*Ciclev10014352*	2.1129	0.249	0.1178
Dacar_LD1	*DCAR_019833*	*DCAR_019836*	6.5743	0.2042	0.0311
Dacar_LD2	*DCAR_019836*	*DCAR_019837*	0.444	0.0118	0.0266
Dacar_LD3	*DCAR_019837*	*DCAR_019833*	7.5527	0.2034	0.0269
Dacar_WGD1	*DCAR_008603*	*DCAR_000606*	2.0932	0.1527	0.073
Dacar_WGD2	*DCAR_008603*	*DCAR_006920*	0.5291	0.0612	0.1157
Dacar_WGD3	*DCAR_013475*	*DCAR_009086*	48.9815	0.4642	0.0095
Dacar_WGD4	*DCAR_019837*	*DCAR_019793*	8.5851	0.1788	0.0208
Eugra_LD1	*Eucgr.A02435*	*Eucgr.A02437*	52.139	0.1876	0.0036
Eugra_LD2	*Eucgr.K01536*	*Eucgr.K01506*	75.4861	0.4086	0.0054
Eugra_WGD1	*Eucgr.D01859*	*Eucgr.D02276*	3.2588	0.2007	0.0616
Gmax_LD1	*Glyma.13G003100*	*Glyma.13G003800*	54.7309	0.5488	0.01
Gmax_LD2	*Glyma.10G092200*	*Glyma.10G091800*	3.6909	0.2183	0.0591
Gmax_WGD1	*Glyma.01G219200*	*Glyma.11G024300*	0.1484	0.0278	0.1872
Gmax_WGD10	*Glyma.11G024300*	*Glyma.18G230800*	2.753	0.2693	0.0978
Gmax_WGD11	*Glyma.03G142500*	*Glyma.11G130400*	1.7122	0.1068	0.0624
Gmax_WGD12	*Glyma.12G239600*	*Glyma.13G342700*	3.6839	0.2071	0.0562
Gmax_WGD13	*Glyma.12G239600*	*Glyma.15G031600*	4.4676	0.212	0.0475
Gmax_WGD14	*Glyma.19G145400*	*Glyma.15G271400*	9.4271	0.1868	0.0198
Gmax_WGD2	*Glyma.19G145400*	*Glyma.02G176700*	54.0627	0.2082	0.0039
Gmax_WGD3	*Glyma.03G142500*	*Glyma.10G091800*	0.7308	0.0473	0.0647
Gmax_WGD4	*Glyma.05G098700*	*Glyma.17G166900*	0.2274	0.021	0.0923
Gmax_WGD5	*Glyma.07G072500*	*Glyma.18G230800*	0.7396	0.1988	0.2688
Gmax_WGD6	*Glyma.07G089100*	*Glyma.09G187900*	0.1434	0.051	0.3559
Gmax_WGD7	*Glyma.12G001500*	*Glyma.08G239300*	0.2153	0.0218	0.1014
Gmax_WGD8	*Glyma.12G239600*	*Glyma.09G003400*	0.1209	0.0212	0.1755
Gmax_WGD9	*Glyma.07G072500*	*Glyma.09G261200*	0.7868	0.1893	0.2406
Gorai_LD1	*Gorai.006G141200*	*Gorai.006G140900*	5.5734	0.1789	0.0321
Gorai_WGD1	*Gorai.004G157900*	*Gorai.007G098200*	0.4827	0.0772	0.1599

Gorai_WGD2	*Gorai.004G210900*	*Gorai.007G098200*	2.2308	0.1755	0.0787
Gorai_WGD3	*Gorai.013G196200*	*Gorai.004G252500*	0.7014	0.1039	0.1481
Gorai_WGD4	*Gorai.006G140900*	*Gorai.004G174500*	1.796	0.1274	0.0709
Maesc_LD1	*Manes.09G031900*	*Manes.09G031600*	3.8199	0.1747	0.0457
Maesc_WGD1	*Manes.05G036600*	*Manes.01G259100*	0.3497	0.0488	0.1397
Maesc_WGD2	*Manes.05G036600*	*Manes.02G022800*	2.0215	0.1427	0.0706
Maesc_WGD3	*Manes.18G104300*	*Manes.02G196900*	0.4024	0.0696	0.1731
Maesc_WGD4	*Manes.14G075000*	*Manes.06G095700*	0.4655	0.0894	0.1921
Maesc_WGD5	*Manes.10G148000*	*Manes.06G168900*	3.3985	0.1765	0.0519
Maesc_WGD6	*Manes.14G014300*	*Manes.06G168900*	0.3545	0.0279	0.0786
Maesc_WGD7	*Manes.06G168900*	*Manes.09G031600*	3.3749	0.1918	0.0568
Maesc_WGD8	*Manes.08G152500*	*Manes.09G139300*	47.857	0.4342	0.0091
Md_LD1	*MDP0000190183*	*MDP0000190184*	49.6193	1.3948	0.0281
Md_LD2	*MDP0000278649*	*MDP0000284750*	5.6231	0.197	0.035
Md_LD3	*MDP0000129945*	*MDP0000733194*	49.6192	1.3949	0.0281
Md_WGD1	*MDP0000637757*	*MDP0000412954*	0.1623	0.0306	0.1888
Md_WGD2	*MDP0000717179*	*MDP0000322075*	0.2006	0.0569	0.2837
Medtr_LD1	*Medtr6g028050*	*Medtr6g028070*	0.1672	0.1015	0.6071
Medtr_WGD1	*Medtr7g076900*	*Medtr8g031270*	1.2032	0.2057	0.171
Musac_WGD1	*Medtr6g084400*	*Medtr8g104330*	1.0309	0.1277	0.1239
Musac_WGD1	*GSMUA_Achr1G00330*	*GSMUA_AchrUn_randomG06200*	1.0309	0.1277	0.1239
Musac_WGD2	*GSMUA_Achr1G06800*	*GSMUA_Achr2G03960*	1.1977	0.168	0.1403
Musac_WGD3	*GSMUA_Achr3G11130*	*GSMUA_Achr9G27720*	0.8755	0.0809	0.0925
Musac_WGD4	*GSMUA_Achr10G10540*	*GSMUA_Achr3G22190*	1.015	0.0775	0.0763
Potri_LD1	*Potri.013G066400*	*Potri.013G066500*	0.0799	0.0116	0.1454
Potri_WGD1	*Potri.001G015700*	*Potri.001G271400*	3.3193	0.1764	0.0531
Potri_WGD10	*Potri.006G058900*	*Potri.016G049100*	0.1972	0.0294	0.149
Potri_WGD11	*Potri.006G058700*	*Potri.016G049300*	0.2293	0.0367	0.1601
Potri_WGD12	*Potri.009G065600*	*Potri.016G049300*	1.4182	0.125	0.0881
Potri_WGD13	*Potri.006G098700*	*Potri.016G113800*	0.3751	0.0687	0.1831
Potri_WGD14	*Potri.013G107700*	*Potri.019G080000*	0.2269	0.0632	0.2787
Potri_WGD2	*Potri.001G105400*	*Potri.002G158000*	1.7581	0.1484	0.0844
Potri_WGD3	*Potri.002G158000*	*Potri.003G125900*	1.7188	0.1584	0.0921
Potri_WGD4	*Potri.001G271400*	*Potri.006G058700*	1.4488	0.1254	0.0866
Potri_WGD5	*Potri.006G058700*	*Potri.009G065600*	1.5257	0.1346	0.0882
Potri_WGD6	*Potri.004G043000*	*Potri.011G052000*	0.216	0.0618	0.2863
Potri_WGD7	*Potri.001G105400*	*Potri.014G081700*	1.659	0.1505	0.0907
Potri_WGD8	*Potri.001G271400*	*Potri.016G049100*	7.3578	0.1675	0.0228
Potri_WGD9	*Potri.003G210800*	*Potri.016G049100*	1.7317	0.096	0.0554
Sb_LD1	*Sobic.010G191800*	*Sobic.010G192500*	53.4339	0.1828	0.0034
Sb_WGD1	*Sobic.009G148000*	*Sobic.006G280300*	26.2389	0.1996	0.0076
Si_LD1	*Seita.4G215700*	*Seita.4G216000*	66.3081	0.1873	0.0028
Solyc_LD1	*Solyc10g080420*	*Solyc10g080430*	0.3346	0.0239	0.0713
Solyc_LD2	*Solyc01g086700*	*Solyc01g086720*	7.2492	0.2903	0.04

Solyc_WGD1	*Solyc01g086700*	*Solyc10g078680*	3.0349	0.2117	0.0697
Solyc_WGD2	*Solyc10g078680*	*Solyc10g080420*	0.5865	0.0362	0.0618
Sotub_LD1	*PGSC0003DMP400061360*	*PGSC0003DMP400016351*	0	0	0.001
Sotub_LD2	*PGSC0003DMP400041033*	*PGSC0003DMP400040957*	0.3124	0.0244	0.0781
Sotub_LD3	*PGSC0003DMP400043834*	*PGSC0003DMP400058879*	8.5442	0.3055	0.0358
Sotub_WGD1	*PGSC0003DMP400020249*	*PGSC0003DMP400040957*	0.63	0.0406	0.0645
Spoly_LD1	*Spipo1G0013600*	*Spipo1G0012200*	15.6212	0.4684	0.03
Spoly_WGD1	*Spipo10G0020900*	*Spipo12G0036300*	2.2062	0.1114	0.0505
Spoly_WGD2	*Spipo28G0004600*	*Spipo10G0020900*	41.5619	0.1868	0.0045
Theco_LD1	*Thecc1EG005920*	*Thecc1EG005933*	7.7901	0.3688	0.0473
Theco_LD2	*Thecc1EG024857*	*Thecc1EG024864*	3.9956	0.1788	0.0447
Theco_WGD1	*Thecc1EG016238*	*Thecc1EG005361*	1.641	0.1573	0.0959
Theco_WGD2	*Thecc1EG024857*	*Thecc1EG005920*	6.5245	0.3624	0.0555
Zm_LD1	*Zm00001d036801*	*Zm00001d036804*	53.9676	0.1602	0.003
Zm_WGD1	*Zm00001d026650*	*Zm00001d001785*	0.1535	0.0222	0.1444
Zm_WGD2	*Zm00001d006371*	*Zm00001d021494*	0.4196	0.049	0.1168
Zm_WGD3	*Zm00001d036801*	*Zm00001d046749*	0.2409	0.0225	0.0935
Zm_WGD4	*Zm00001d038340*	*Zm00001d010202*	0.2531	0.0178	0.0702

Table S4. Primer sequences used for RT-qPCR analysis.

Gene name	Forward primer (5'-3')	Reverse primer (5'-3')
ZmActin1	GGGATTGCCGATCGTATGAG	GAGCCACCGATCCAGACACT
GADPH	CTTCGGCATTGTTGAGGGTTTG	TCCTTGGCTGAGGGTCCGTC
ZmMCTP1	GCAAGTGGTTCAGCGGTGTC	GGTTGCGGCTGGTAGGGAAT
ZmMCTP2	GGTGGCGAAGTGGATGGAG	CAGGAACACGGTGGGCAG
ZmMCTP3	TAGTGCTGGTGGCAGAATGG	GTACCACAGCTTGGGCGTAA
ZmMCTP4	AGCCAACTCCATCTCAAATCC	GCTGCTGCTGGTCTCCGTA
ZmMCTP5	CGGTACGACAGGCTGAGGA	GAACGGTGCCAGGAGGGT
ZmMCTP6	AAGGGTGCCAAGGGACAAT	CTCGCTAACACGCTCATACGG
ZmMCTP7	AGTGCGGTCCCATCTTCCC	AACAGCAGGTCCTCGTTCCAG
ZmMCTP8	CCGTCATCACTGTAGGTGTGTTTG	GCAGCATCAGCAGAGGGTAG
ZmMCTP9	CGAAGCCTCTGTGGAAACCT	TGAAGCTGTCGATTATAGTCCTAGT
ZmMCTP10	GTGCGCTTCACCTGCCTGT	GCATGTACTCCACCACCTCCC
ZmMCTP11	ACGCACCACAGACGCCTATT	CCTCCCAAGTGTACTGCTCATTC
ZmMCTP12	GCCGCCTGATGCTACTGCTA	CCGTCATTACTTGTGCGTCTTC
ZmMCTP13	CCTCGAAGGCGGCTACCAT	GCATCACAGGACCCACGG
ZmMCTP14	AAGGATGCGCTACGACAGGC	CGACCACCAGGGACAAGAAA
ZmMCTP15	GAGGGCATCGTCTACTTTCCG	CATCATTGCAGGCTCATCGT
ZmMCTP16	ACCGCTGCGGAAGGAAATCG	CCACGGCAAACAGGGGGCTC
ZmMCTP17	TCCGCTACCGTCCTCGTTAT	GCTGGTCGGGAATGTATCAA
